# Exosomes from osteoarthritic fibroblast-like synoviocytes promote cartilage ferroptosis and damage via delivering microRNA-19b-3p to target SLC7A11 in osteoarthritis

**DOI:** 10.3389/fimmu.2023.1181156

**Published:** 2023-08-24

**Authors:** Ruina Kong, Lianmei Ji, Yafei Pang, Dongbao Zhao, Jie Gao

**Affiliations:** ^1^ Department of Rheumatology and Immunology, Changhai Hospital, Naval Medical University, Shanghai, China; ^2^ Department of Rheumatology, Shanghai Baoshan District Hospital of Integrated Traditional Chinese and Western Medicine, Shanghai, China

**Keywords:** osteoarthritis, ferroptosis, fibroblast-like synoviocytes, exosomes, miR-19b-3p

## Abstract

**Objective:**

Our previous studies revealed that normal synovial exosomes promoted chondrogenesis, and microRNA (miR)-19b-3p independently related to osteoarthritis (OA) risk. Subsequently, this study intended to further explore the effect of OA fibroblast-like synoviocyte (OA-FLS) exosomal miR-19b-3p on OA ferroptosis and its potential mechanisms.

**Methods:**

Interleukin (IL)-1β-stimulated chondrocytes and medial meniscus surgery were used to construct the OA cellular model and the OA rat model, respectively. OA-FLS exosomes with/without miR-19b-3p modification were added to the IL-1β-stimulated chondrocytes and OA rat models, followed by direct miR-19b-3p mimic/inhibitor transfection with/without SLC7A11 overexpression plasmids. miR-19b-3p, ferroptosis-related markers (malondialdehyde (MDA), glutathione (GSH)/oxidized glutathione (GSSG), ferrous ion (Fe^2+^), glutathione peroxidase 4 (GPX4), solute carrier family 7 member 11 (SLC7A11), and acyl-CoA synthetase long-chain family member 4 (ACSL4)), mitochondrial membrane potential (MMP), and reactive oxygen species (ROS) levels were detected.

**Results:**

Enhanced ferroptosis reflected by dysregulated ferroptosis-related markers, a reduced MMP, and an increased ROS was observed in cartilage tissues from OA patients vs. controls, IL-1β-stimulated chondrocytes vs. normal ones, and OA rat models vs. sham, so did miR-19b-3p. OA-FLS exosomes promoted MDA, Fe^2+^, ACSL4, and ROS but reduced cell viability, GSH/GSSG, GPX4, SLC7A11, and MMP in IL-1β-stimulated chondrocytes, whose effect was enhanced by miR-19b-3p mimics and attenuated by miR-19b-3p inhibitors. miR-19b-3p negatively regulated SLC7A11 and directly bound to SLC7A11 via luciferase reporter gene assay. Furthermore, SLC7A11 overexpression weakened miR-19b-3p mimics’ effect on ferroptosis-related markers, MMP, or ROS in IL-1β-stimulated chondrocytes. OA-FLS exosomes also induced cartilage damage and ferroptosis in OA rats whose influence was tempered by miR-19b-3p inhibitors.

**Conclusion:**

OA-FLS exosomal miR-19b-3p enhances cartilage ferroptosis and damage by sponging SLC7A11 in OA, indicating a potential linkage among synovium, cartilage, and ferroptosis during the OA process.

## 1 Introduction

Osteoarthritis (OA) is a common chronic, degenerative, and inflammatory disease affecting a vast number of populations worldwide, especially in elderly ones (1, 2). According to a recent electronic database analysis, the crude OA incidence has increased by 102% during the recent 30 years (3). Even though various treatments have been proposed for OA management, such as traditional ones [lifestyle control, physical therapy, nonsteroidal anti-inflammatory drugs (NSAIDs), and glucosamine] (4, 5) and novelty emerging ones (targeted therapy, gene-based therapy, and cell therapy) (6–8), the general outcomes of OA patients are still not satisfactory and the disease-related burden is still high along the lifetime (9). Therefore, the ongoing exploration of the deep pathogenesis of OA is necessary to discover more effective treatment methods further.

Synovial dysregulation is increasingly accepted as a critical factor accelerating OA development and progress in multiple ways (10–15). For instance, synovial fibrosis relates to OA pain, hyperalgesia, and stiffness by regulating neural sprouting, pannus shaping, and extracellular matrix changing (10). Synovial macrophage M1 polarization promotes inflammation and facilitates the impairment but hinders the regeneration of cartilages (11), targeting which has been explored as a potential treatment option recently (12). Synovial inflammation would then promote radiographic and pain progression of OA joints (13). In addition, OA fibroblast-like synoviocytes (OA-FLSs) break the balance of homeostatic and hypertrophic chondrocytes (14, 15).

Since first proposed in 2012, ferroptosis has attracted much attention in various fields (16–19). Ferroptosis is a new and unique mode of cell death involving redox homeostasis, iron handling, mitochondrial activity, and metabolism in an iron-dependent way (16, 20). Regarding ferroptosis’s implication in OA, the related shreds of evidence are insufficient. A recent study observes the accumulation of iron in cartilage and synovial fluid during OA progress and a reduction of GPX4 and GSH expressions (21). Another recent study reports that ferroptosis facilitates OA progression and promotes inflammation and matrix metalloproteinase 13 (MMP-13) expression (22). Moreover, the latest review comprehensively analyzes the potential relationship between ferroptosis and OA, while direct evidence is deficient (23).

Our previous studies discovered that normal synovial mesenchymal stem cells promoted chondrogenesis via transmitting exosomes (24) and microRNA (miR)-19b-3p independently related to OA susceptibility and severity (25). Subsequently, the current study aimed to further investigate the effect of OA-FLS exosomal miR-19b-3p on the ferroptosis of OA *in vitro* and *in vivo*, as well as its potential mechanisms.

## 2 Methods

### 2.1 Collection and detection of human samples

Articular samples were collected from 10 knee OA patients who underwent knee arthroplasty surgery and 10 controls who suffered trauma. The cartilage tissues were isolated to detect MDA, GSH/GSSG, Fe^2+^, GPX4, ACSL4, SLC7A11, and miR-19b-3p levels. The Ethics Committee of Changhai Hospital, Naval Medical University, approved the study, and all participants signed the informed consent.

### 2.2 Isolation of FLS and chondrocytes

The FLSs were isolated from the knee synovium of OA patients (OA patients-FLS) and controls (Controls-FLS) (26), and the chondrocytes were isolated from cartilage tissues from controls as described previously (27). The FLSs and chondrocytes were both cultured in Dulbecco’s modified Eagle’s medium (DMEM) (Gibco, USA) plus 10% fetal bovine serum (Gibco, USA) and penicillin/streptomycin (Sangon, China) at 37°C in 5% CO_2_. The level of miR-19b-3p in the FLS was assessed by reverse transcription-quantitative polymerase chain reaction (RT-qPCR).

### 2.3 Isolation and identification of FLS exosomes

The FLS exosomes were isolated using Hieff^®^ Quick exosome isolation kit (Yeasen, China) according to the kit’s protocol. The exosomes were quantified by a bicinchoninic Acid (BCA) kit (Sangon, China). Nanoparticle tracking analysis (NTA) was carried out by the Nanosight NS300 (Malvern, UK). The marker proteins of exosomes were analyzed by Western blot, and the medium without culturing with FLS served as negative control (NC). The miR-19b-3p expression in the exosomes of OA patients-FLS and Controls-FLS was assessed by RT-qPCR.

### 2.4 Chondrocytes cocultured with OA-FLS exosomes

The chondrocytes were seeded in six-well plates (4 × 10^5^ cells/well) and stimulated with IL-1β (10 ng/mL; MCE, China) for 24 h. The chondrocytes were treated with OA-FLS exosomes (100 μg/mL), ferrostatin-1 (Fer-1; 1 μM; MCE, China), or their combination for 48 h. Further assays including RT-qPCR (miR-19b-3p expression), cell counting kit-8 (CCK-8) (cell viability), and ferroptosis-related detections [MDA, GSH/GSSG, Fe^2+^, mitochondrial membrane potential (MMP), ROS, GPX4, ACSL4, and SLC7A11] were performed.

### 2.5 Chondrocytes cocultured with exosomes of miR-19b-3p modified OA-FLS

The miR-19b-3p mimics, miR-19b-3p inhibitors, and negative control (NC mimics or NC inhibitors) (Generay, China) were transfected into OA-FLS using Lipofectamine^®^ 3000 (Invitrogen, USA) according to the kit’s instructions. Afterward, each group’s exosomes (100 μg/mL) were isolated to treat IL-1β-stimulated chondrocytes. The IL-1β-stimulated chondrocytes without exosome treatment were set as control. RT-qPCR, CCK-8, and ferroptosis-related detections were performed after 48-h treatment.

### 2.6 miR-19b-3p regulation experiment in chondrocytes

The chondrocytes were transfected with miR-19b-3p mimics, miR-19b-3p inhibitors, and negative control (NC mimics or NC inhibitors), as mentioned above, using Lipofectamine^®^ 3000. Cells were then stimulated with IL-1β for 24 h. After being cultured for another 48 h, cells were collected for RT-qPCR, CCK-8, and ferroptosis-related detections.

### 2.7 SLC7A11 regulation experiment in chondrocytes

Luciferase reporter gene assay was performed to study transcription regulation between miR-19b-3p and SLC7A11. Briefly, the wild-type (WT) and mutant-type (MUT) plasmids of SLC7A11 were constructed with pGL6-TA (Beyotime, China). The SLC7A11 WT and MUT plasmids were cotransfected with miR-19b-3p or NC mimics into 293T cells (ATCC, USA). After 48-h culture, the fluorescence intensity was detected in the presence of Luciferase Reporter Gene Assay Kit (Beyotime, China). For the SLC7A11 regulation experiment, the chondrocytes were transfected with miR-19b-3p mimics, SLC7A11 overexpression plasmids (Generay, China), or their combination using Lipofectamine^®^ 3000. The chondrocytes transfected with NC mimics and scramble plasmids (Generay, China) served as the NC group. Transfected cells were then cultured and stimulated with IL-1β for 24 h. Further assays, including RT-qPCR, CCK-8, and ferroptosis-related detections, were performed after 48-h culture.

### 2.8 Animal research

The animal experiments were approved by the Animal Care and Use Committee and by the guidelines of our institute. The Sprague–Dawley (SD) rats (8 weeks old) (SLAC, China) were caged in a controlled temperature (25°C ± 1°C) and humidity (60% ± 5%). The medial meniscus (DMM) surgery was destabilized to create experimental OA rats, as described previously (28). After 4 weeks, the FLS of OA rats (OA rats-FLS) was isolated, cultured, and transfected with miR-19b-3p inhibitors or NC inhibitors. Then, the exosomes of OA-FLS (OA rats-FLS Exo) were collected. The RT-qPCR was performed to detect the miR-19b-3p expression of OA rats-FLS and OA rats-FLS exosomes.

Meanwhile, 30 rats were used to study the influence of OA rats-FLS exosomes in the OA models. Among them, 24 rats received DMM surgery to establish the OA model, and the others served in the Sham group (n = 6) without surgery. After 4 weeks, the OA rats were divided into the Model group, Exo group, Exo+Fer-1 group, and miR(-) Exo group (n = 6 per group). The Sham and Model group rats were given articular cavity injections of phosphate buffer saline (PBS). The rats in the Exo group were given articular cavity injections of 100-μg exosomes from OA rats-FLS (transfected with NC inhibitors). The rats in the Exo+Fer-1 group were given articular cavity injections of 100-μg exosomes from OA rats-FLS (transfected with NC inhibitors) and Fer-1 (0.1 mg/kg). The miR(-) Exo group rats were given articular cavity injections of 100-μg exosomes from OA rats-FLS (transfected with miR-19b-3p inhibitors). The injections were carried out twice a week for four consecutive weeks. The rats were euthanized after treatment, and the cartilage samples were collected for safranin-O and fast green (SOFA) staining, hematoxylin and eosin (H&E) staining, immunohistochemical (IHC) staining, and the detection of miR-19b-3p, MDA, GSH/GSSG, and Fe^2+^ level.

### 2.9 MDA, GSH/GSSG, and Fe^2+^ detection

The cartilage tissues and chondrocytes were lysed using Cell lysis buffer (Solarbio, China) and detected with BCA kit (Servicebio, China). The MDA and GSH concentration were detected using MDA Assay Kit (Beyotime, China) or GSH/GSSG Assay Kit (Beyotime, China) and normalized based on the protein concentration. The Fe^2+^ detection was performed using Iron Assay Kit (Dojindo, Japan) according to the kit’s instructions.

### 2.10 Western blot

Total protein from cartilage tissues, FLS exosomes, and chondrocytes was isolated using radio immunoprecipitation assay (RIPA) (Beyotime, China). The protein was separated using sulfate-polyacrylamide gel electrophoresis (SDS-PAGE) precast gel (Willget, China) and transferred to nitrocellulose membranes (Pall, USA). Then, the membranes were incubated with anti-GPX4 (1:1,000; Affinity, China), anti-ACSL4 (1:1,000; Affinity, China), anti-SLC7A11 (1:1,000; Affinity, China), anti-CD9 (1:1,000; Affinity, China), anti-CD63 (1:1,000; Affinity, China), anti-CD81 (1:1,000; Affinity, China), anti-TSG101 (1:1,000; Affinity, China), or anti-β-actin (1:5,000; Abcam, UK) primary antibodies. After incubating with a secondary antibody (1:5,000; Affinity, China), the blots were visualized using ECL Kit (Beyotime, China).

### 2.11 RT-qPCR

Total RNA from cartilage tissues, FLSs, and chondrocytes was isolated with RNA Purification Kit (Takara, China). The RNA of FLS exosomes was extracted using an Exosome RNA Isolation Kit (Invitrogen, USA). The RT-qPCR was performed using Reverse Transcription Kit (Takara, China) and qPCR Mix (Takara, China) according to the kit’s instructions. The miR-19b-3p and SLC7A11 expression was assessed using the 2^-ΔΔCt^ method and normalized to U6 or β-actin. The primer sequences used in the study were as follows (5’–3’): miR-19b-3p (forward, TCTACAGGTGTGCAAATCCATG; reverse, TGTCGTGGAGTCGGCAATTC); U6 (forward, CGCTTCGGCAGCACATATACTA; reverse, ATGGAACGCTTCACGAATTTGC); SLC7A11 (forward, TGCTGGGCTGATTTTATCTTCG; reverse, GAAAGGGCAACCATGAAGAGG); β-actin (forward, TGGTATCGTGGAAGGACTC; reverse, AGTAGAGGCAGGGATGATG).

### 2.12 Cell viability assay

The cell viability of chondrocytes was assessed in the presence of a CCK-8 kit (Yeason, China). In brief, treated chondrocytes were incubated with CCK-8 reagent at 37°C for 2 h. The optical density at 450 nm was then assessed by a microplate reader (Molecular Devices, USA).

### 2.13 MMP and ROS detection

The MMP of chondrocytes was determined using MMP Assay Kit with JC-1 (Beyotime, China). The ROS level of chondrocytes was assessed using ROS Assay Kit (Beyotime, China). The images were taken under a fluorescent microscope (Olympus, Japan).

### 2.14 SOFA, H&E, and IHC staining

The knee joints were fixed in 4% paraformaldehyde and decalcified with 20% ethylene diamine tetraacetic acid (EDTA). The tissues were embedded in paraffin and cut into sections. The SOFA and H&E staining were performed using Safranin O/Fast green Kit (Solarbio, China) or Hematoxylin-Eosin Staining Kit (Beyotime, China). The Osteoarthritis Research Society Internationa (OARSI) score was used to analyze the severity of cartilage degeneration (29). For IHC staining, the sections were dewaxed and treated with Antigen Retrieval Solution (Beyotime, China). The sections were incubated successfully with blocking buffer (Beyotime, China), antibodies, and diaminobenzidine (DAB) staining solution (Beyotime, China). The antibodies used in IHC staining included anti-MMP-13 (1:200; Affinity, China), anti-Collagen II (1:100; Invitrogen, USA), anti-GPX4 (1:100; Affinity, China), anti-ACSL4 (1:200; Affinity, China), anti-SLC7A11 (1:100; Affinity, China), and Goat Anti-Rabbit IgG (H+L) HRP antibody (1:500; Affinity, China).

### 2.15 Statistical analysis

Data were analyzed by unpaired t-test or one-way ANOVA followed by Tukey’s multiple comparison tests using GraphPad Prism 7.01 (GraphPad, USA). P < 0.05 was considered statistically significant.

## 3 Results

### 3.1 Ferroptosis markers and miR-19b-3p in OA patients versus controls

**Figure 1:**
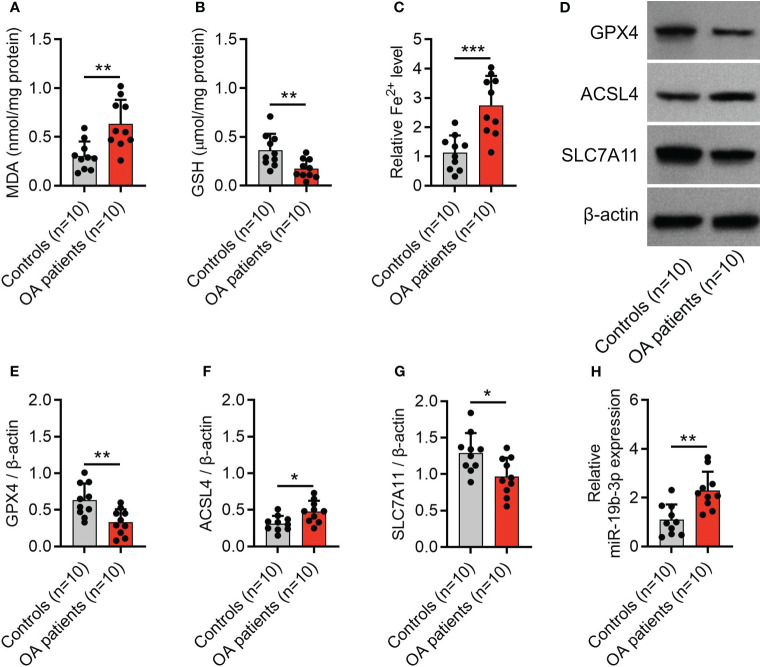
Enhanced ferroptosis in OA patients. Levels of MDA **(A)**, GSH **(B)**, Fe^2+^ **(C)**, western blots images **(D)** and quantified expressions of GPX4 **(E)**, ACSL4 **(F)**, and SLC7A11 **(G)** in cartilage tissues between OA patients and controls. MiR-19b-3p expression **(H)** in cartilage tissues between OA patients and controls. There were 10 controls and 10 OA patients enrolled. The error bars stood for the standard deviation. The unpaired t-test was applied for the comparison tests. * stand for P<0.05, ** stand for P<0.01, *** stand for P<0.001. OA, osteoarthritis; MDA, malondialdehyde; GSH, glutathione; Fe2+, ferrous ion; GPX4, glutathione peroxidase 4; ACSL4, acyl-CoA synthetase long-chain family member 4; SLC7A11, solute carrier family 7 member 11; MiR, microRNA.

The MDA concentration (P < 0.01) and Fe^2+^ level (P < 0.001) were elevated, while the GSH concentration (P < 0.01) was reduced in cartilage tissues from OA patients compared with controls (**Figures 1A–C**). Meanwhile, the expression of GPX4 (P < 0.01) and SLC7A11 (P < 0.05) was decreased, but the expression of ACSL4 (P < 0.05) and miR-19b-3p were increased in cartilage tissues from OA patients compared with controls (**Figures 1D–H**). These indicated an enhanced ferroptosis in OA patients vs. controls. Notably, it was also observed that miR-19b-3p was higher in the cartilage tissues of OA patients compared with that of controls.

### 3.2 Effect of OA-FLS exosomes on ferroptosis in IL-1β-stimulated chondrocytes

FLSs were isolated and observed under the microscope for morphology (**Supplementary Figure S1A**), then miR-19b-3p was discovered to be higher in OA-FLS compared to control-FLS (P < 0.001) (**Supplementary Figure S1B**). Afterward, the FLS exosomes were extracted, their size distribution mainly within 50–150 nm (**Supplementary Figure S1C**), and the exosome markers (CD9, CD63, CD81, and TSG101) were detected for validation of exosomes (**Supplementary Figure S1D**). Finally, it was also found that miR-19b-3p was higher in OA-FLS exosomes compared with control-FLS exosomes (P < 0.001) (**Supplementary Figure S1E**).

**Figure 2:**
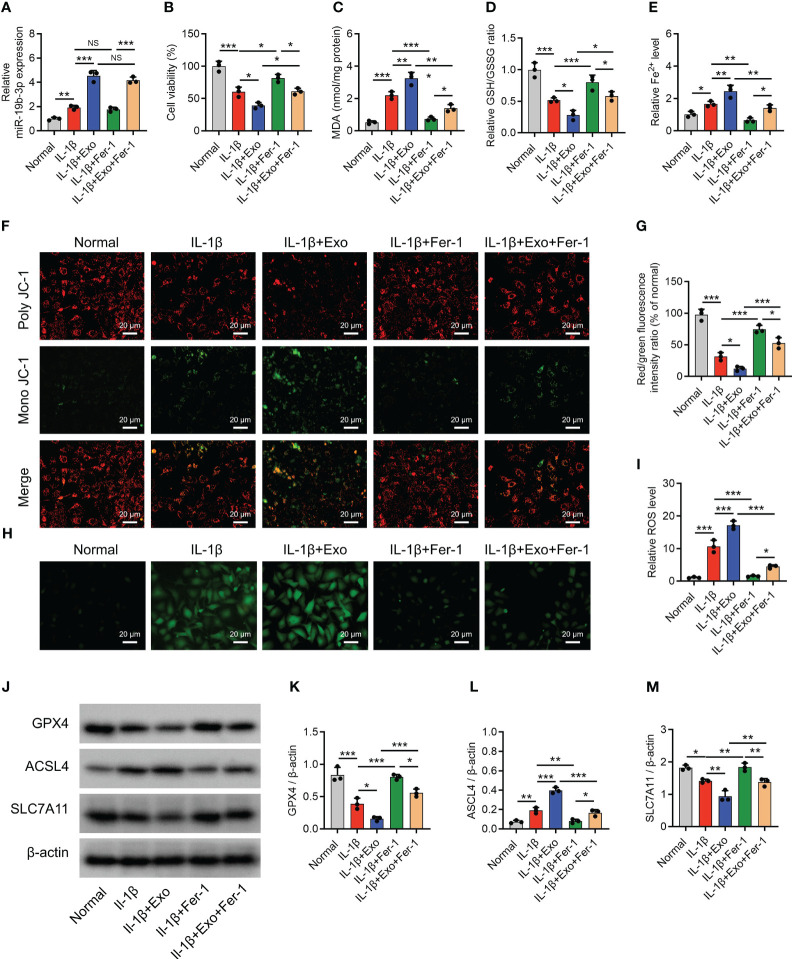
OA-FLS exosomes promoted chondrocyte ferroptosis. MiR-19b-3p expression **(A)**, cell viability **(B)**, levels of MDA **(C)**, GSH **(D)**, Fe^2+^ **(E)**, poly JC-1 and mono JC-1 staining images **(F)** and quantification **(G)**, ROS staining **(H)** and quantification **(I)**, western blots images **(J)** and quantified expressions of GPX4 **(K)**, ACSL4 **(L)**, SLC7A11 **(M)**, among Normal, IL-1β, IL-1β+Exo, IL-1β+Fer-1, and IL-1β+Exo+Fer-1 groups. Triple repetitions were carried out for each experiment. The error bars stood for the standard deviation. The one-way ANOVA followed by Tukey’s multiple comparison tests was carried out for the comparison test. * stand for P<0.05, ** stand for P<0.01, *** stand for P<0.001. OA, osteoarthritis; FLS, fibroblast-like synoviocytes; MiR, microRNA; MDA, malondialdehyde; GSH, glutathione; GSSG, oxidized glutathione; Fe^2+^, ferrous ion; GPX4, glutathione peroxidase 4; ACSL4, acyl-CoA synthetase long-chain family member 4; SLC7A11, solute carrier family 7 member 11; JC-1, 5,5',6,6'-tetrachloro-1,1',3,3'-tetraethylbenzimidazolylcarbocyanine iodide; ROS, reactive oxygen species; IL, interleukin; Exo, exosome; Fer-1, ferrostatin-1; ANOVA, one-way analysis of variance.

After IL-1β stimulation in chondrocytes to mimic the OA cellular model, miR-19b-3p was elevated (P < 0.05) (**Figure 2A**), but cell viability was reduced (P < 0.001) (**Figure 2B**). Besides, MDA concentration and Fe^2+^ level were increased, while GSH/GSSG concentration was decreased after IL-1β stimulation (all P<0.05) (**Figures 2C–E**). Subsequently, MMP and ROS levels were detected, showing that after IL-1β stimulation: poly JC-1 was dissociated into mono JC-1 (P<0.001) (**Figures 2F, G**), and the ROS level was raised (P<0.001) (**Figures 2H, I**). These suggested an induced ferroptosis by IL-1β stimulation in chondrocytes (OA cellular model). Moreover, the IL-1β stimulation in chondrocytes could mimic the OA phenotype. Furthermore, it was observed that GPX4, SCL7A11 were decreased, while ASCL4 was elevated after IL-1β stimulation (all P<0.05) (**Figures 2J–M**).

Interestingly, OA-FLS exosomes further raised miR-19b-3p level (P < 0.001) (**Figure 2A**) and reduced cell viability (P < 0.05) (**Figure 2B**) in IL-1β-stimulated chondrocytes. Furthermore, OA-FLS exosomes further elevated the MDA concentration (P < 0.01), Fe^2+^ level (P < 0.05), and ACSL4 expression (P < 0.01) while cutting down the GSH/GSSG concentration (P < 0.05), GPX4 expression (P < 0.05), and SLC7A11 expression (P < 0.05) in IL-1β-stimulated chondrocytes (**Figures 2C–E**). Next, OA-FLS exosomes lowered the MMP (P < 0.05) (**Figures 2F, G**) but promoted the ROS level (P < 0.01) (**Figures 2H, I**) in IL-1β-stimulated chondrocytes. Notably, the effect of OA-FLS exosomes on the indexes mentioned above was attenuated by Fer-1 treatment in IL-1β-stimulated chondrocytes. These implied that OA-FLS exosomes accelerated ferroptosis in IL-1β-stimulated chondrocytes (OA cellular model).

### 3.3 Effect of miR-19b-3p-modified OA-FLS exosomes on ferroptosis in IL-1β-stimulated chondrocytes

miR-19b-3p was sharply elevated after miR-19b-3p mimic transfection (both P < 0.01) but reduced after miR-19b-3p inhibitor transfection (both P < 0.05) in both OA-FLS itself (**Supplementary Figure S2A**) and OA-FLS exosomes (**Supplementary Figure S2B**).

NC(-) exosomes increased the miR-19b-3p level (P < 0.001) (**Supplementary Figure S3A**) and reduced cell viability (P < 0.01) (**Supplementary Figure S3B**) in non-treated chondrocytes. Furthermore, NC(-) exosomes elevated the MDA concentration (P < 0.01) and Fe^2+^ level (P < 0.05) while reducing the GSH/GSSG ratio (P < 0.01) (**Supplementary Figures S3C–S3E**) in non-treated chondrocytes. Next, NC(-) exosomes lowered the MMP (P < 0.01) (**Supplementary Figures S3F, G**) but promoted the ROS level (P < 0.05) (**Supplementary Figures S3H, I**). Furthermore, NC(-) exosomes also lowered GPX4 and SLC7A11 expression (both P < 0.05) while enhancing the ACSL4 expression (P < 0.05) (**Supplementary Figures S3J–M**). Notably, the effect of NC(-) exosomes on the indexes mentioned above was attenuated by miR(-) exosome treatment in non-treated chondrocytes. These findings implied that the normal exosomes contained a certain level of miR-19b-3p level and its treatment could elevate the miR-19b-3p level in the non-treated chondrocytes, while the miR(-) exosome treatment could not alter the miR-19b-3p level in the non-treated chondrocytes.

**Figure 3:**
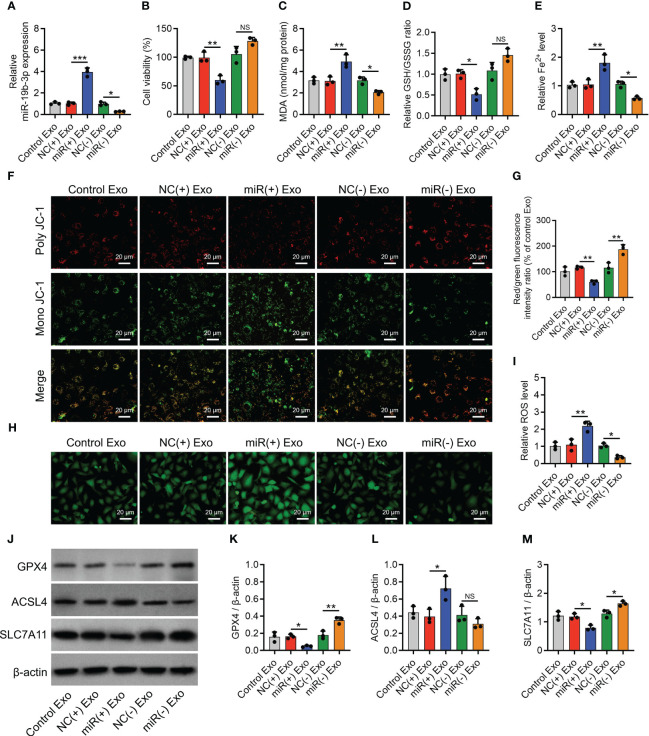
OA-FLS exosomal miR-19b-3p enhanced chondrocyte ferroptosis. MiR-19b-3p expression **(A)**, cell viability **(B)**, levels of MDA **(C)**, GSH **(D)**, Fe^2+^ **(E)**, poly JC-1 and mono JC-1 staining images **(F)** and quantification **(G)**, ROS staining **(H)** and quantification **(I)**, western blots images **(J)** and quantified expressions of GPX4 **(K)**, ACSL4 **(L)**, SLC7A11 **(M)**, among Control Exo, NC(+) Exo, miR(+) Exo, NC(-) Exo, and miR(-) Exo groups. Triple repetitions were carried out for each experiment. The error bars stood for the standard deviation. The one-way ANOVA followed by Tukey’s multiple comparison tests was carried out for the comparison test. * stand for P<0.05, ** stand for P<0.01, *** stand for P<0.001, NS stand for no significance. OA, osteoarthritis; FLS, fibroblast-like synoviocytes; MiR, microRNA; MDA, malondialdehyde; GSH, glutathione; GSSG, oxidized glutathione; Fe^2+^, ferrous ion; GPX4, glutathione peroxidase 4; ACSL4, acyl-CoA synthetase long-chain family member 4; SLC7A11, solute carrier family 7 member 11; JC-1, 5,5',6,6'-tetrachloro-1,1',3,3'-tetraethylbenzimidazolylcarbocyanine iodide; ROS, reactive oxygen species; Exo, exosome; ANOVA, one-way analysis of variance.

miR-19b-3p-modified OA-FLS exosomes were added to treat IL-1β-stimulated chondrocytes. It was discovered that miR-19b-3p mimic exosomes elevated miR-19b-3p expression (P < 0.001) (**Figure 3A**) but crippled cell viability (P < 0.01) (**Figure 3B**) compared to NC mimic exosomes in IL-1β-stimulated chondrocytes. MiR-19b-3p mimics exosomes also increased the MDA concentration (P<0.01) and Fe^2+^ level (P<0.01), while decreasing the GSH/GSSG concentration (P<0.05) compared with NC mimics exosomes in IL-1β-stimulated chondrocytes (**Figures 3C–E**). Moreover, miR-19b-3p mimics exosomes attenuated the MMP (P<0.01) (**Figures 3F, G**), then enhanced the ROS level (P<0.01) (**Figures 3H, I**) compared with NC mimics exosomes in IL-1β-stimulated chondrocytes. Besides, miR-19b-3p mimics exosomes also decreased the GPX4 and SCL7A11, while increased the ACSL4 level (all P<0.05) (**Figures 3J–M**) compared with NC mimics exosomes in IL-1β-stimulated chondrocytes. On the contrary, it was found that miR-19b-3p inhibitor exosomes exhibited the opposite effect as miR-19b-3p mimic exosomes did in IL-1β-stimulated chondrocytes, except that the miR-19b-3p mimic exosomes affected cell viability, GSH/GSSG concentration, and ACSL4 expression (**Figures 3A–M**). These manifested OA-FLS exosomal miR-19b-3p induced ferroptosis in IL-1β-stimulated chondrocytes (OA cellular model).

### 3.4 The interaction between miR-19b-3p and SLC7A11 and their effects on ferroptosis in IL-1β-stimulated chondrocytes

**Figure 4:**
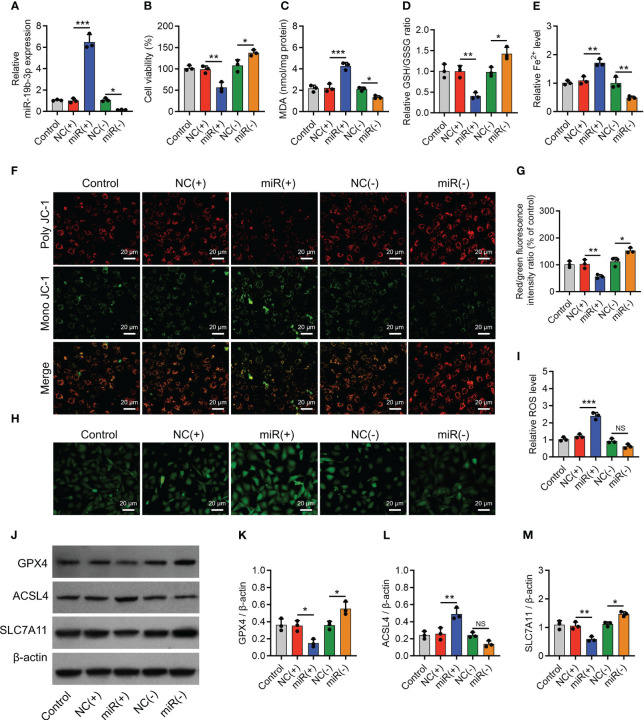
MiR-19b-3p directly facilitated chondrocyte ferroptosis. MiR-19b-3p expression **(A)**, cell viability **(B)**, levels of MDA **(C)**, GSH **(D)**, Fe^2+^ **(E)**, poly JC-1 and mono JC-1 staining images **(F)** and quantification **(G)**, ROS staining **(H)** and quantification **(I)**, western blots images **(J)** and quantified expressions of GPX4 **(K)**, ACSL4 **(L)**, SLC7A11 **(M)**, among Control, NC(+), miR(+), NC(-), and miR(-) groups. Triple repetitions were carried out for each experiment. The error bars stood for the standard deviation. The one-way ANOVA followed by Tukey’s multiple comparison tests was carried out for the comparison test. * stand for P<0.05, ** stand for P<0.01, *** stand for P<0.001, NS stand for no significance. MiR, microRNA; MDA, malondialdehyde; GSH, glutathione; GSSG, oxidized glutathione; Fe^2+^, ferrous ion; JC-1, 5,5',6,6'-tetrachloro-1,1',3,3'-tetraethylbenzimidazolylcarbocyanine iodide; ROS, reactive oxygen species; GPX4, glutathione peroxidase 4; ACSL4, acyl-CoA synthetase long-chain family member 4; SLC7A11, solute carrier family 7 member 11; ANOVA, one-way analysis of variance.

To further confirm the implication of miR-19b-3p in OA-FLS exosomes’ effect on ferroptosis and its potential molecular mechanism, direct miR-19b-3p mimics or inhibitors were transfected into IL-1β-stimulated chondrocytes. Then, it was observed that miR-19b-3p was correspondingly increased (P < 0.001) or decreased (P < 0.05) following the transfection of direct miR-19b-3p mimics or inhibitors in IL-1β-stimulated chondrocytes (**Figure 4A**). Subsequently, miR-19b-3p mimics suppressed the cell viability (P < 0.01) (**Figure 4B**) and heightened the MDA concentration (P < 0.001) and Fe^2+^ level (P < 0.01) but lowered the GSH/GSSG concentration (P < 0.01) in IL-1β-stimulated chondrocytes (**Figures 4C–E**). miR-19b-3p mimics also decreased the MMP (P < 0.01) (**Figures 4F, G**), while it increased the ROS level (P < 0.001) (**Figures 4H, I**) in IL-1β-stimulated chondrocytes. Furthermore, miR-19b-3p mimics decreased the expression of GPX4 (P < 0.05) and SLC7A11 (P < 0.01), but it increased the expression of ACSL4 (P < 0.01) in IL-1β-stimulated chondrocytes (**Figures 4J–M**). Reversely, the miR-19b-3p inhibitors showed the opposite effect as miR-19b-3p mimics functioned in IL-1β-stimulated chondrocytes, except that it did not regulate ACSL4 expression (**Figures 4A–M**). These suggested that miR-19b-3p directly enhanced ferroptosis in IL-1β-stimulated chondrocytes (OA cellular model).

Noteworthily, miR-19b-3p negatively regulated SLC7A11 in IL-1β-stimulated chondrocytes (**Figures 4J–M**), and miR-19b-3p was predicted to target SLC7A11 via the starBase database (starbase.sysu.edu.cn); therefore, the luciferase reporter gene assay was performed, which observed that miR-19b-3p directly bound to SLC7A11 at the predicted sequence (P < 0.001) (**Supplementary Figures S4A, B**).

**Figure 5:**
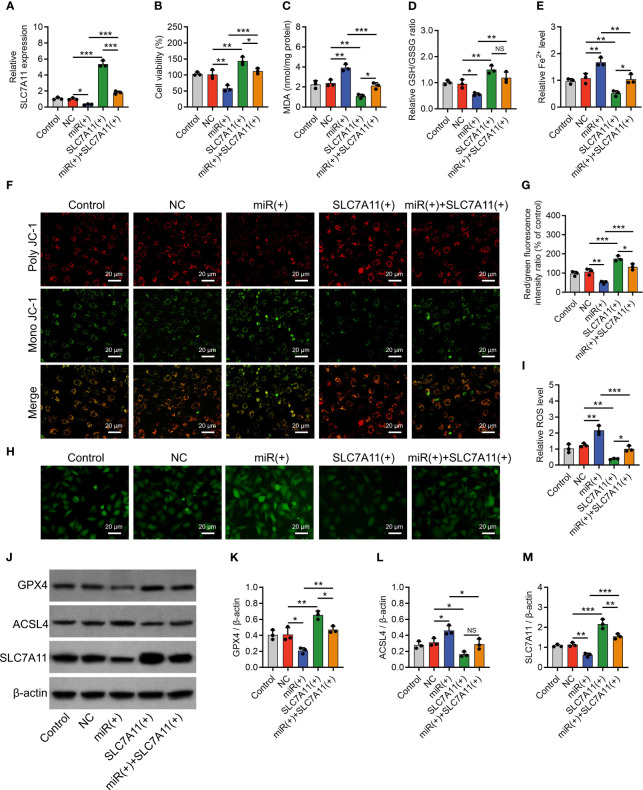
SLC7A11 compensated the effect of miR-19b-3p on chondrocyte ferroptosis. SLC7A11 mRNA expression **(A)**, cell viability **(B)**, levels of MDA **(C)**, GSH **(D)**, Fe^2+^ **(E)**, poly JC-1 and mono JC-1 staining images **(F)** and quantification **(G)**, ROS staining **(H)** and quantification **(I)**, western blots images **(J)** and quantified expressions of GPX4 **(K)**, ACSL4 (L), SLC7A11 **(M)**, among Control, NC, miR(+), SLC7A11(+), and miR(+)+SLC7A11(+) groups. Triple repetitions were carried out for each experiment. The error bars stood for the standard deviation. The one-way ANOVA followed by Tukey’s multiple comparison tests was carried out for the comparison test. * stand for P<0.05, ** stand for P<0.01, *** stand for P<0.001, NS stand for no significance. SLC7A11, solute carrier family 7 member 11; MiR, microRNA; MDA, malondialdehyde; GSH, glutathione; GSSG, oxidized glutathione; Fe2+, ferrous ion; JC-1, 5,5',6,6'-tetrachloro-1,1',3,3'-tetraethylbenzimidazolylcarbocyanine iodide; ROS, reactive oxygen species; GPX4, glutathione peroxidase 4; ACSL4, acyl-CoA synthetase long-chain family member 4; ANOVA, one-way analysis of variance.

Subsequently, SLC7A11 overexpression plasmids with or without miR-19b-3p mimics were transfected into IL-1β-stimulated chondrocytes, with SLC7A11 expression validated (**Figure 5A**). Then, SLC7A11 overexpression plasmids facilitated the cell viability (P < 0.01) (**Figure 5B**), repressed the MDA concentration (P < 0.01) and Fe^2+^ level (P < 0.01), and promoted GSH/GSSG concentration (P < 0.01) in IL-1β-stimulated chondrocytes (**Figures 5C–E**). In addition, SLC7A11 overexpression plasmids elevated the MMP (P < 0.001) (**Figures 5F, G**) and reduced the ROS level (P < 0.01) (**Figures 5H, I**); meanwhile, it enhanced the GPX4 (P < 0.01) and SLC7A11 (protein aspect) (P < 0.001) expressions, but it inhibited ACSL4 expression (P < 0.05) in IL-1β-stimulated chondrocytes (**Figures 5J–M**). It was worth noting that SLC7A11 overexpression plasmids also attenuated the effect of miR-19b-3p mimics on regulating the above ferroptosis-related indexes (**Figures 5B–M**). These implied that miR-19b-3p promoted ferroptosis via targeting SLC7A11 in IL-1β-stimulated chondrocytes (OA cellular model).

### 3.5 Effect of untransfected and miR-19b-3p knockdown OA-FLS exosomes on cartilage damage and ferroptosis

**Figure 6:**
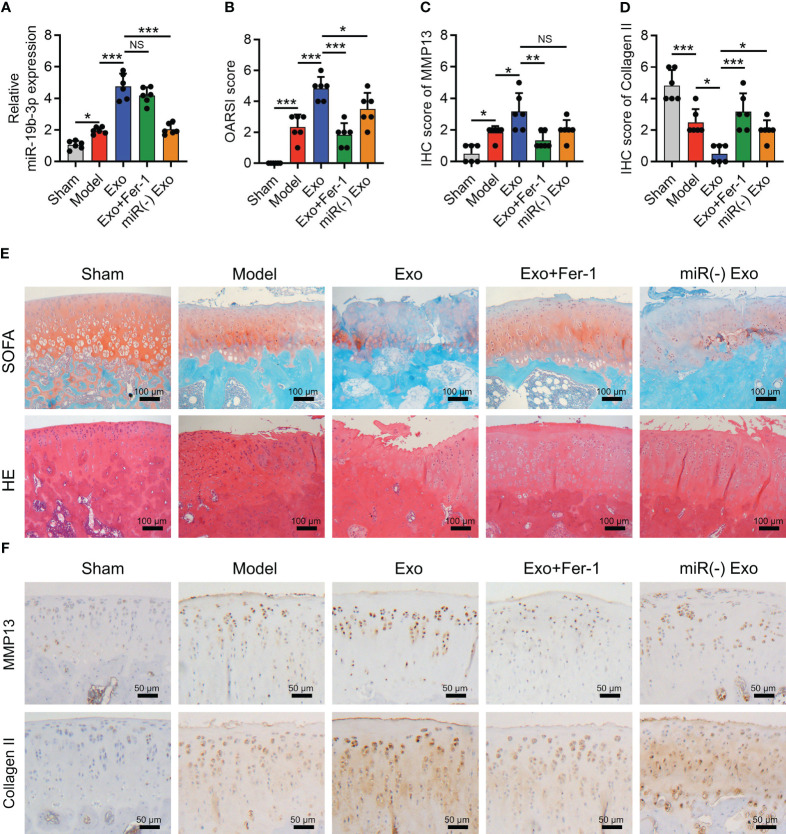
OA-FLS exosomes promoted cartilage damage by transmitting miR-19b-3p in OA rats. OARSI score **(A)**, SOFA staining and H&E staining **(B)**, quantified IHC score of MMP-13 **(C)**, quantified IHC score of Collagen II **(D)**, IHC staining images of MMP-13 and Collagen II **(E)**, and miR-19b-3p expression **(F)** among Sham, Model, Exo, Exo+Fer-1, and miR(-) Exo groups. There were six rats in each group. The error bars stood for the standard deviation. The one-way ANOVA followed by Tukey’s multiple comparison tests was carried out for the comparison test. * stand for P<0.05, ** stand for P<0.01, *** stand for P<0.001, NS stand for no significance. OA, osteoarthritis; FLS, fibroblast-like synoviocytes; OARSI, Osteoarthritis Research Society International; SOFA, safranin-O & fast green; HE, hematoxylin-eosin; IHC, immunohistochemical; MMP13, matrix metalloproteinase 13; MiR, microRNA; Exo, exosome; Fer-1, ferrostatin-1; ANOVA, one-way analysis of variance.

**Figure 7:**
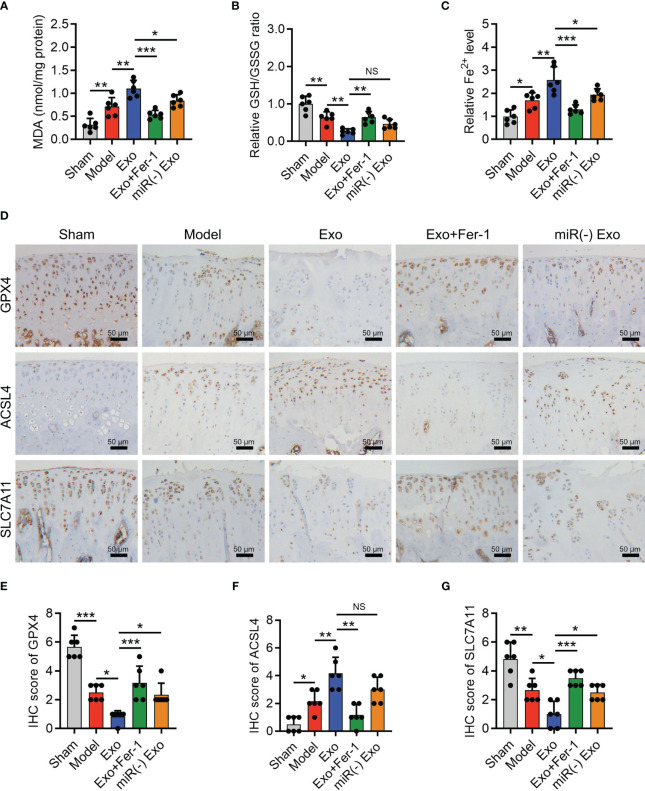
OA-FLS exosomes enhanced cartilage ferroptosis by transmitting miR-19b-3p in OA rats. Levels of MDA **(A)**, GSH **(B)**, Fe^2+^ **(C)**, IHC staining images **(D)** and quantified expressions of GPX4 **(E)**, ACSL4 **(F)**, SLC7A11 IHC scores **(G)**, among Sham, Model, Exo, Exo+Fer-1, miR(-) Exo groups. There were six rats in each group. The error bars stood for the standard deviation. The one-way ANOVA followed by Tukey’s multiple comparison tests was carried out for the comparison test. * stand for P<0.05, ** stand for P<0.01, *** stand for P<0.001, NS stand for no significance. OA, osteoarthritis; FLS, fibroblast-like synoviocytes; OARSI, Osteoarthritis Research Society International; SOFA, safranin-O & fast green; HE, hematoxylin-eosin; IHC, immunohistochemical; MMP13, matrix metalloproteinase 13; MiR, microRNA; Exo, exosome; Fer-1, ferrostatin-1; ANOVA, one-way analysis of variance.

The OARSI score (P < 0.001) reflected by SOFA staining, cartilage damage, and hypocellularity reflected by HE staining (**Figures 6A, B**) were elevated in the Model group compared to the Sham group; meanwhile, MMP-13 was increased (P < 0.05) but collagen II was decreased (P < 0.001) in the Model group compared with those in the Sham group (**Figures 6C–E**), indicating the success of OA rat model establishment. Interestingly, it was also observed that miR-19b-3p was higher in the Model group compared to that in the Sham group (P < 0.05) (**Figure 6F**). Meanwhile, the MDA concentration (P < 0.01) and Fe^2+^ level (P < 0.05) were raised but the GSH concentration (P < 0.01) was reduced in the Model group compared to those in the Sham group (**Figure 7A**). At the same time, the GPX4 (P < 0.001) and SLC7A11 (P < 0.01) expressions were suppressed but the ACSL4 expression (P < 0.05) was promoted in the Model group compared to those in the Sham group (**Figures 7B, C**). These manifested an activated cartilage ferroptosis in the OA rat models.

Next, FLSs from OA rats were isolated and transfected by NC inhibitors or miR-19b-3p inhibitors followed by exosome extraction. It was observed that miR-19b-3p expression was dramatically lower after transfection in both OA rats-FLS and OA rats-FLS exosomes (P < 0.001) (**Supplementary Figures S5A, B**), suggesting transfection success.

In OA rat models, OA-FLS exosomes increased OARSI score (P < 0.001) (**Figure 6A**), cartilage damage, and hypocellularity (**Figure 6B**), which also enhanced MMP-13 expression (P < 0.05) but reduced collagen II (P < 0.05) expression (**Figures 6C–E**) as well as miR-19b-3p expression (P < 0.001) (**Figure 6F**). Interestingly, the cartilage damage caused by OA-FLS exosomes was improved by Fer-1 treatment or miR-19b-3p inhibitor transfection (transfected into OA-FLS exosomes) (**Figures 6A–E**). These indicated that OA-FLS exosomes facilitated cartilage damage via delivering miR-19b-3p in OA rat models.

Furthermore, OA-FLS exosomes enhanced the MDA concentration (P < 0.01) and Fe^2+^ level (P < 0.01) and repressed the GSH concentration (P < 0.01) (**Figures 7A–C**); meanwhile, it also lowered the GPX4 (P < 0.05) and SLC7A11 (P < 0.05) expressions and heightened the ACSL4 expression (P < 0.01) (**Figures 7D–G**) in OA rat models. Significantly, the effect of OA-FLS exosomes on the above ferroptosis-related indexes was attenuated by Fer-1 treatment or miR-19b-3p inhibitor transfection (transfected into OA-FLS exosomes) (**Figures 7A–G**). These indicated that OA-FLS exosomes enhanced cartilage ferroptosis via delivering miR-19b-3p in OA rat models.

### 3.6 Speculative schematic diagram about OA-FLS exosomal miR-19b-3p and OA ferroptosis

**Figure 8:**
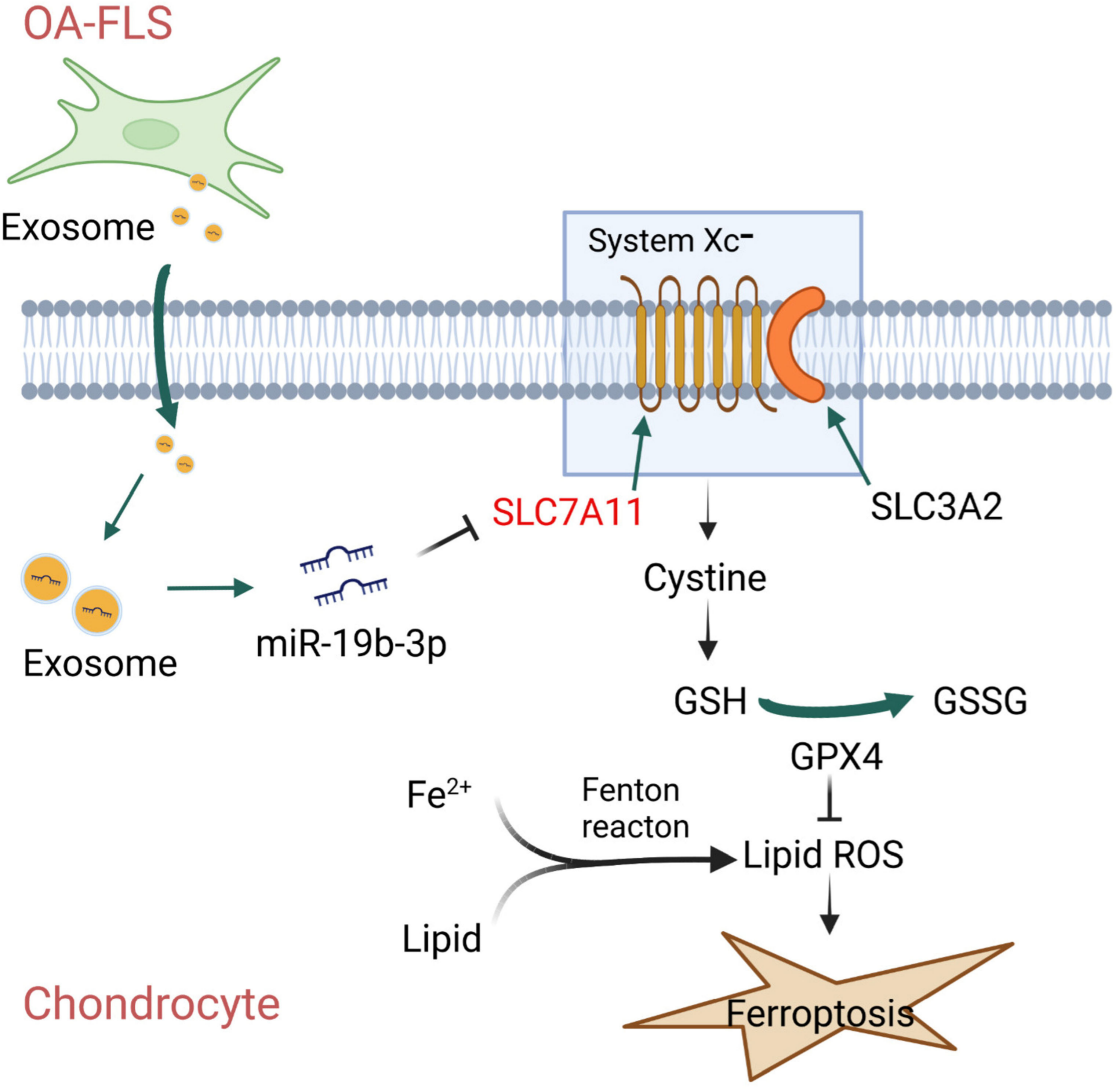
Speculative schematic diagram.

To briefly and pellucidly speculate the mechanism between OA-FLS exosomal miR-19b-3p and OA ferroptosis, a schematic diagram was drawn (**Figure 8**), which showed that OA-FLSs secreted exosomes to deliver miR-19b-3p into chondrocytes, then targeted SLC7A11 to regulate MMP and cystine, followed by GSH/GSSG-mediated GPX4, therefore promoting OA ferroptosis.

## 4 Discussion

Ferroptosis is recently observed to be closely involved in degenerative diseases and arthritis (30–36). For instance, ferroptosis and its related oxidative stress, redox metal, and neuron death can promote the pathological process of Alzheimer’s disease (AD) (30), which is observed to be enhanced in AD brains (31–33), also shows potency to be a treatment target for AD (34). Moreover, ferroptosis-related markers are dysregulated in rats with adjuvant arthritis (35); targeting ferroptosis also reveals a potential for rheumatoid arthritis treatment (36). In terms of OA, a previous study observed accumulative iron and suppressed levels of GPX4 and GSH in cartilage and synovial fluid during the OA pathologic process (21). Our present study observed enhanced ferroptosis in cartilage tissues from human OA patients, cartilage tissues from OA rat models, and IL-1β-stimulated chondrocytes (OA cellular model). These data (clinical samples, *in vivo*, and *in vitro*) comprehensively manifested an enhanced ferroptosis in OA, indicating the close involvement of ferroptosis in OA development. The possible explanation was that ferroptosis-related redox homeostasis, iron accumulation, mitochondrial activity, etc., contributed to OA pathogenesis (16, 20, 23).

Exosomes, as extracellular vesicles secreted by almost all types of cells, consist of abundant mRNA, miRNA, proteins, lipids, etc. (37). An interesting study reports that exosomes derived from IL-1β-stimulated FLS promote IL-6, MMP-3, MMP-13, and vascular endothelial growth factor (VEGF) but suppress collagen II in OA chondrocytes (38). Reversely, it is also observed that exosomes from osteoarthritic chondrocytes activate ROS and inflammation to deteriorate synovitis (39). The abovementioned studies reveal a potential positive loop between synovitis and cartilage damage during the OA process. Moreover, our previous study discovered that exosomes derived from normal synovial mesenchymal stem cells (but not OA synovial ones) improved OA chondrogenesis (24). However, the effect of OA-FLS exosomes on cartilage ferroptosis in OA is never reported. The current study found that OA-FLS exosomes facilitated ferroptosis in IL-1β-stimulated chondrocytes (OA cellular model) and promoted cartilage ferroptosis and cartilage damage in OA rat models. This might result from the following reasons: 1) OA-FLS exosomes were abundant in lipids and then transmitted into chondrocytes; meanwhile, the lipids were essential for ferroptosis (40); 2) OA-FLS exosomes promoted inflammation, extracellular matrix (ECM), and ROS to activate ferroptosis in OA chondrocytes and cartilage and caused cartilage damage (38, 39); 3) OA-FLS exosomes delivered RNA such as miR-19b-3p as discovered in our subsequent study to enhance ferroptosis in OA.

miR-19b-3p is a famous noncoding RNA involved in the pathogenesis of various diseases such as cancers, skeletal abnormalities, and degenerative diseases (41–43). In the aspect of OA, miR-19b-3p is reported to regulate ECM degradation and inflammation in OA (44), and its change during/post-treatment relates to OA recovery (45). Moreover, our previous study used microarray and RT-qPCR assay to confirm that miR-19b-3p was elevated in knee OA patients compared to healthy controls, which was further revealed to be an independent risk factor for OA susceptibility (25). In line with our previous study (25), the present study observed that miR-19b-3p was higher in cartilage tissues of OA patients and elevated in IL-1β-stimulated chondrocytes (OA cellular model) as well as in cartilage tissues of OA rat models. These data further validated the miR-19b-3p involvement in OA development, which might be ascribed to the effect of miR-19b-3p on regulating chondrocyte functions, ECM degradation, and inflammation (44–46). Importantly, our present study also discovered that OA-FLS exosomes promote the damage and ferroptosis of chondrocyte and cartilage via delivering miR-19b-3p *in vitro* and *in vivo*, respectively. These might result from the effect of miR-19b-3p on oxidative stress, inflammation, and direct ferroptosis and its abundant contents in OA-FLS exosomes (44–49).

To further confirm the potential mechanism, the targets of miR-19b-3p were predicted via an online database, then it was observed that a critical ferroptosis-related factor (SLC7A11) (50, 51) was able to bind miR-19b-3p directly. Therefore, a luciferase reporter gene assay was subsequently carried out, revealing a direct RNA sequence combination between the miR-19b-3p and SLC7A11. Furthermore, a rescue experiment using miR-19b-3p mimics and SLC7A11 overexpression plasmids alone or in combination observed that miR-19b-3p enhanced ferroptosis via targeting SLC7A11 in IL-1β-stimulated chondrocytes (OA cellular model). These could be explained by the direct involvement of SLC7A11 in ferroptosis regulation (50, 51). This study could provide more evidence for the clinical development of exosome-related agents or drugs to help manage OA. Moreover, it also points out the potential mechanism of the ferroptosis of the chondrocyte, which could supply more potential targets for the clinical development of targeted drugs to treat OA.

Conclusively, OA-FLS exosomal miR-19b-3p promotes cartilage ferroptosis and damage via targeting SLC7A11 in OA, shedding some light on the interaction between synovium and cartilage as well as their relationship to ferroptosis during the OA process.

## Data availability statement

The original contributions presented in the study are included in the article/**Supplementary Material**. Further inquiries can be directed to the corresponding author.

## Ethics statement

The animal experiments were approved by the Animal Care and Use Committee and by the guidelines of Ethics Committee of Changhai Hospital, Naval Medical University.

## Author contributions

JG, DZ, and YP contributed to the study conception and design. RK performed data collection and analysis. LJ was responsible for the interpretation of data for the work. All authors contributed to the drafting of the article and revising it critically for important intellectual content. All authors read and approved the final article.
