# Supplementary material for: Exosomes from osteoarthritic fibroblast-like synoviocytes promote cartilage ferroptosis and damage via delivering microRNA-19b-3p to target SLC7A11 in osteoarthritis
Source: Front Immunol. 2023 Aug 24;14:1181156. doi: 10.3389/fimmu.2023.1181156 (PMC10484587; doi:10.3389/fimmu.2023.1181156)
Supplement: Supplementary file 1 [file Presentation_1.pdf]

Supplementary Materials

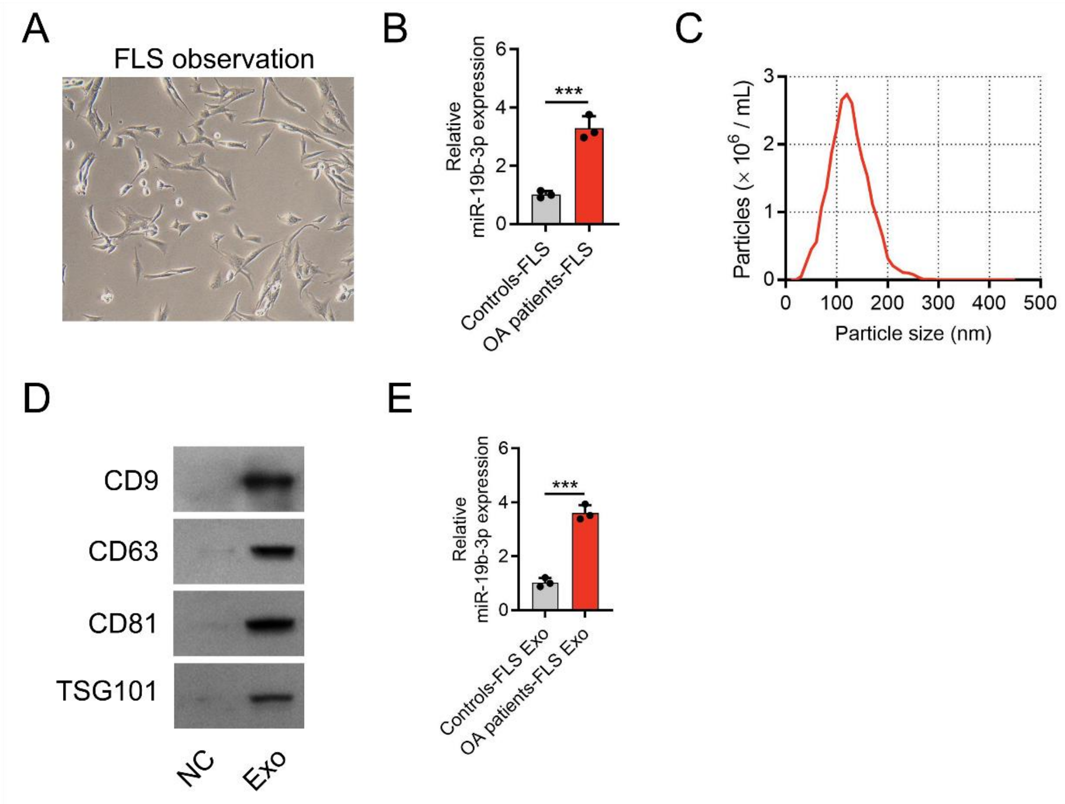

Supplementary Figure 1. FLS isolation, FLS exosome extraction, and miR-19b-3p expression. Morphology of FLS (A), the miR-19b-3p expression between Control-FLS and OA-FLS, the size distribution of FLS exosomes (C), expression of markers of exosomes (D), the miR-19b-3p expression between Control-FLS exosomes and OA-FLS exosomes (E). Triple repetition were carried out for each experiment. The error bars stood for the standard deviation. The one-way ANOVA followed by Tukey's multiple comparisons tests was carried out for the comparison test.

FLS, fibroblast-like synoviocytes; MiR, microRNA; OA, osteoarthritis; ANOVA, one-way analysis of variance.

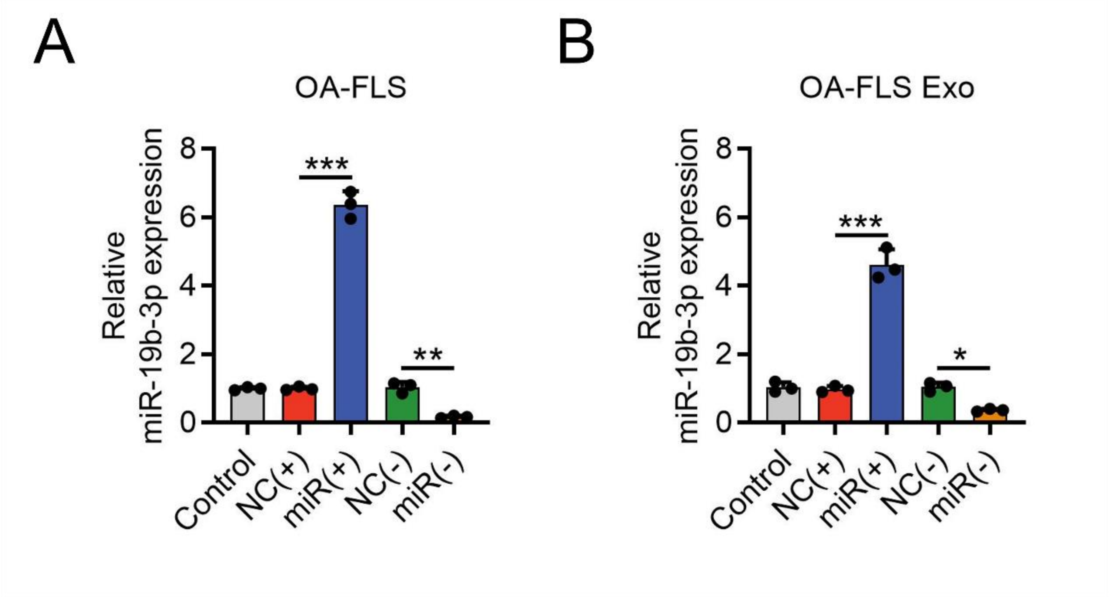

Supplementary Figure 2. Expression of miR-19b-3p after transfection. MiR-19b-3p expression in OA-FLS among Control, NC(+), miR(+), NC(-), and miR(-) groups (A). MiR-19b-3p expression in OA-FLS exosomes among Control, NC(+), miR(+), NC(-), and miR(-) groups (B). Triple repetition were carried out for each experiment. The error bars stood for the standard deviation. The one-way ANOVA followed by Tukey's multiple comparisons tests was carried out for the comparison test.

MiR, microRNA; OA, osteoarthritis; FLS, fibroblast-like synoviocytes; NC, negative control; ANOVA, one-way analysis of variance.

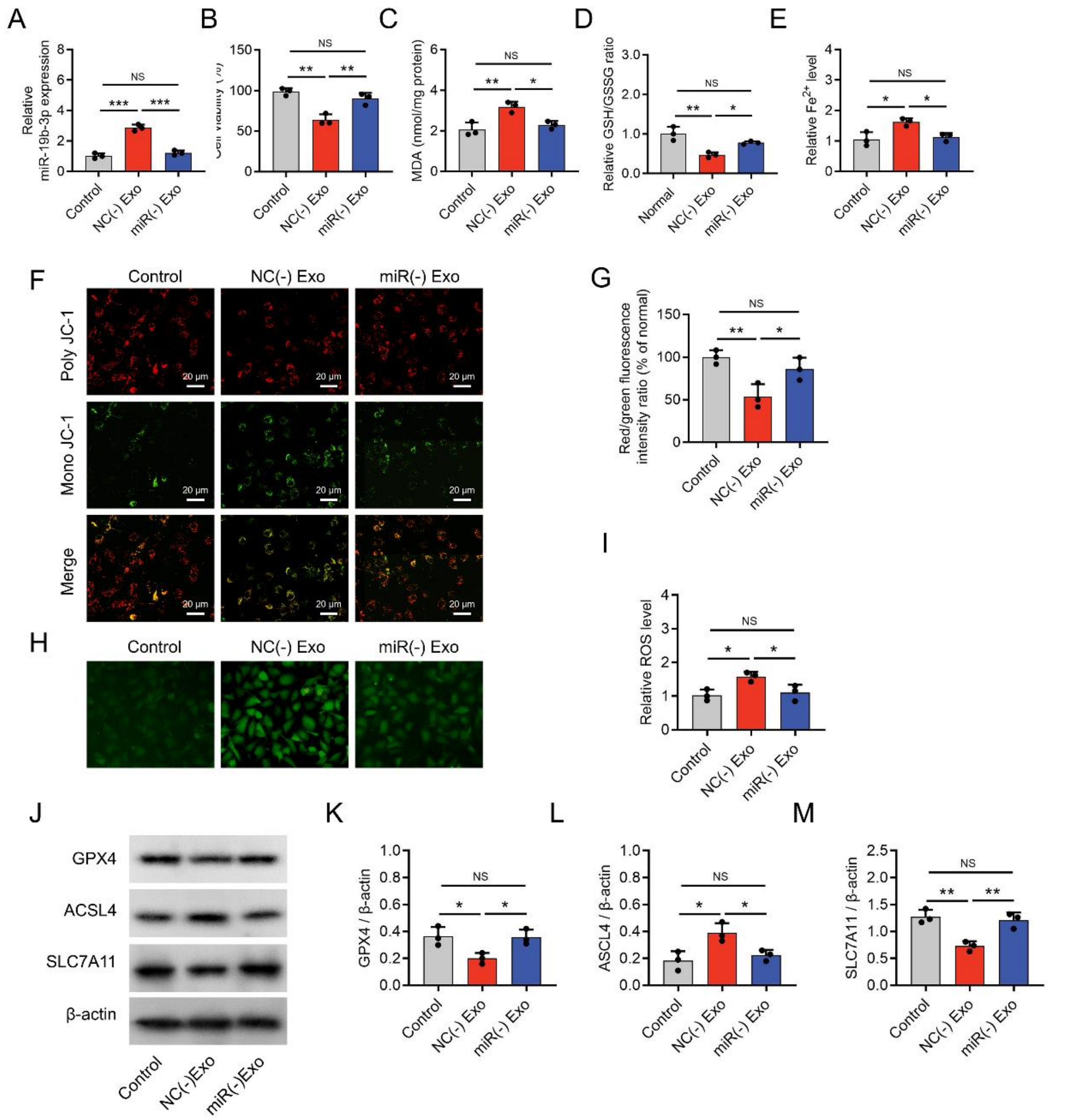

Supplementary Figure 3. miR(-) exosome treatment could not alter the miR-19b-3p level in the non-treated chondrocytes. MiR-19b-3p expression (A), cell viability (B), levels of MDA (C), GSH/GSSG (D), Fe^{2+} (E), poly JC-1 and mono JC-1 staining images and quantification (F-G), ROS staining and quantification (H-I), western blots images (J) and quantified expressions of GPX4 (K), ACSL4 (L), SLC7A11 (M), among Control, NC(-) Exo, and miR(-) Exo groups. Triple repetition were carried out for each experiment. The error bars stood for the standard deviation. The one-way ANOVA followed by Tukey's multiple comparisons tests was carried out for the comparison test. MiR, microRNA; OA, osteoarthritis; FLS, fibroblast-like synoviocytes; MDA, malondialdehyde; GSH, glutathione; Fe2+, ferrous ion; JC-1, 5,5',6,6'-tetrachloro-1,1',3,3'-tetraethylbenzimidazolylcarbocyanine iodide; ROS, reactive oxygen species; GPX4, glutathione peroxidase 4; ACSL4, acyl-CoA synthetase long-chain family member 4; SLC7A11, solute carrier family 7 member 11; NC, negative control; Exo, exosome; Fer-1, ferrostatin-1; ANOVA, one-way analysis of variance.

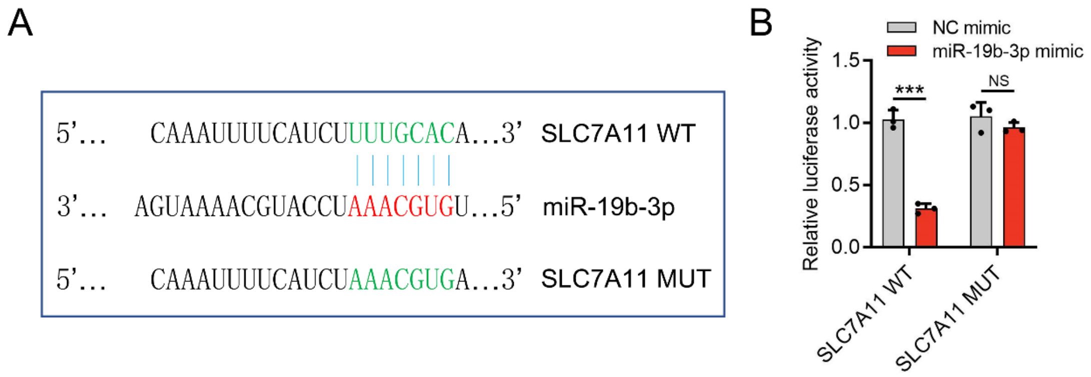

Supplementary Figure 4. Luciferase reporter gene assay. The predicted binding site between miR-19b-3p and SLC7A11, and designed mutant type of SLC7A11 sequence (A), comparison of relative luciferase activity (B). Triple repetition were carried out for each experiment. The error bars stood for the standard deviation. The one-way ANOVA followed by Tukey's multiple comparisons tests was carried out for the comparison test. MiR, microRNA; OA, osteoarthritis; FLS, fibroblast-like synoviocytes; SLC7A11, solute carrier family 7 member 11; NC, negative control; Exo, exosome; Fer-1, ferrostatin-1; ANOVA, one-way analysis of variance.

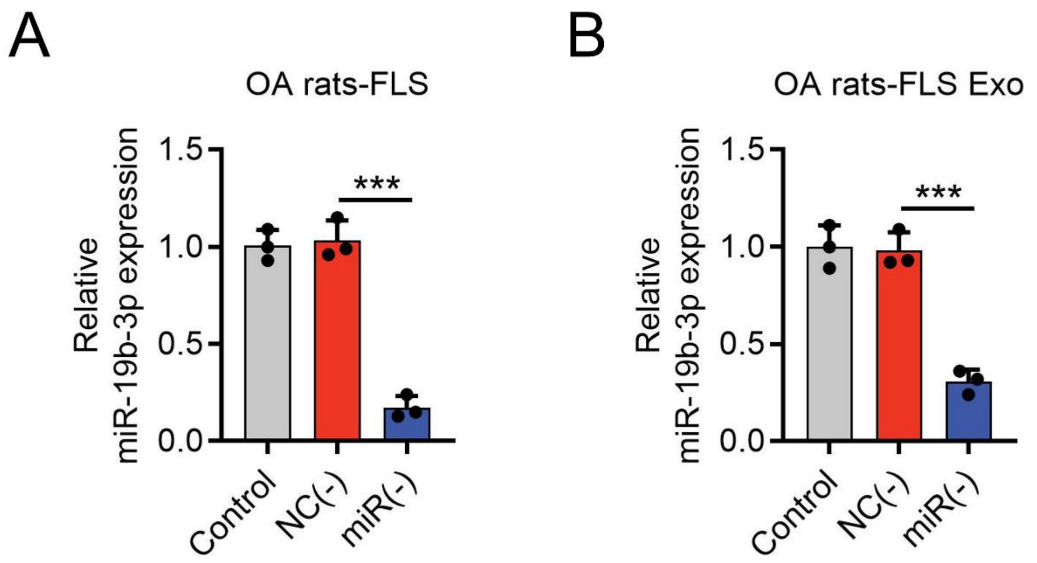

Supplementary Figure 5. MiR-19b-3p expression in OA rats FLS after transfection. MiR-19b-3p expression in OA rats FLS among Control, NC(-), and miR(-) groups (A). MiR-19b-3p expression in OA rats FLS exosomes among Control, NC(-), and miR(-) groups (B). Triple repetition were carried out for each experiment. The error bars stood for the standard deviation. The one-way ANOVA followed by Tukey's multiple comparisons tests was carried out for the comparison test.

MiR, microRNA; OA, osteoarthritis; FLS, fibroblast-like synoviocytes; NC, negative control; Exo, exosome; Fer-1, ferrostatin-1; ANOVA, one-way analysis of variance.
