# Supplementary figures and images for: Exosomes from osteoarthritic fibroblast-like synoviocytes promote cartilage ferroptosis and damage via delivering microRNA-19b-3p to target SLC7A11 in osteoarthritis (part 1 of 6)
Source: Front Immunol. 2023 Aug 24;14:1181156. doi: 10.3389/fimmu.2023.1181156 (PMC10484587; doi:10.3389/fimmu.2023.1181156)

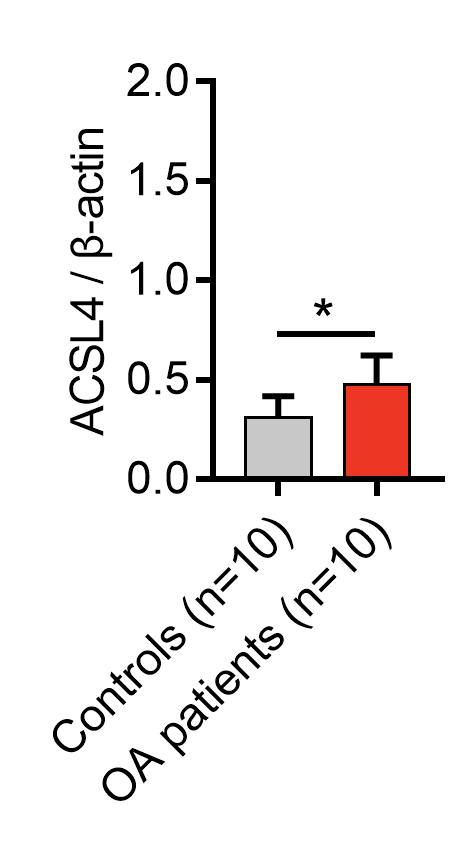

Supplement: Supplementary file 2 [file DataSheet_1.zip › Exported images from GraphPad/Fig1-ACSL4.jpg]

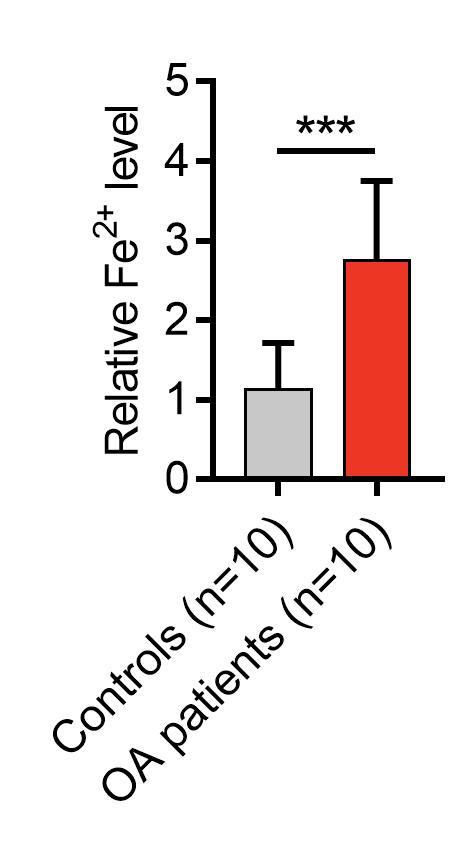

Supplement: Supplementary file 2 [file DataSheet_1.zip › Exported images from GraphPad/Fig1-Fe.jpg]

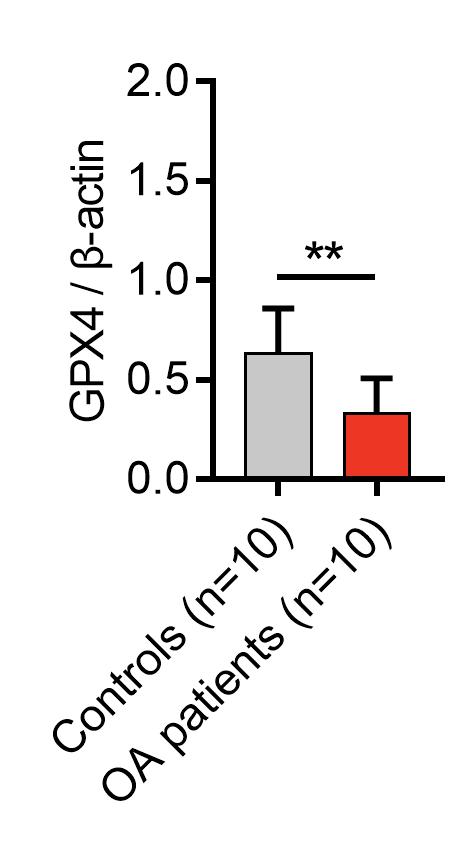

Supplement: Supplementary file 2 [file DataSheet_1.zip › Exported images from GraphPad/Fig1-GPX4.jpg]

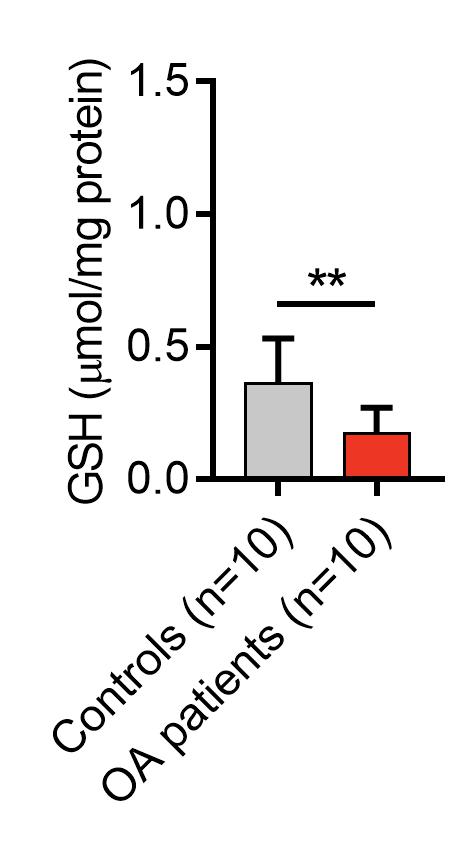

Supplement: Supplementary file 2 [file DataSheet_1.zip › Exported images from GraphPad/Fig1-GSH.jpg]

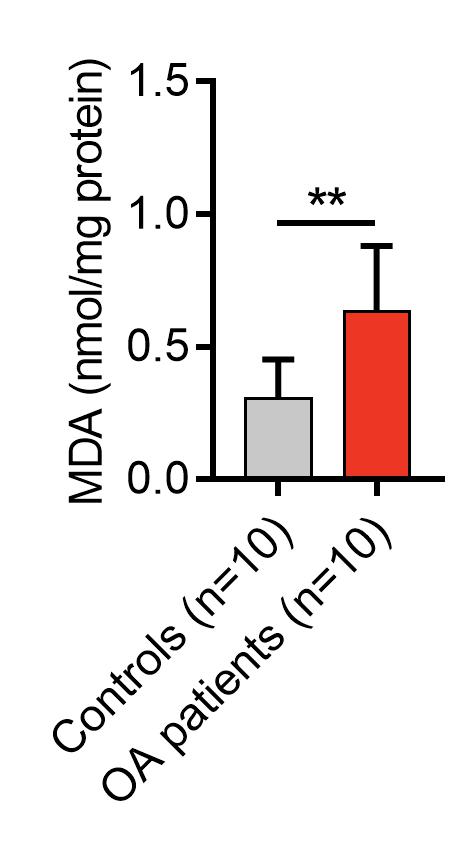

Supplement: Supplementary file 2 [file DataSheet_1.zip › Exported images from GraphPad/Fig1-MDA.jpg]

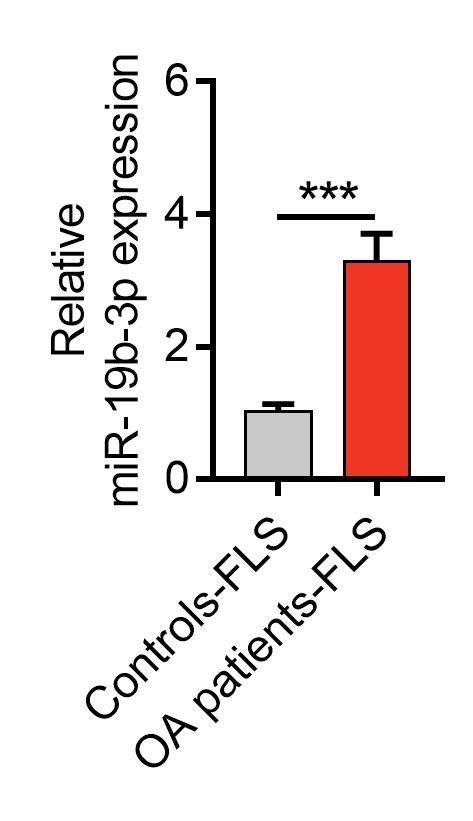

Supplement: Supplementary file 2 [file DataSheet_1.zip › Exported images from GraphPad/Fig1-S1-PCR.jpg]

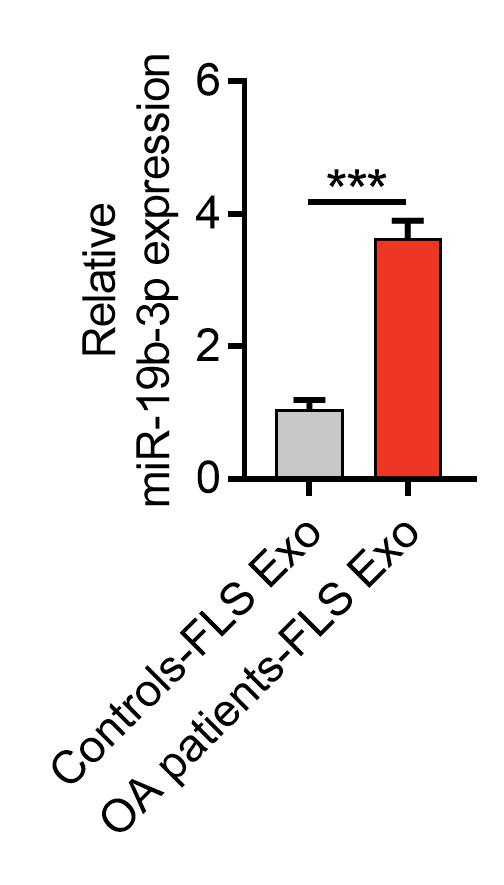

Supplement: Supplementary file 2 [file DataSheet_1.zip › Exported images from GraphPad/Fig1-S1-PCR2.jpg]

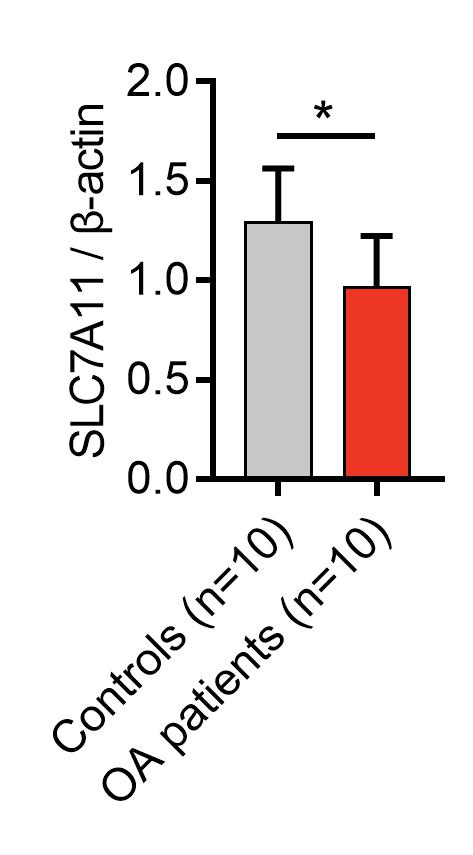

Supplement: Supplementary file 2 [file DataSheet_1.zip › Exported images from GraphPad/Fig1-SLC7A11.jpg]

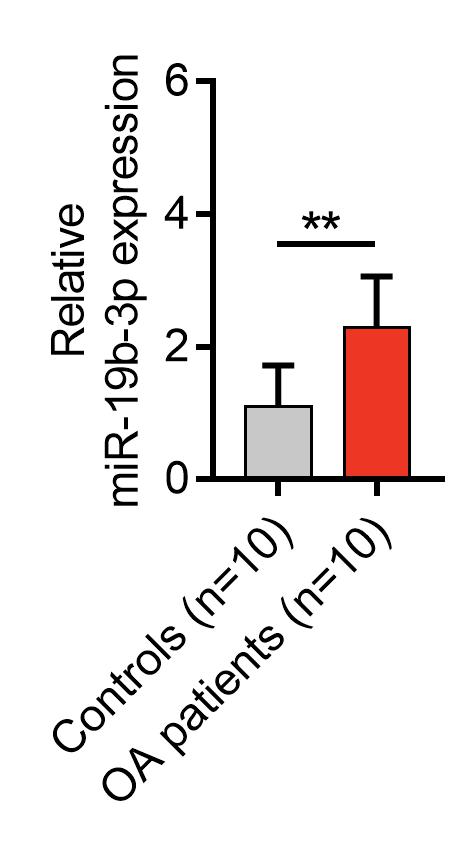

Supplement: Supplementary file 2 [file DataSheet_1.zip › Exported images from GraphPad/Fig1-miR-19b-3p.jpg]

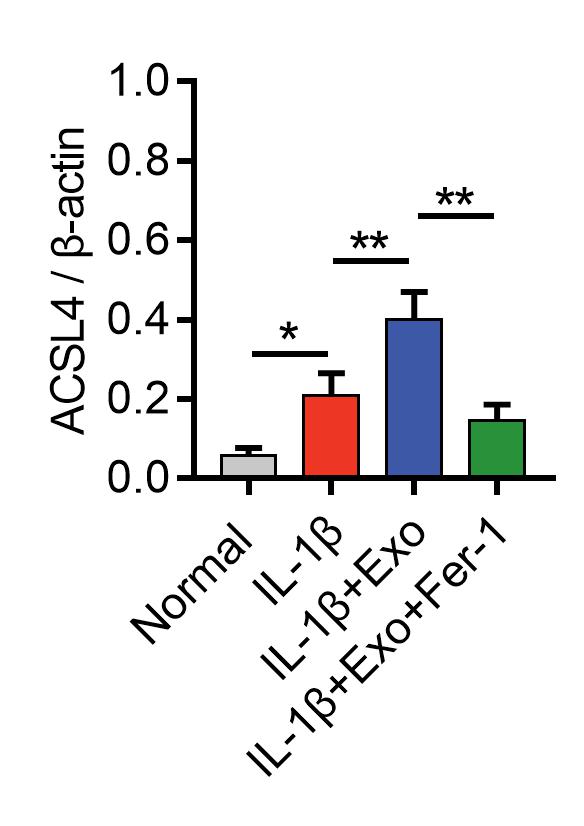

Supplement: Supplementary file 2 [file DataSheet_1.zip › Exported images from GraphPad/Fig2-ACSL4.jpg]

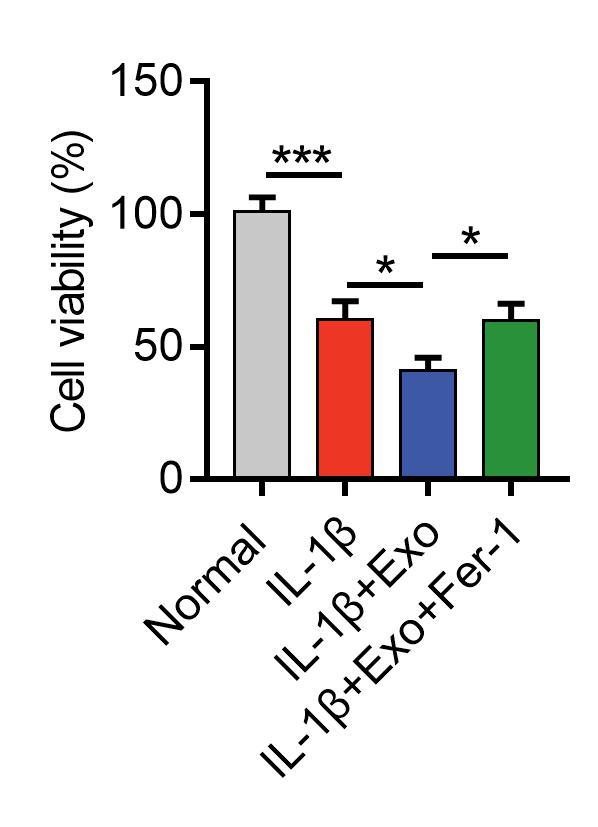

Supplement: Supplementary file 2 [file DataSheet_1.zip › Exported images from GraphPad/Fig2-CCK.jpg]

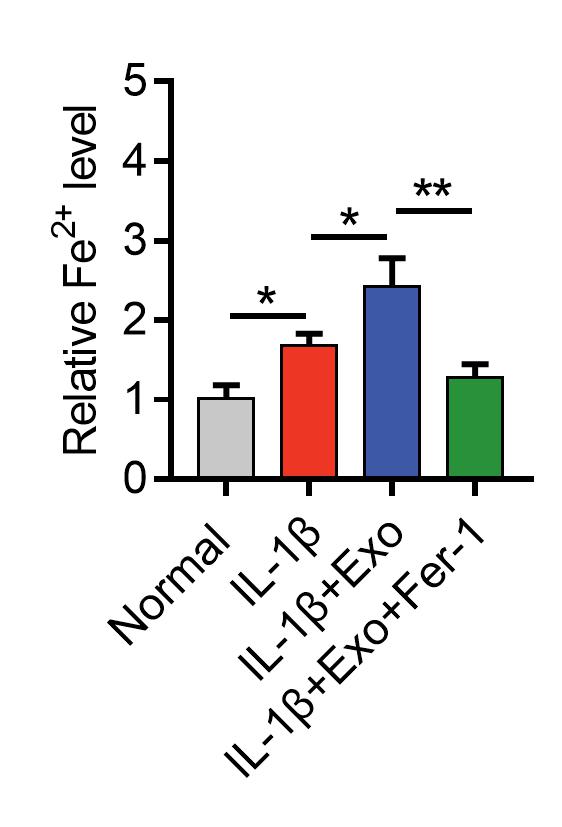

Supplement: Supplementary file 2 [file DataSheet_1.zip › Exported images from GraphPad/Fig2-Fe.jpg]

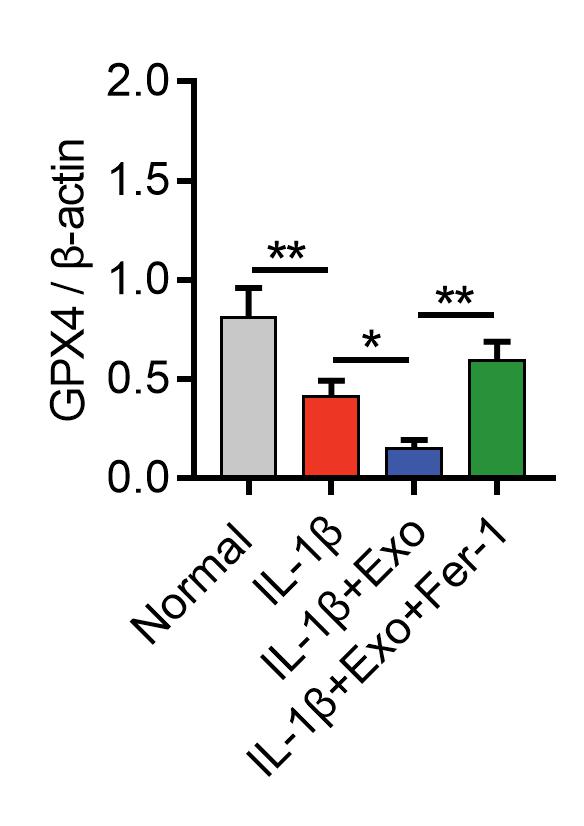

Supplement: Supplementary file 2 [file DataSheet_1.zip › Exported images from GraphPad/Fig2-GPX4.jpg]

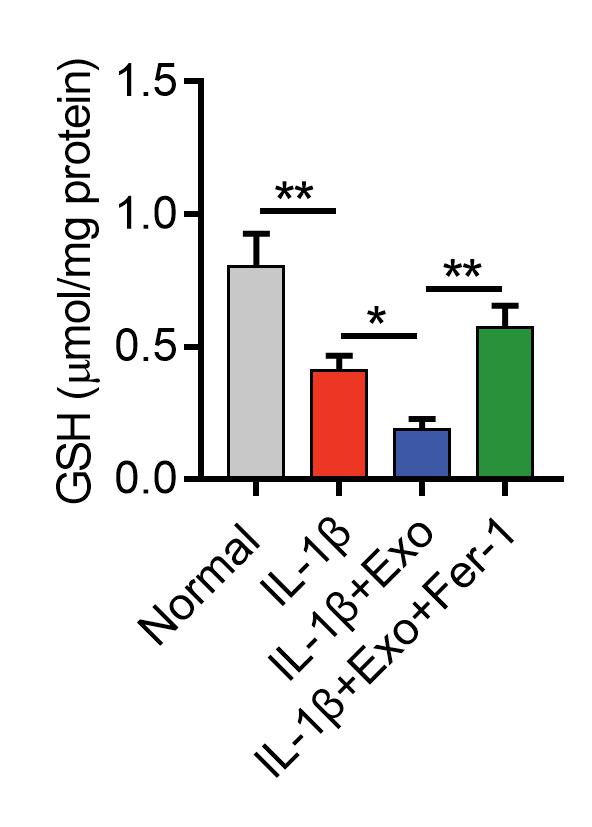

Supplement: Supplementary file 2 [file DataSheet_1.zip › Exported images from GraphPad/Fig2-GSH.jpg]

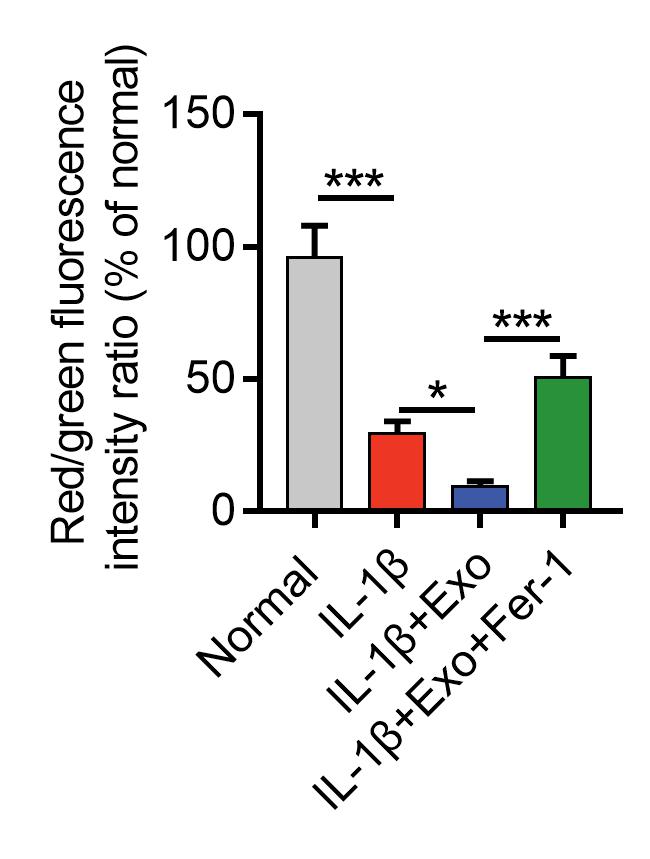

Supplement: Supplementary file 2 [file DataSheet_1.zip › Exported images from GraphPad/Fig2-JC-1.jpg]

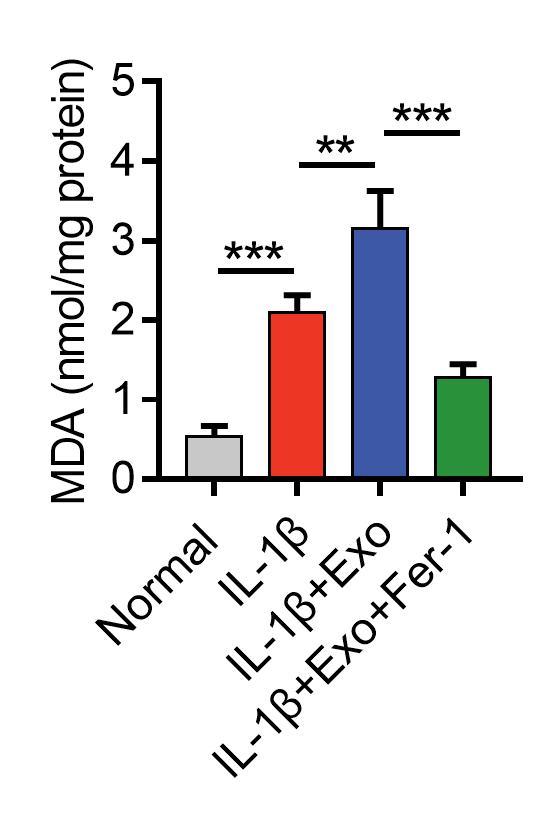

Supplement: Supplementary file 2 [file DataSheet_1.zip › Exported images from GraphPad/Fig2-MDA.jpg]

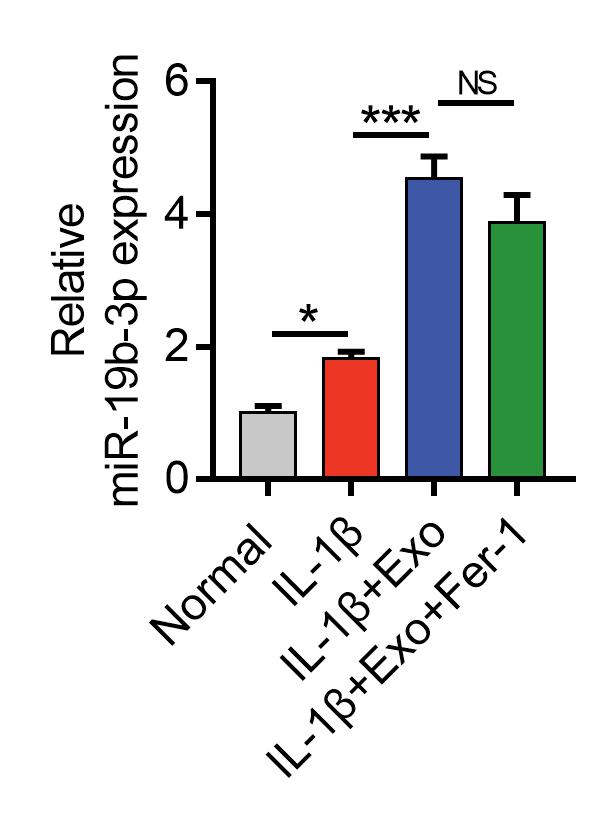

Supplement: Supplementary file 2 [file DataSheet_1.zip › Exported images from GraphPad/Fig2-PCR.jpg]

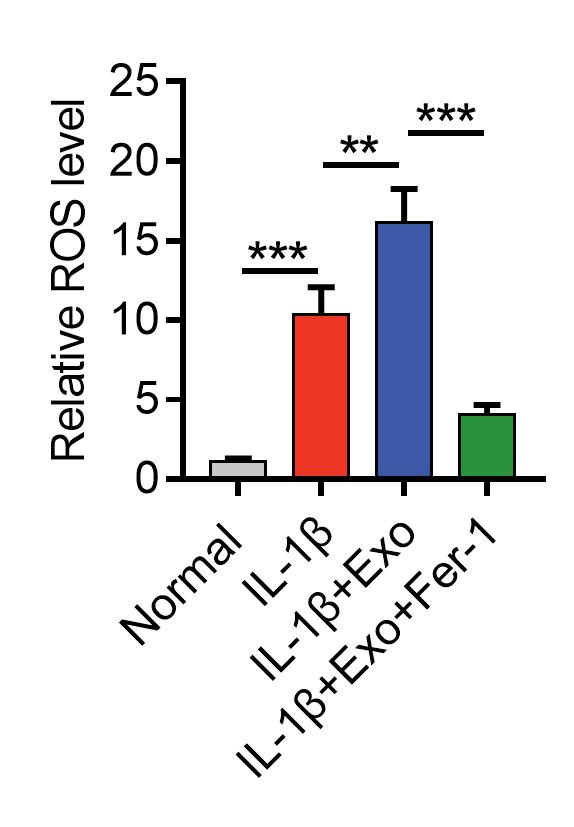

Supplement: Supplementary file 2 [file DataSheet_1.zip › Exported images from GraphPad/Fig2-ROS.jpg]

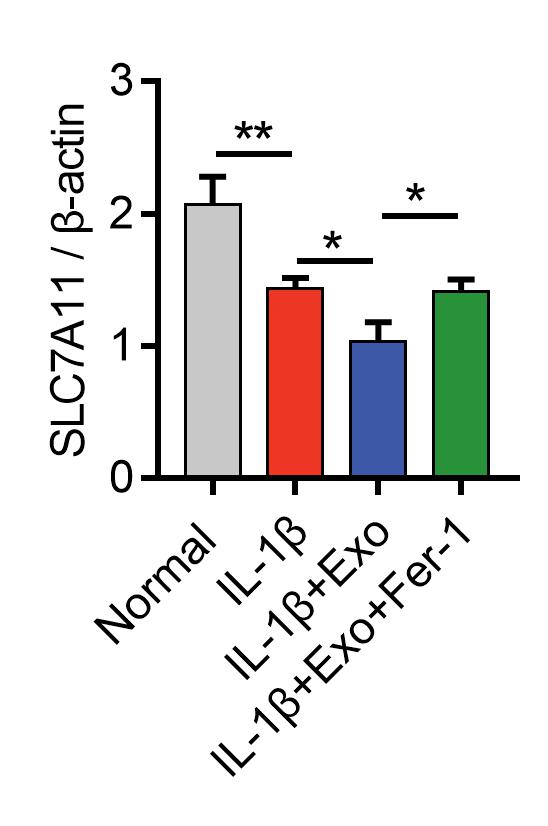

Supplement: Supplementary file 2 [file DataSheet_1.zip › Exported images from GraphPad/Fig2-SLC7A11.jpg]

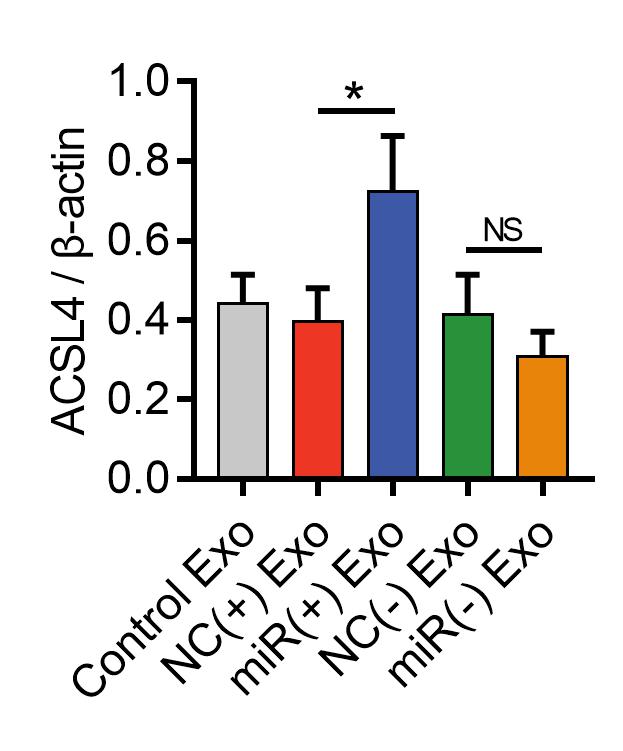

Supplement: Supplementary file 2 [file DataSheet_1.zip › Exported images from GraphPad/Fig3-ACSL4.jpg]

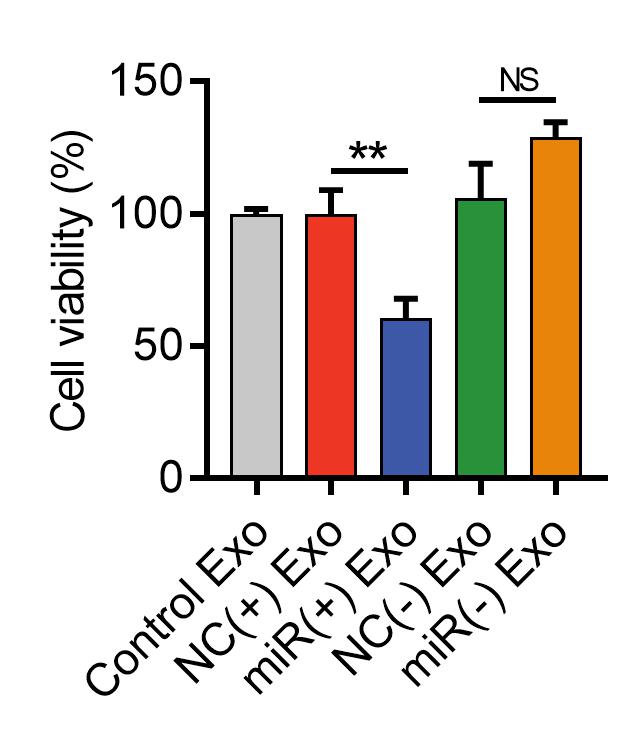

Supplement: Supplementary file 2 [file DataSheet_1.zip › Exported images from GraphPad/Fig3-CCK.jpg]

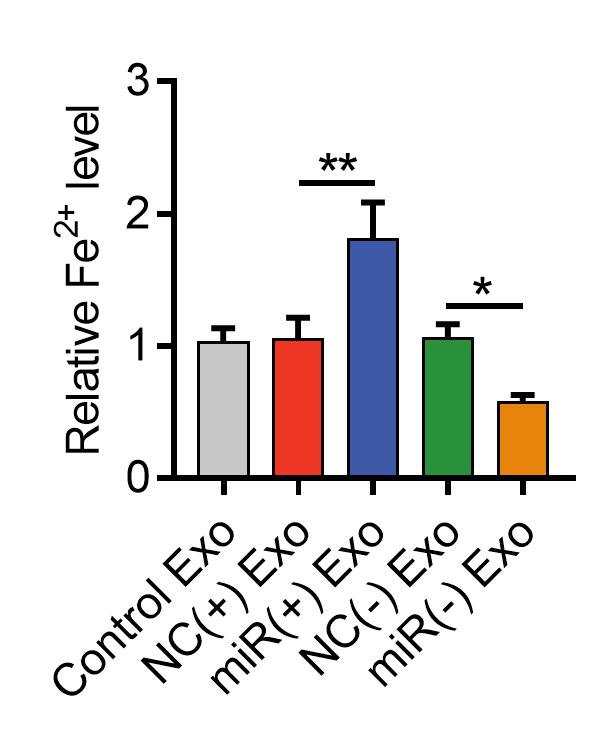

Supplement: Supplementary file 2 [file DataSheet_1.zip › Exported images from GraphPad/Fig3-Fe.jpg]

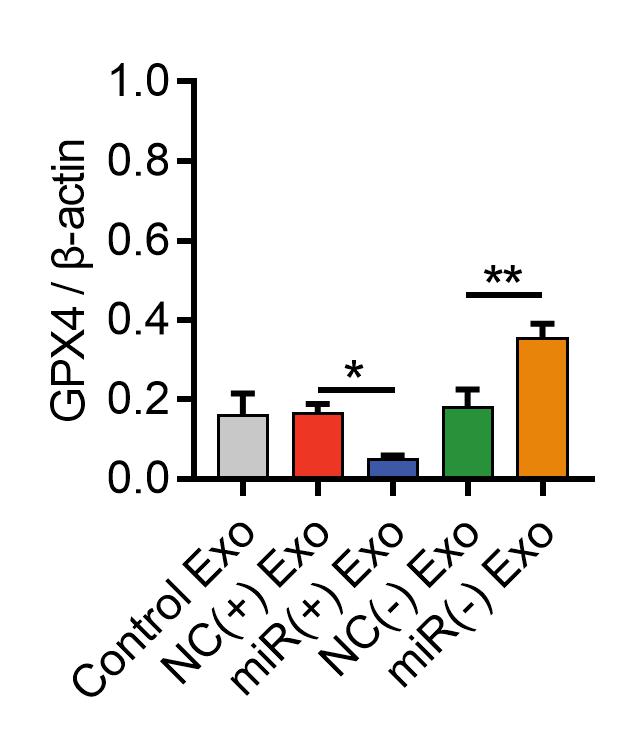

Supplement: Supplementary file 2 [file DataSheet_1.zip › Exported images from GraphPad/Fig3-GPX4.jpg]

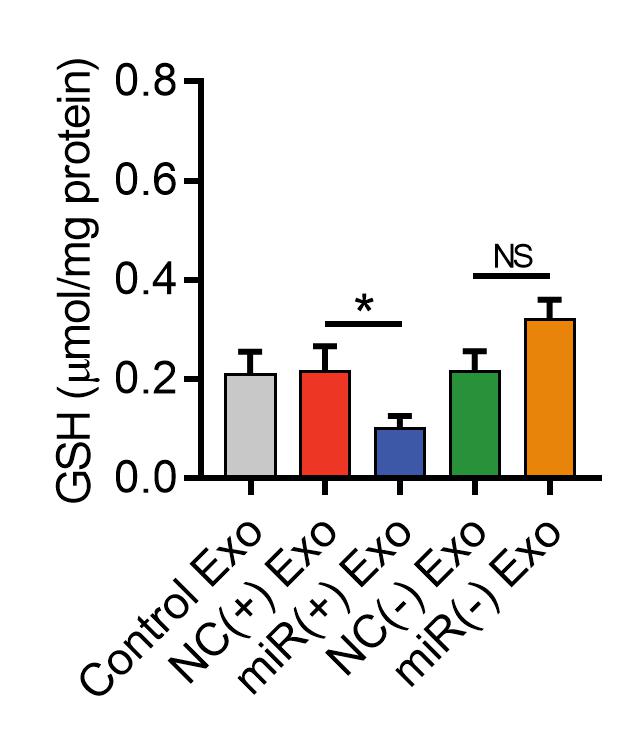

Supplement: Supplementary file 2 [file DataSheet_1.zip › Exported images from GraphPad/Fig3-GSH.jpg]

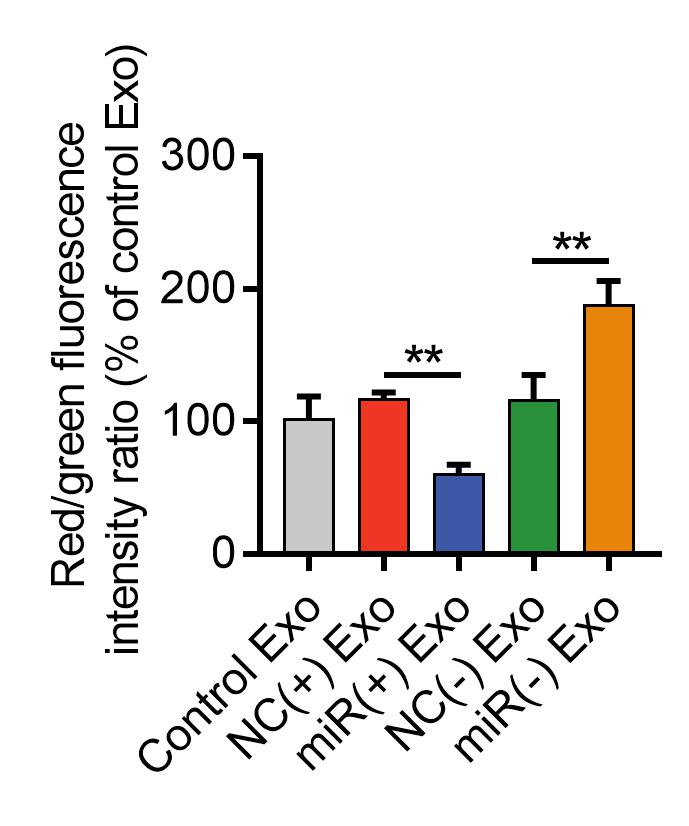

Supplement: Supplementary file 2 [file DataSheet_1.zip › Exported images from GraphPad/Fig3-JC-1.jpg]

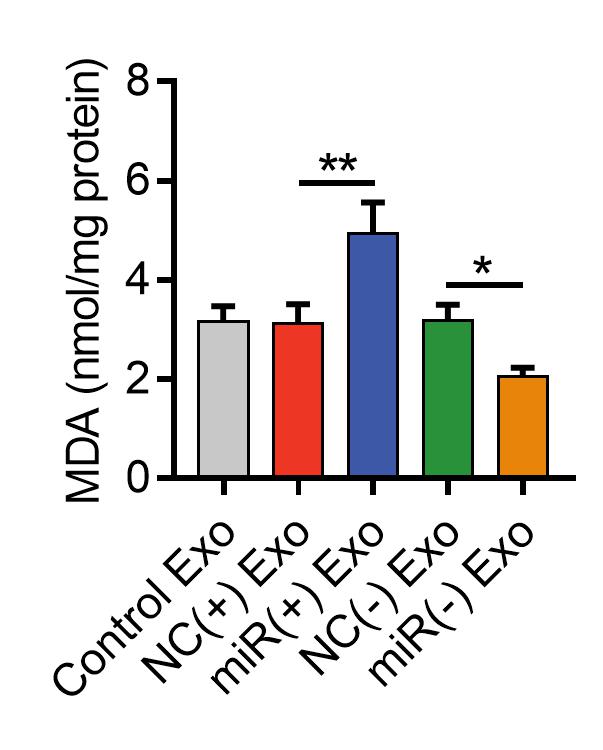

Supplement: Supplementary file 2 [file DataSheet_1.zip › Exported images from GraphPad/Fig3-MDA.jpg]

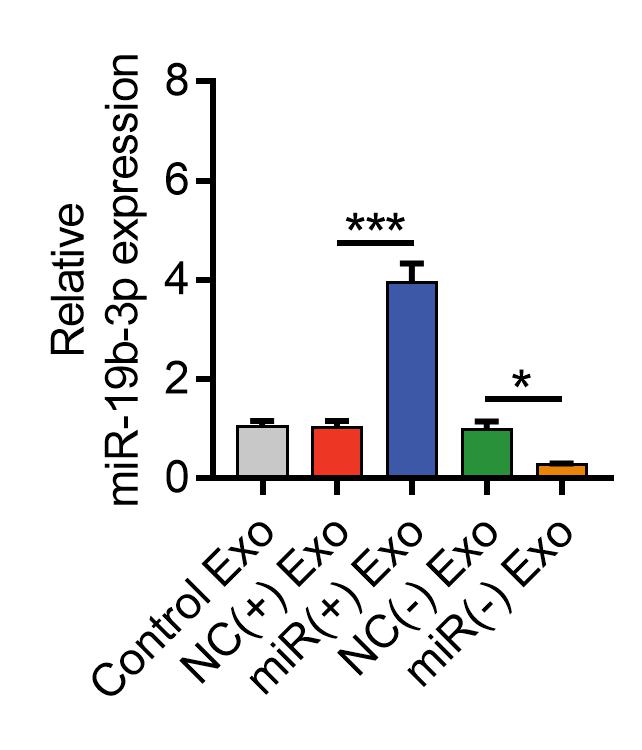

Supplement: Supplementary file 2 [file DataSheet_1.zip › Exported images from GraphPad/Fig3-PCR.jpg]

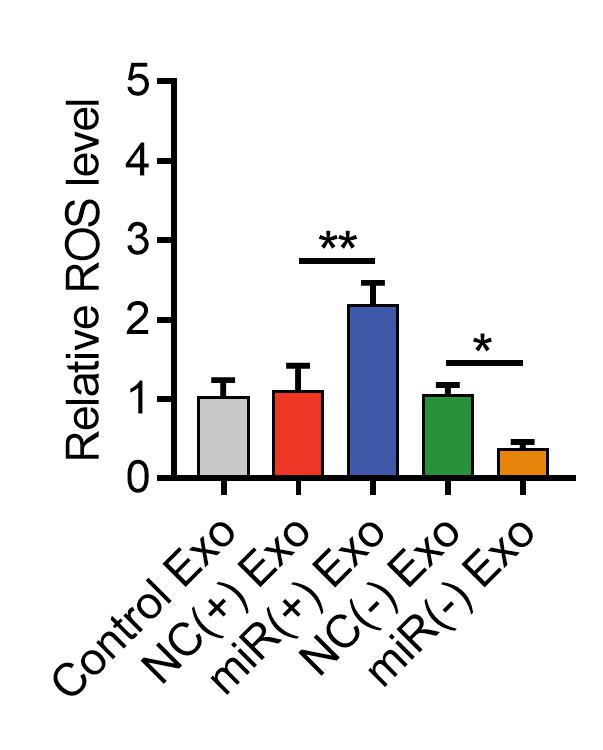

Supplement: Supplementary file 2 [file DataSheet_1.zip › Exported images from GraphPad/Fig3-ROS.jpg]

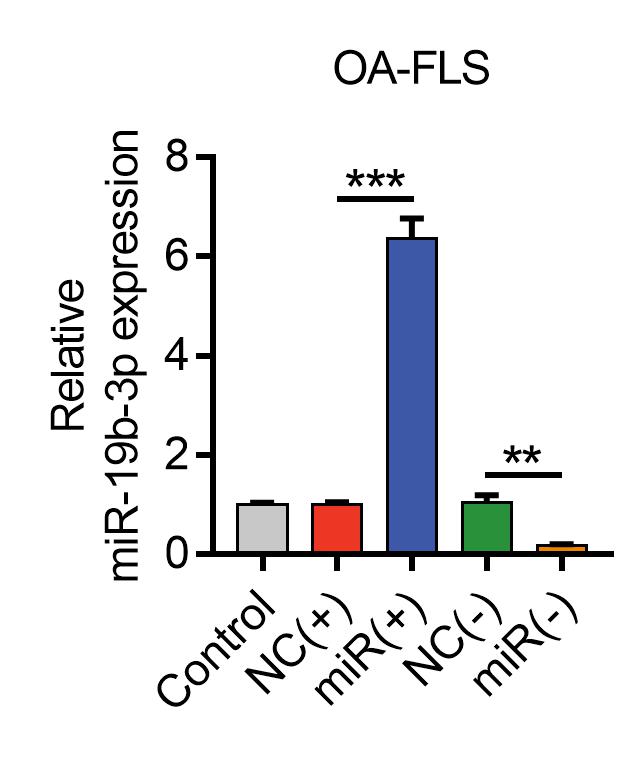

Supplement: Supplementary file 2 [file DataSheet_1.zip › Exported images from GraphPad/Fig3-S2-PCR.jpg]

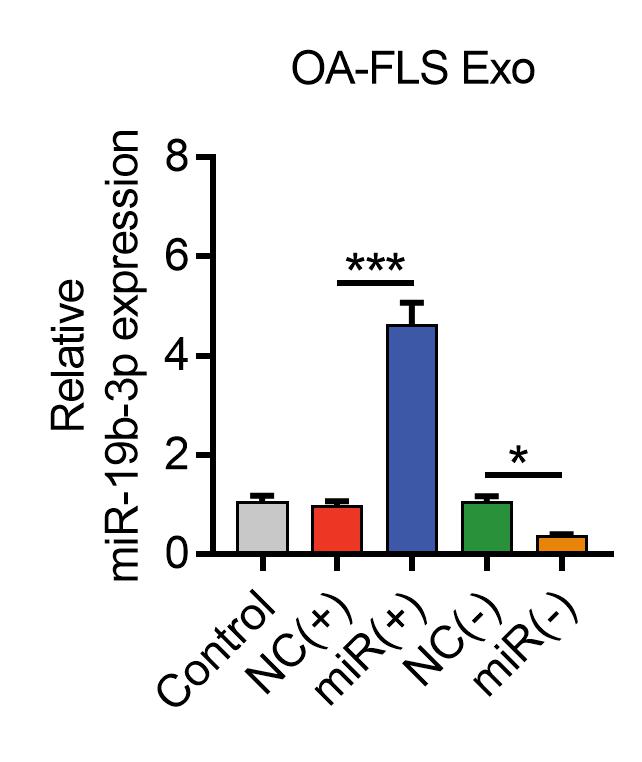

Supplement: Supplementary file 2 [file DataSheet_1.zip › Exported images from GraphPad/Fig3-S2-PCR2.jpg]

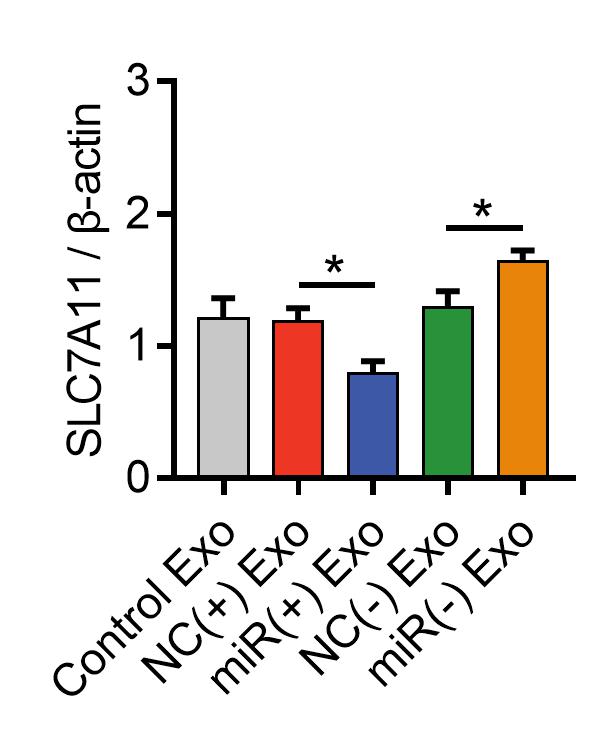

Supplement: Supplementary file 2 [file DataSheet_1.zip › Exported images from GraphPad/Fig3-SLC7A11.jpg]

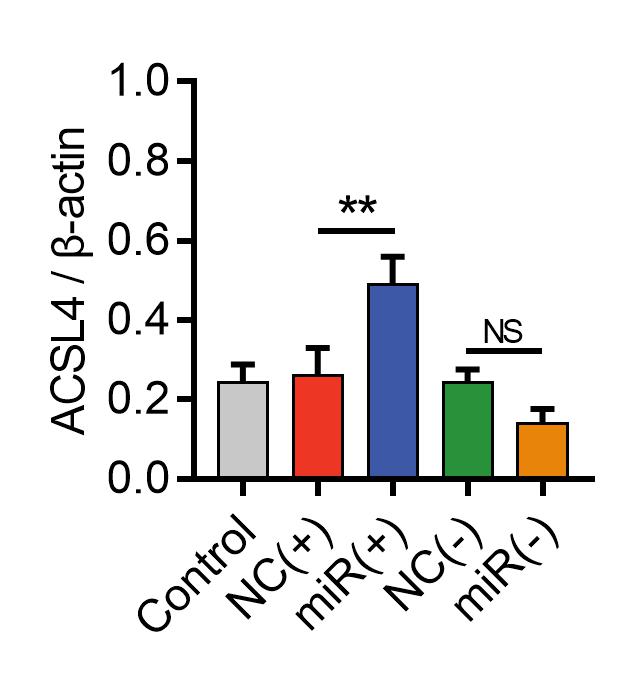

Supplement: Supplementary file 2 [file DataSheet_1.zip › Exported images from GraphPad/Fig4-ACSL4.jpg]

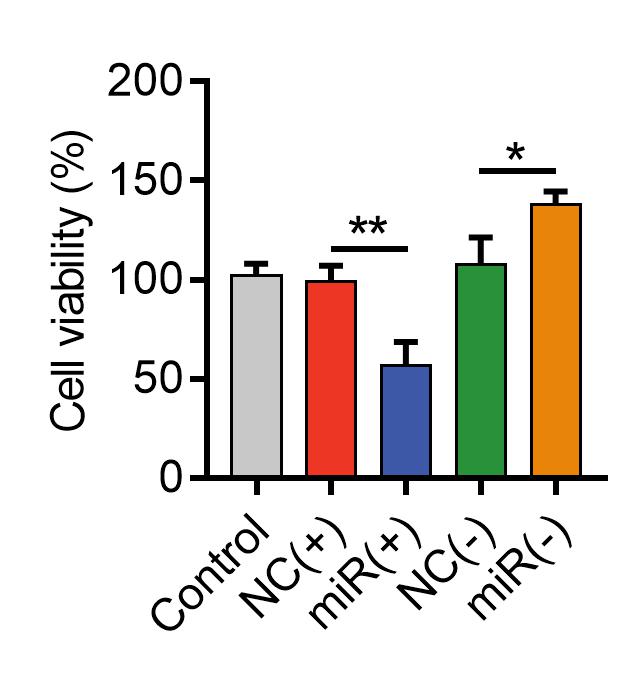

Supplement: Supplementary file 2 [file DataSheet_1.zip › Exported images from GraphPad/Fig4-CCK.jpg]

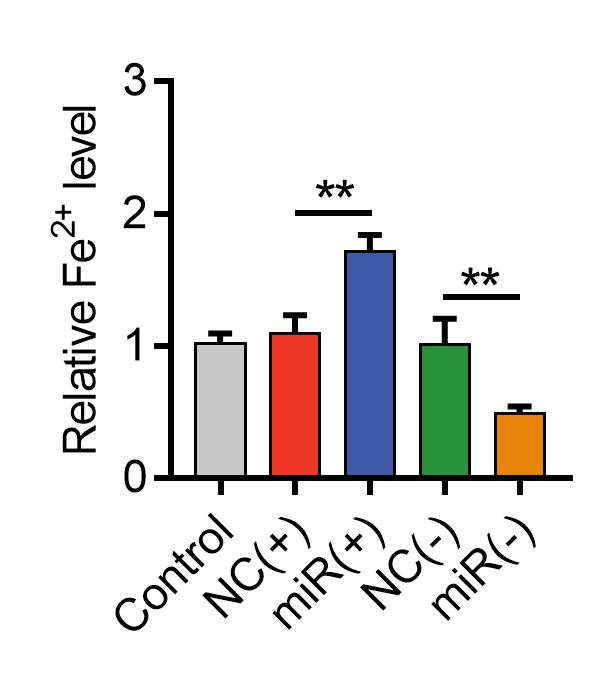

Supplement: Supplementary file 2 [file DataSheet_1.zip › Exported images from GraphPad/Fig4-Fe.jpg]

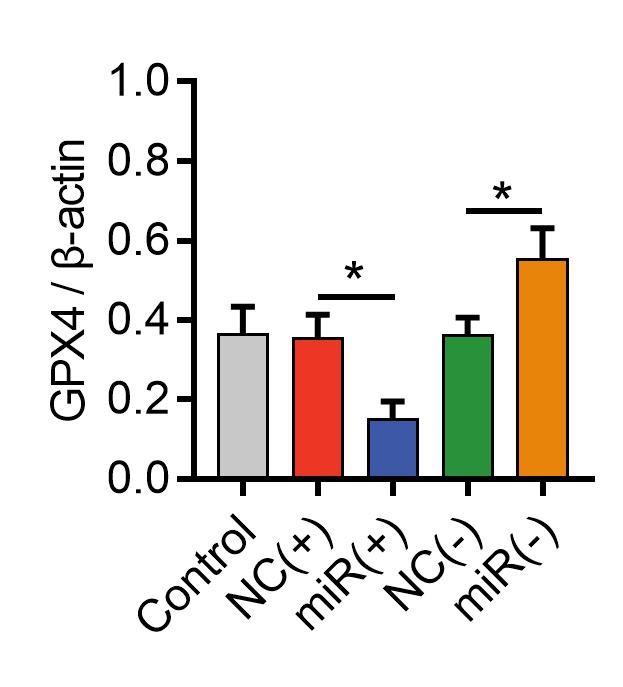

Supplement: Supplementary file 2 [file DataSheet_1.zip › Exported images from GraphPad/Fig4-GPX4.jpg]

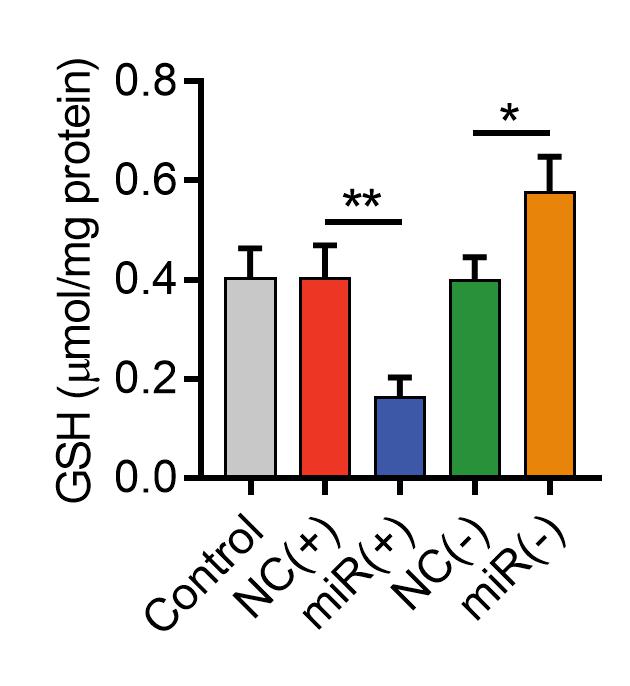

Supplement: Supplementary file 2 [file DataSheet_1.zip › Exported images from GraphPad/Fig4-GSH.jpg]

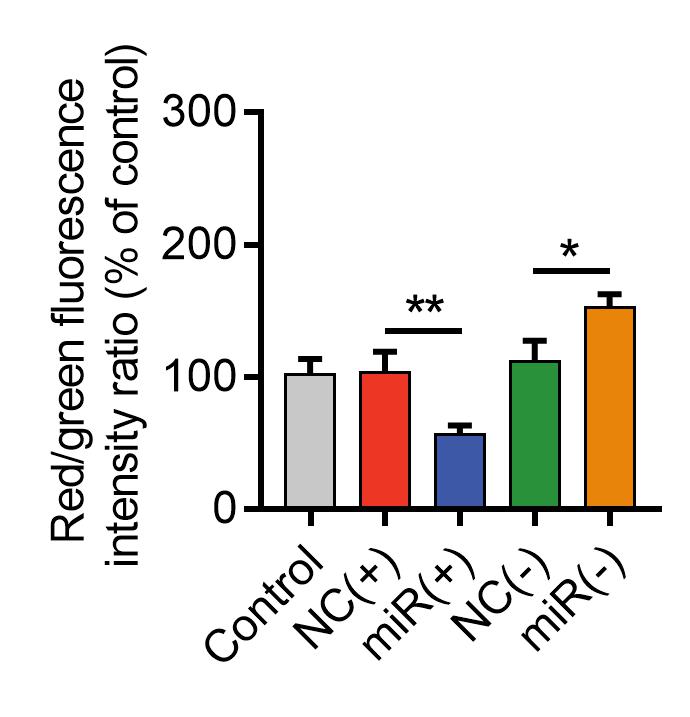

Supplement: Supplementary file 2 [file DataSheet_1.zip › Exported images from GraphPad/Fig4-JC-1.jpg]

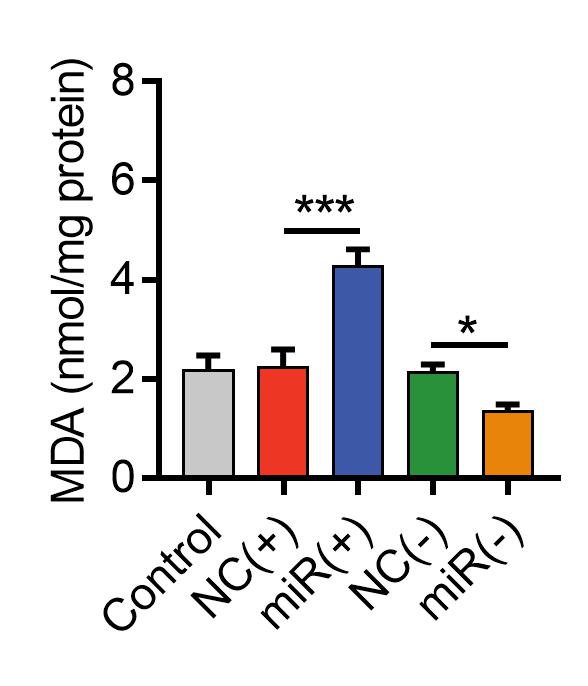

Supplement: Supplementary file 2 [file DataSheet_1.zip › Exported images from GraphPad/Fig4-MDA.jpg]

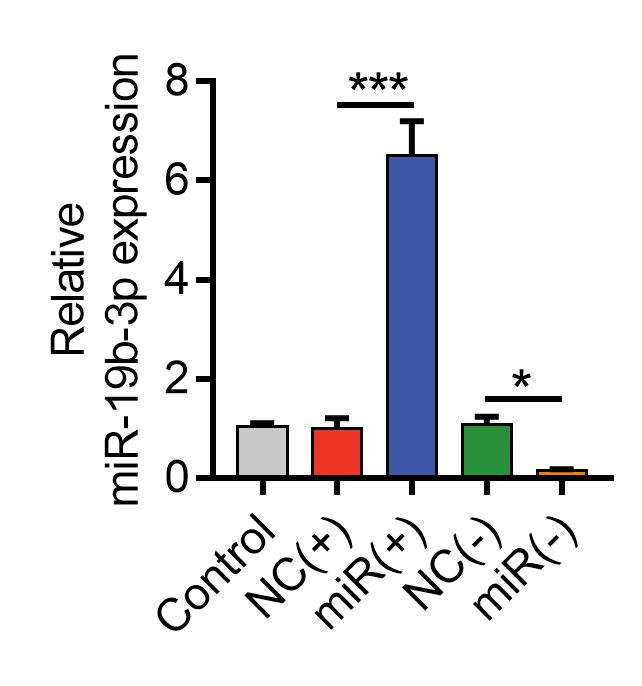

Supplement: Supplementary file 2 [file DataSheet_1.zip › Exported images from GraphPad/Fig4-PCR.jpg]

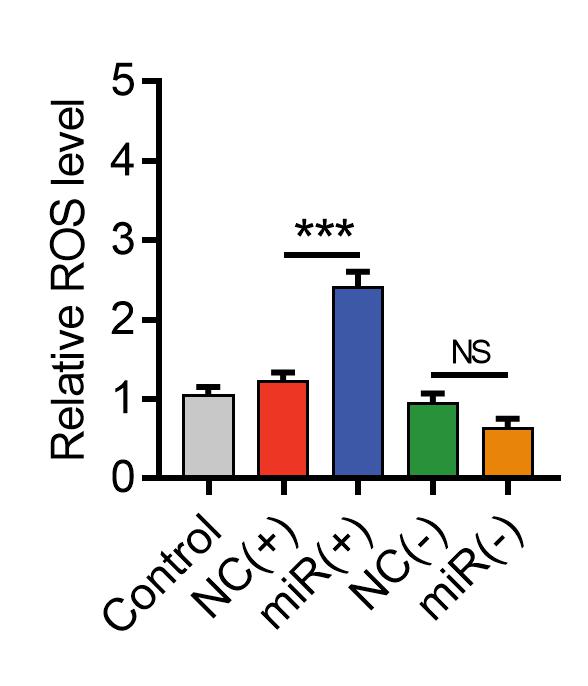

Supplement: Supplementary file 2 [file DataSheet_1.zip › Exported images from GraphPad/Fig4-ROS.jpg]

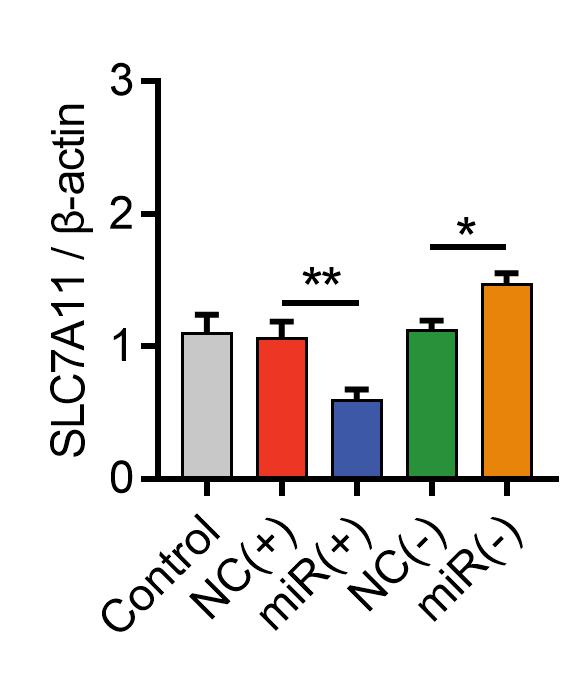

Supplement: Supplementary file 2 [file DataSheet_1.zip › Exported images from GraphPad/Fig4-SLC7A11.jpg]

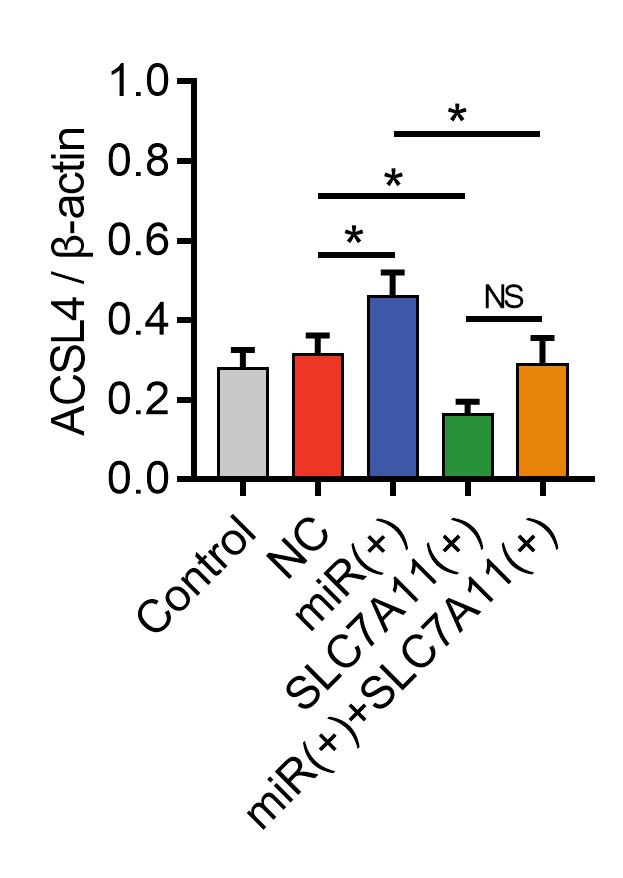

Supplement: Supplementary file 2 [file DataSheet_1.zip › Exported images from GraphPad/Fig5-ACSL4.jpg]

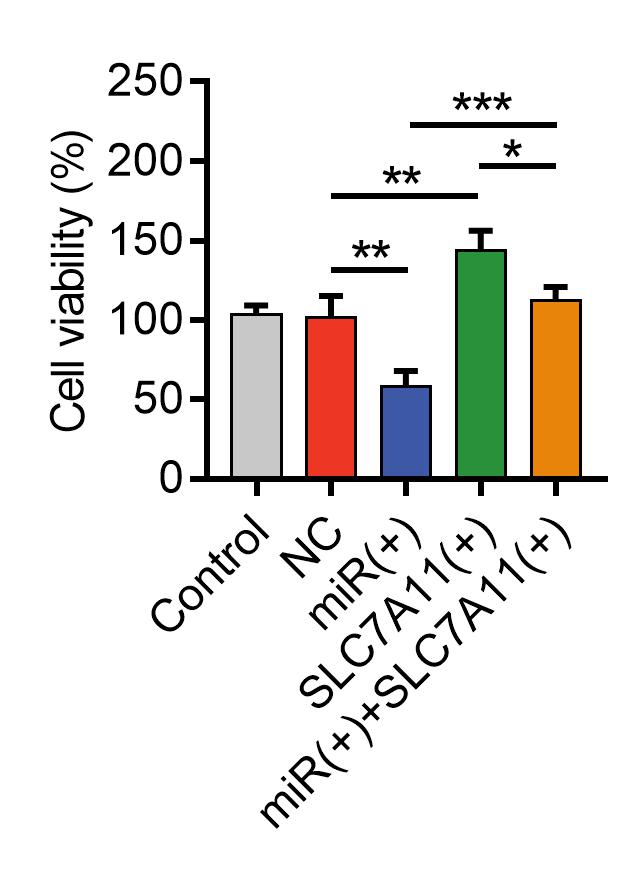

Supplement: Supplementary file 2 [file DataSheet_1.zip › Exported images from GraphPad/Fig5-CCK.jpg]

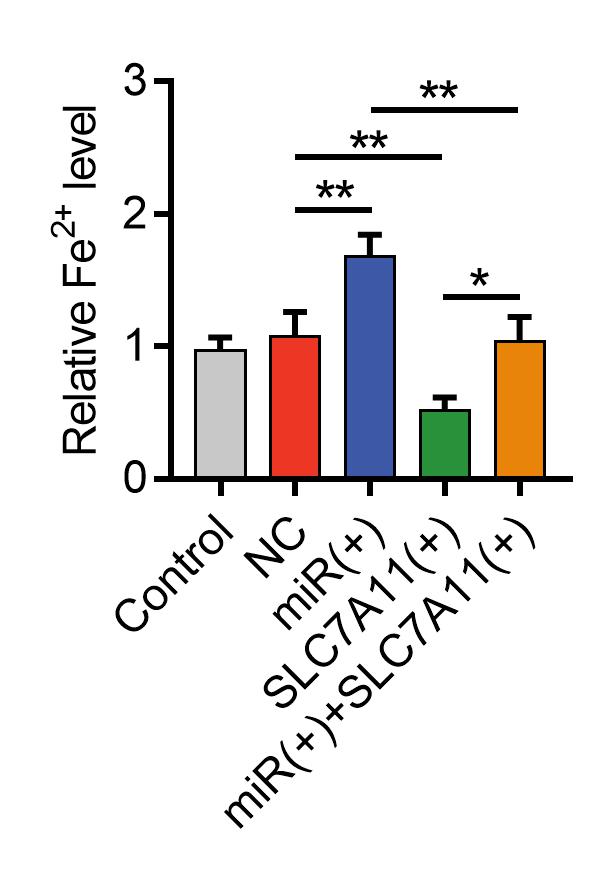

Supplement: Supplementary file 2 [file DataSheet_1.zip › Exported images from GraphPad/Fig5-Fe.jpg]

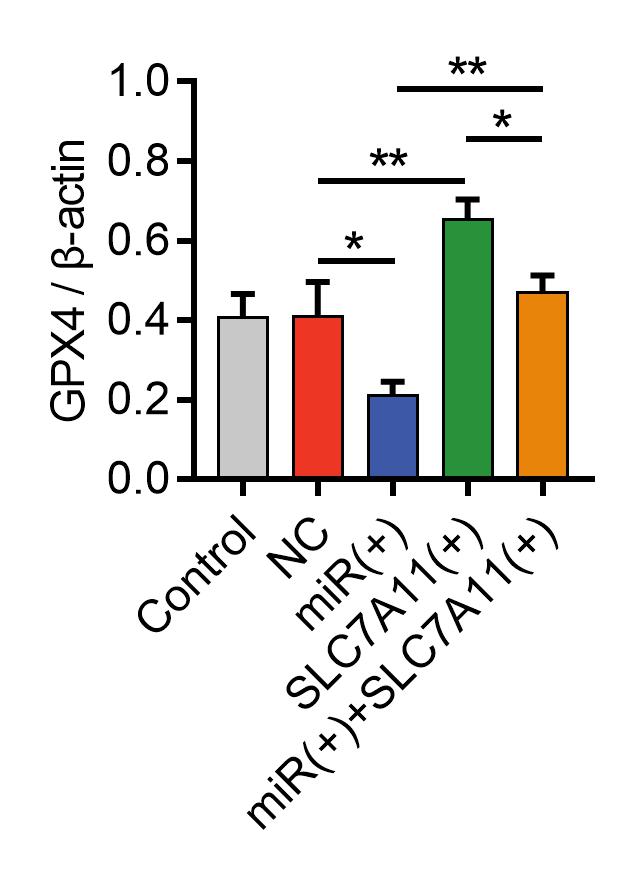

Supplement: Supplementary file 2 [file DataSheet_1.zip › Exported images from GraphPad/Fig5-GPX4.jpg]

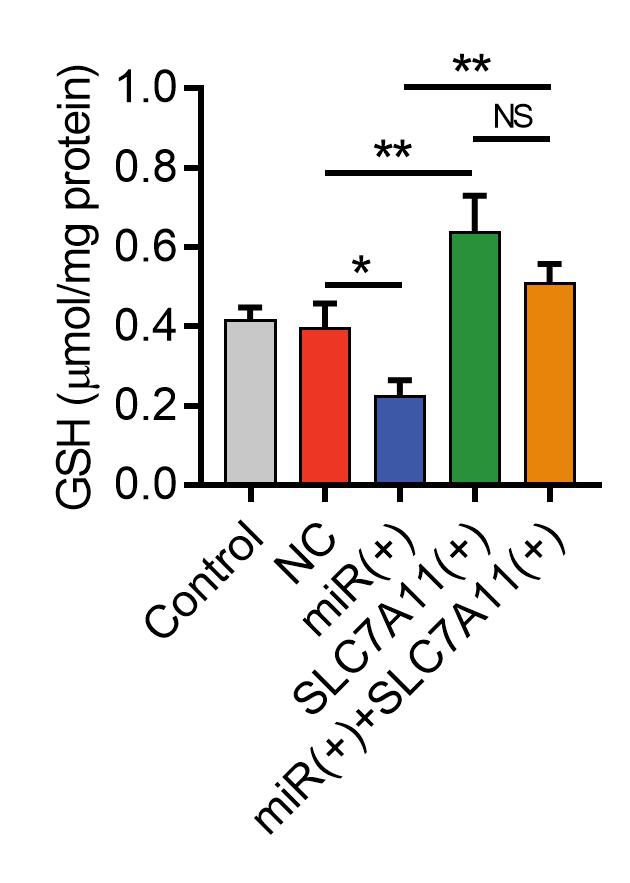

Supplement: Supplementary file 2 [file DataSheet_1.zip › Exported images from GraphPad/Fig5-GSH.jpg]

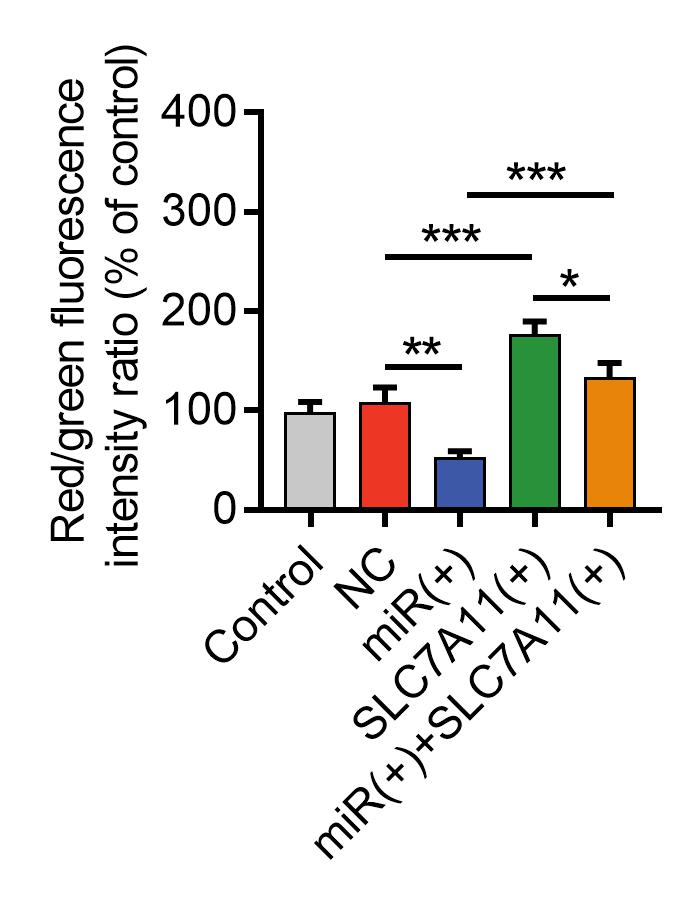

Supplement: Supplementary file 2 [file DataSheet_1.zip › Exported images from GraphPad/Fig5-JC-1.jpg]

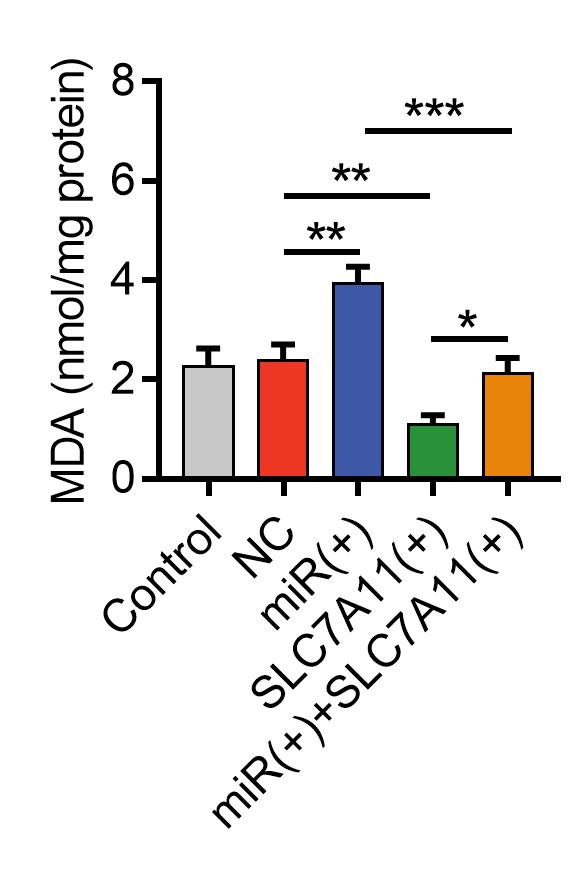

Supplement: Supplementary file 2 [file DataSheet_1.zip › Exported images from GraphPad/Fig5-MDA.jpg]

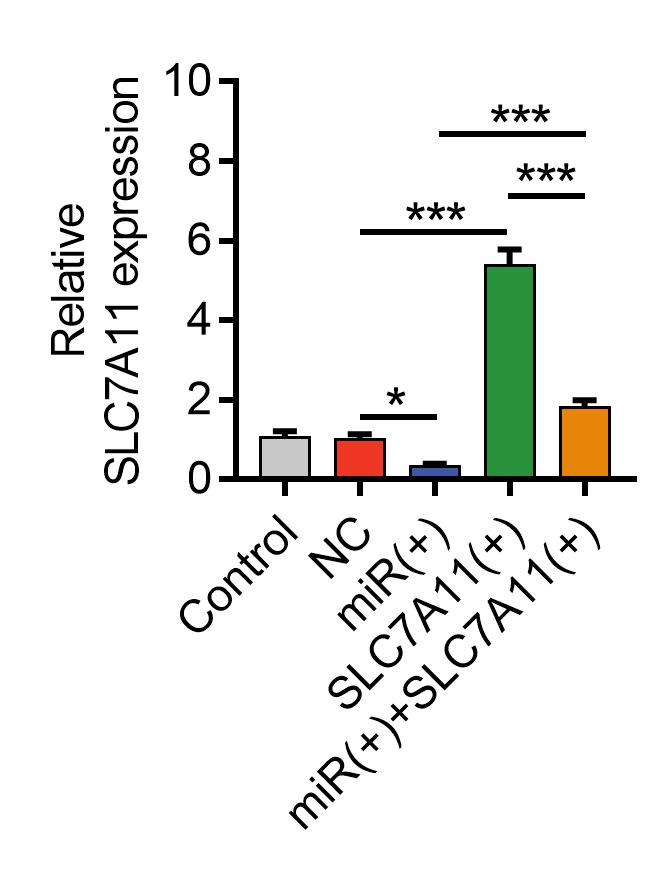

Supplement: Supplementary file 2 [file DataSheet_1.zip › Exported images from GraphPad/Fig5-PCR.jpg]

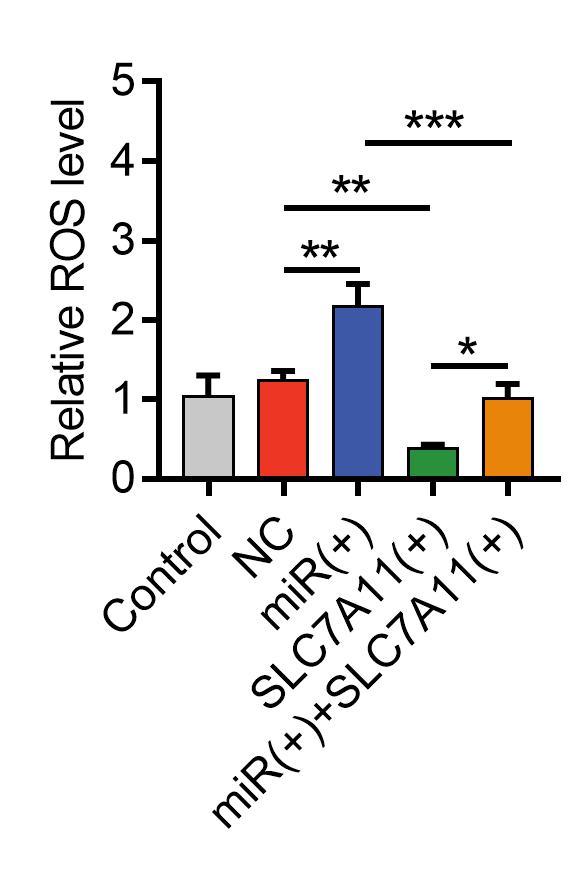

Supplement: Supplementary file 2 [file DataSheet_1.zip › Exported images from GraphPad/Fig5-ROS.jpg]

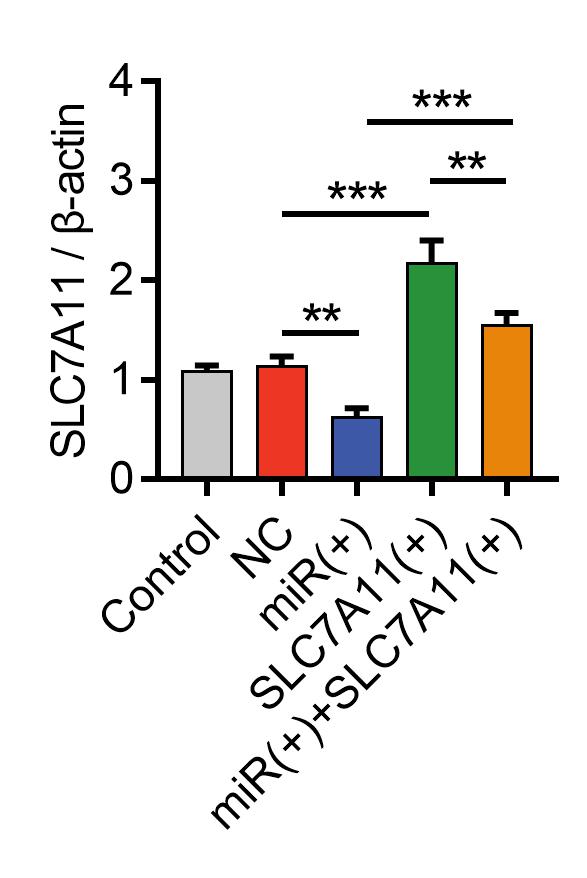

Supplement: Supplementary file 2 [file DataSheet_1.zip › Exported images from GraphPad/Fig5-SLC7A11.jpg]

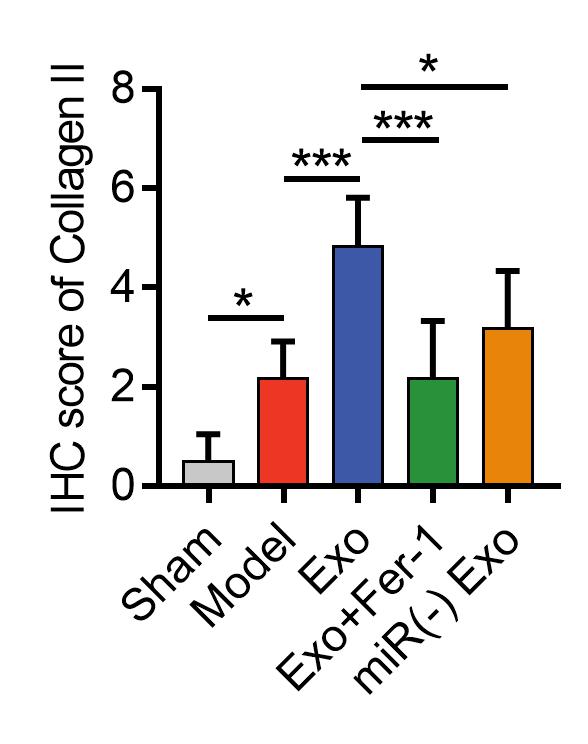

Supplement: Supplementary file 2 [file DataSheet_1.zip › Exported images from GraphPad/Fig6-COL2.jpg]

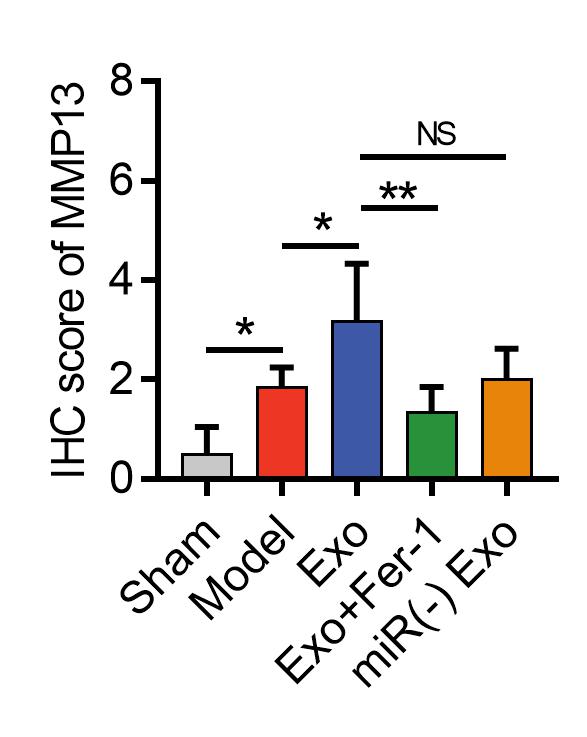

Supplement: Supplementary file 2 [file DataSheet_1.zip › Exported images from GraphPad/Fig6-MMP13.jpg]

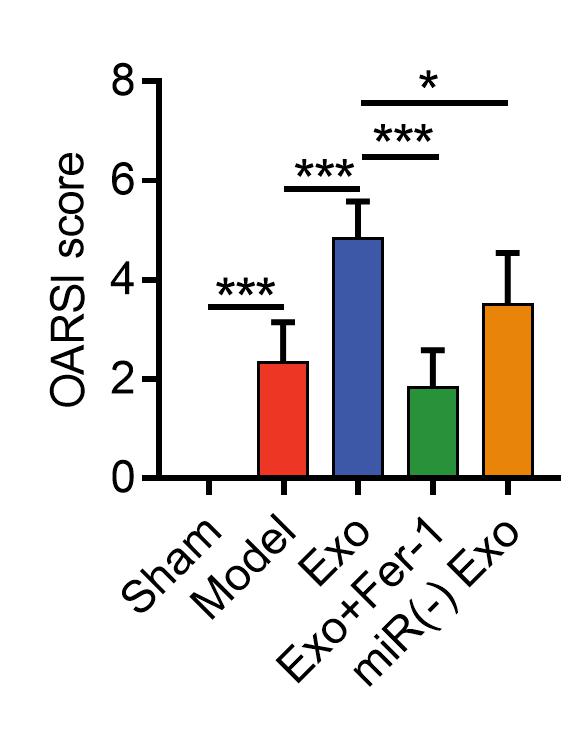

Supplement: Supplementary file 2 [file DataSheet_1.zip › Exported images from GraphPad/Fig6-OARSI.jpg]

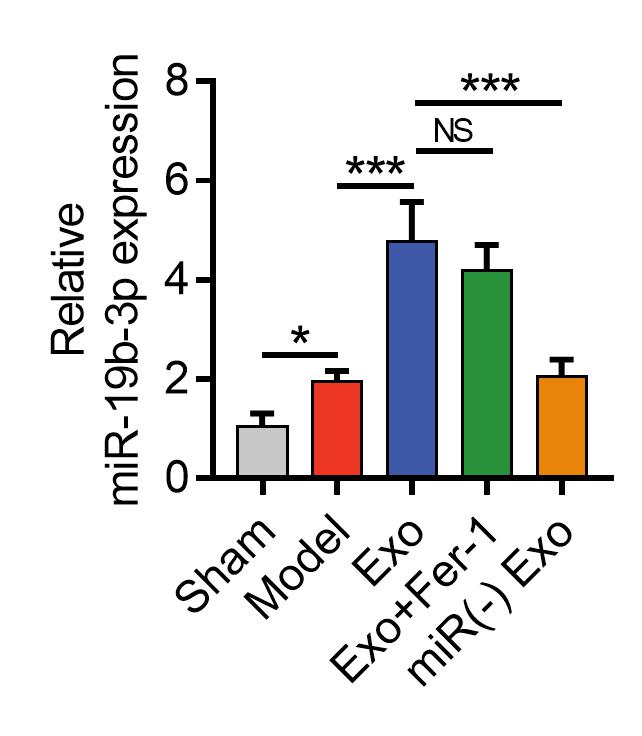

Supplement: Supplementary file 2 [file DataSheet_1.zip › Exported images from GraphPad/Fig6-PCR.jpg]

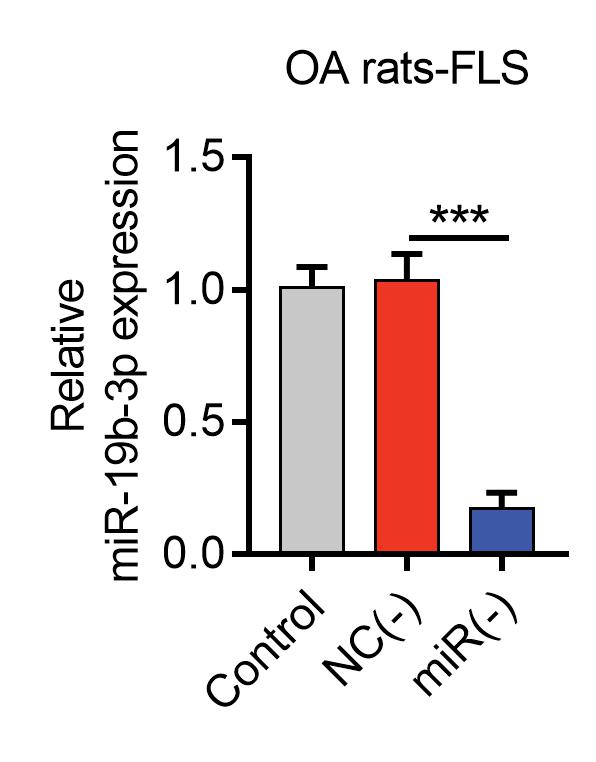

Supplement: Supplementary file 2 [file DataSheet_1.zip › Exported images from GraphPad/Fig6-S4-PCR.jpg]

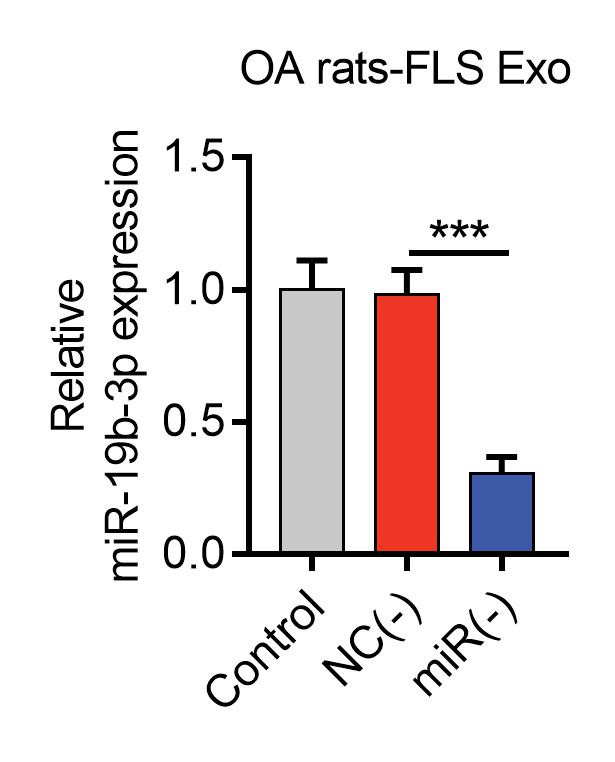

Supplement: Supplementary file 2 [file DataSheet_1.zip › Exported images from GraphPad/Fig6-S4-PCR2.jpg]

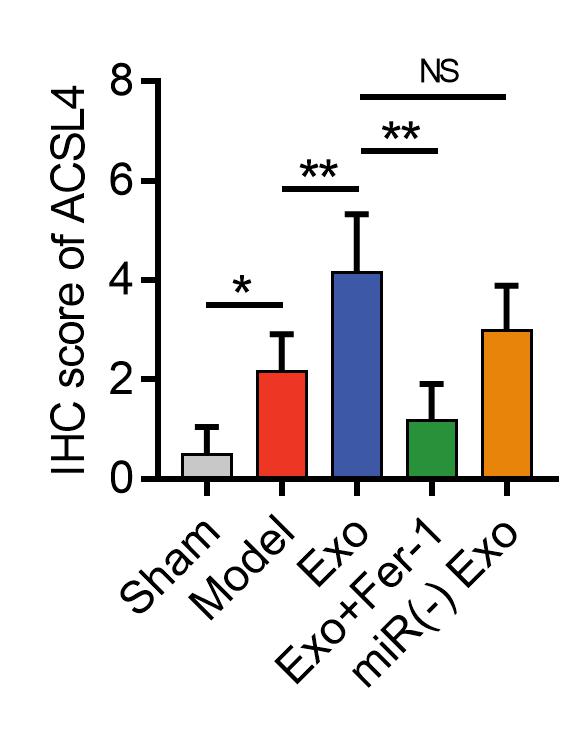

Supplement: Supplementary file 2 [file DataSheet_1.zip › Exported images from GraphPad/Fig7-ACSL4.jpg]

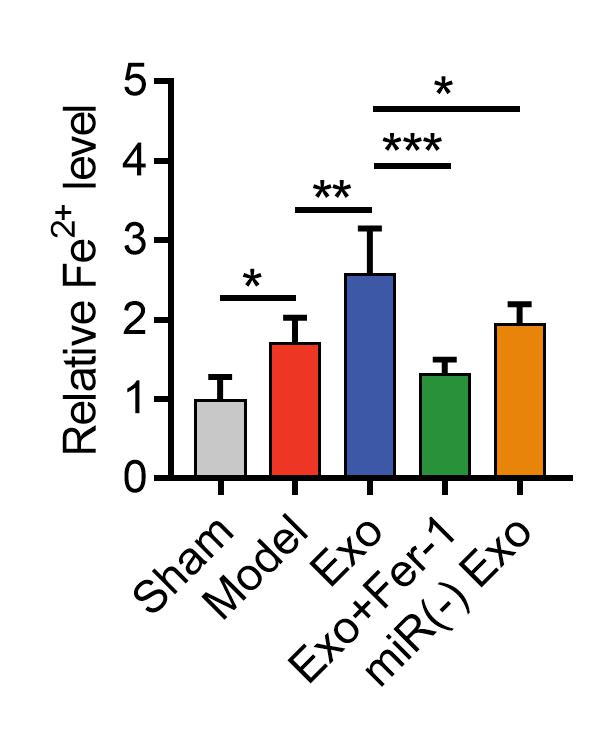

Supplement: Supplementary file 2 [file DataSheet_1.zip › Exported images from GraphPad/Fig7-Fe.jpg]

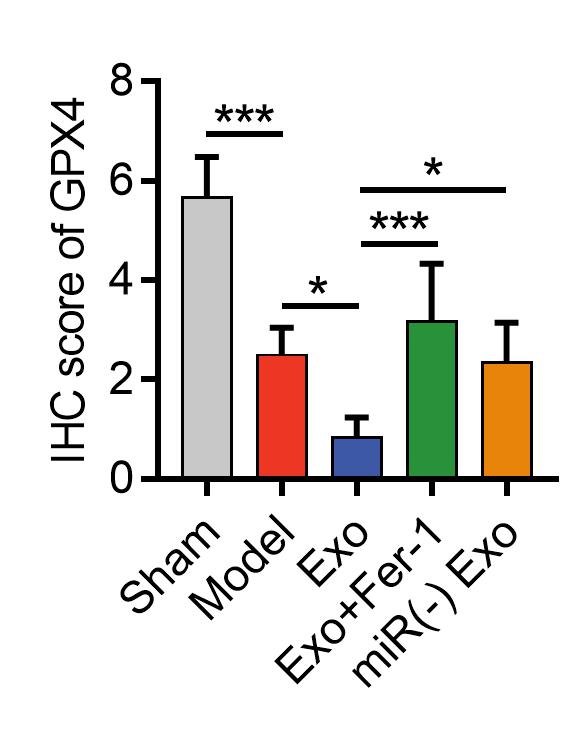

Supplement: Supplementary file 2 [file DataSheet_1.zip › Exported images from GraphPad/Fig7-GPX4.jpg]

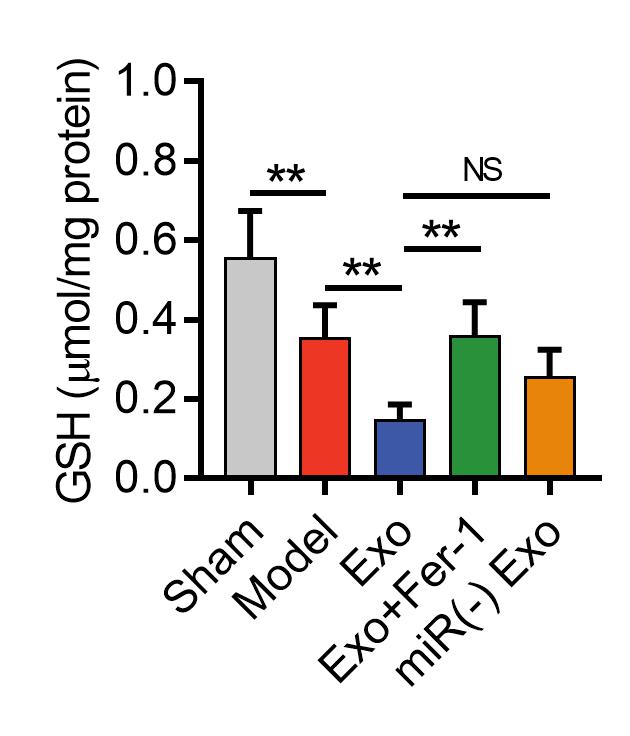

Supplement: Supplementary file 2 [file DataSheet_1.zip › Exported images from GraphPad/Fig7-GSH.jpg]

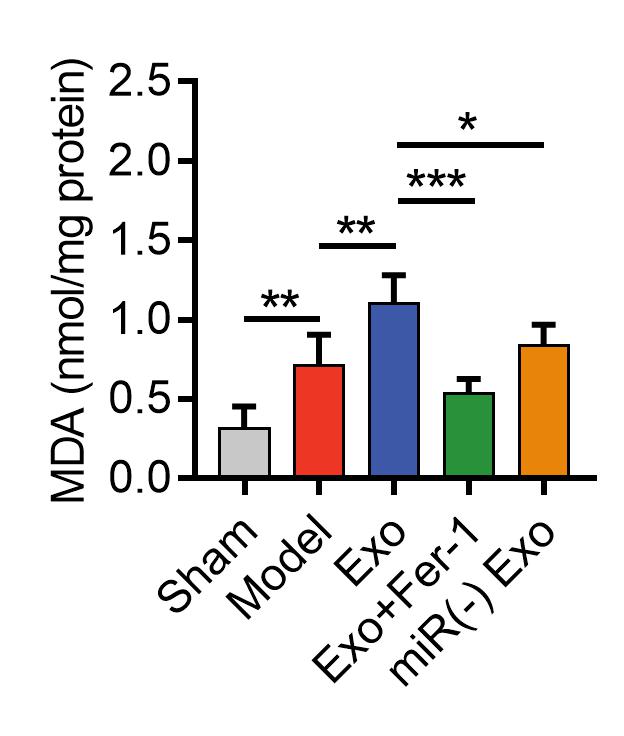

Supplement: Supplementary file 2 [file DataSheet_1.zip › Exported images from GraphPad/Fig7-MDA.jpg]

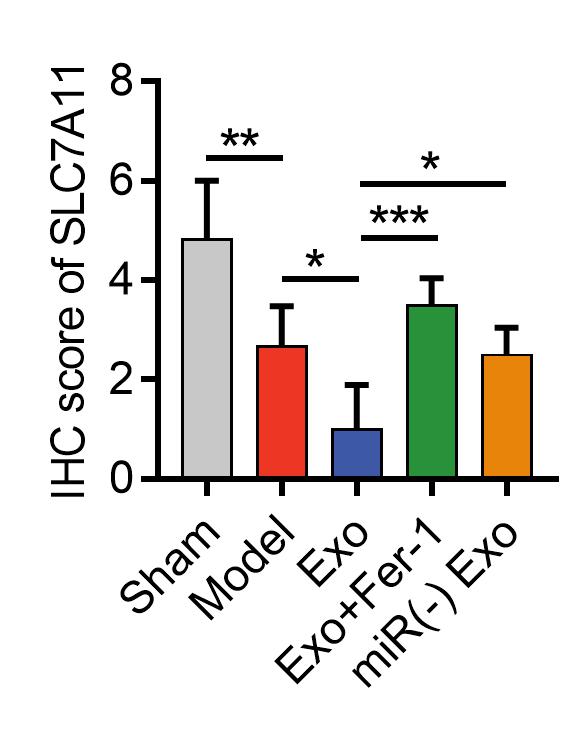

Supplement: Supplementary file 2 [file DataSheet_1.zip › Exported images from GraphPad/Fig7-SLC7A11.jpg]

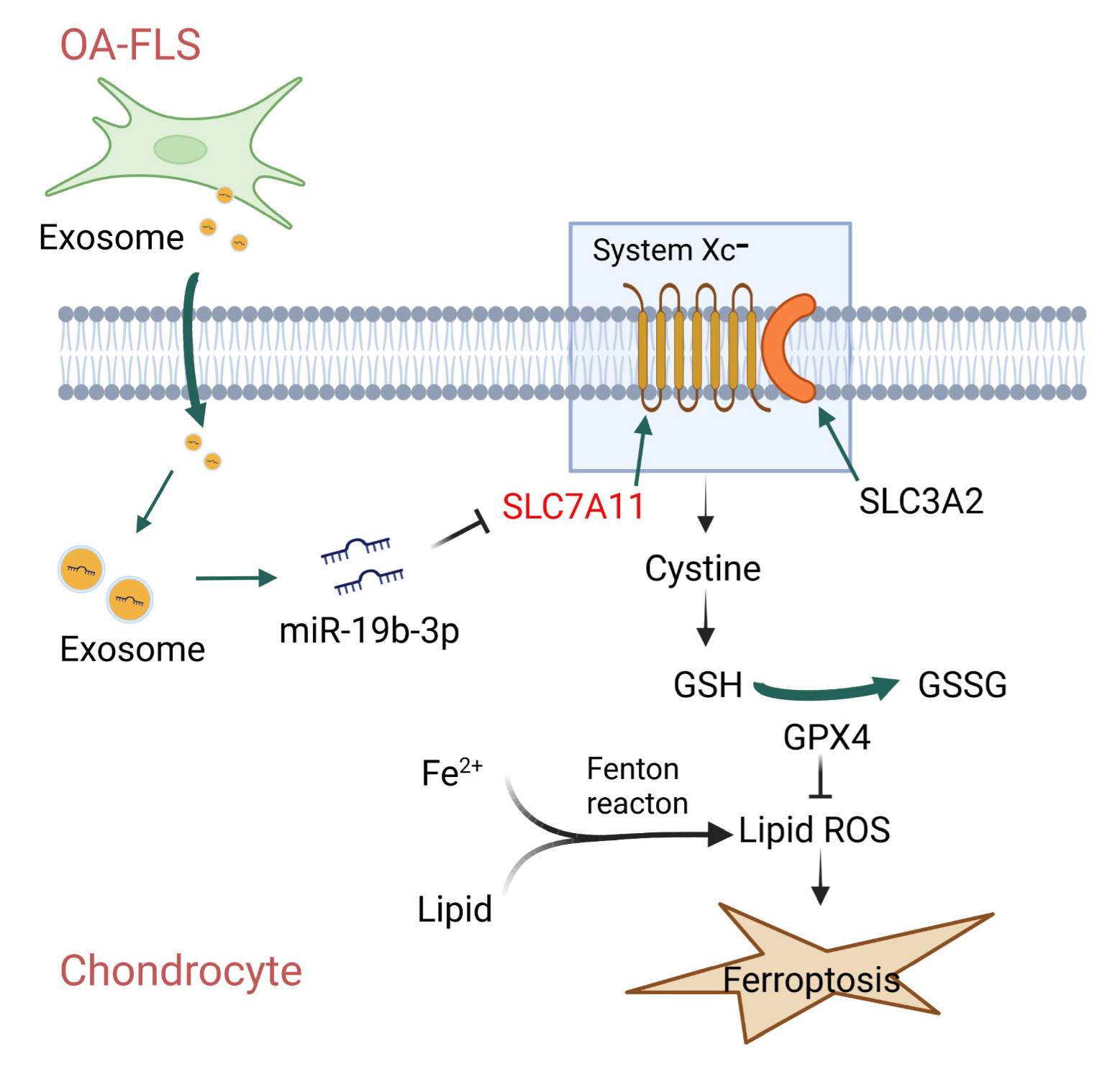

Supplement: Supplementary file 2 [file DataSheet_1.zip › Exported images from GraphPad/Fig8.jpg]

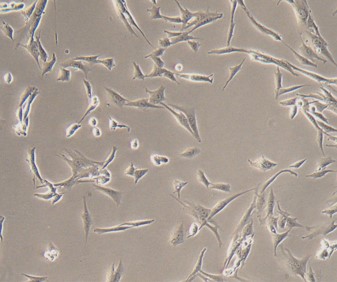

Supplement: Supplementary file 2 [file DataSheet_1.zip › Exported images from GraphPad/FigS1A.jpg]

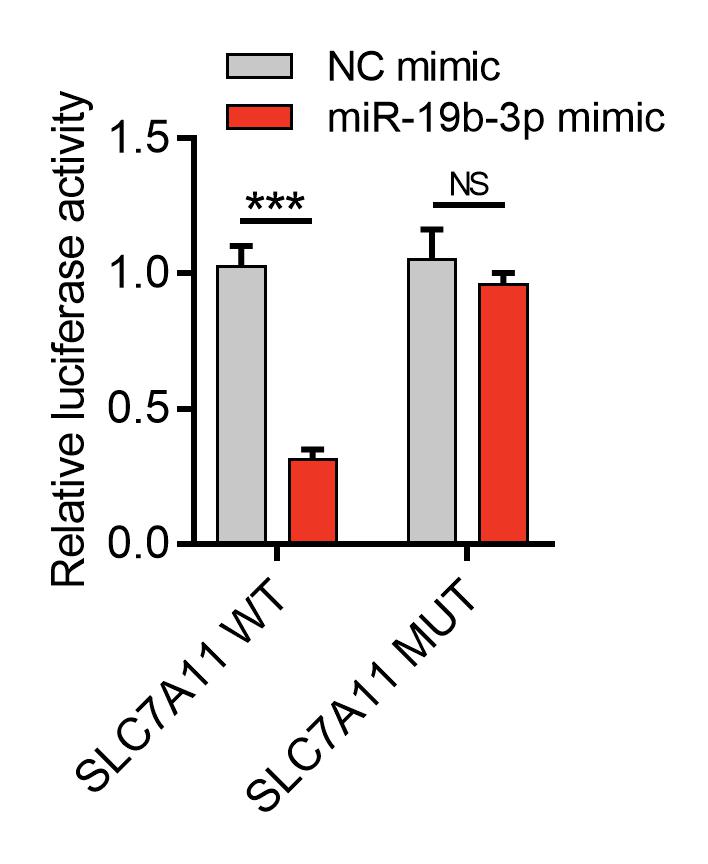

Supplement: Supplementary file 2 [file DataSheet_1.zip › Exported images from GraphPad/FigS3B.jpg]

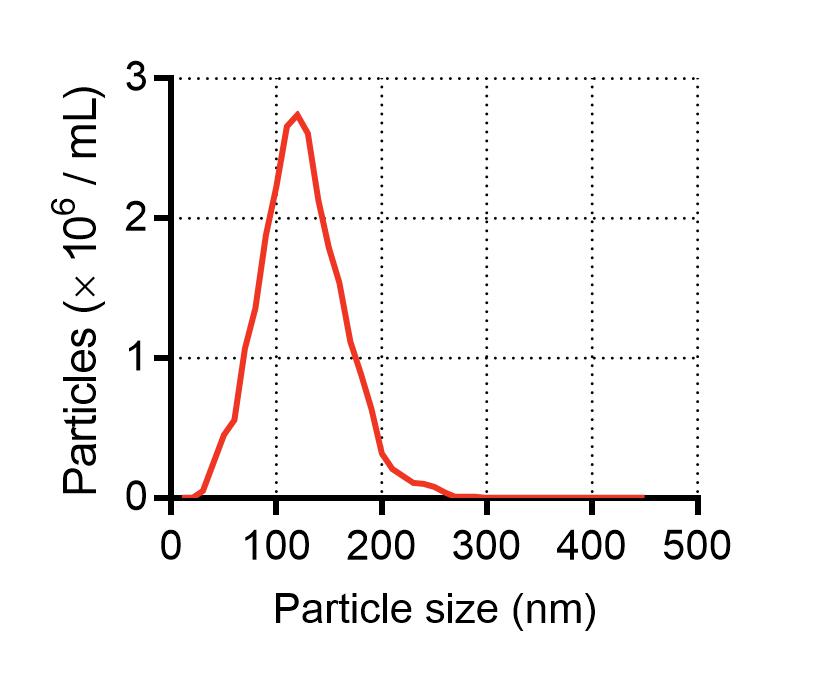

Supplement: Supplementary file 2 [file DataSheet_1.zip › Exported images from GraphPad/NTA.jpg]

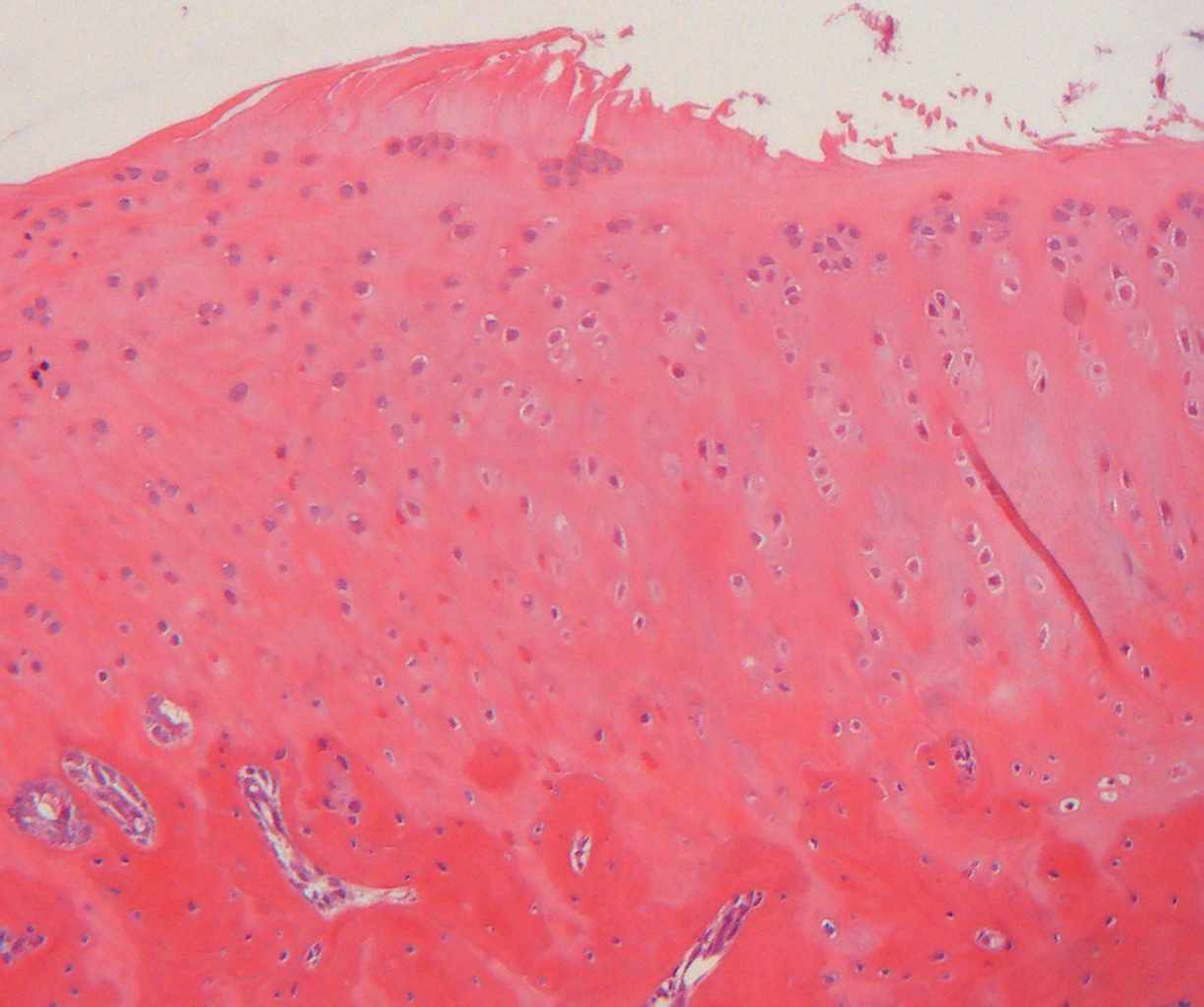

Supplement: Supplementary file 2 [file DataSheet_1.zip › HE staining/Exo/1.jpg]

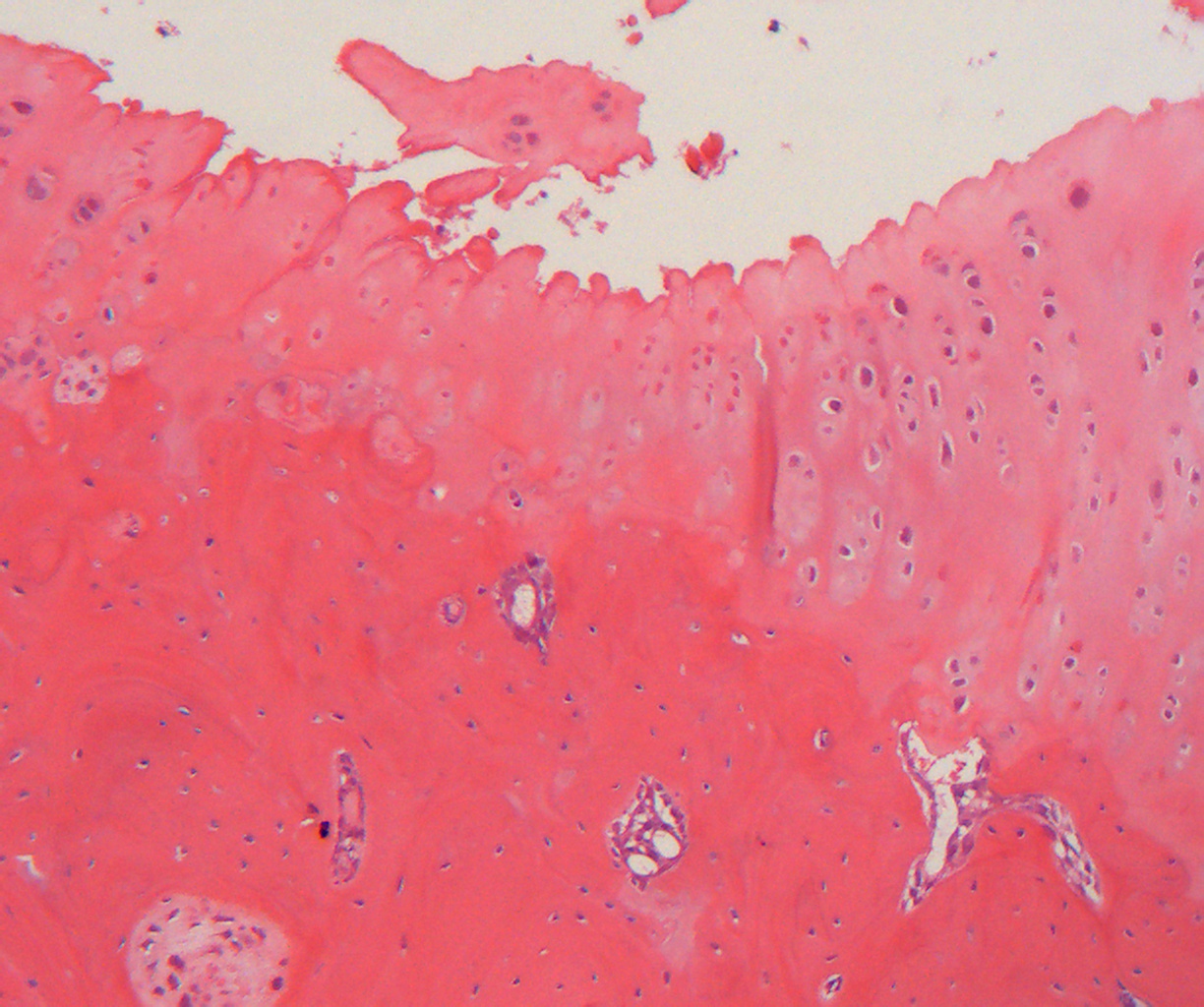

Supplement: Supplementary file 2 [file DataSheet_1.zip › HE staining/Exo/2.jpg]

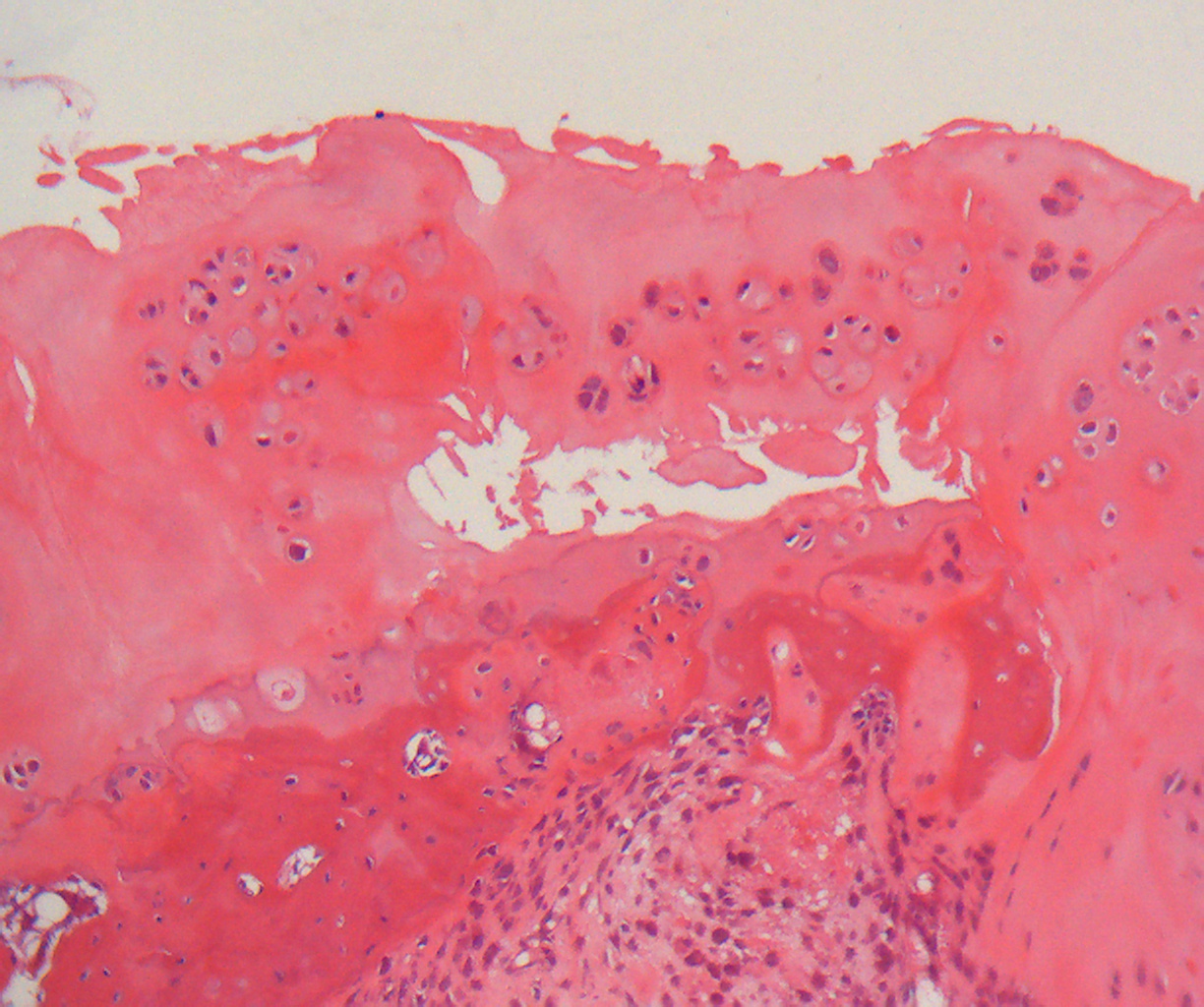

Supplement: Supplementary file 2 [file DataSheet_1.zip › HE staining/Exo/3.jpg]

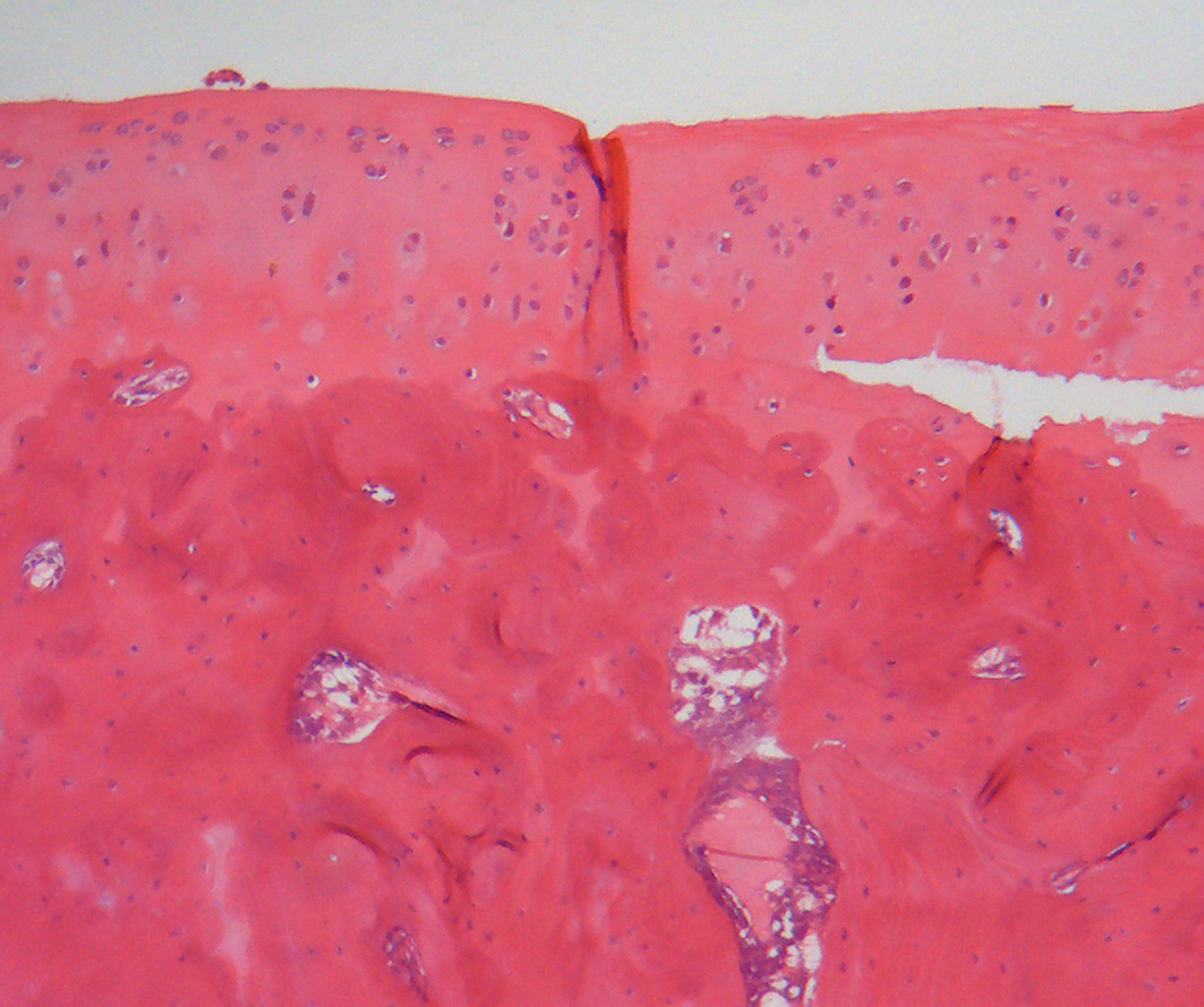

Supplement: Supplementary file 2 [file DataSheet_1.zip › HE staining/Exo/4.jpg]

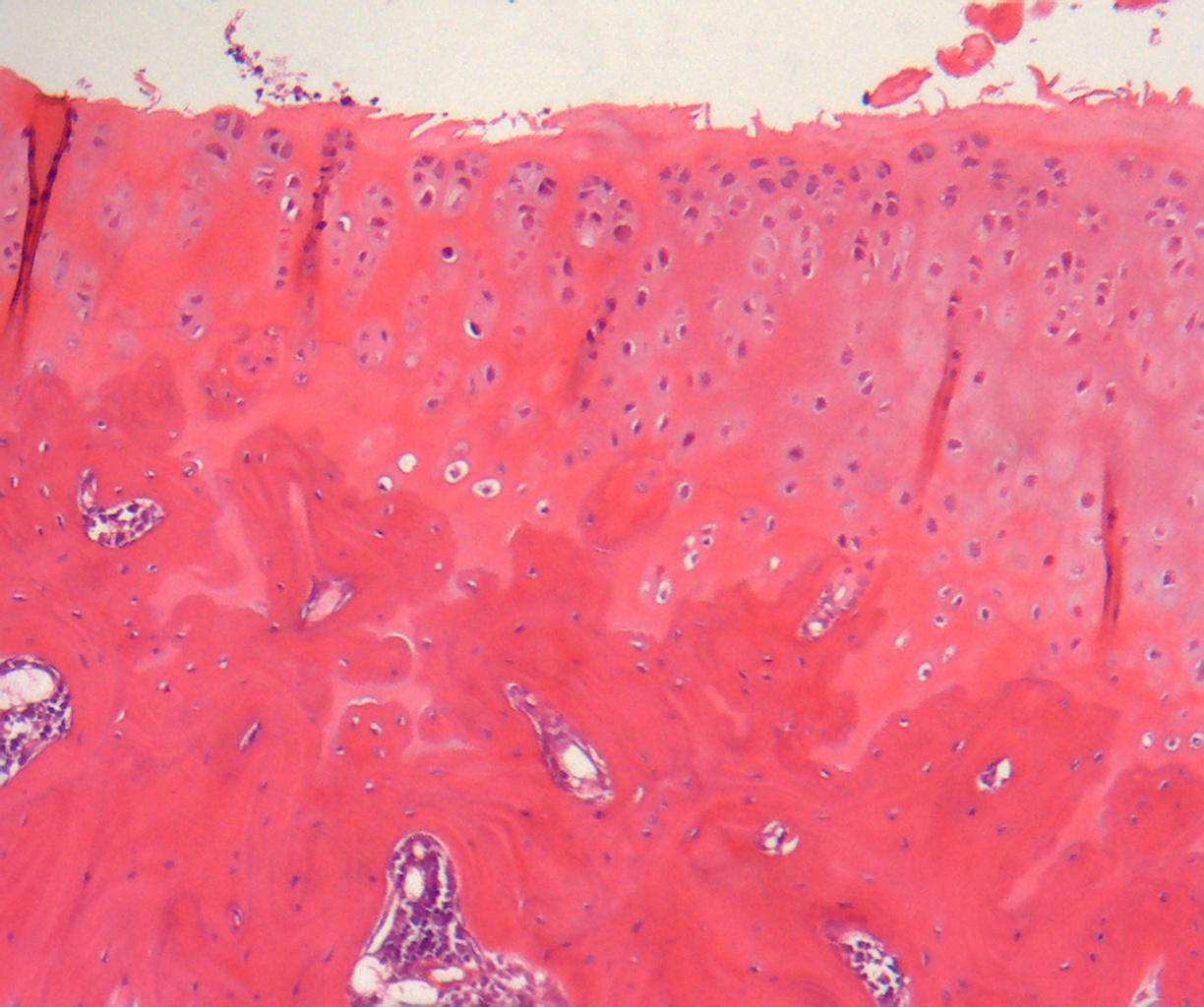

Supplement: Supplementary file 2 [file DataSheet_1.zip › HE staining/Exo/5.jpg]

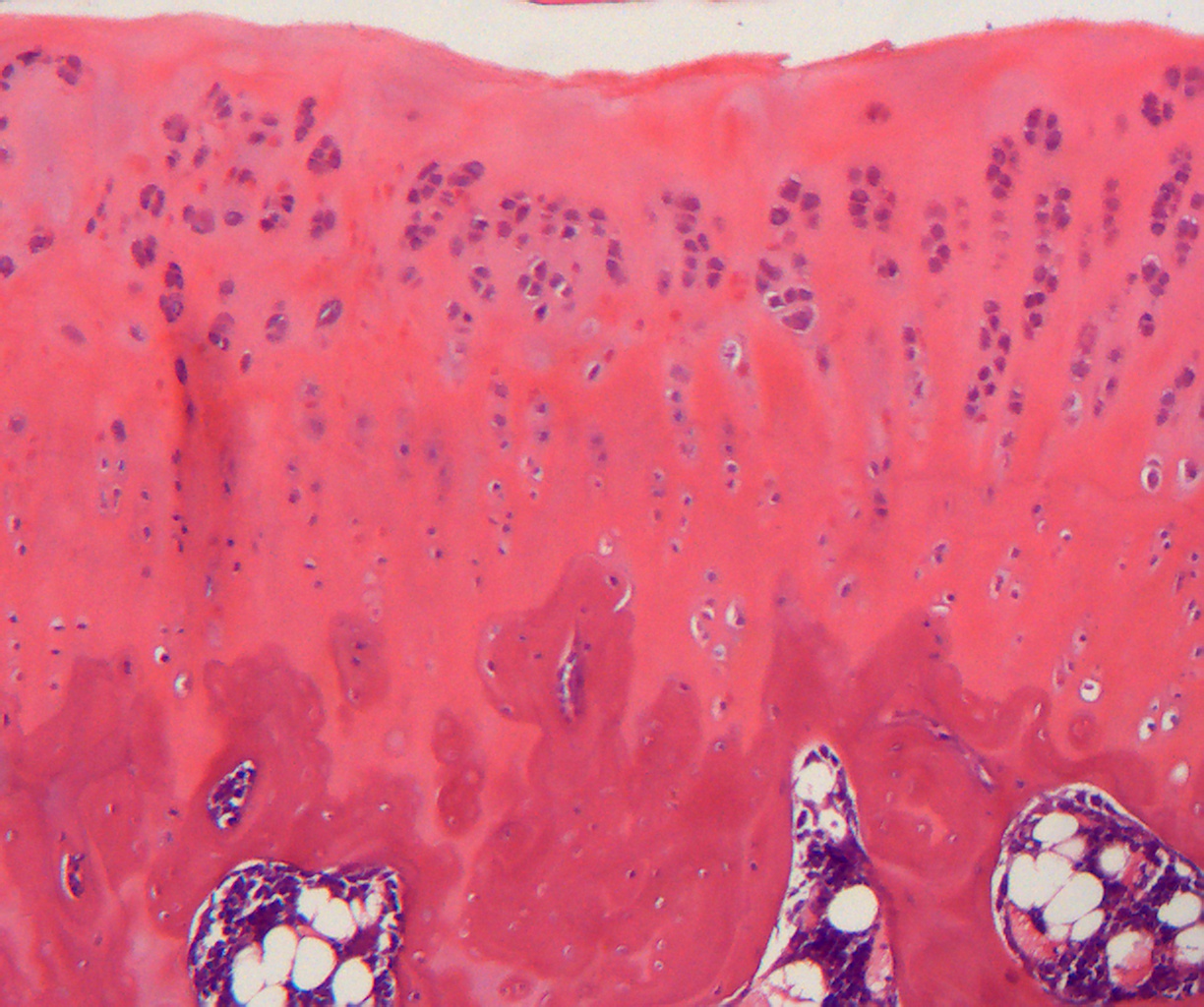

Supplement: Supplementary file 2 [file DataSheet_1.zip › HE staining/Exo/6.jpg]

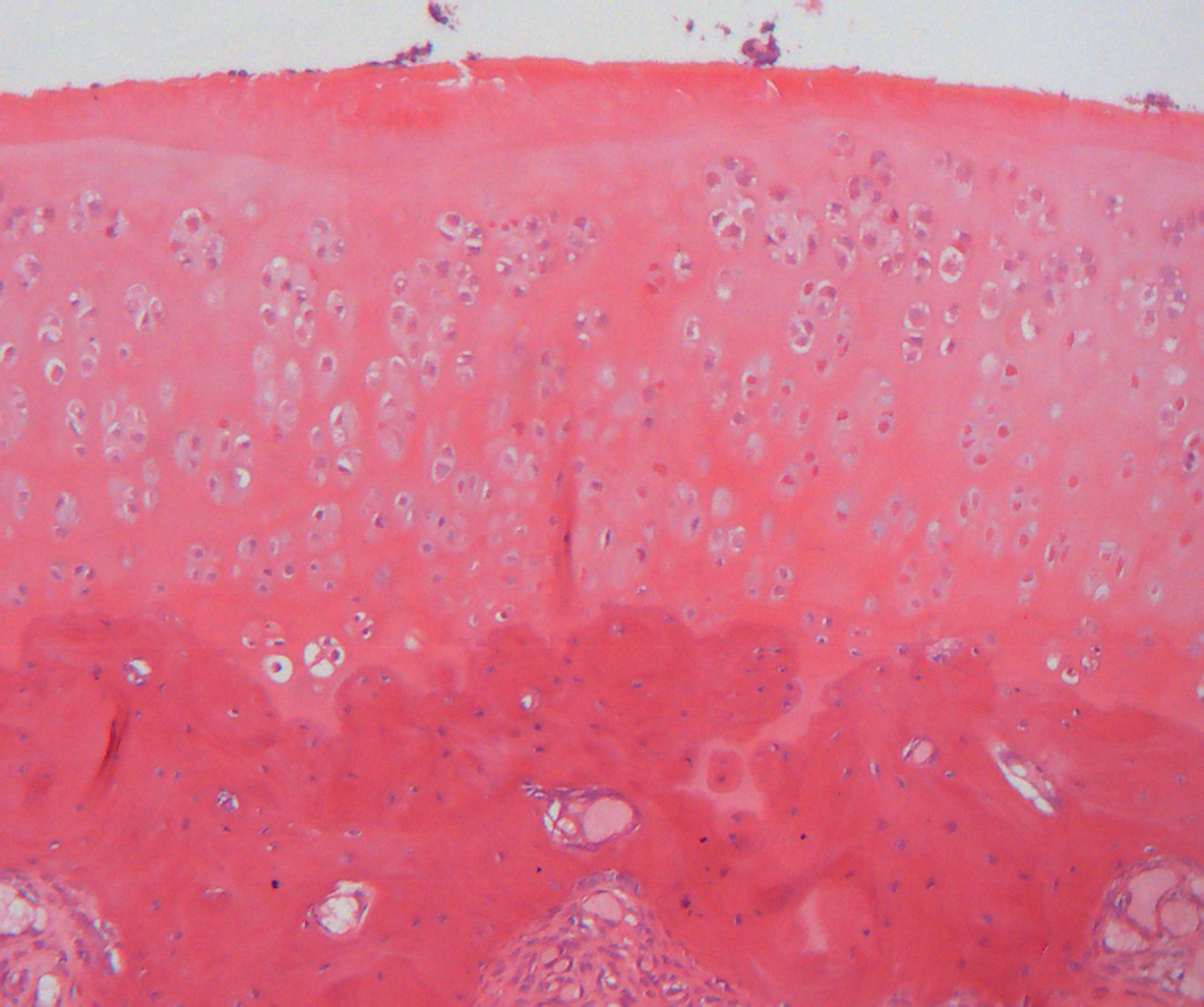

Supplement: Supplementary file 2 [file DataSheet_1.zip › HE staining/Exo+Fer-1/1.jpg]

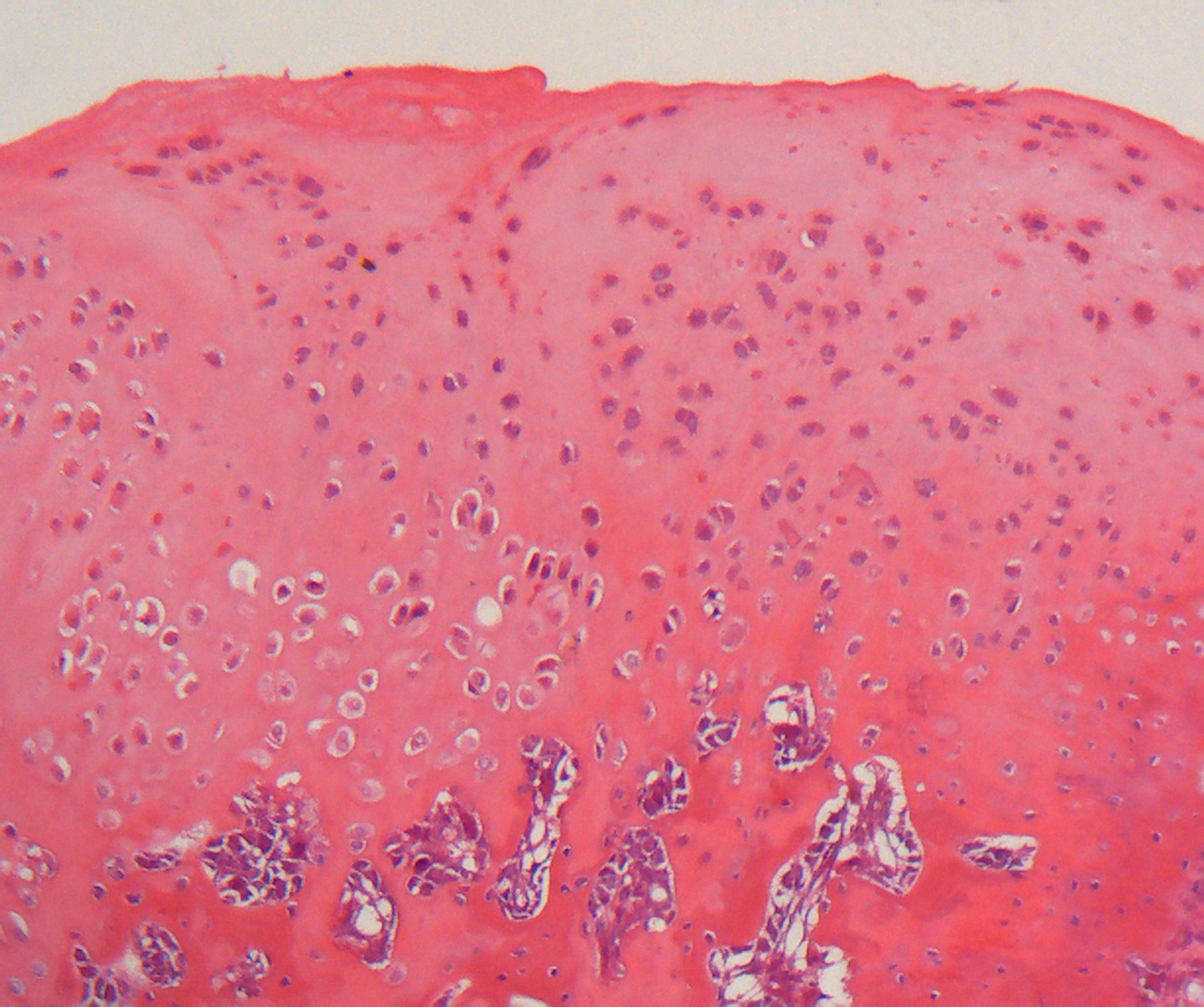

Supplement: Supplementary file 2 [file DataSheet_1.zip › HE staining/Exo+Fer-1/2.jpg]

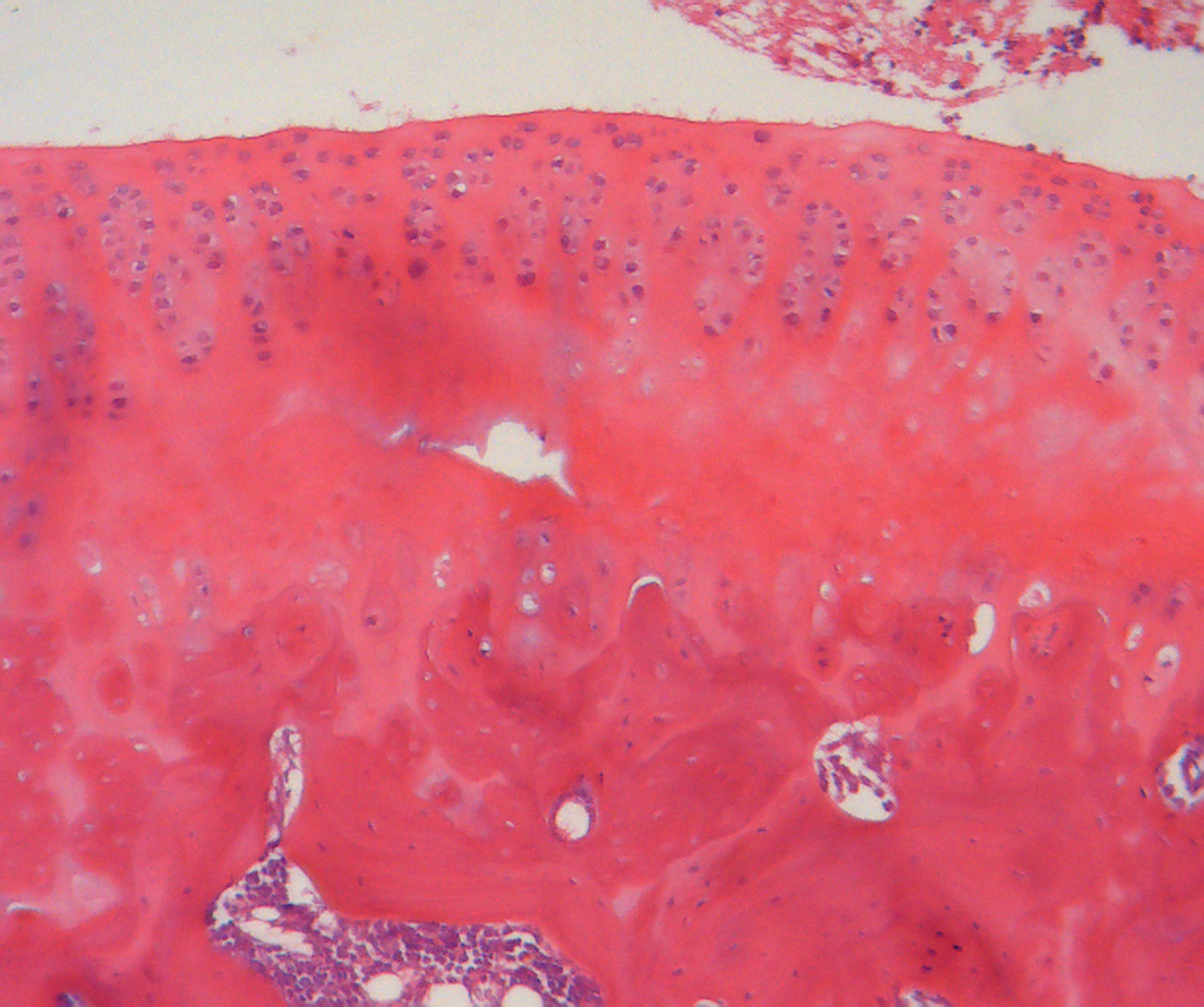

Supplement: Supplementary file 2 [file DataSheet_1.zip › HE staining/Exo+Fer-1/3.jpg]

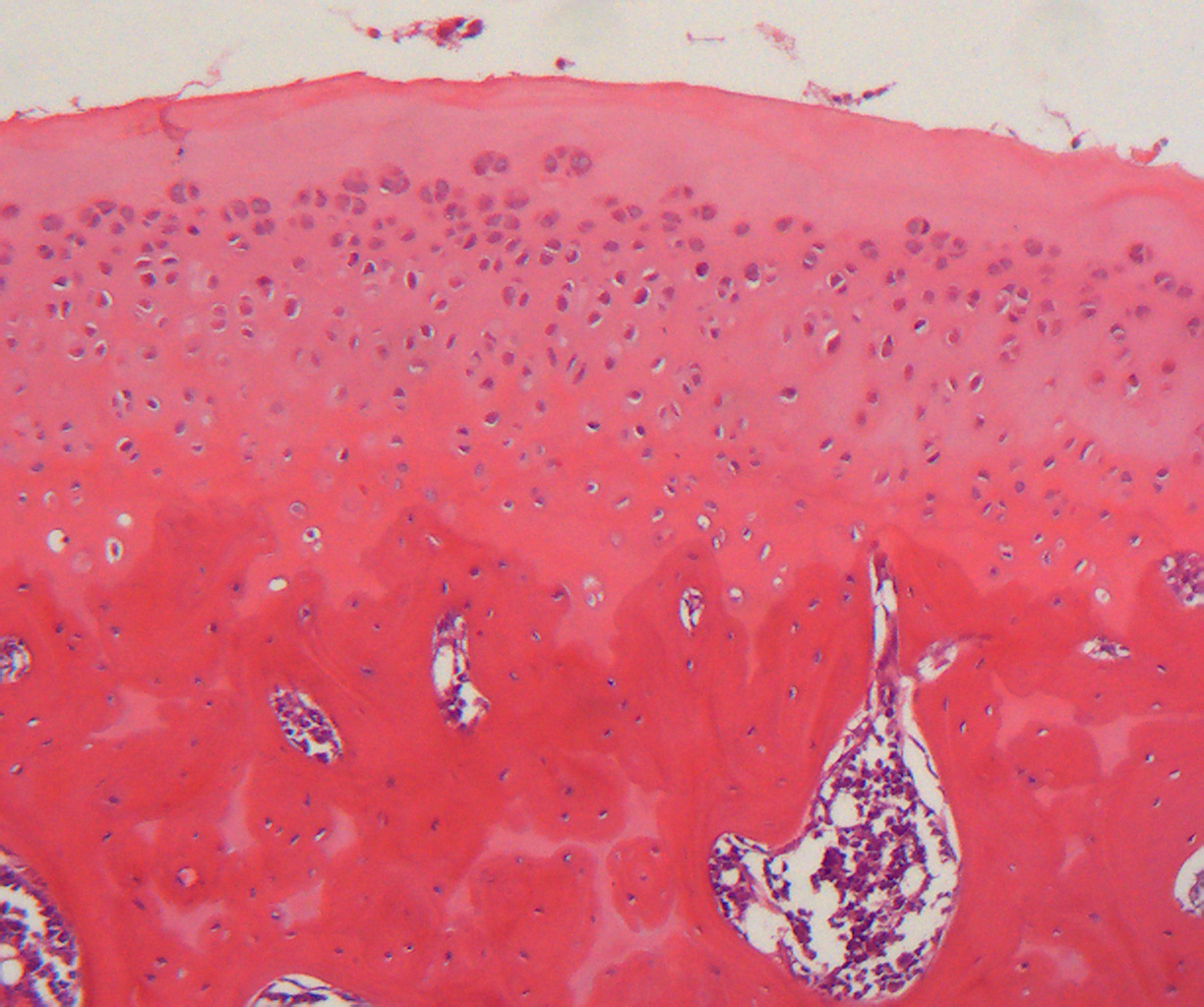

Supplement: Supplementary file 2 [file DataSheet_1.zip › HE staining/Exo+Fer-1/4.jpg]

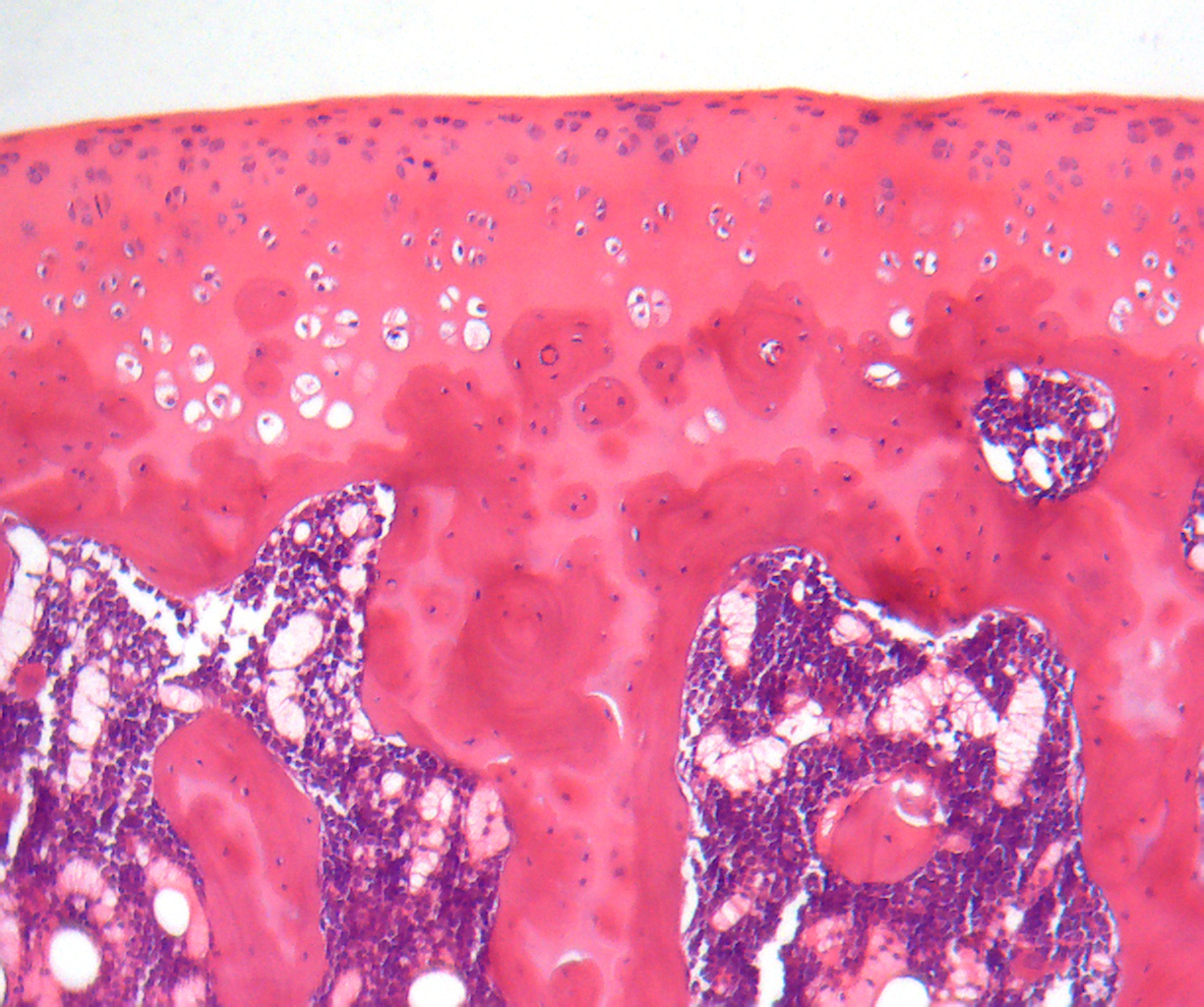

Supplement: Supplementary file 2 [file DataSheet_1.zip › HE staining/Exo+Fer-1/5.jpg]

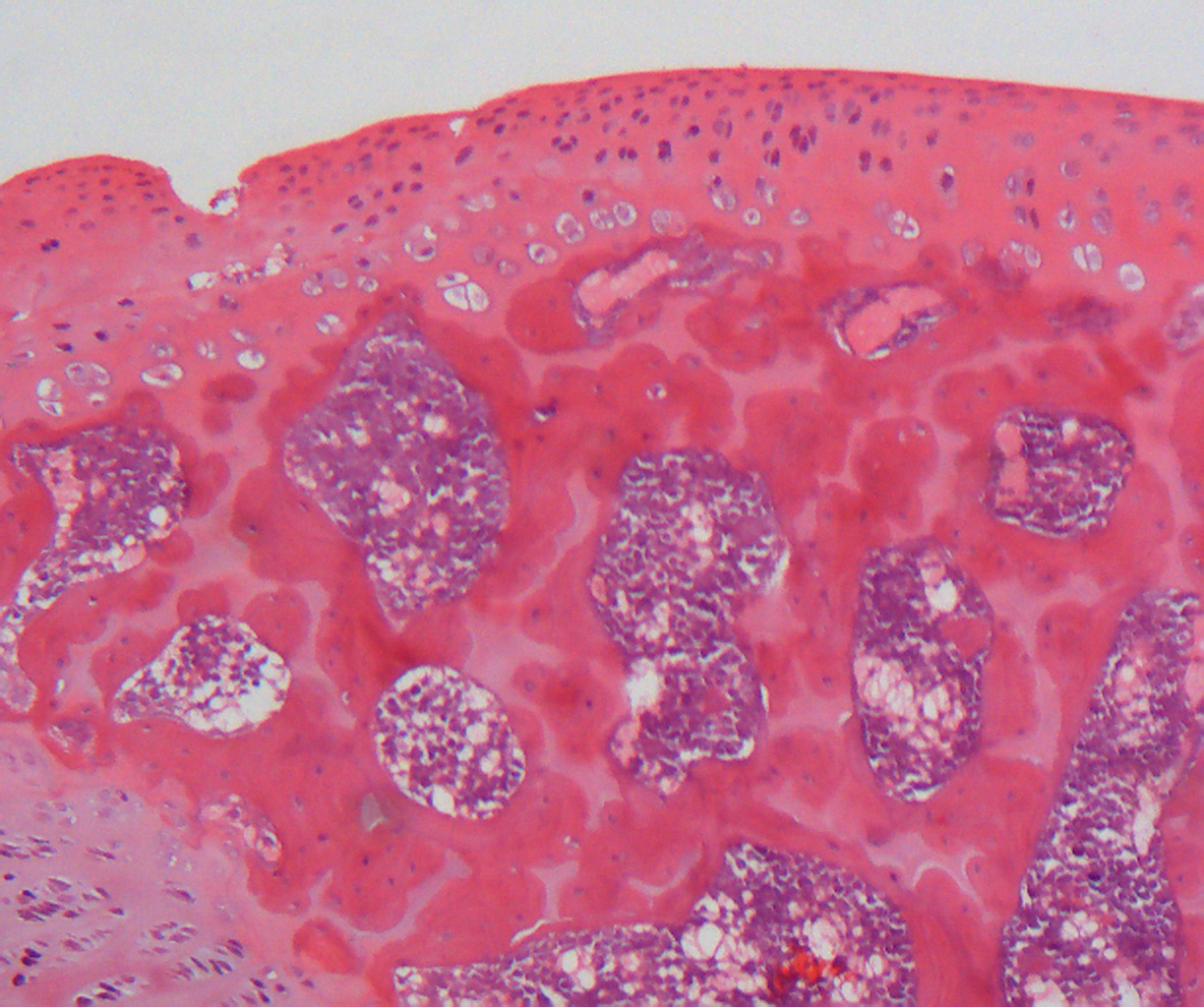

Supplement: Supplementary file 2 [file DataSheet_1.zip › HE staining/Exo+Fer-1/6.jpg]

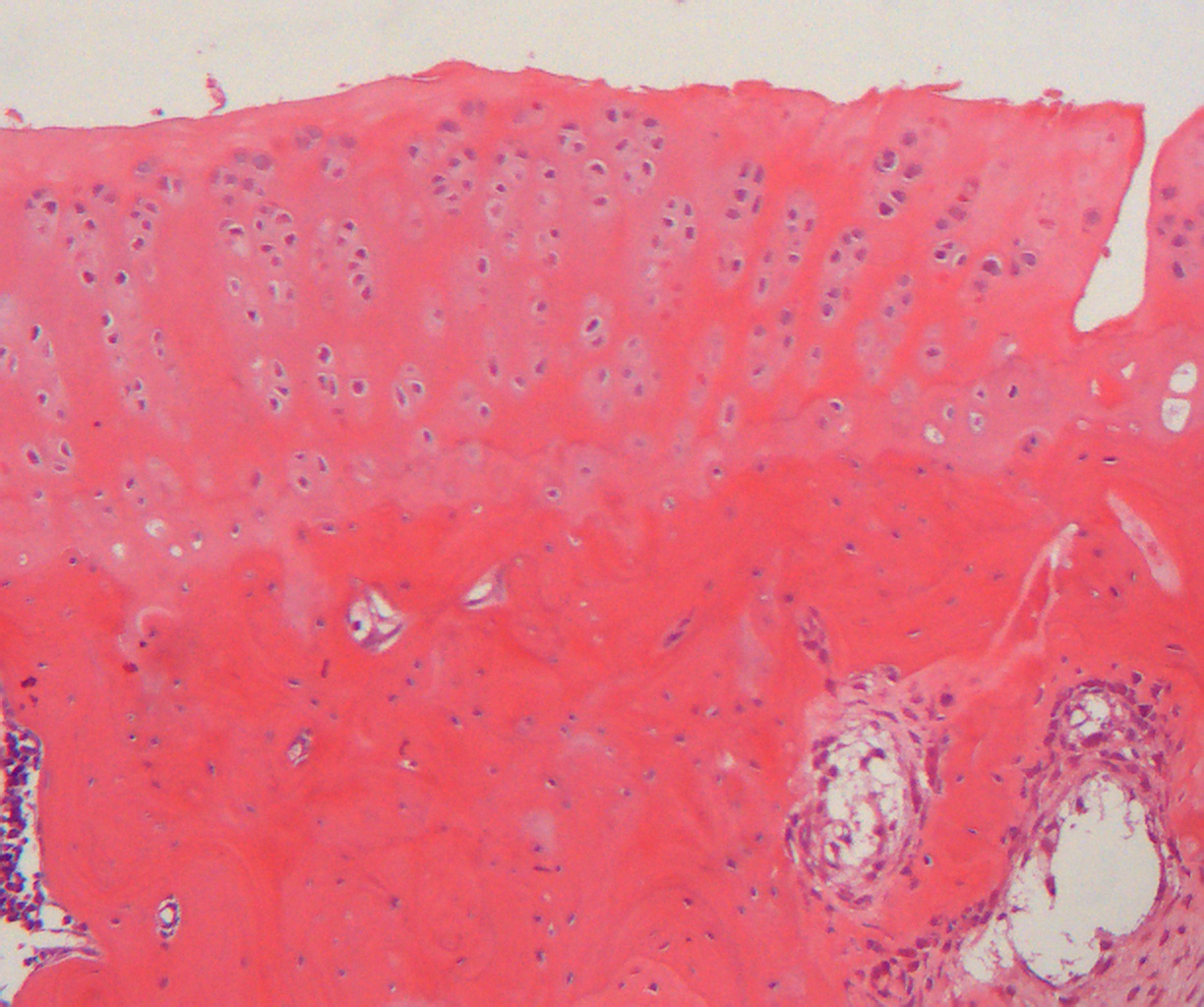

Supplement: Supplementary file 2 [file DataSheet_1.zip › HE staining/Model/1.jpg]

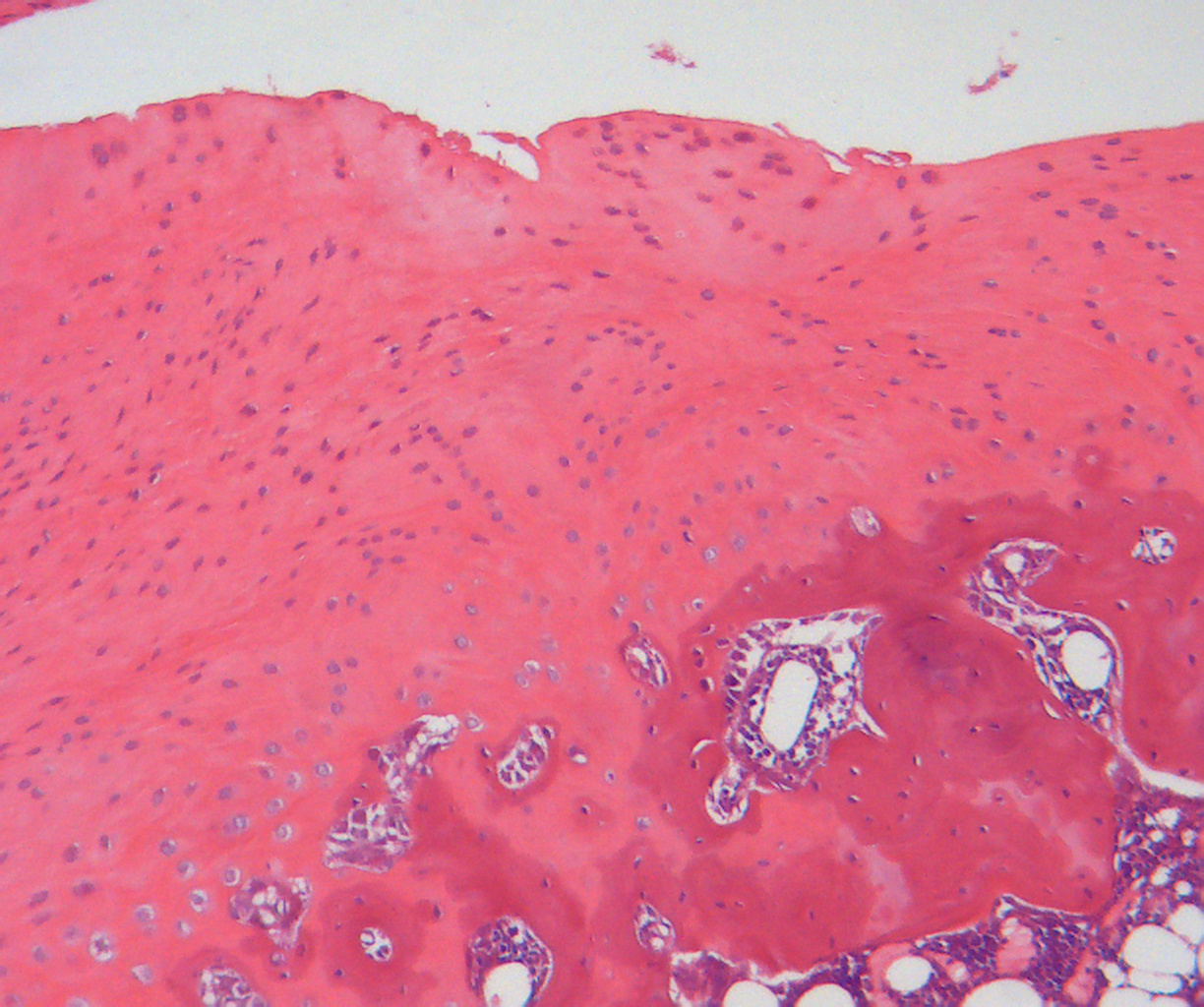

Supplement: Supplementary file 2 [file DataSheet_1.zip › HE staining/Model/2.jpg]

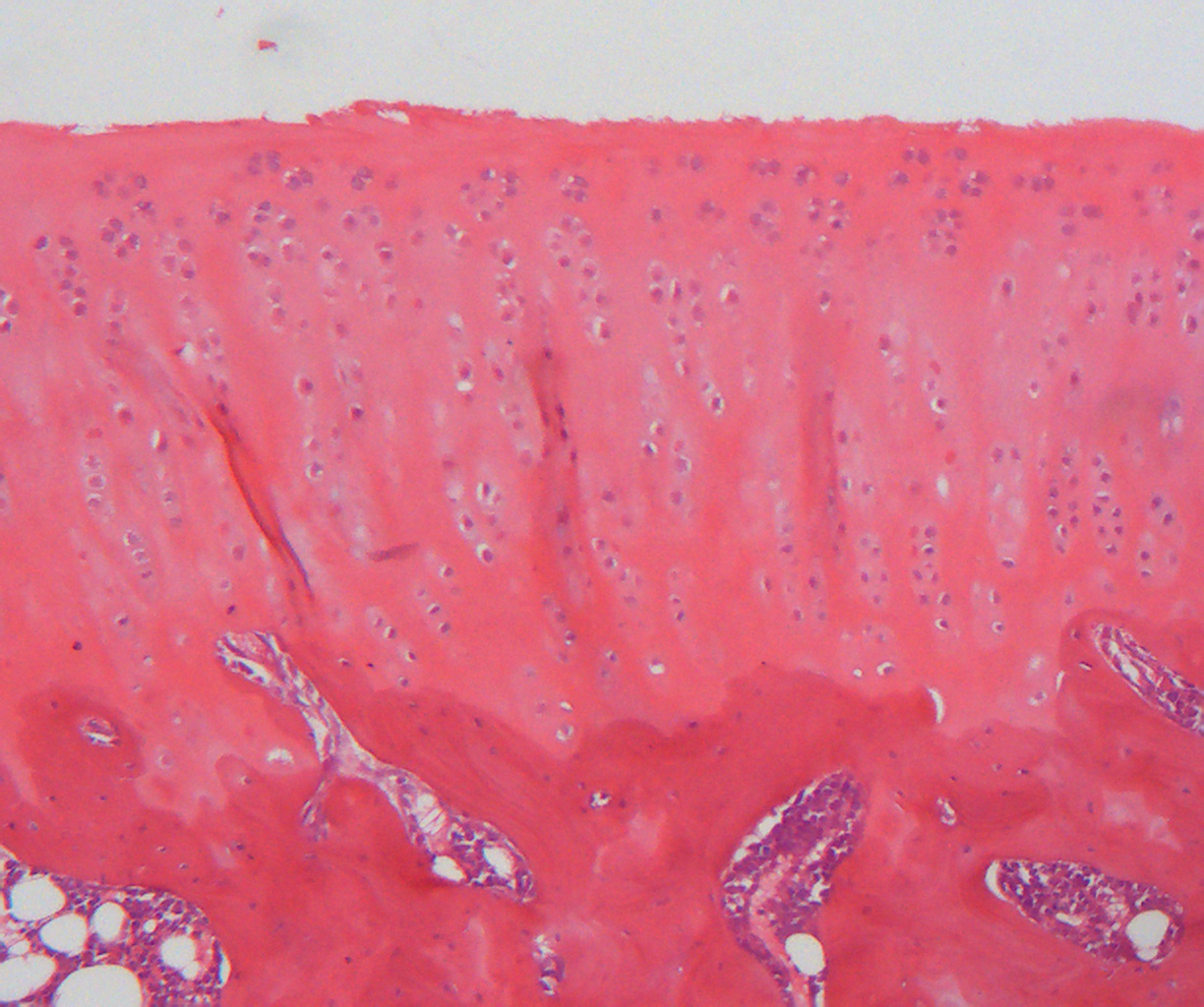

Supplement: Supplementary file 2 [file DataSheet_1.zip › HE staining/Model/3.jpg]

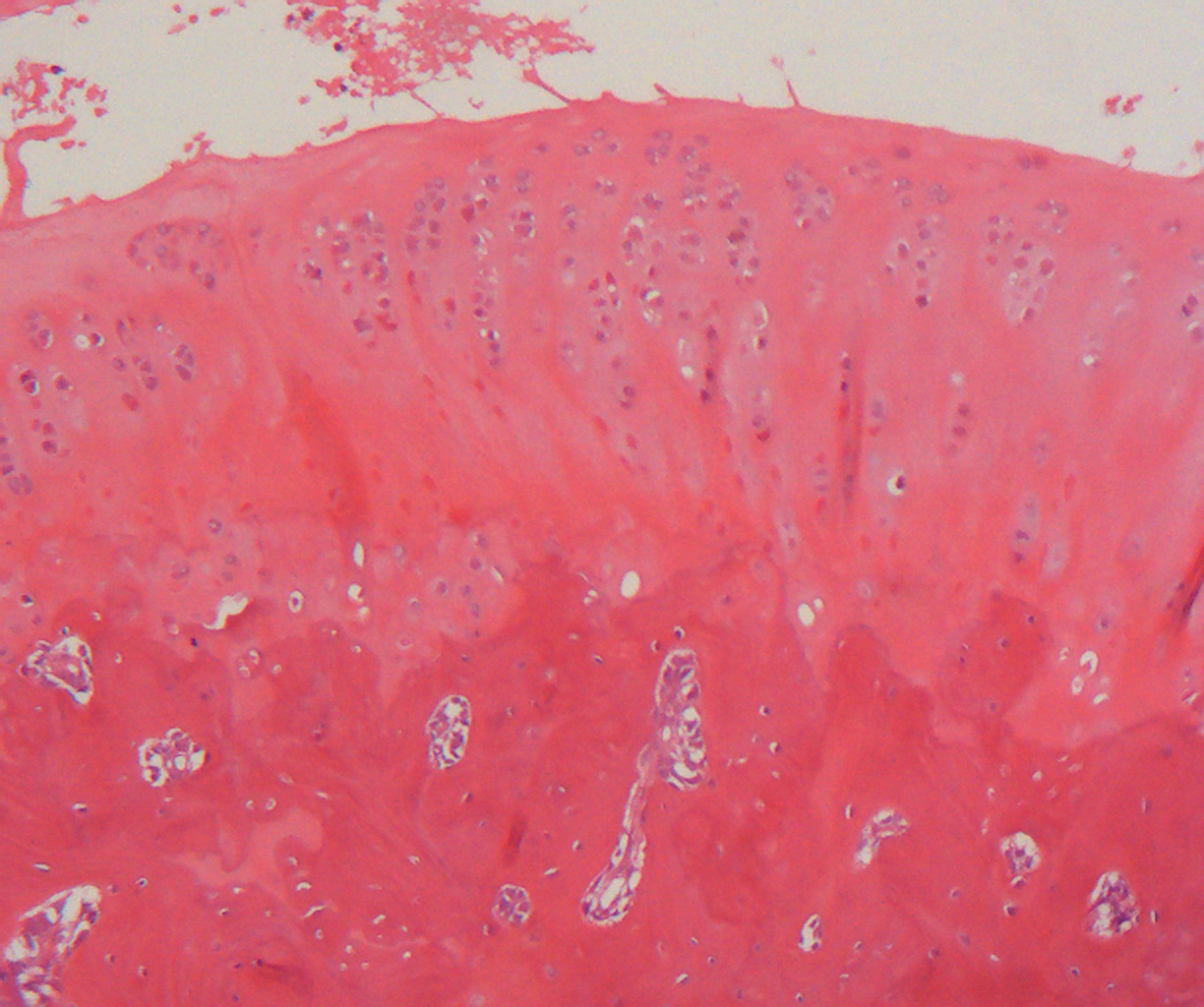

Supplement: Supplementary file 2 [file DataSheet_1.zip › HE staining/Model/4.jpg]

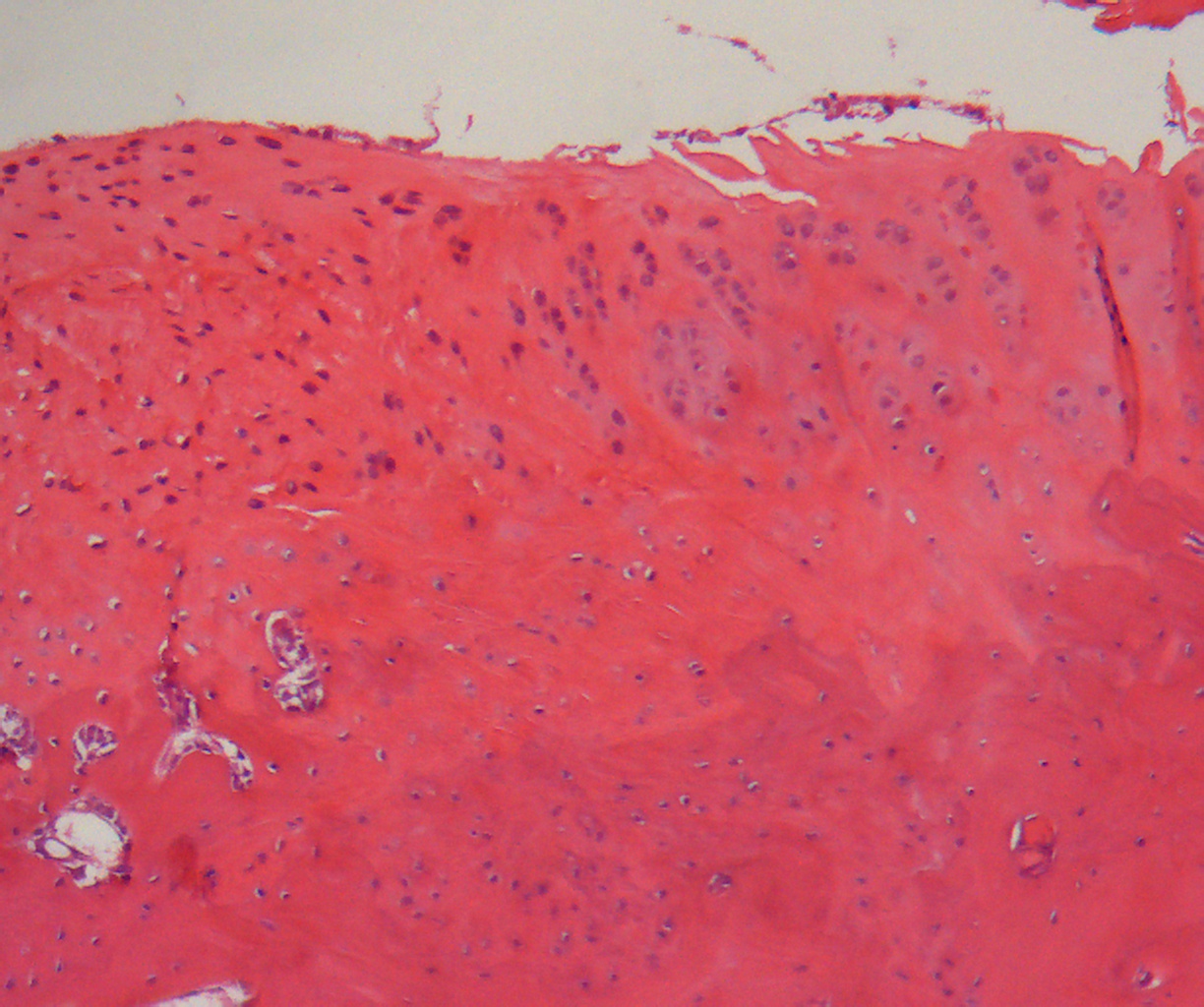

Supplement: Supplementary file 2 [file DataSheet_1.zip › HE staining/Model/5.jpg]

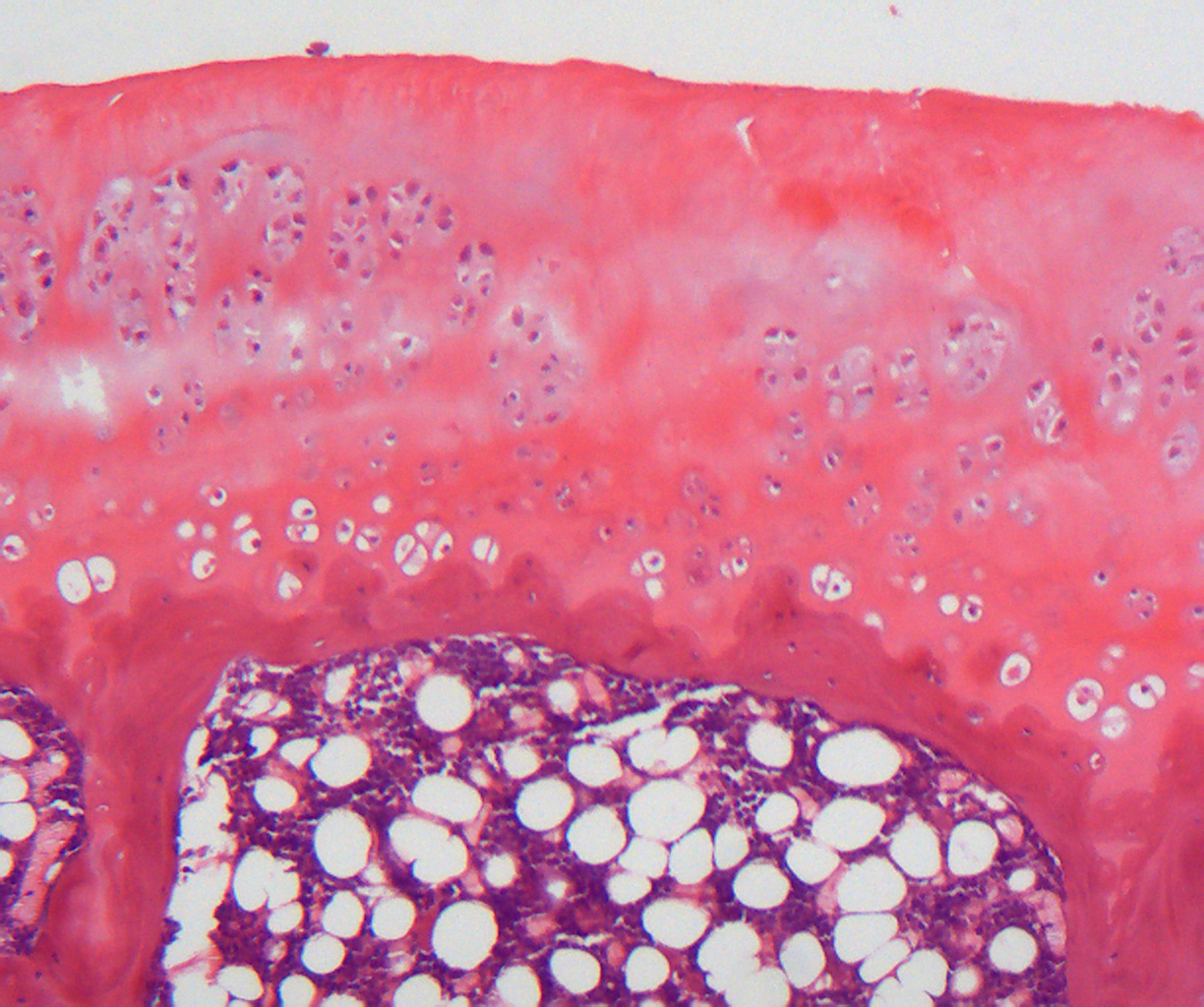

Supplement: Supplementary file 2 [file DataSheet_1.zip › HE staining/Model/6.jpg]

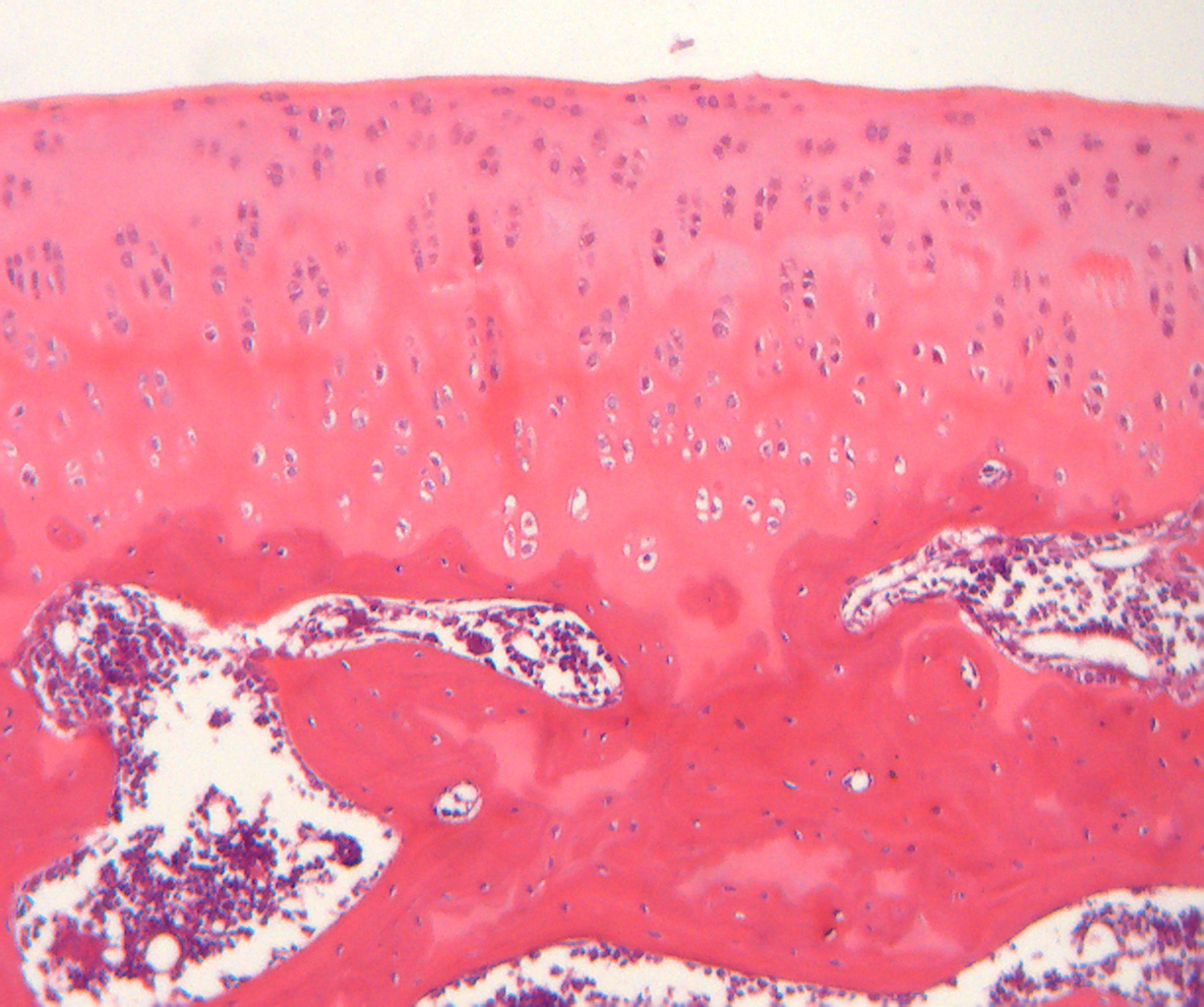

Supplement: Supplementary file 2 [file DataSheet_1.zip › HE staining/Sham/1.jpg]

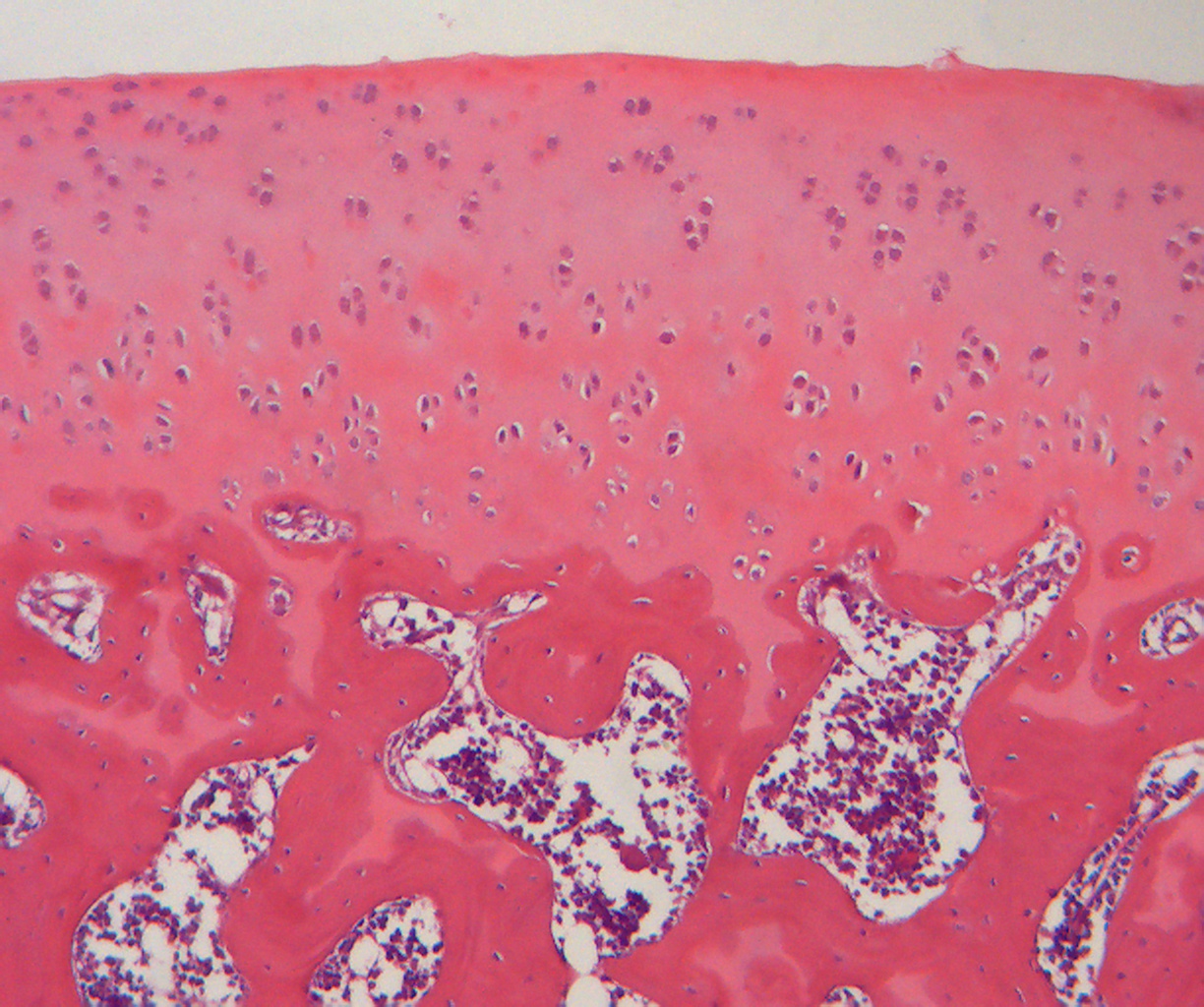

Supplement: Supplementary file 2 [file DataSheet_1.zip › HE staining/Sham/2.jpg]

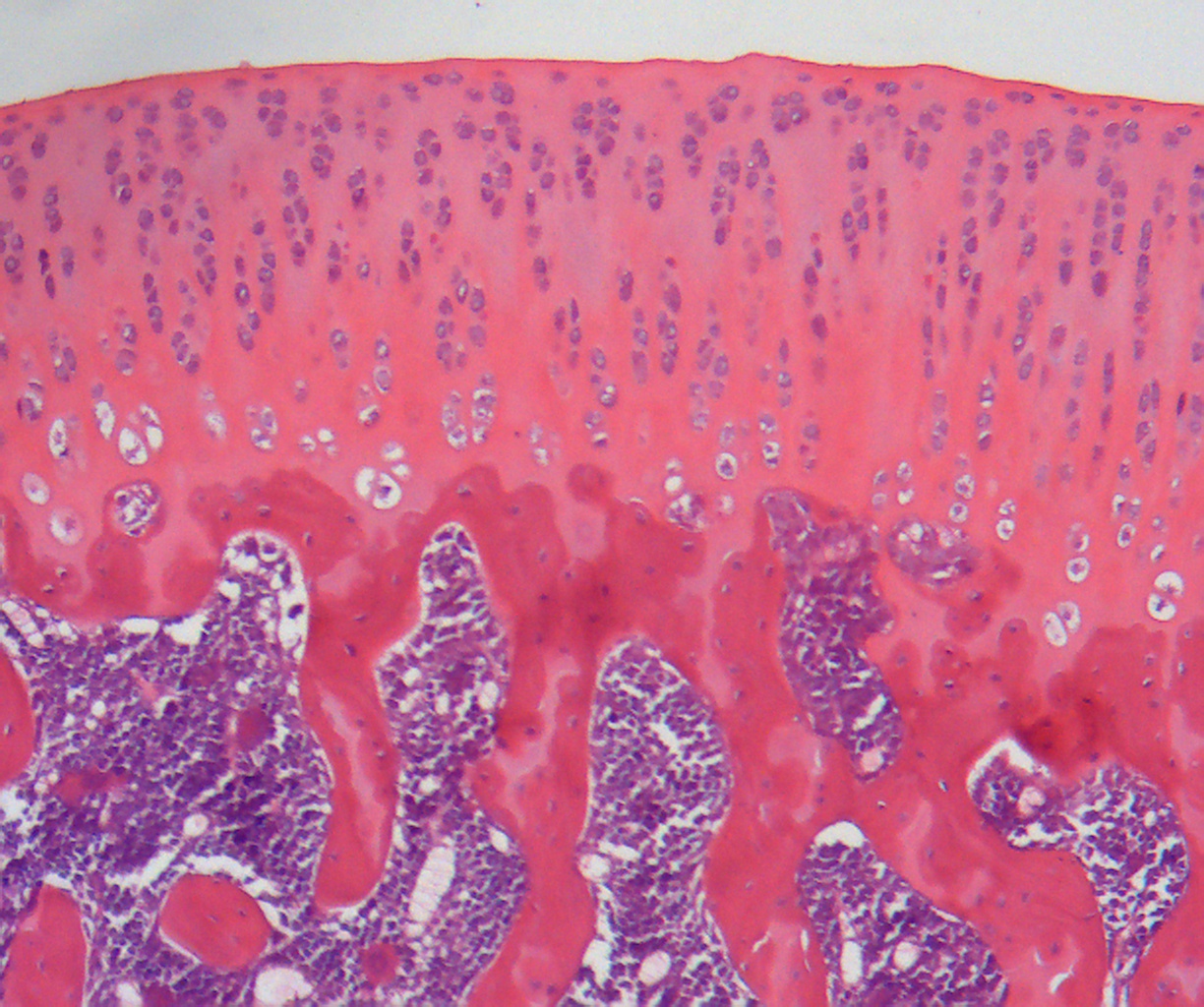

Supplement: Supplementary file 2 [file DataSheet_1.zip › HE staining/Sham/3.jpg]

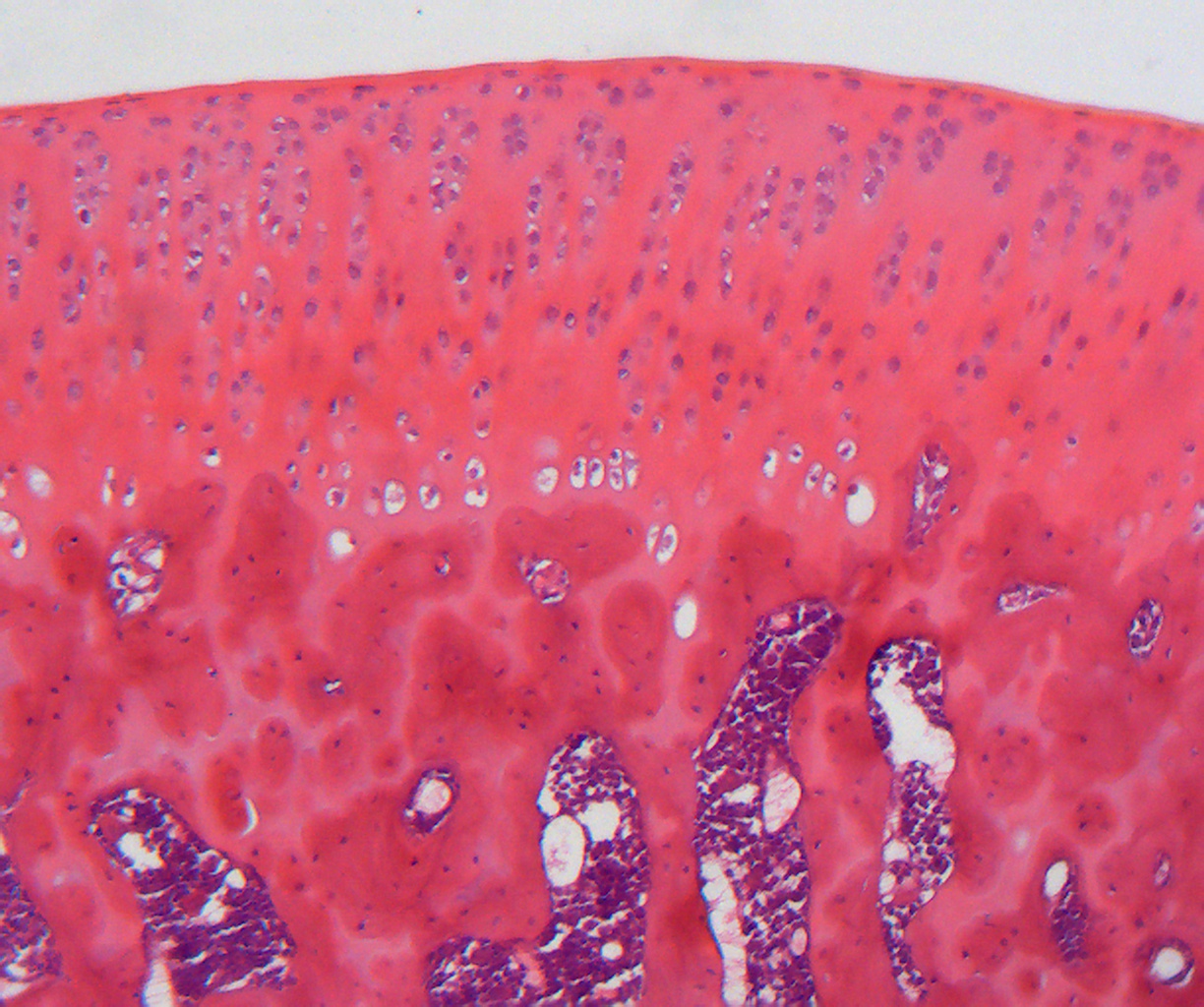

Supplement: Supplementary file 2 [file DataSheet_1.zip › HE staining/Sham/4.jpg]

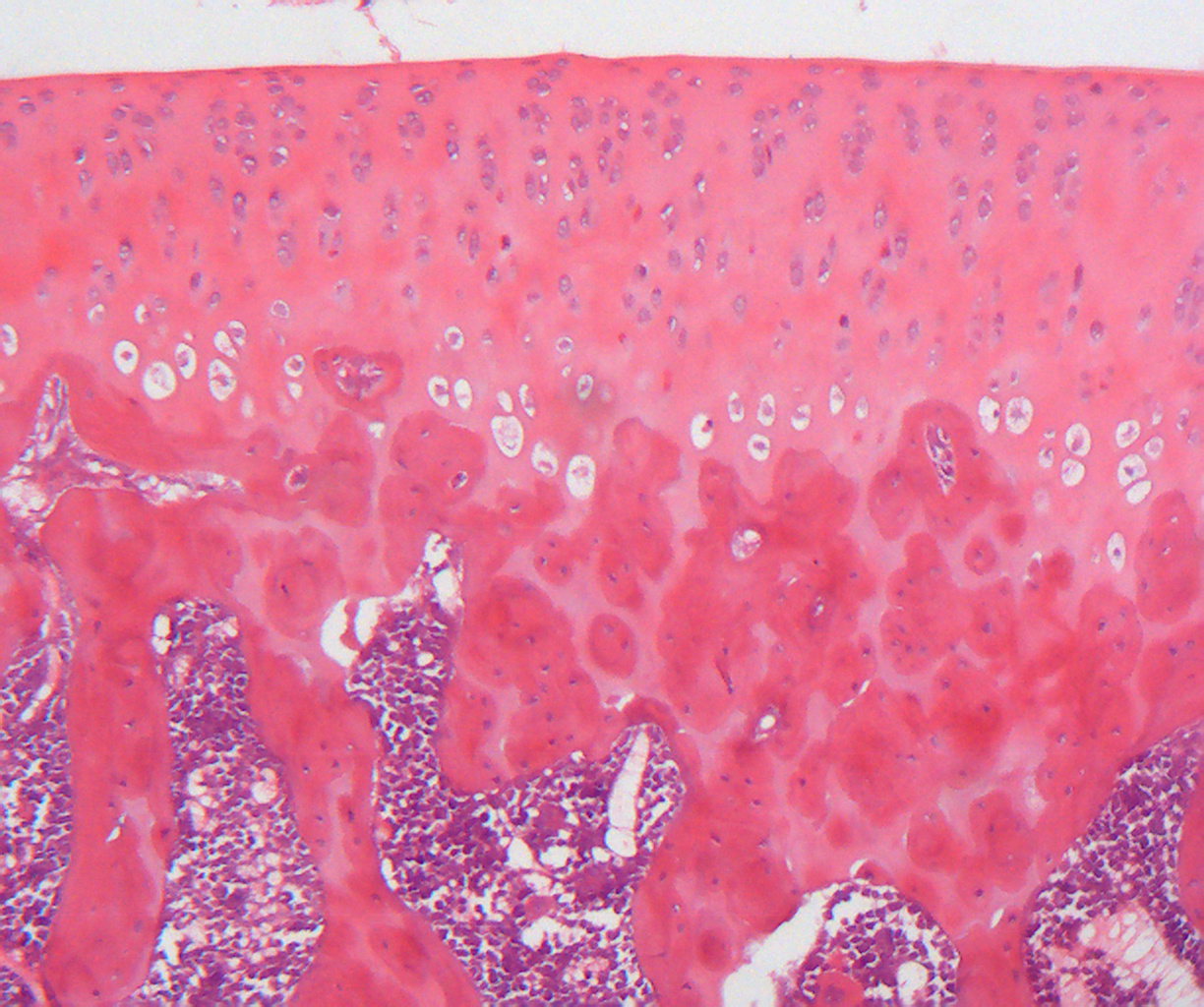

Supplement: Supplementary file 2 [file DataSheet_1.zip › HE staining/Sham/5.jpg]

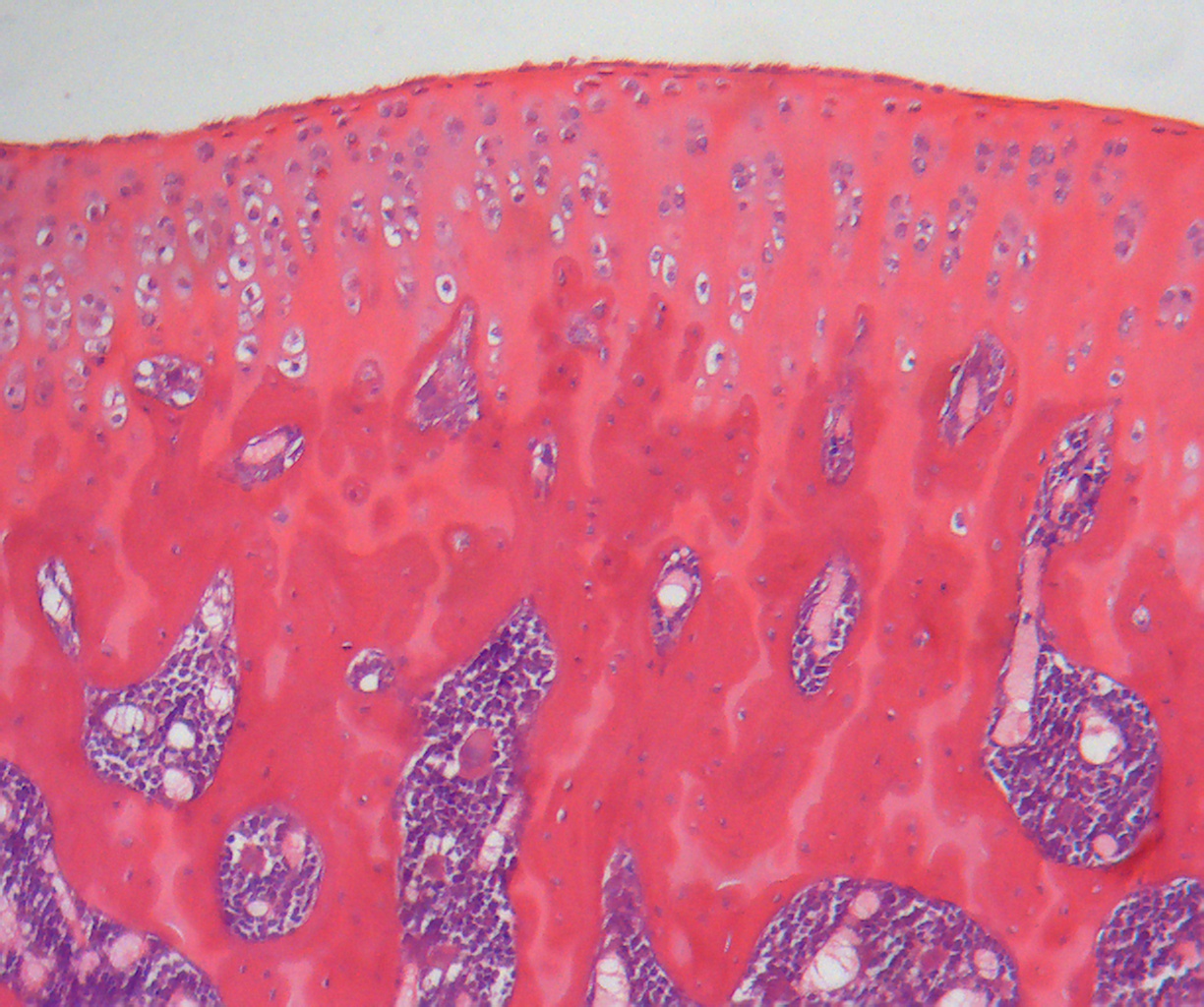

Supplement: Supplementary file 2 [file DataSheet_1.zip › HE staining/Sham/6.jpg]

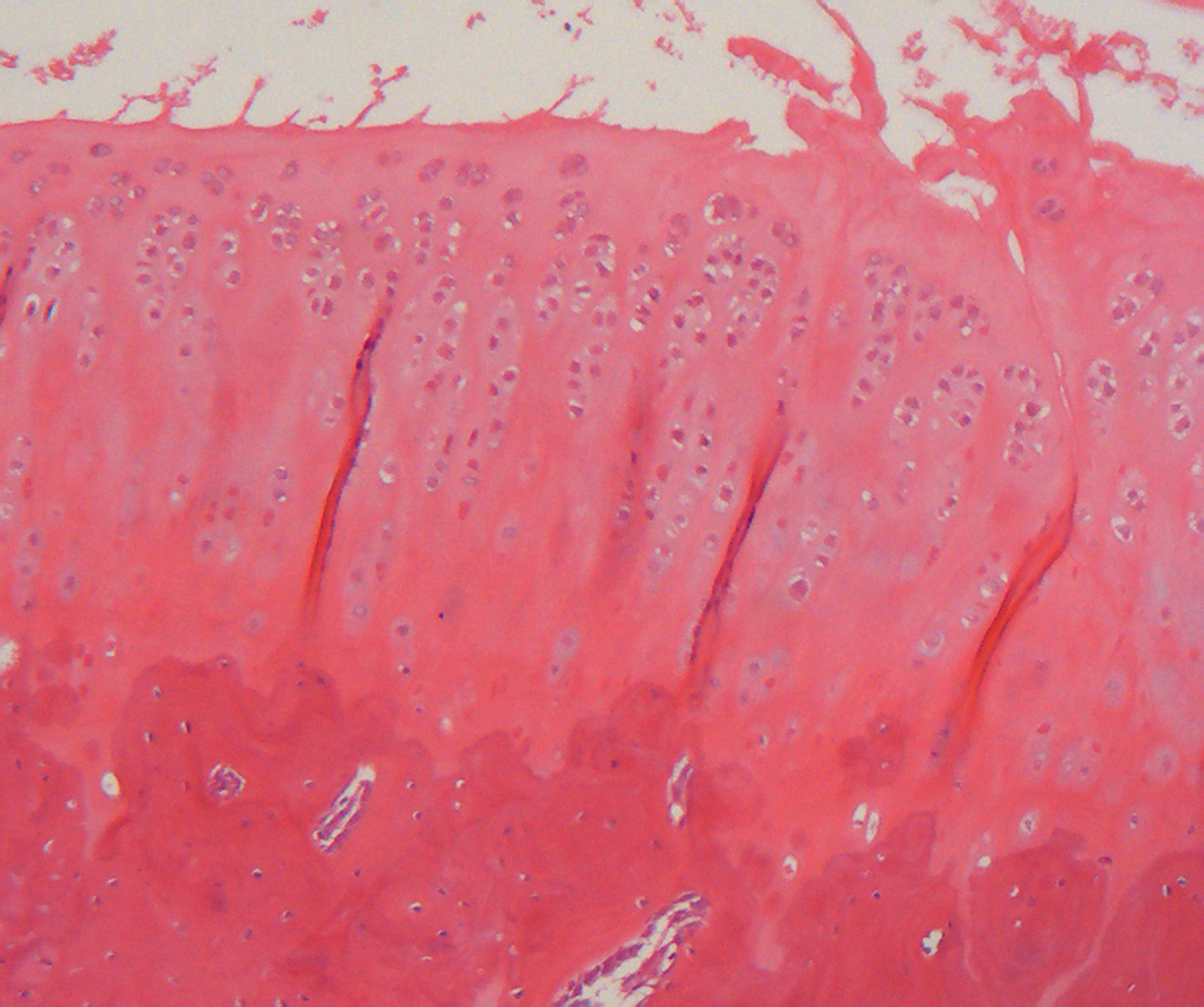

Supplement: Supplementary file 2 [file DataSheet_1.zip › HE staining/miR(-) Exo/1.jpg]

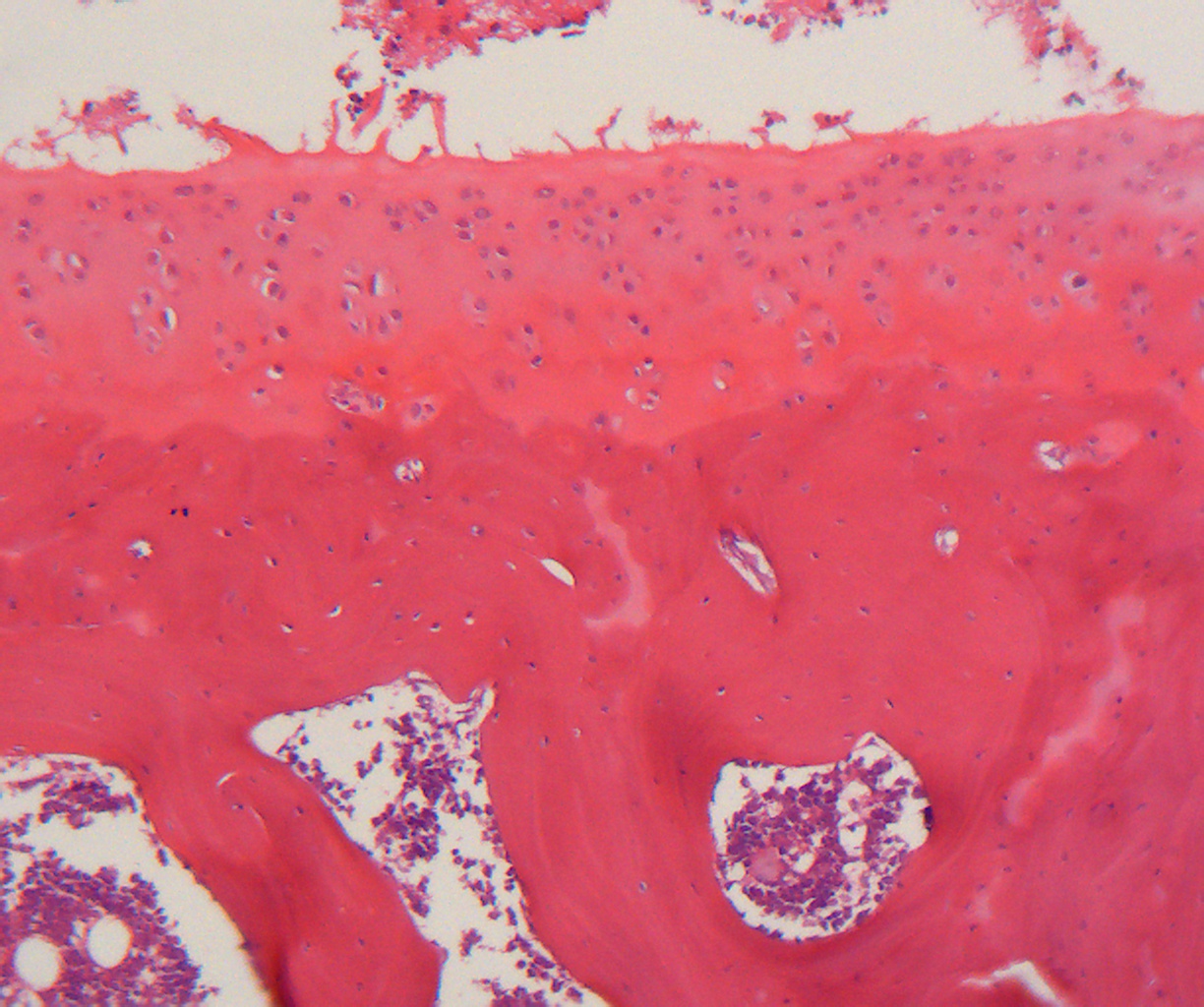

Supplement: Supplementary file 2 [file DataSheet_1.zip › HE staining/miR(-) Exo/2.jpg]

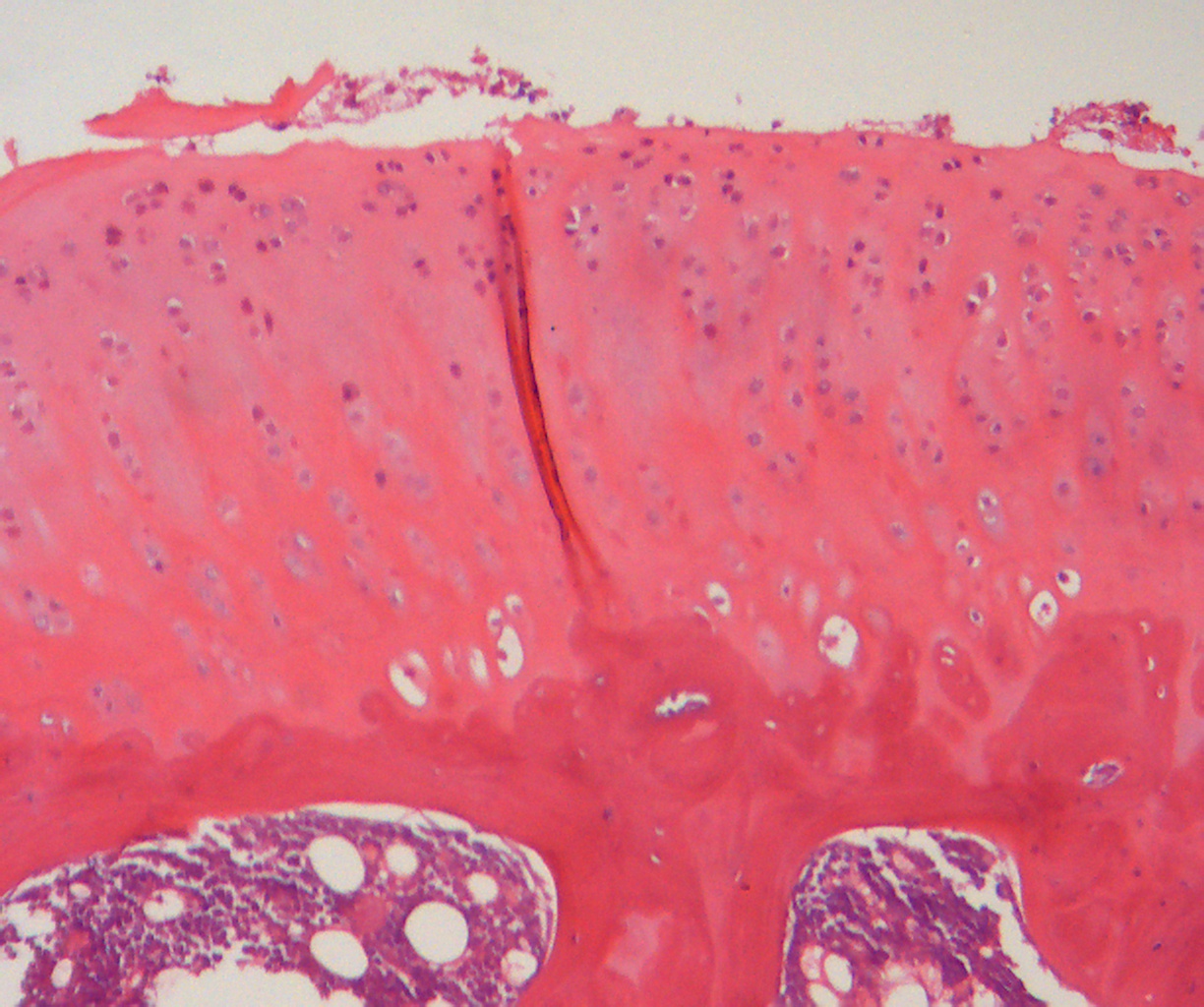

Supplement: Supplementary file 2 [file DataSheet_1.zip › HE staining/miR(-) Exo/3.jpg]

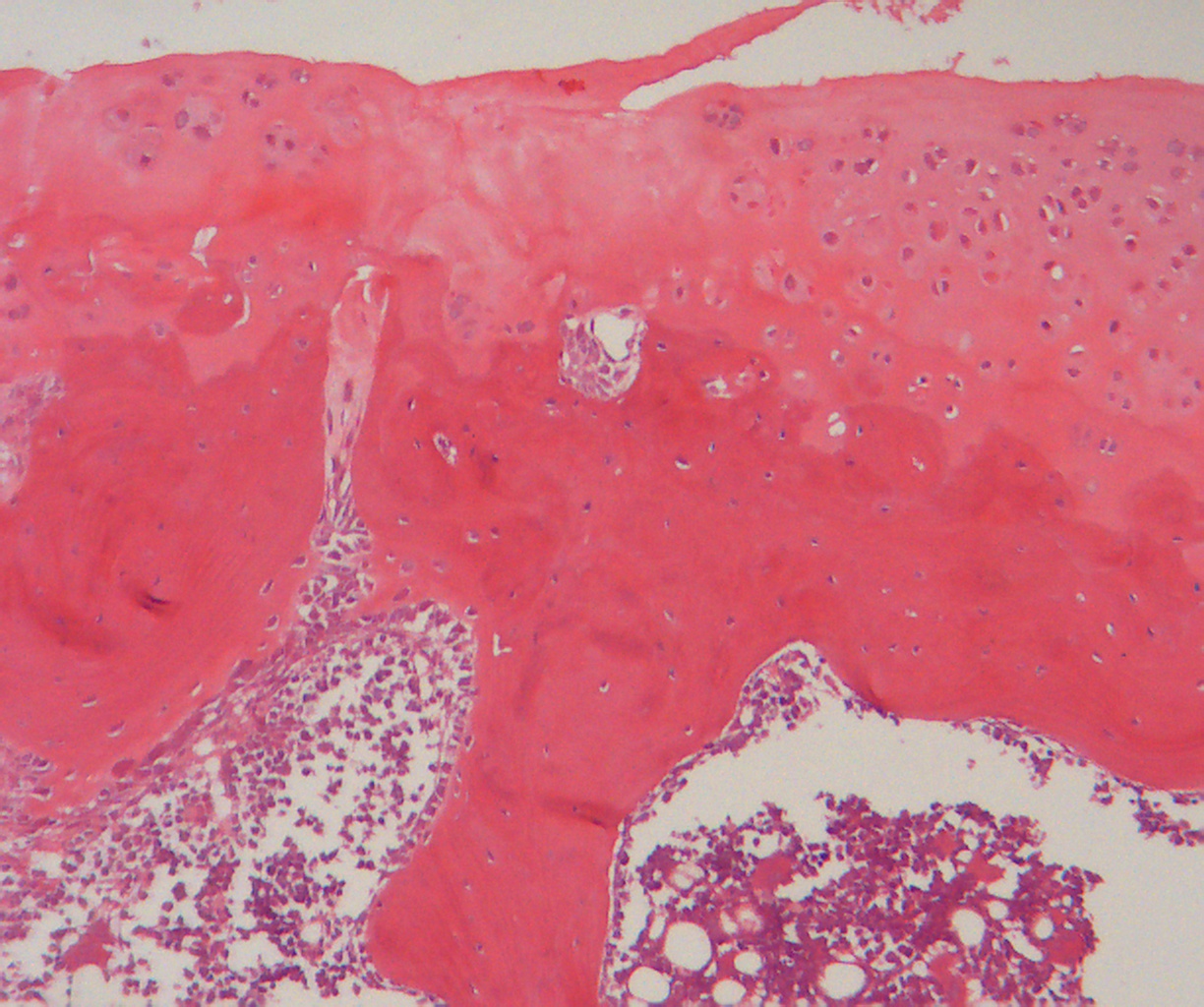

Supplement: Supplementary file 2 [file DataSheet_1.zip › HE staining/miR(-) Exo/4.jpg]

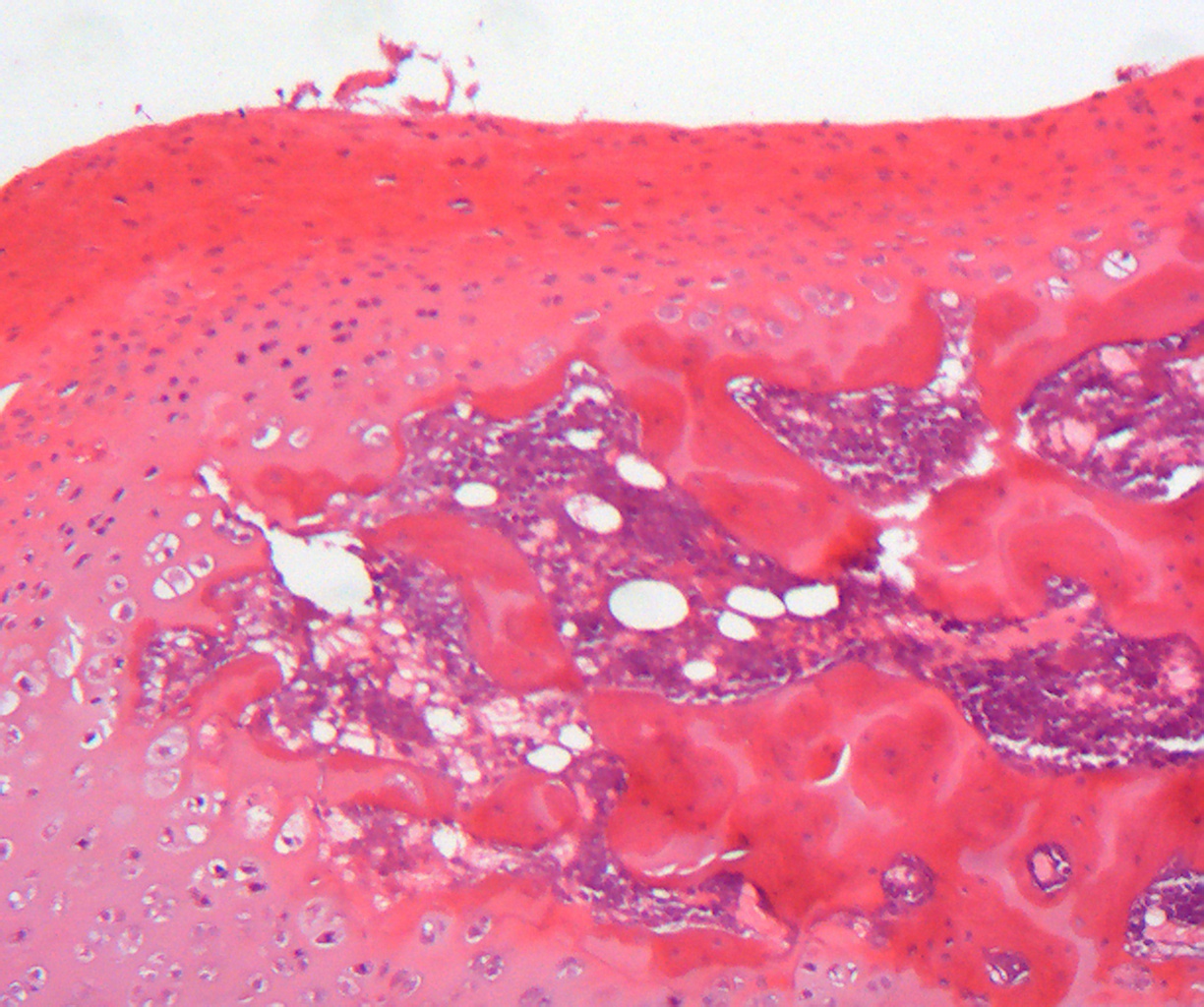

Supplement: Supplementary file 2 [file DataSheet_1.zip › HE staining/miR(-) Exo/5.jpg]

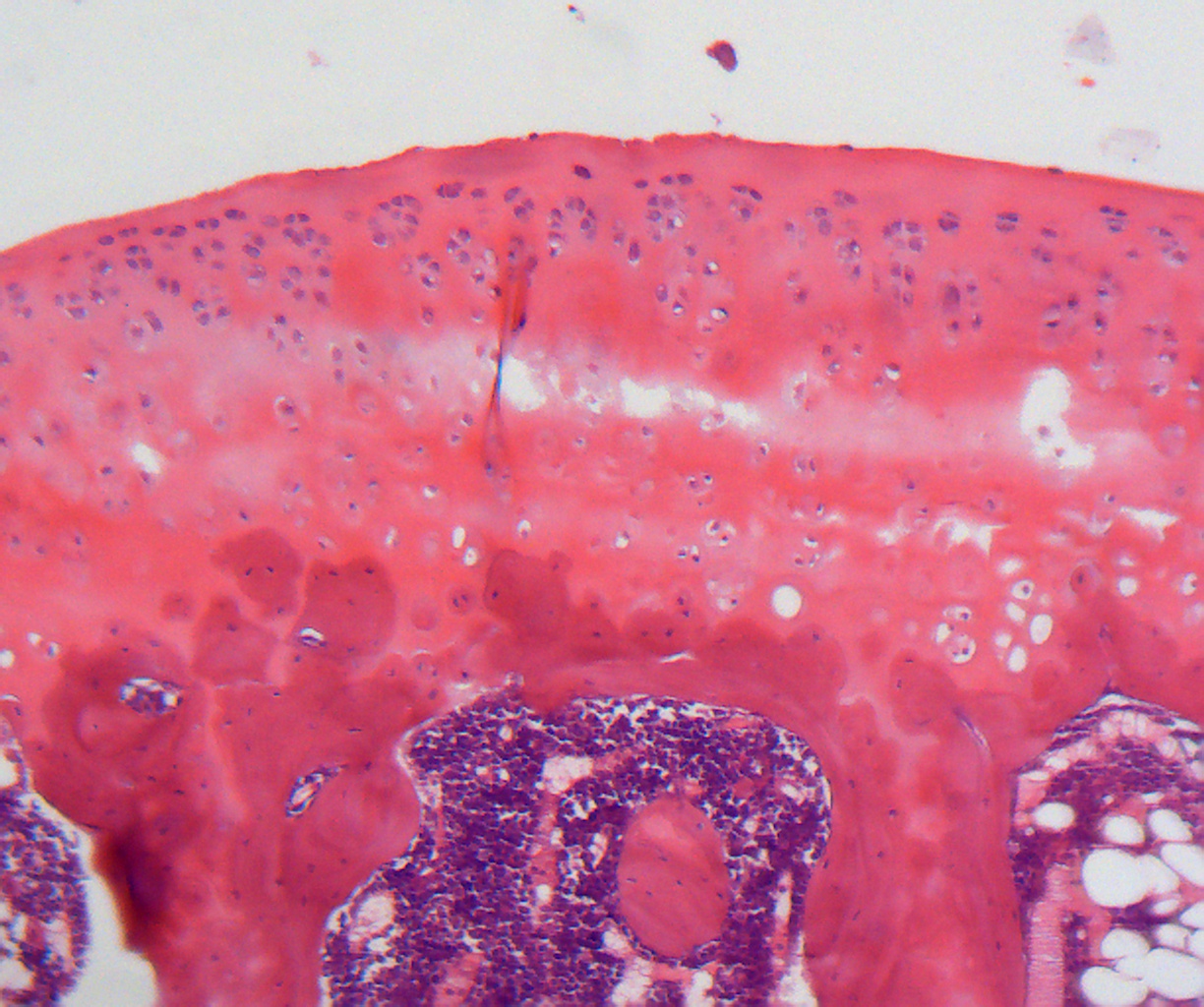

Supplement: Supplementary file 2 [file DataSheet_1.zip › HE staining/miR(-) Exo/6.jpg]

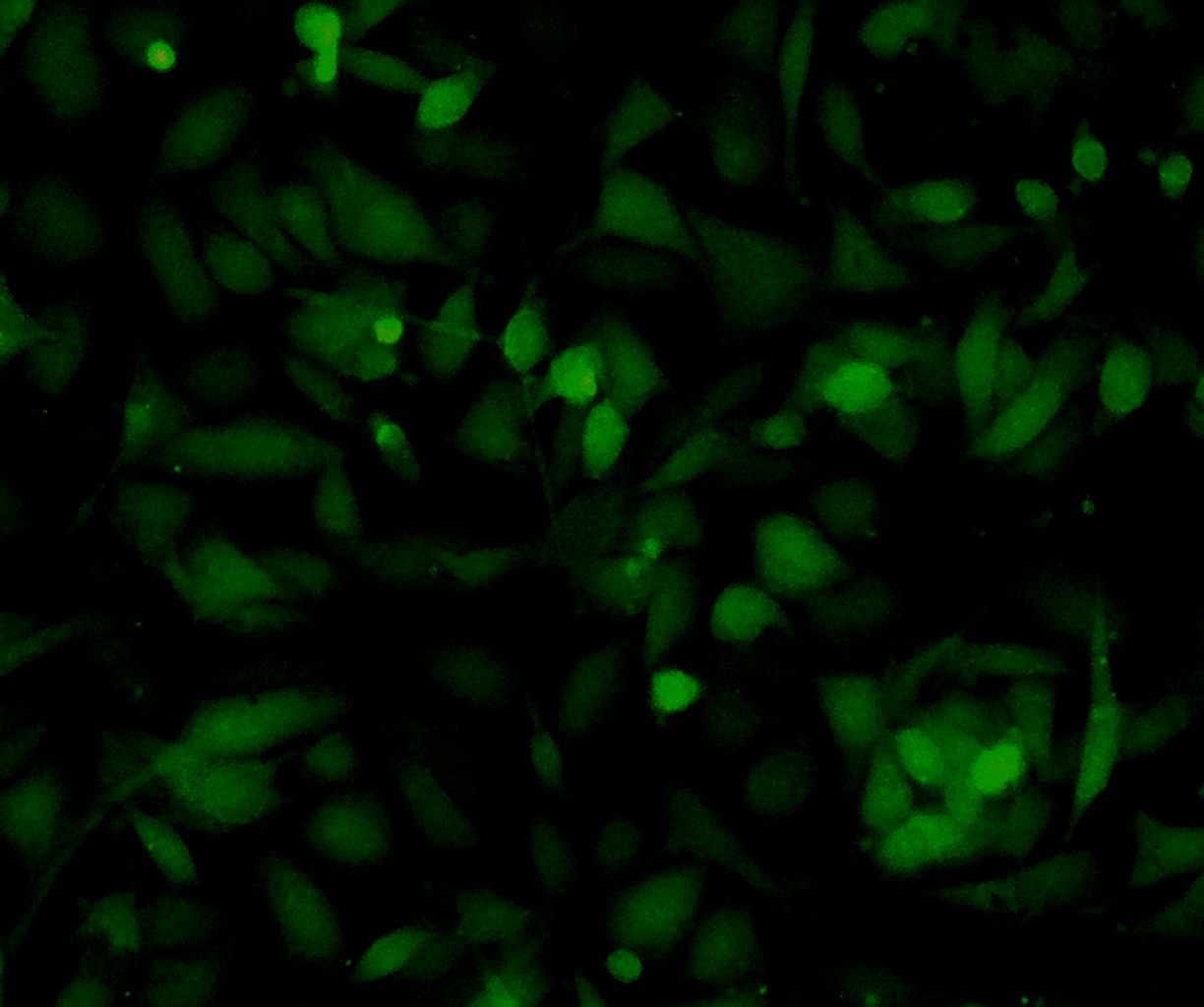

Supplement: Supplementary file 3 [file DataSheet_2.zip › ROS/Fig2/IL-1a┬/1.jpg]

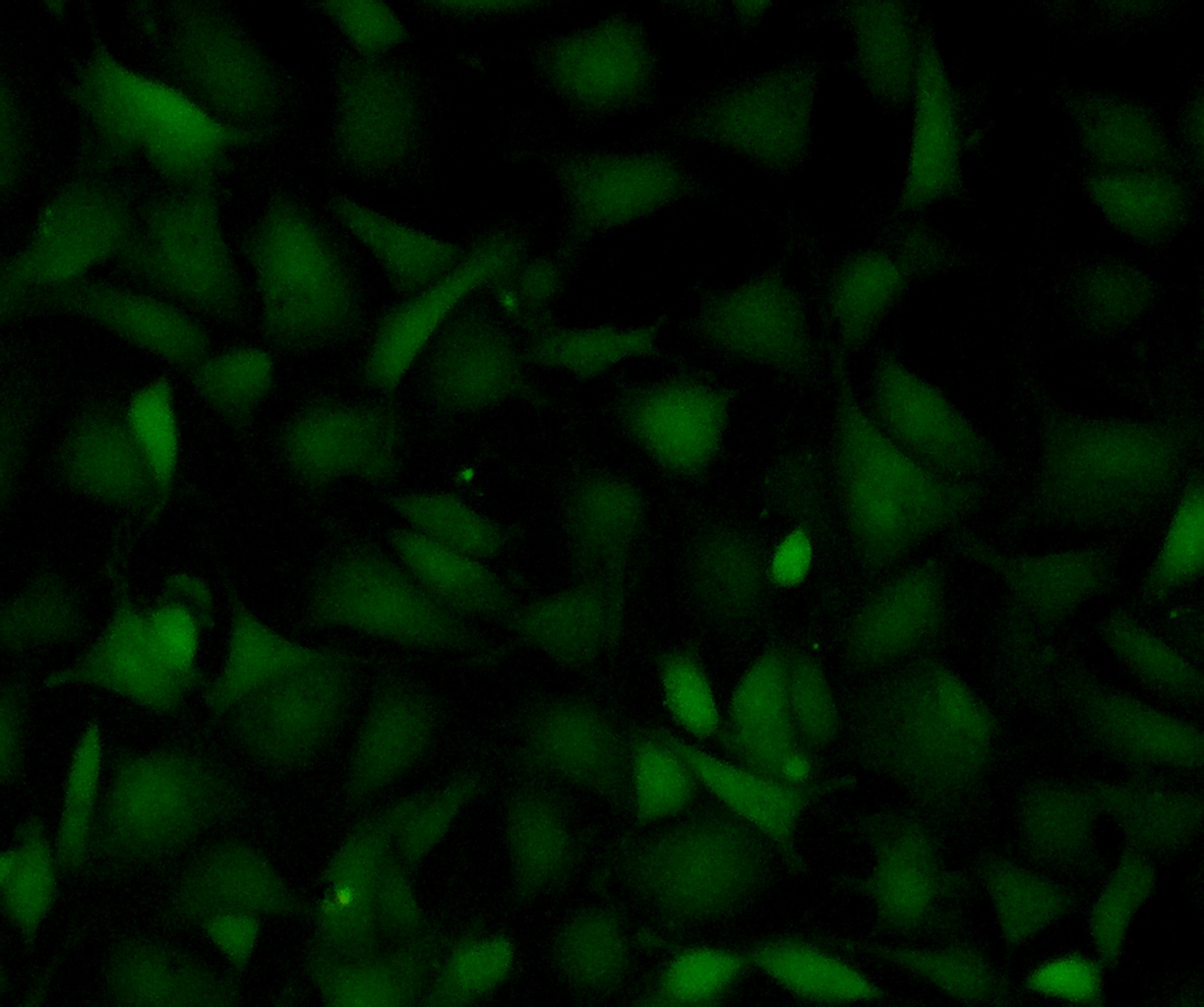

Supplement: Supplementary file 3 [file DataSheet_2.zip › ROS/Fig2/IL-1a┬/2.jpg]

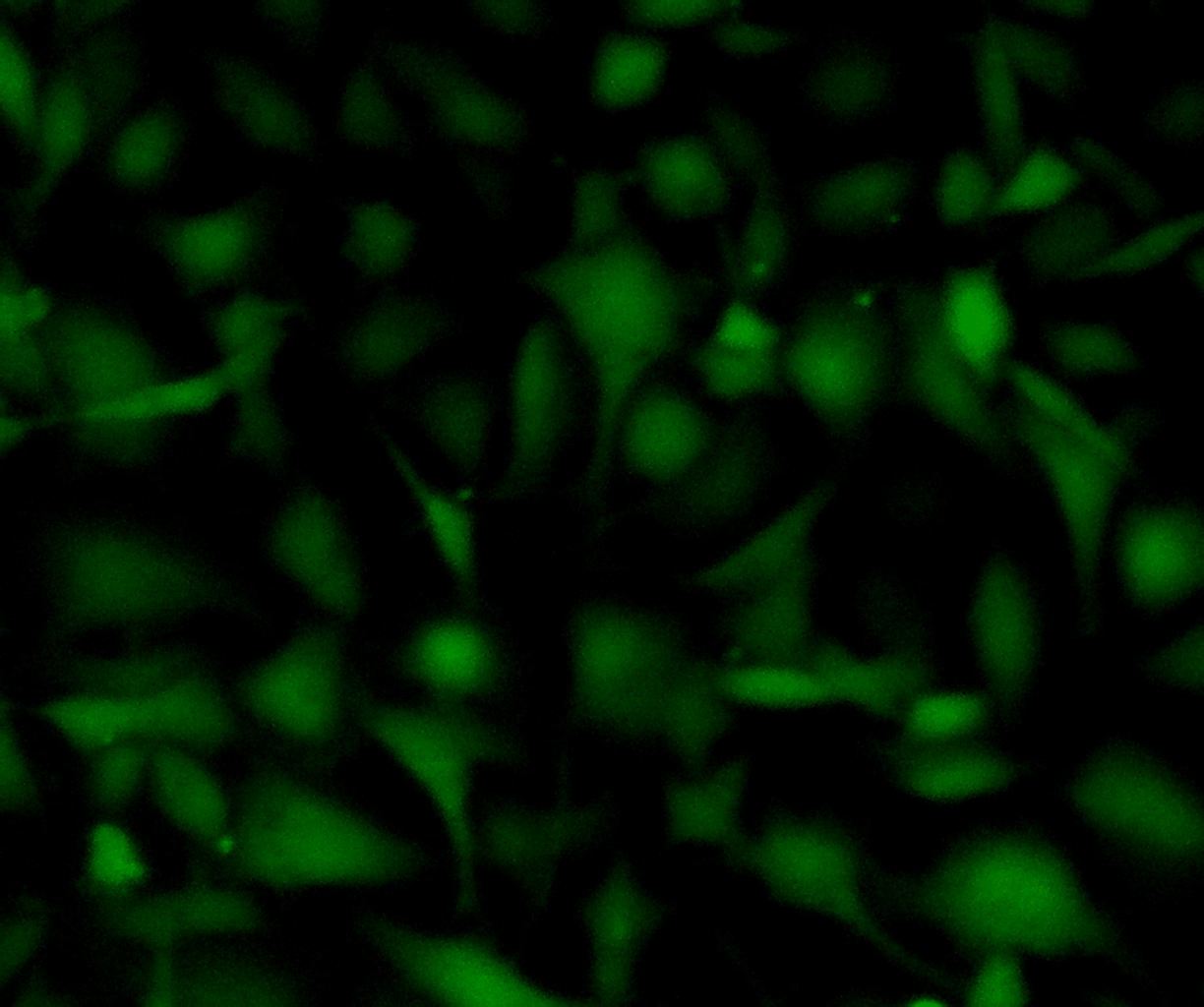

Supplement: Supplementary file 3 [file DataSheet_2.zip › ROS/Fig2/IL-1a┬/3.jpg]
