# Supplementary figures and images for: Exosomes from osteoarthritic fibroblast-like synoviocytes promote cartilage ferroptosis and damage via delivering microRNA-19b-3p to target SLC7A11 in osteoarthritis (part 2 of 6)
Source: Front Immunol. 2023 Aug 24;14:1181156. doi: 10.3389/fimmu.2023.1181156 (PMC10484587; doi:10.3389/fimmu.2023.1181156)

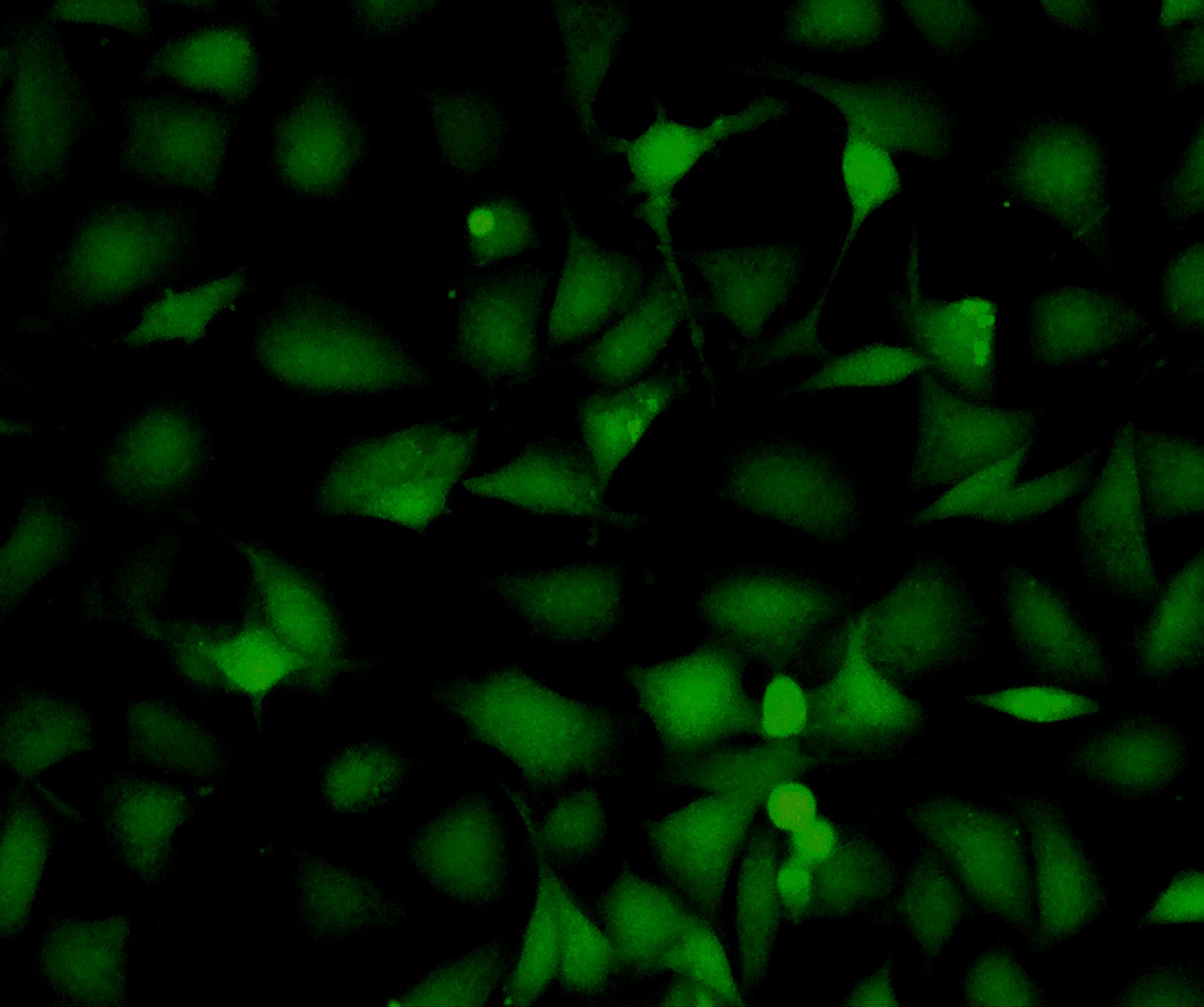

Supplement: Supplementary file 3 [file DataSheet_2.zip › ROS/Fig2/IL-1a┬+Exo/1.jpg]

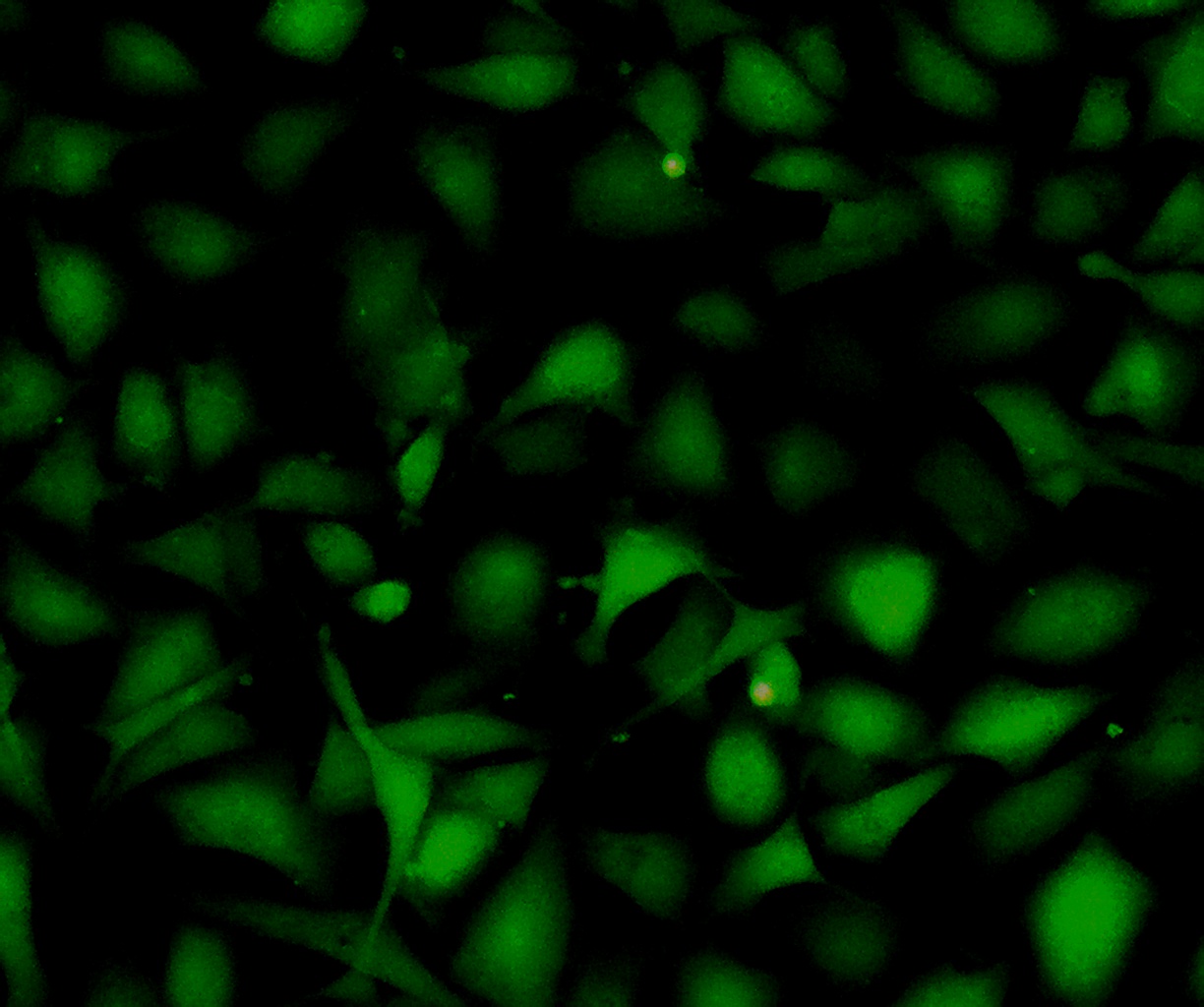

Supplement: Supplementary file 3 [file DataSheet_2.zip › ROS/Fig2/IL-1a┬+Exo/2.jpg]

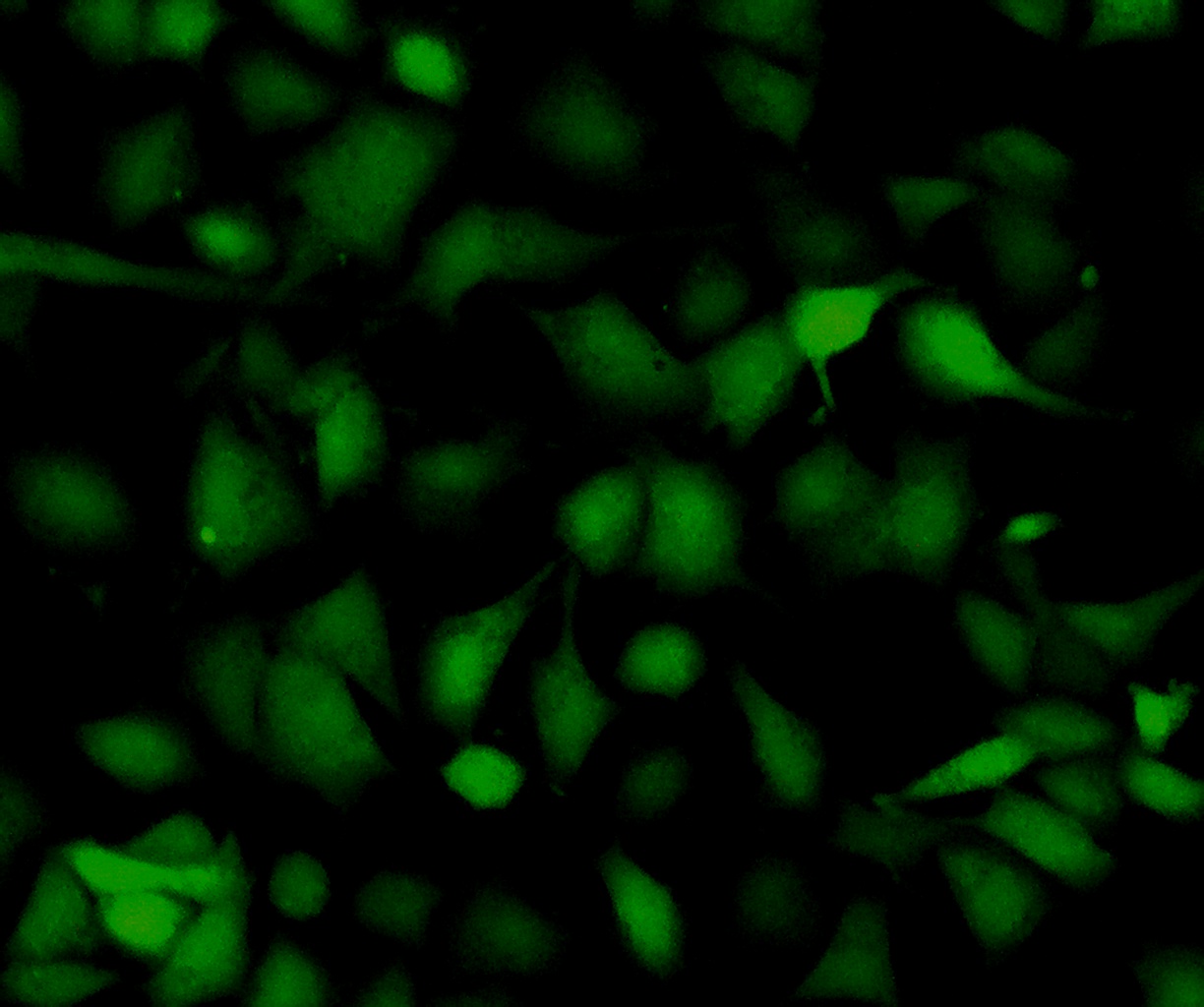

Supplement: Supplementary file 3 [file DataSheet_2.zip › ROS/Fig2/IL-1a┬+Exo/3.jpg]

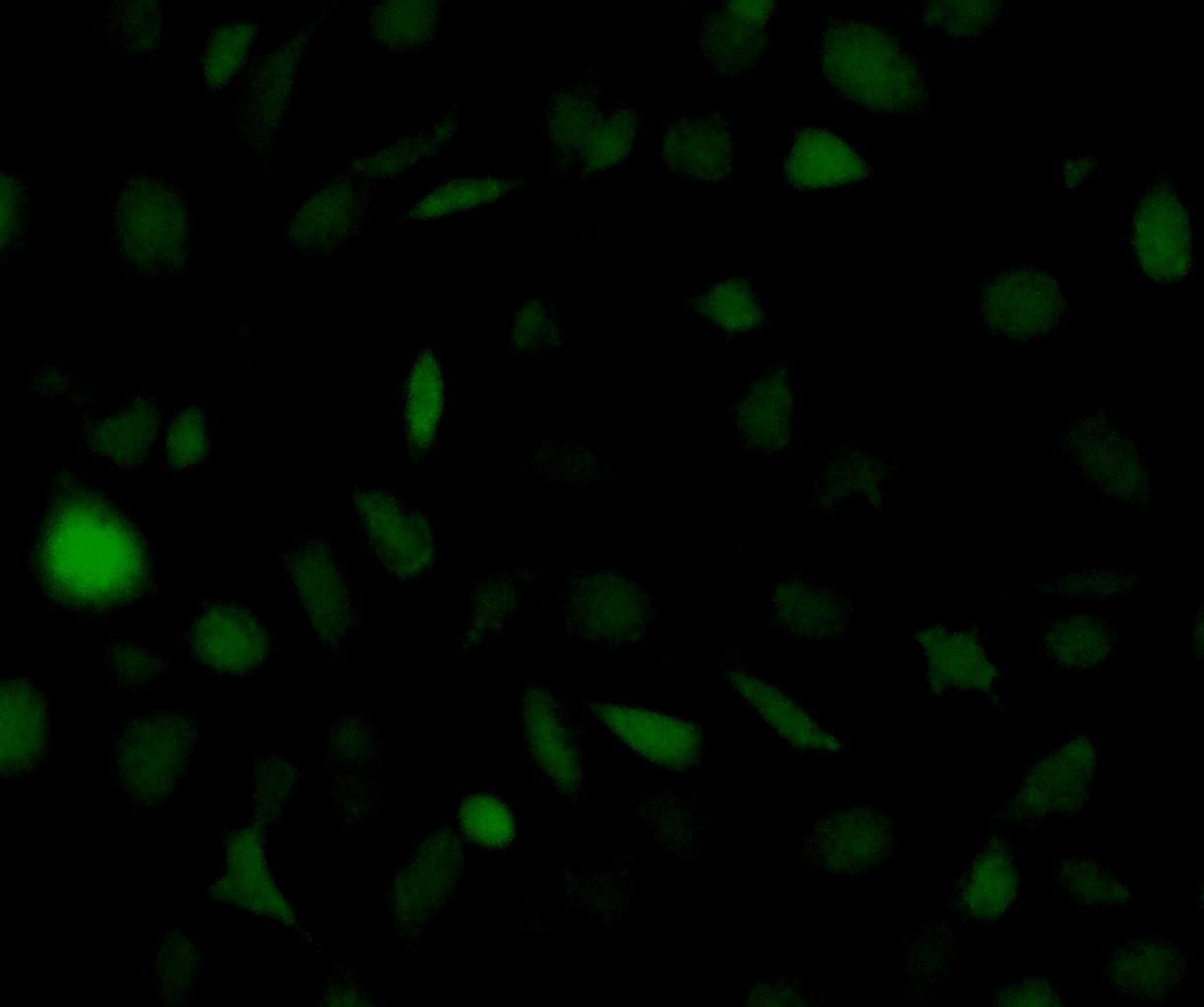

Supplement: Supplementary file 3 [file DataSheet_2.zip › ROS/Fig2/IL-1a┬+Exo+Fer-1/1.jpg]

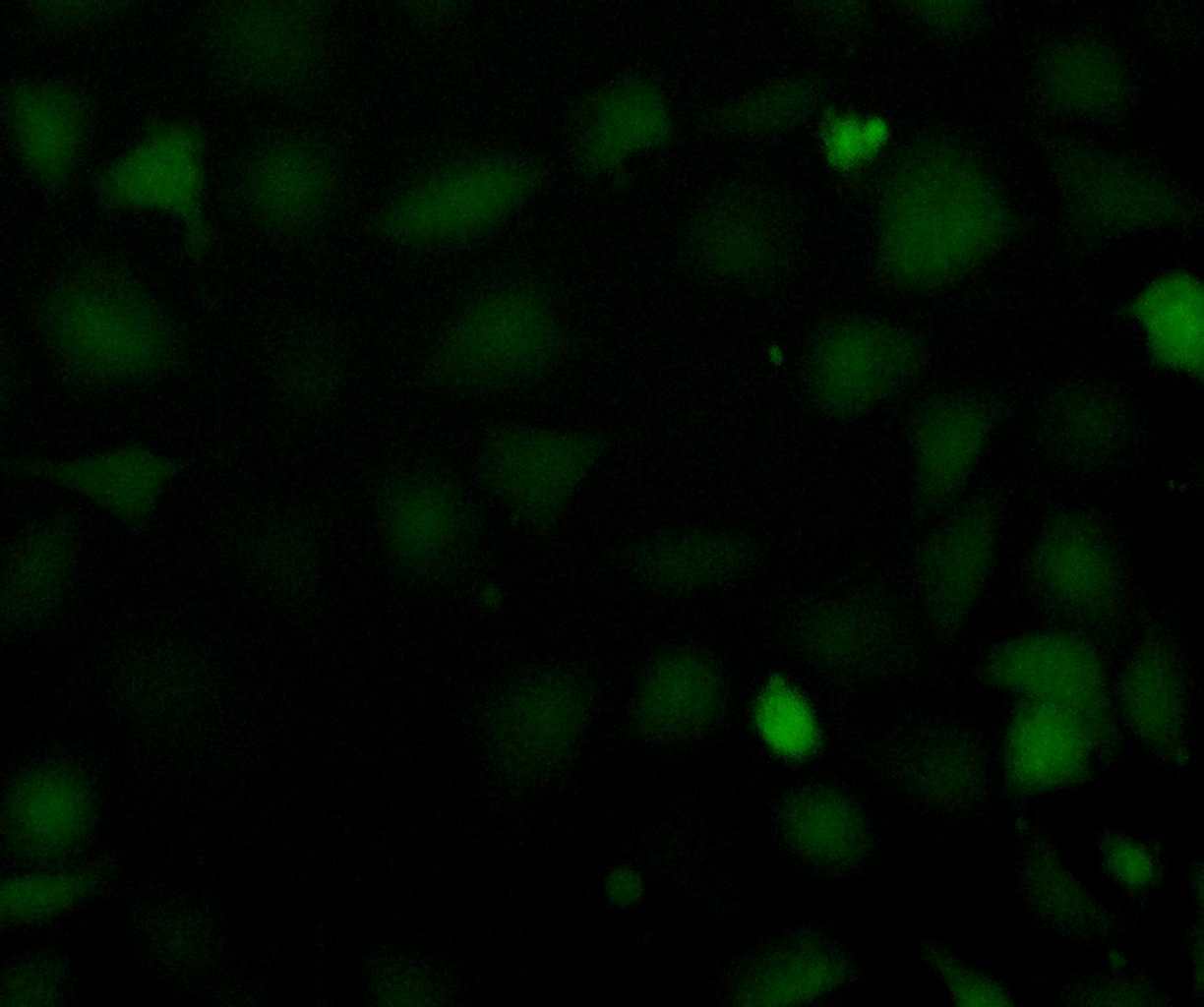

Supplement: Supplementary file 3 [file DataSheet_2.zip › ROS/Fig2/IL-1a┬+Exo+Fer-1/2.jpg]

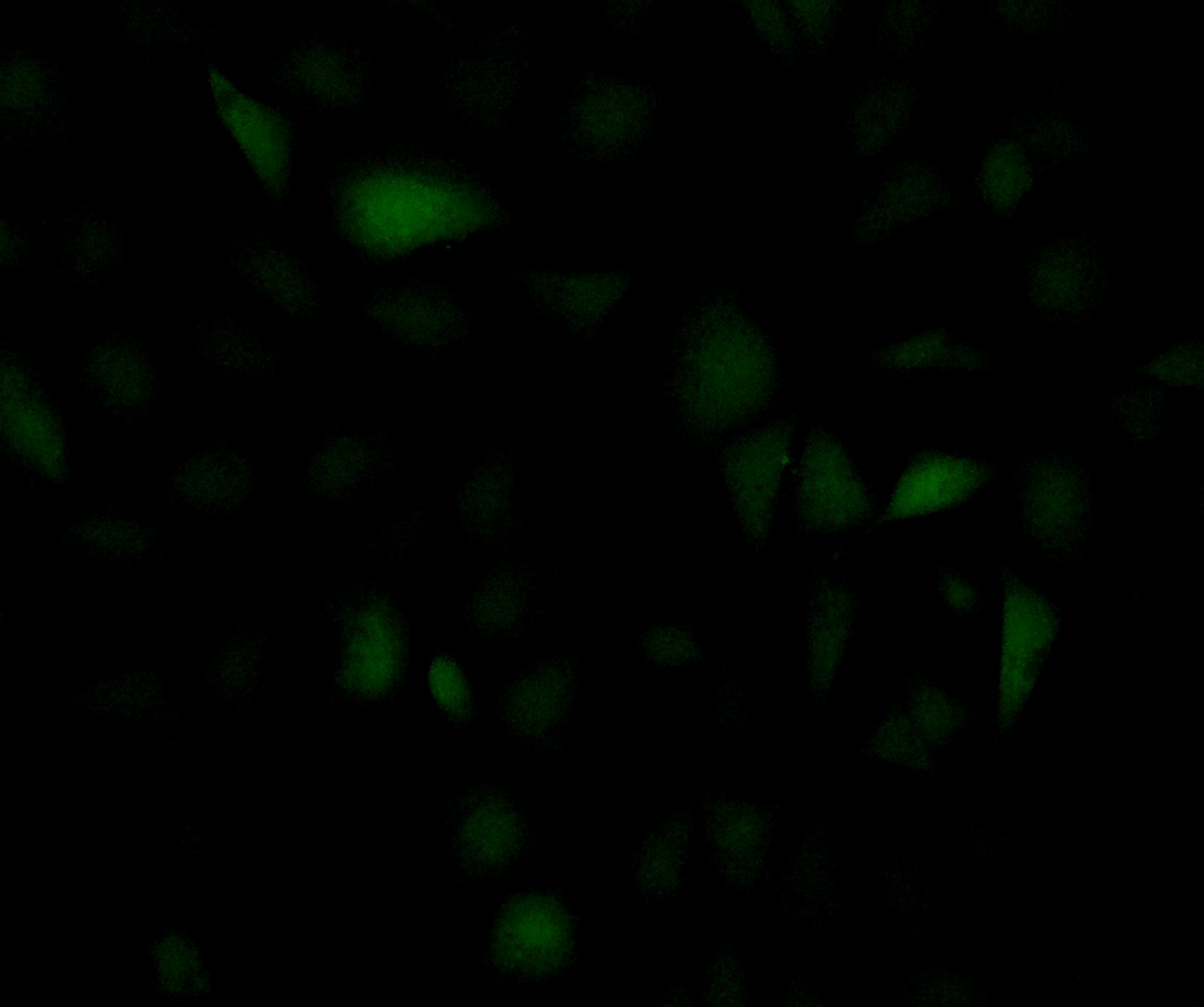

Supplement: Supplementary file 3 [file DataSheet_2.zip › ROS/Fig2/IL-1a┬+Exo+Fer-1/3.jpg]

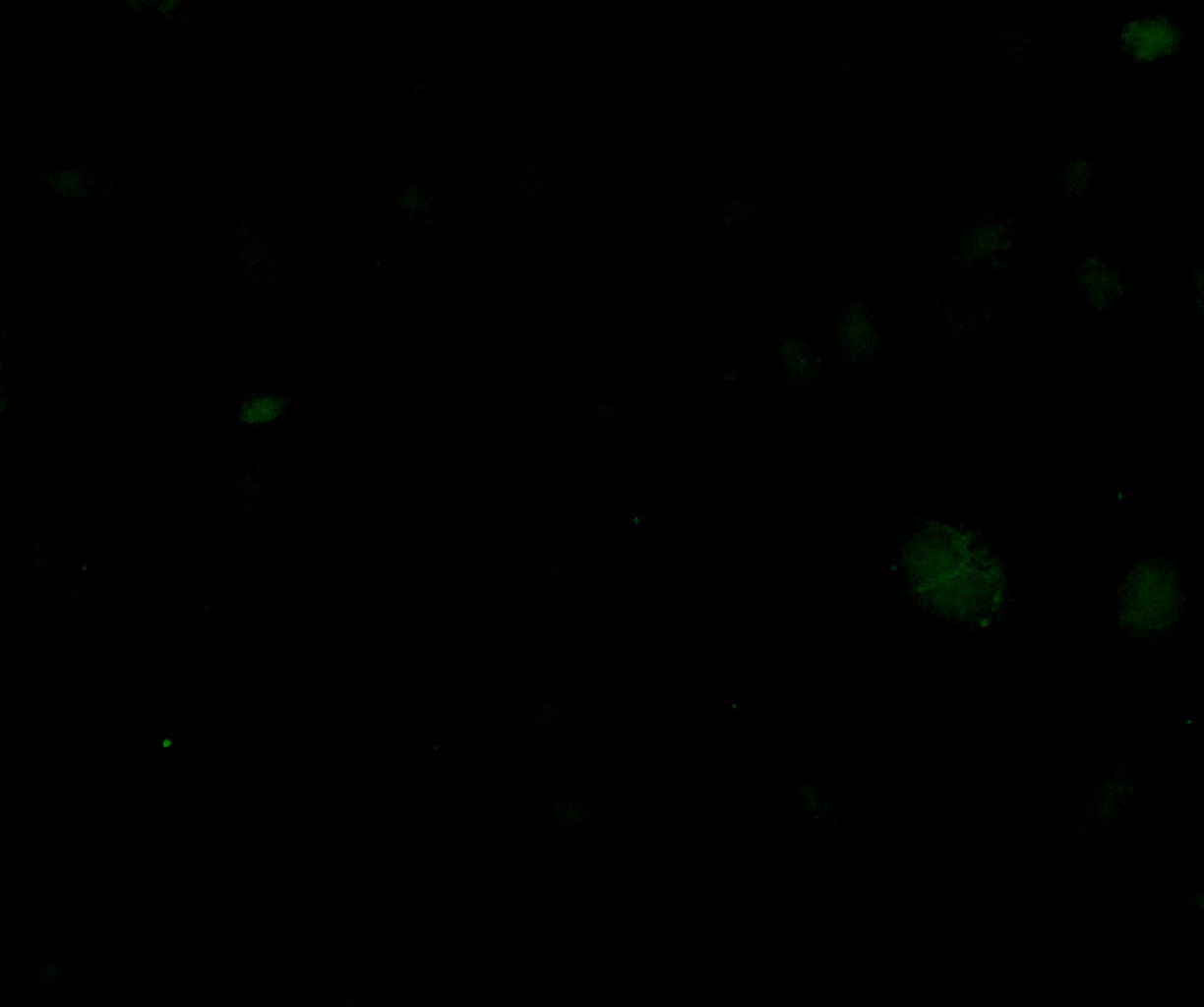

Supplement: Supplementary file 3 [file DataSheet_2.zip › ROS/Fig2/Normal/1.jpg]

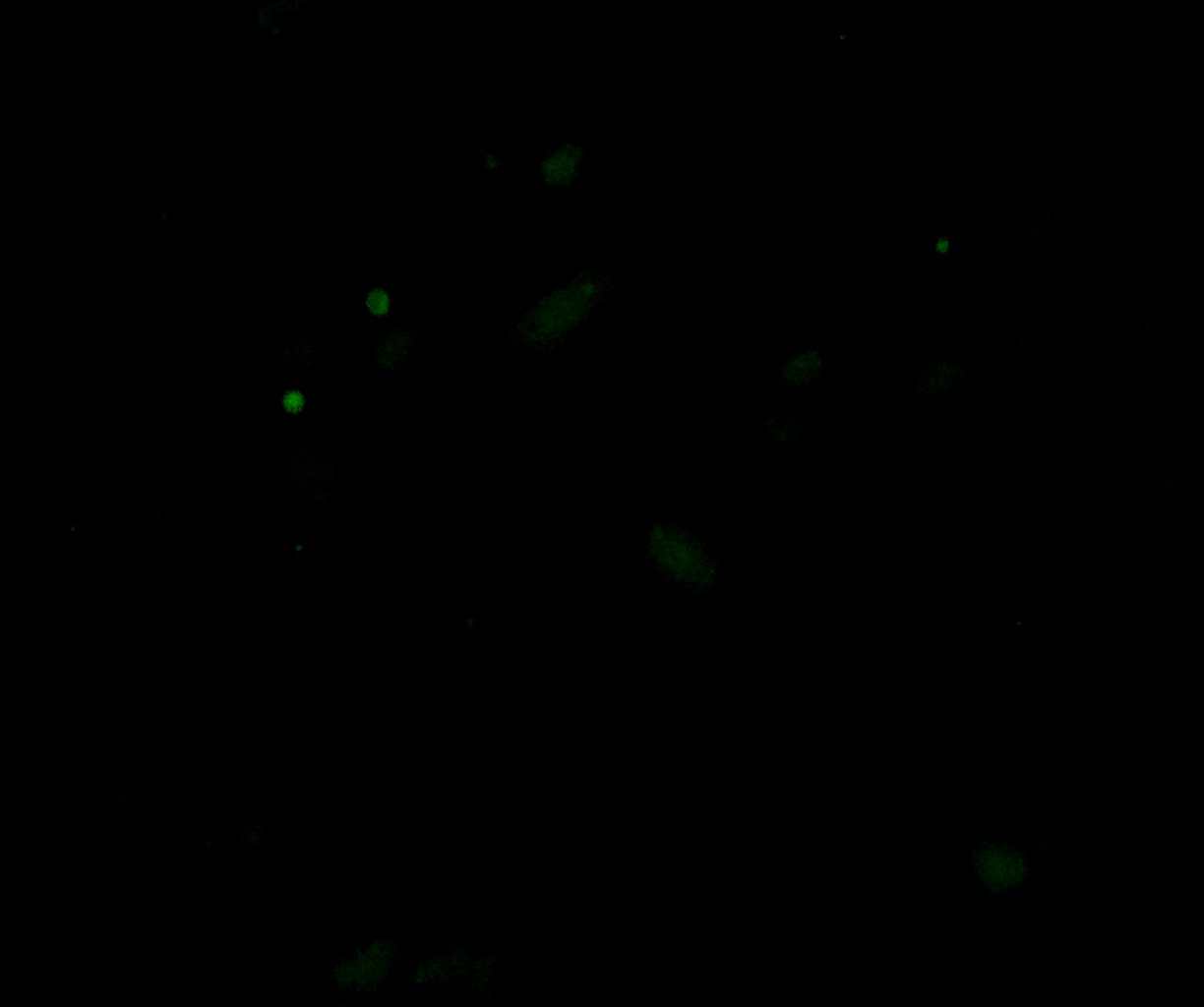

Supplement: Supplementary file 3 [file DataSheet_2.zip › ROS/Fig2/Normal/2.jpg]

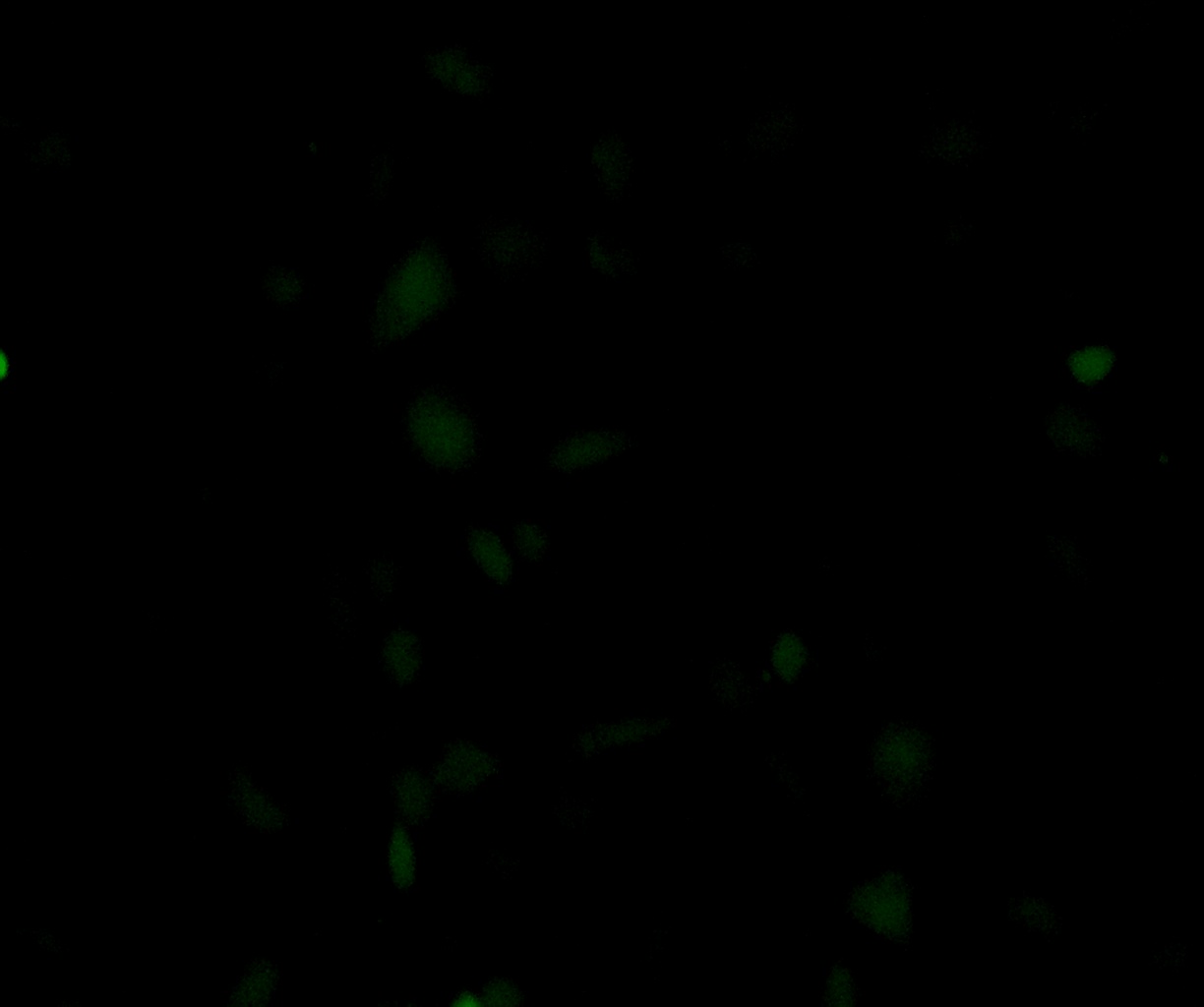

Supplement: Supplementary file 3 [file DataSheet_2.zip › ROS/Fig2/Normal/3.jpg]

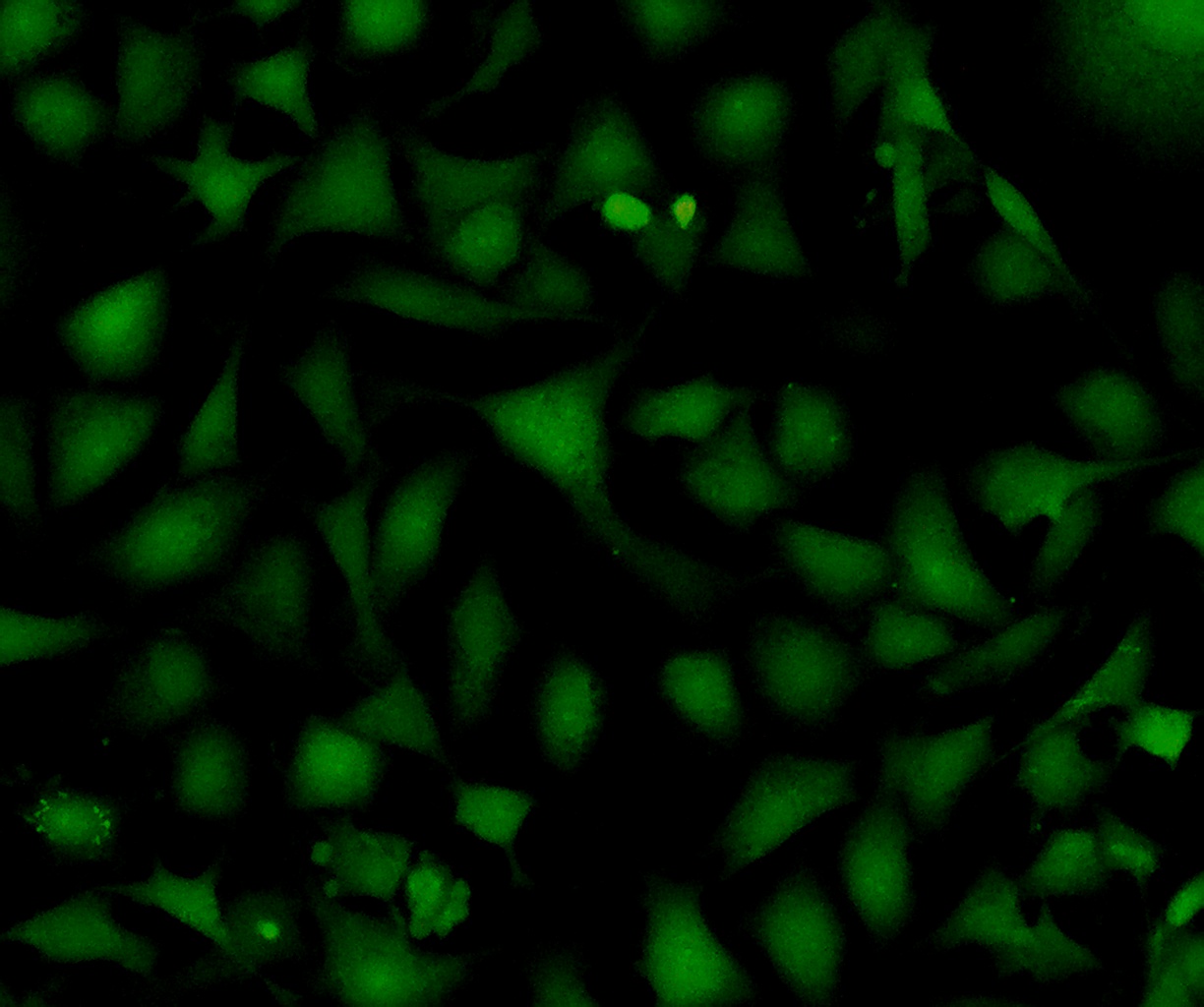

Supplement: Supplementary file 3 [file DataSheet_2.zip › ROS/Fig3/Control Exo/1.jpg]

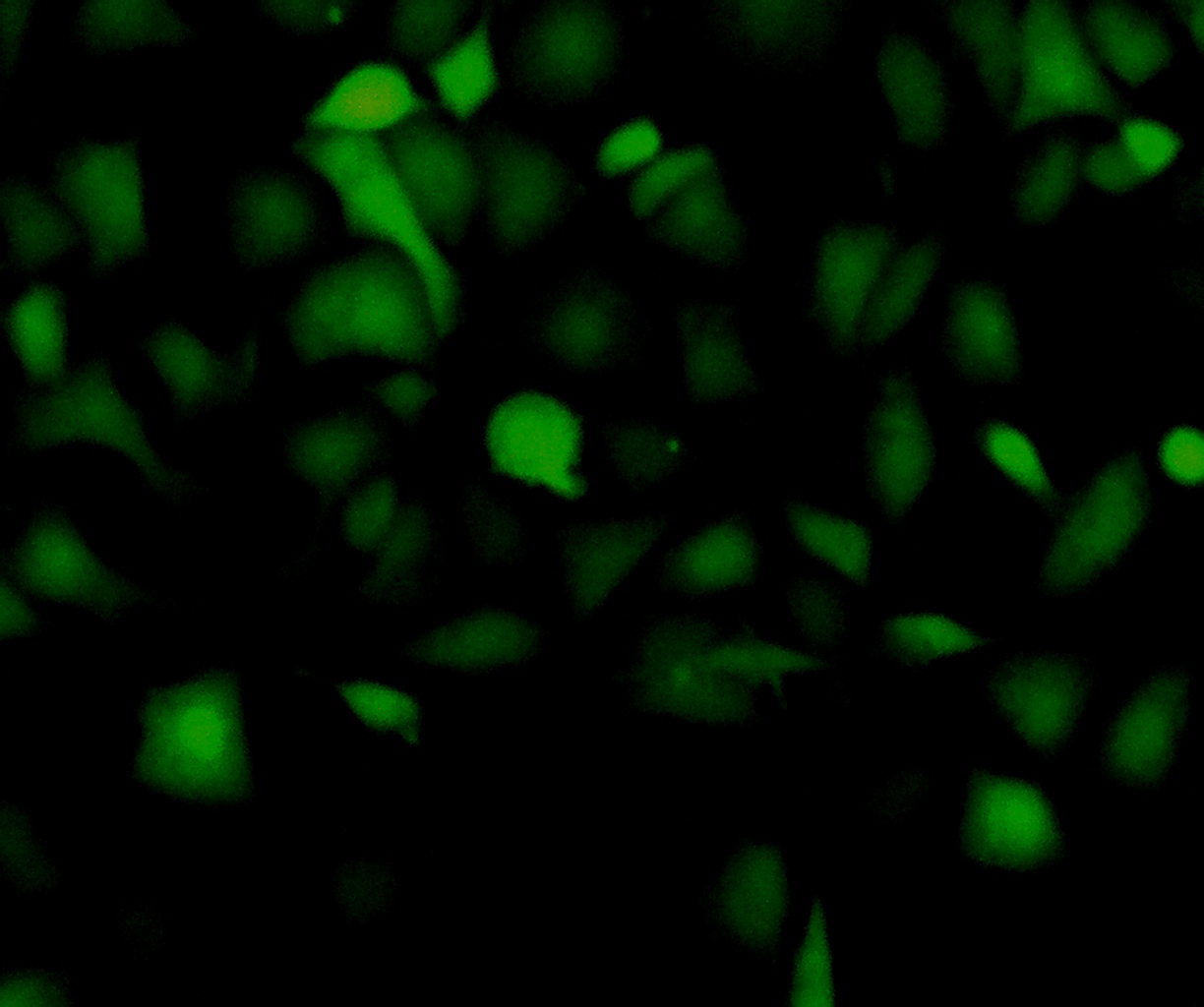

Supplement: Supplementary file 3 [file DataSheet_2.zip › ROS/Fig3/Control Exo/2.jpg]

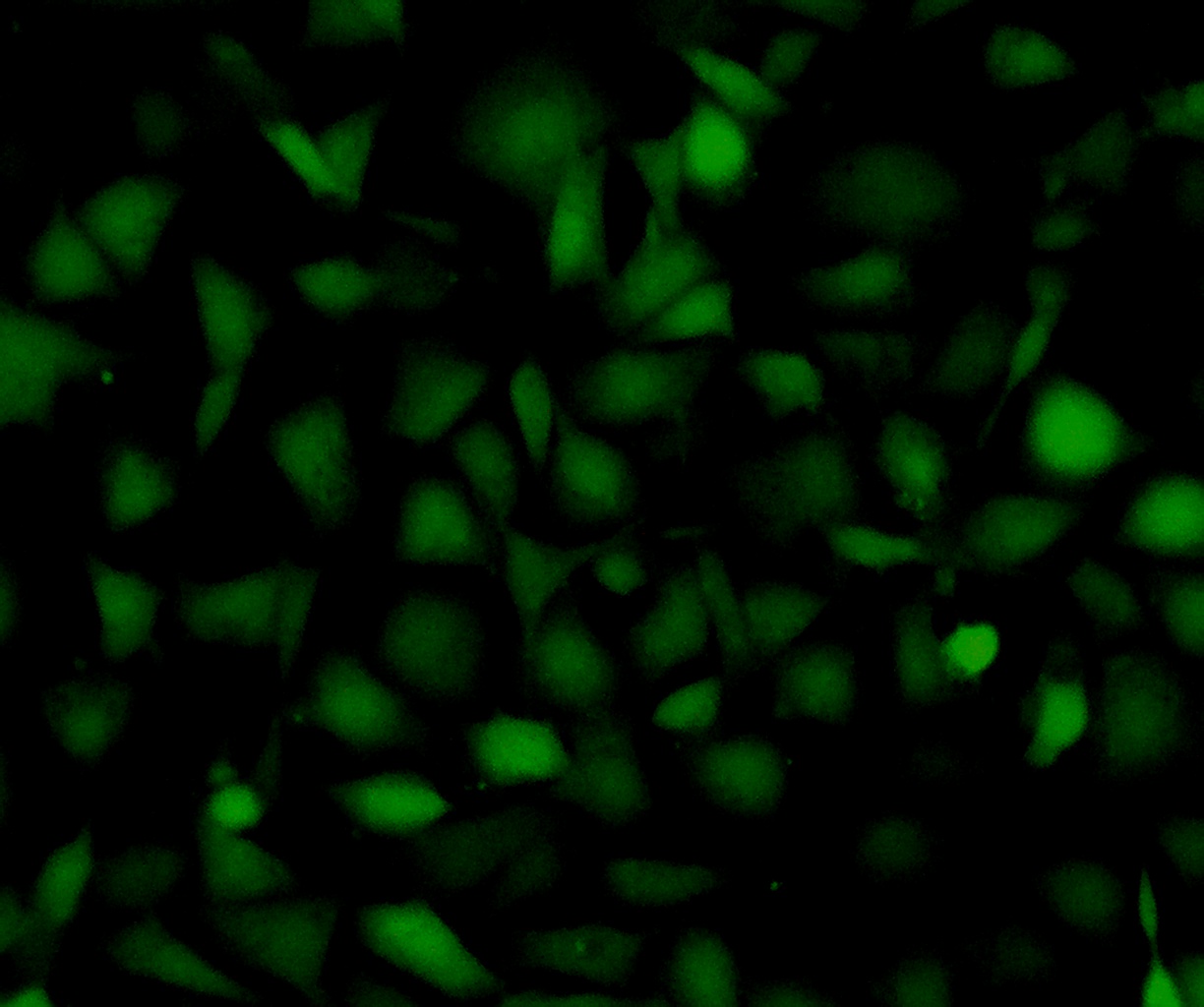

Supplement: Supplementary file 3 [file DataSheet_2.zip › ROS/Fig3/Control Exo/3.jpg]

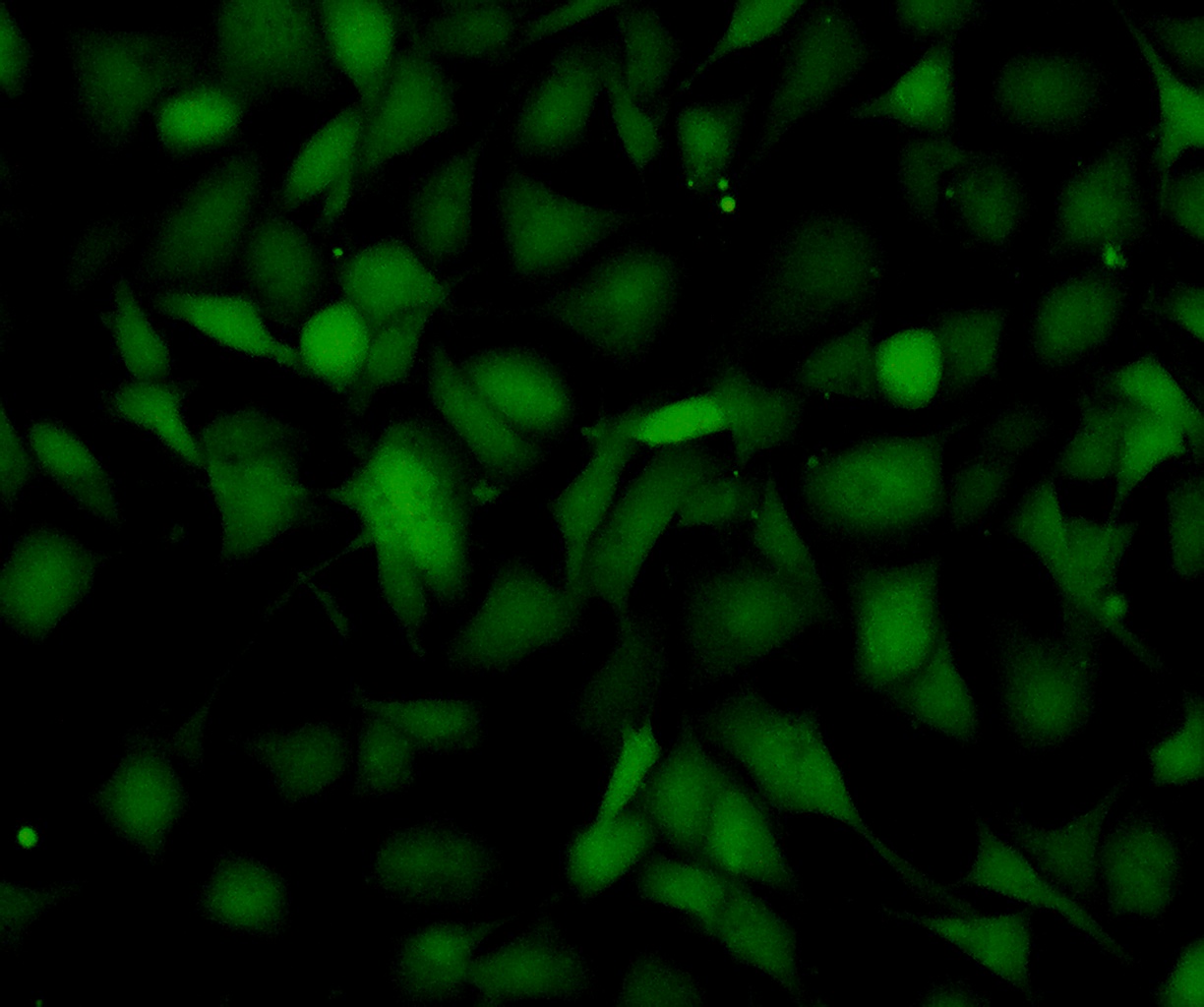

Supplement: Supplementary file 3 [file DataSheet_2.zip › ROS/Fig3/NC(+) Exo/1.jpg]

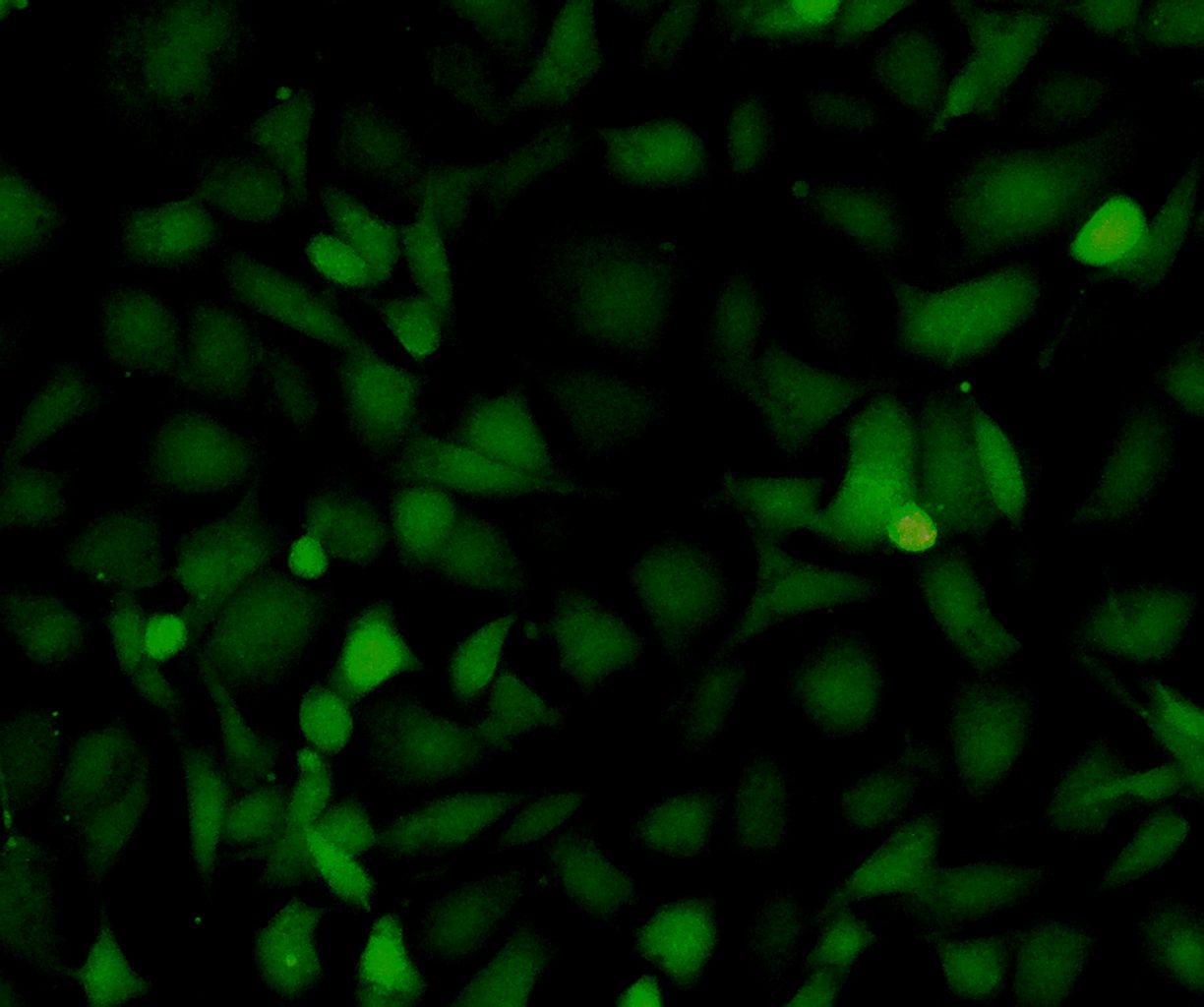

Supplement: Supplementary file 3 [file DataSheet_2.zip › ROS/Fig3/NC(+) Exo/2.jpg]

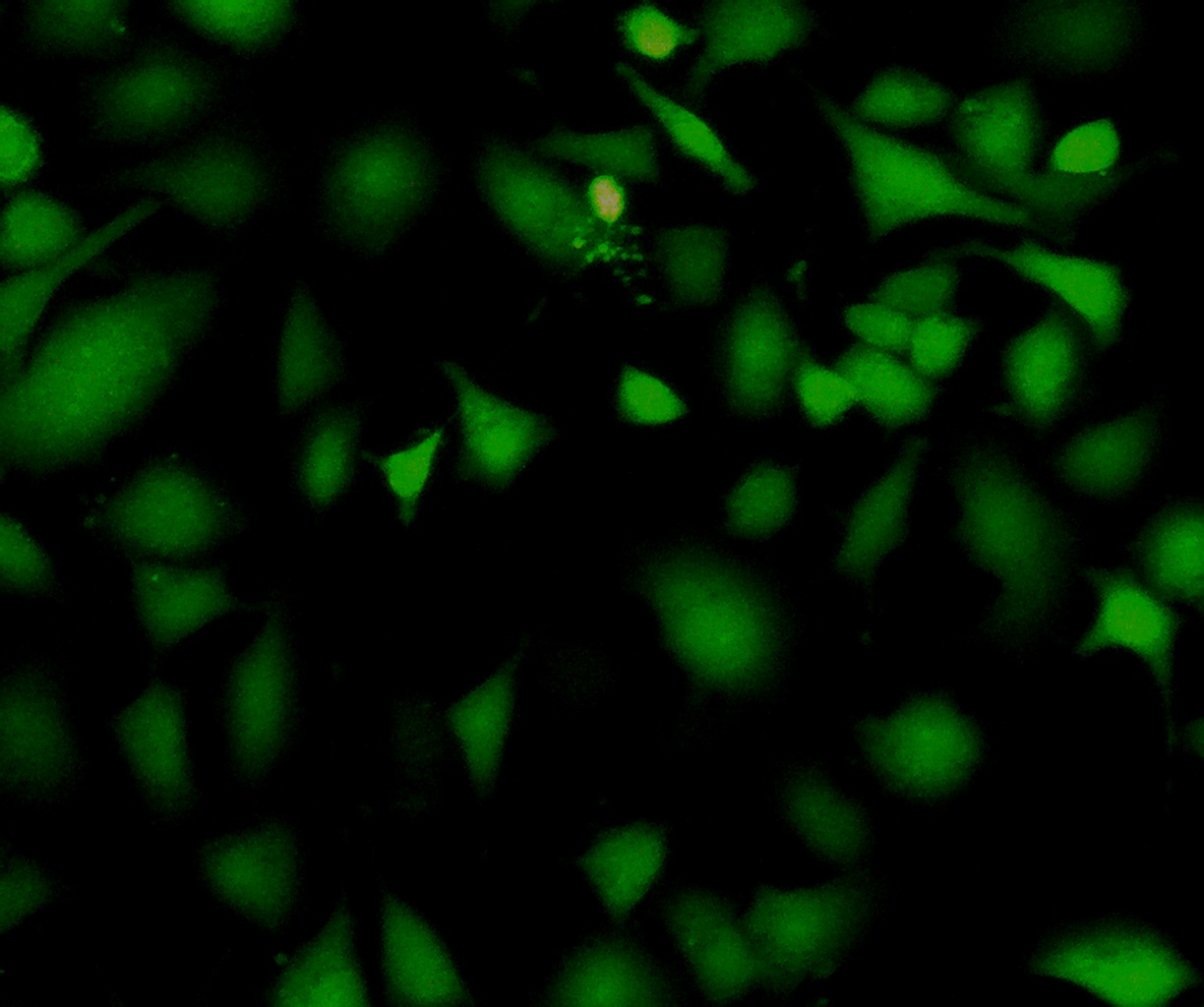

Supplement: Supplementary file 3 [file DataSheet_2.zip › ROS/Fig3/NC(+) Exo/3.jpg]

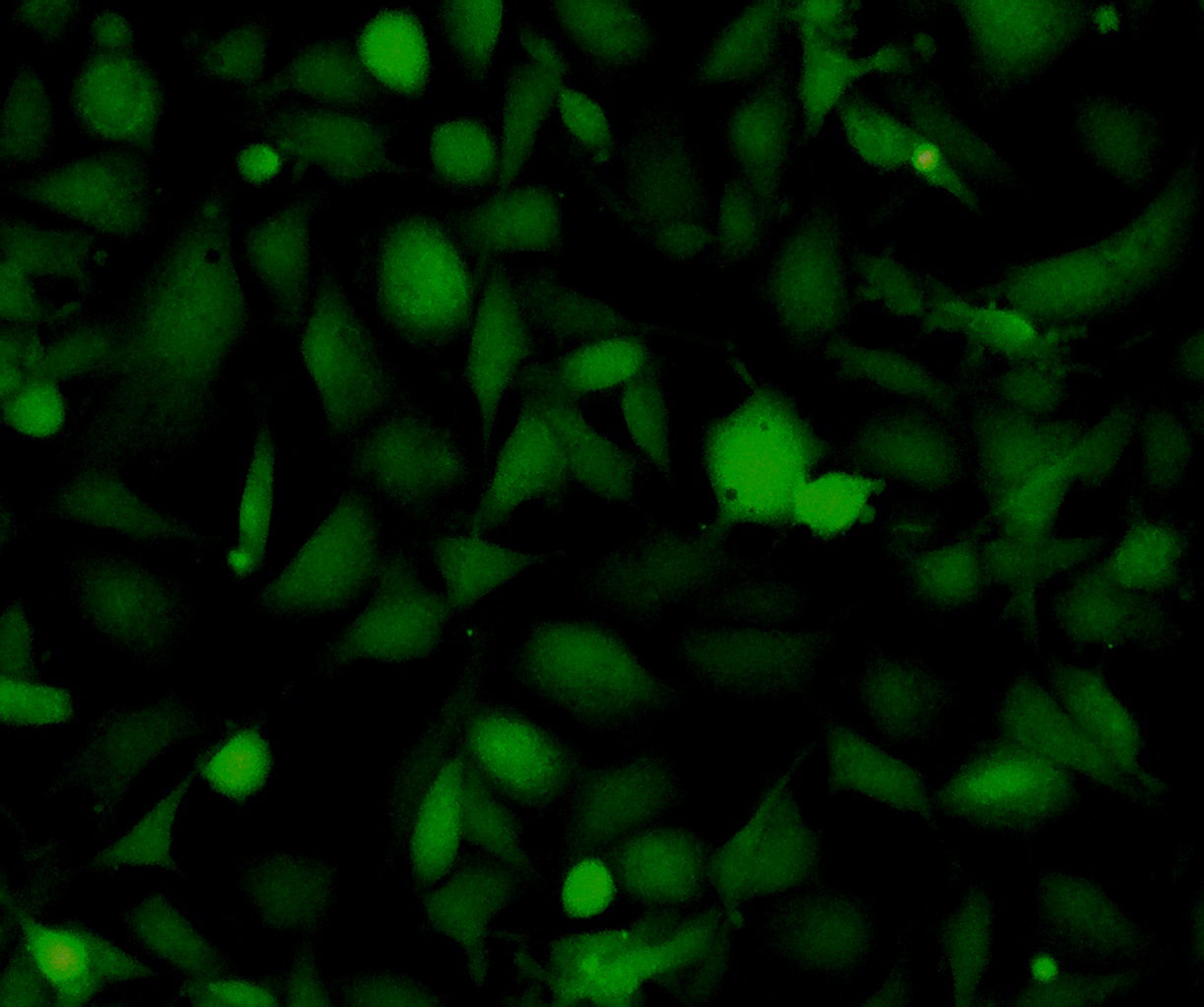

Supplement: Supplementary file 3 [file DataSheet_2.zip › ROS/Fig3/NC(-) Exo/1.jpg]

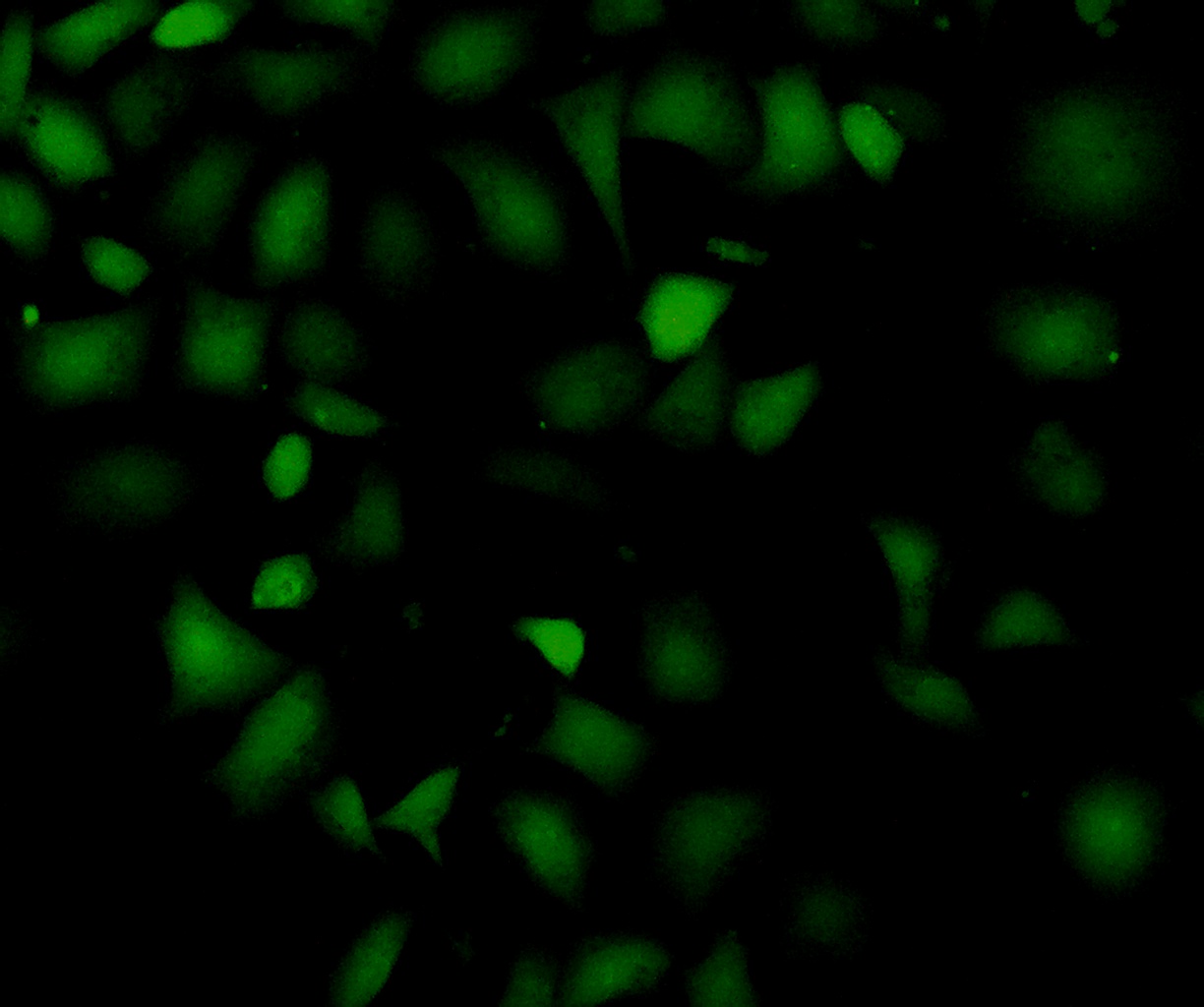

Supplement: Supplementary file 3 [file DataSheet_2.zip › ROS/Fig3/NC(-) Exo/2.jpg]

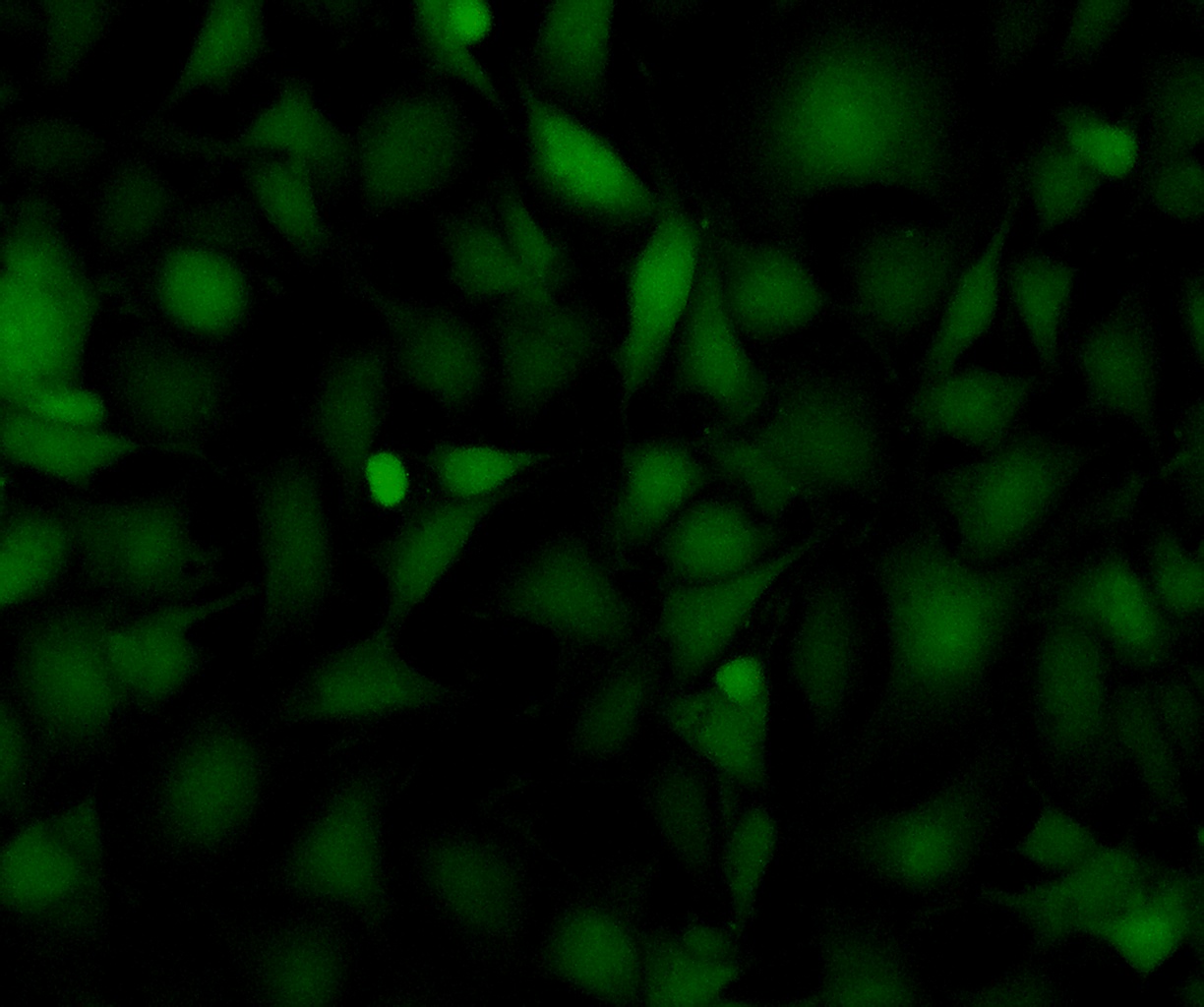

Supplement: Supplementary file 3 [file DataSheet_2.zip › ROS/Fig3/NC(-) Exo/3.jpg]

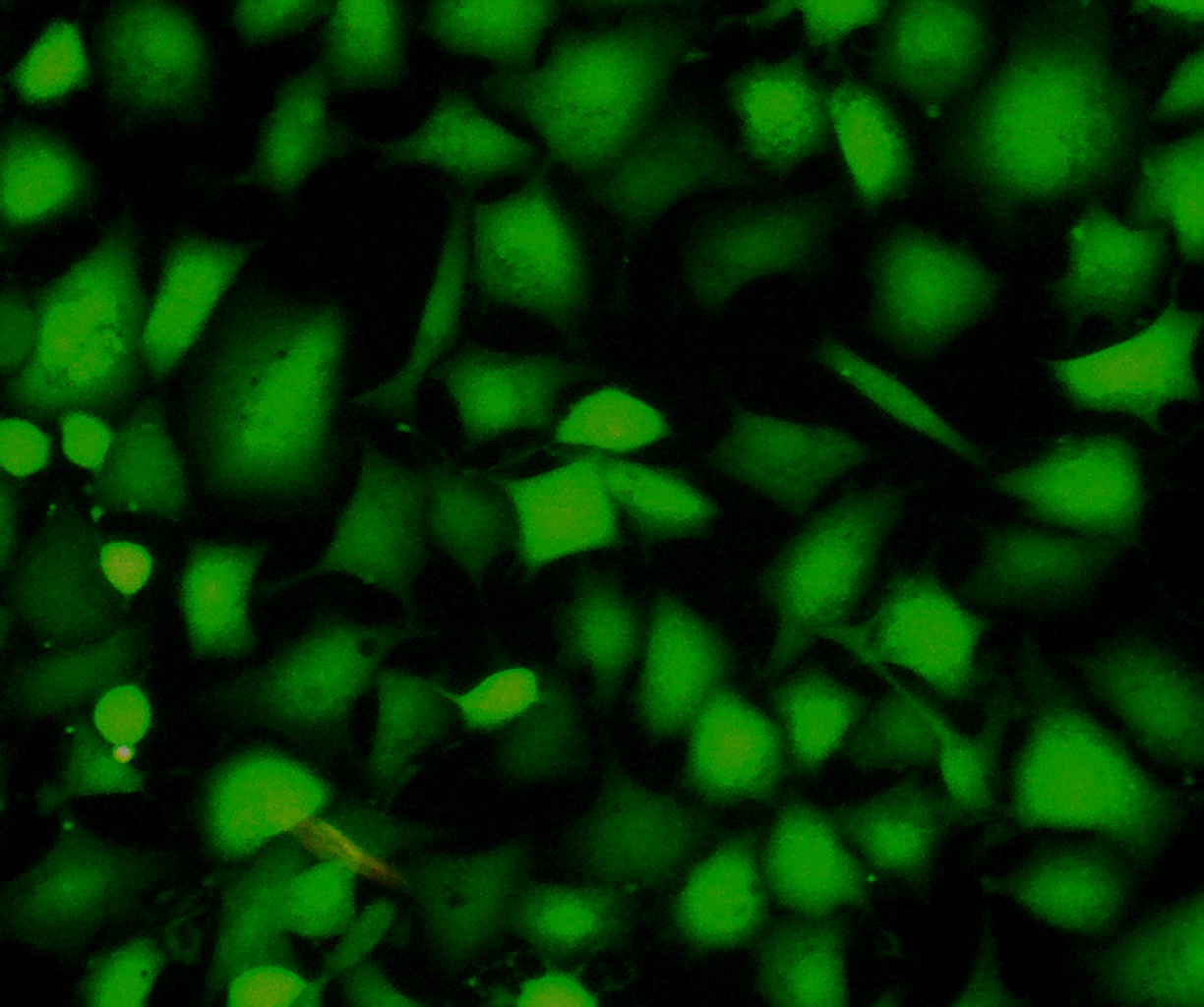

Supplement: Supplementary file 3 [file DataSheet_2.zip › ROS/Fig3/miR(+) Exo/1.jpg]

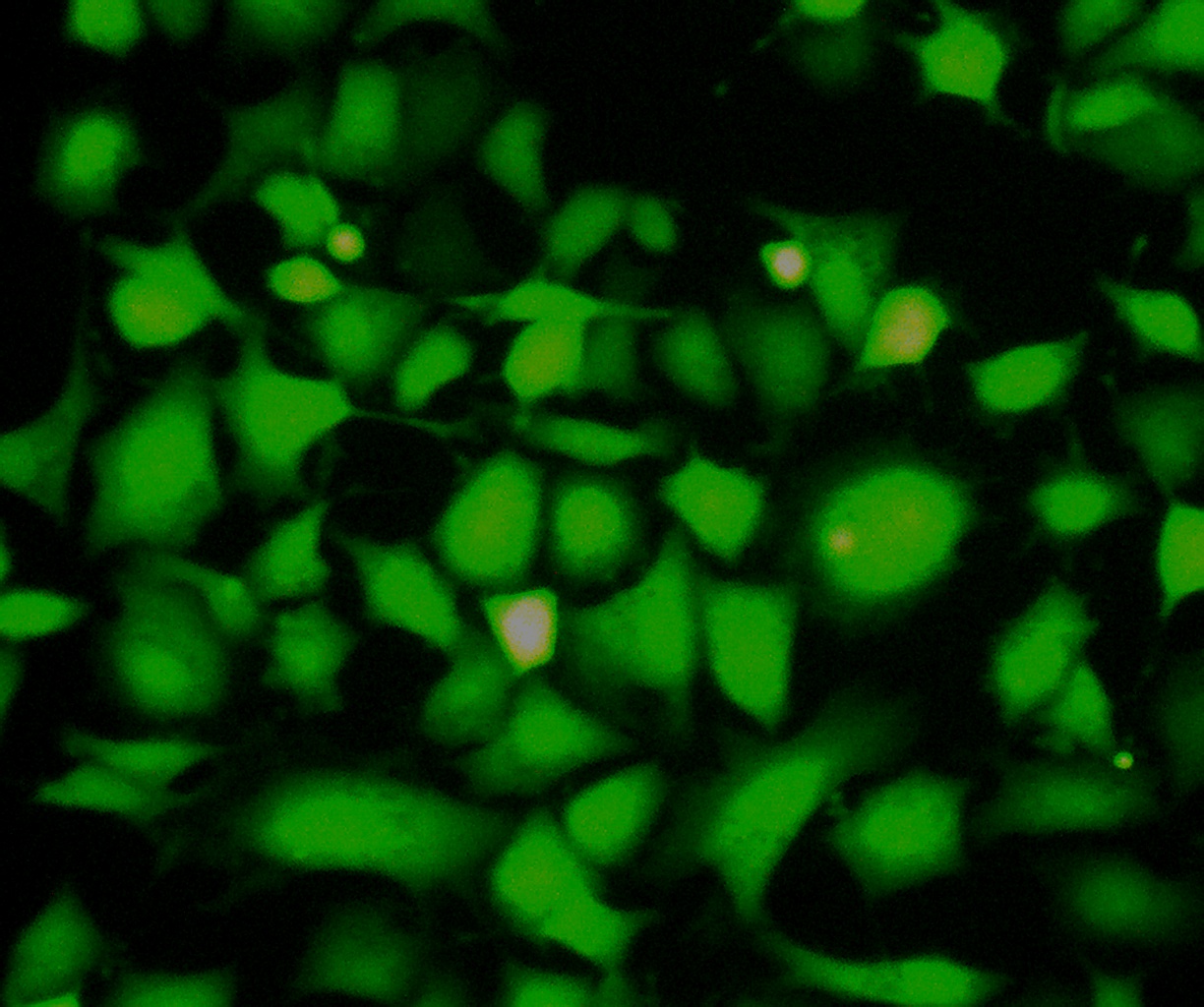

Supplement: Supplementary file 3 [file DataSheet_2.zip › ROS/Fig3/miR(+) Exo/2.jpg]

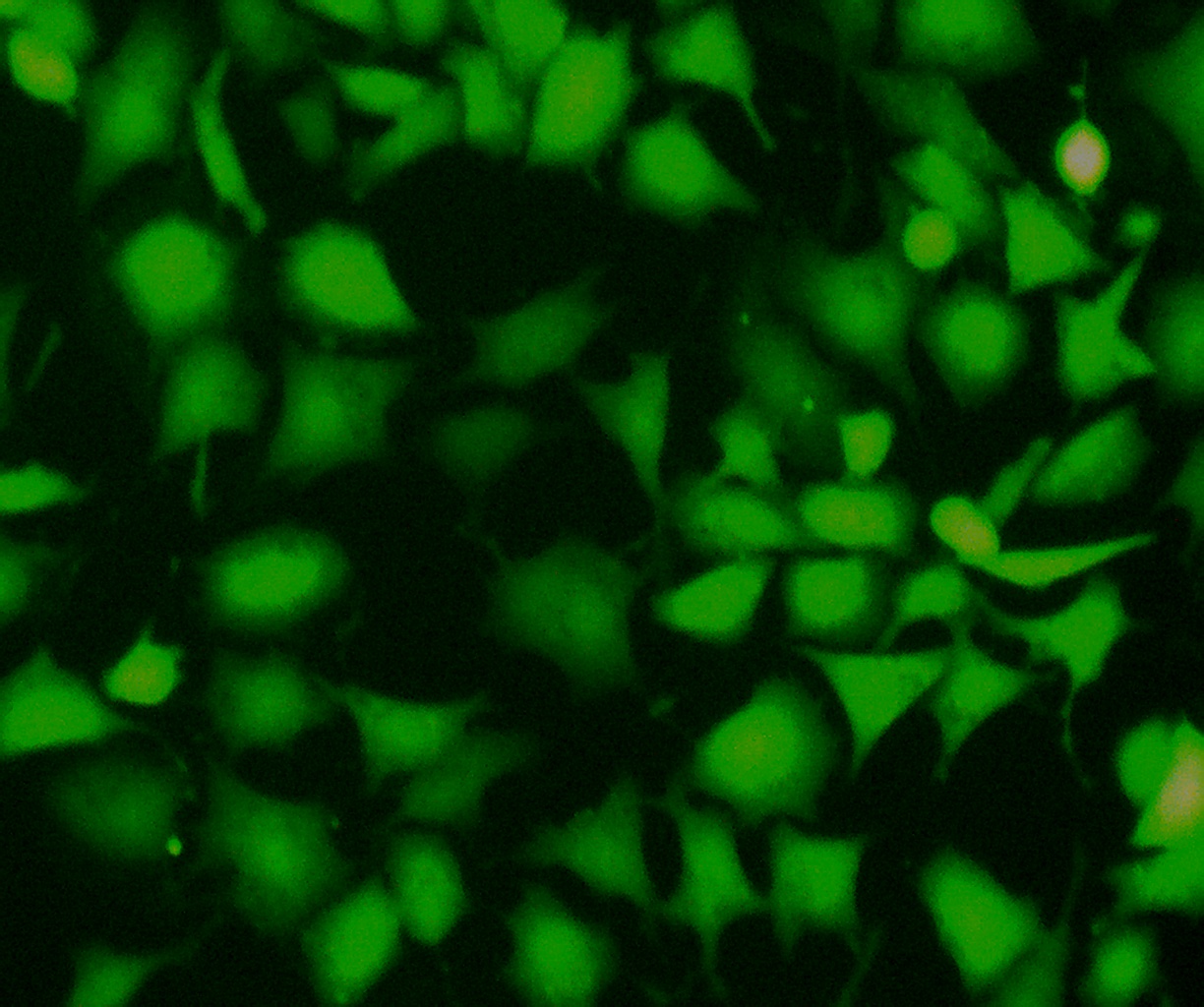

Supplement: Supplementary file 3 [file DataSheet_2.zip › ROS/Fig3/miR(+) Exo/3.jpg]

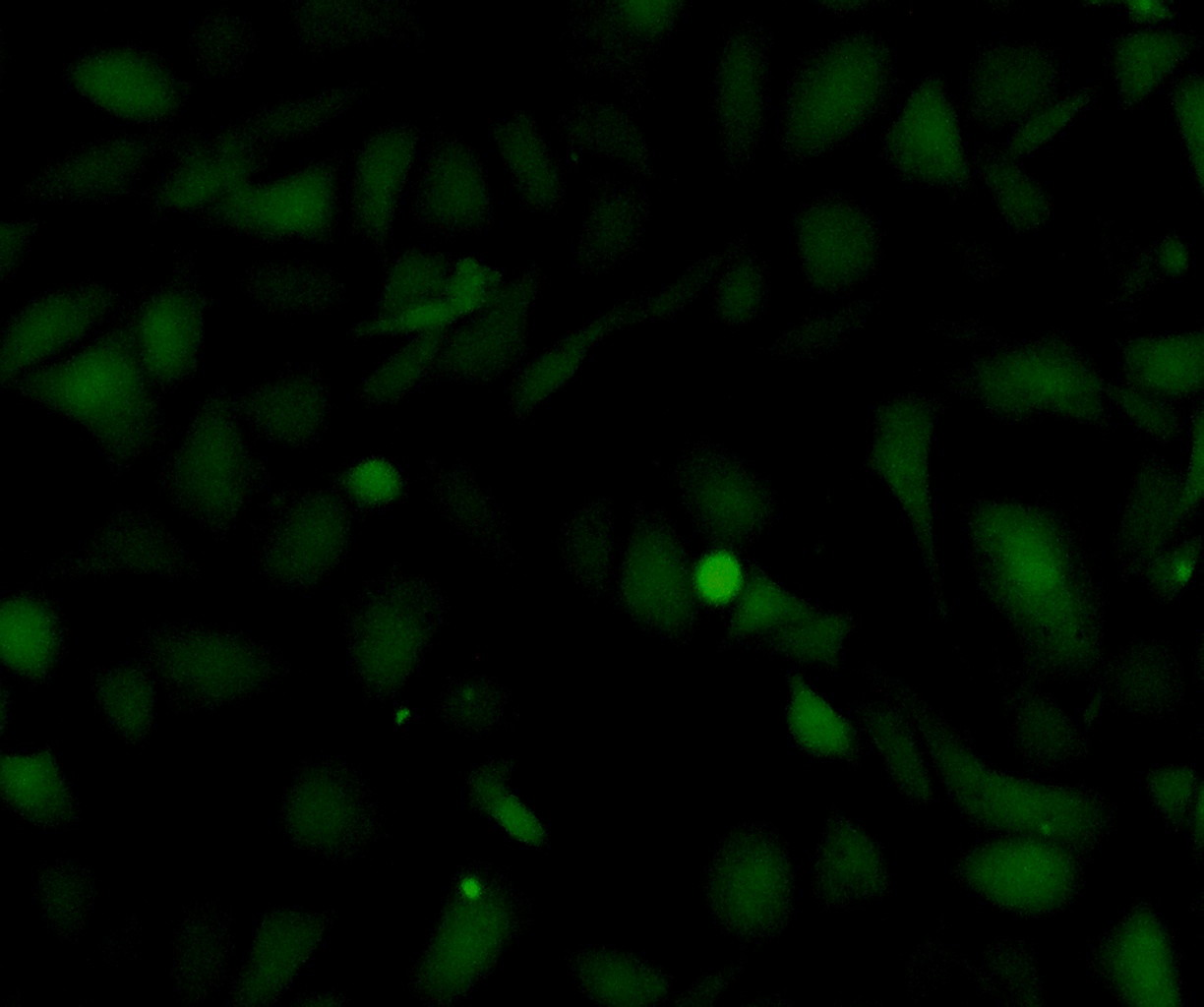

Supplement: Supplementary file 3 [file DataSheet_2.zip › ROS/Fig3/miR(-) Exo/1.jpg]

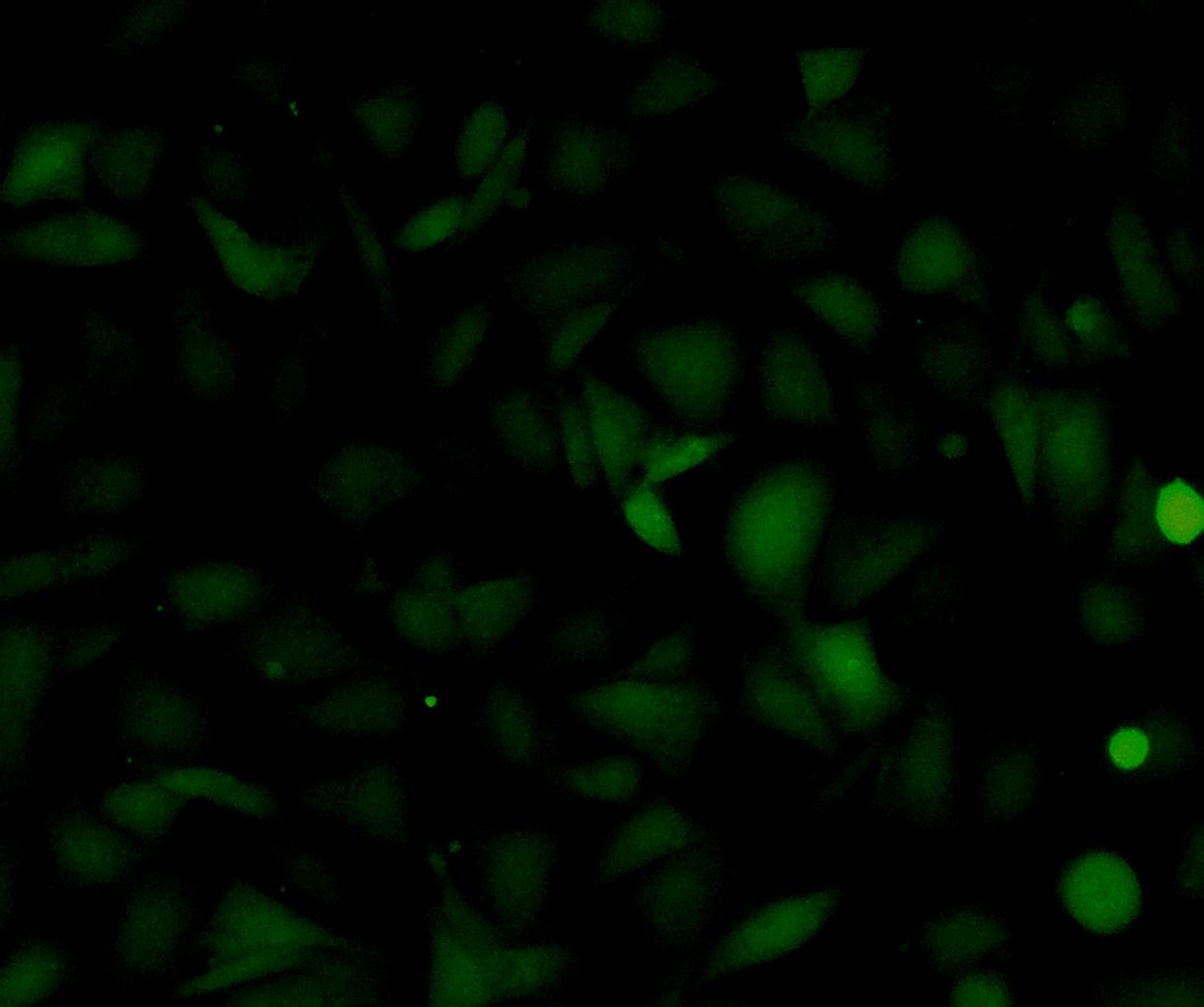

Supplement: Supplementary file 3 [file DataSheet_2.zip › ROS/Fig3/miR(-) Exo/2.jpg]

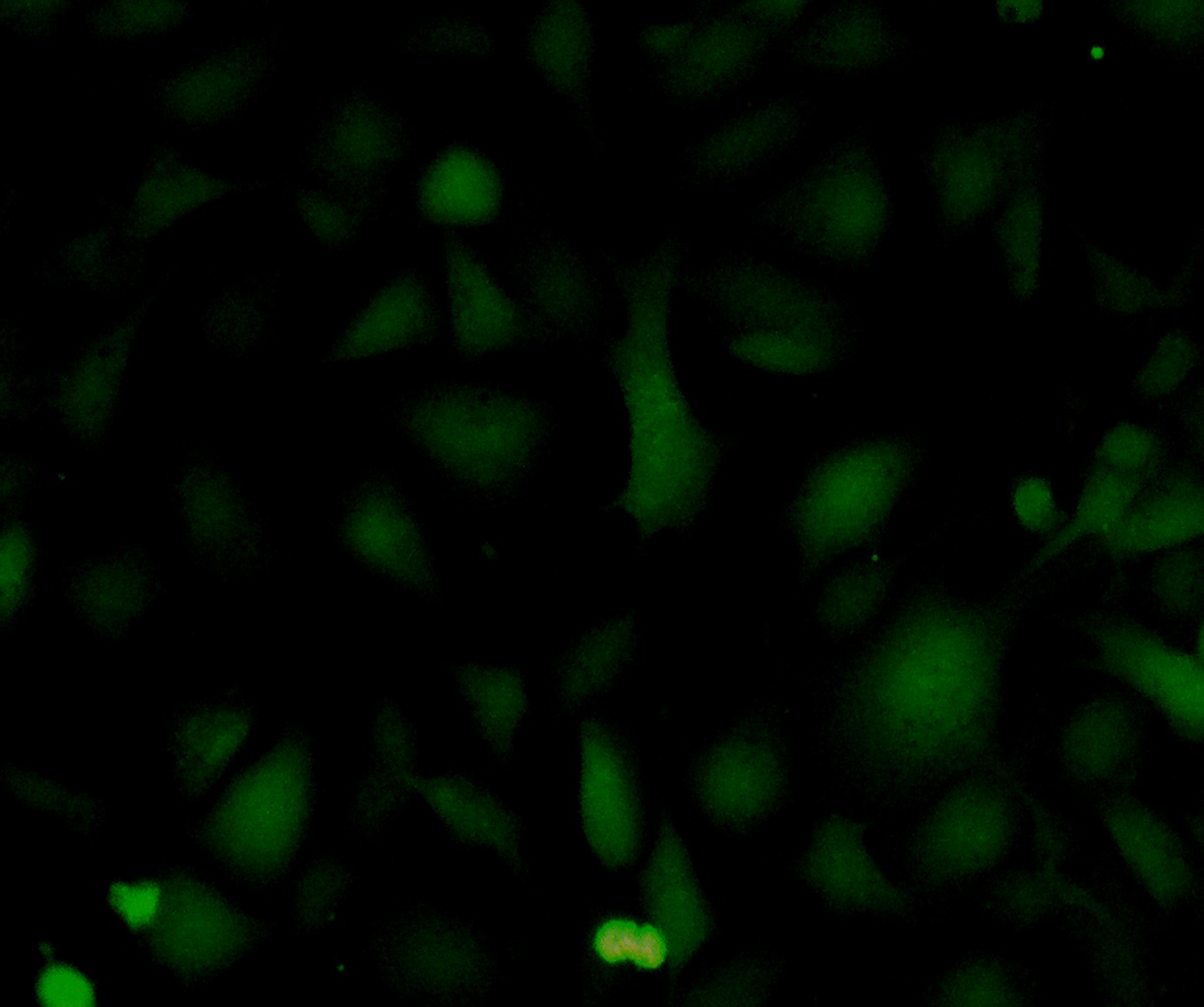

Supplement: Supplementary file 3 [file DataSheet_2.zip › ROS/Fig3/miR(-) Exo/3.jpg]

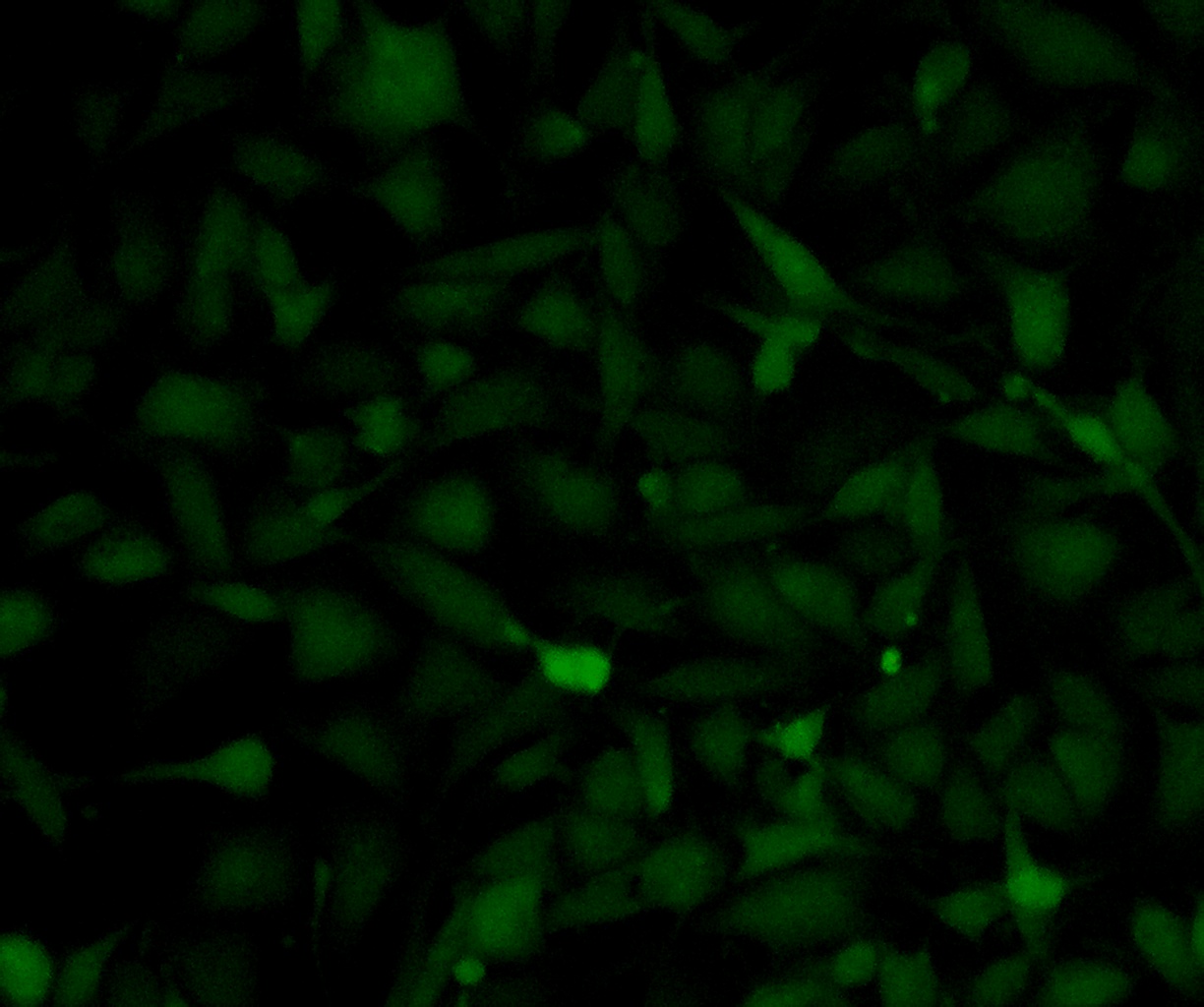

Supplement: Supplementary file 3 [file DataSheet_2.zip › ROS/Fig4/Control/1.jpg]

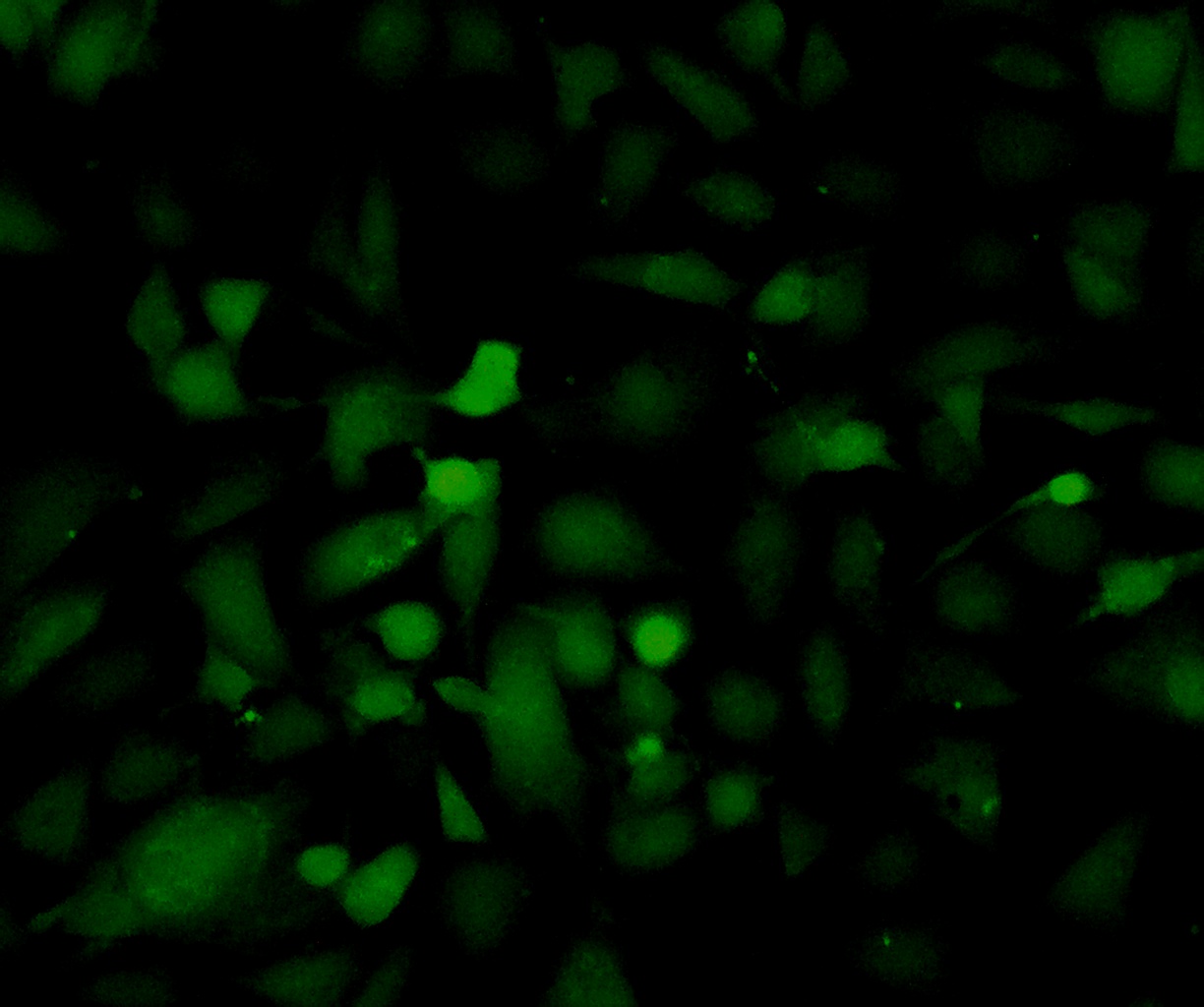

Supplement: Supplementary file 3 [file DataSheet_2.zip › ROS/Fig4/Control/2.jpg]

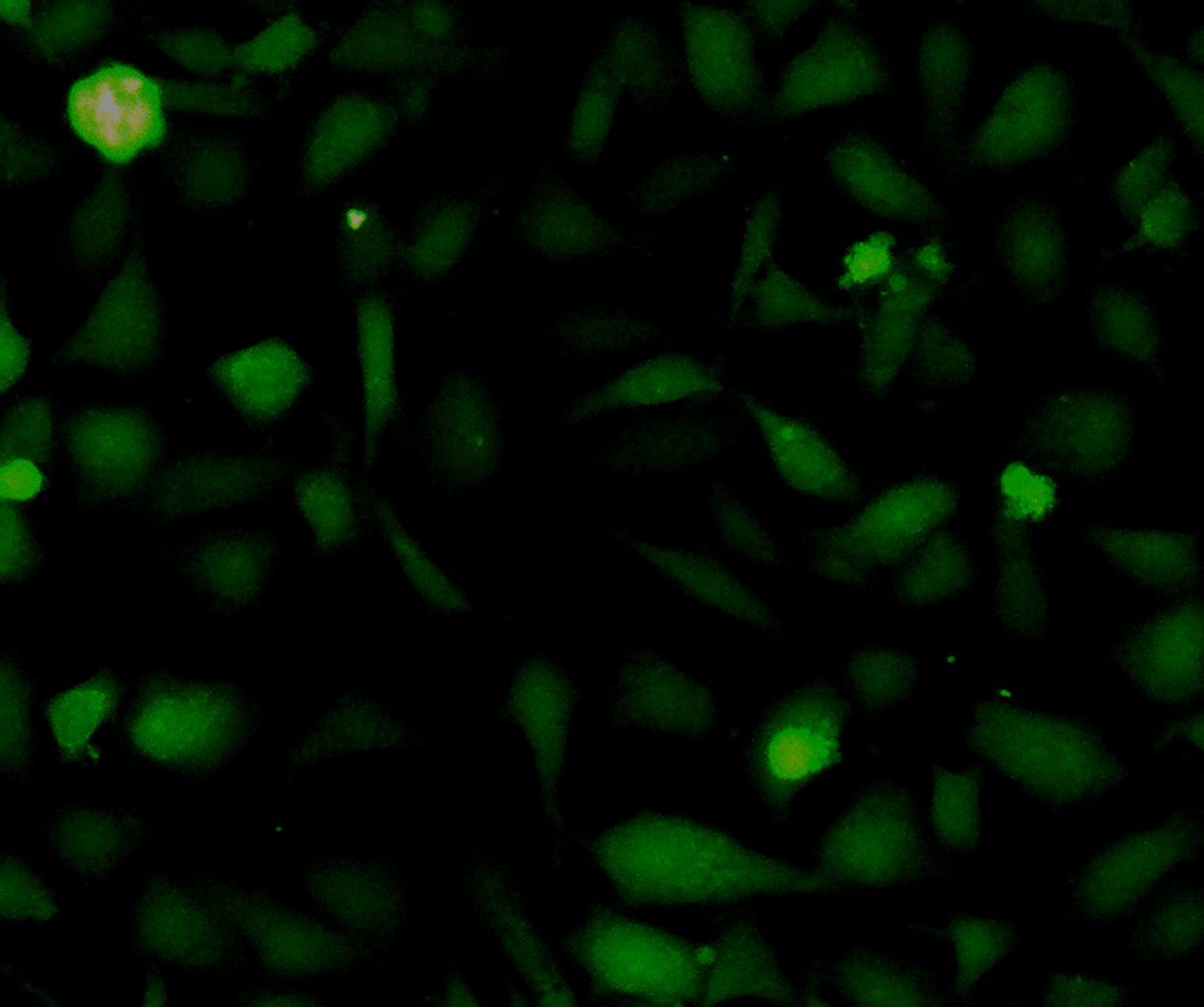

Supplement: Supplementary file 3 [file DataSheet_2.zip › ROS/Fig4/Control/3.jpg]

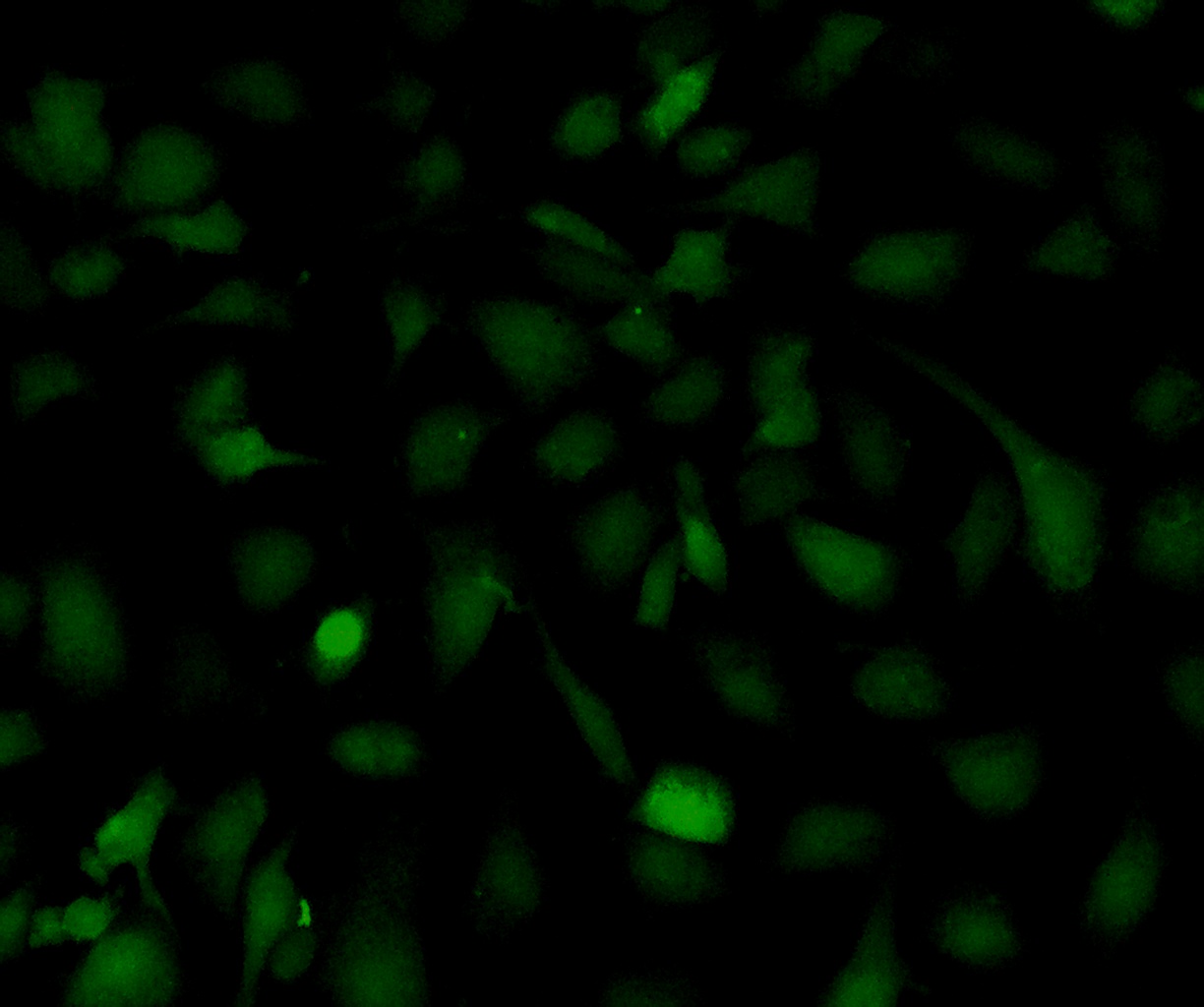

Supplement: Supplementary file 3 [file DataSheet_2.zip › ROS/Fig4/NC(+)/1.jpg]

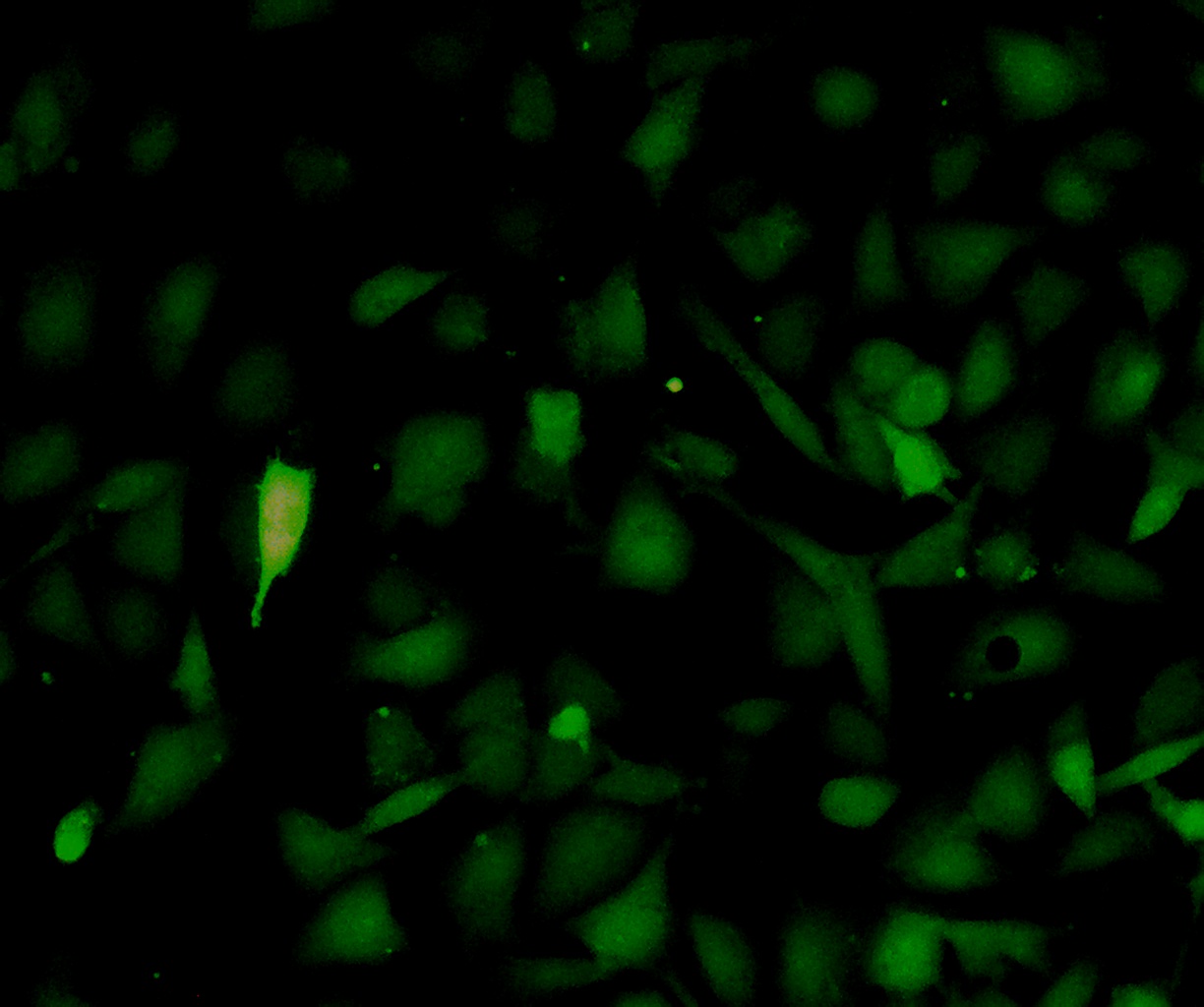

Supplement: Supplementary file 3 [file DataSheet_2.zip › ROS/Fig4/NC(+)/2.jpg]

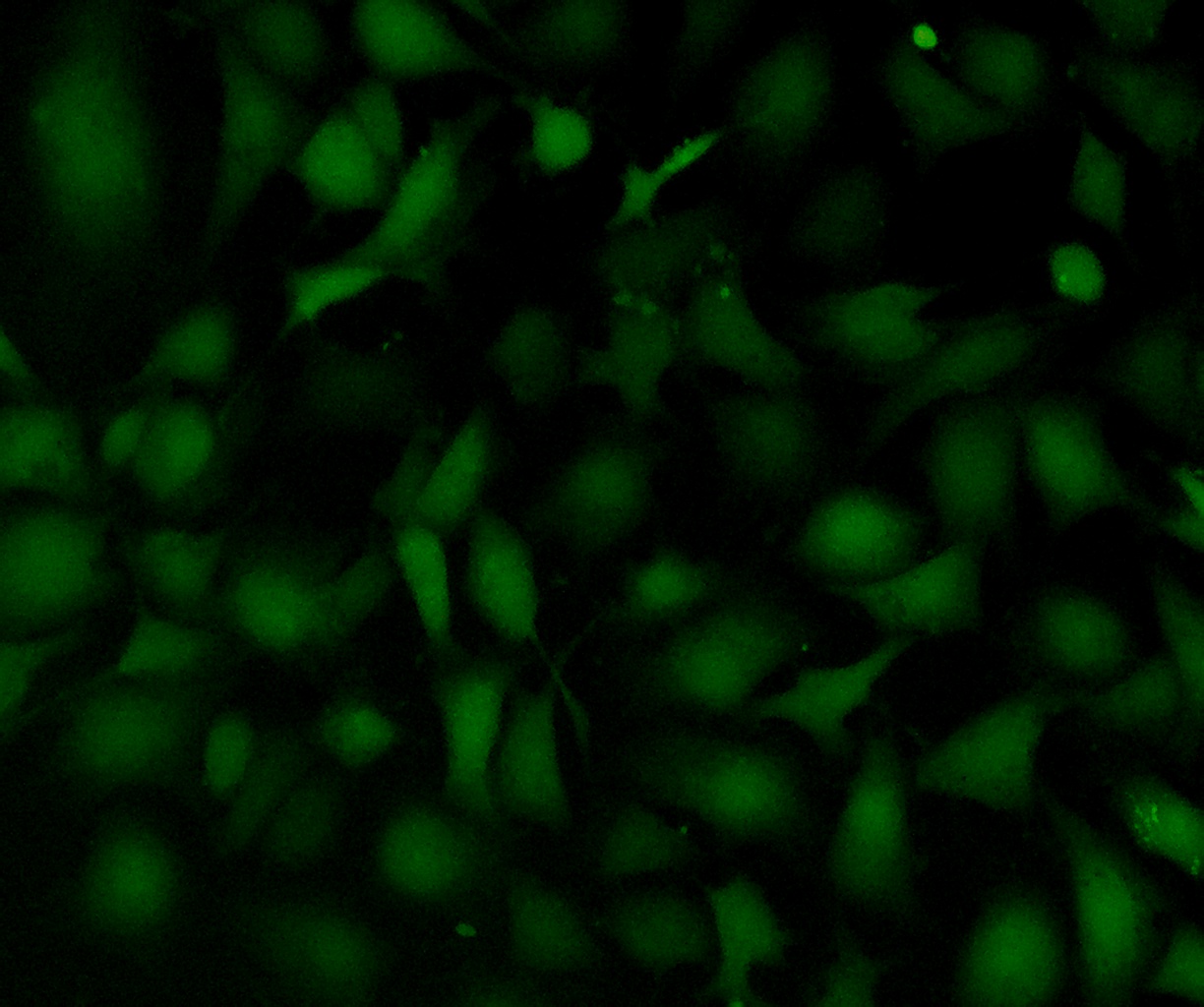

Supplement: Supplementary file 3 [file DataSheet_2.zip › ROS/Fig4/NC(+)/3.jpg]

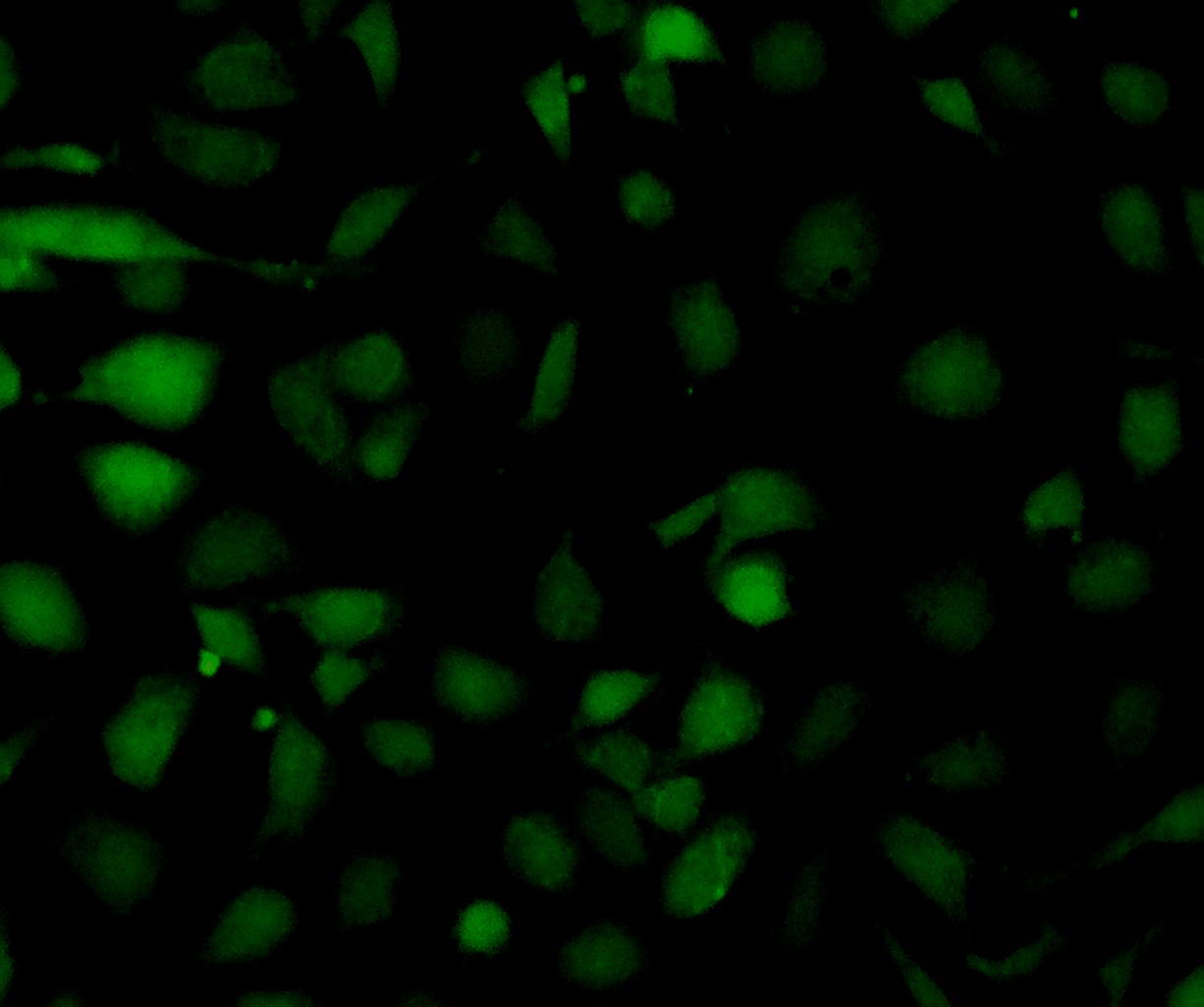

Supplement: Supplementary file 3 [file DataSheet_2.zip › ROS/Fig4/NC(-)/1.jpg]

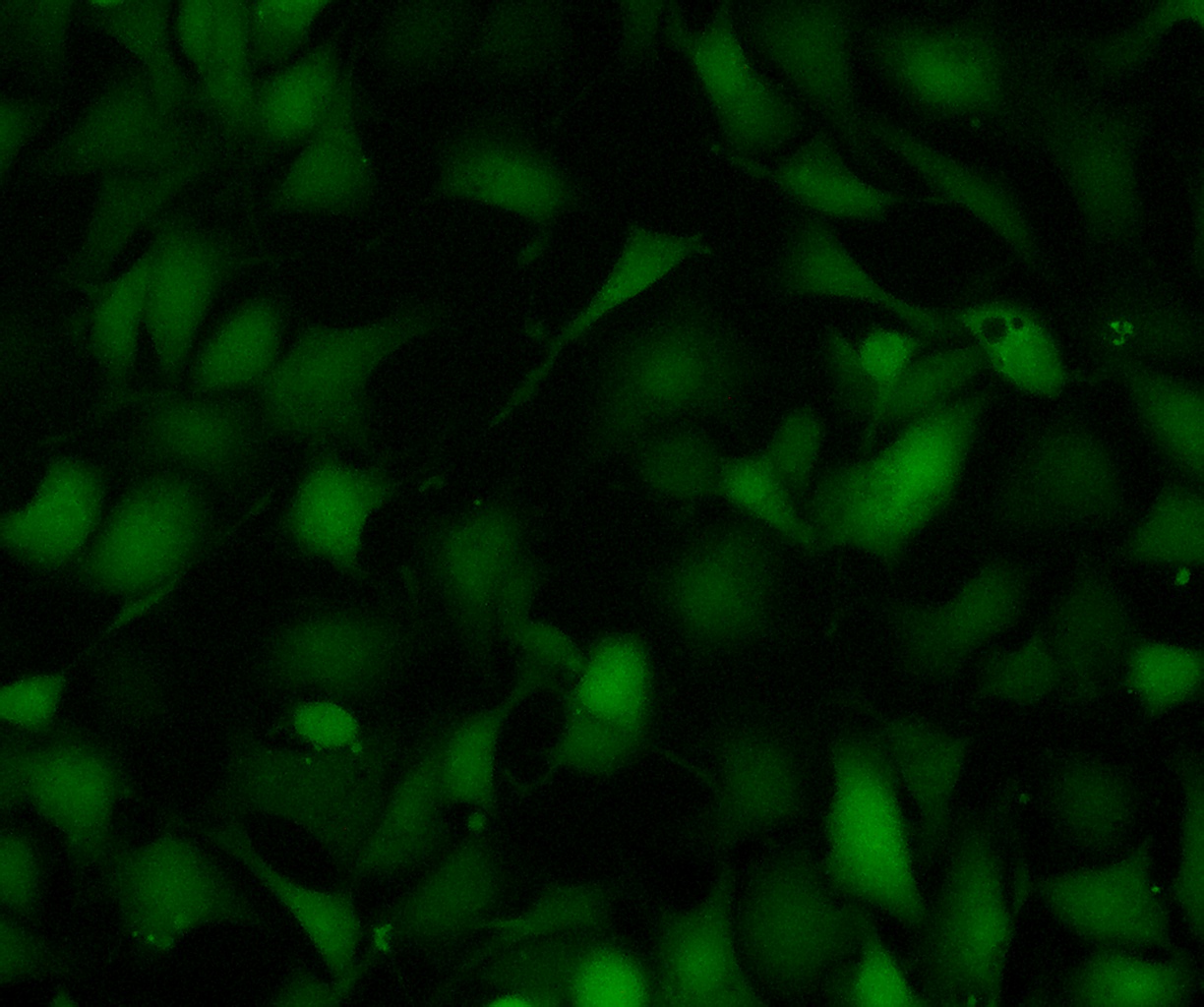

Supplement: Supplementary file 3 [file DataSheet_2.zip › ROS/Fig4/NC(-)/2.jpg]

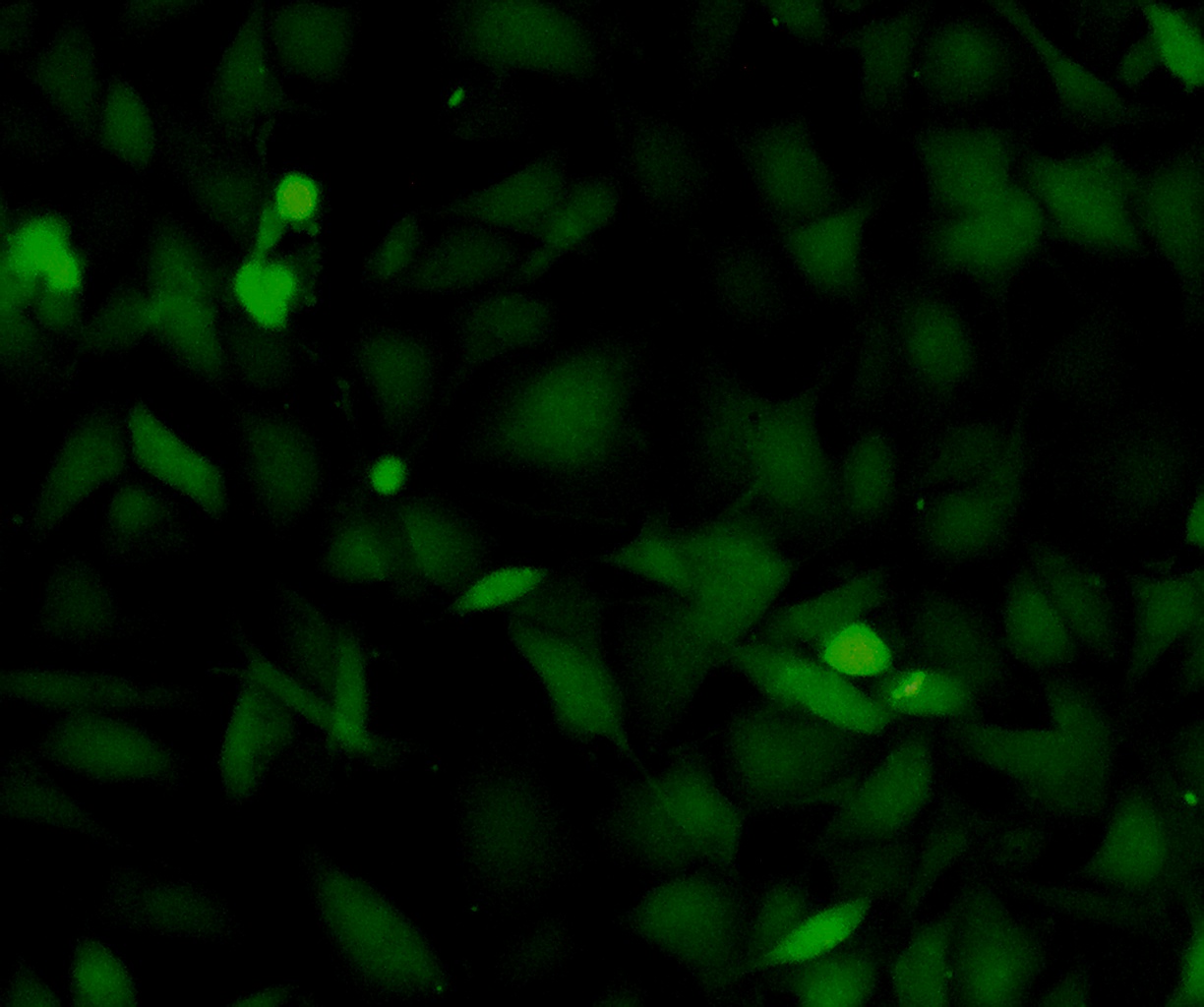

Supplement: Supplementary file 3 [file DataSheet_2.zip › ROS/Fig4/NC(-)/3.jpg]

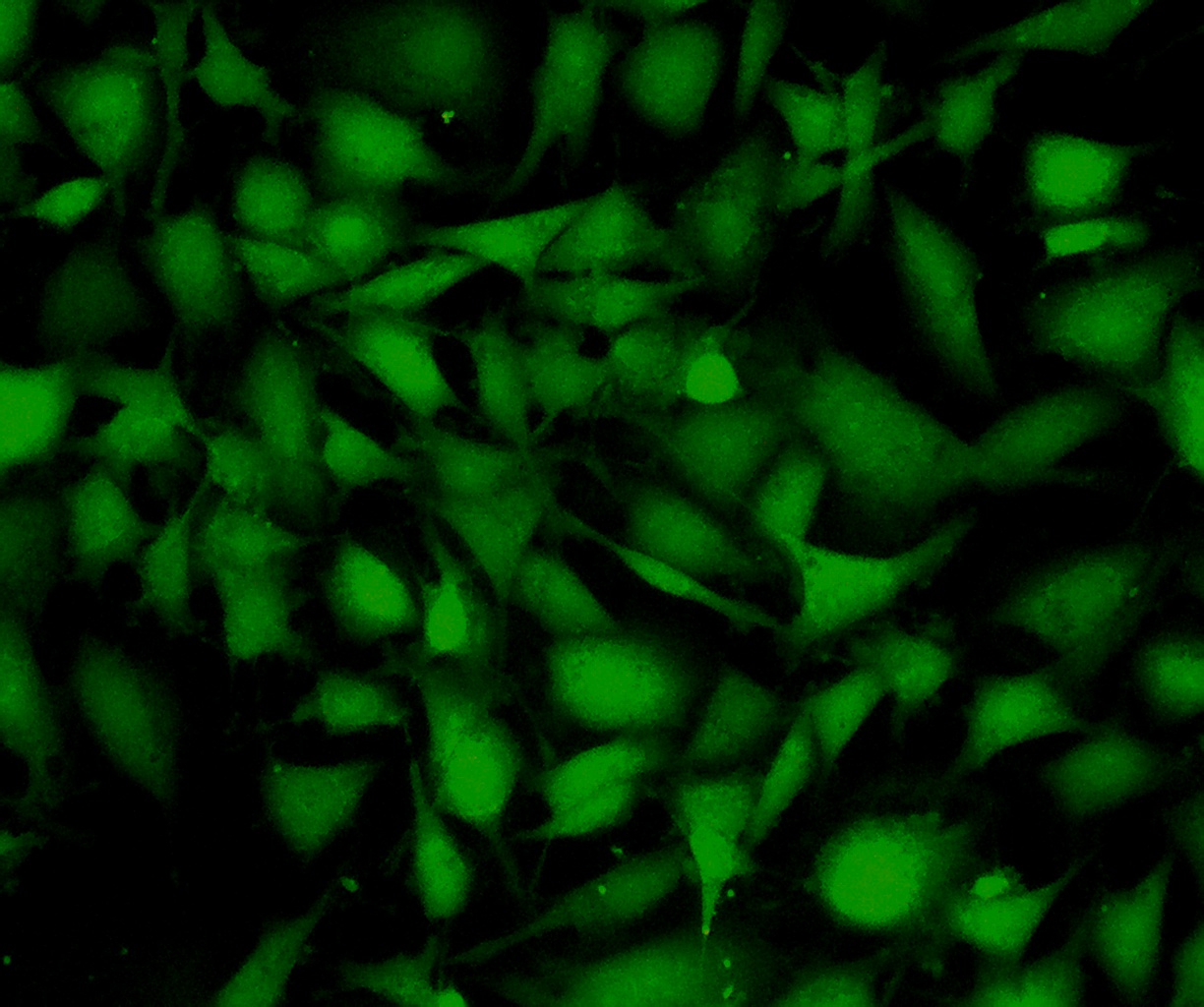

Supplement: Supplementary file 3 [file DataSheet_2.zip › ROS/Fig4/miR(+)/1.jpg]

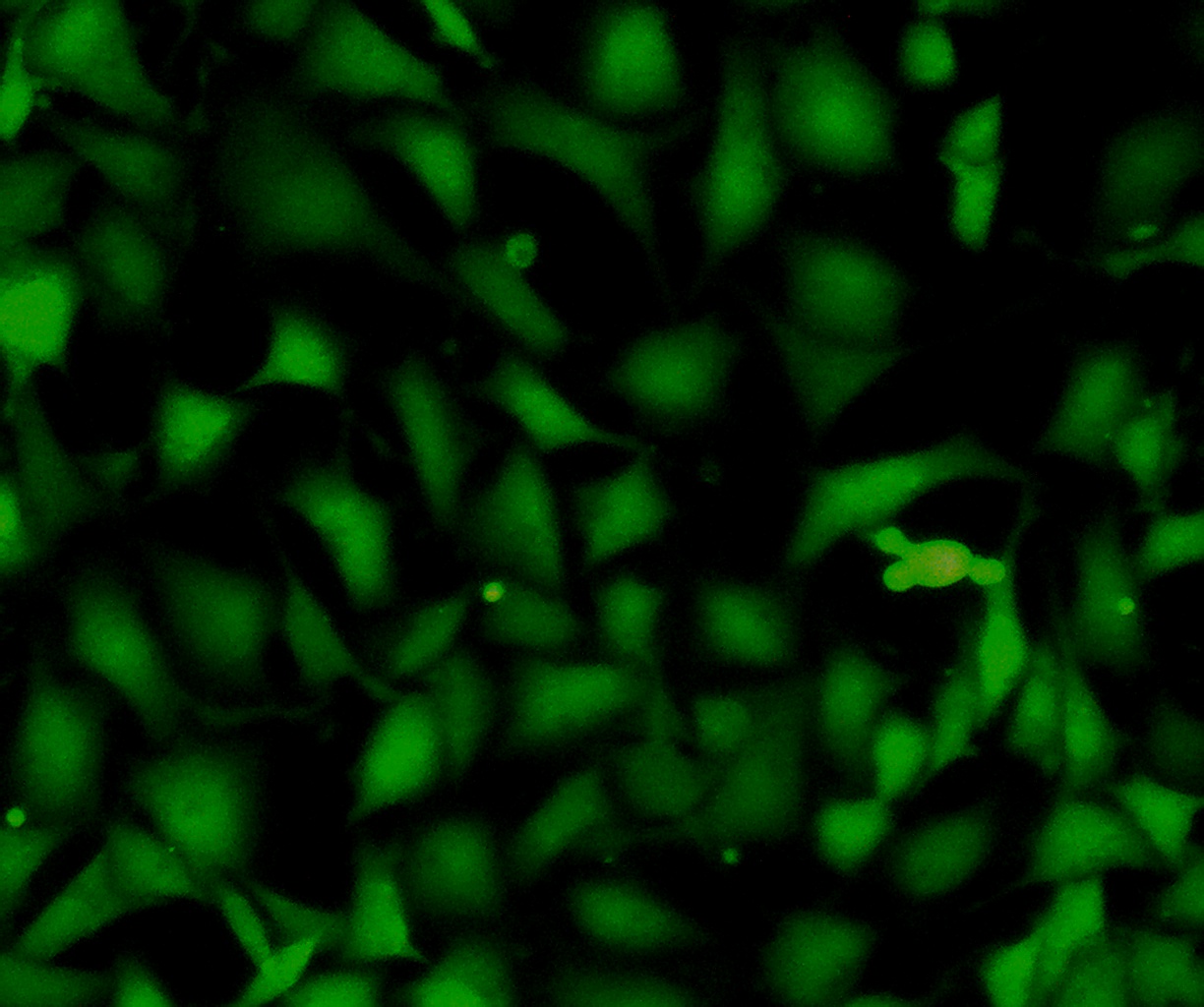

Supplement: Supplementary file 3 [file DataSheet_2.zip › ROS/Fig4/miR(+)/2.jpg]

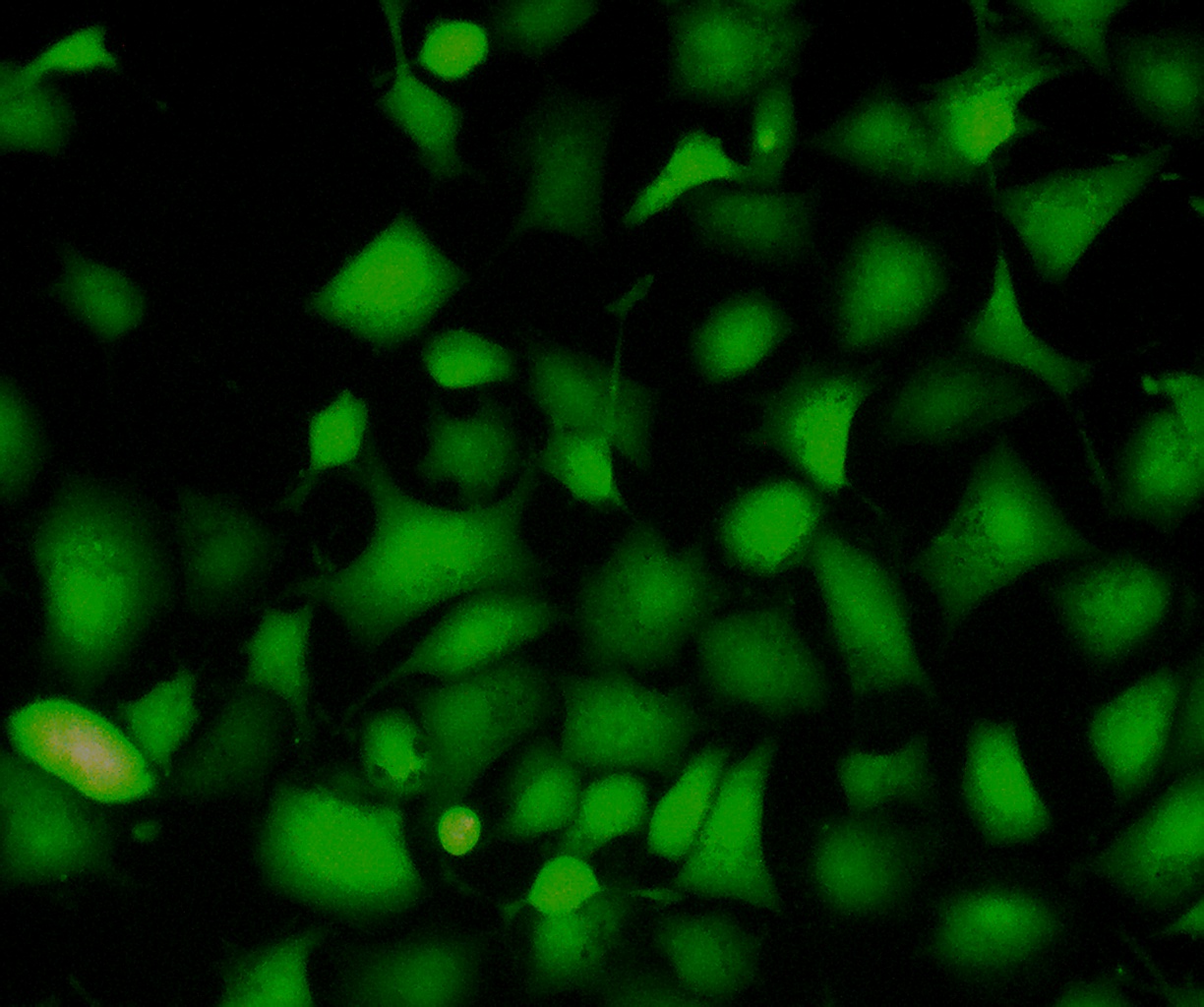

Supplement: Supplementary file 3 [file DataSheet_2.zip › ROS/Fig4/miR(+)/3.jpg]

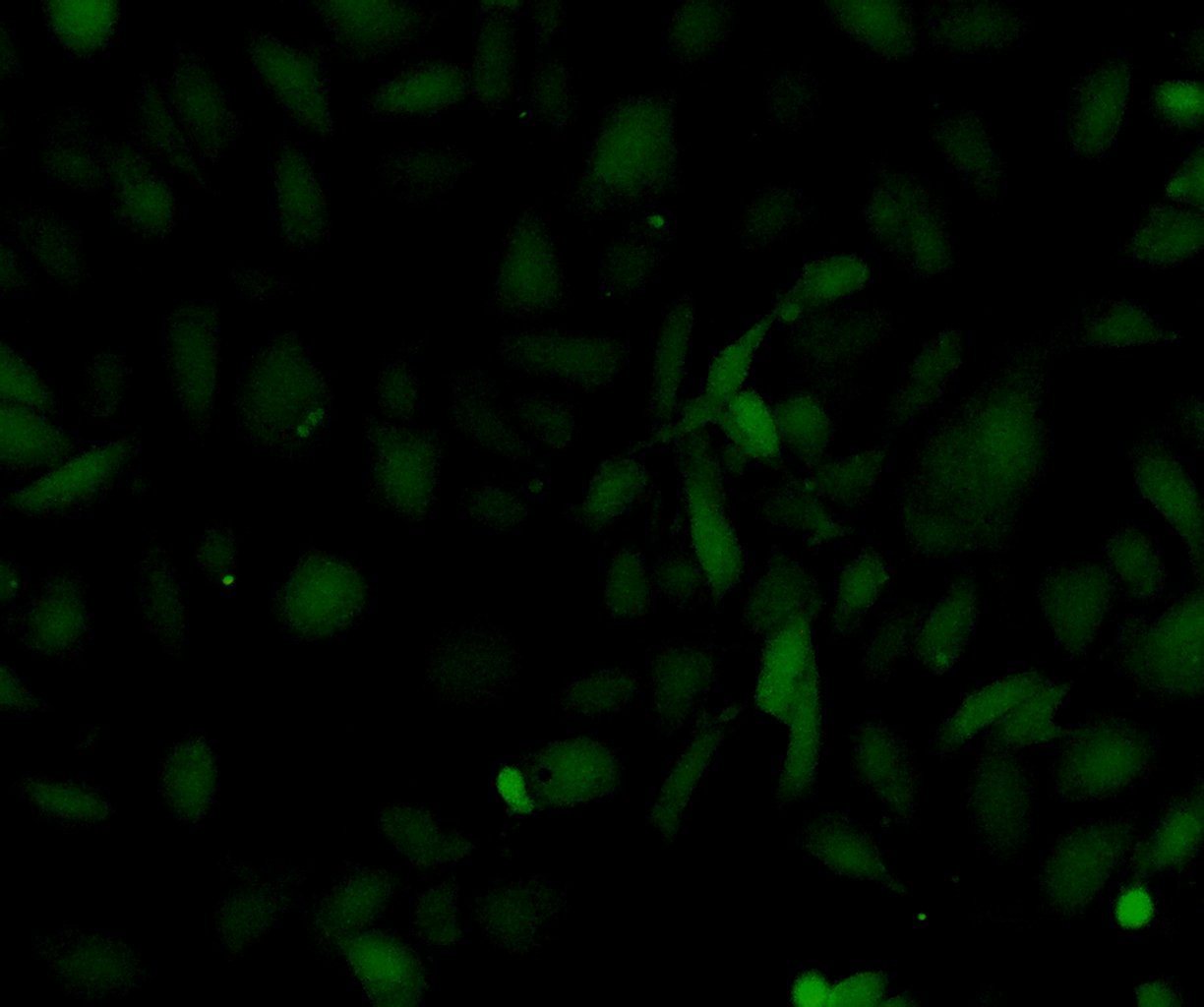

Supplement: Supplementary file 3 [file DataSheet_2.zip › ROS/Fig4/miR(-)/1.jpg]

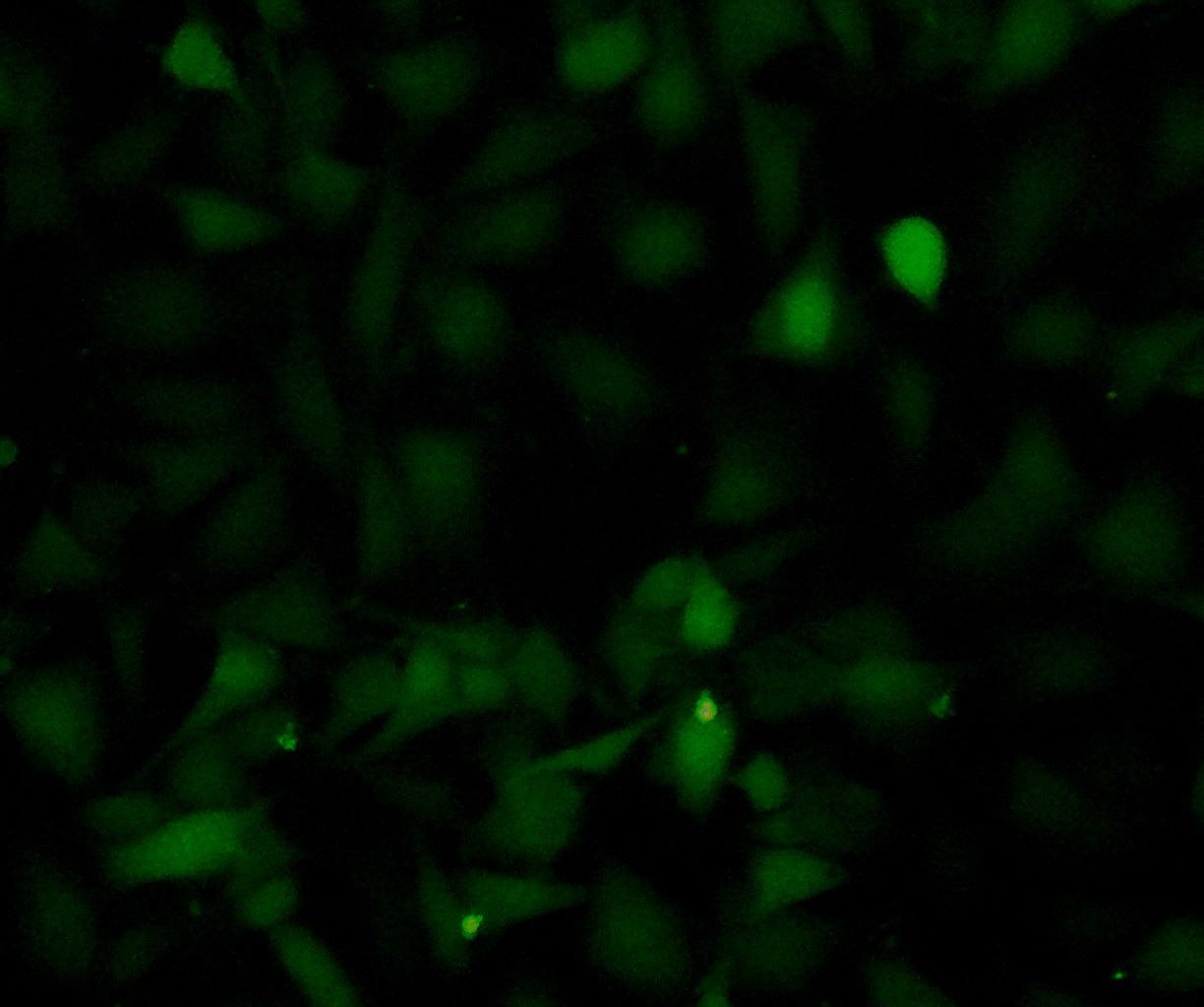

Supplement: Supplementary file 3 [file DataSheet_2.zip › ROS/Fig4/miR(-)/2.jpg]

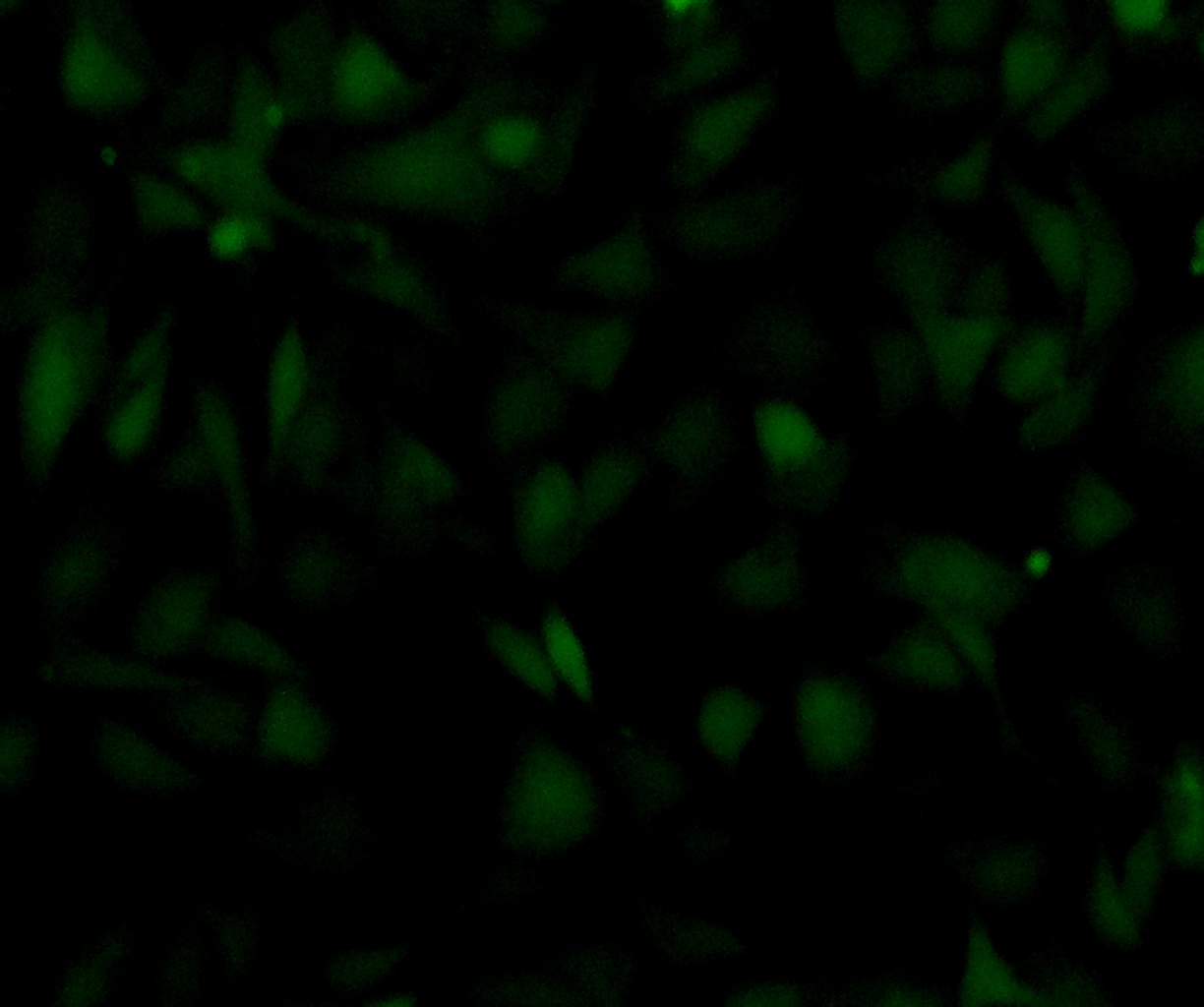

Supplement: Supplementary file 3 [file DataSheet_2.zip › ROS/Fig4/miR(-)/3.jpg]

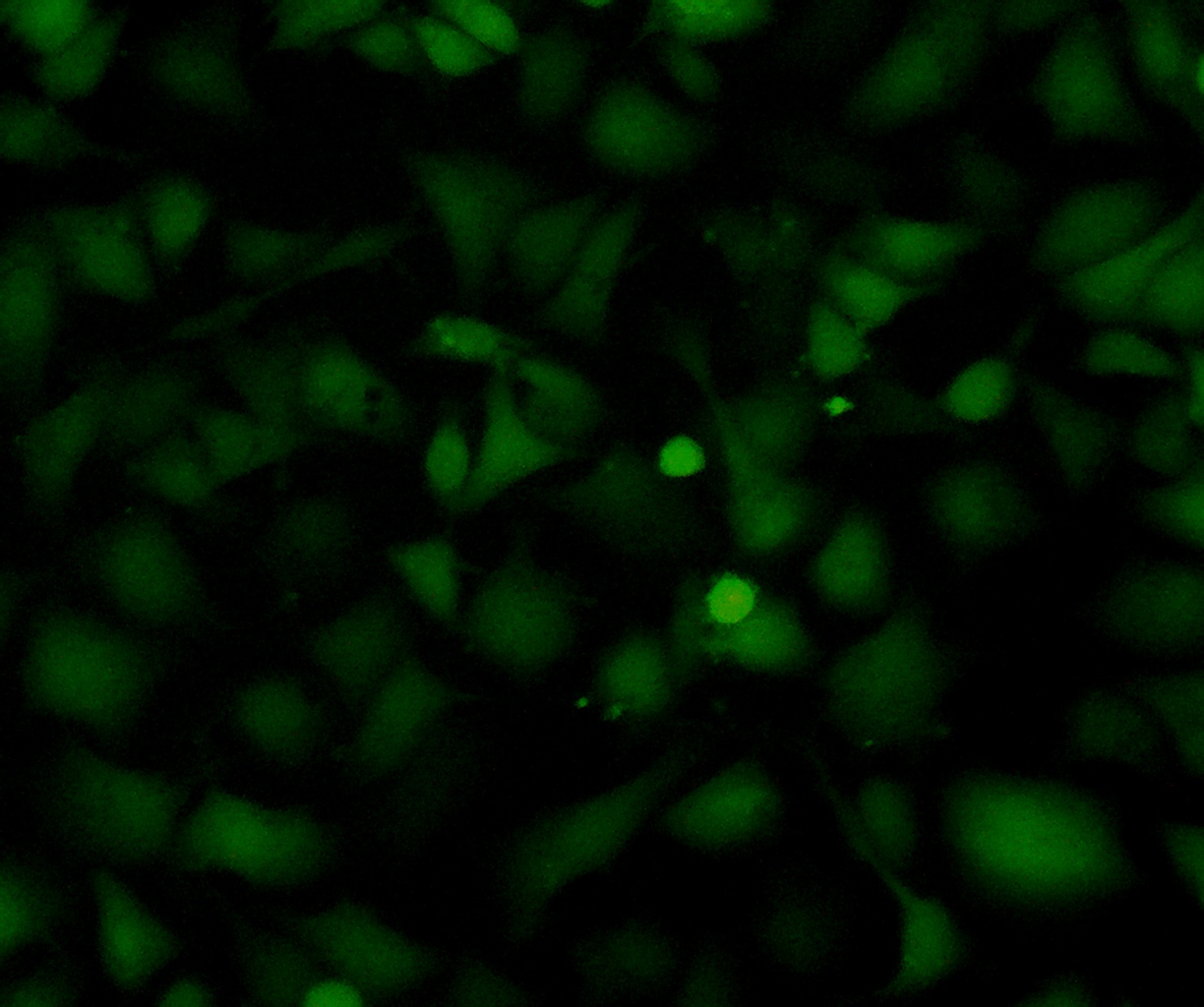

Supplement: Supplementary file 3 [file DataSheet_2.zip › ROS/Fig5/Control/1.jpg]

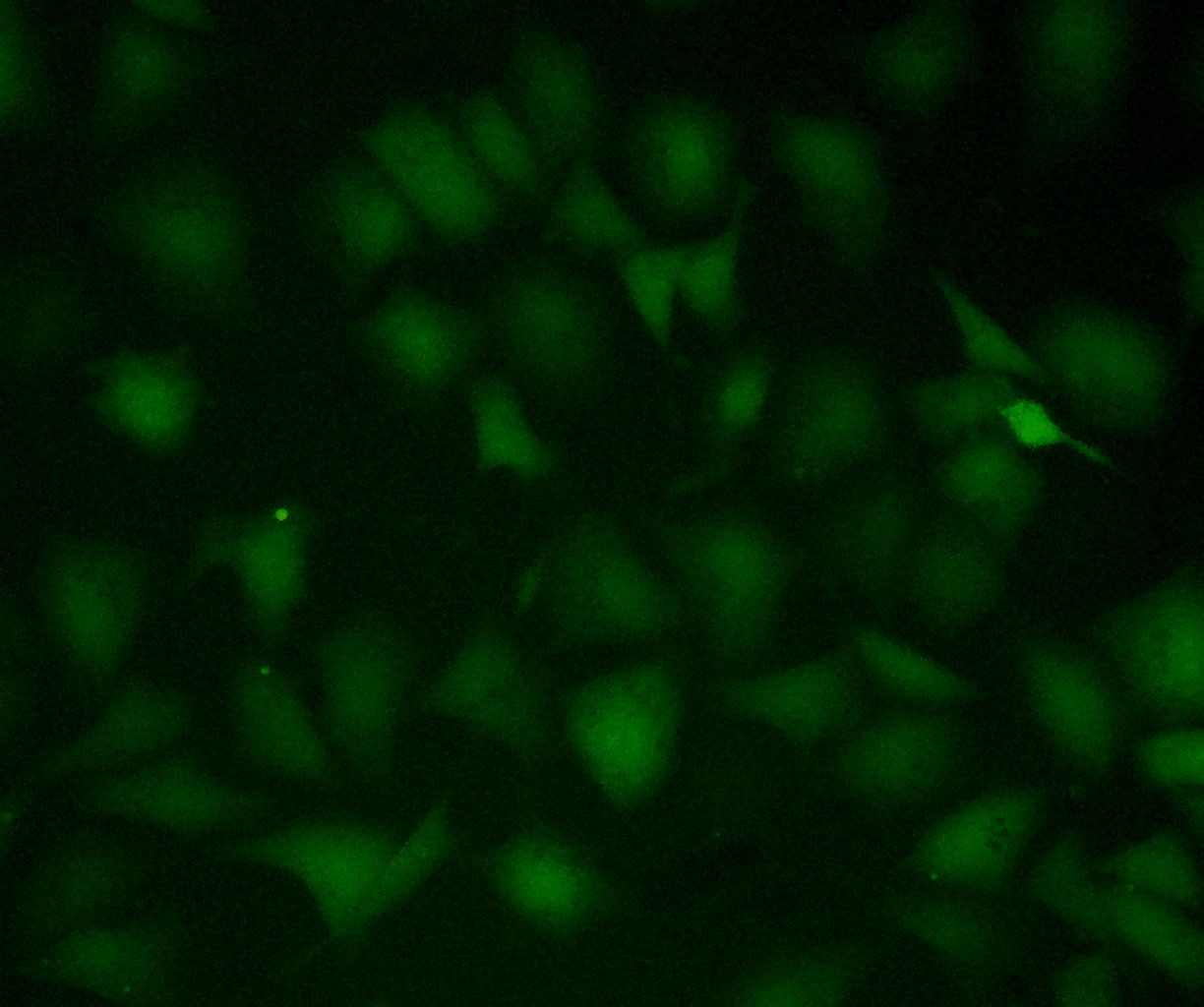

Supplement: Supplementary file 3 [file DataSheet_2.zip › ROS/Fig5/Control/2.jpg]

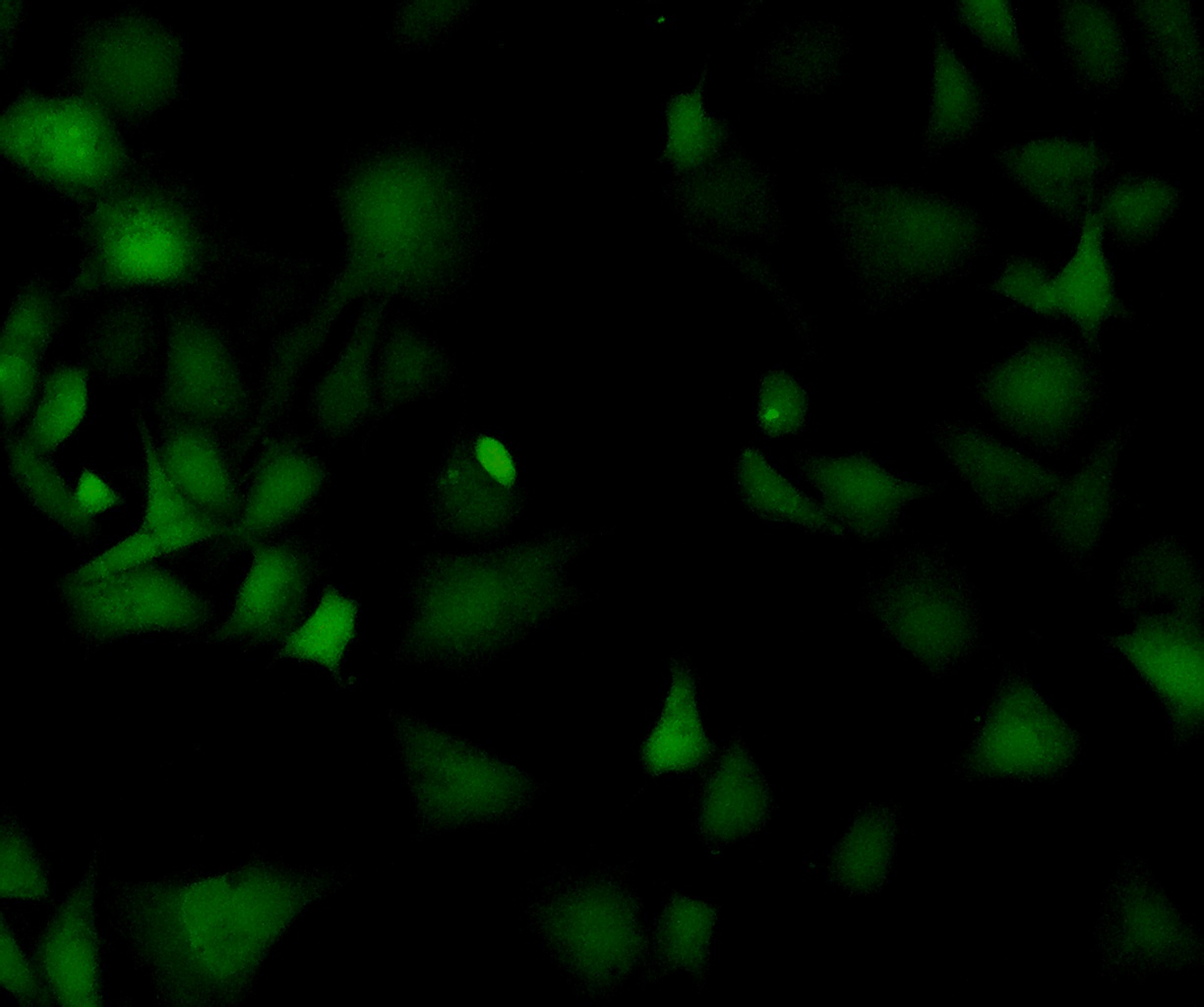

Supplement: Supplementary file 3 [file DataSheet_2.zip › ROS/Fig5/Control/3.jpg]

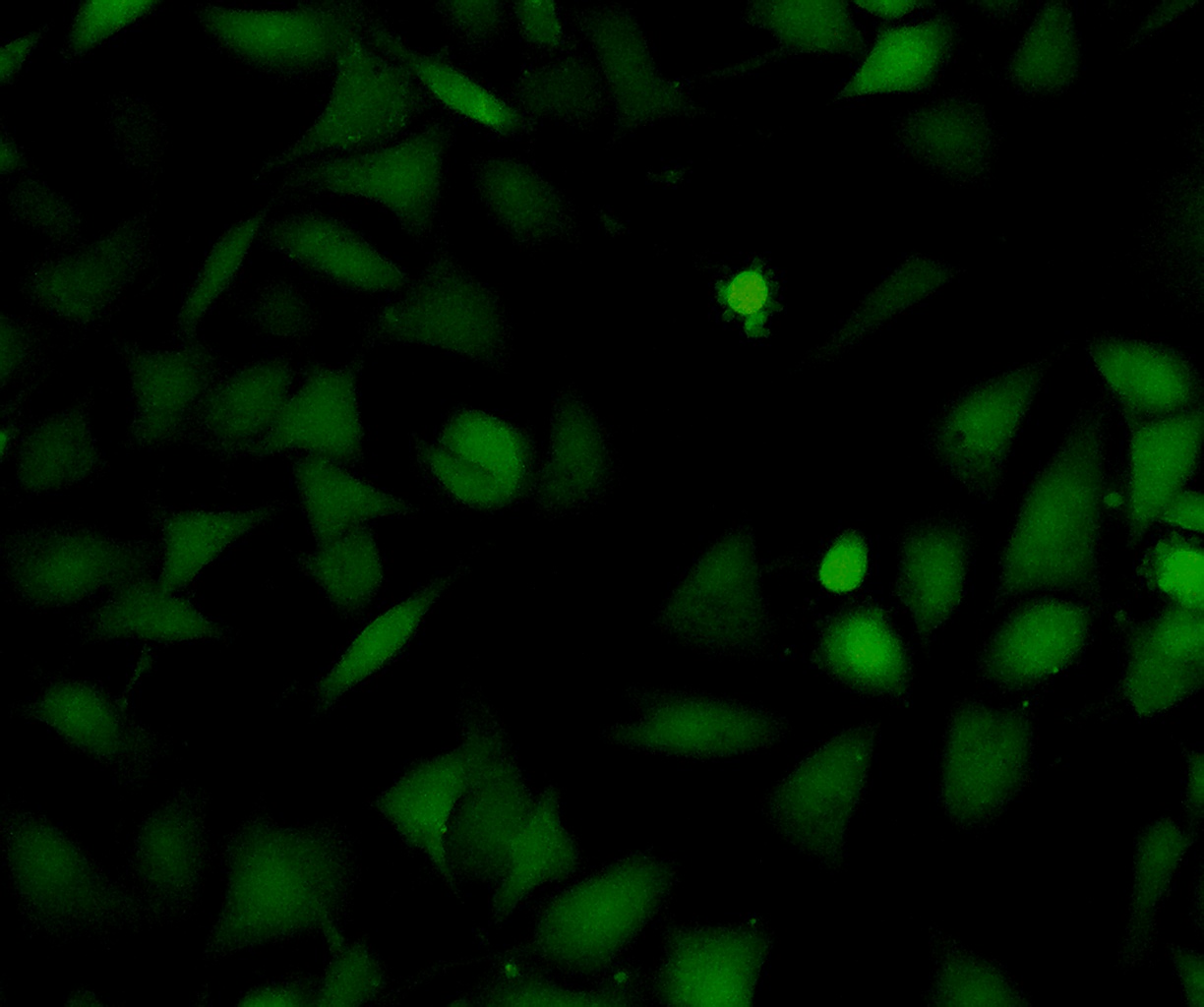

Supplement: Supplementary file 3 [file DataSheet_2.zip › ROS/Fig5/NC/1.jpg]

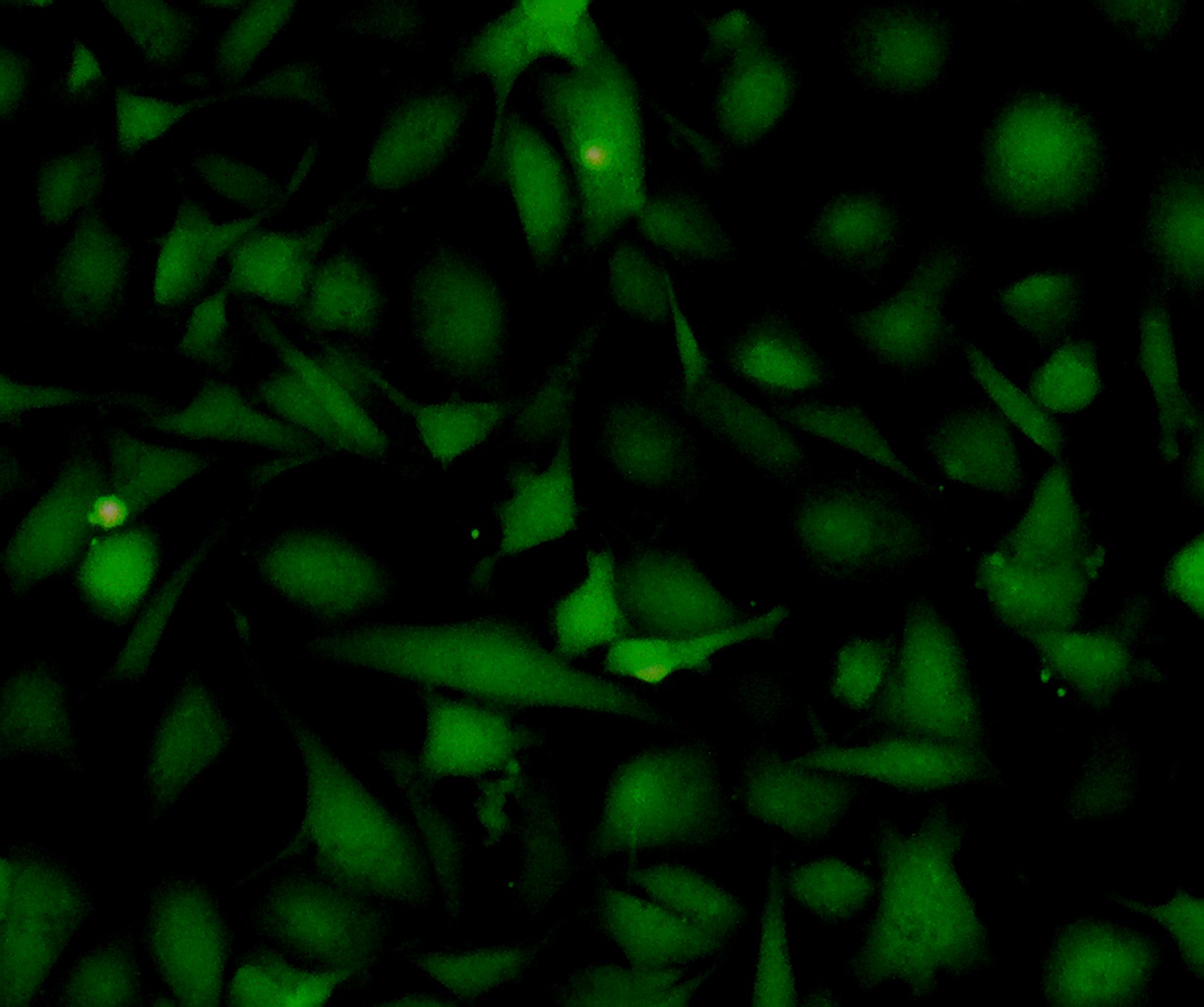

Supplement: Supplementary file 3 [file DataSheet_2.zip › ROS/Fig5/NC/2.jpg]

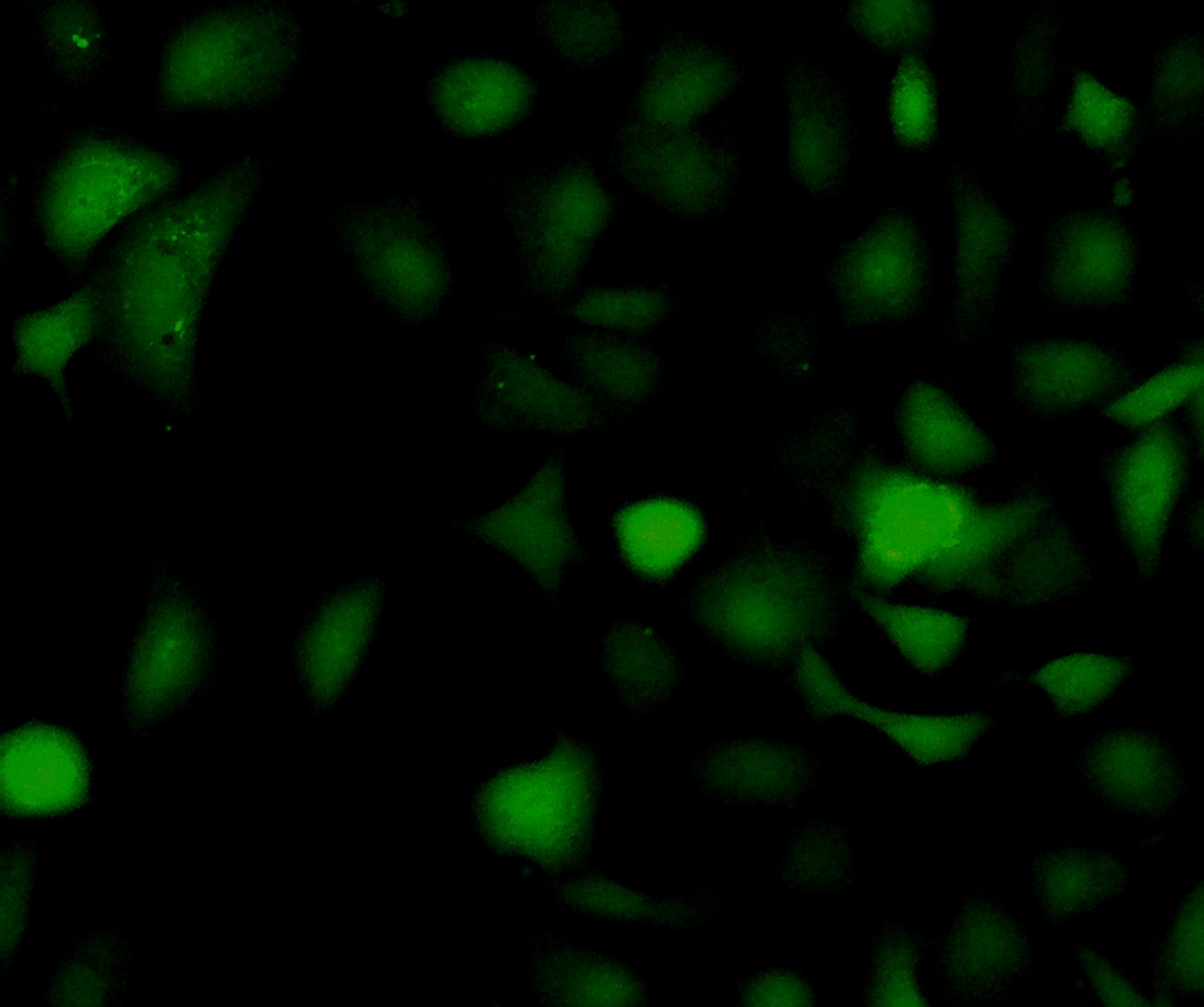

Supplement: Supplementary file 3 [file DataSheet_2.zip › ROS/Fig5/NC/3.jpg]

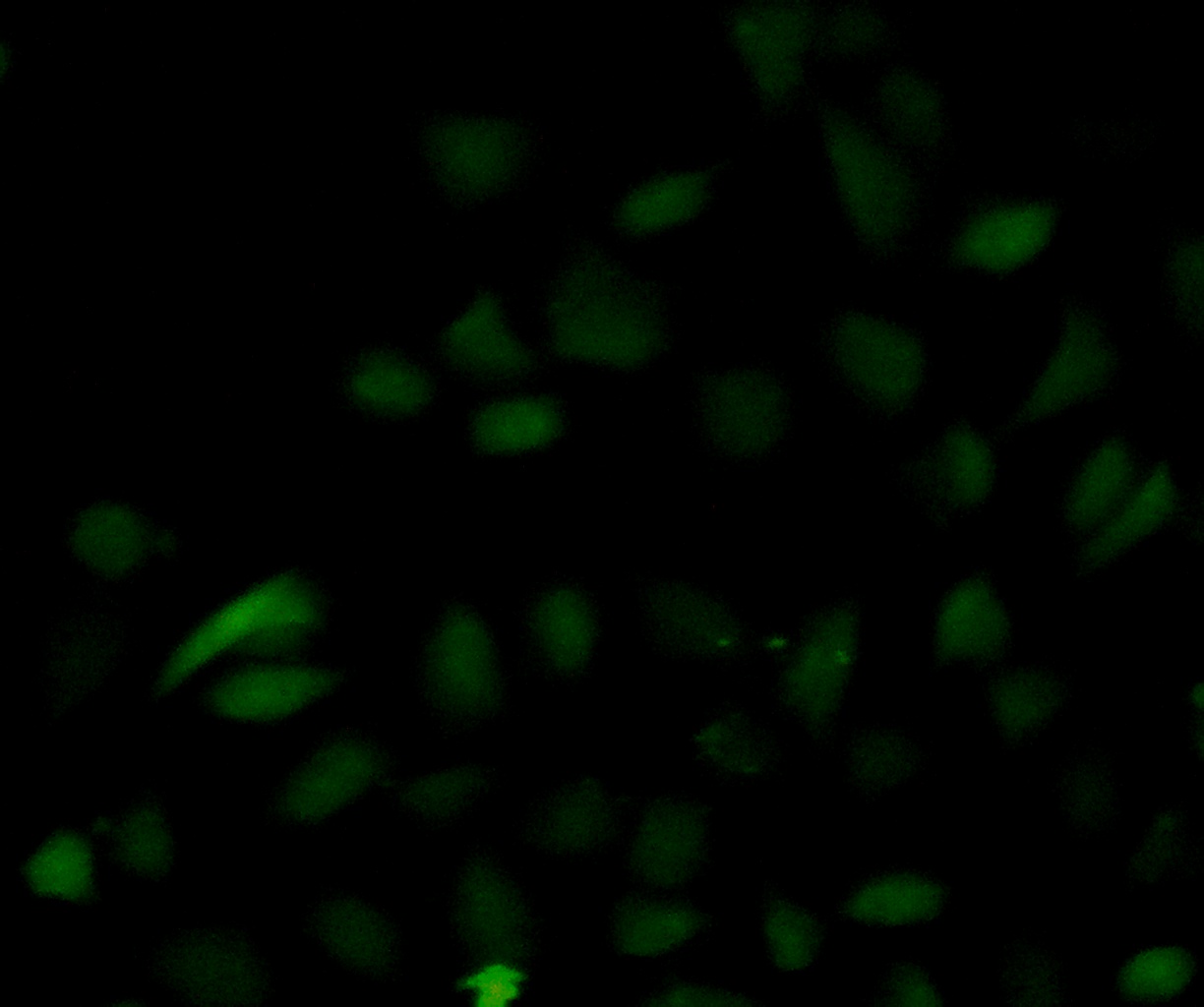

Supplement: Supplementary file 3 [file DataSheet_2.zip › ROS/Fig5/SLC7A11(+)/1.jpg]

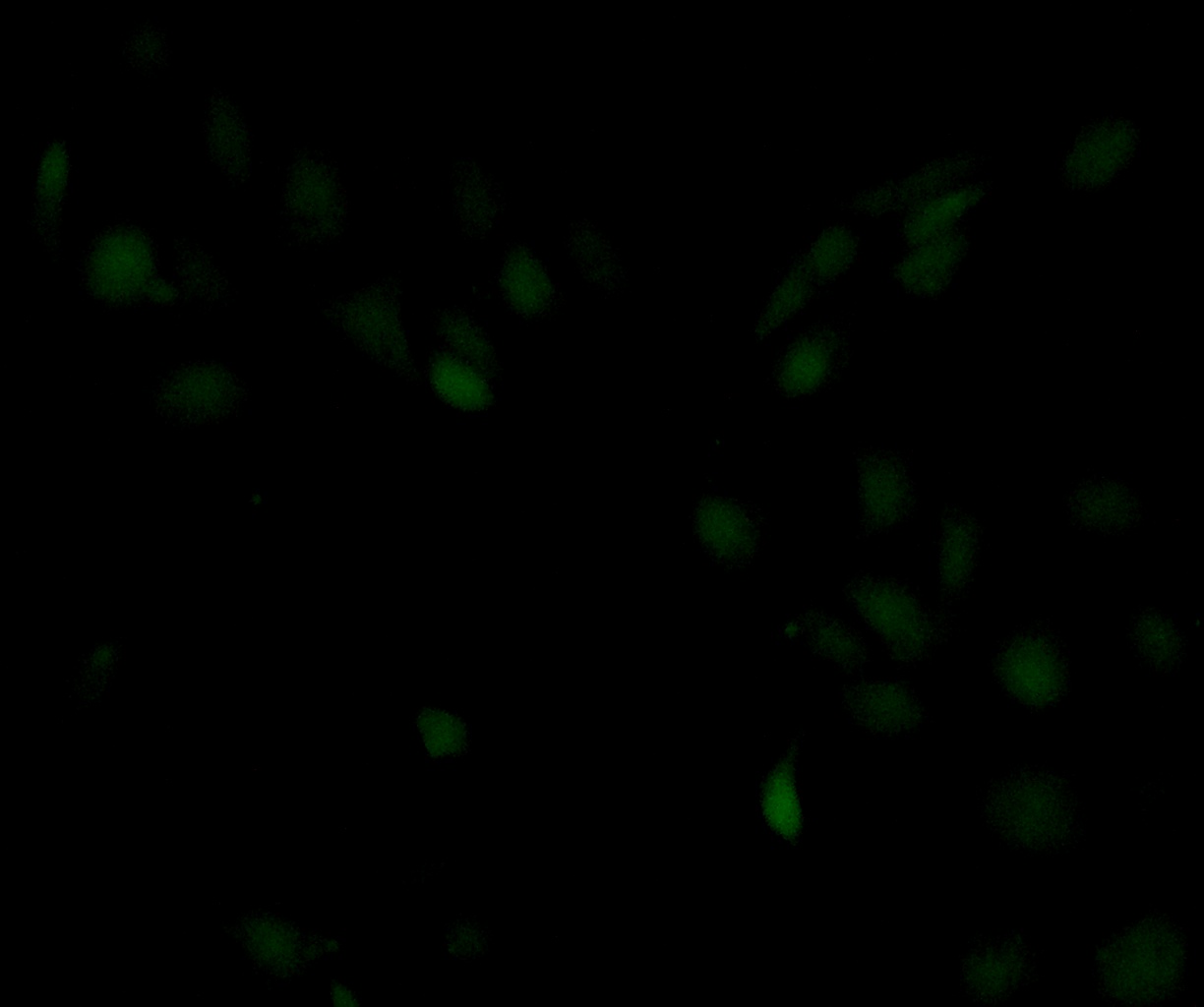

Supplement: Supplementary file 3 [file DataSheet_2.zip › ROS/Fig5/SLC7A11(+)/2.jpg]

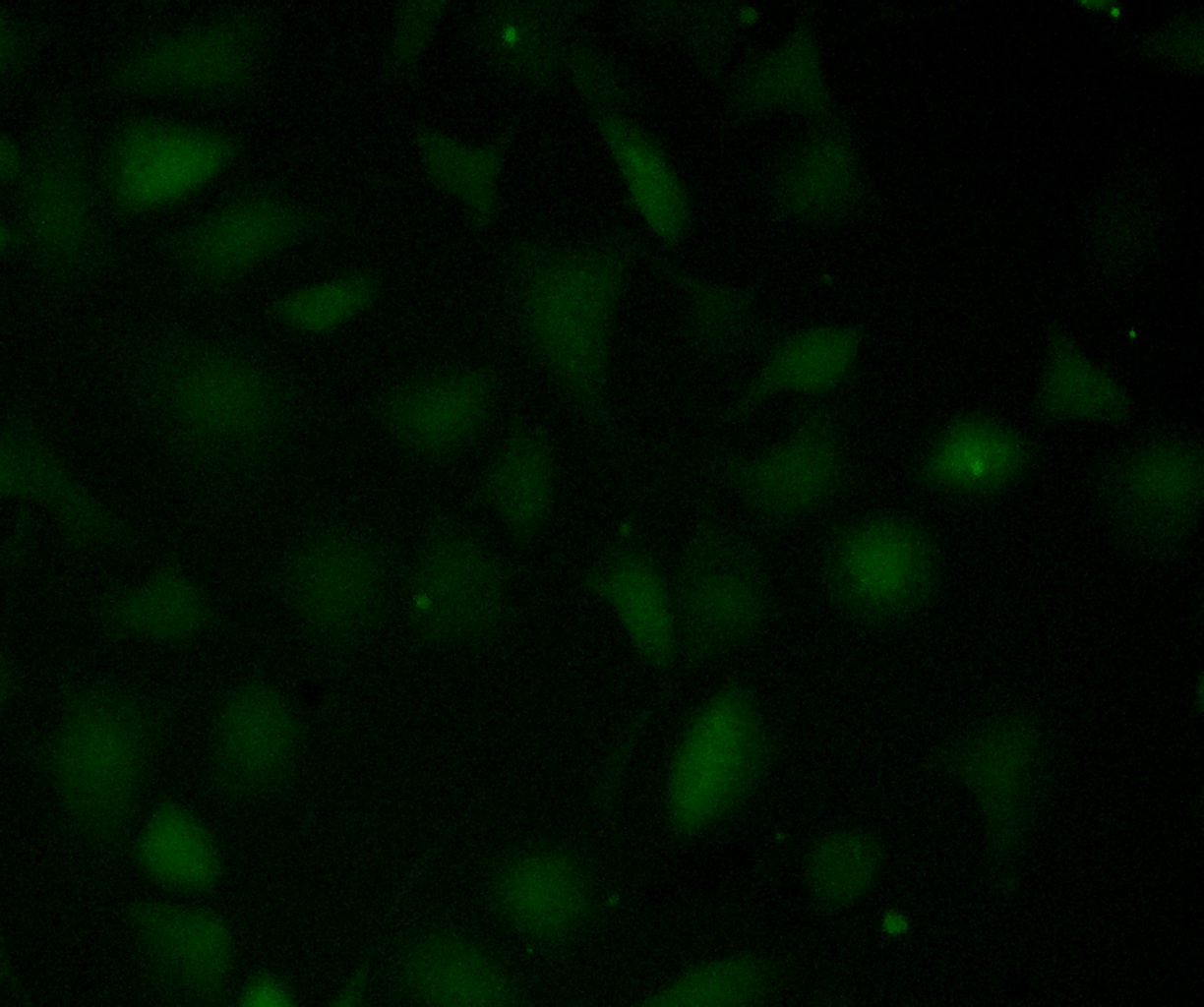

Supplement: Supplementary file 3 [file DataSheet_2.zip › ROS/Fig5/SLC7A11(+)/3.jpg]

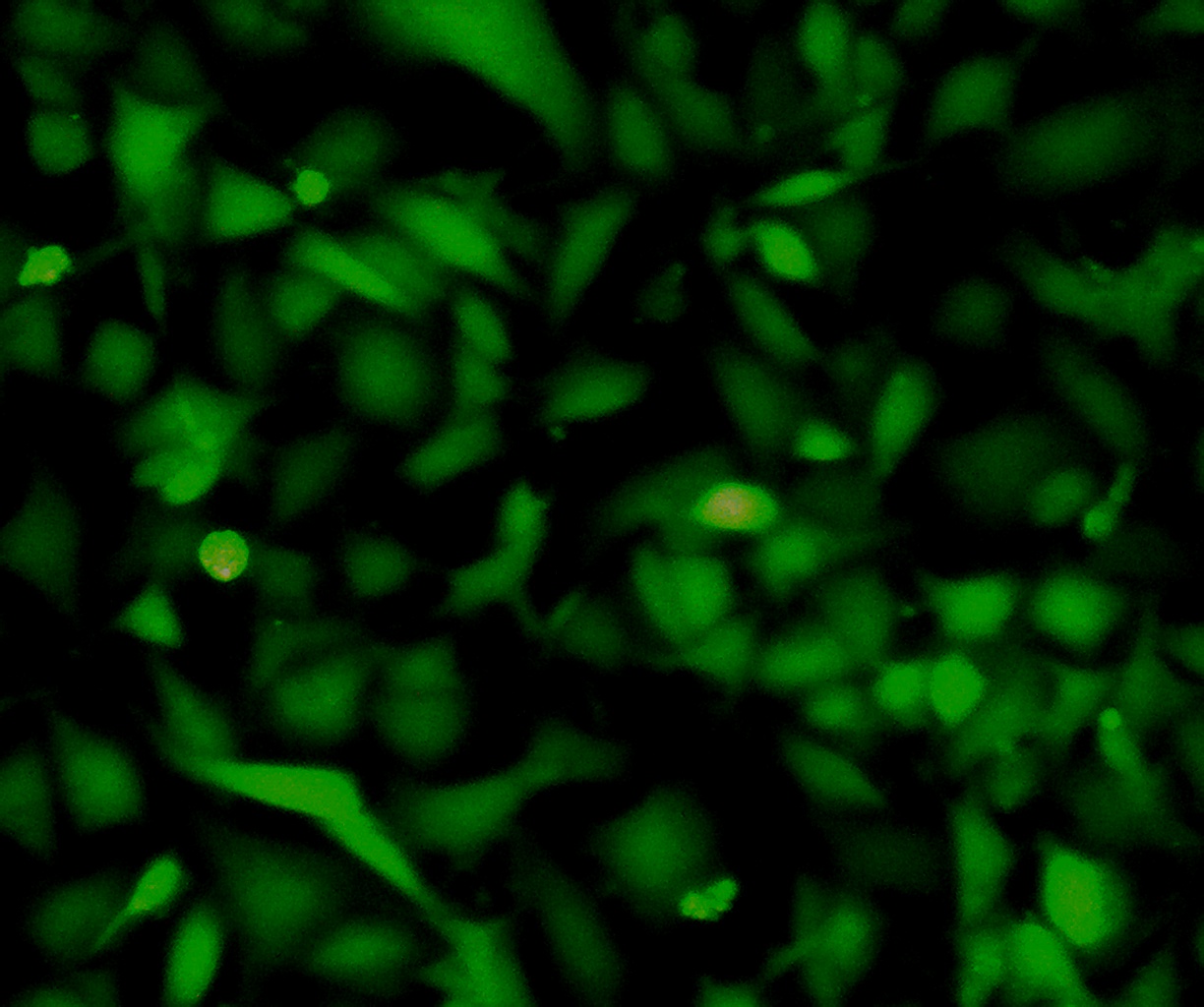

Supplement: Supplementary file 3 [file DataSheet_2.zip › ROS/Fig5/miR(+)/1.jpg]

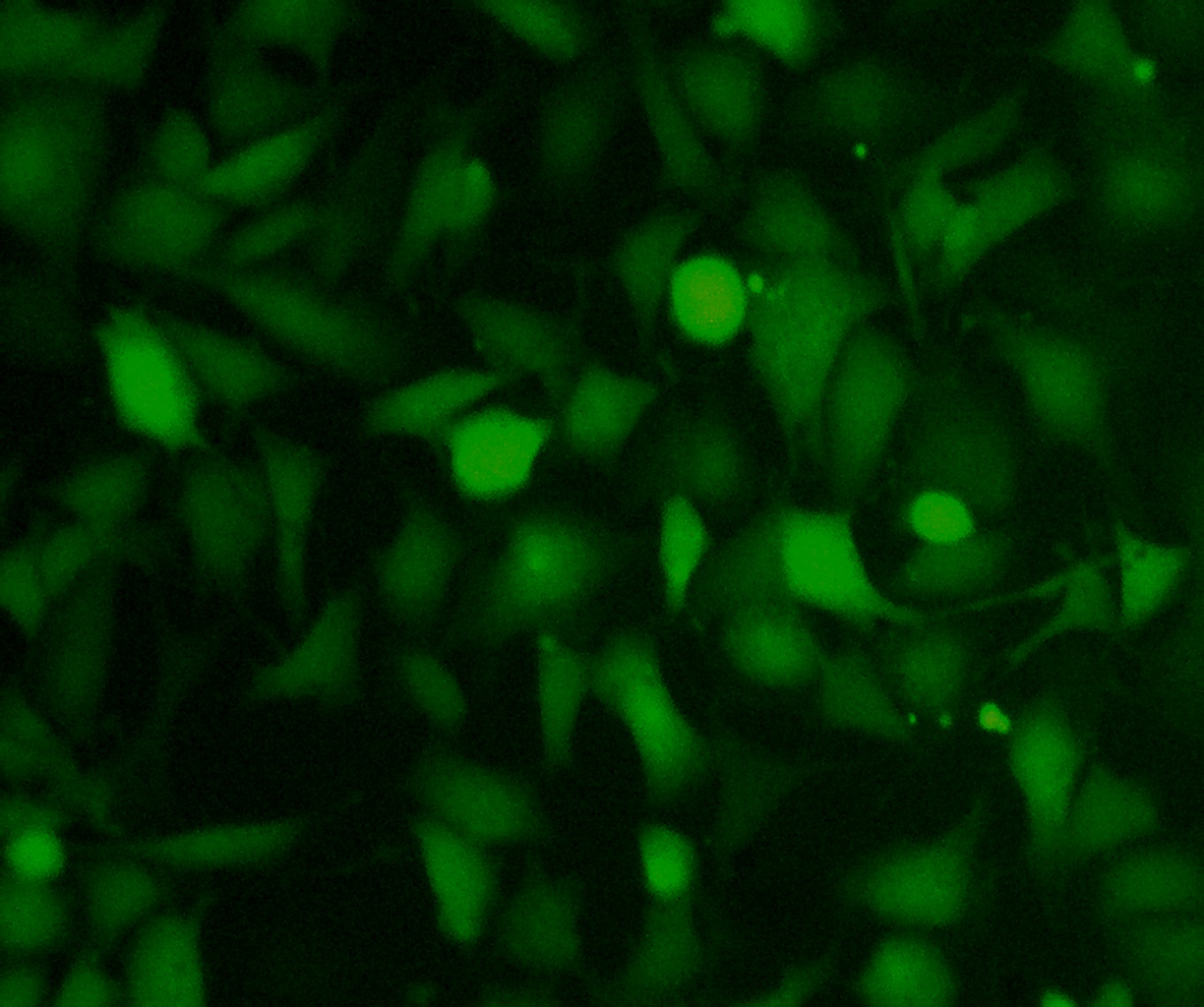

Supplement: Supplementary file 3 [file DataSheet_2.zip › ROS/Fig5/miR(+)/2.jpg]

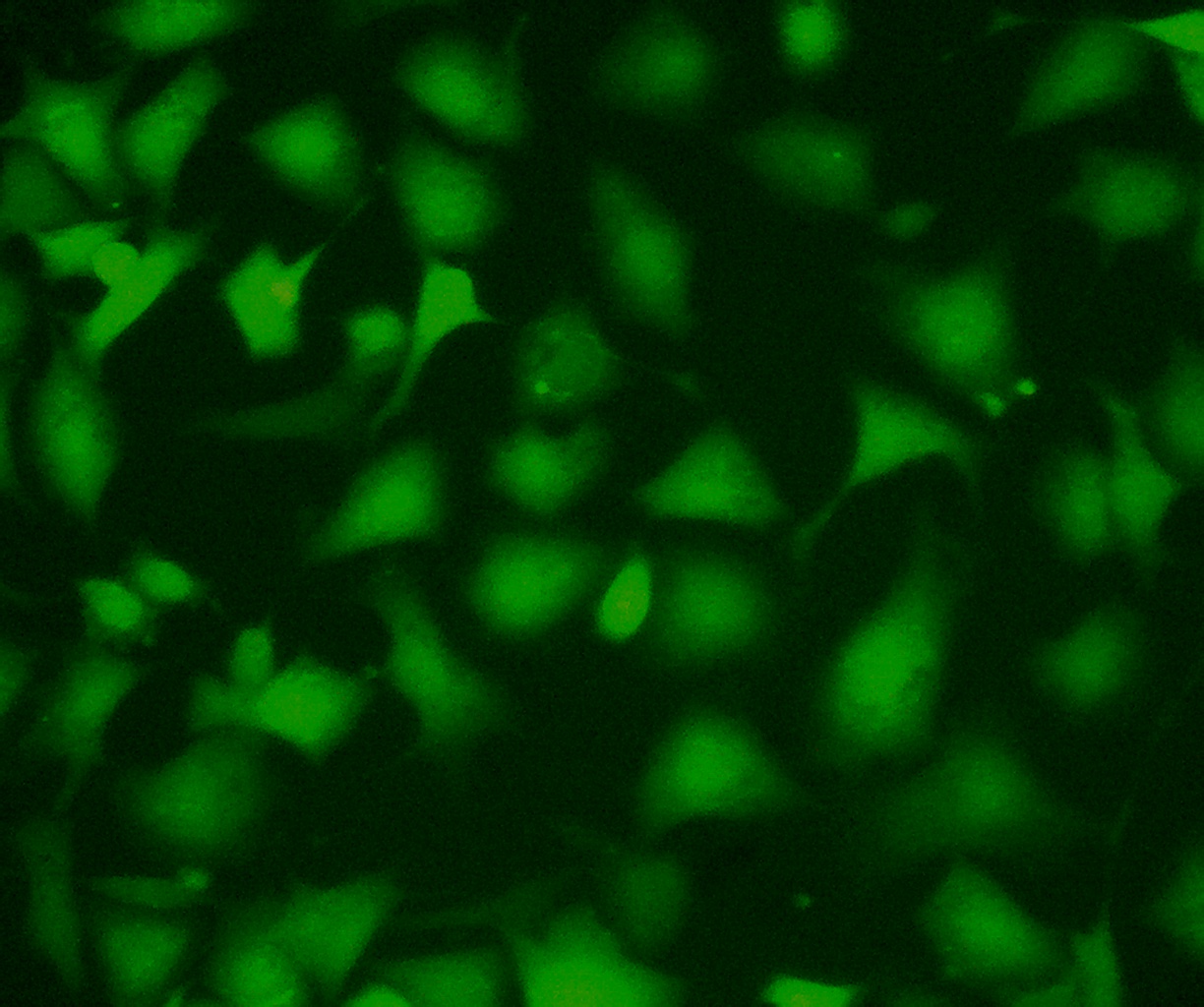

Supplement: Supplementary file 3 [file DataSheet_2.zip › ROS/Fig5/miR(+)/3.jpg]

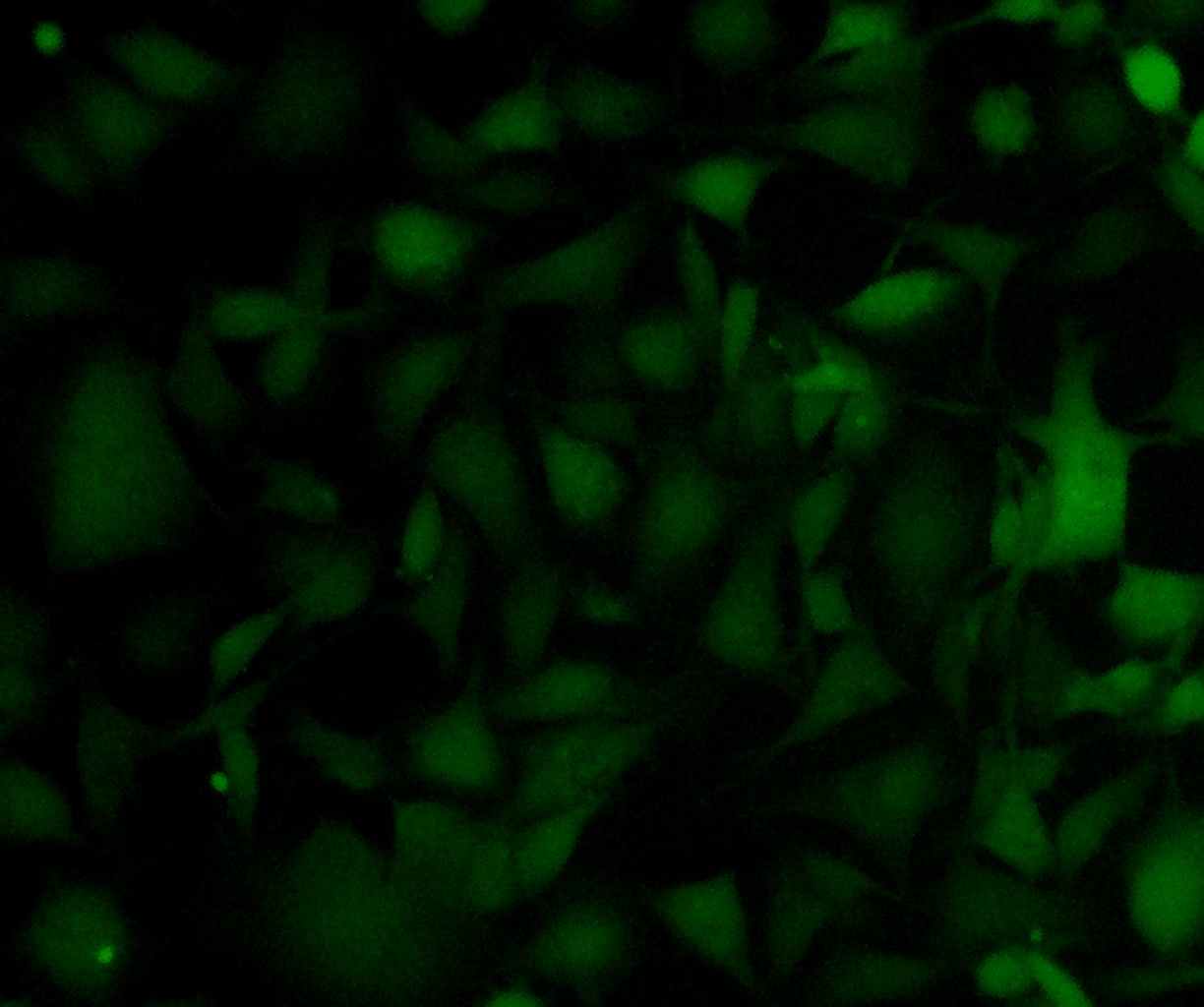

Supplement: Supplementary file 3 [file DataSheet_2.zip › ROS/Fig5/miR(+)+SLC7A11(+)/1.jpg]

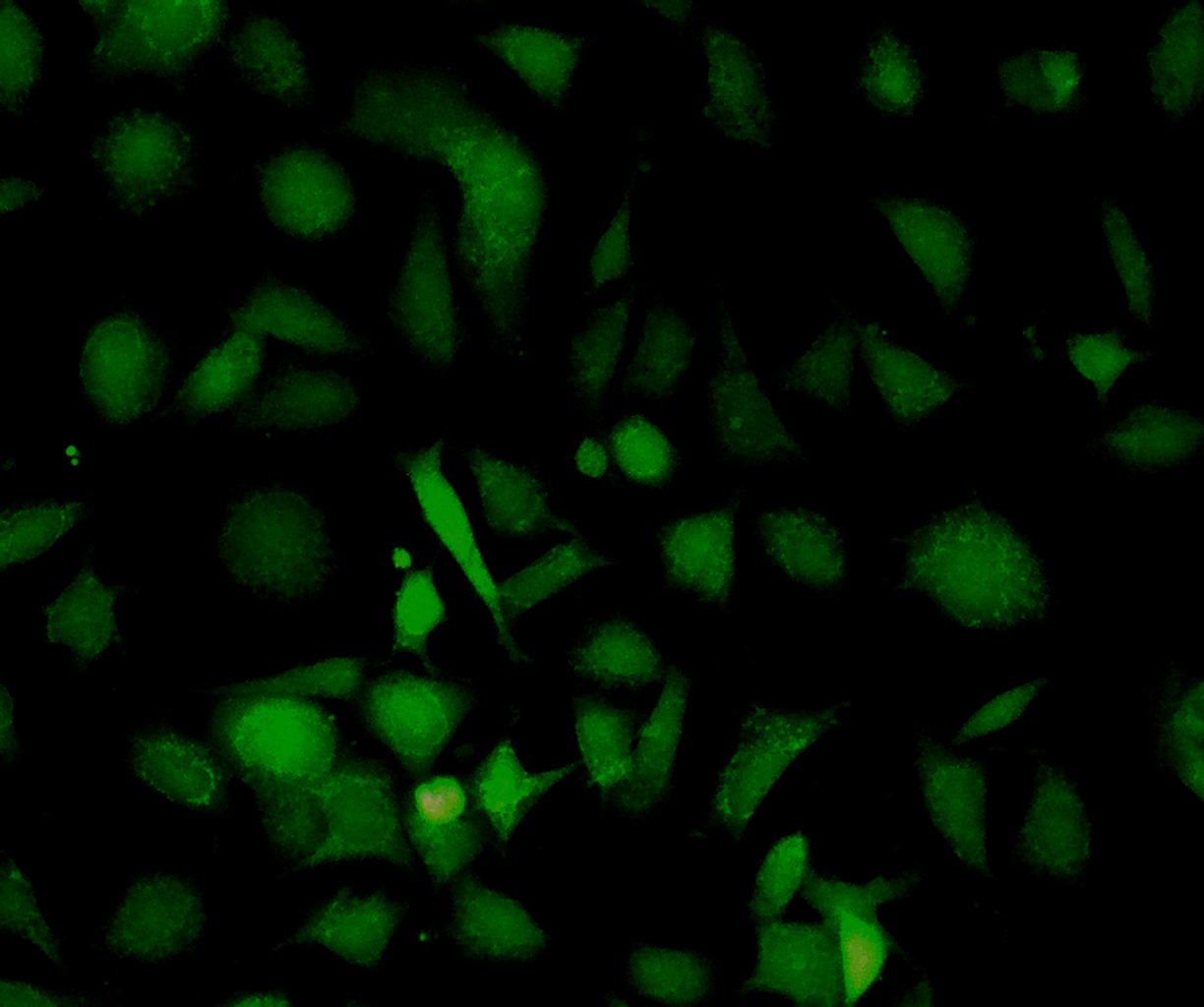

Supplement: Supplementary file 3 [file DataSheet_2.zip › ROS/Fig5/miR(+)+SLC7A11(+)/2.jpg]

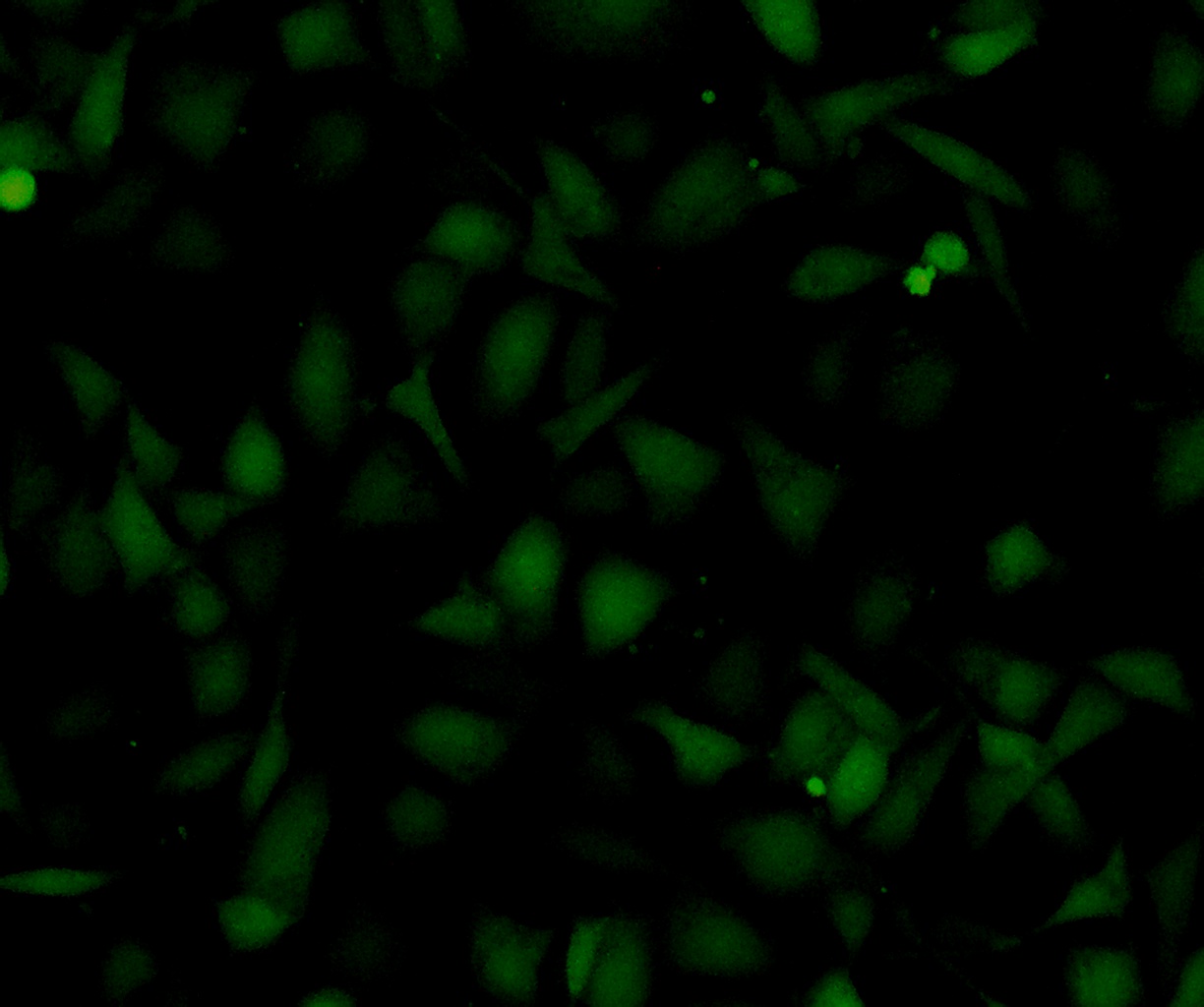

Supplement: Supplementary file 3 [file DataSheet_2.zip › ROS/Fig5/miR(+)+SLC7A11(+)/3.jpg]

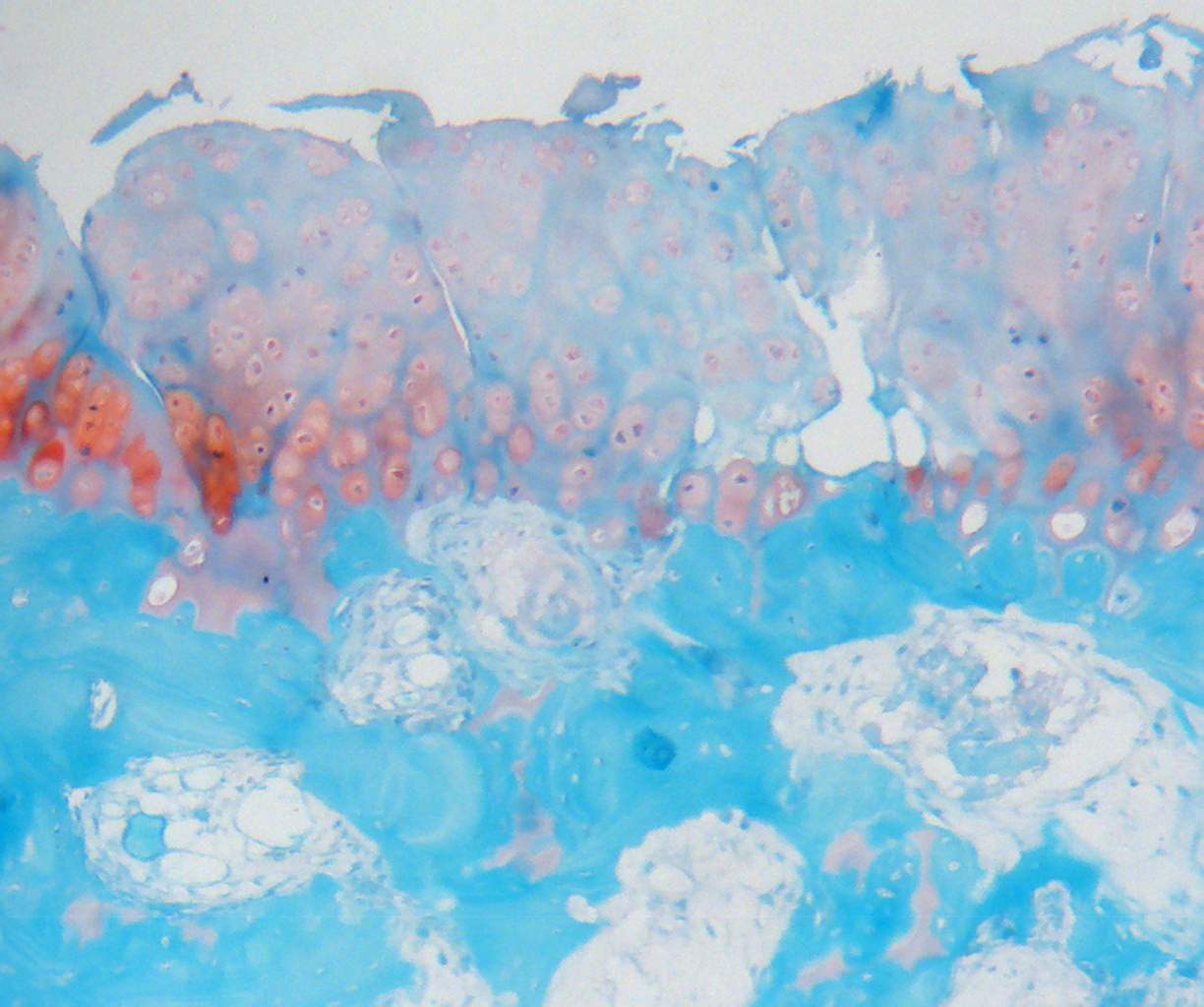

Supplement: Supplementary file 3 [file DataSheet_2.zip › SOFA/Exo/1.jpg]

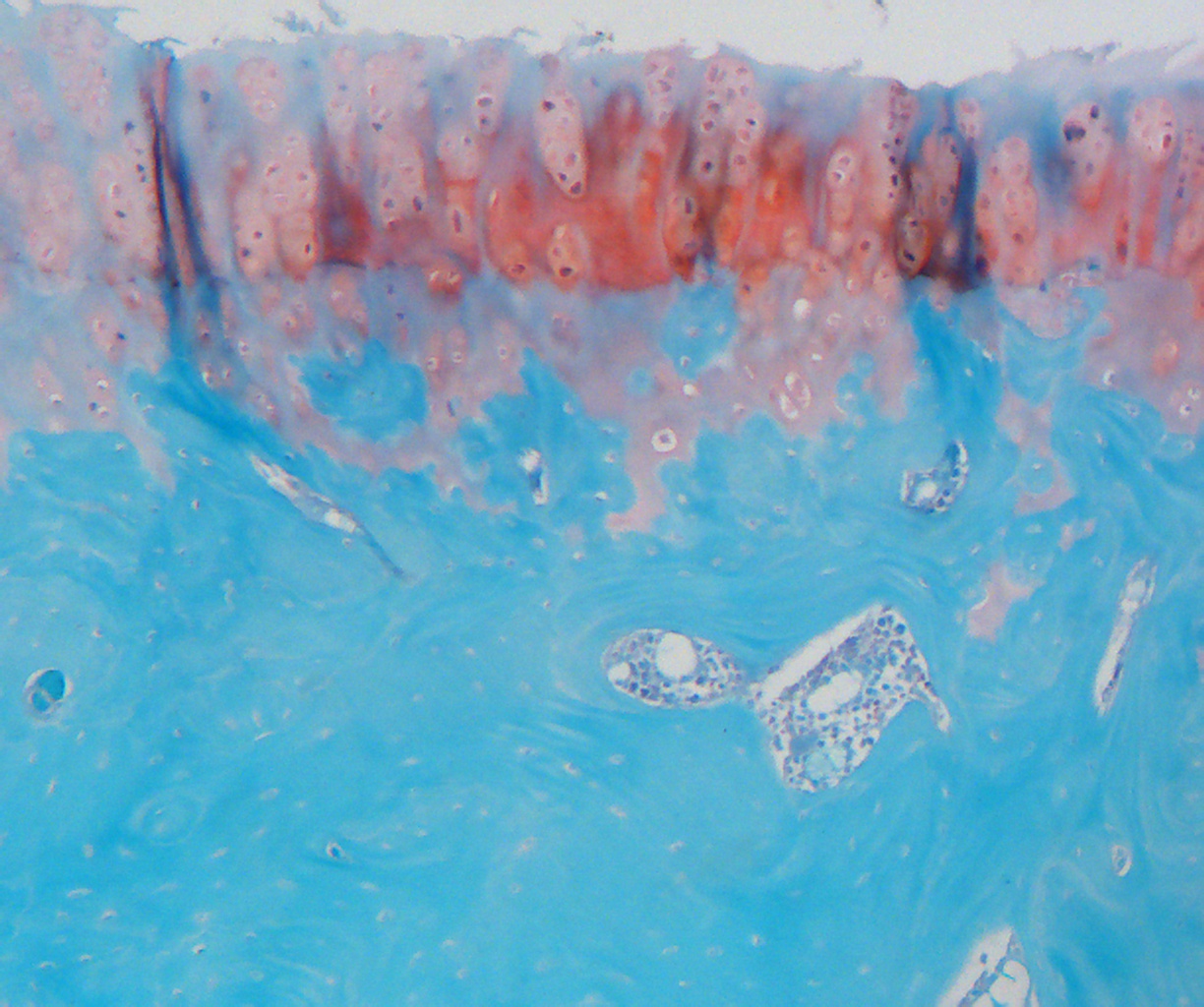

Supplement: Supplementary file 3 [file DataSheet_2.zip › SOFA/Exo/2.jpg]

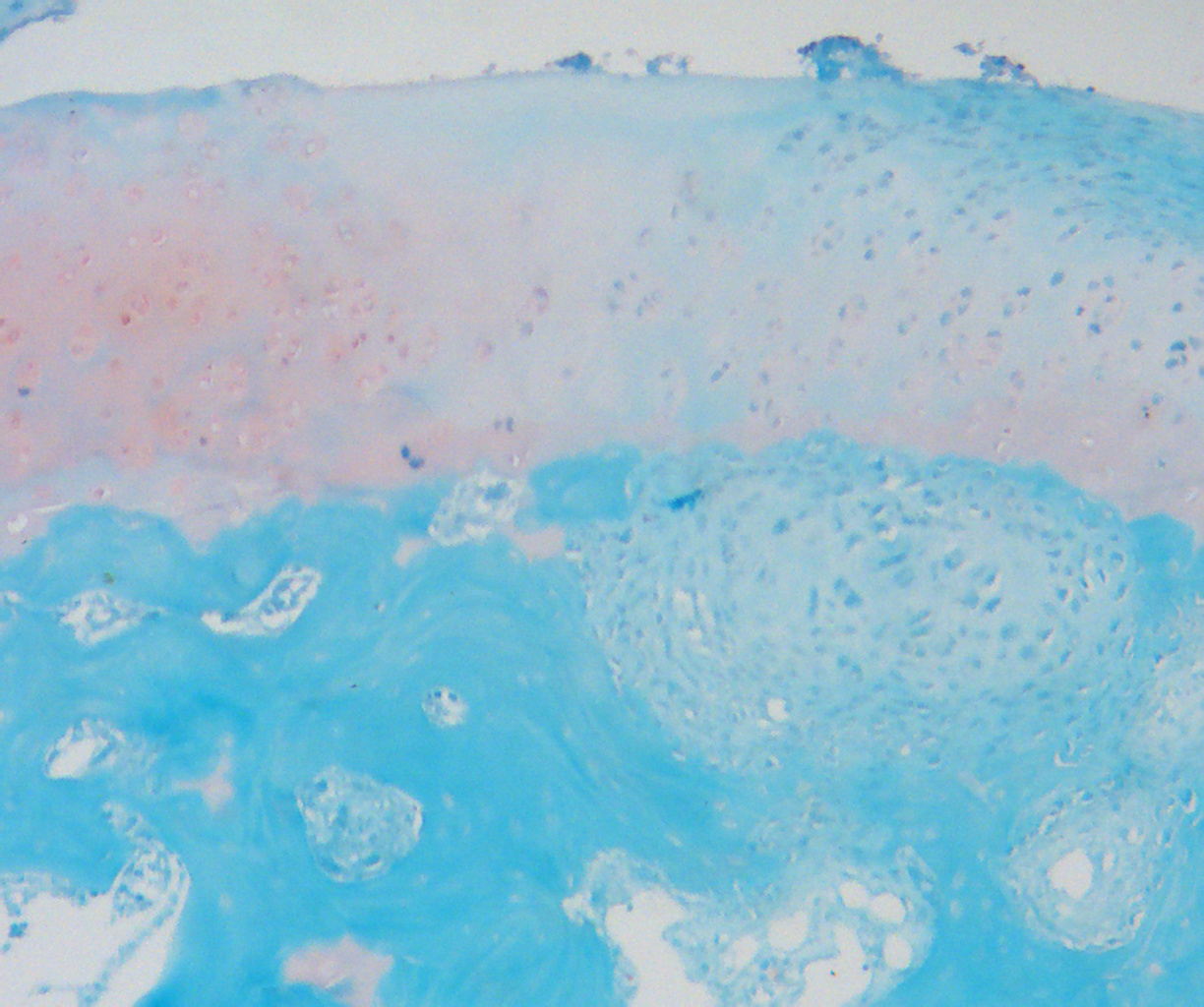

Supplement: Supplementary file 3 [file DataSheet_2.zip › SOFA/Exo/3.jpg]

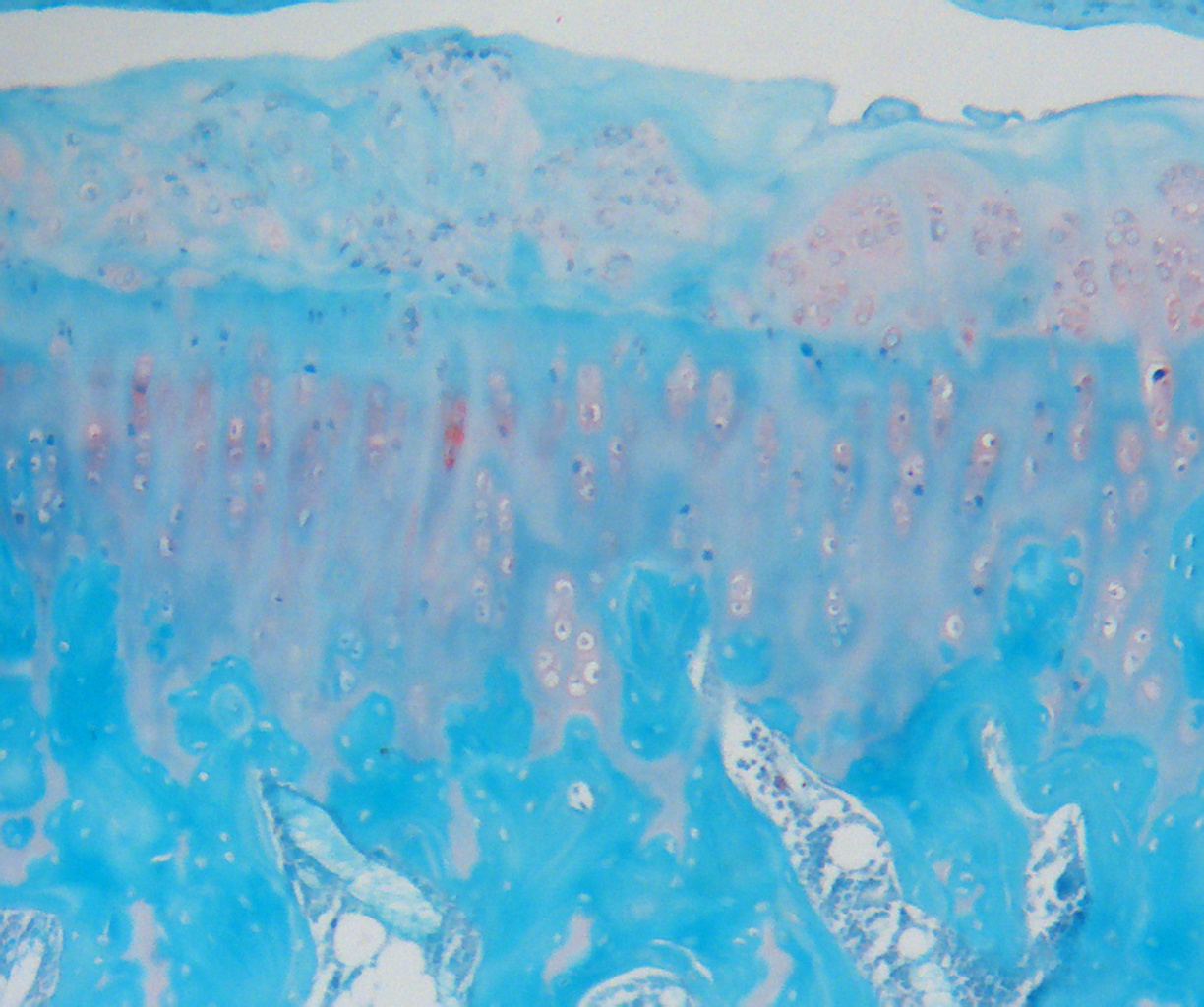

Supplement: Supplementary file 3 [file DataSheet_2.zip › SOFA/Exo/4.jpg]

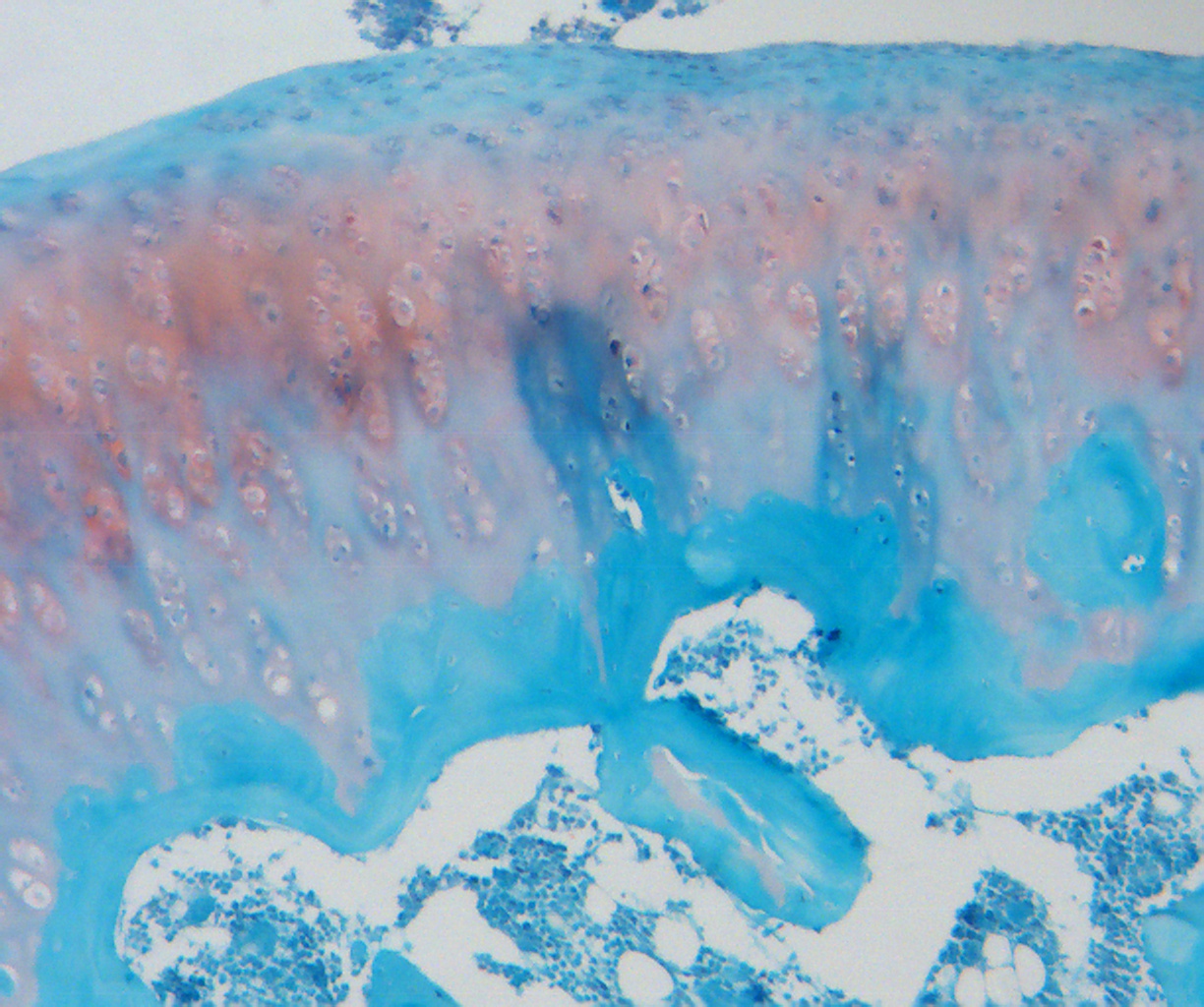

Supplement: Supplementary file 3 [file DataSheet_2.zip › SOFA/Exo/5.jpg]

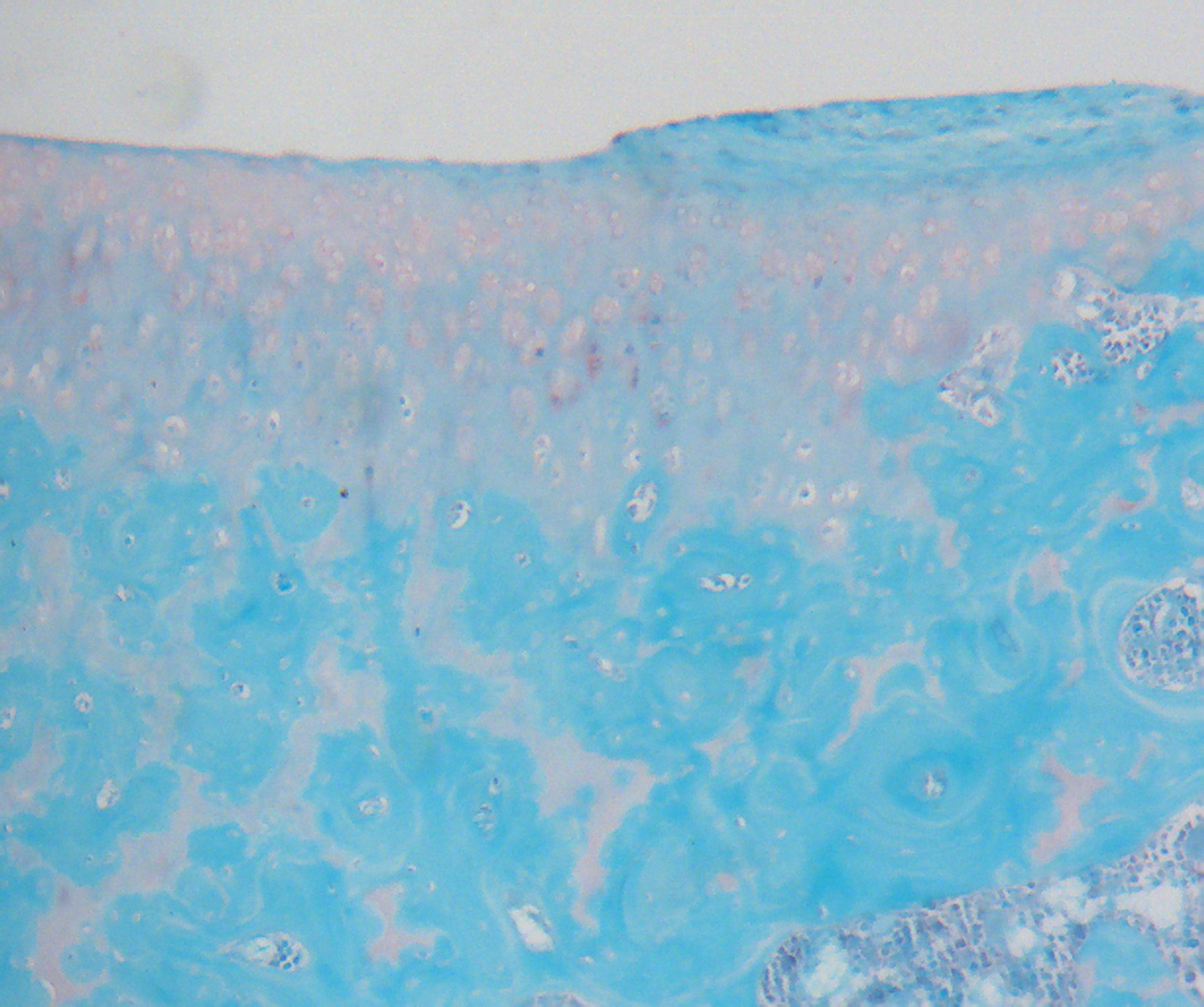

Supplement: Supplementary file 3 [file DataSheet_2.zip › SOFA/Exo/6.jpg]

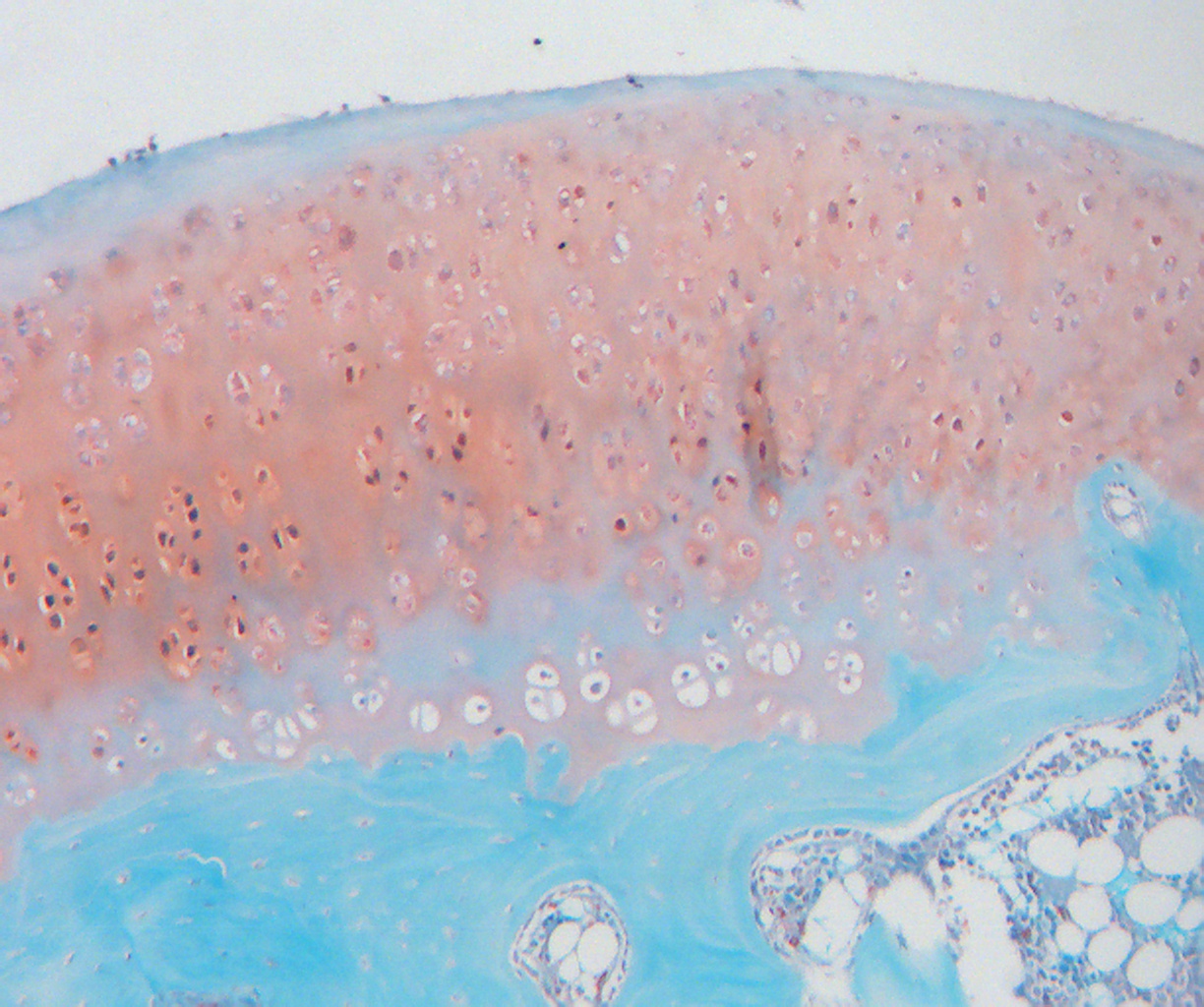

Supplement: Supplementary file 3 [file DataSheet_2.zip › SOFA/Exo+Fer-1/1.jpg]

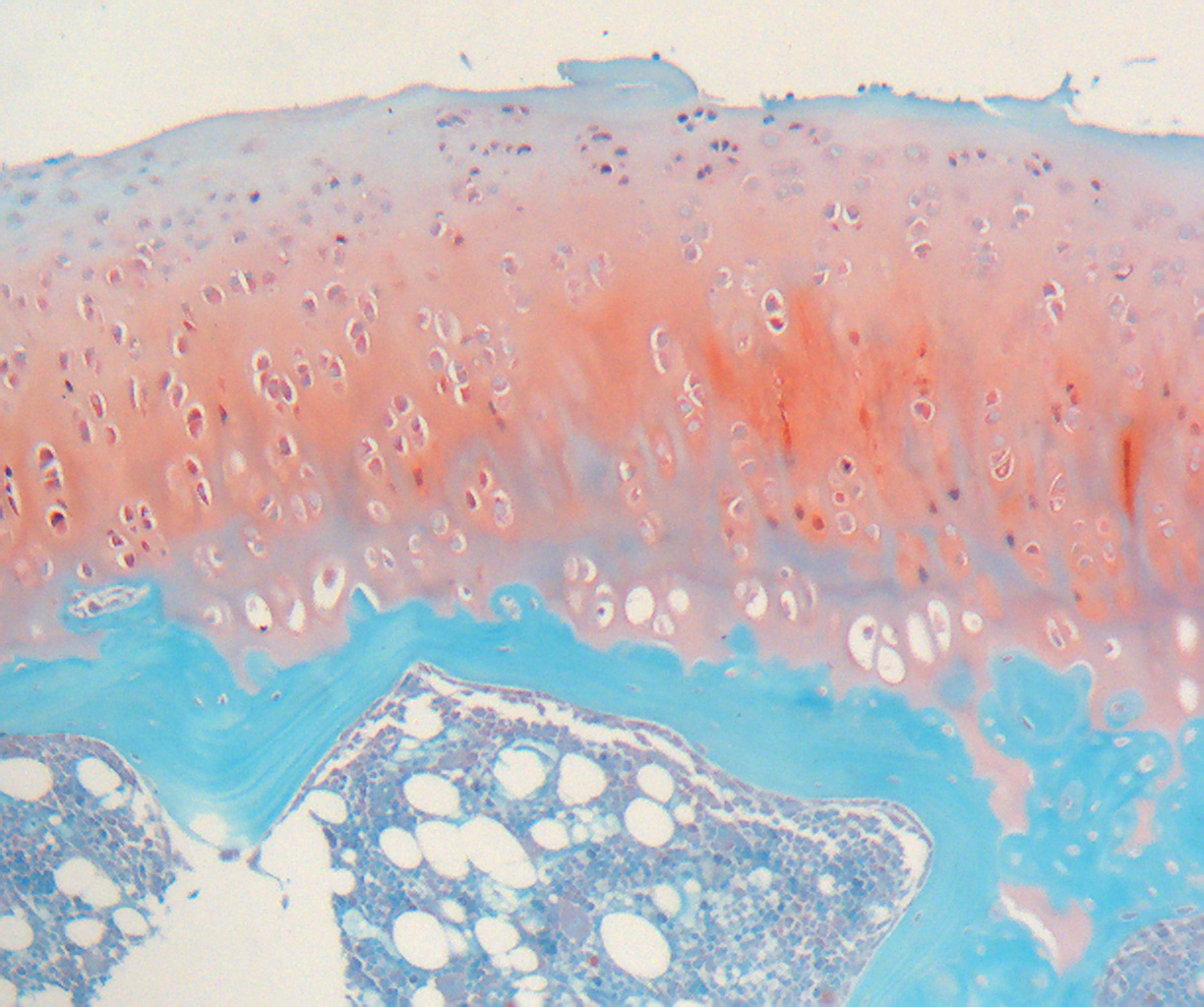

Supplement: Supplementary file 3 [file DataSheet_2.zip › SOFA/Exo+Fer-1/2.jpg]

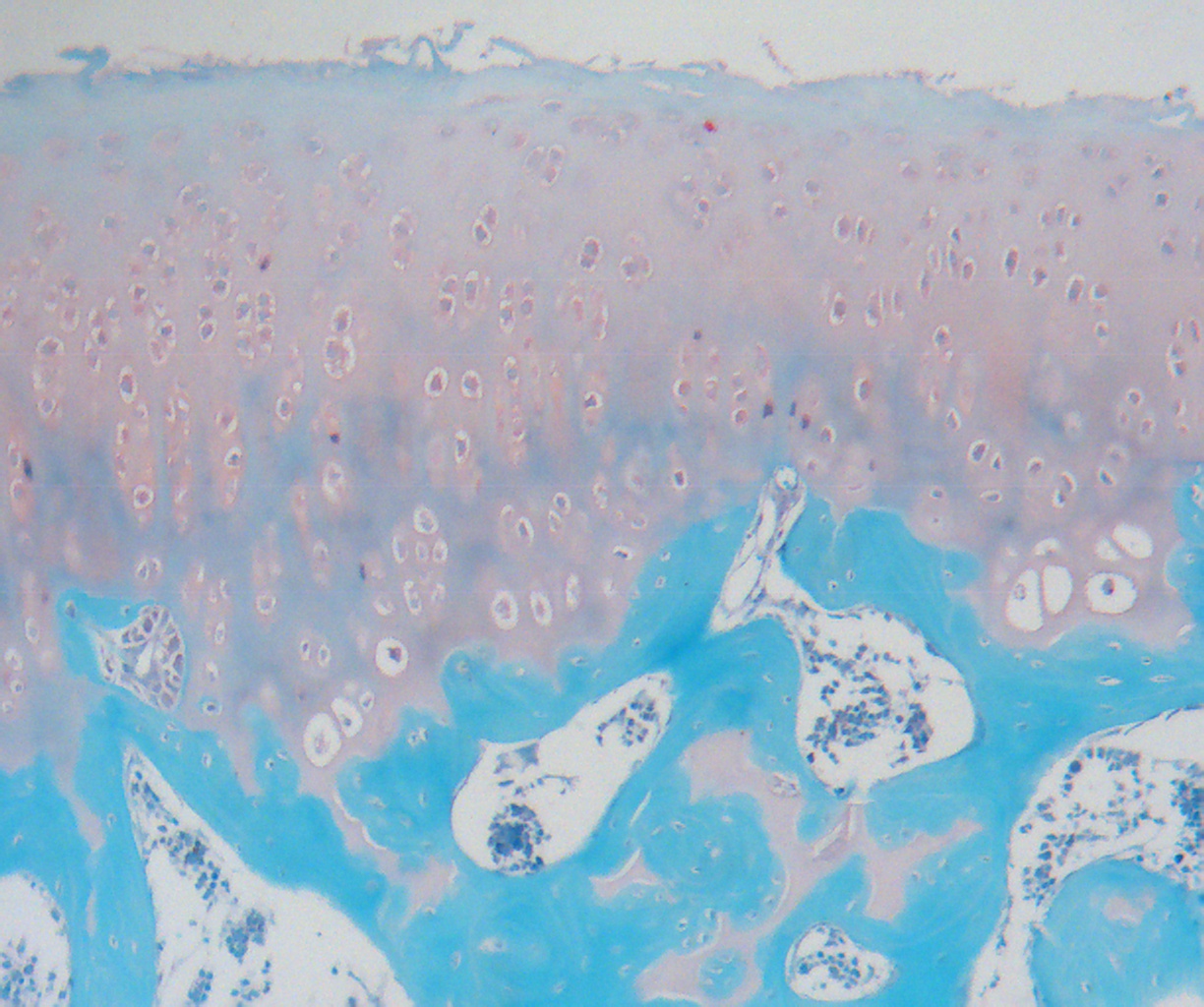

Supplement: Supplementary file 3 [file DataSheet_2.zip › SOFA/Exo+Fer-1/3.jpg]

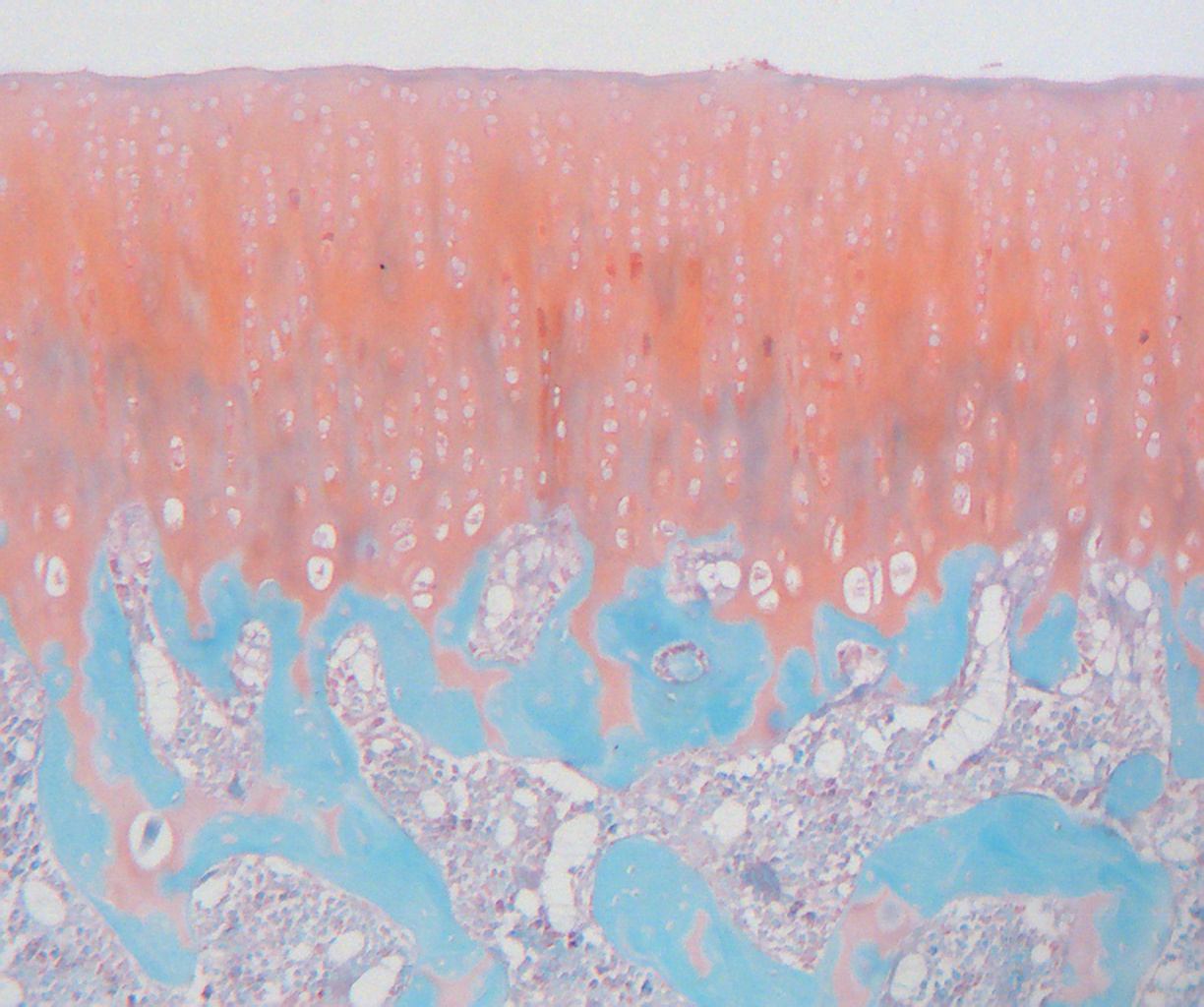

Supplement: Supplementary file 3 [file DataSheet_2.zip › SOFA/Exo+Fer-1/4.jpg]

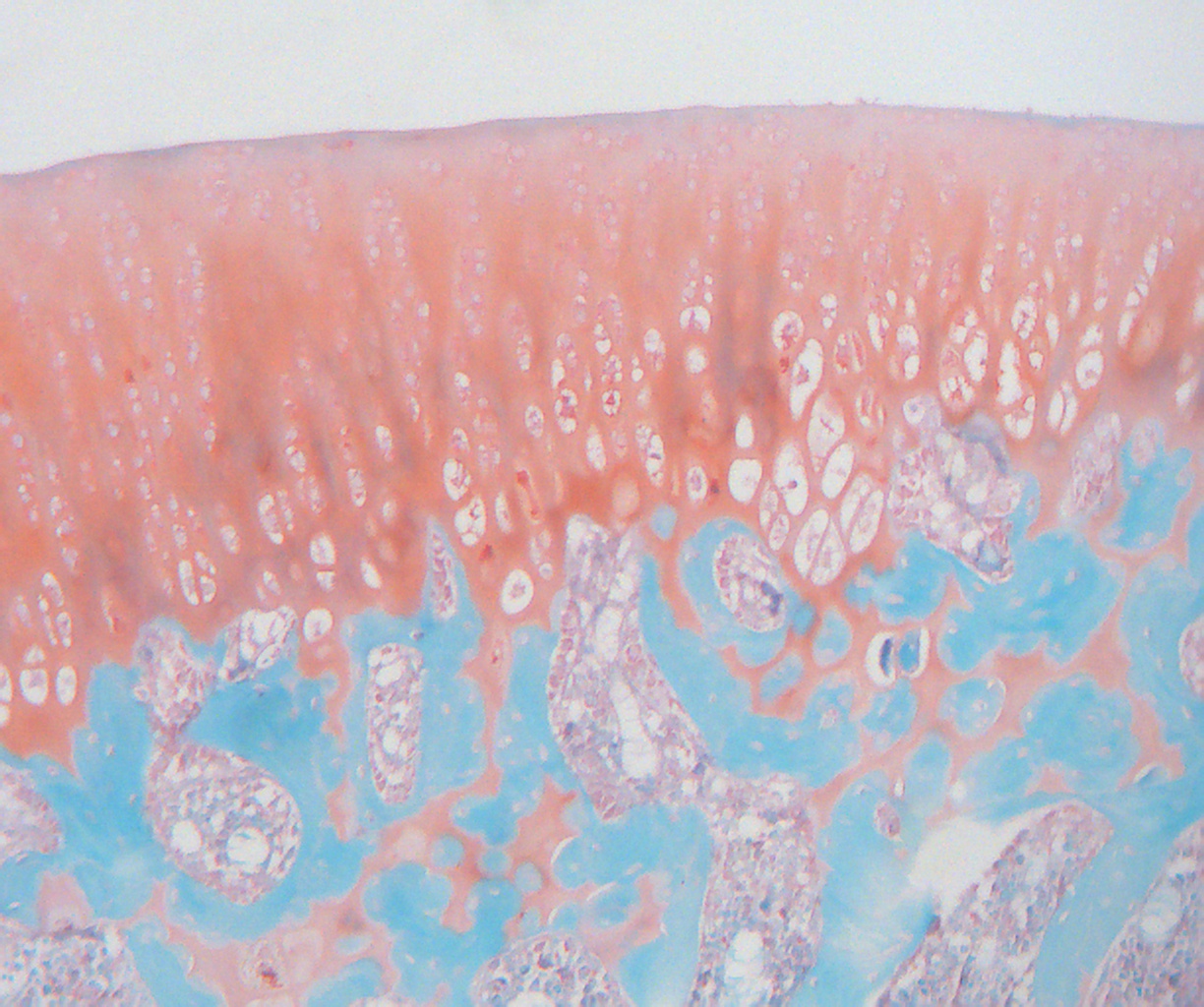

Supplement: Supplementary file 3 [file DataSheet_2.zip › SOFA/Exo+Fer-1/5.jpg]

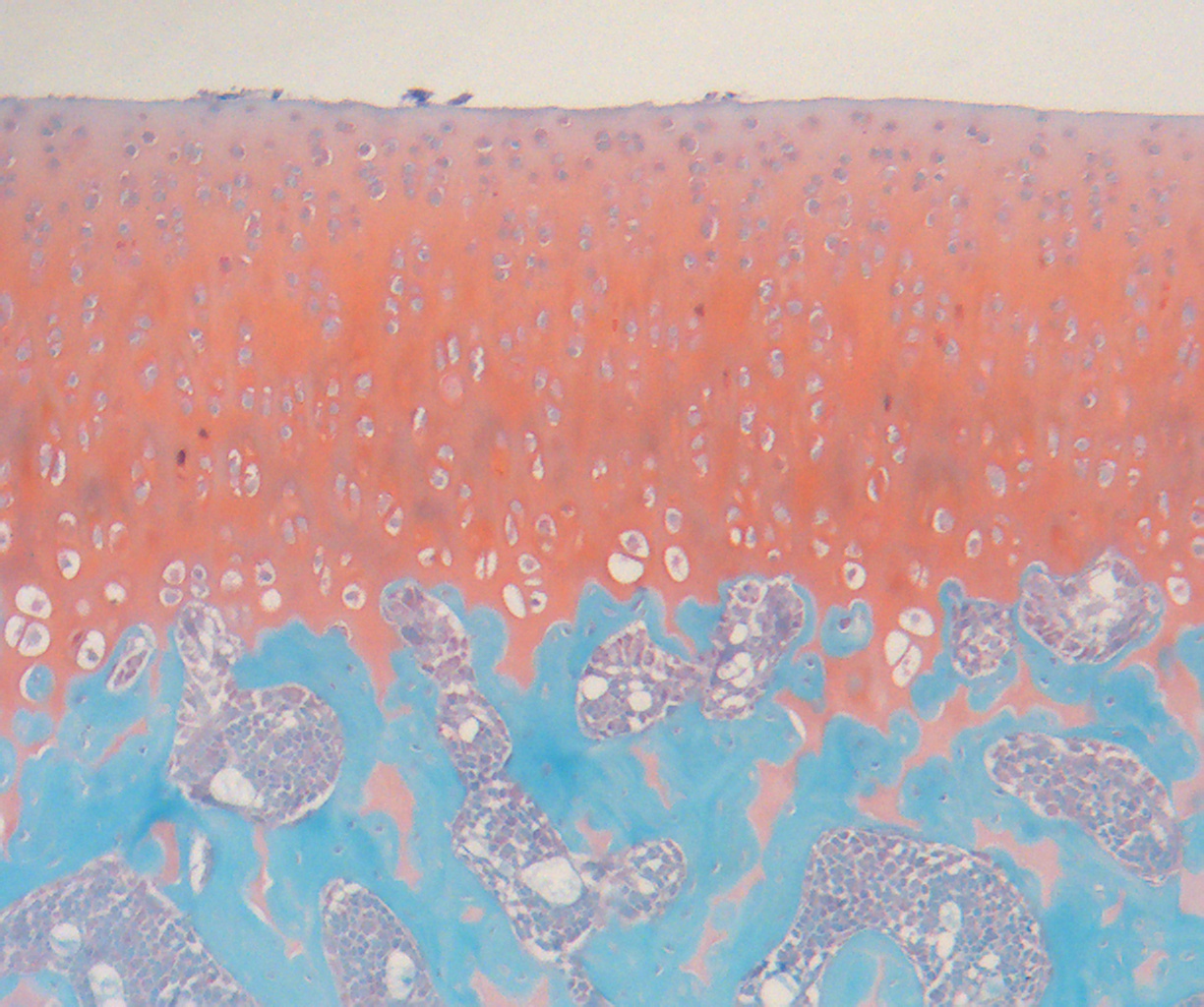

Supplement: Supplementary file 3 [file DataSheet_2.zip › SOFA/Exo+Fer-1/6.jpg]

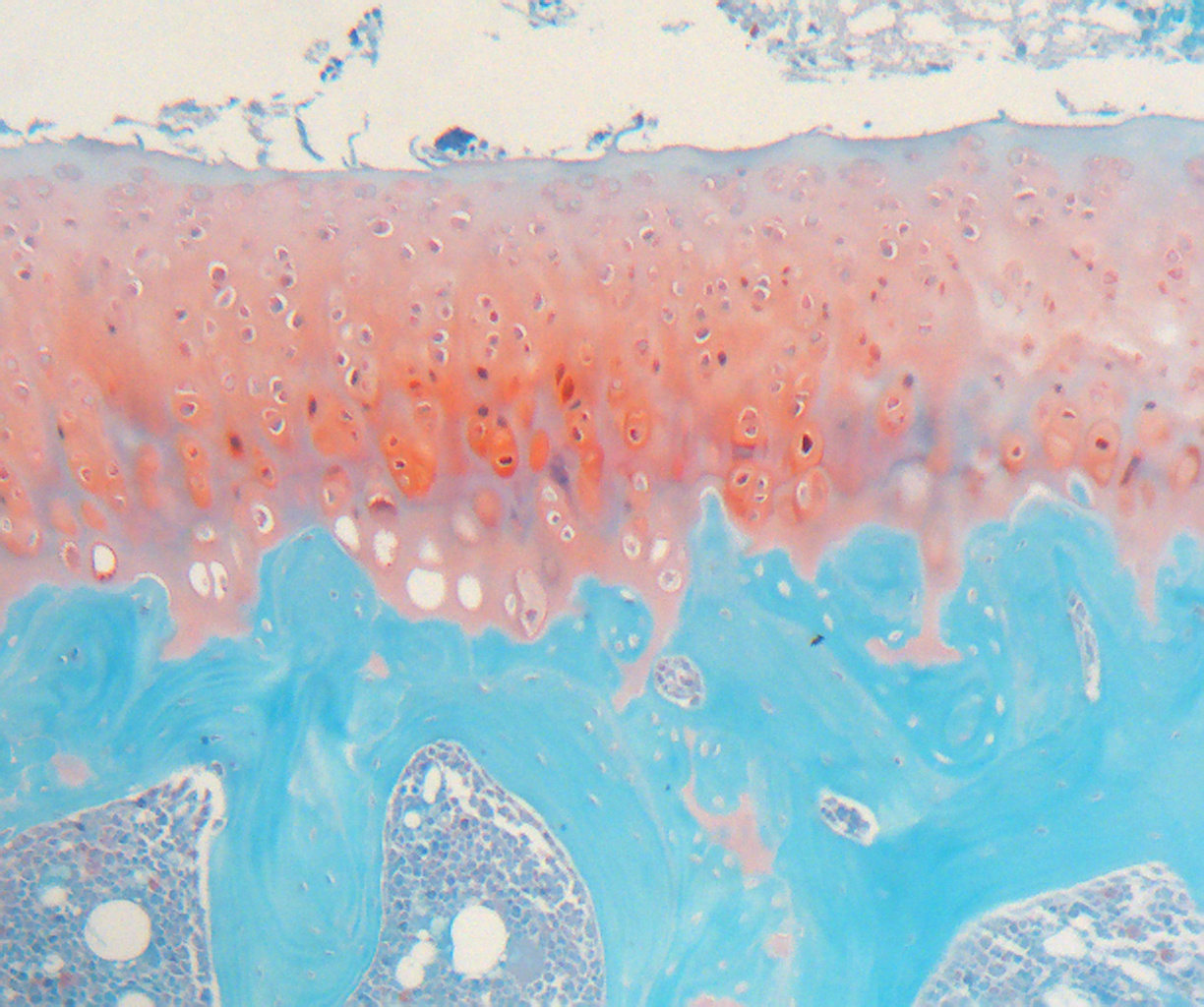

Supplement: Supplementary file 3 [file DataSheet_2.zip › SOFA/Model/1.jpg]

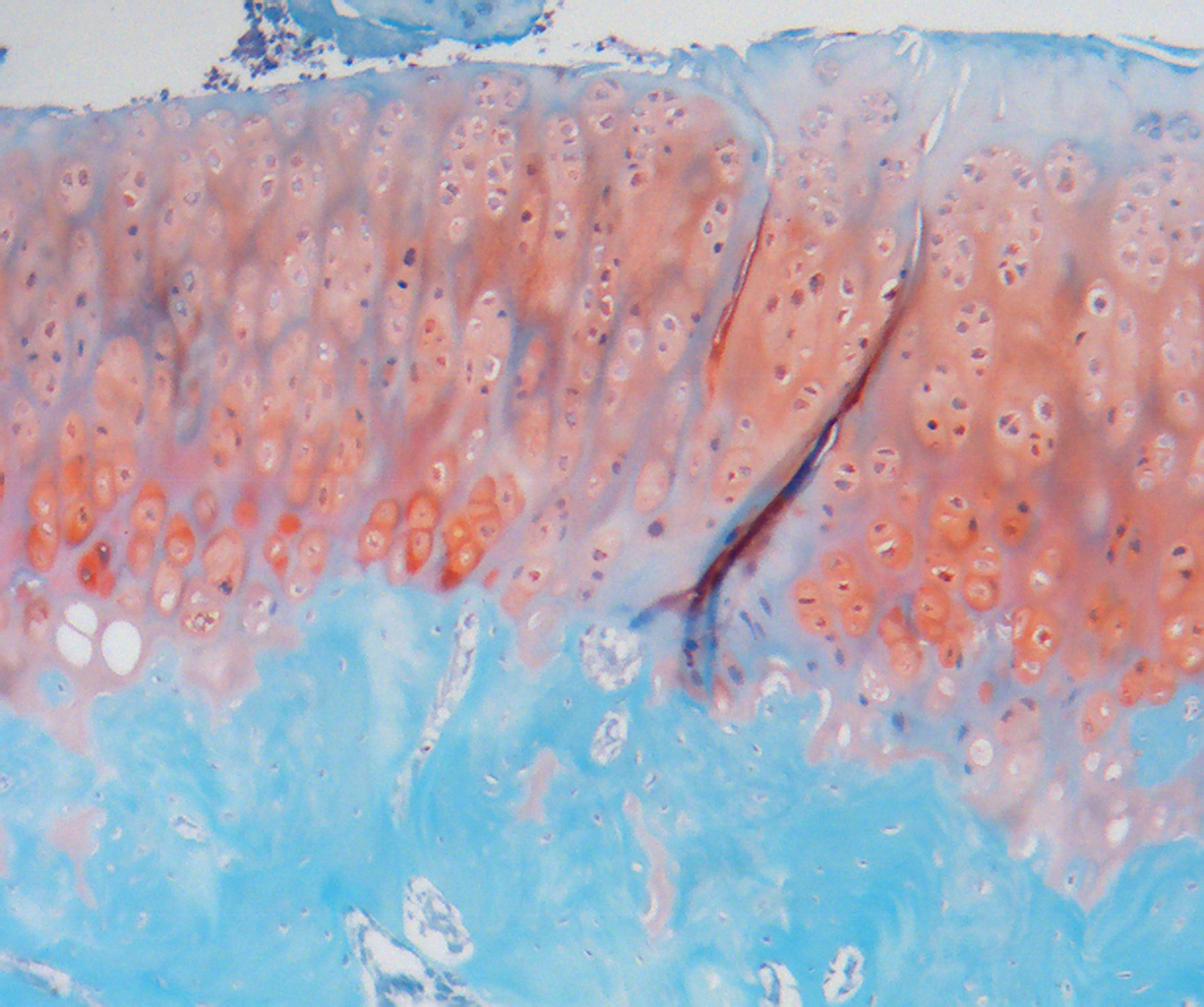

Supplement: Supplementary file 3 [file DataSheet_2.zip › SOFA/Model/2.jpg]

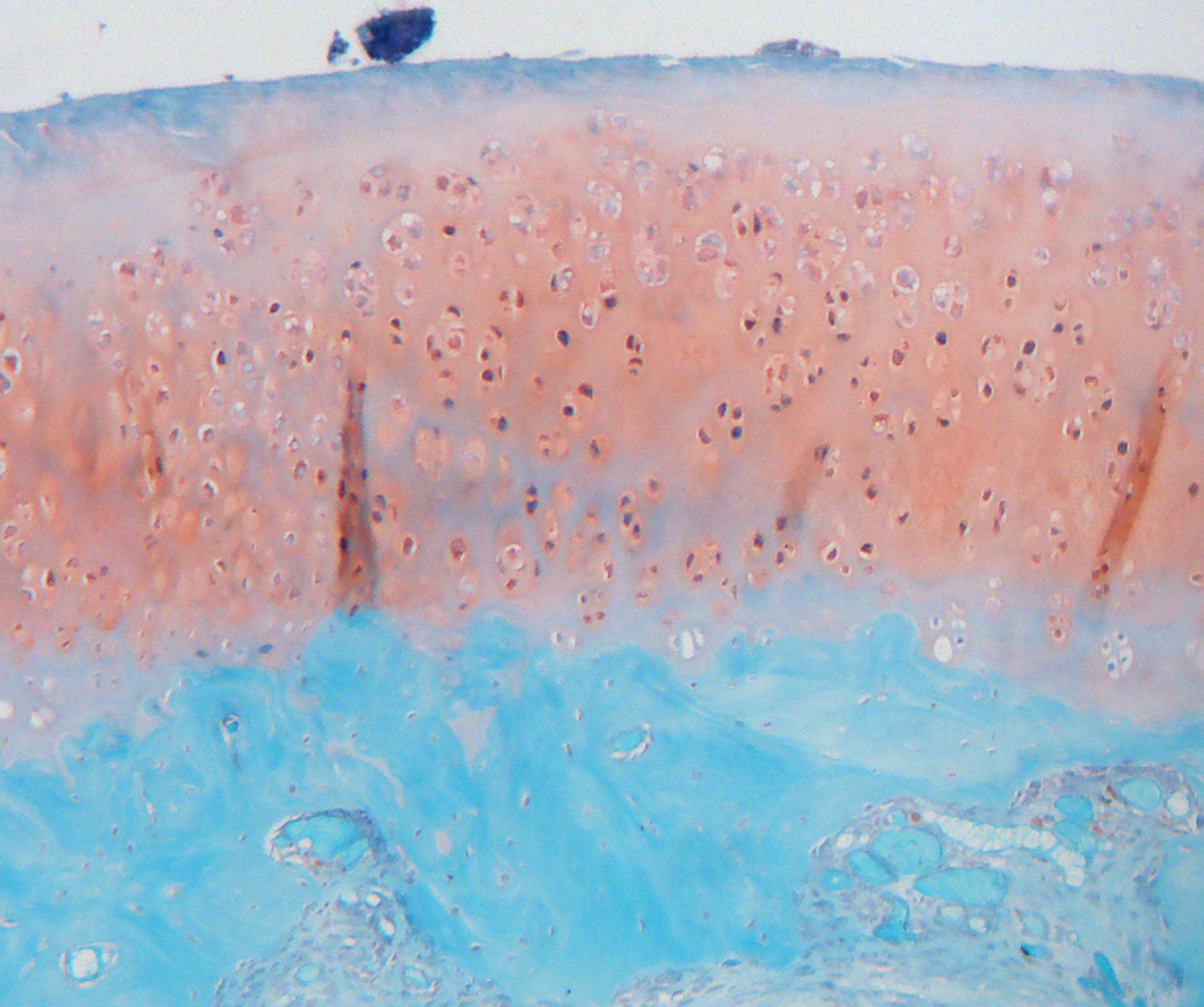

Supplement: Supplementary file 3 [file DataSheet_2.zip › SOFA/Model/3.jpg]

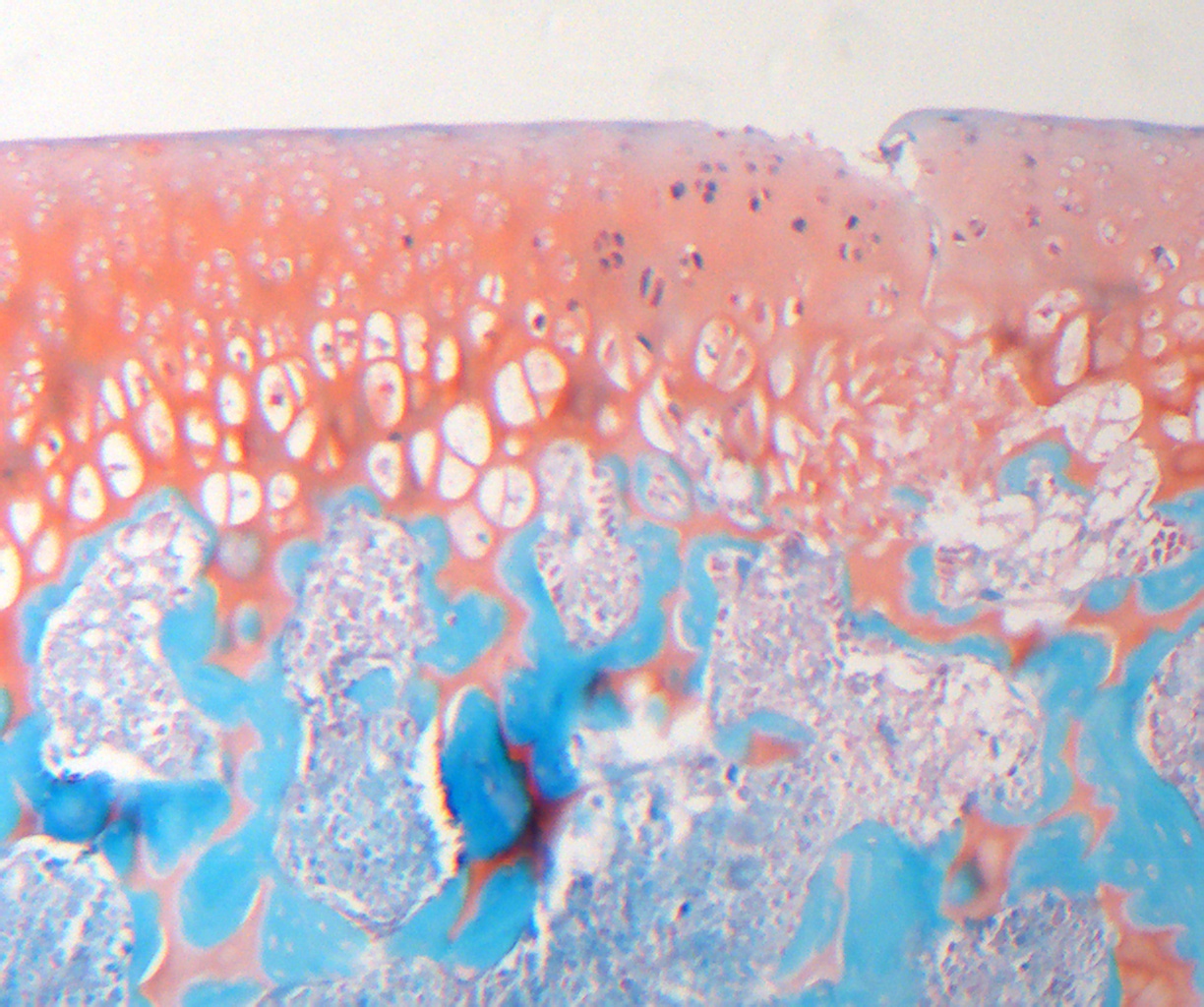

Supplement: Supplementary file 3 [file DataSheet_2.zip › SOFA/Model/4.jpg]

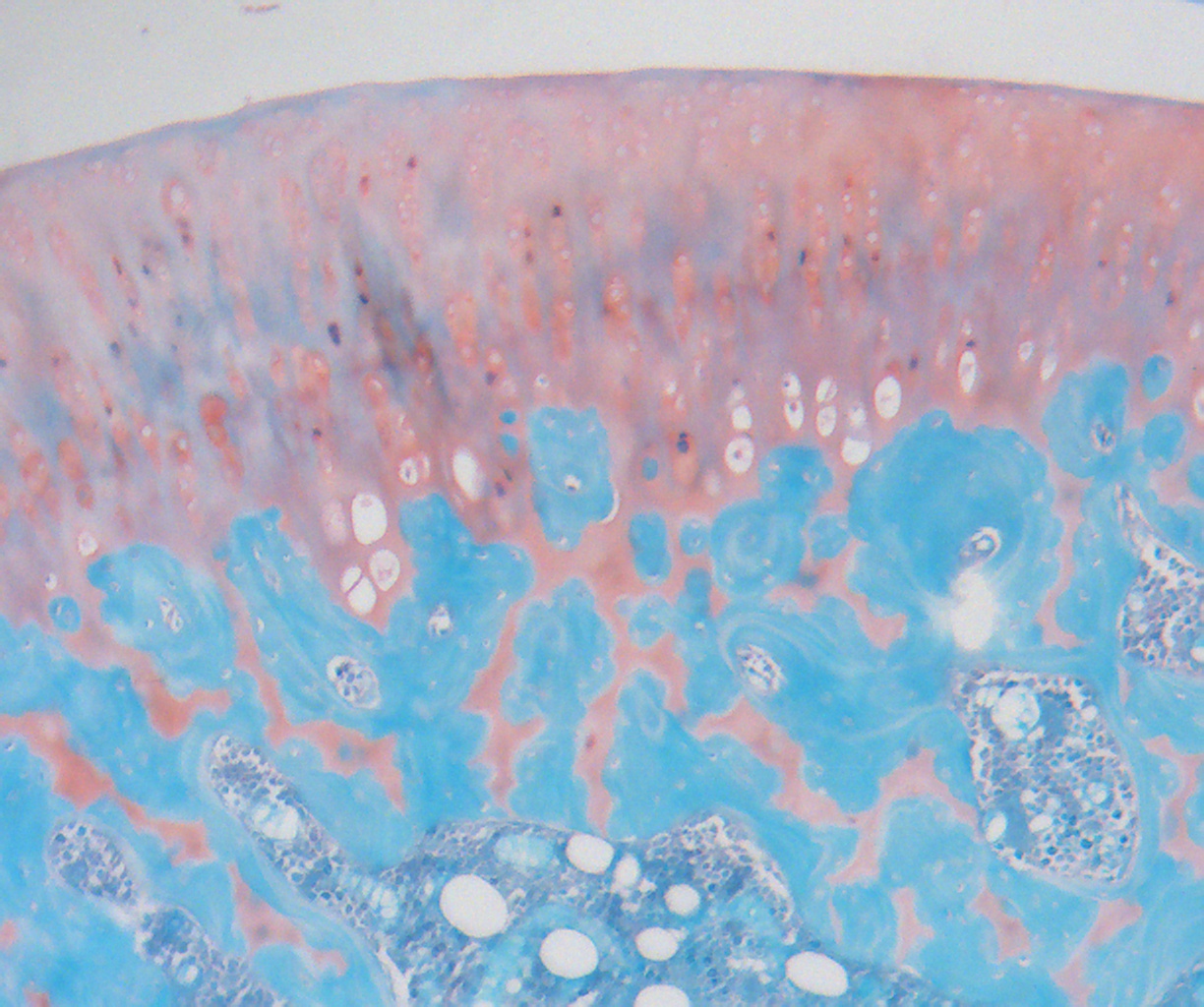

Supplement: Supplementary file 3 [file DataSheet_2.zip › SOFA/Model/5.jpg]

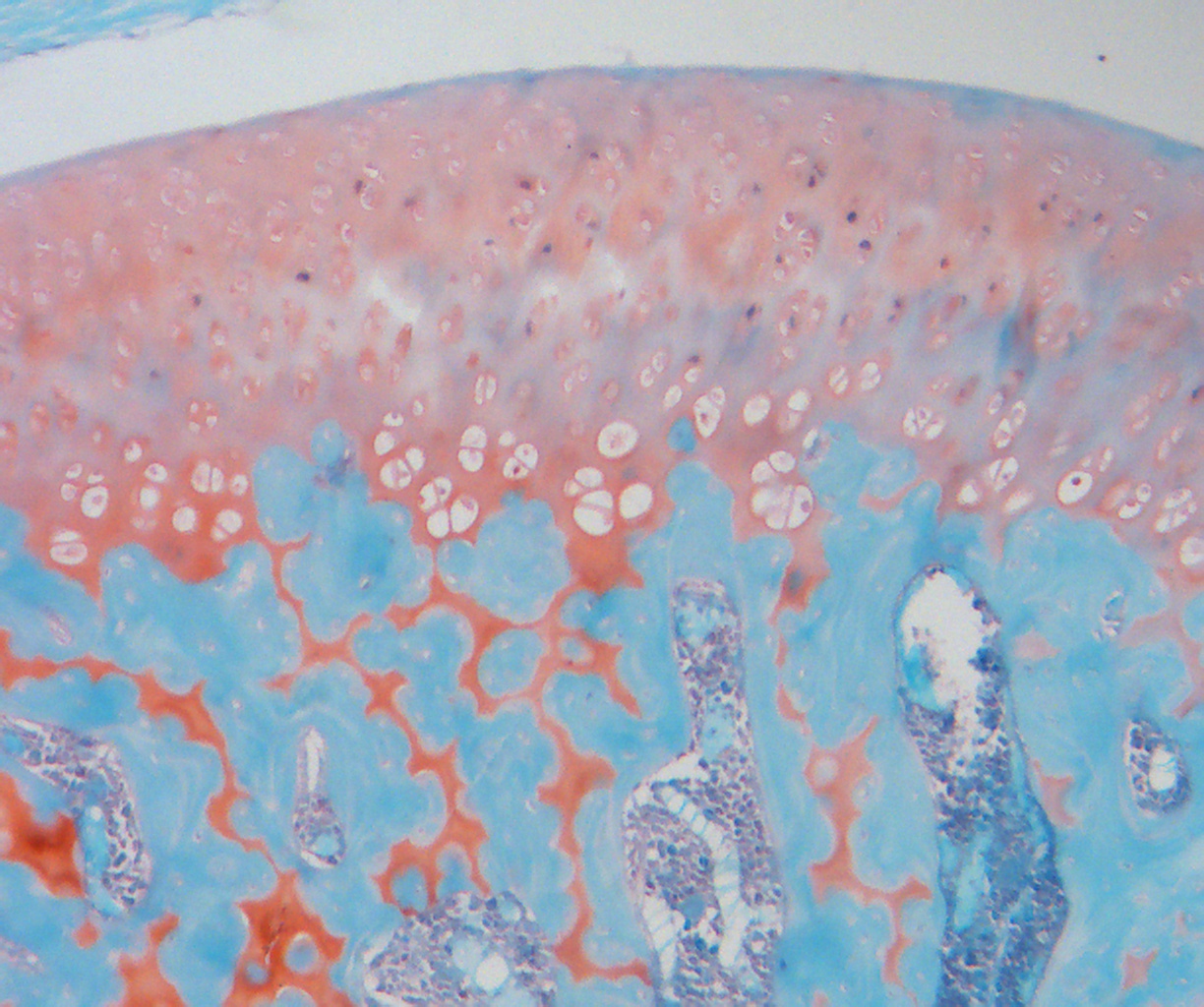

Supplement: Supplementary file 3 [file DataSheet_2.zip › SOFA/Model/6.jpg]

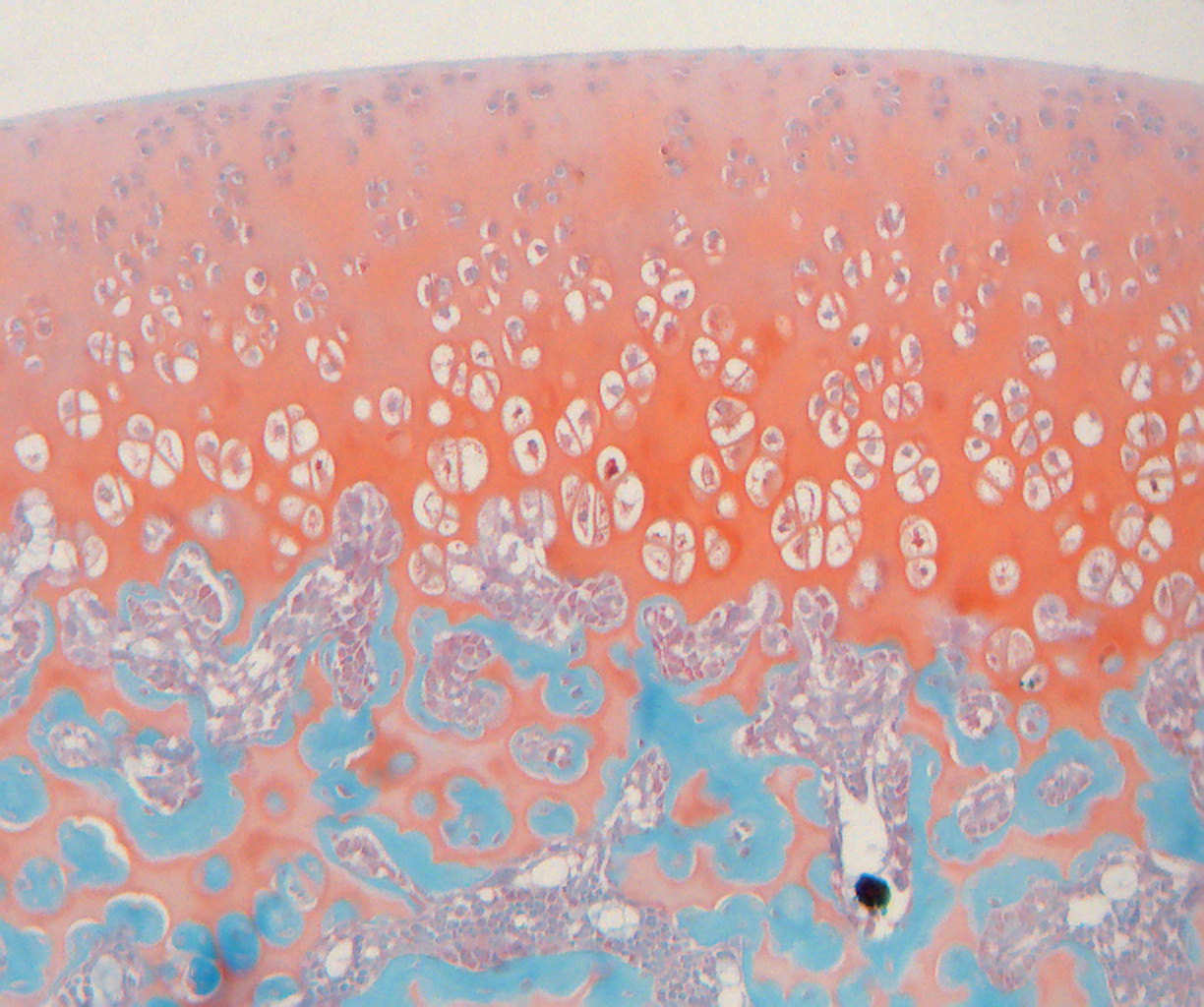

Supplement: Supplementary file 3 [file DataSheet_2.zip › SOFA/Sham/1.jpg]

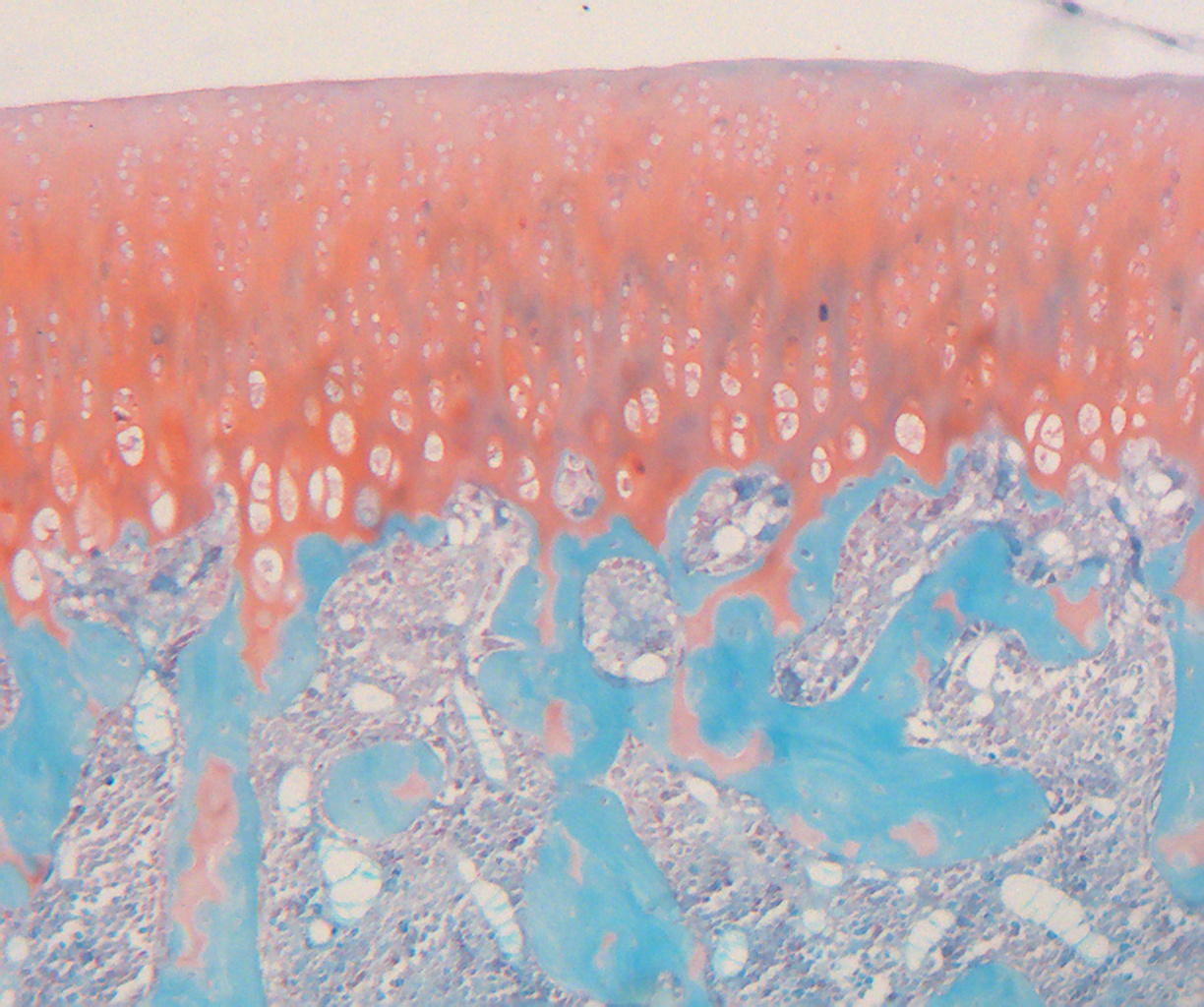

Supplement: Supplementary file 3 [file DataSheet_2.zip › SOFA/Sham/2.jpg]

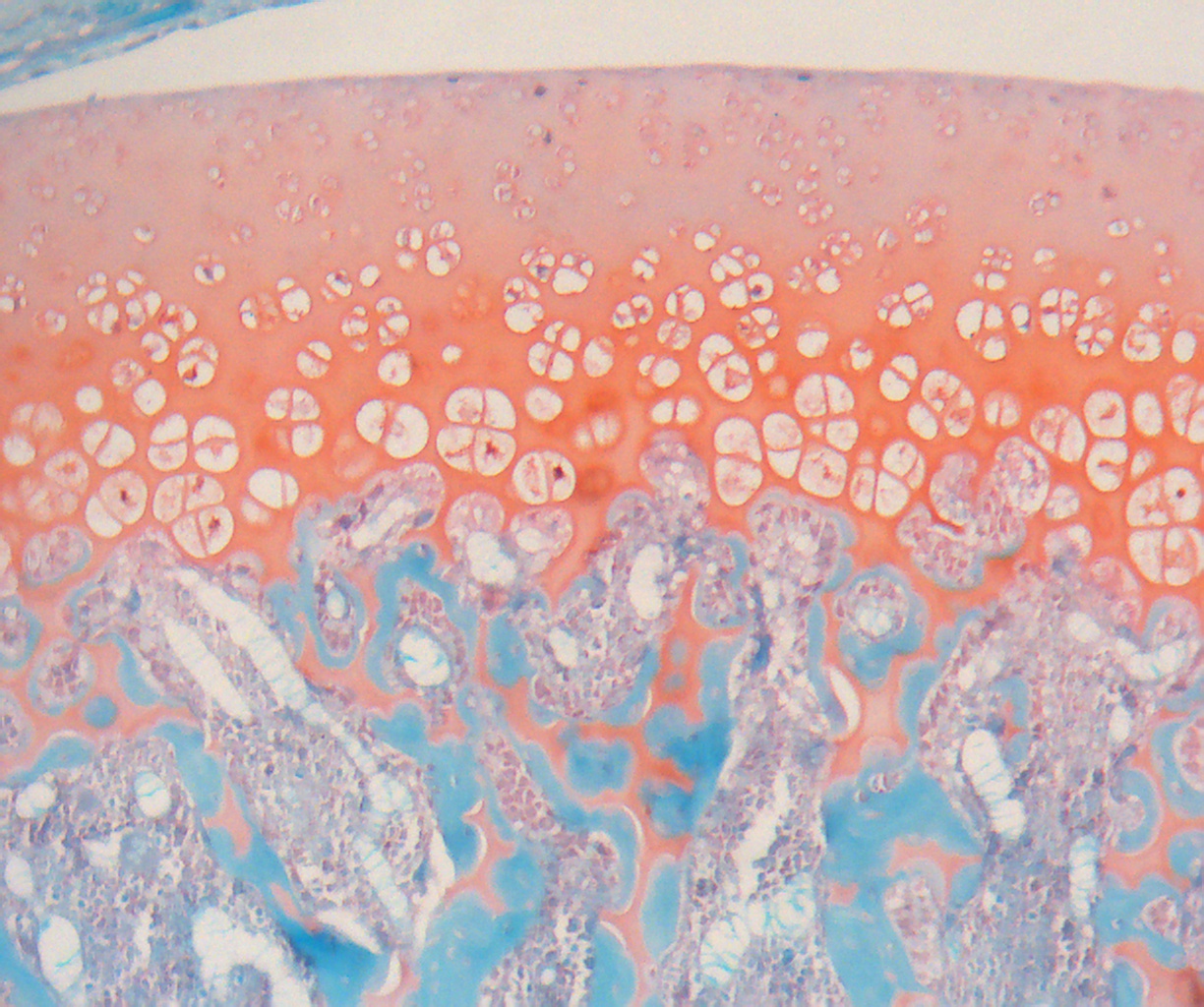

Supplement: Supplementary file 3 [file DataSheet_2.zip › SOFA/Sham/3.jpg]

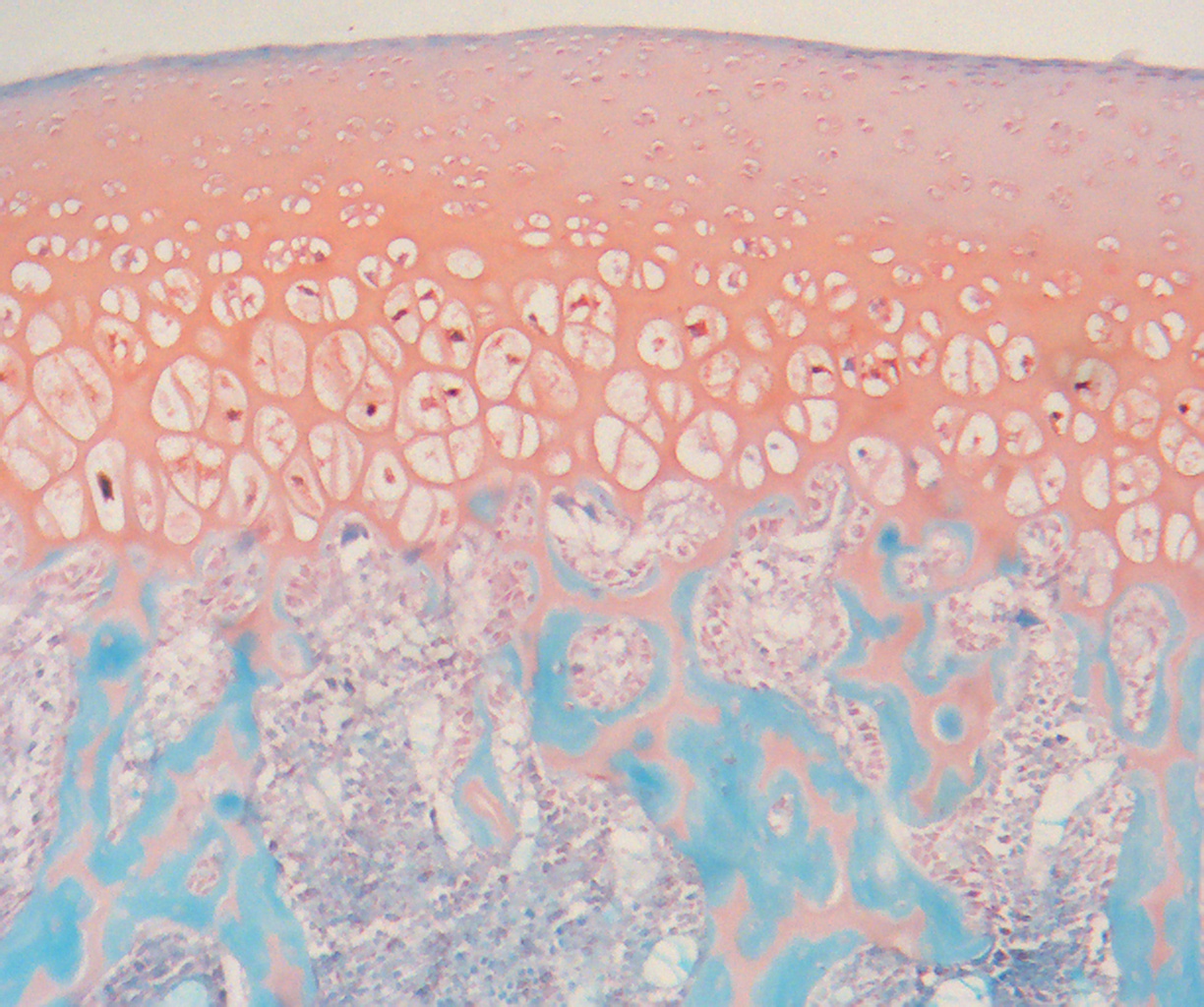

Supplement: Supplementary file 3 [file DataSheet_2.zip › SOFA/Sham/4.jpg]

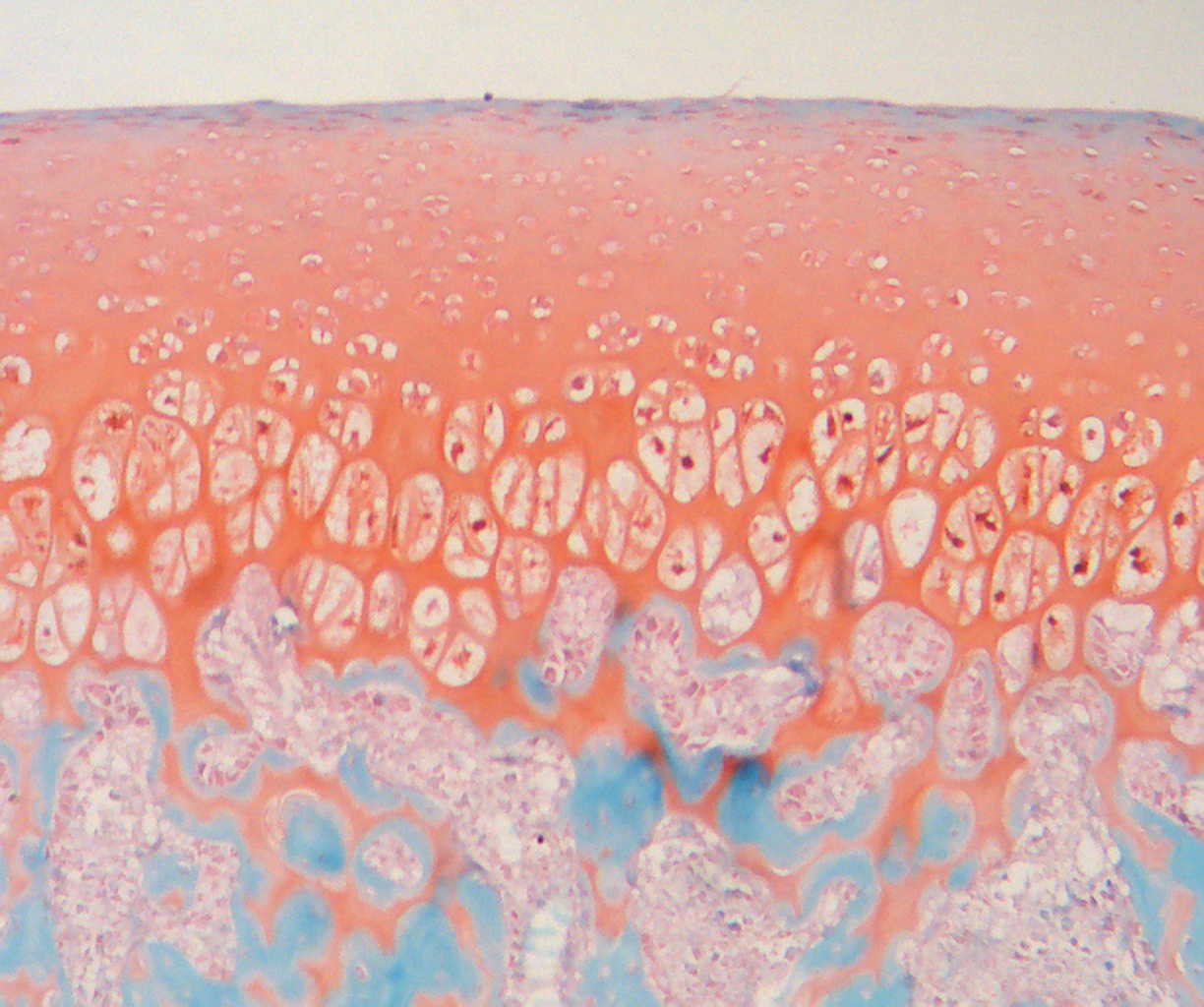

Supplement: Supplementary file 3 [file DataSheet_2.zip › SOFA/Sham/5.jpg]

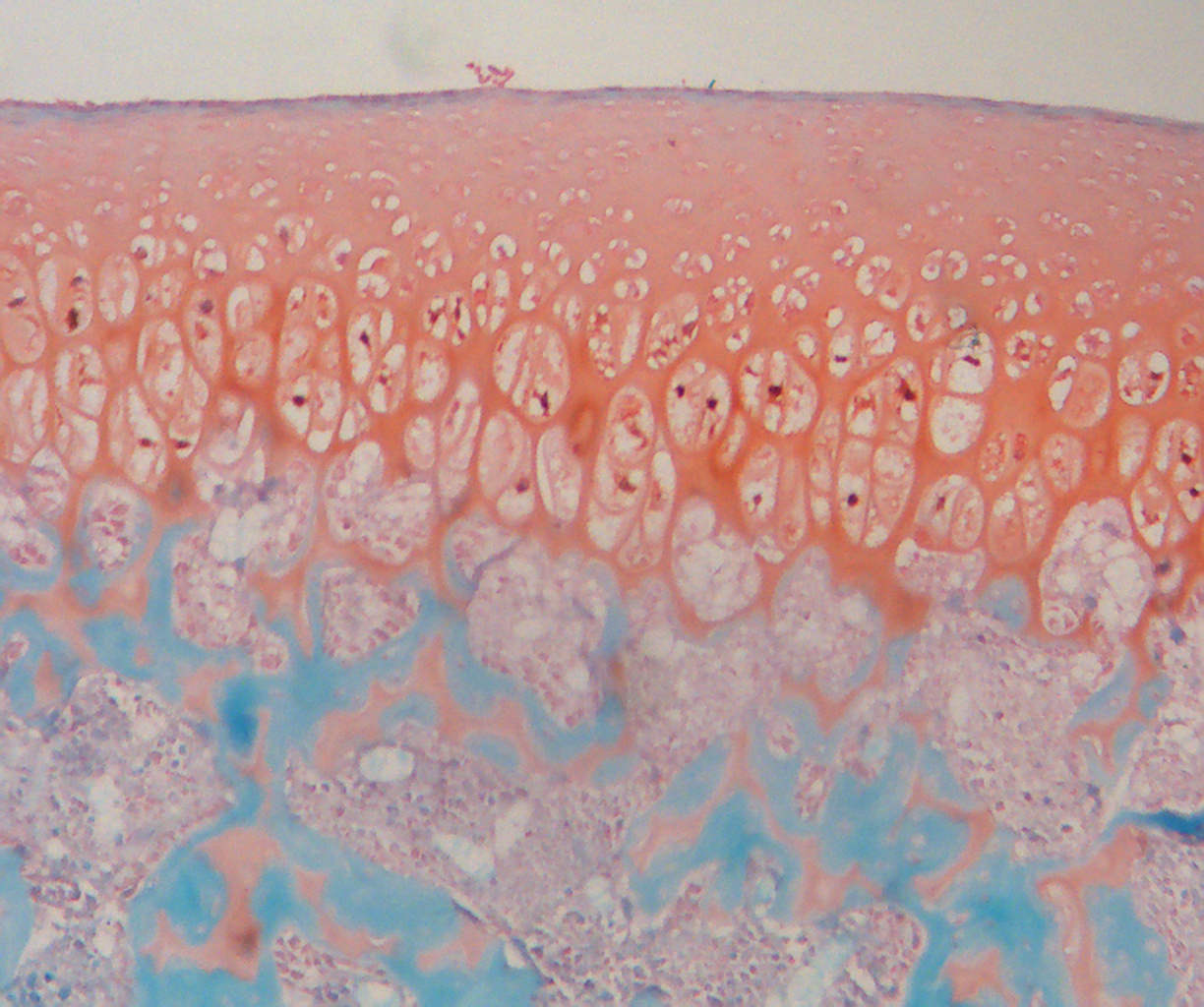

Supplement: Supplementary file 3 [file DataSheet_2.zip › SOFA/Sham/6.jpg]

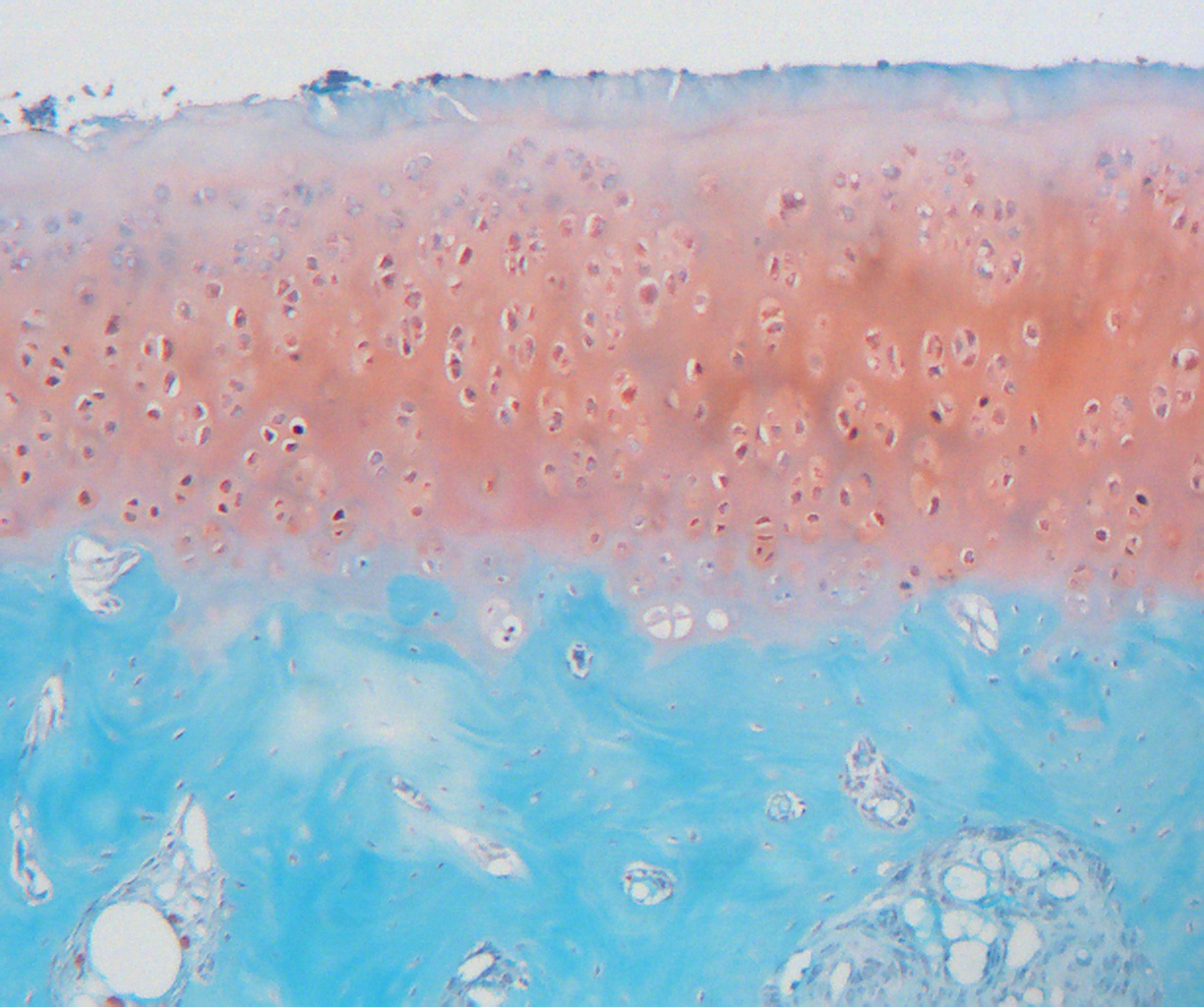

Supplement: Supplementary file 3 [file DataSheet_2.zip › SOFA/miR(-) Exo/1.jpg]

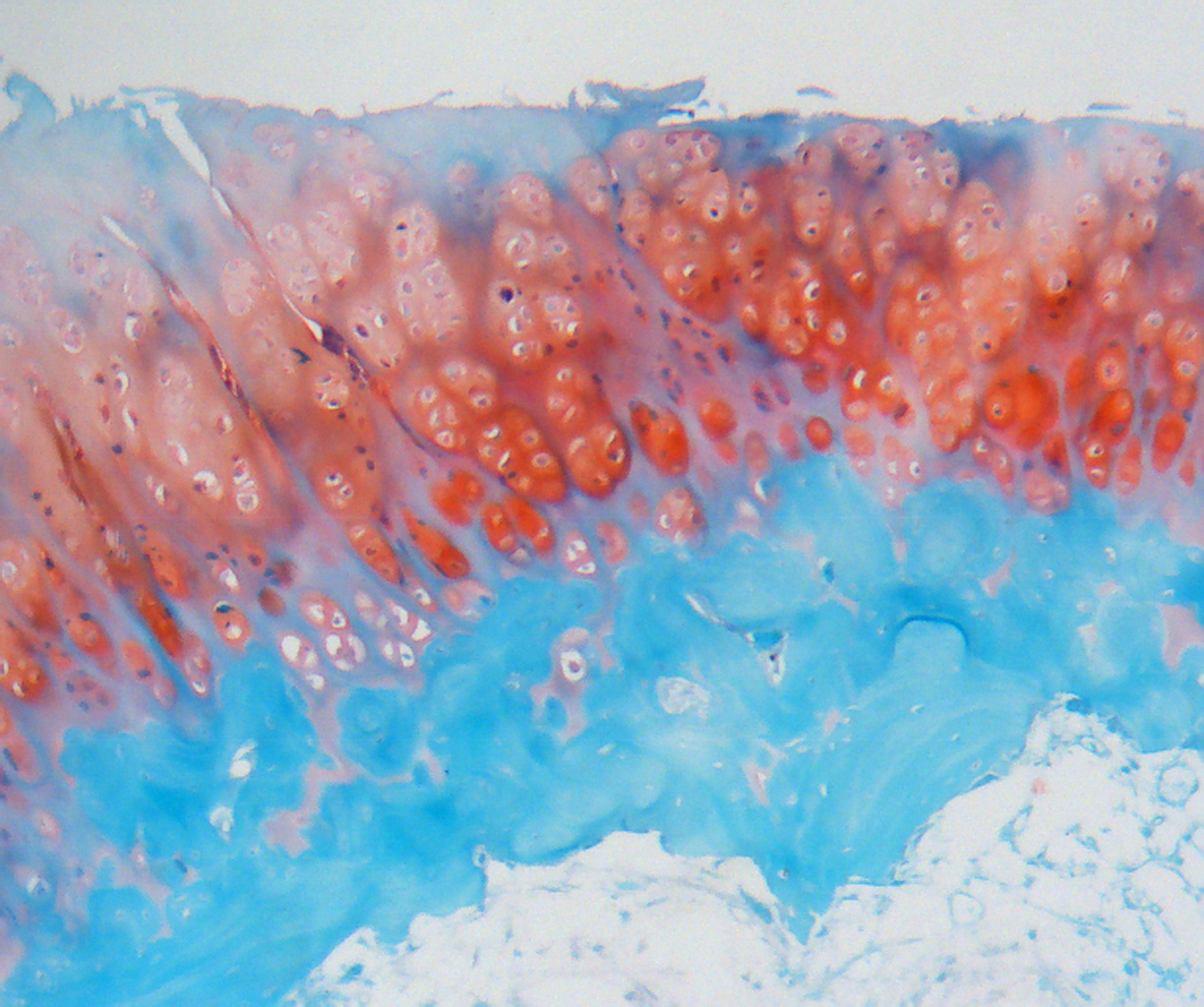

Supplement: Supplementary file 3 [file DataSheet_2.zip › SOFA/miR(-) Exo/2.jpg]

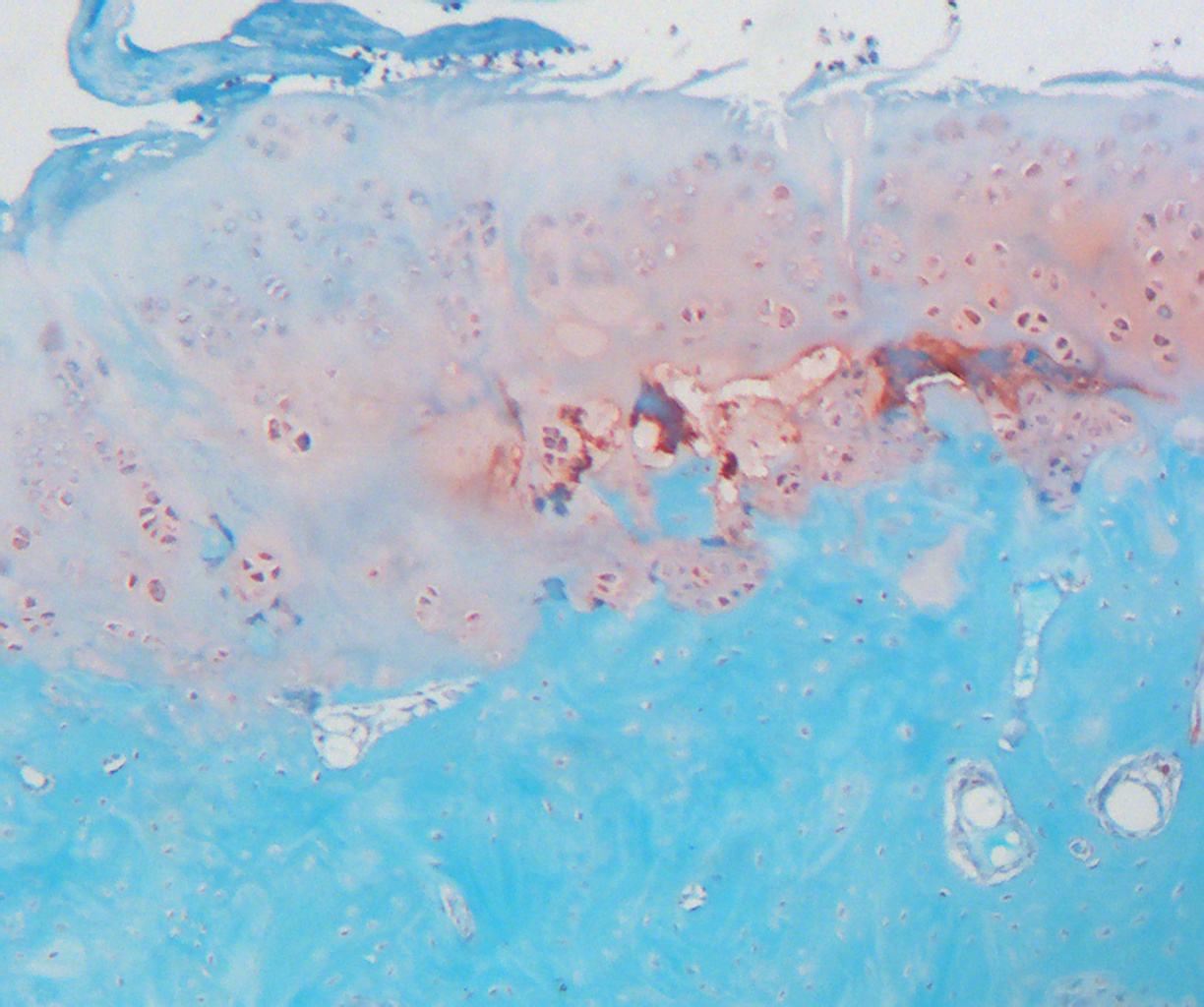

Supplement: Supplementary file 3 [file DataSheet_2.zip › SOFA/miR(-) Exo/3.jpg]

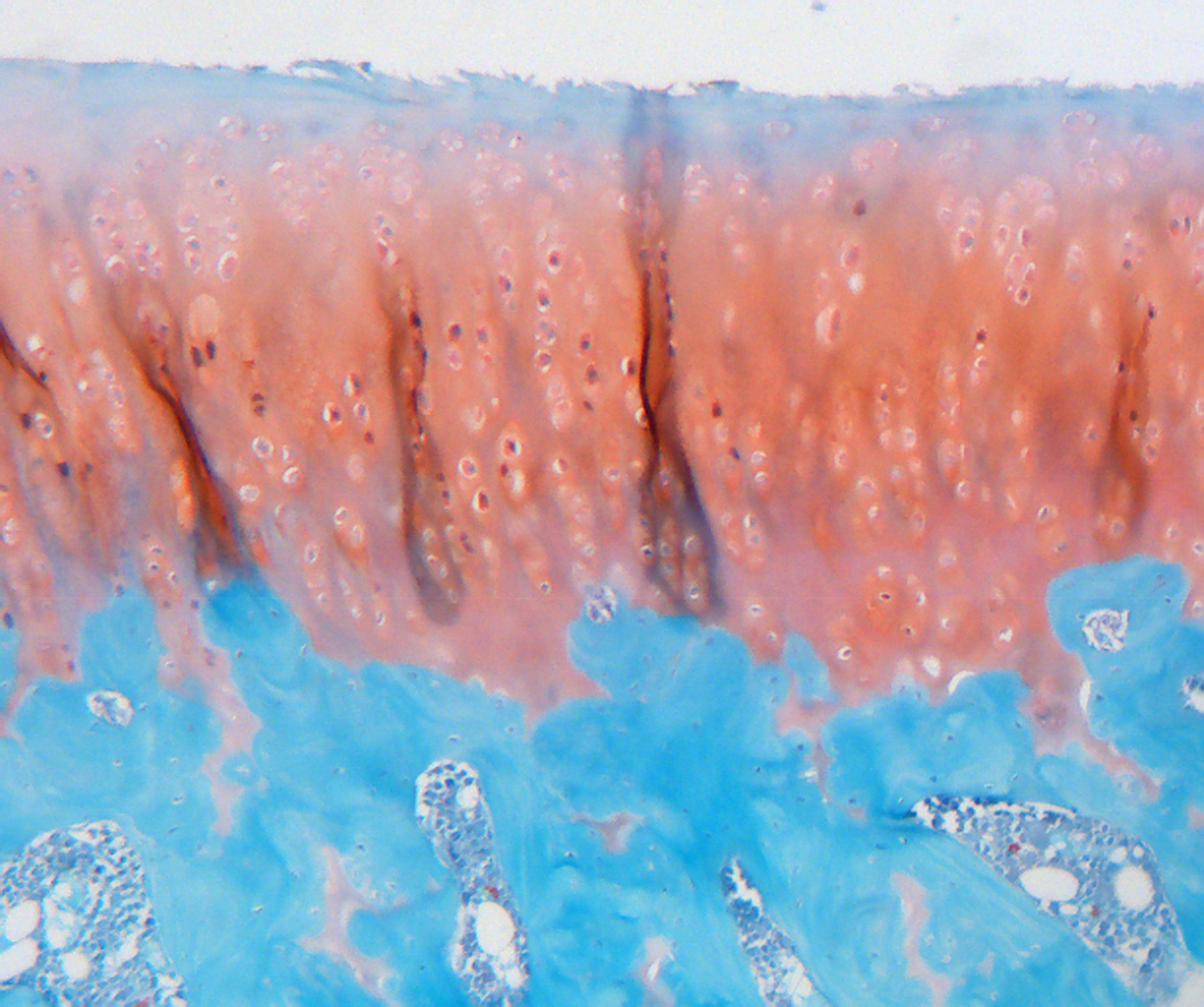

Supplement: Supplementary file 3 [file DataSheet_2.zip › SOFA/miR(-) Exo/4.jpg]

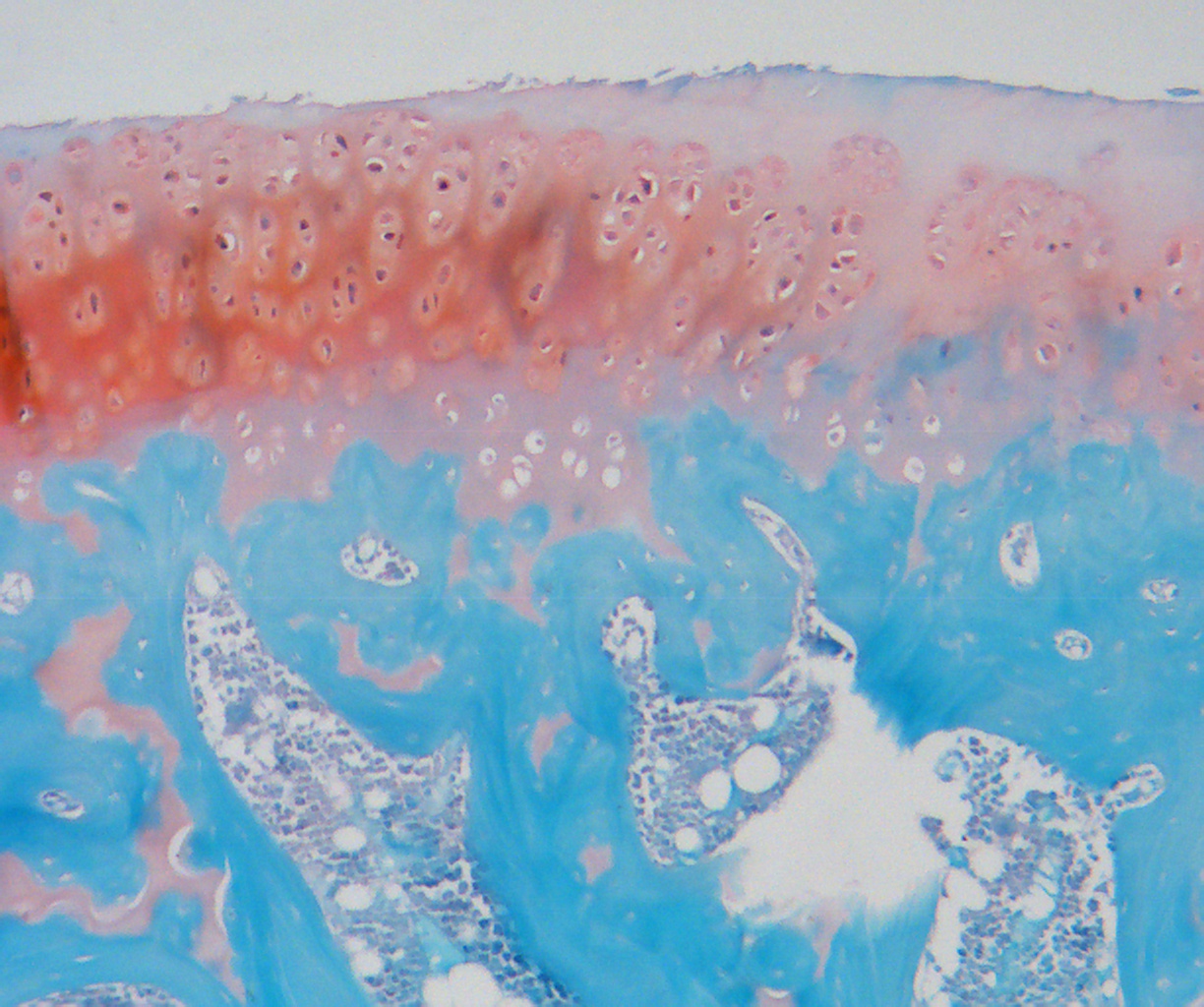

Supplement: Supplementary file 3 [file DataSheet_2.zip › SOFA/miR(-) Exo/5.jpg]

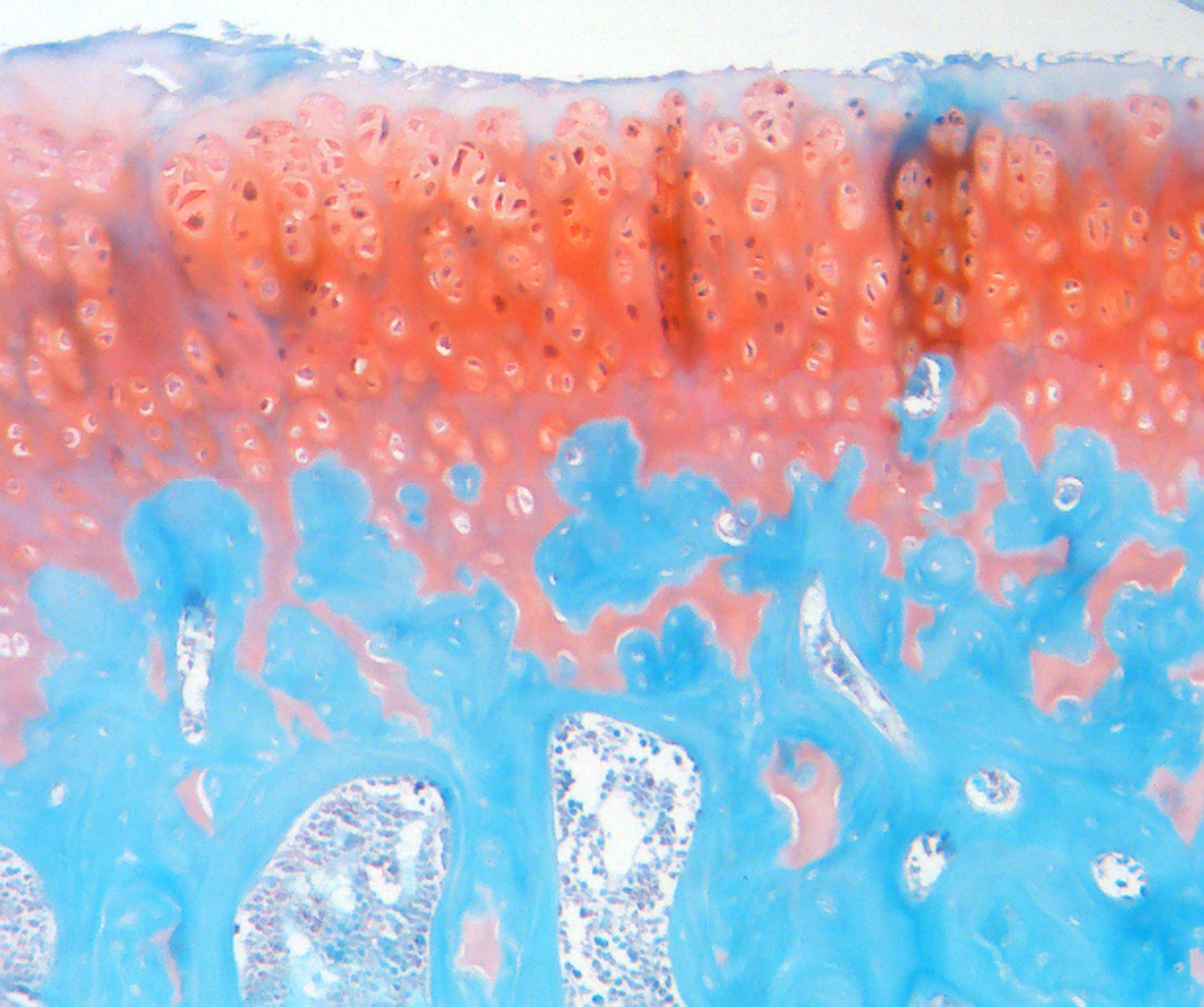

Supplement: Supplementary file 3 [file DataSheet_2.zip › SOFA/miR(-) Exo/6.jpg]

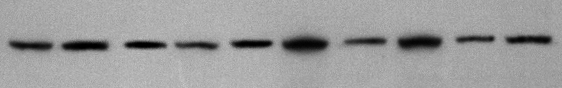

Supplement: Supplementary file 4 [file DataSheet_3.zip › WB/Cartilage/ACSL4/ACSL4-1.jpg]

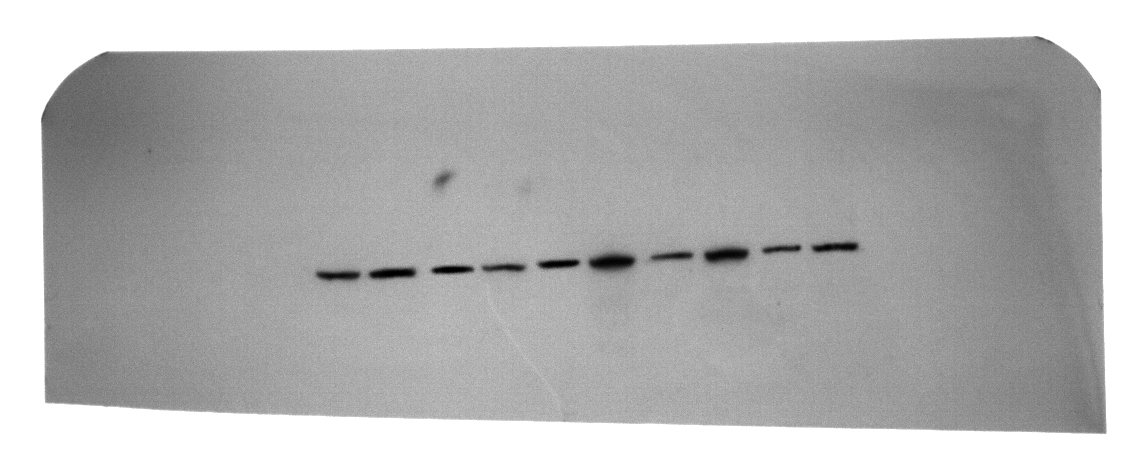

Supplement: Supplementary file 4 [file DataSheet_3.zip › WB/Cartilage/ACSL4/ACSL4-1╘¡═╝.jpg]

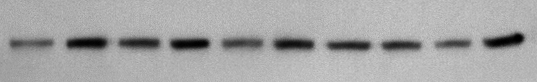

Supplement: Supplementary file 4 [file DataSheet_3.zip › WB/Cartilage/ACSL4/ACSL4-2.jpg]

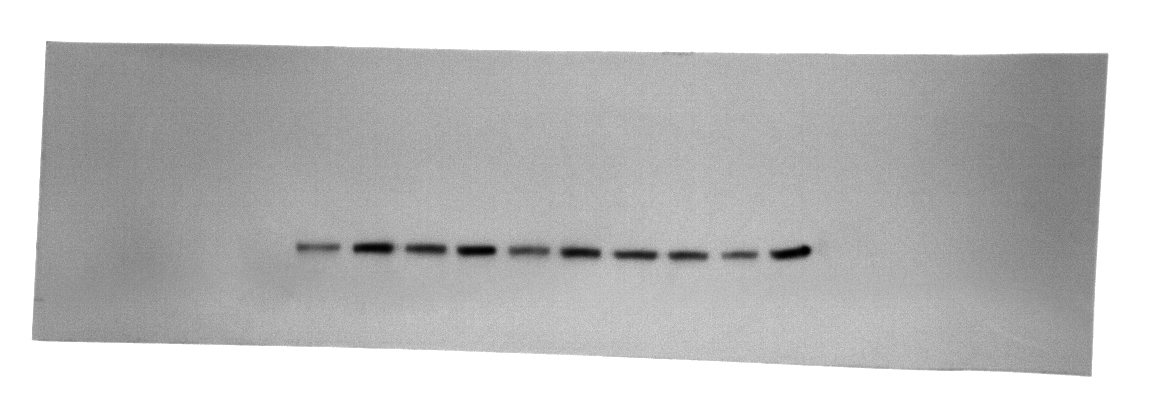

Supplement: Supplementary file 4 [file DataSheet_3.zip › WB/Cartilage/ACSL4/ACSL4-2╘¡═╝.jpg]

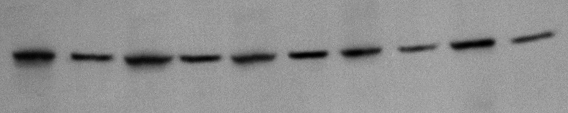

Supplement: Supplementary file 4 [file DataSheet_3.zip › WB/Cartilage/GPX4/GPX4-1.jpg]

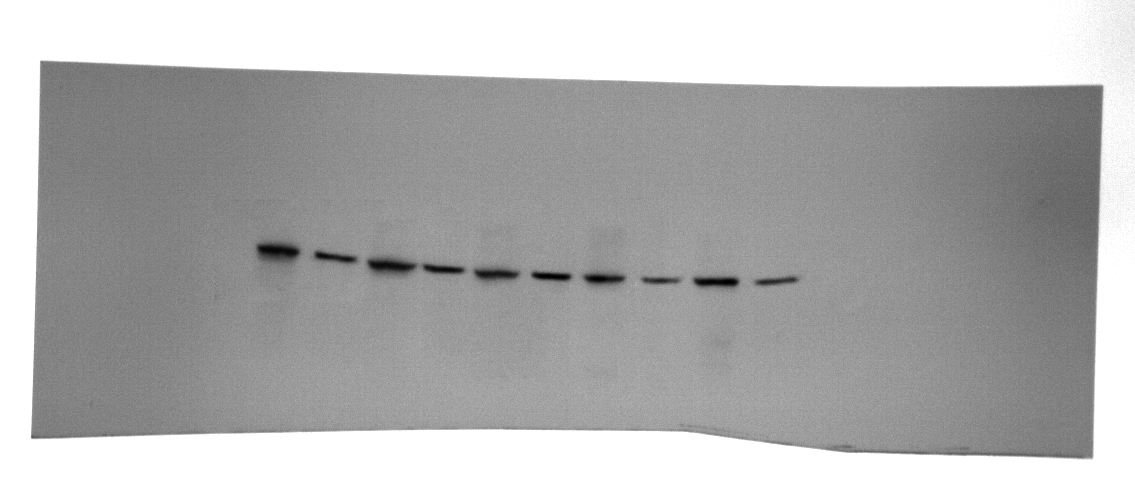

Supplement: Supplementary file 4 [file DataSheet_3.zip › WB/Cartilage/GPX4/GPX4-1╘¡═╝.jpg]

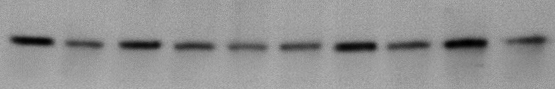

Supplement: Supplementary file 4 [file DataSheet_3.zip › WB/Cartilage/GPX4/GPX4-2.jpg]

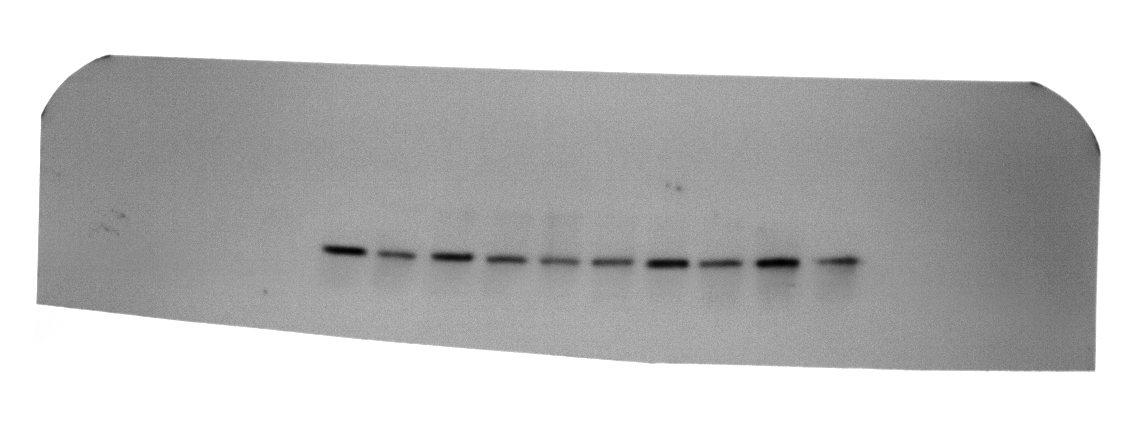

Supplement: Supplementary file 4 [file DataSheet_3.zip › WB/Cartilage/GPX4/GPX4-2╘¡═╝.jpg]

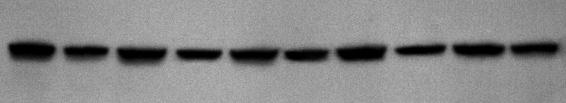

Supplement: Supplementary file 4 [file DataSheet_3.zip › WB/Cartilage/SLC7A11/SLC7A11-1.jpg]

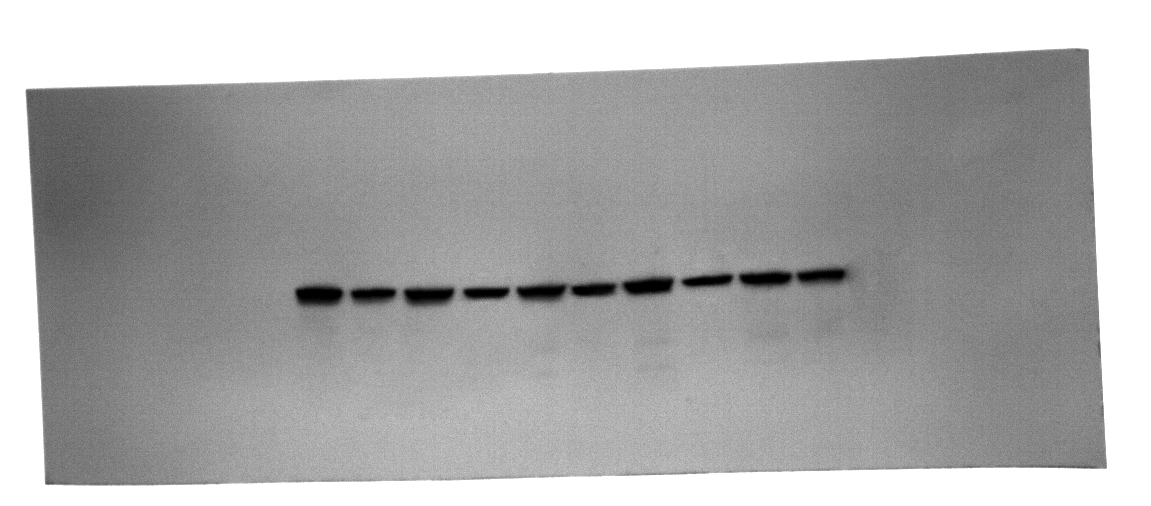

Supplement: Supplementary file 4 [file DataSheet_3.zip › WB/Cartilage/SLC7A11/SLC7A11-1╘¡═╝.jpg]

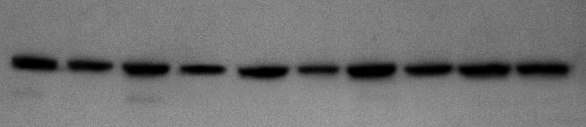

Supplement: Supplementary file 4 [file DataSheet_3.zip › WB/Cartilage/SLC7A11/SLC7A11-2.jpg]

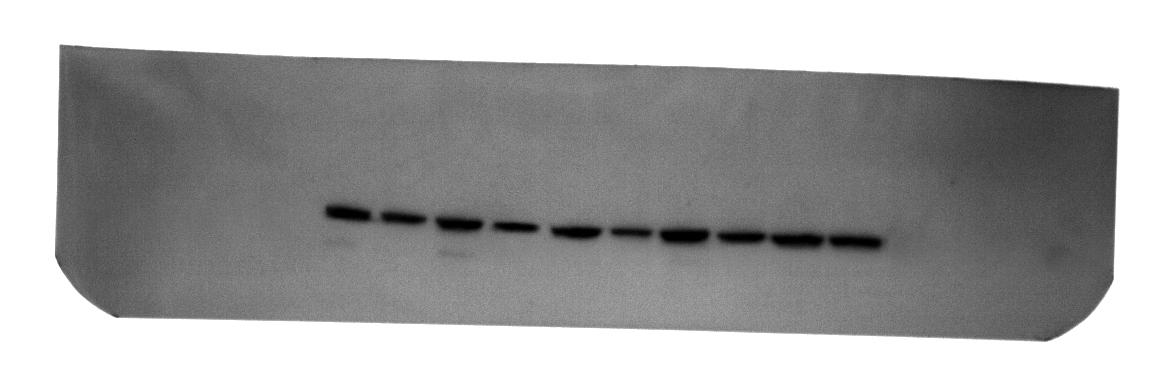

Supplement: Supplementary file 4 [file DataSheet_3.zip › WB/Cartilage/SLC7A11/SLC7A11-2╘¡═╝.jpg]

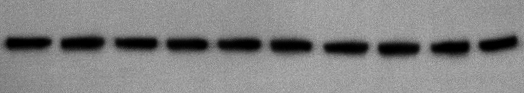

Supplement: Supplementary file 4 [file DataSheet_3.zip › WB/Cartilage/a┬-actin/a┬-actin-1.jpg]

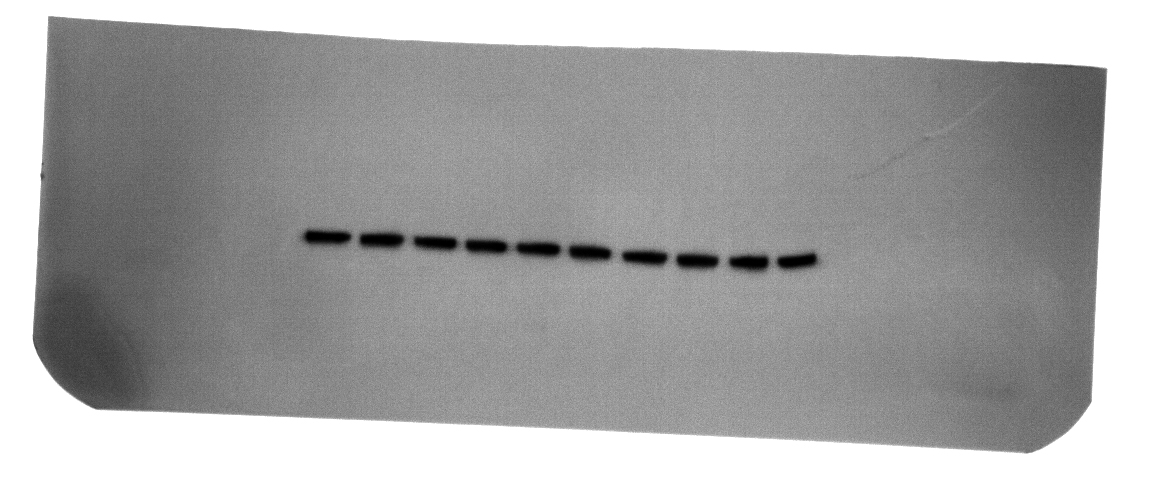

Supplement: Supplementary file 4 [file DataSheet_3.zip › WB/Cartilage/a┬-actin/a┬-actin-1╘¡═╝.jpg]

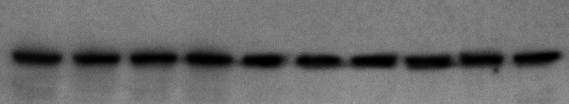

Supplement: Supplementary file 4 [file DataSheet_3.zip › WB/Cartilage/a┬-actin/a┬-actin-2.jpg]

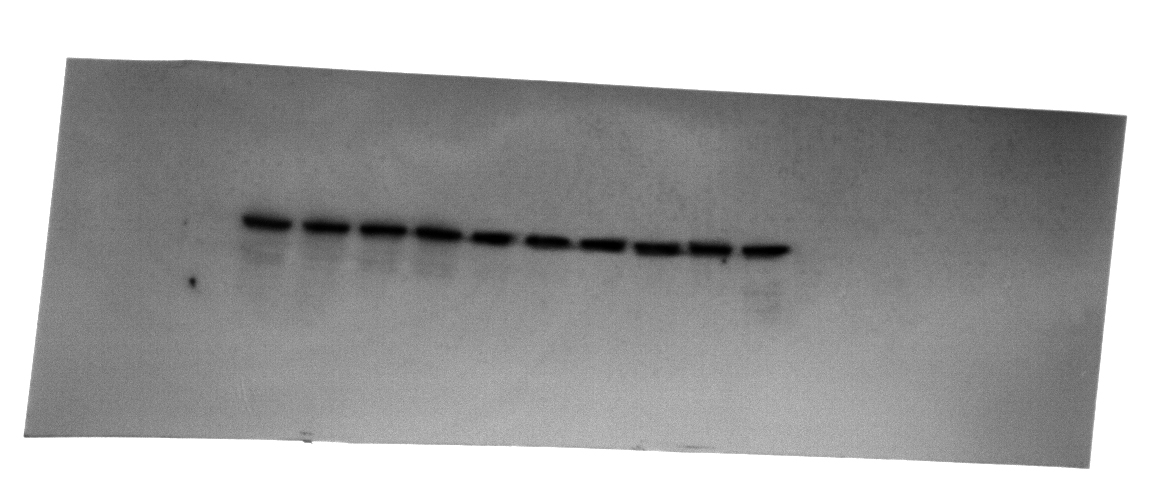

Supplement: Supplementary file 4 [file DataSheet_3.zip › WB/Cartilage/a┬-actin/a┬-actin-2╘¡═╝.jpg]
