# Supplementary figures and images for: Exosomes from osteoarthritic fibroblast-like synoviocytes promote cartilage ferroptosis and damage via delivering microRNA-19b-3p to target SLC7A11 in osteoarthritis (part 3 of 6)
Source: Front Immunol. 2023 Aug 24;14:1181156. doi: 10.3389/fimmu.2023.1181156 (PMC10484587; doi:10.3389/fimmu.2023.1181156)

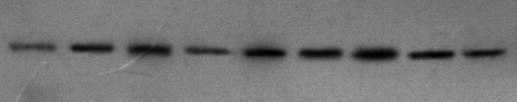

Supplement: Supplementary file 4 [file DataSheet_3.zip › WB/Cell experiments/ACSL4/1/Fig2+Fig3.jpg]

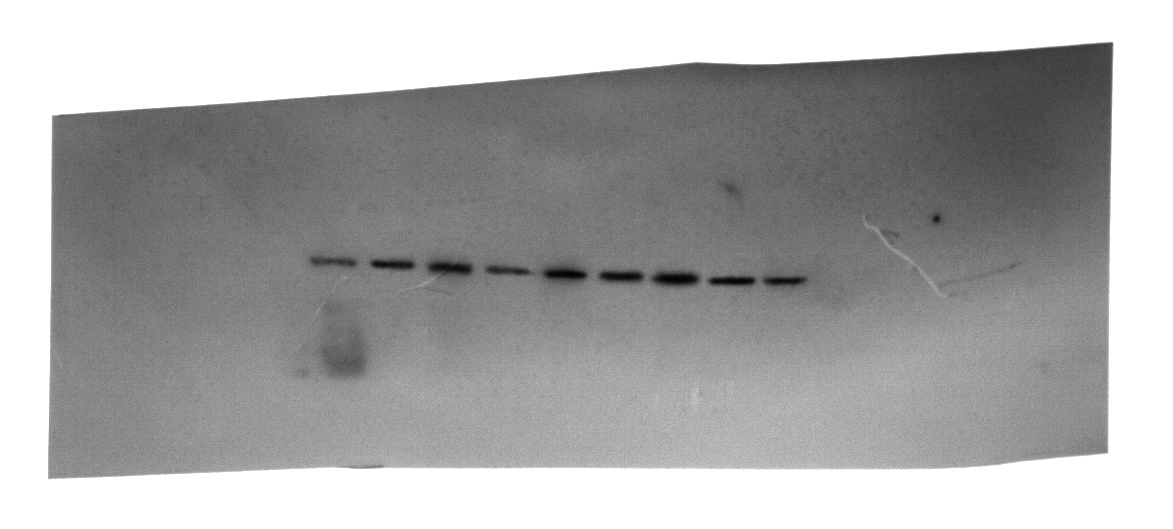

Supplement: Supplementary file 4 [file DataSheet_3.zip › WB/Cell experiments/ACSL4/1/Fig2+Fig3╘¡═╝.jpg]

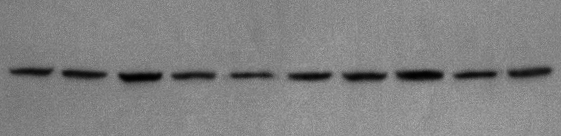

Supplement: Supplementary file 4 [file DataSheet_3.zip › WB/Cell experiments/ACSL4/1/Fig4+Fig5.jpg]

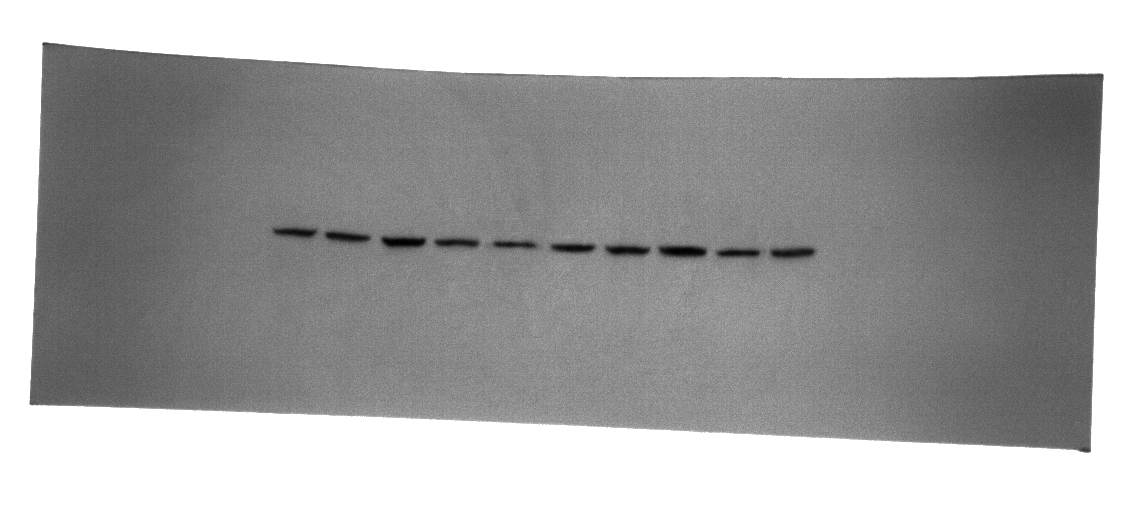

Supplement: Supplementary file 4 [file DataSheet_3.zip › WB/Cell experiments/ACSL4/1/Fig4+Fig5╘¡═╝.jpg]

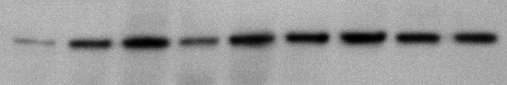

Supplement: Supplementary file 4 [file DataSheet_3.zip › WB/Cell experiments/ACSL4/2/Fig2+Fig3.jpg]

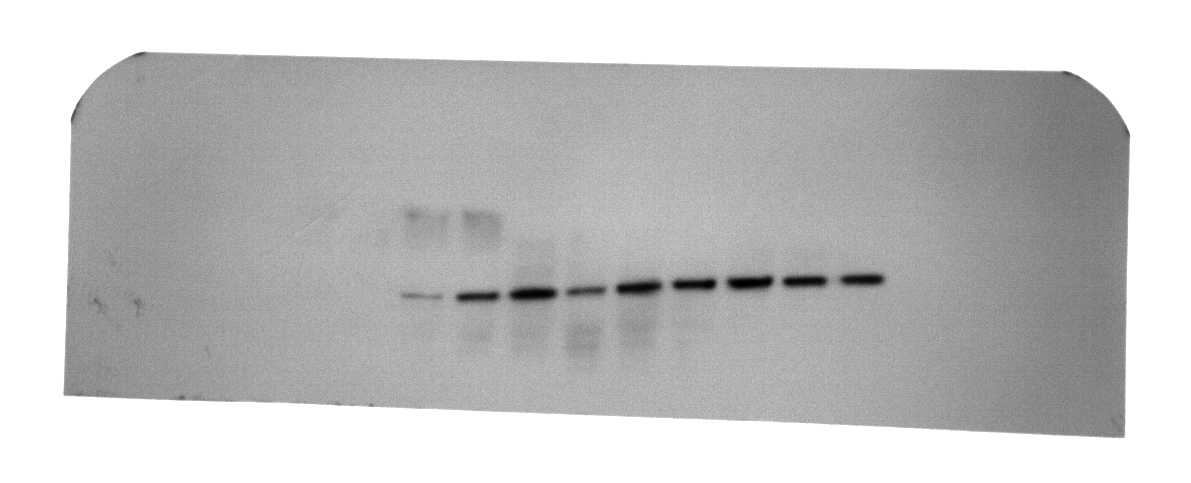

Supplement: Supplementary file 4 [file DataSheet_3.zip › WB/Cell experiments/ACSL4/2/Fig2+Fig3╘¡═╝.jpg]

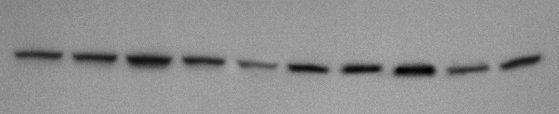

Supplement: Supplementary file 4 [file DataSheet_3.zip › WB/Cell experiments/ACSL4/2/Fig4+Fig5.jpg]

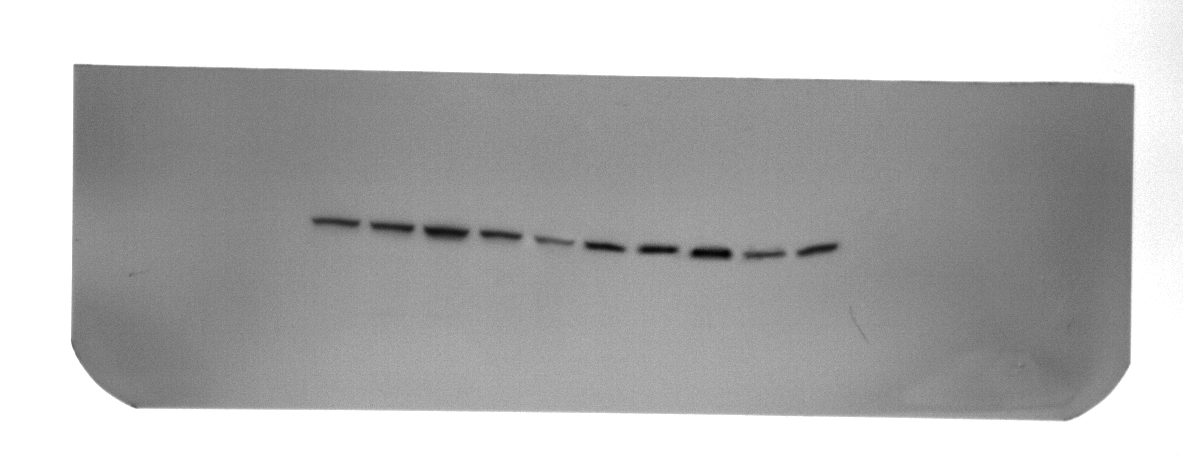

Supplement: Supplementary file 4 [file DataSheet_3.zip › WB/Cell experiments/ACSL4/2/Fig4+Fig5╘¡═╝.jpg]

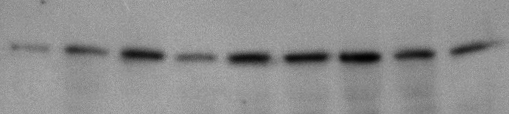

Supplement: Supplementary file 4 [file DataSheet_3.zip › WB/Cell experiments/ACSL4/3/Fig2+Fig3.jpg]

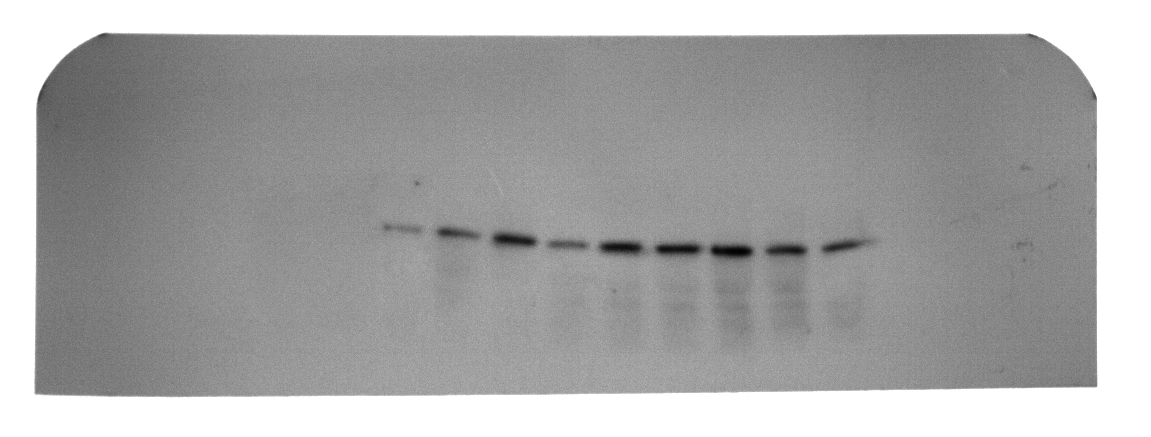

Supplement: Supplementary file 4 [file DataSheet_3.zip › WB/Cell experiments/ACSL4/3/Fig2+Fig3╘¡═╝.jpg]

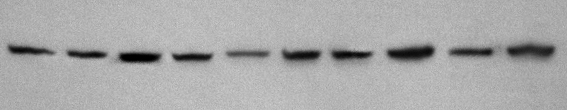

Supplement: Supplementary file 4 [file DataSheet_3.zip › WB/Cell experiments/ACSL4/3/Fig4+Fig5.jpg]

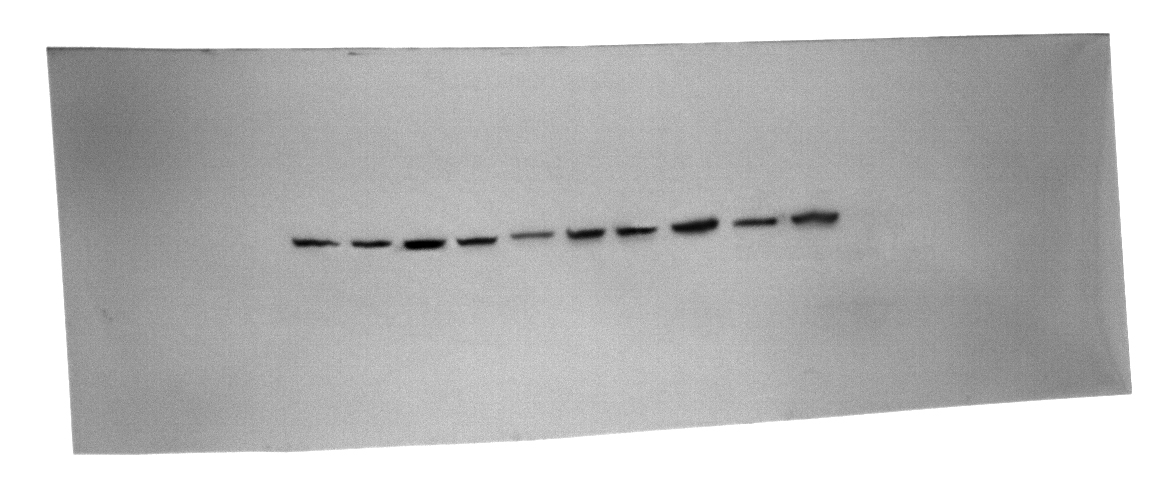

Supplement: Supplementary file 4 [file DataSheet_3.zip › WB/Cell experiments/ACSL4/3/Fig4+Fig5╘¡═╝.jpg]

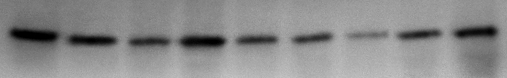

Supplement: Supplementary file 4 [file DataSheet_3.zip › WB/Cell experiments/GPX4/1/Fig2+Fig3.jpg]

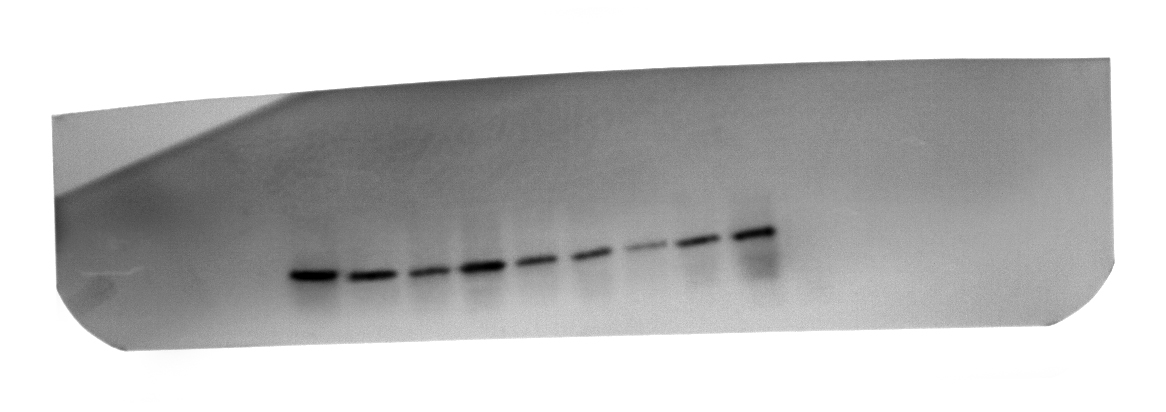

Supplement: Supplementary file 4 [file DataSheet_3.zip › WB/Cell experiments/GPX4/1/Fig2+Fig3╘¡═╝.jpg]

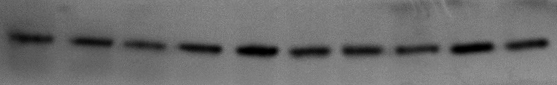

Supplement: Supplementary file 4 [file DataSheet_3.zip › WB/Cell experiments/GPX4/1/Fig4+Fig5.jpg]

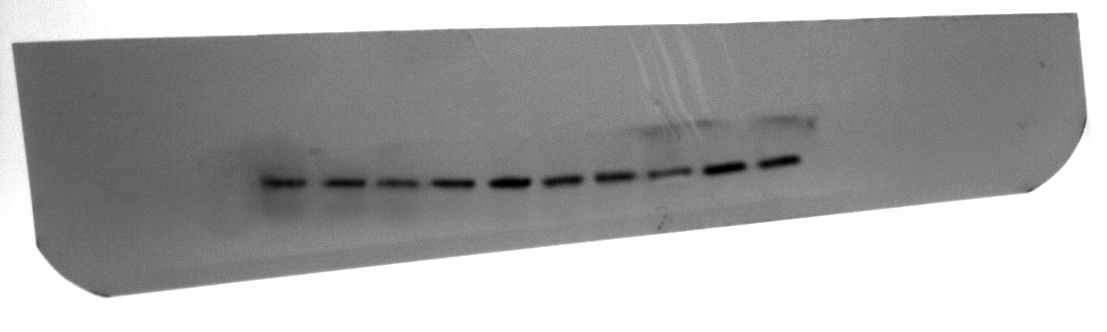

Supplement: Supplementary file 4 [file DataSheet_3.zip › WB/Cell experiments/GPX4/1/Fig4+Fig5╘¡═╝.jpg]

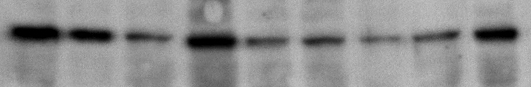

Supplement: Supplementary file 4 [file DataSheet_3.zip › WB/Cell experiments/GPX4/2/Fig2+Fig3.jpg]

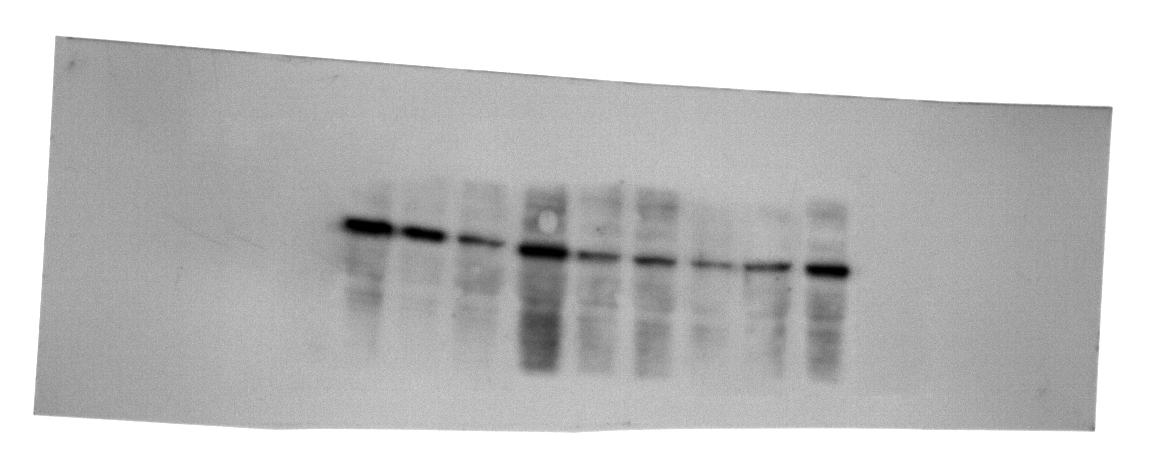

Supplement: Supplementary file 4 [file DataSheet_3.zip › WB/Cell experiments/GPX4/2/Fig2+Fig3╘¡═╝.jpg]

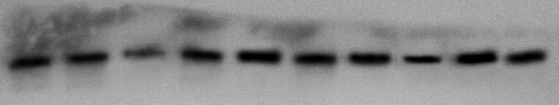

Supplement: Supplementary file 4 [file DataSheet_3.zip › WB/Cell experiments/GPX4/2/Fig4+Fig5.jpg]

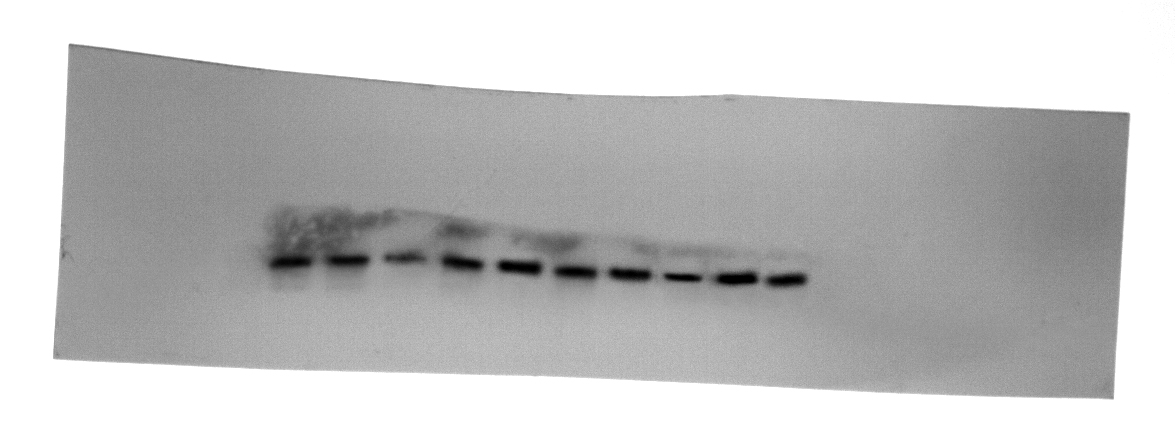

Supplement: Supplementary file 4 [file DataSheet_3.zip › WB/Cell experiments/GPX4/2/Fig4+Fig5╘¡═╝.jpg]

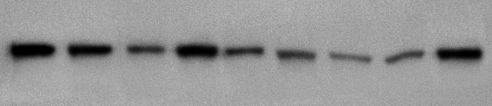

Supplement: Supplementary file 4 [file DataSheet_3.zip › WB/Cell experiments/GPX4/3/Fig2+Fig3.jpg]

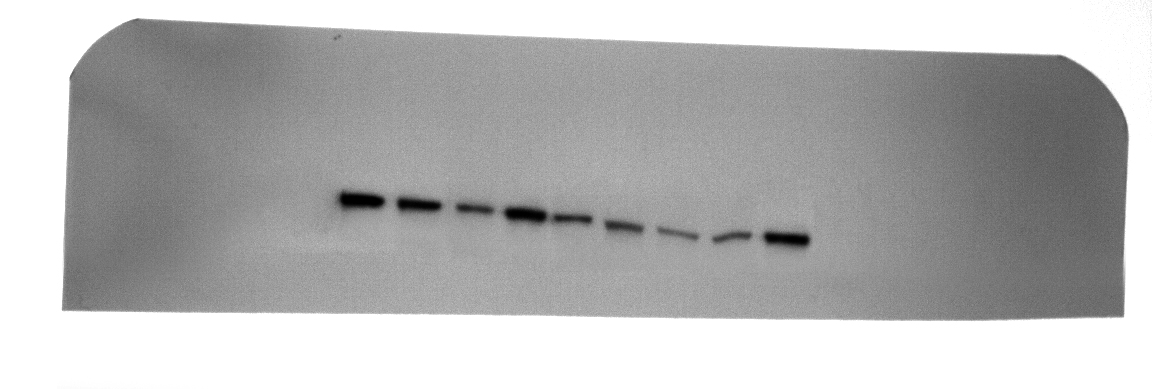

Supplement: Supplementary file 4 [file DataSheet_3.zip › WB/Cell experiments/GPX4/3/Fig2+Fig3╘¡═╝.jpg]

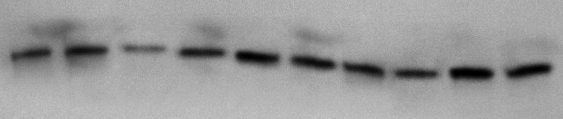

Supplement: Supplementary file 4 [file DataSheet_3.zip › WB/Cell experiments/GPX4/3/Fig4+Fig5.jpg]

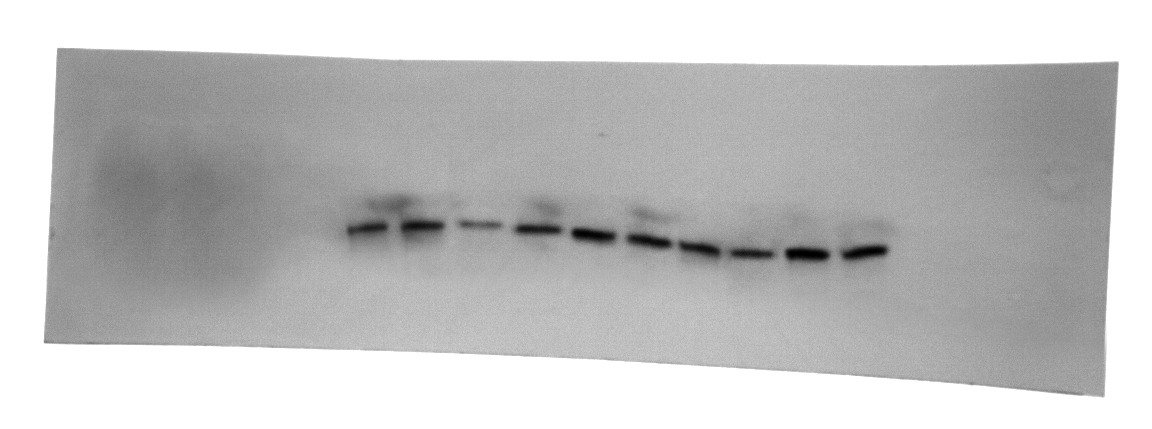

Supplement: Supplementary file 4 [file DataSheet_3.zip › WB/Cell experiments/GPX4/3/Fig4+Fig5╘¡═╝.jgp]

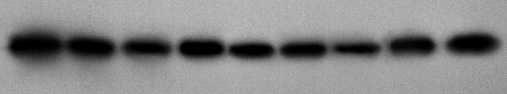

Supplement: Supplementary file 4 [file DataSheet_3.zip › WB/Cell experiments/SLC7A11/1/Fig2+Fig3.jpg]

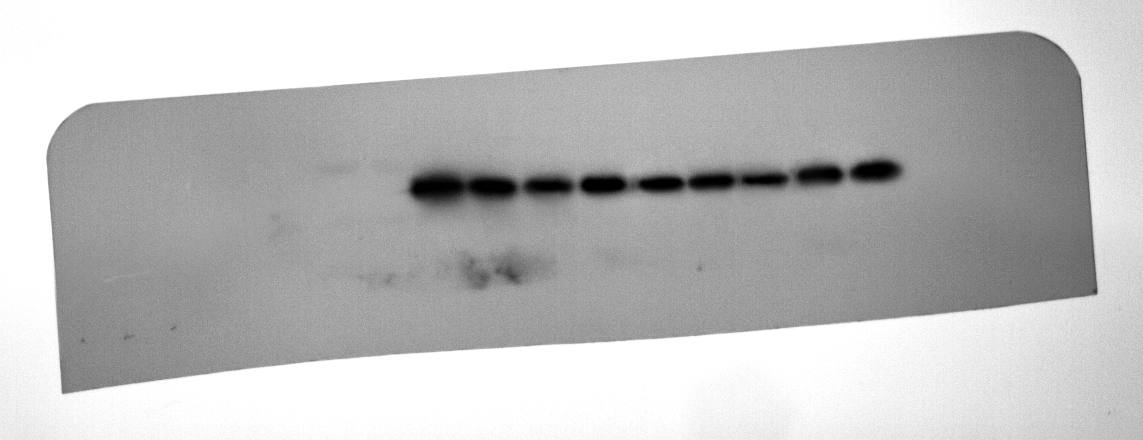

Supplement: Supplementary file 4 [file DataSheet_3.zip › WB/Cell experiments/SLC7A11/1/Fig2+Fig3╘¡═╝.jpg]

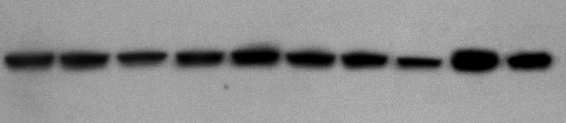

Supplement: Supplementary file 4 [file DataSheet_3.zip › WB/Cell experiments/SLC7A11/1/Fig4+Fig5.jpg]

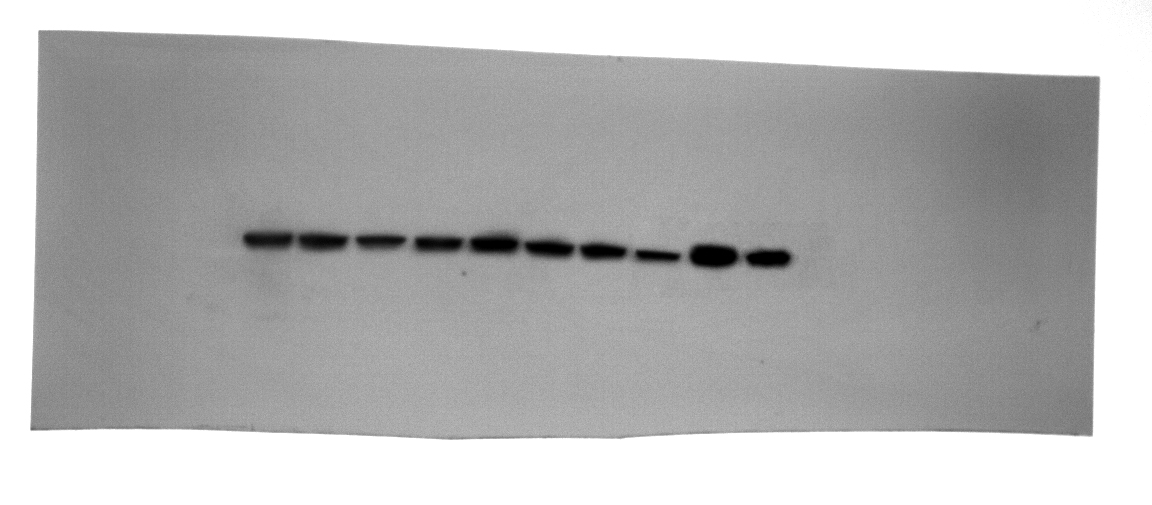

Supplement: Supplementary file 4 [file DataSheet_3.zip › WB/Cell experiments/SLC7A11/1/Fig4+Fig5╘¡═╝.jpg]

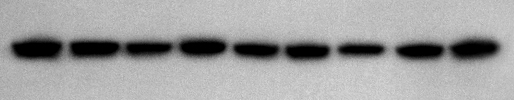

Supplement: Supplementary file 4 [file DataSheet_3.zip › WB/Cell experiments/SLC7A11/2/Fig2+Fig3.jpg]

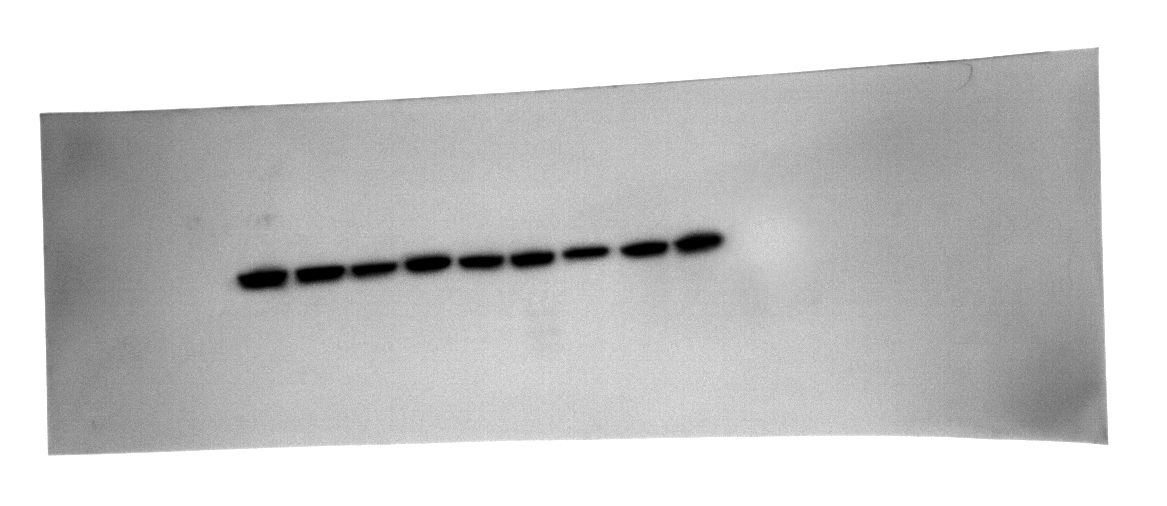

Supplement: Supplementary file 4 [file DataSheet_3.zip › WB/Cell experiments/SLC7A11/2/Fig2+Fig3╘¡═╝.jpg]

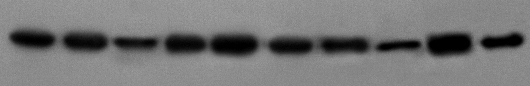

Supplement: Supplementary file 4 [file DataSheet_3.zip › WB/Cell experiments/SLC7A11/2/Fig4+Fig5.jpg]

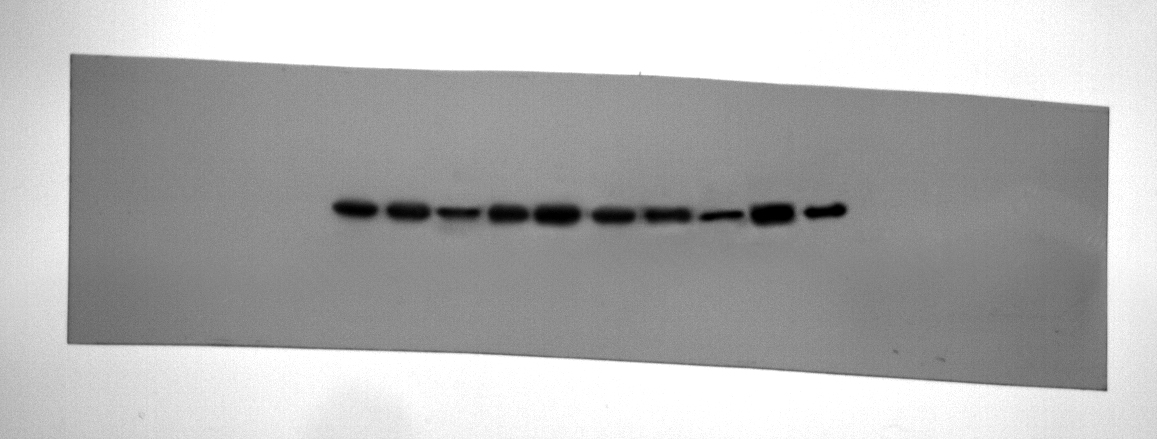

Supplement: Supplementary file 4 [file DataSheet_3.zip › WB/Cell experiments/SLC7A11/2/Fig4+Fig5╘¡═╝.jpg]

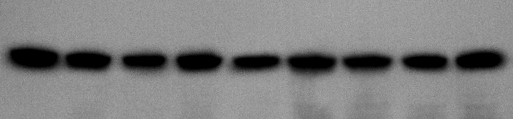

Supplement: Supplementary file 4 [file DataSheet_3.zip › WB/Cell experiments/SLC7A11/3/Fig2+Fig3.jpg]

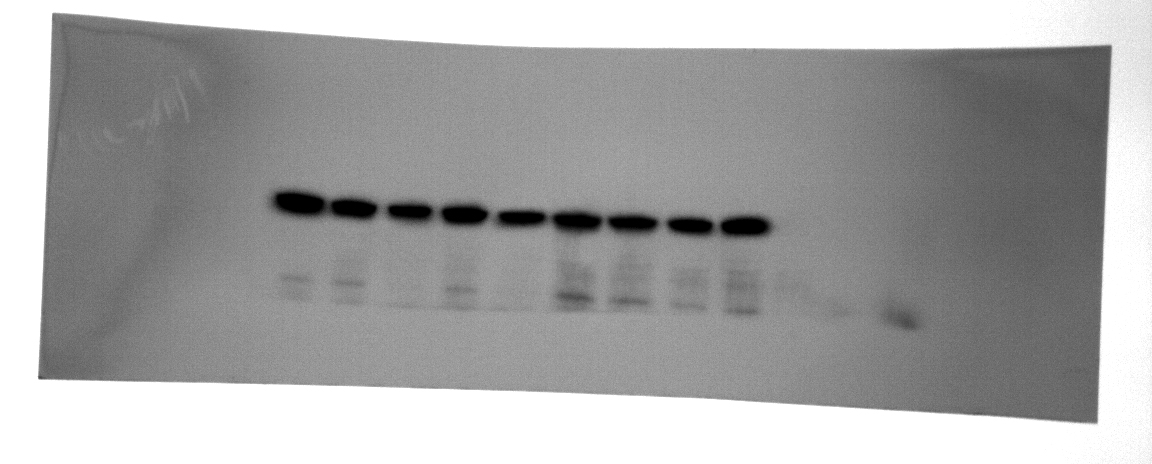

Supplement: Supplementary file 4 [file DataSheet_3.zip › WB/Cell experiments/SLC7A11/3/Fig2+Fig3╘¡═╝.jpg]

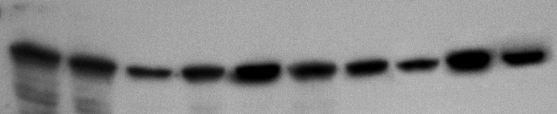

Supplement: Supplementary file 4 [file DataSheet_3.zip › WB/Cell experiments/SLC7A11/3/Fig4+Fig5.jpg]

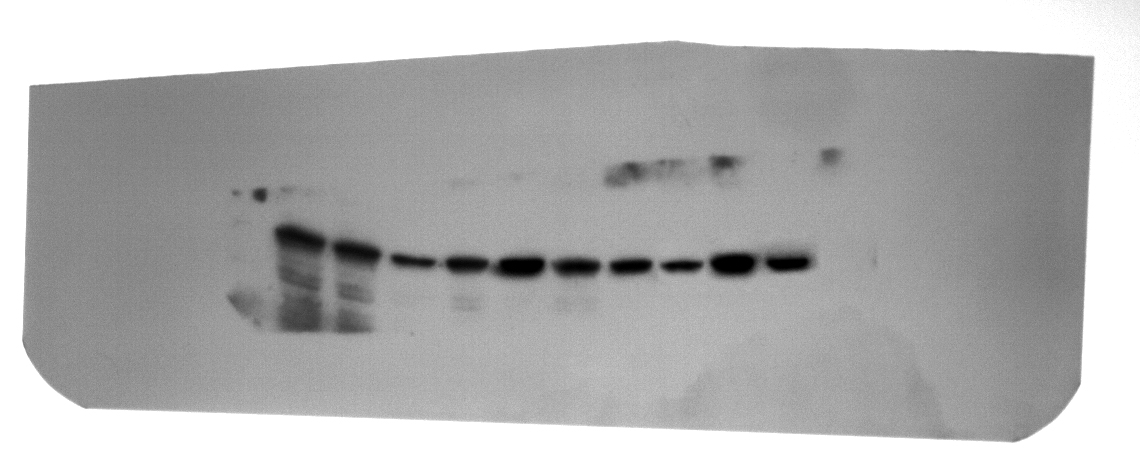

Supplement: Supplementary file 4 [file DataSheet_3.zip › WB/Cell experiments/SLC7A11/3/Fig4+Fig5╘¡═╝.jpg]

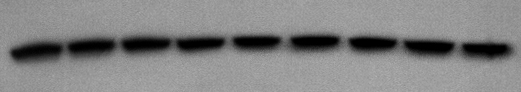

Supplement: Supplementary file 4 [file DataSheet_3.zip › WB/Cell experiments/a┬-actin/1/Fig2+Fig3.jpg]

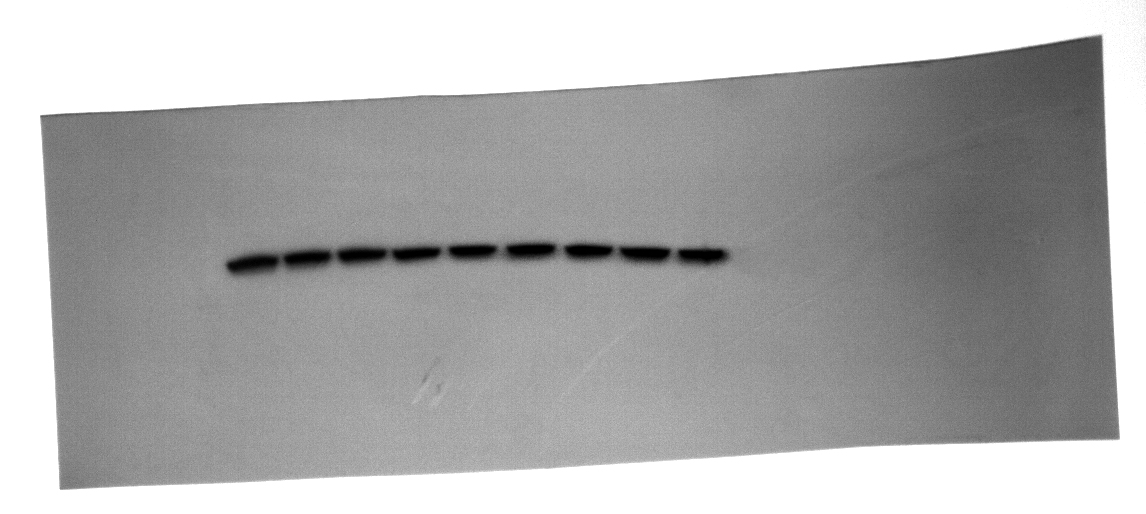

Supplement: Supplementary file 4 [file DataSheet_3.zip › WB/Cell experiments/a┬-actin/1/Fig2+Fig3╘¡═╝.jpg]

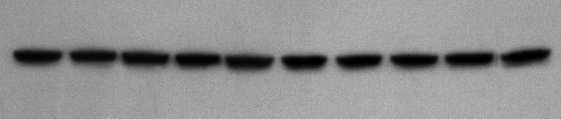

Supplement: Supplementary file 4 [file DataSheet_3.zip › WB/Cell experiments/a┬-actin/1/Fig4+Fig5.jpg]

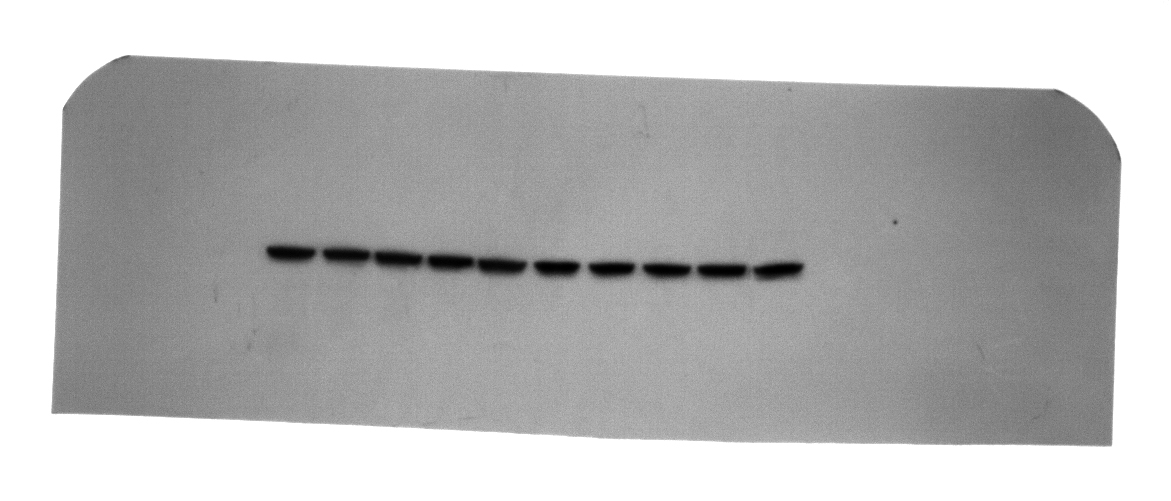

Supplement: Supplementary file 4 [file DataSheet_3.zip › WB/Cell experiments/a┬-actin/1/Fig4+Fig5╘¡═╝.jpg]

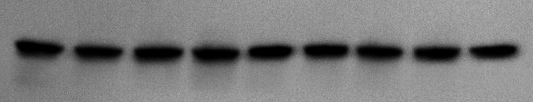

Supplement: Supplementary file 4 [file DataSheet_3.zip › WB/Cell experiments/a┬-actin/2/Fig2+Fig3.jpg]

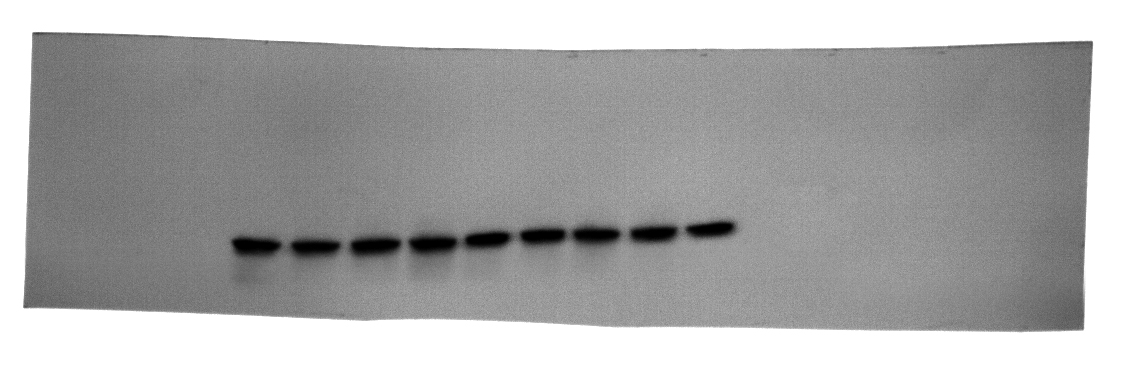

Supplement: Supplementary file 4 [file DataSheet_3.zip › WB/Cell experiments/a┬-actin/2/Fig2+Fig3╘¡═╝.jpg]

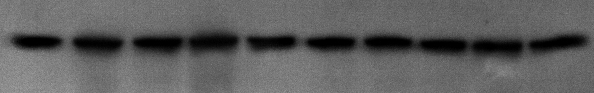

Supplement: Supplementary file 4 [file DataSheet_3.zip › WB/Cell experiments/a┬-actin/2/Fig4+Fig5.jpg]

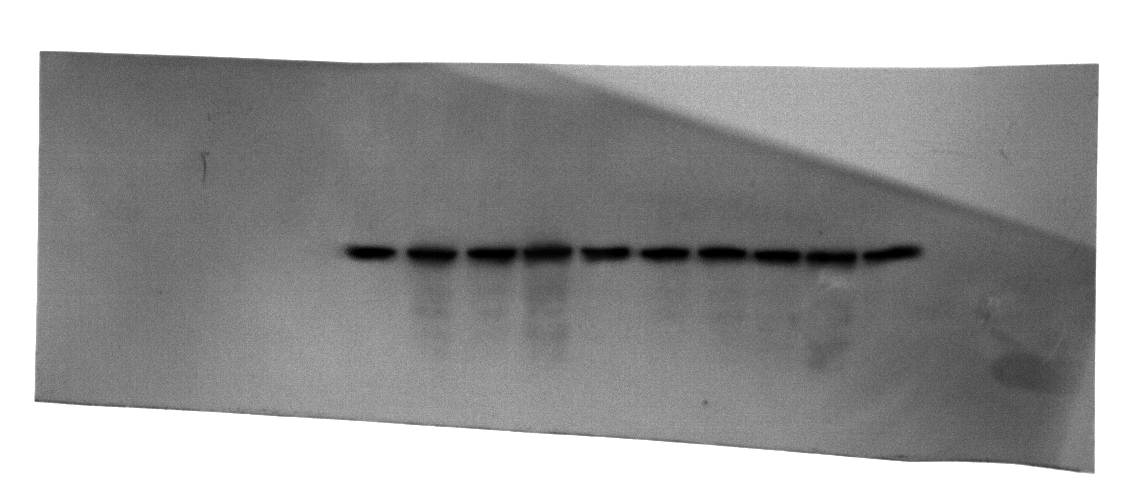

Supplement: Supplementary file 4 [file DataSheet_3.zip › WB/Cell experiments/a┬-actin/2/Fig4+Fig5╘¡═╝.jpg]

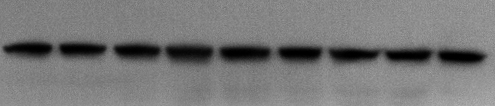

Supplement: Supplementary file 4 [file DataSheet_3.zip › WB/Cell experiments/a┬-actin/3/Fig2+Fig3.jpg]

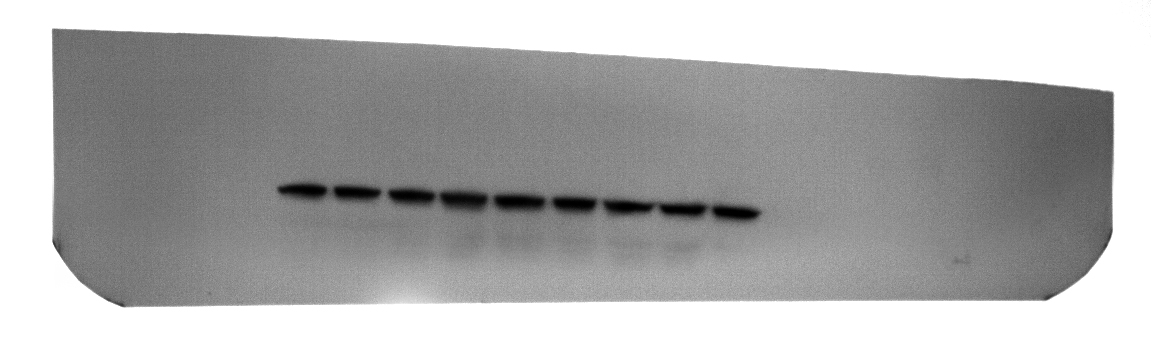

Supplement: Supplementary file 4 [file DataSheet_3.zip › WB/Cell experiments/a┬-actin/3/Fig2+Fig3╘¡═╝.jpg]

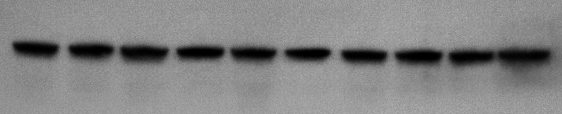

Supplement: Supplementary file 4 [file DataSheet_3.zip › WB/Cell experiments/a┬-actin/3/Fig4+Fig5.jpg]

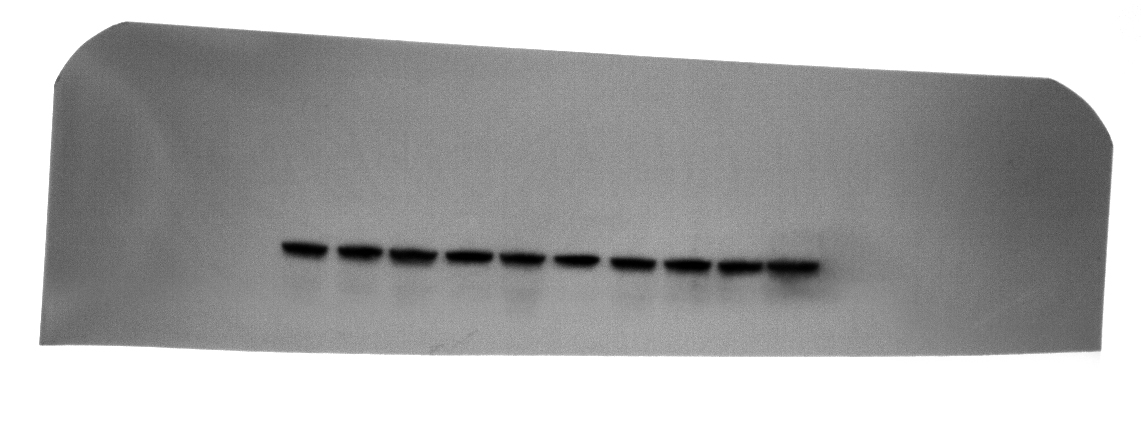

Supplement: Supplementary file 4 [file DataSheet_3.zip › WB/Cell experiments/a┬-actin/3/Fig4+Fig5╘¡═╝.jpg]

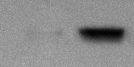

Supplement: Supplementary file 4 [file DataSheet_3.zip › WB/Exosomes/CD63.jpg]

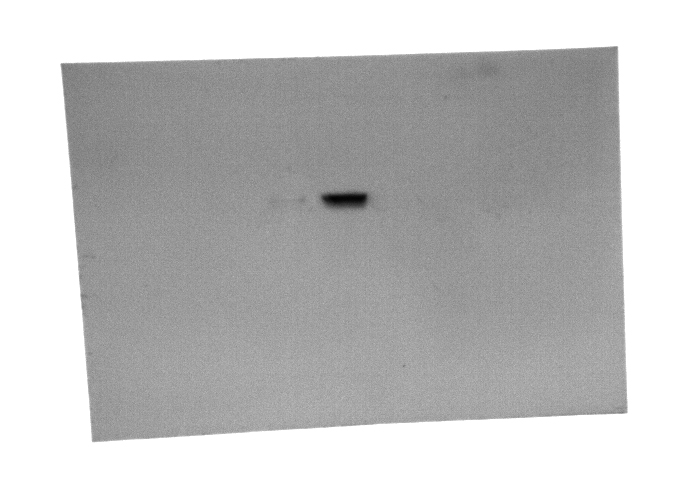

Supplement: Supplementary file 4 [file DataSheet_3.zip › WB/Exosomes/CD63╘¡═╝.jpg]

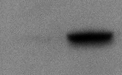

Supplement: Supplementary file 4 [file DataSheet_3.zip › WB/Exosomes/CD81.jpg]

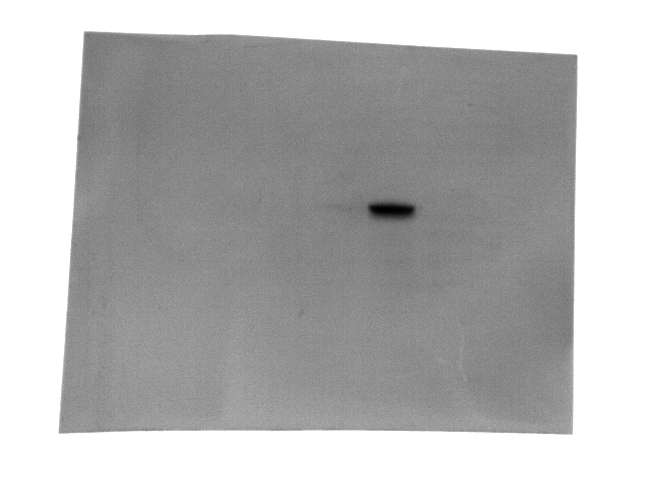

Supplement: Supplementary file 4 [file DataSheet_3.zip › WB/Exosomes/CD81╘¡═╝.jpg]

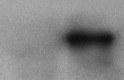

Supplement: Supplementary file 4 [file DataSheet_3.zip › WB/Exosomes/CD9.jpg]

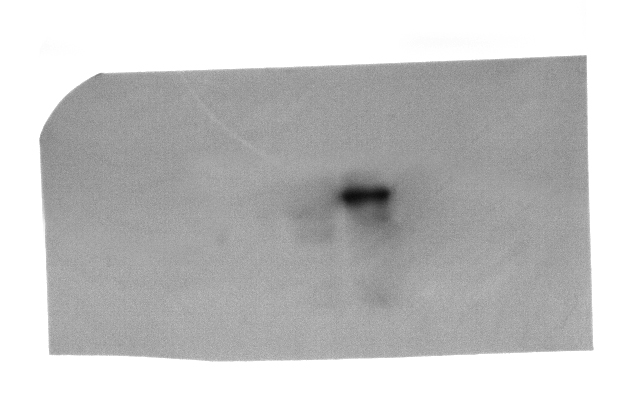

Supplement: Supplementary file 4 [file DataSheet_3.zip › WB/Exosomes/CD9╘¡═╝.jpg]

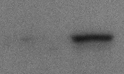

Supplement: Supplementary file 4 [file DataSheet_3.zip › WB/Exosomes/TSG101.jpg]

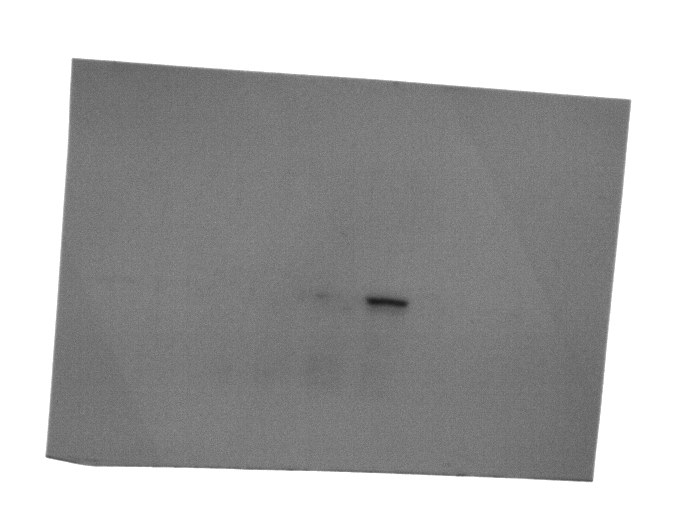

Supplement: Supplementary file 4 [file DataSheet_3.zip › WB/Exosomes/TSG101╘¡═╝.jpg]

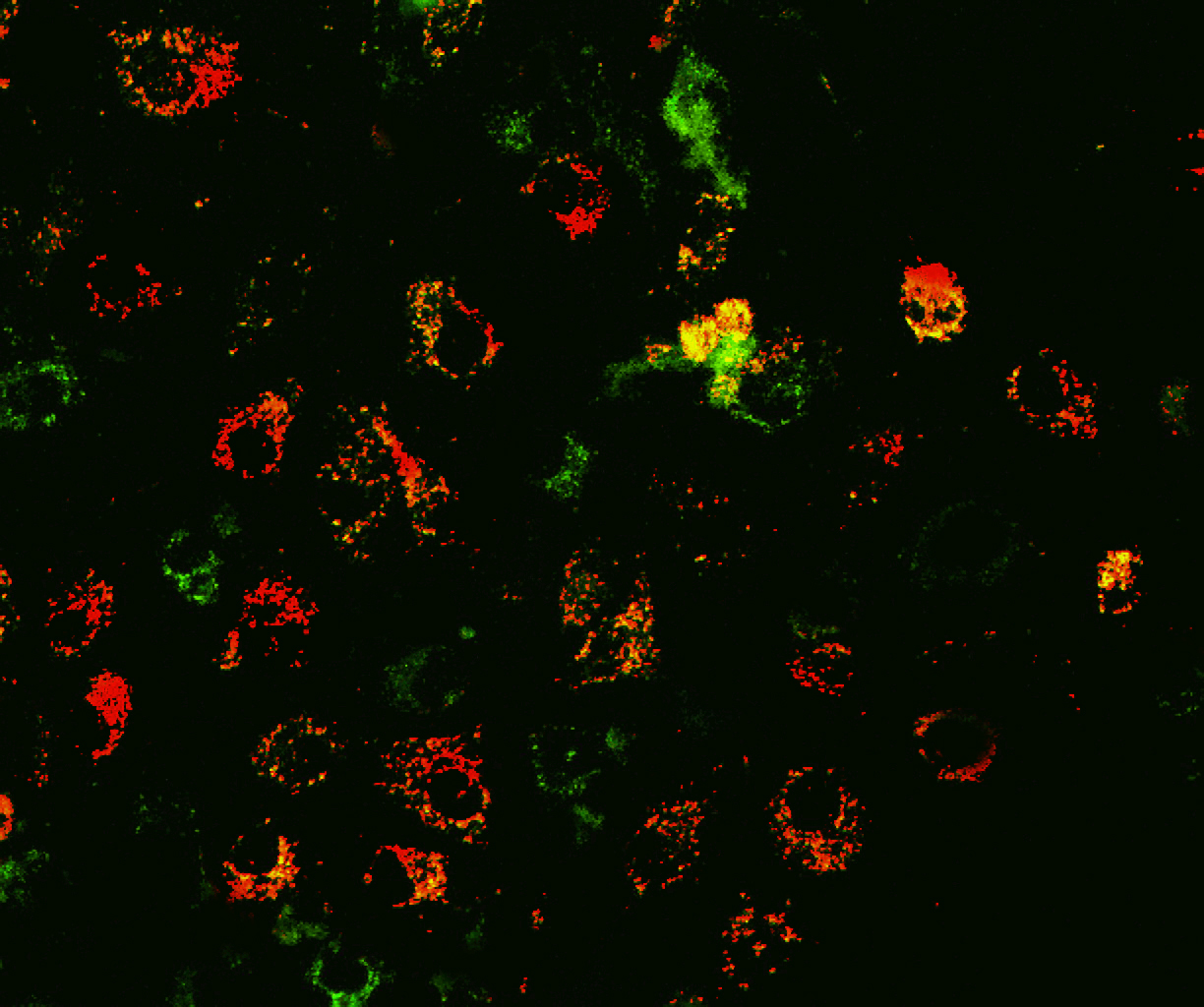

Supplement: Supplementary file 5 [file DataSheet_4.zip › FIG2/IL-1a┬/1/merge.jpg]

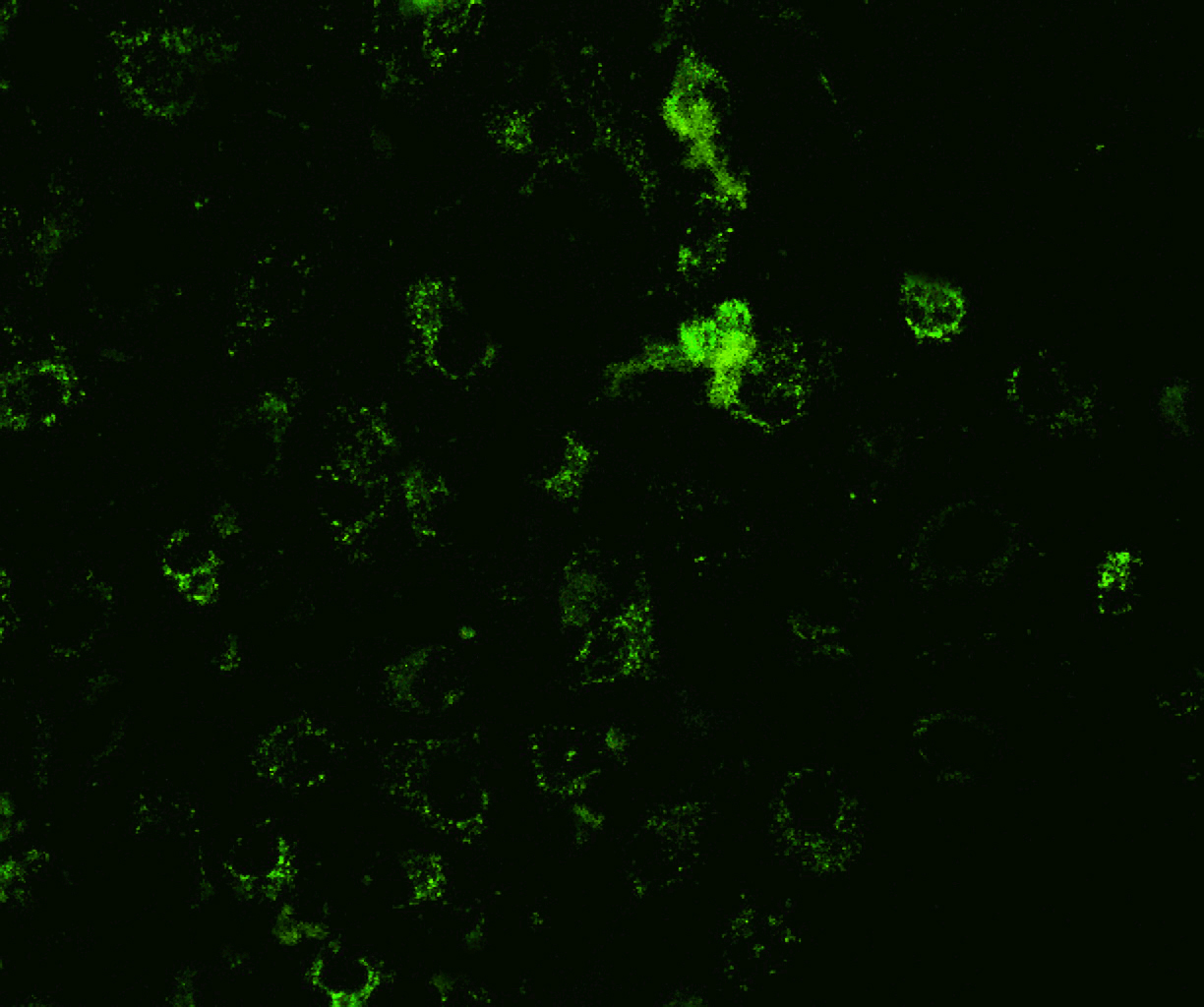

Supplement: Supplementary file 5 [file DataSheet_4.zip › FIG2/IL-1a┬/1/mono.jpg]

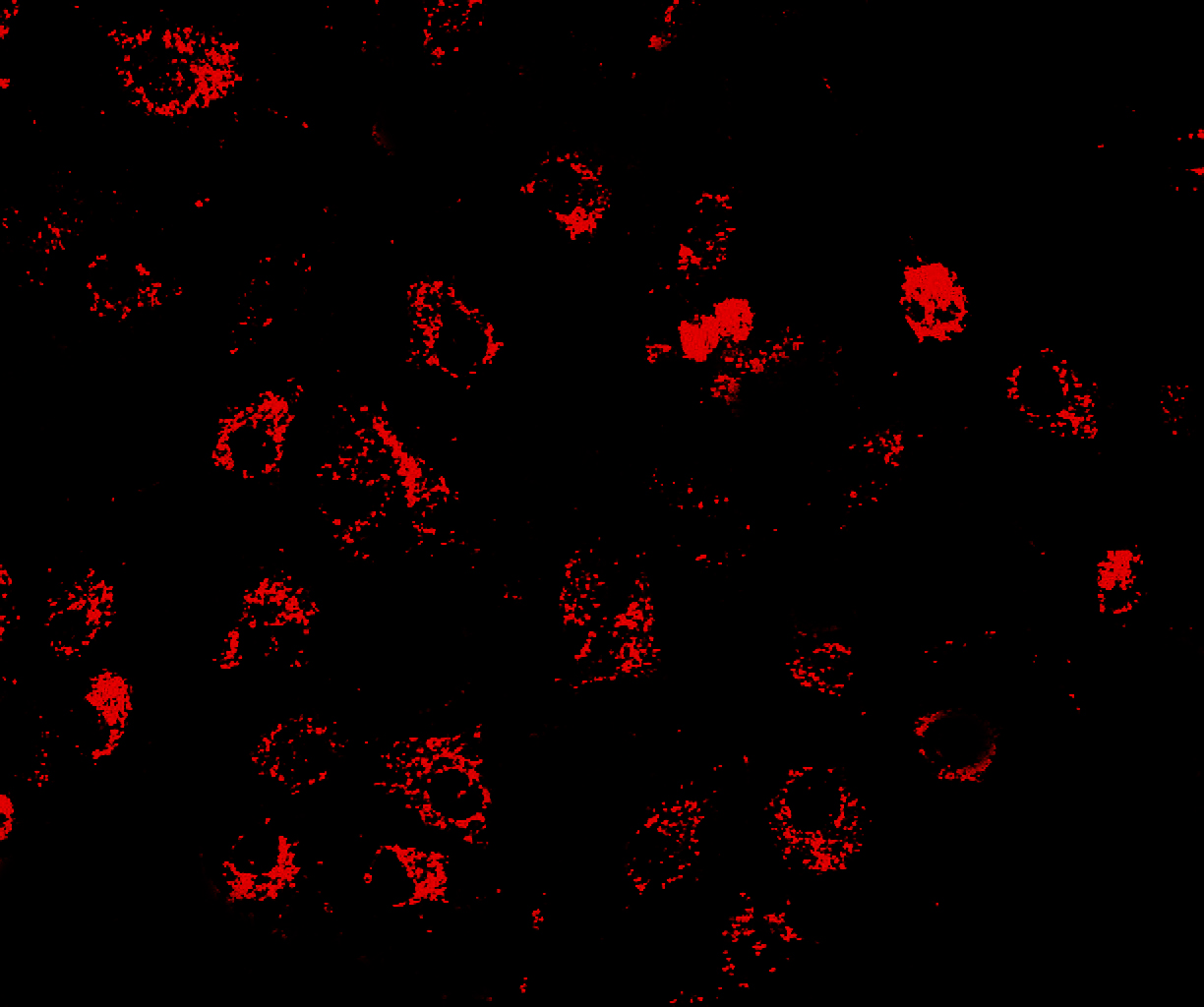

Supplement: Supplementary file 5 [file DataSheet_4.zip › FIG2/IL-1a┬/1/poly.jpg]

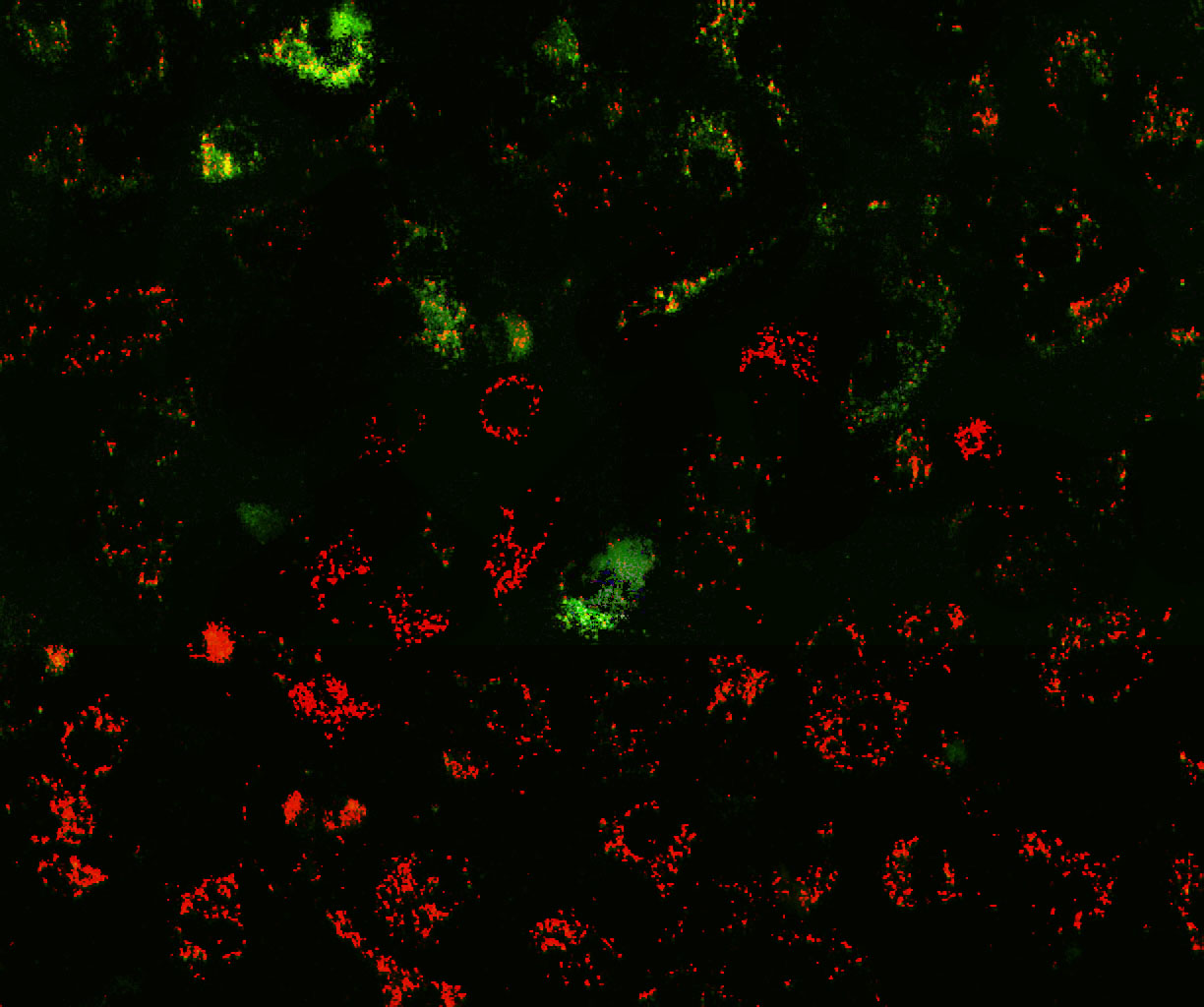

Supplement: Supplementary file 5 [file DataSheet_4.zip › FIG2/IL-1a┬/2/merge.jpg]

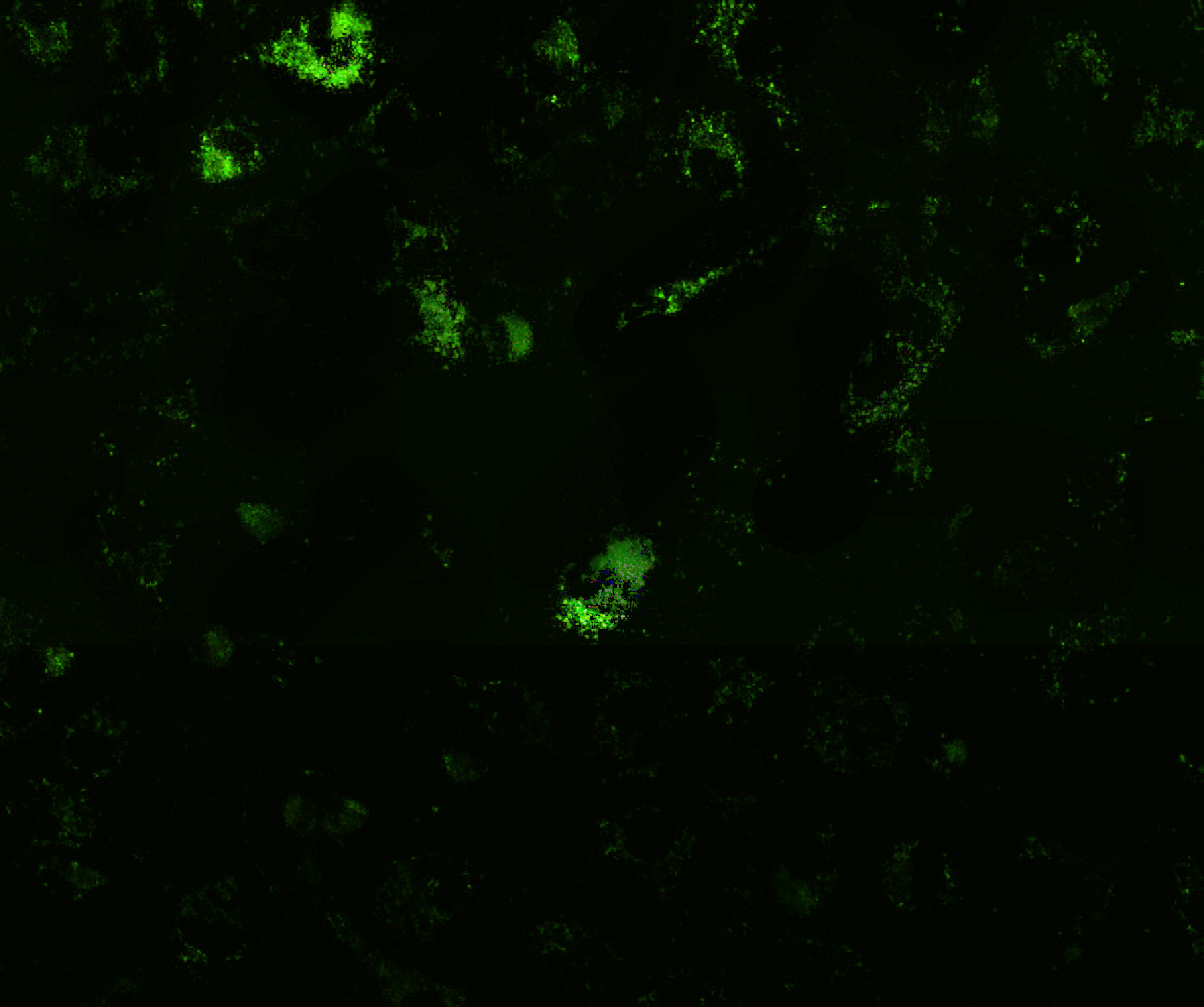

Supplement: Supplementary file 5 [file DataSheet_4.zip › FIG2/IL-1a┬/2/mono.jpg]

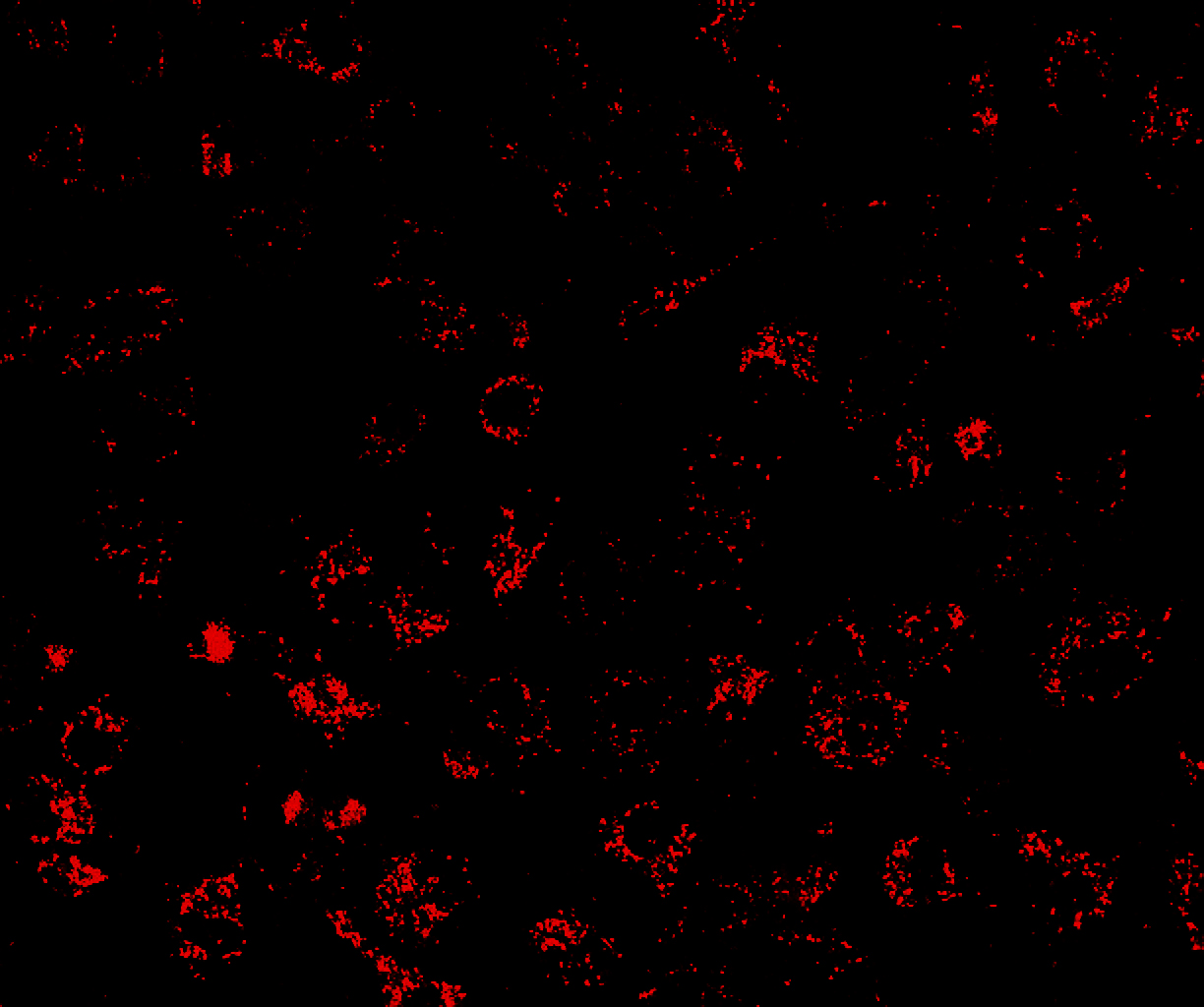

Supplement: Supplementary file 5 [file DataSheet_4.zip › FIG2/IL-1a┬/2/poly.jpg]

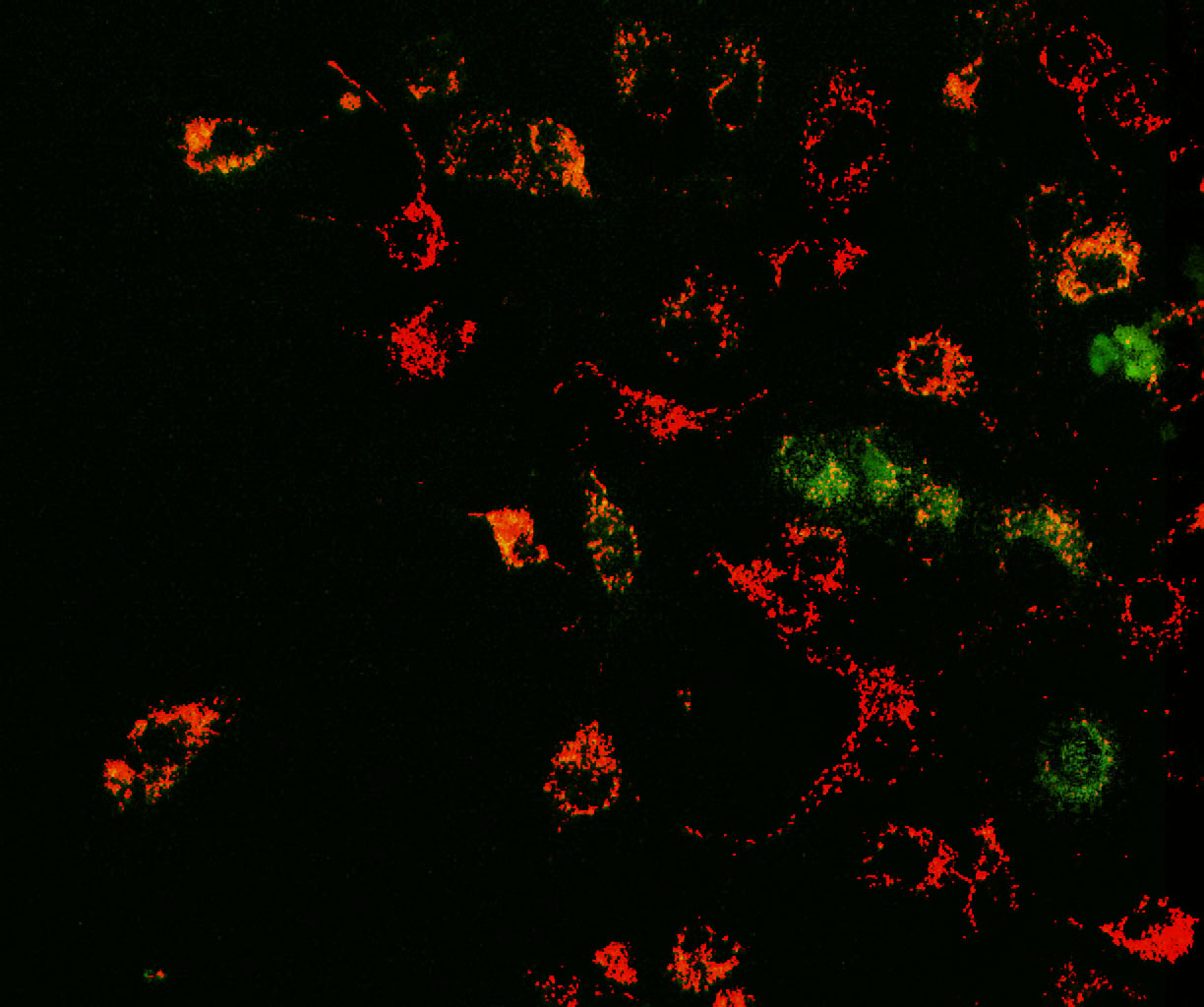

Supplement: Supplementary file 5 [file DataSheet_4.zip › FIG2/IL-1a┬/3/merge.jpg]

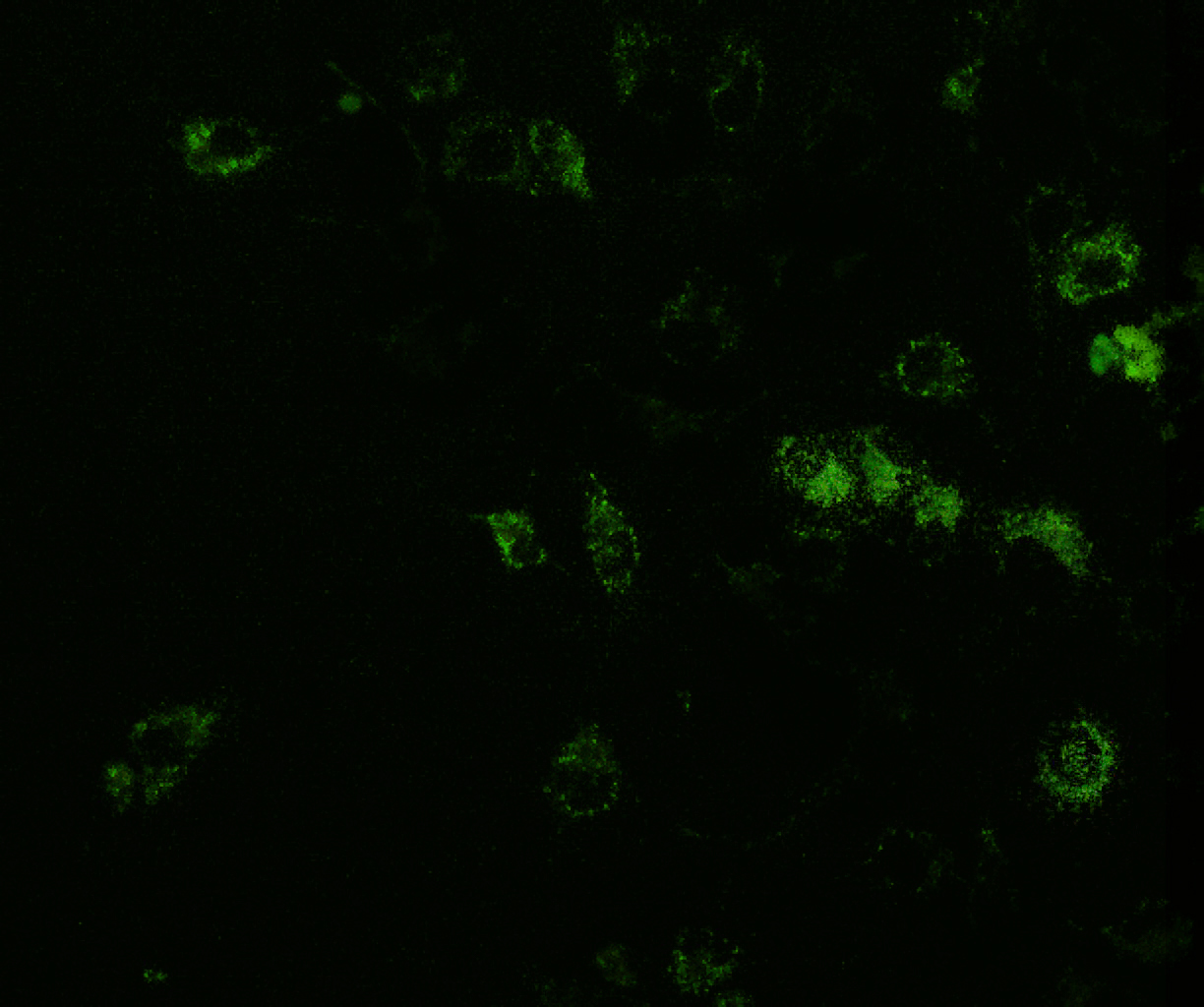

Supplement: Supplementary file 5 [file DataSheet_4.zip › FIG2/IL-1a┬/3/mono.jpg]

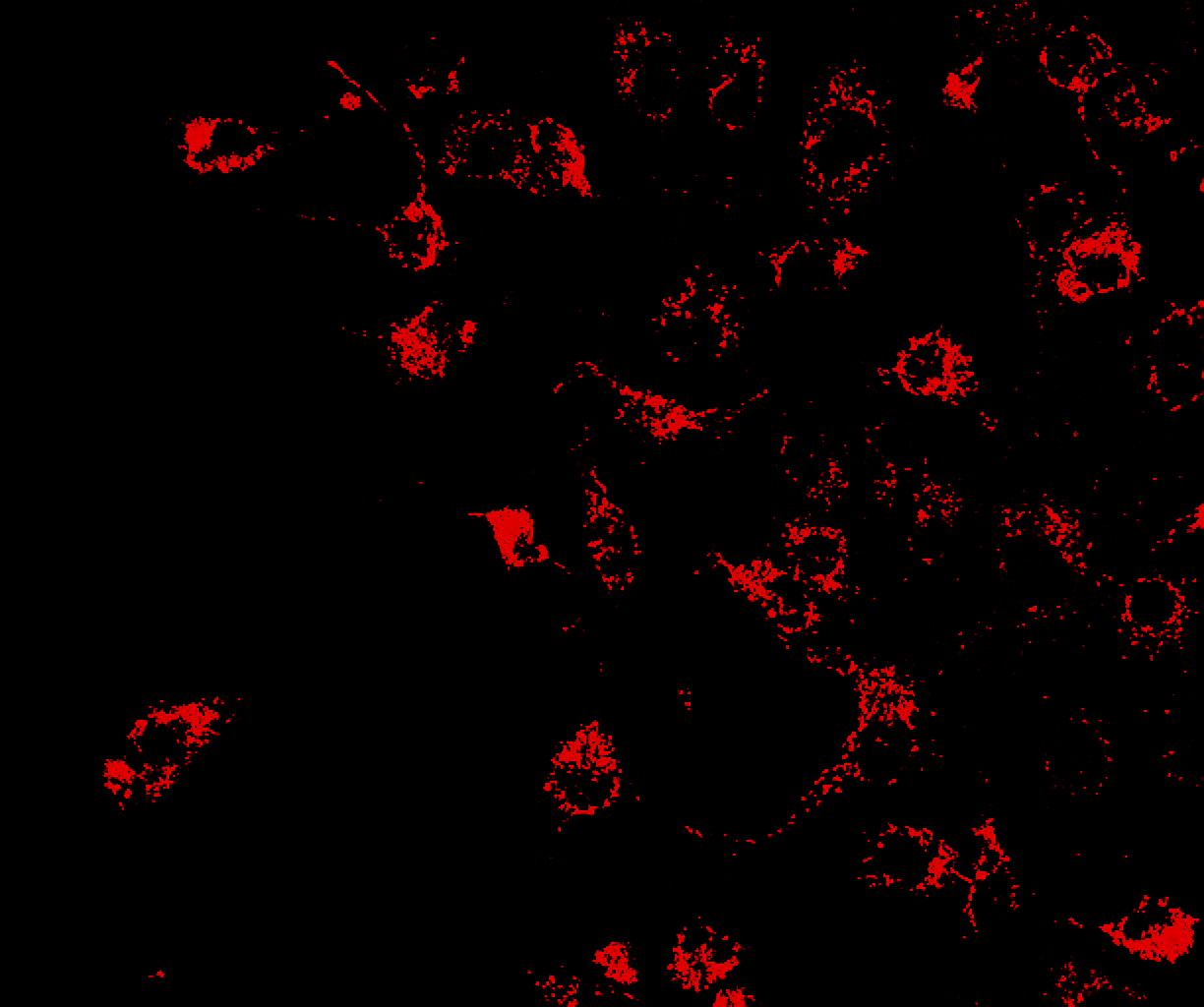

Supplement: Supplementary file 5 [file DataSheet_4.zip › FIG2/IL-1a┬/3/poly.jpg]

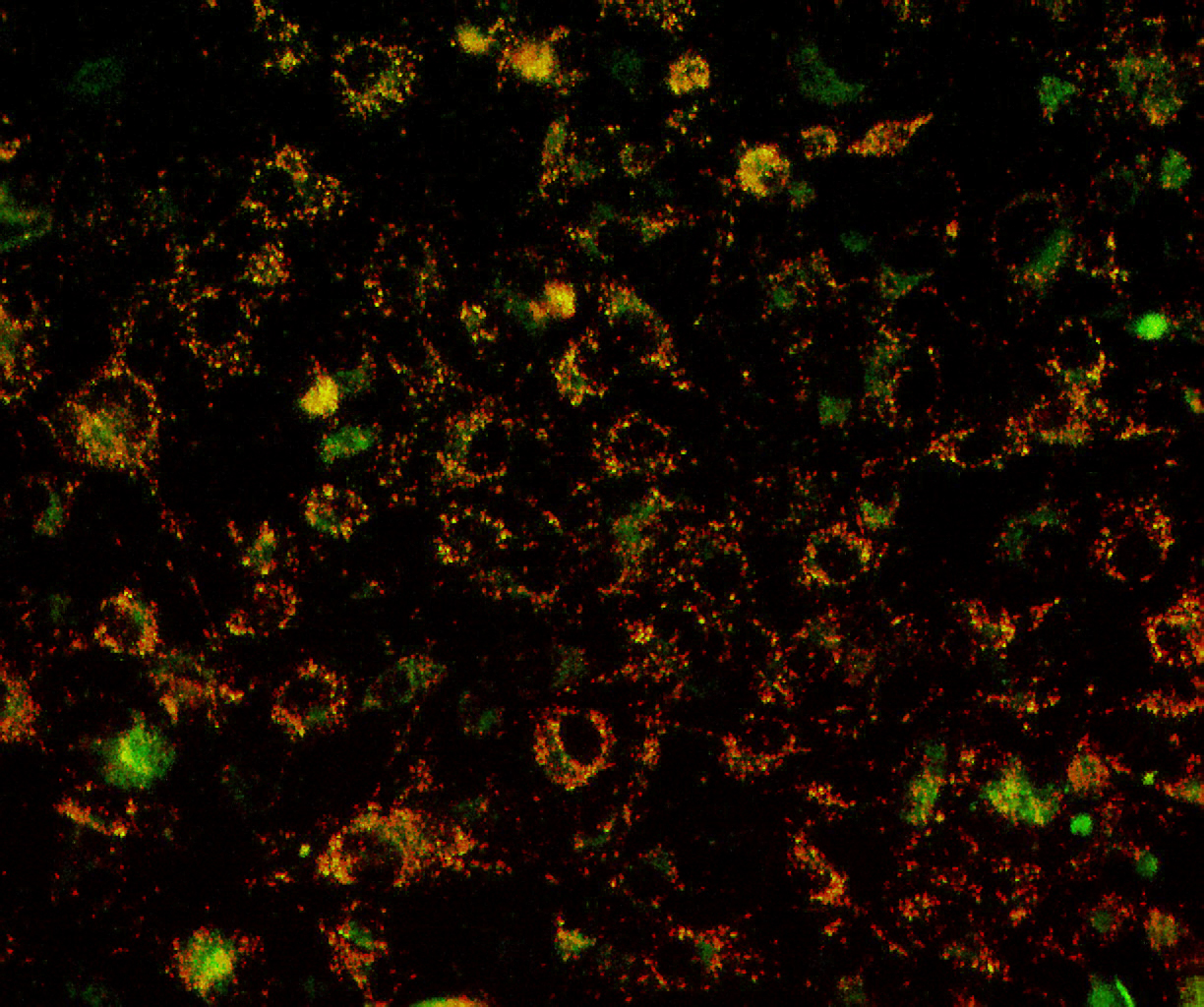

Supplement: Supplementary file 5 [file DataSheet_4.zip › FIG2/IL-1a┬+Exo/1/merge.jpg]

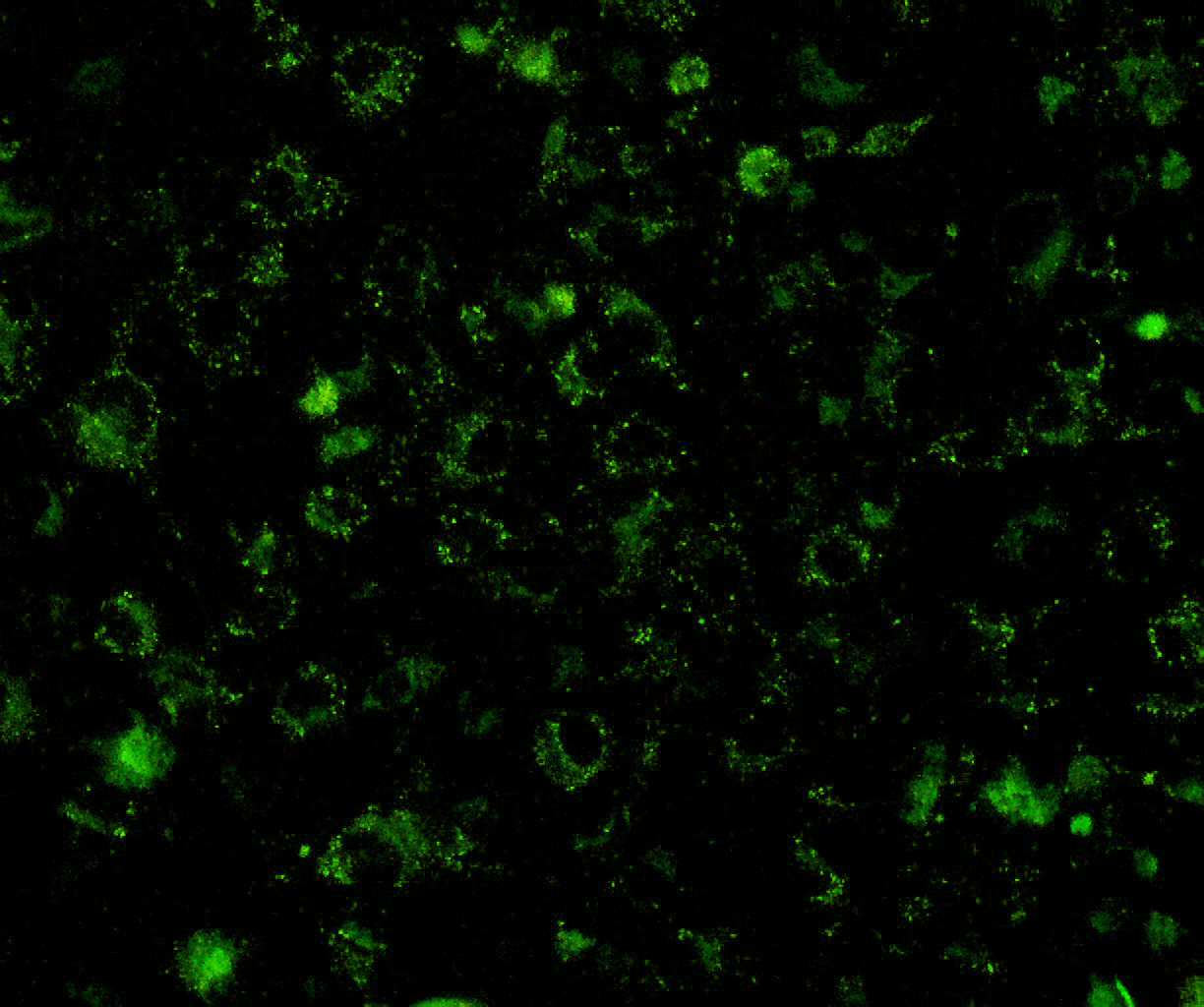

Supplement: Supplementary file 5 [file DataSheet_4.zip › FIG2/IL-1a┬+Exo/1/mono.jpg]

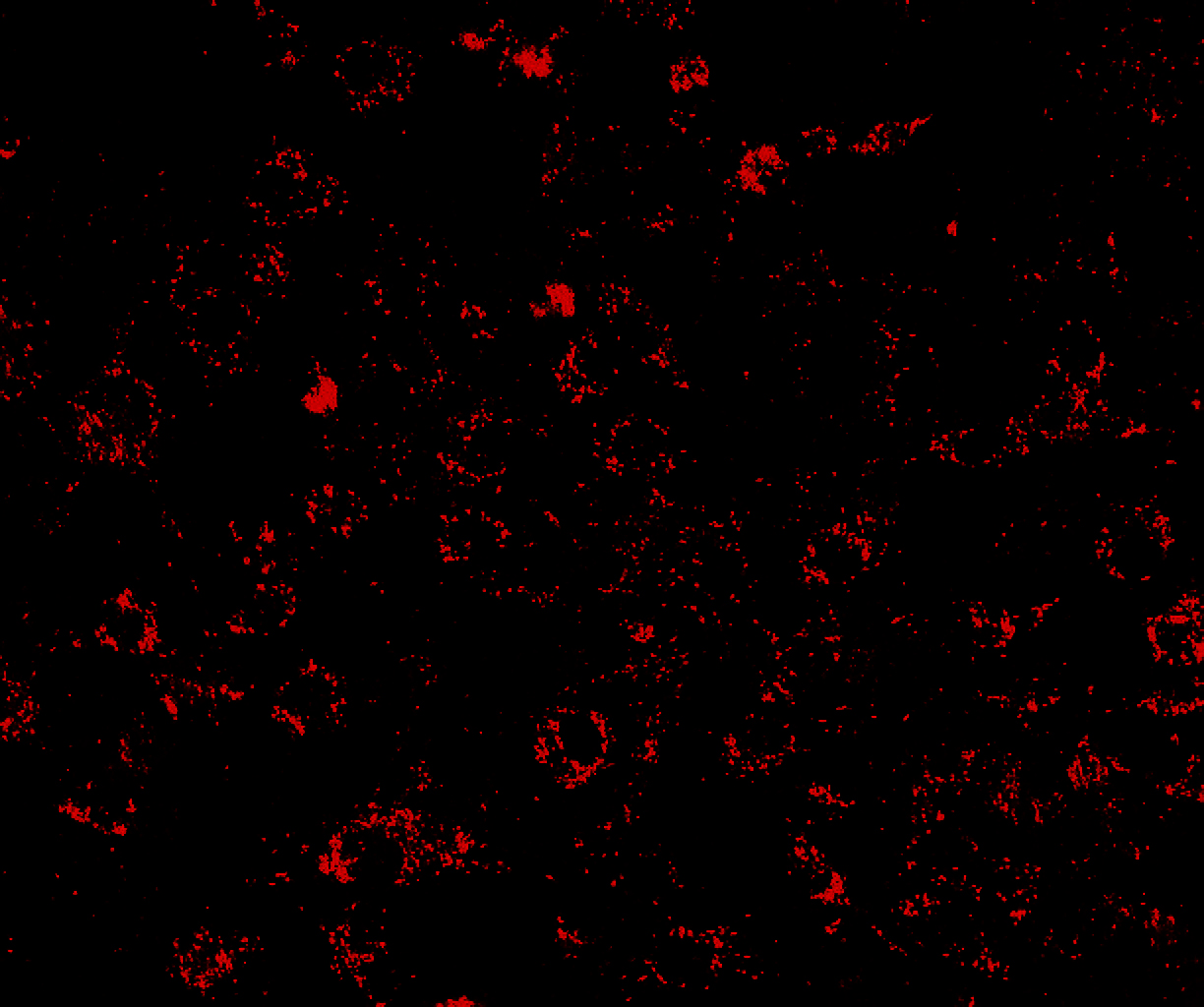

Supplement: Supplementary file 5 [file DataSheet_4.zip › FIG2/IL-1a┬+Exo/1/poly.jpg]

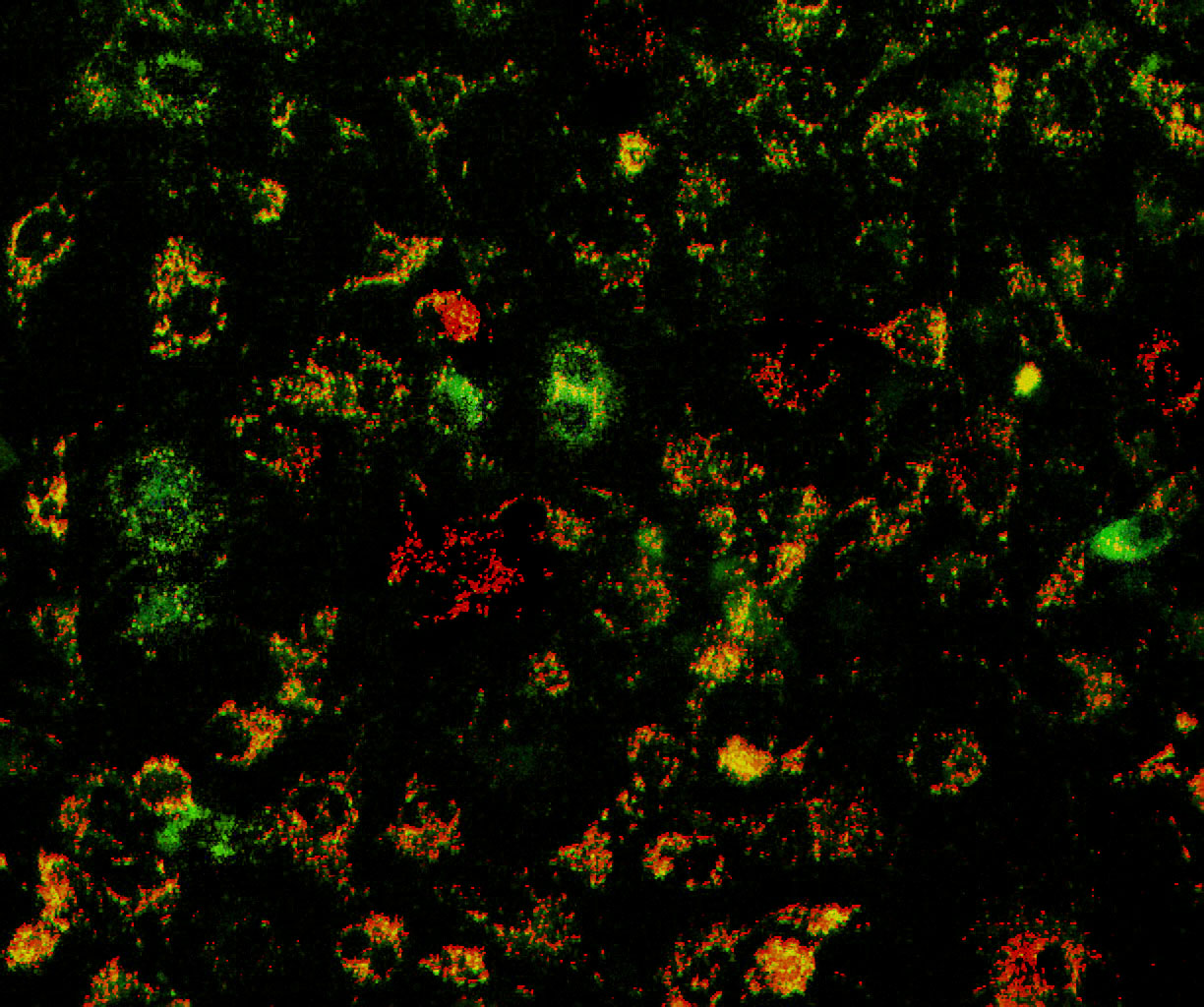

Supplement: Supplementary file 5 [file DataSheet_4.zip › FIG2/IL-1a┬+Exo/2/merge.jpg]

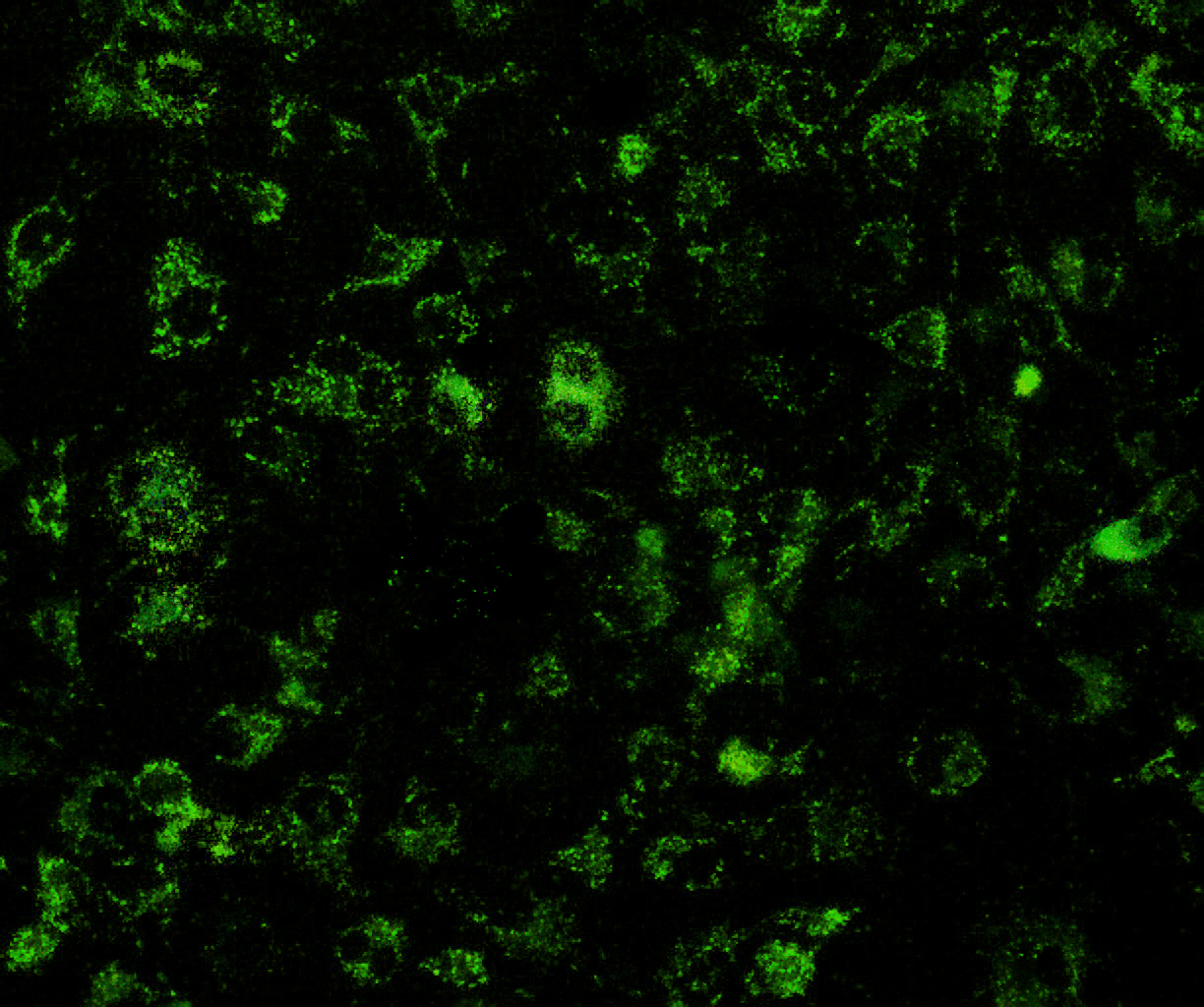

Supplement: Supplementary file 5 [file DataSheet_4.zip › FIG2/IL-1a┬+Exo/2/mono.jpg]

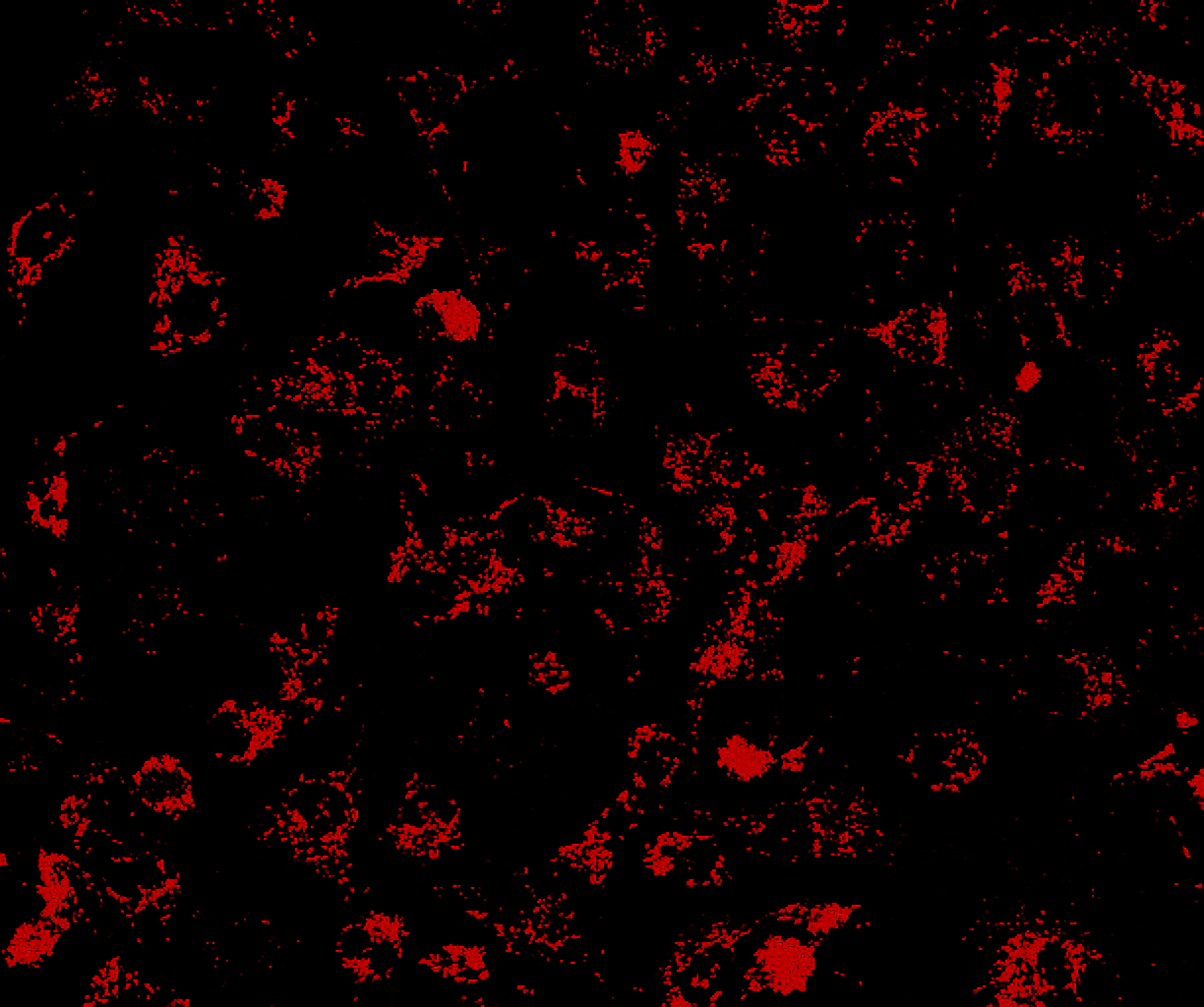

Supplement: Supplementary file 5 [file DataSheet_4.zip › FIG2/IL-1a┬+Exo/2/poly.jpg]

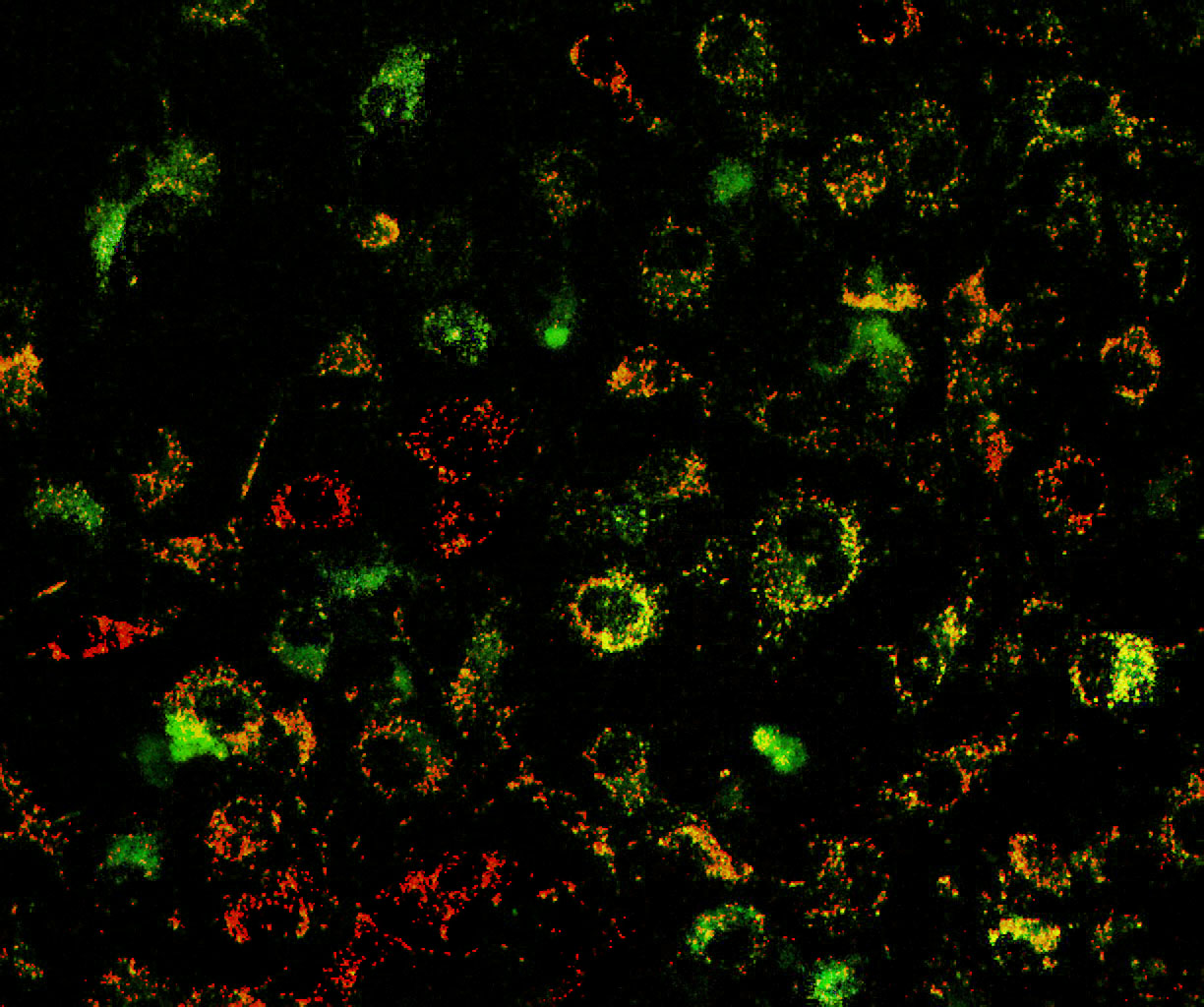

Supplement: Supplementary file 5 [file DataSheet_4.zip › FIG2/IL-1a┬+Exo/3/merge.jpg]

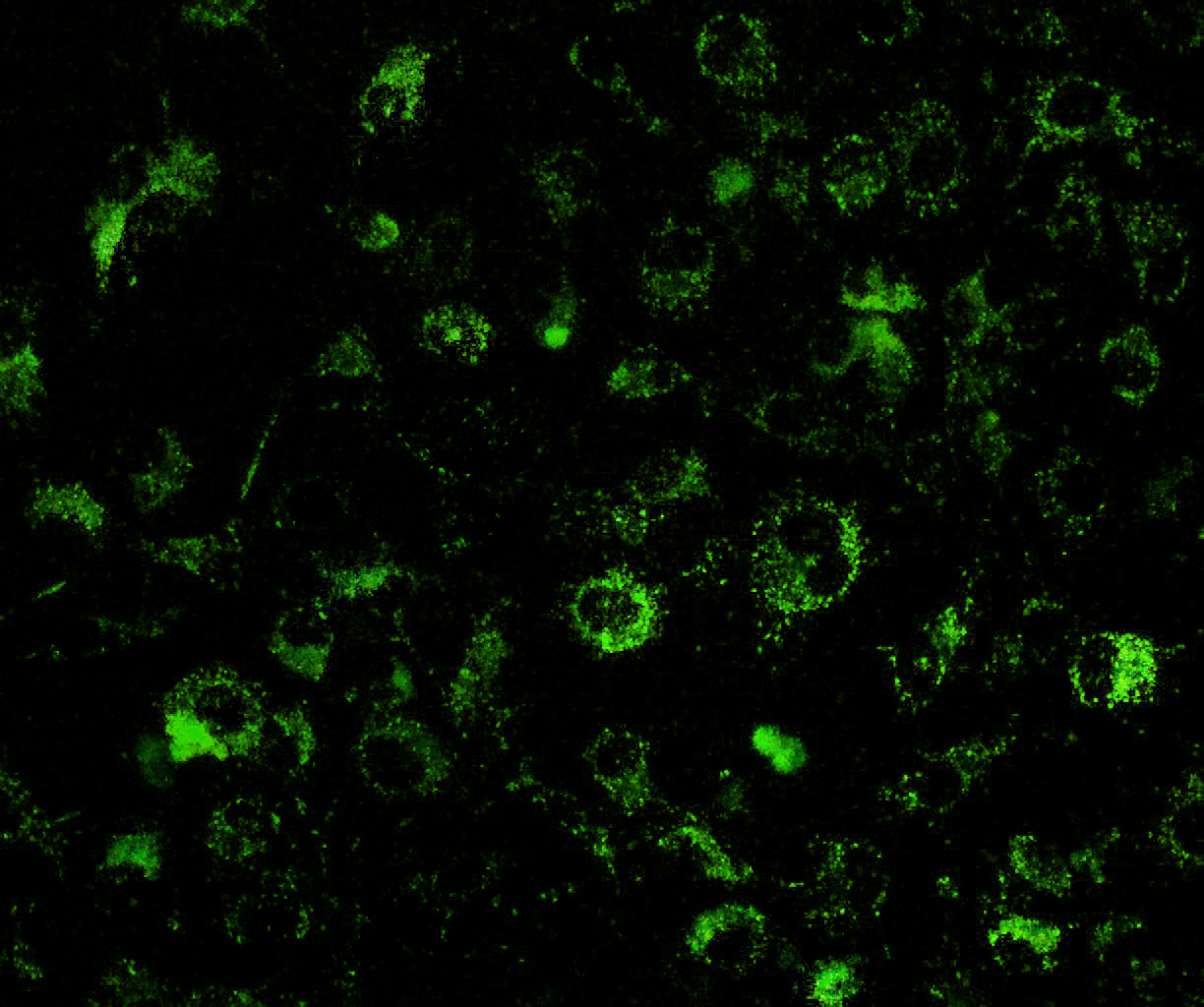

Supplement: Supplementary file 5 [file DataSheet_4.zip › FIG2/IL-1a┬+Exo/3/mono.jpg]

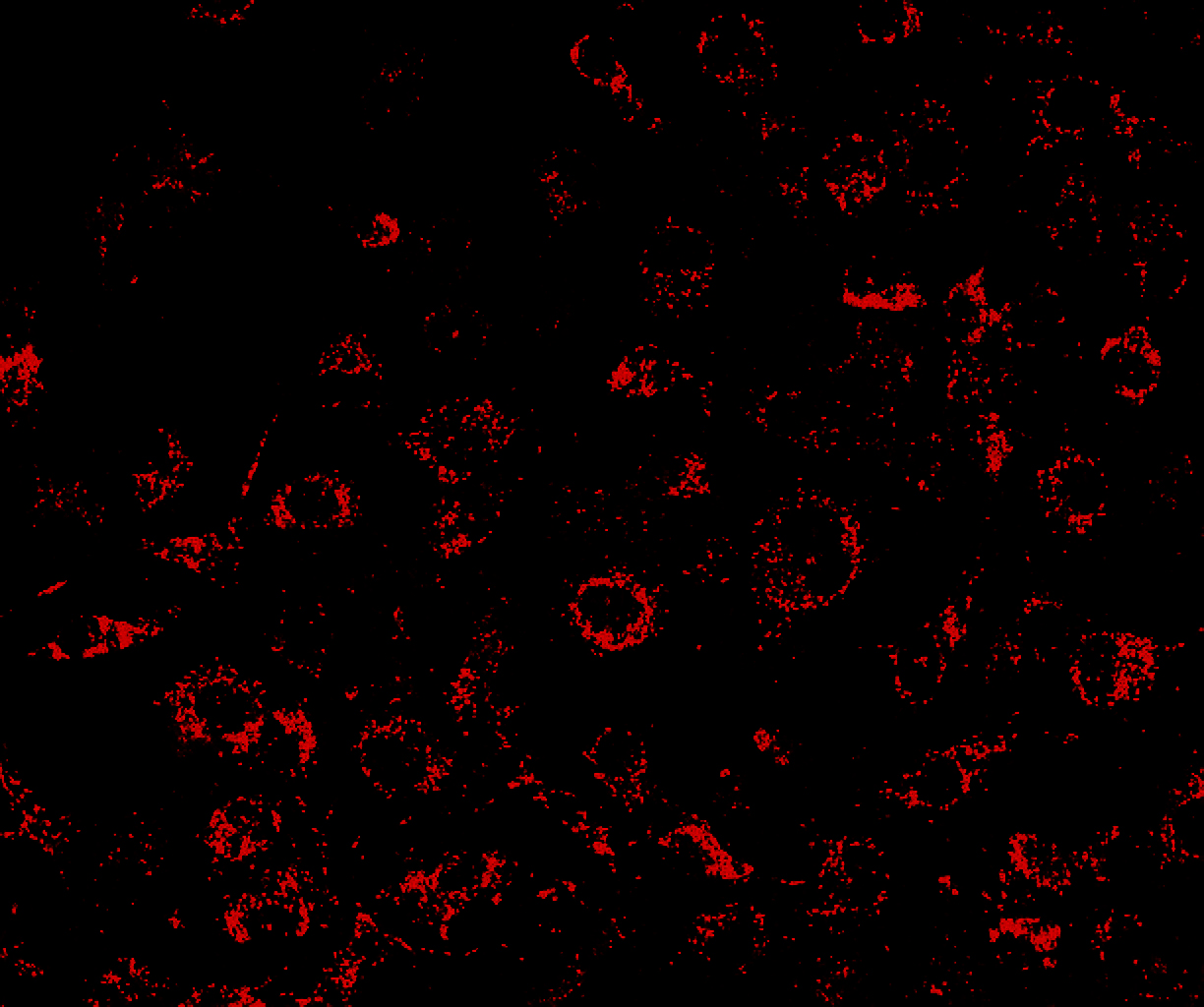

Supplement: Supplementary file 5 [file DataSheet_4.zip › FIG2/IL-1a┬+Exo/3/poly.jpg]

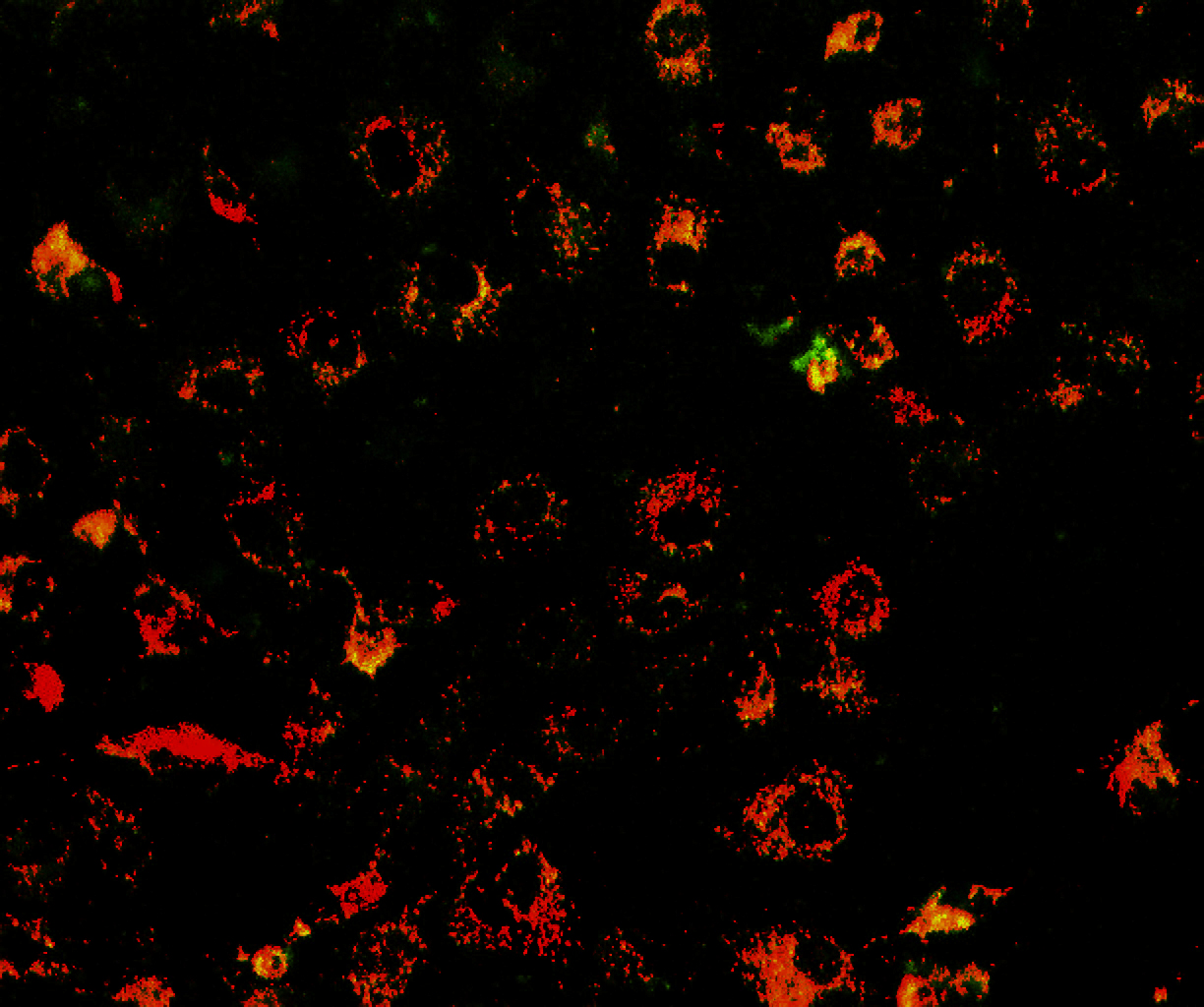

Supplement: Supplementary file 5 [file DataSheet_4.zip › FIG2/IL-1a┬+Exo+Fer-1/1/merge.jpg]

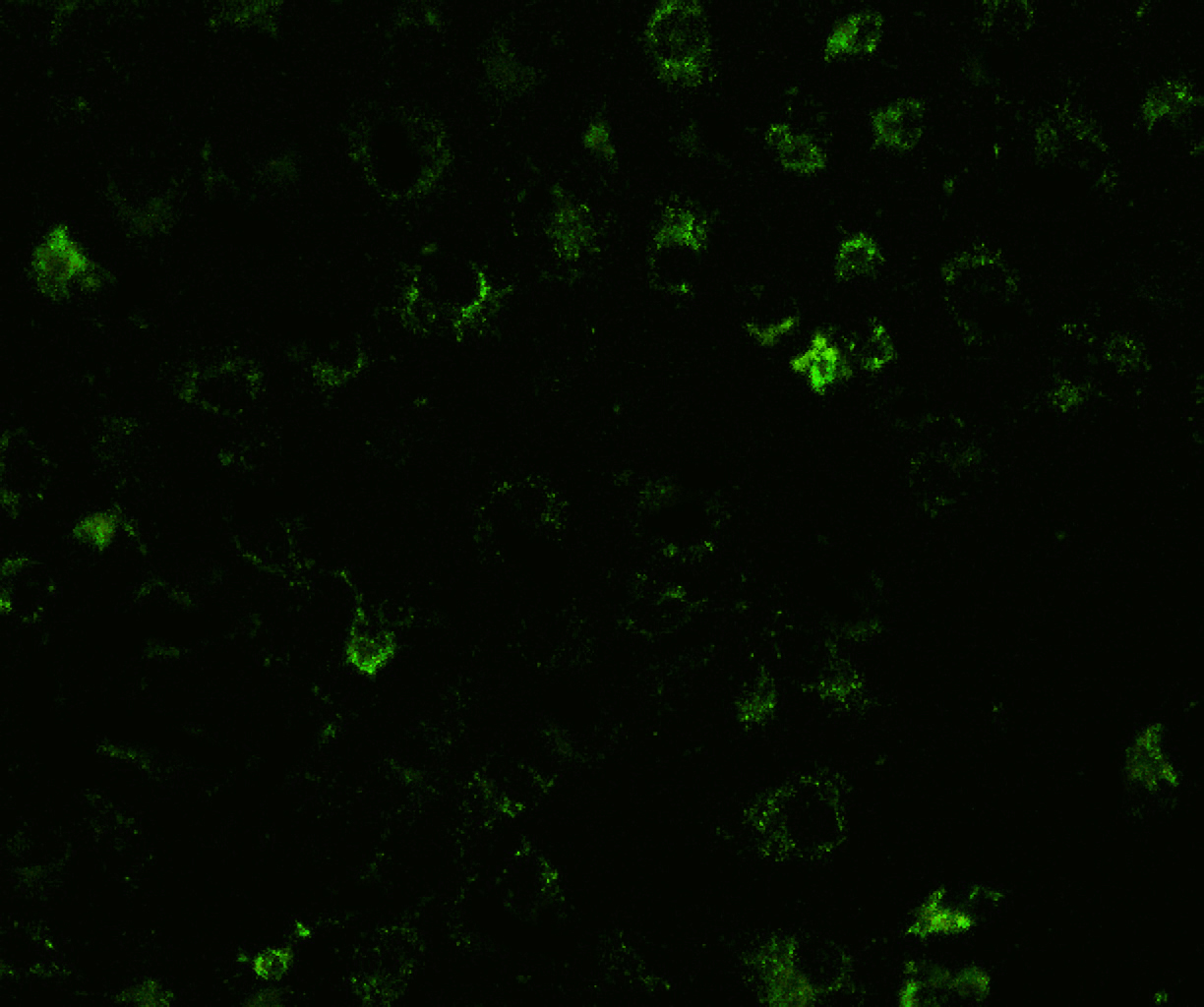

Supplement: Supplementary file 5 [file DataSheet_4.zip › FIG2/IL-1a┬+Exo+Fer-1/1/mono.jpg]

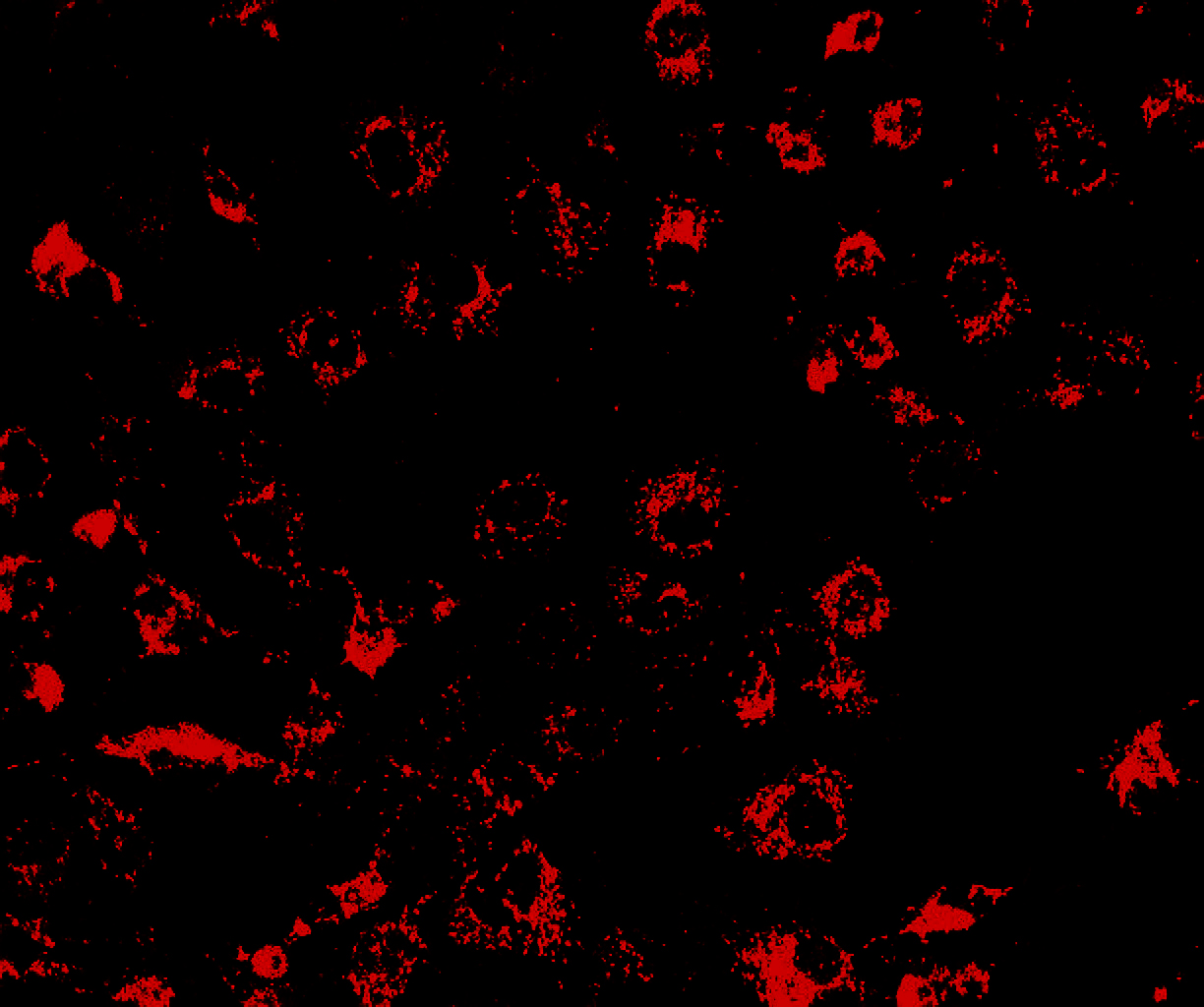

Supplement: Supplementary file 5 [file DataSheet_4.zip › FIG2/IL-1a┬+Exo+Fer-1/1/poly.jpg]

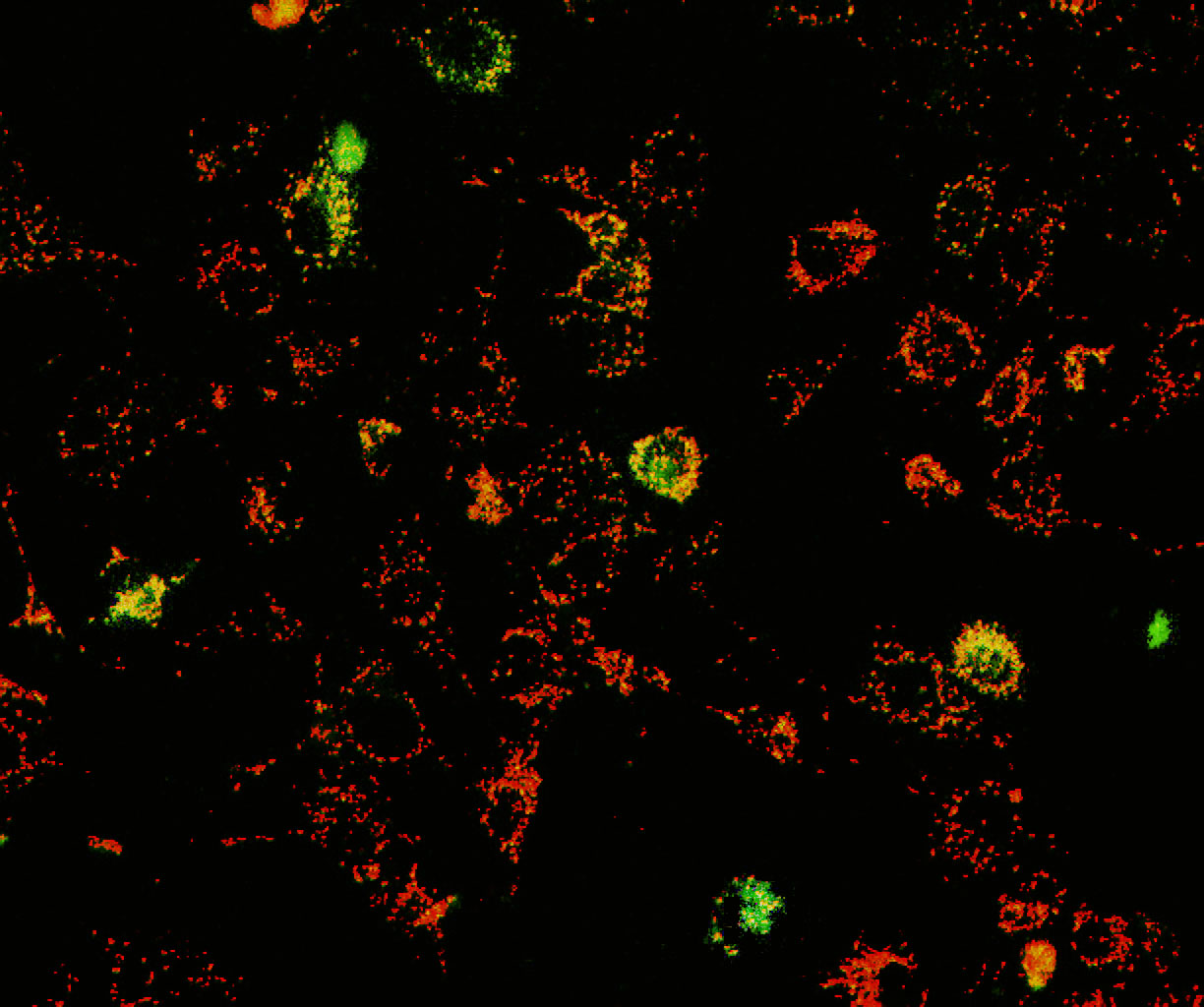

Supplement: Supplementary file 5 [file DataSheet_4.zip › FIG2/IL-1a┬+Exo+Fer-1/2/merge.jpg]

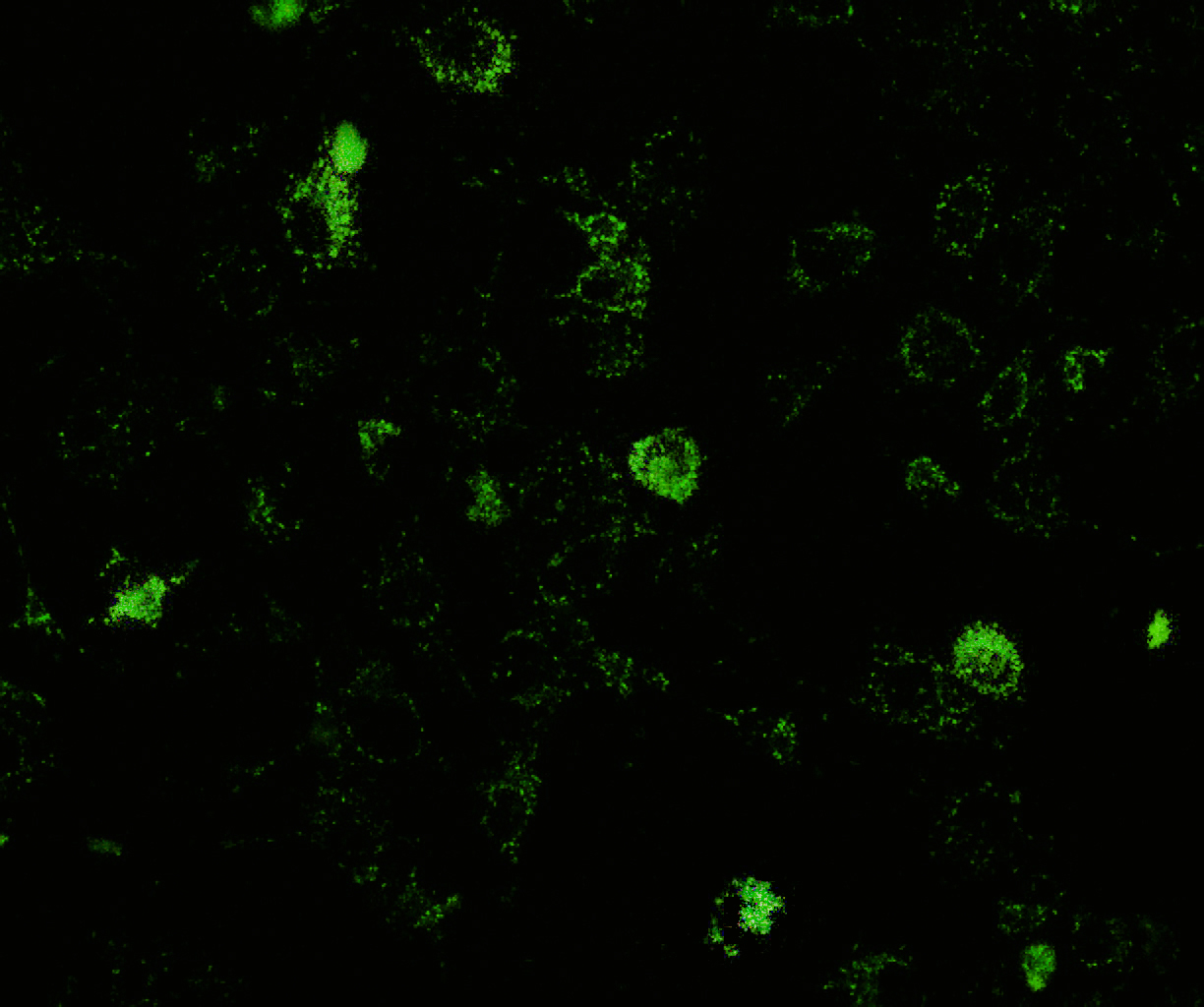

Supplement: Supplementary file 5 [file DataSheet_4.zip › FIG2/IL-1a┬+Exo+Fer-1/2/mono.jpg]

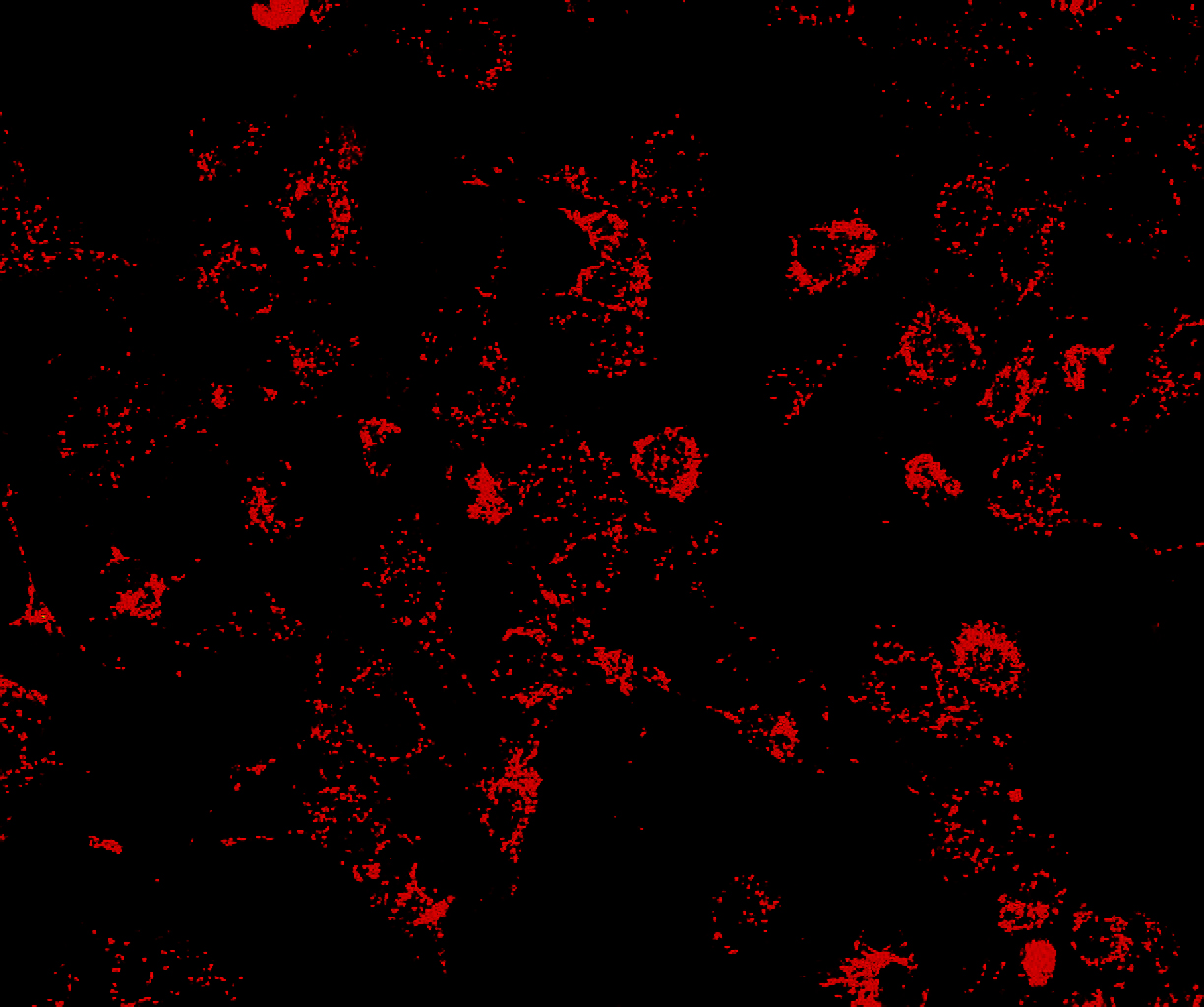

Supplement: Supplementary file 5 [file DataSheet_4.zip › FIG2/IL-1a┬+Exo+Fer-1/2/poly.jpg]

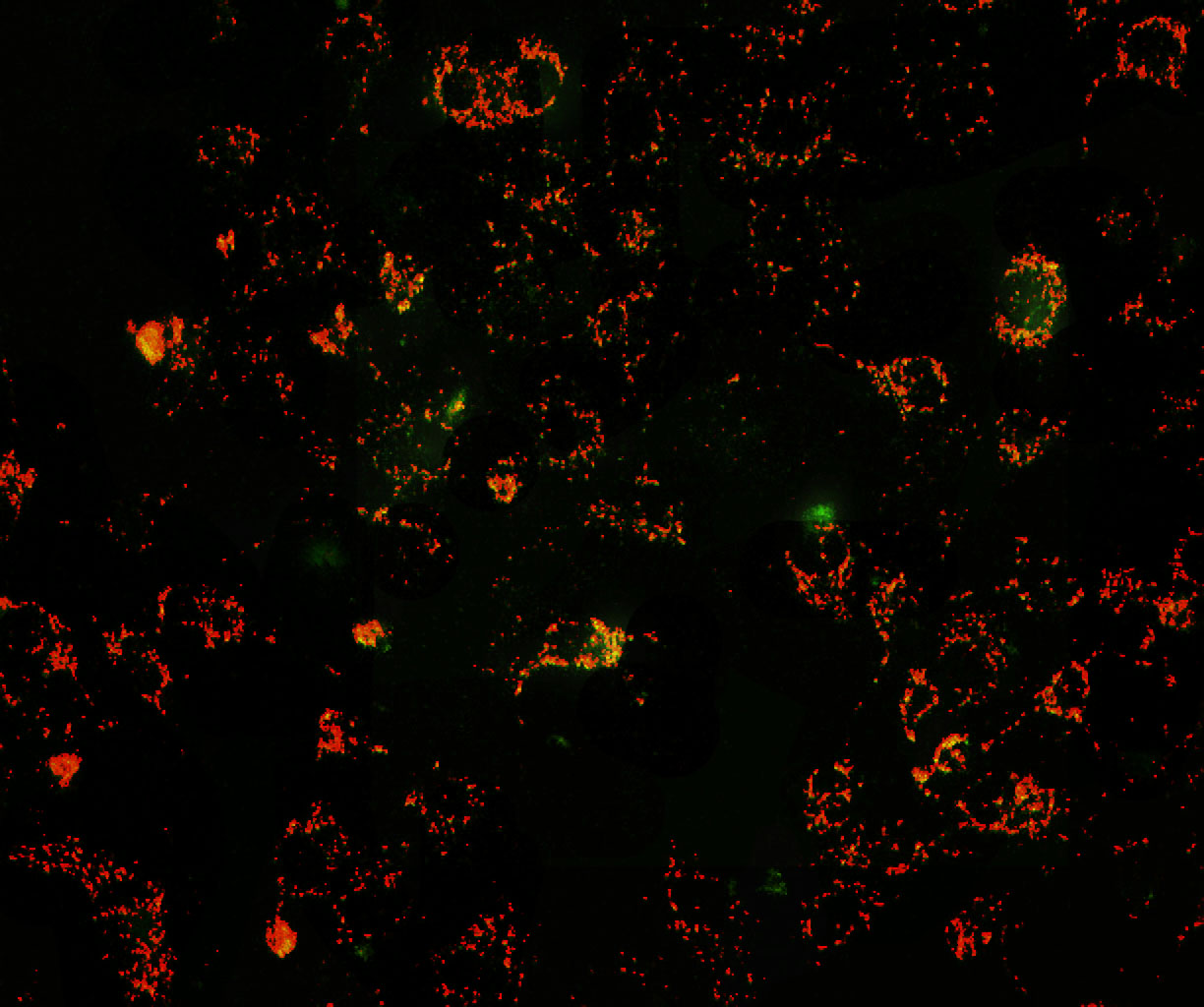

Supplement: Supplementary file 5 [file DataSheet_4.zip › FIG2/IL-1a┬+Exo+Fer-1/3/merge.jpg]

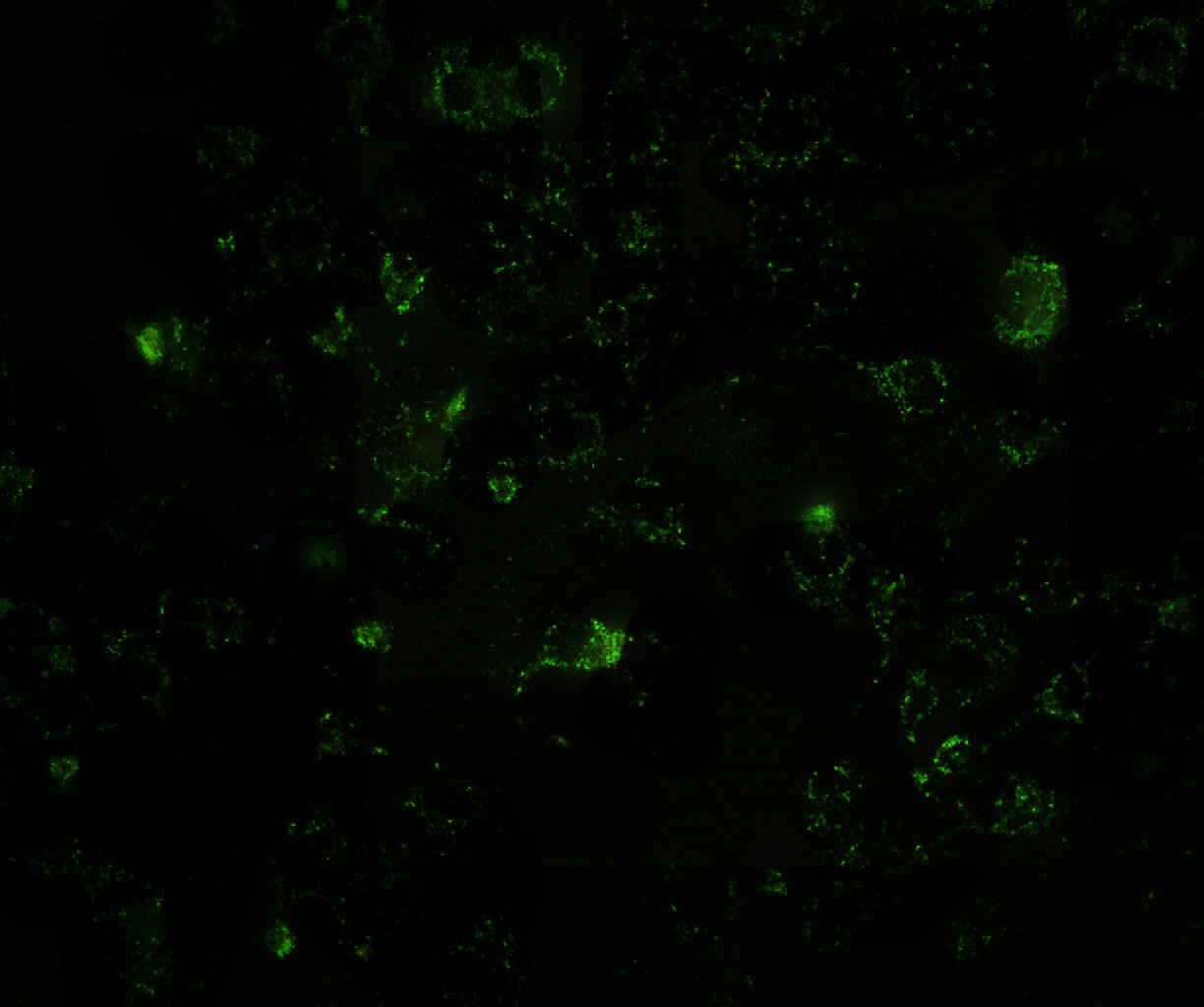

Supplement: Supplementary file 5 [file DataSheet_4.zip › FIG2/IL-1a┬+Exo+Fer-1/3/mono.jpg]

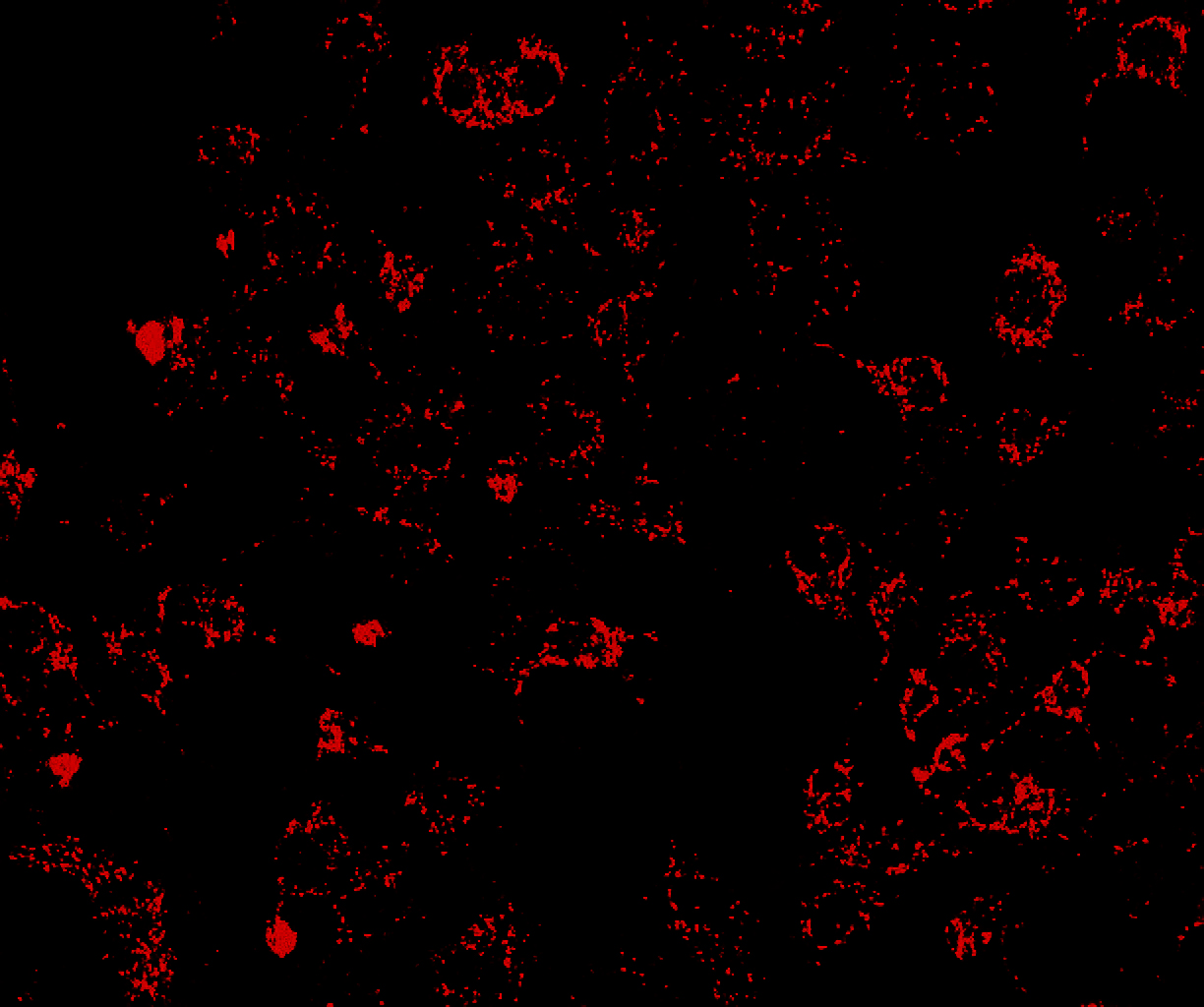

Supplement: Supplementary file 5 [file DataSheet_4.zip › FIG2/IL-1a┬+Exo+Fer-1/3/poly.jpg]

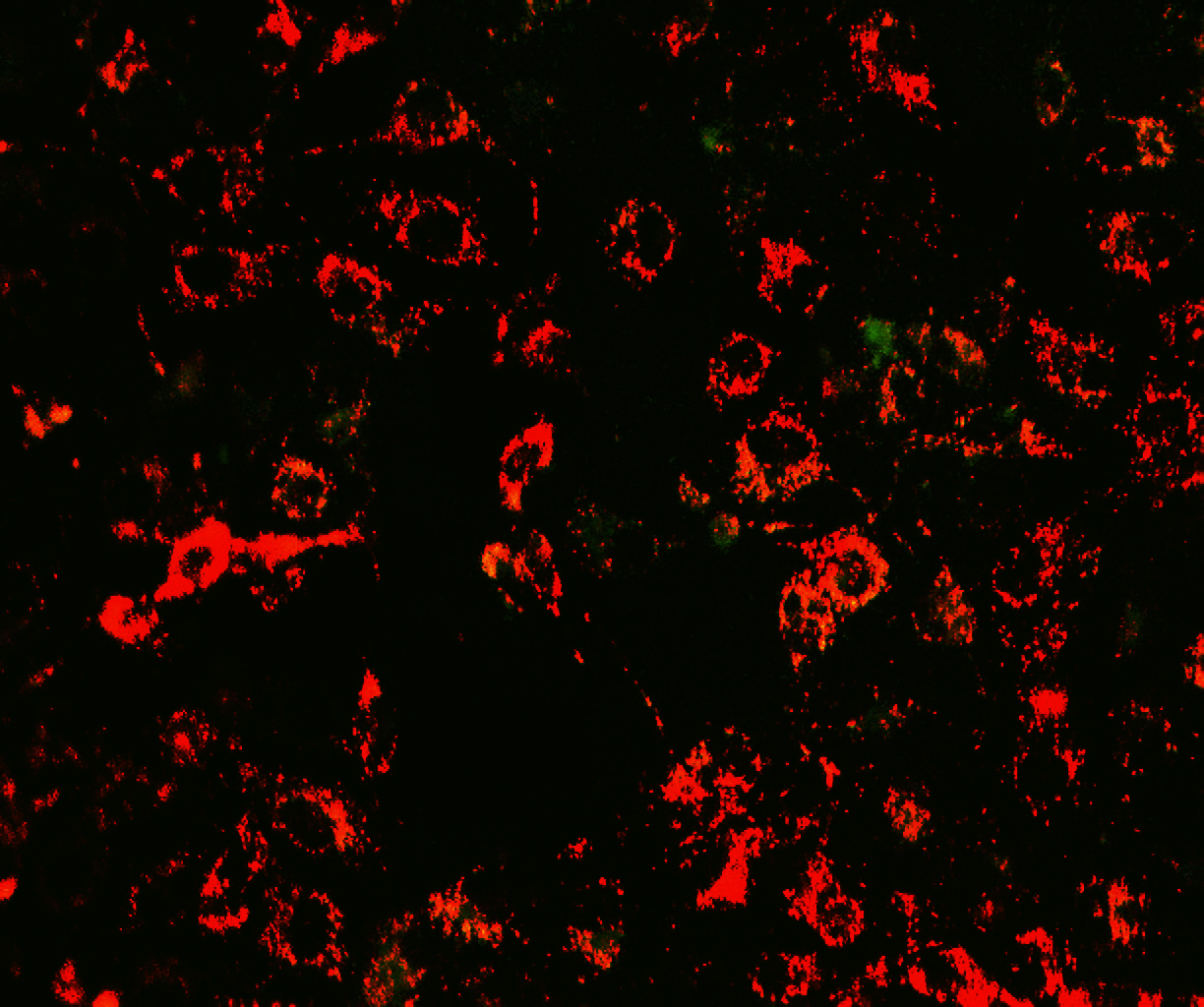

Supplement: Supplementary file 5 [file DataSheet_4.zip › FIG2/Normal/1/merge.jpg]

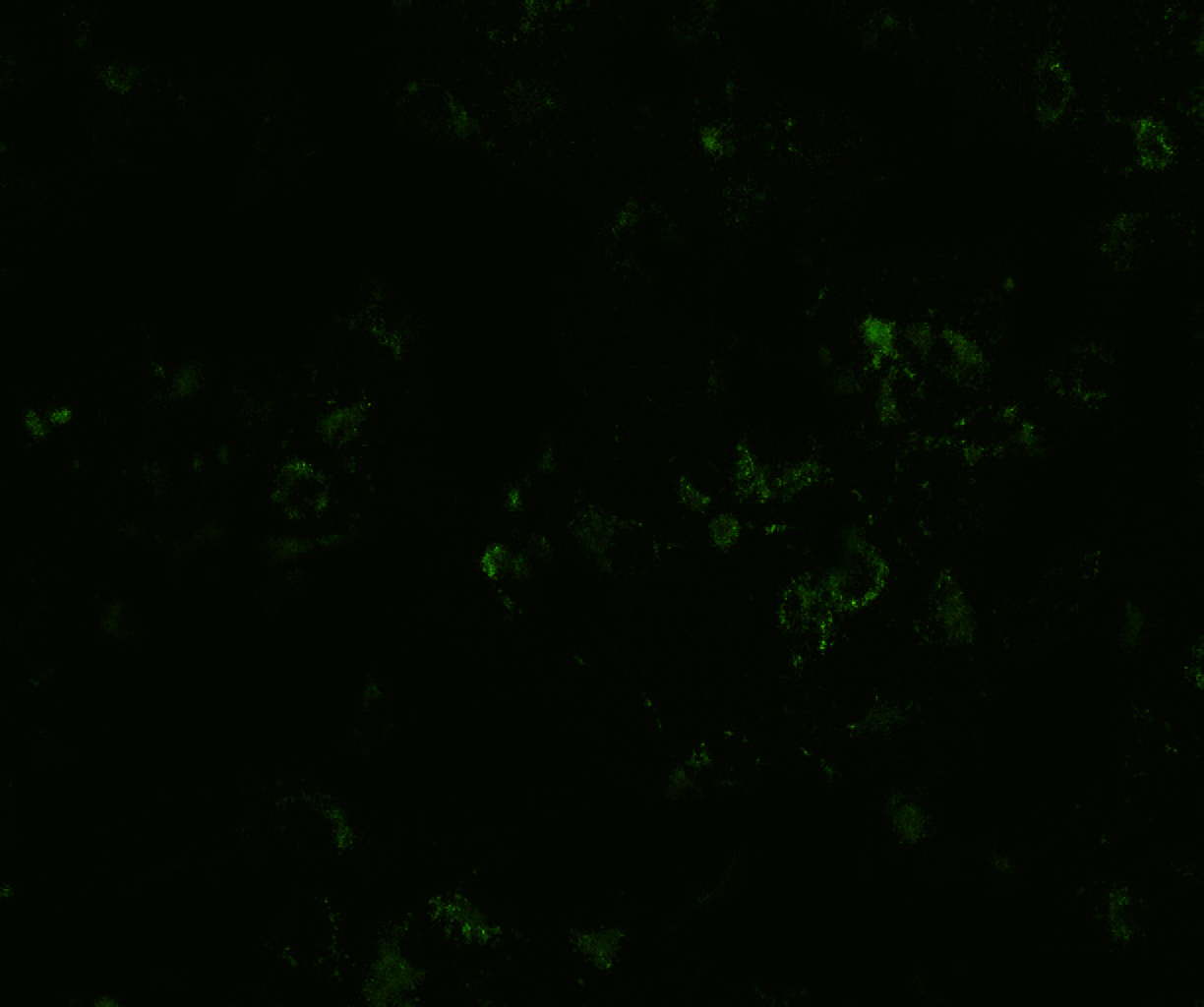

Supplement: Supplementary file 5 [file DataSheet_4.zip › FIG2/Normal/1/mono.jpg]

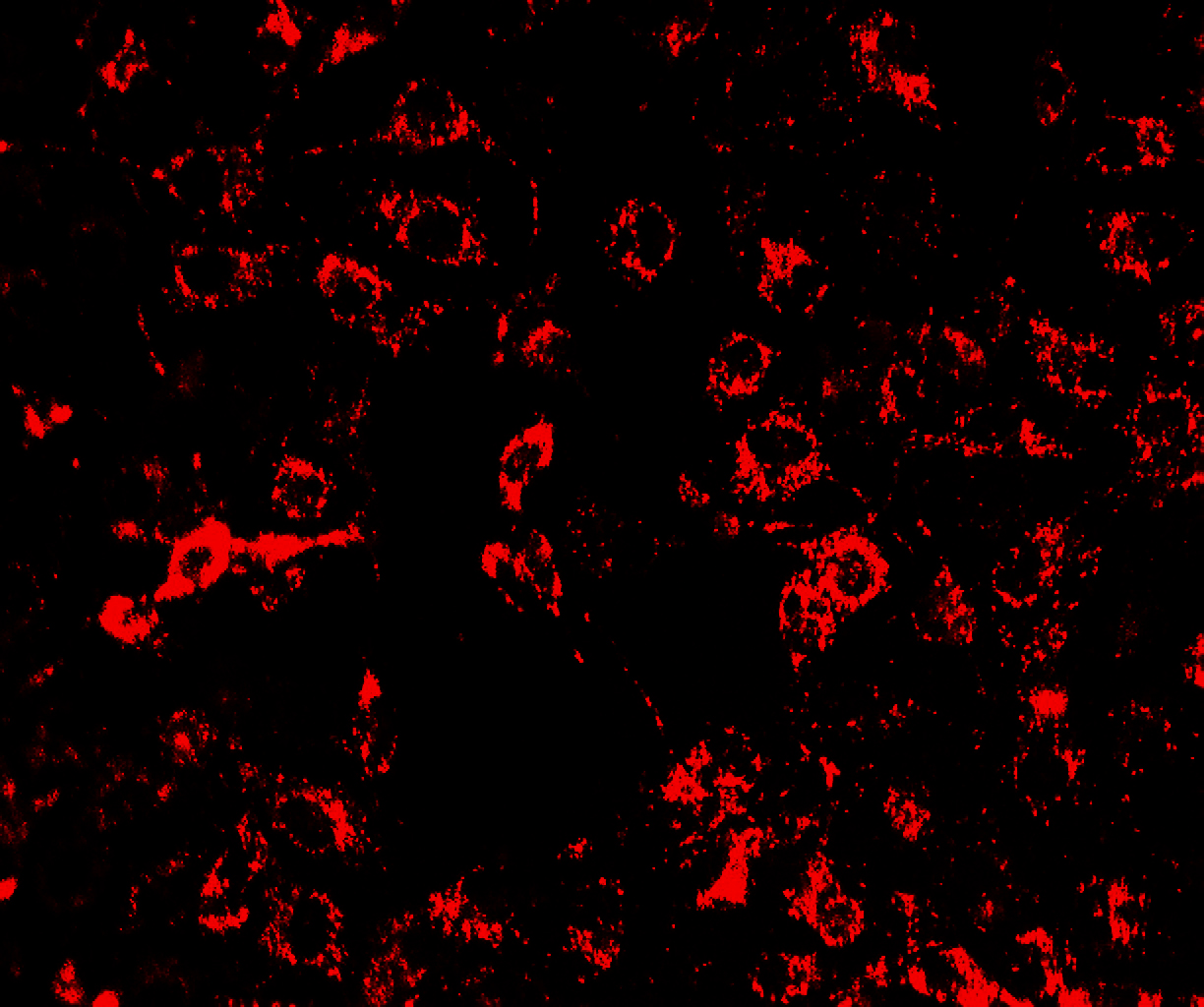

Supplement: Supplementary file 5 [file DataSheet_4.zip › FIG2/Normal/1/poly.jpg]

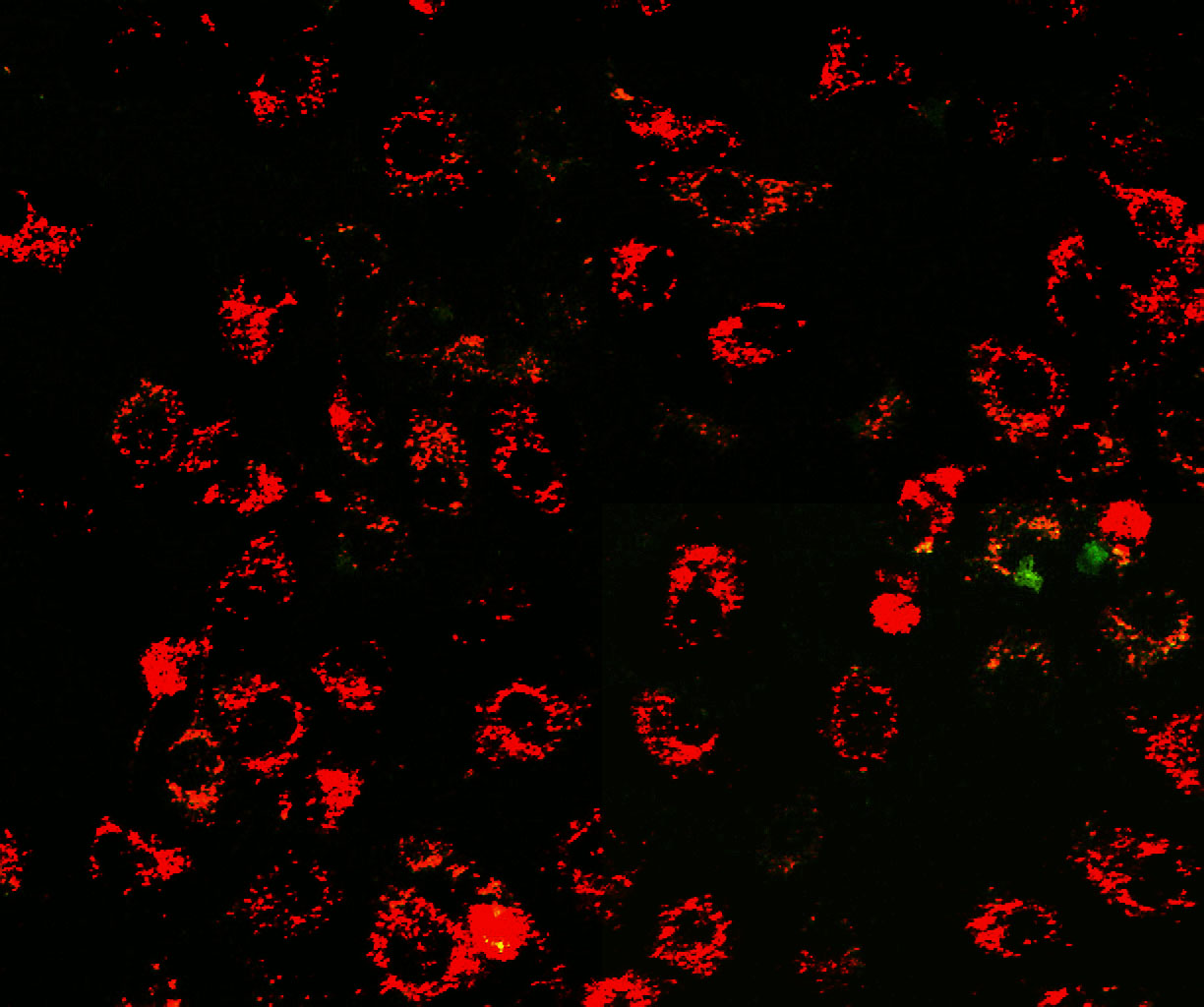

Supplement: Supplementary file 5 [file DataSheet_4.zip › FIG2/Normal/2/merge.jpg]

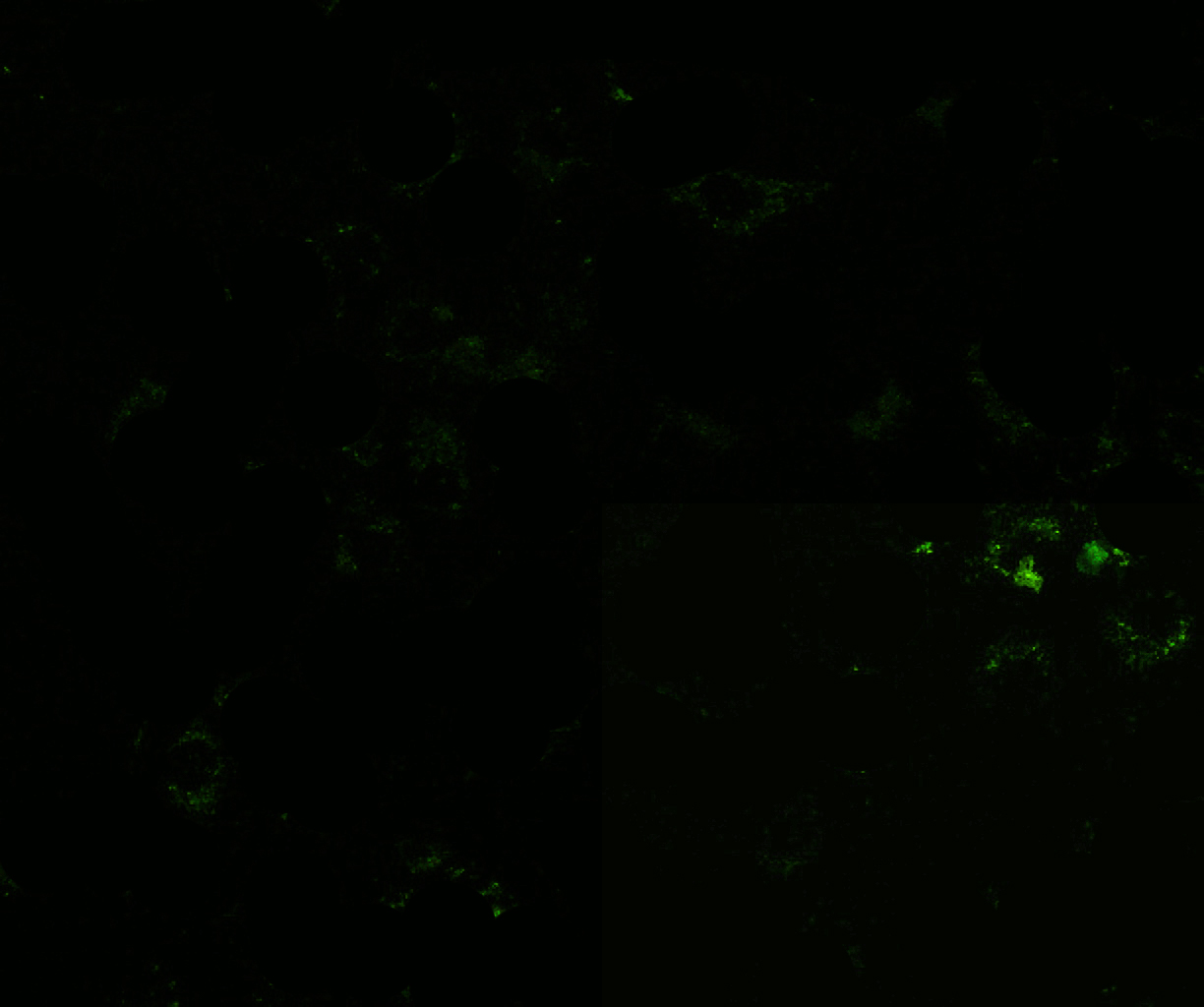

Supplement: Supplementary file 5 [file DataSheet_4.zip › FIG2/Normal/2/mono.jpg]

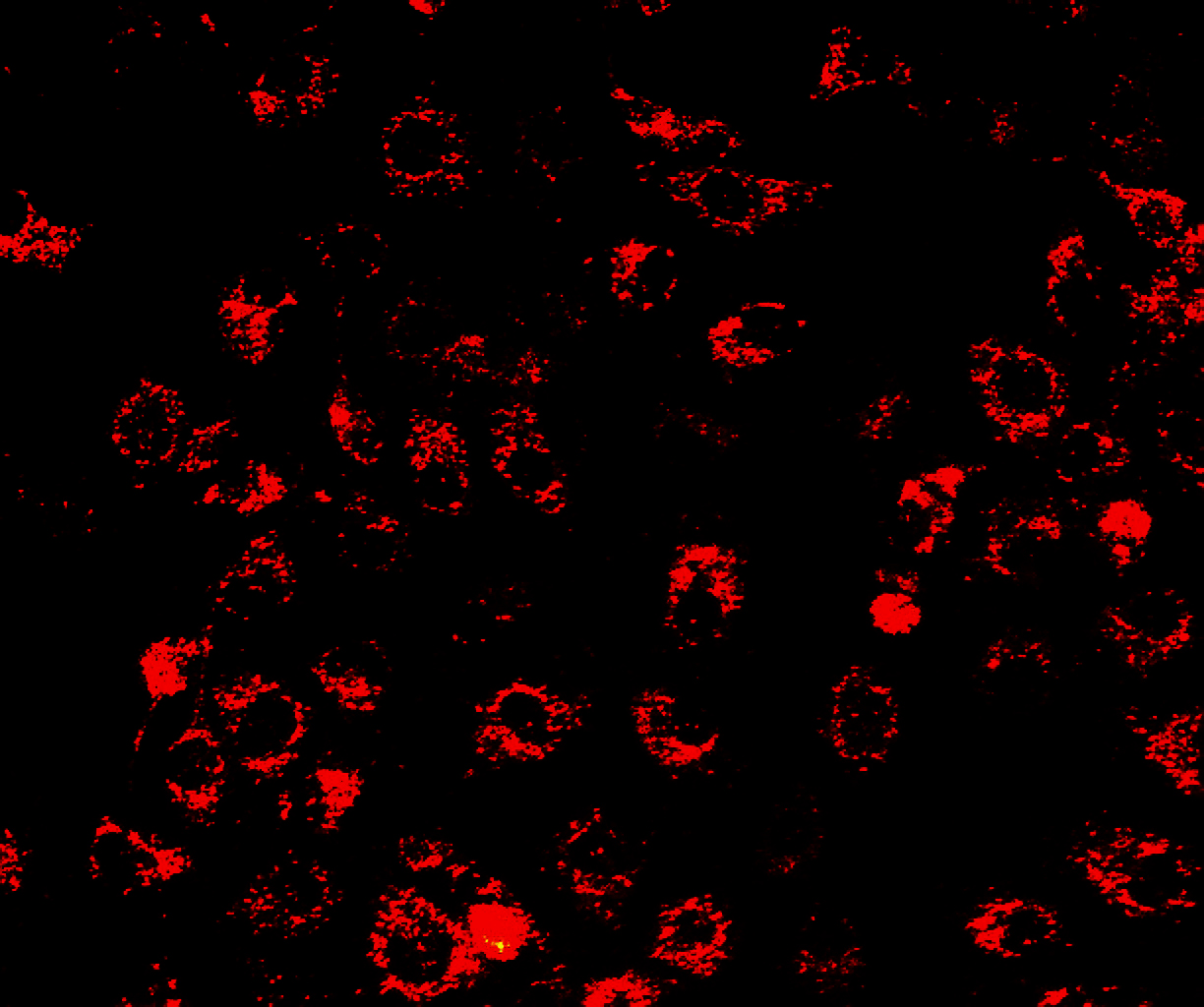

Supplement: Supplementary file 5 [file DataSheet_4.zip › FIG2/Normal/2/poly.jpg]

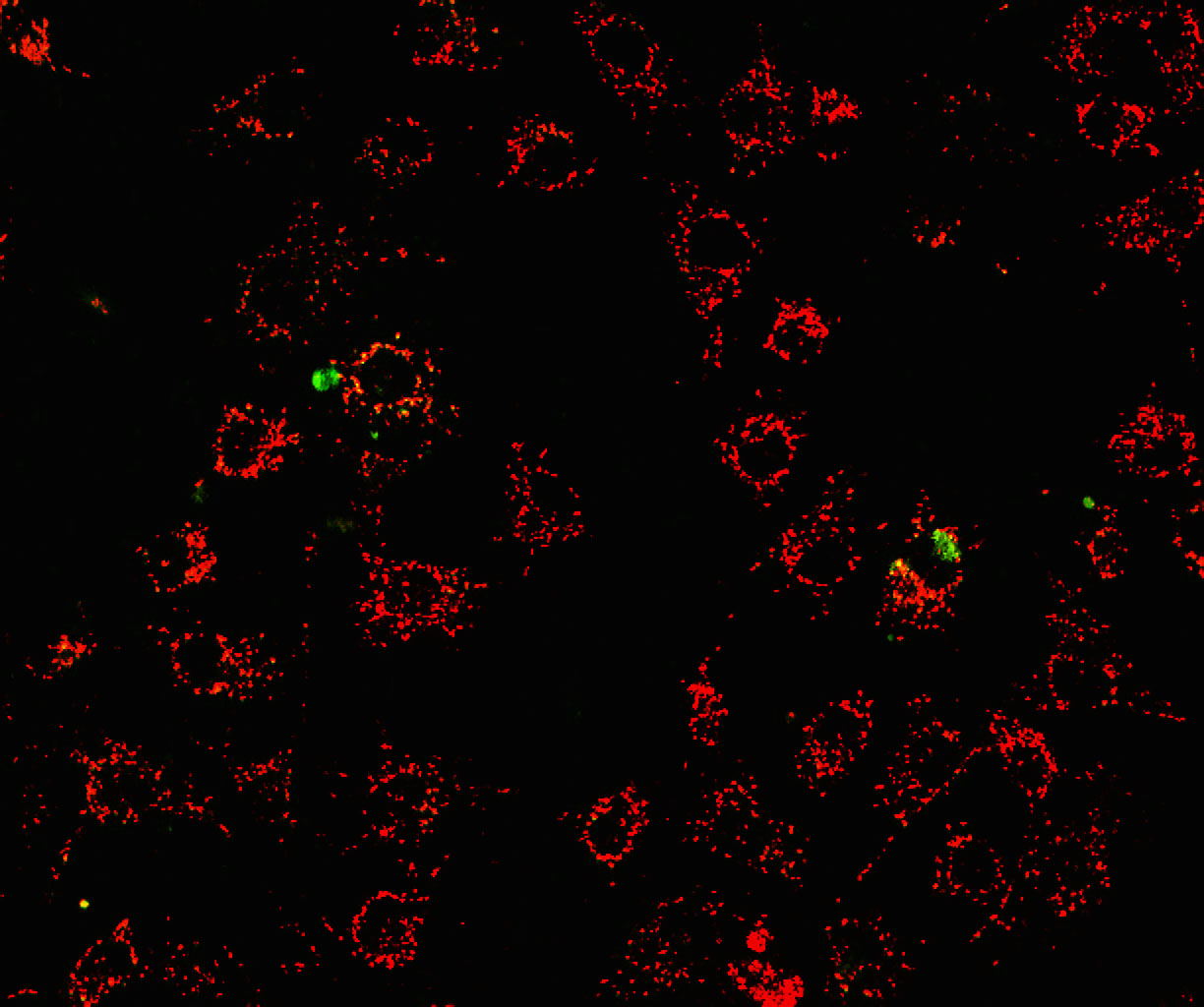

Supplement: Supplementary file 5 [file DataSheet_4.zip › FIG2/Normal/3/merge.jpg]

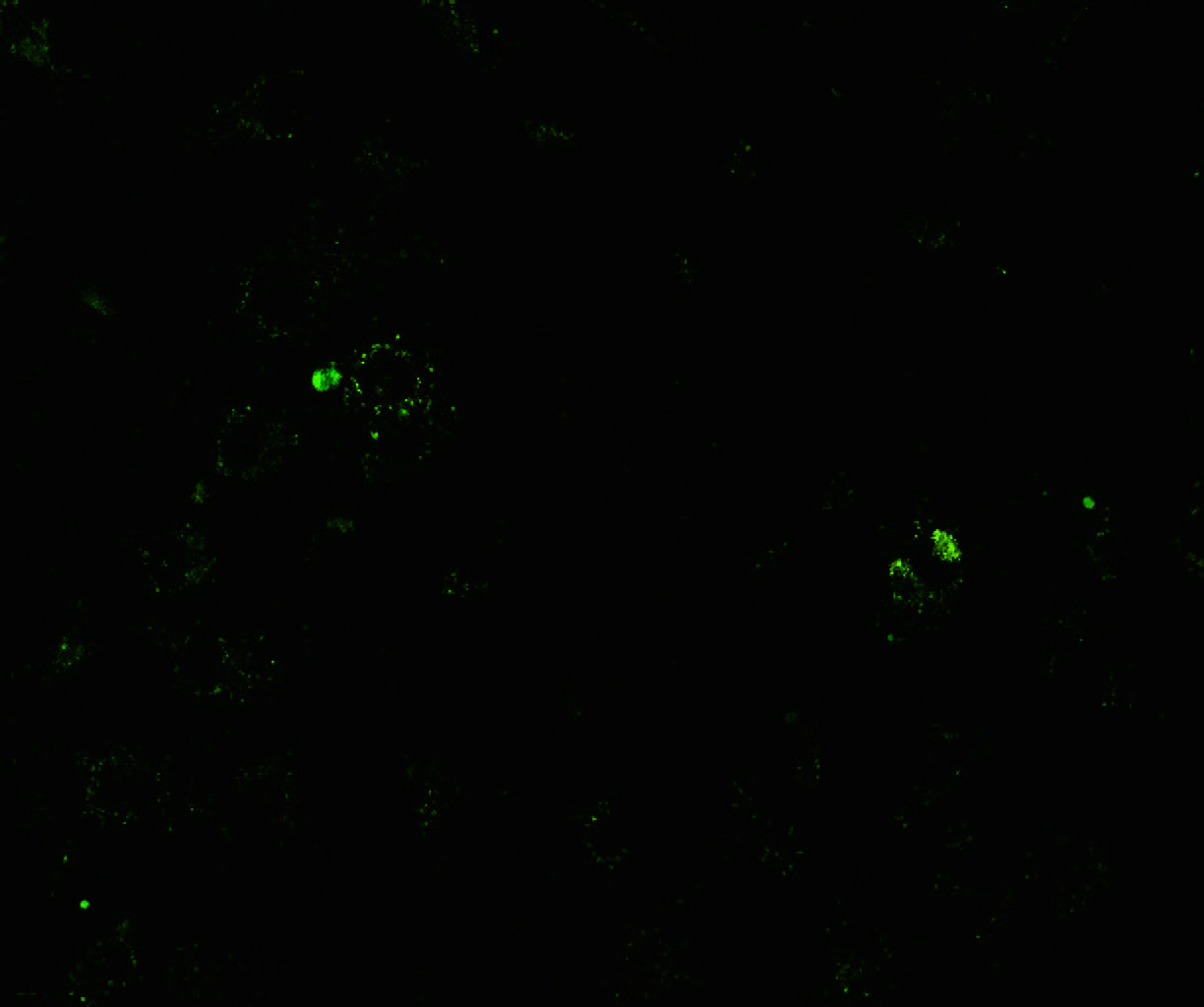

Supplement: Supplementary file 5 [file DataSheet_4.zip › FIG2/Normal/3/mono.jpg]

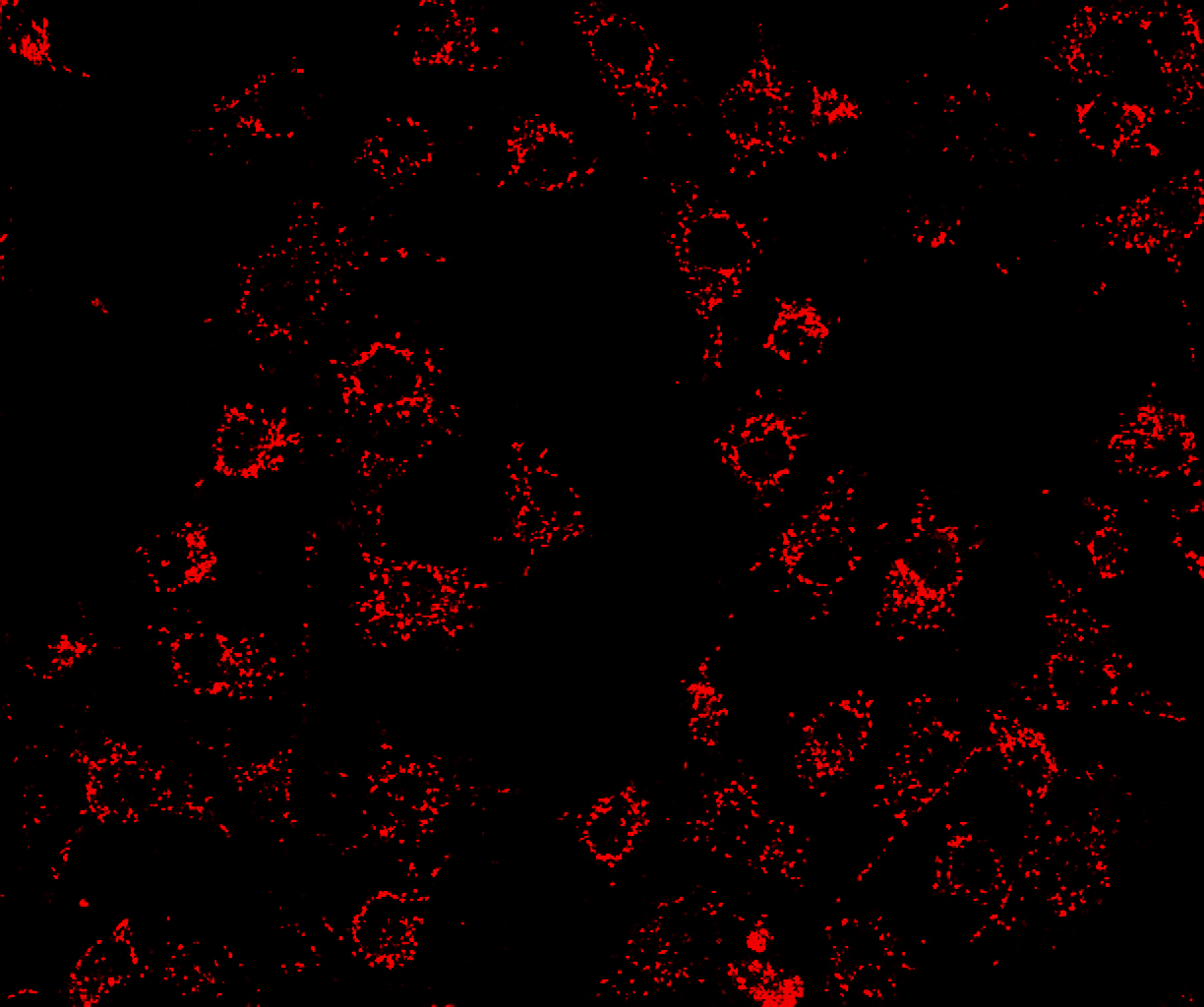

Supplement: Supplementary file 5 [file DataSheet_4.zip › FIG2/Normal/3/poly.jpg]

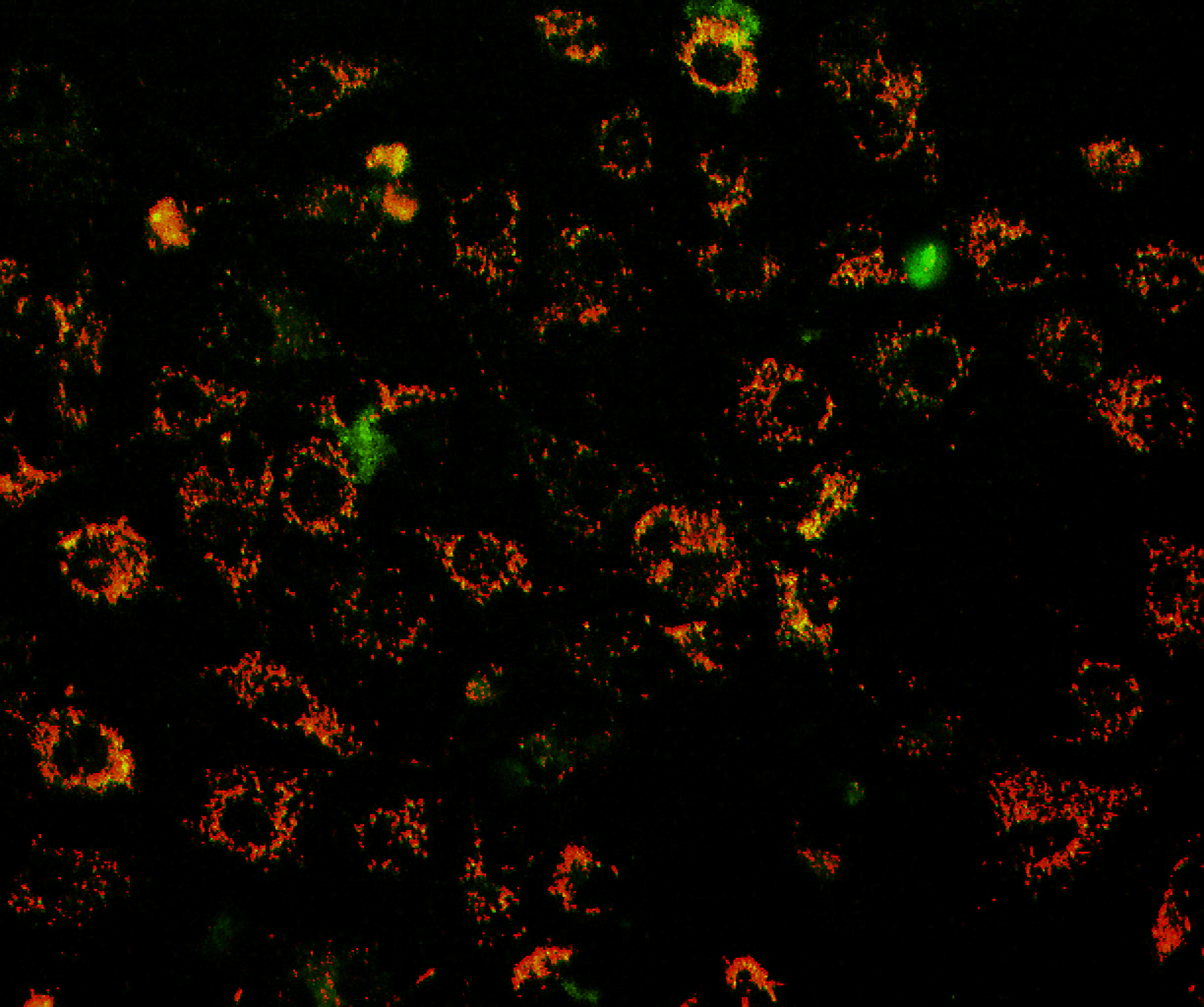

Supplement: Supplementary file 6 [file DataSheet_5.zip › FIG4/Control/1/merge.jpg]

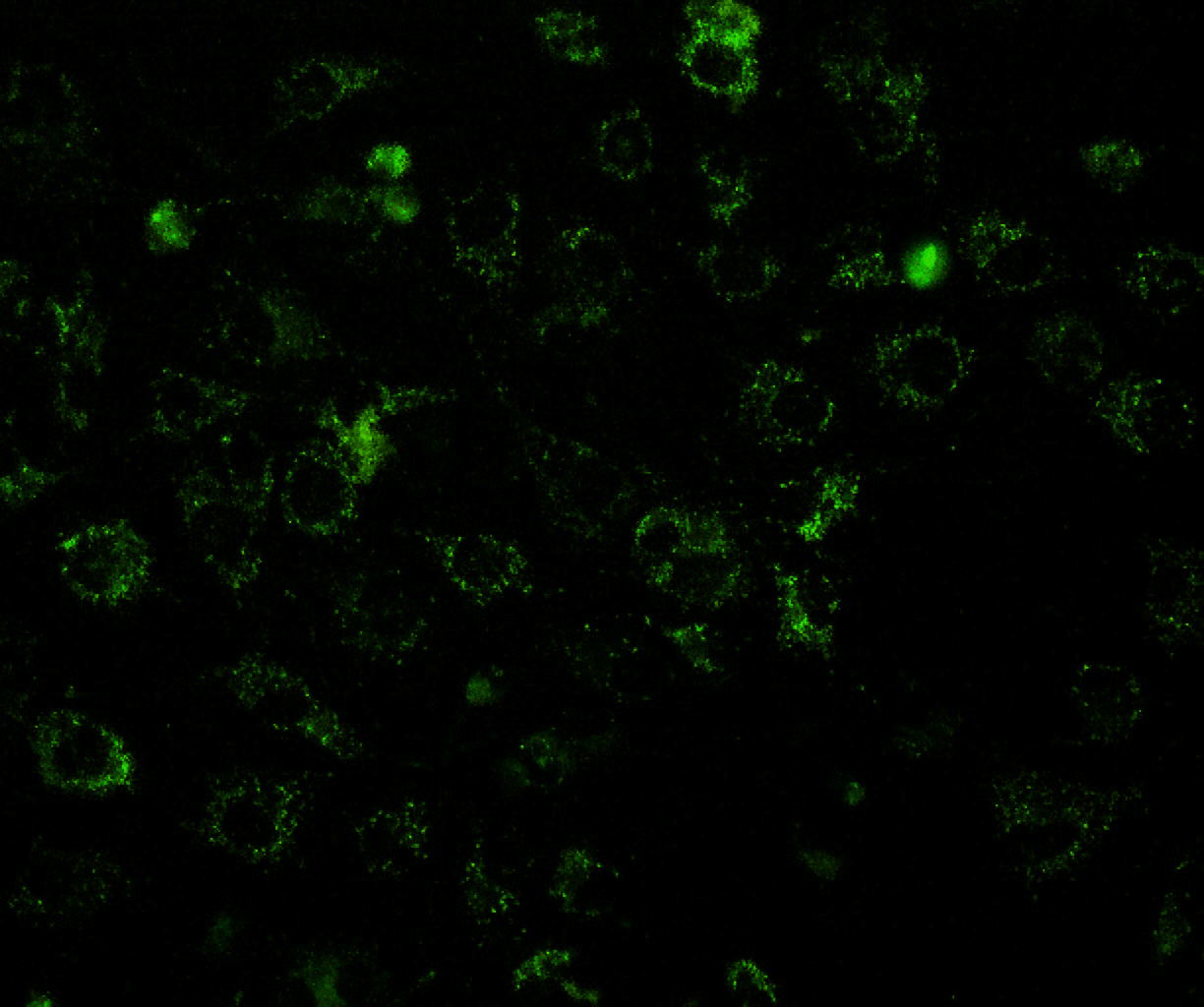

Supplement: Supplementary file 6 [file DataSheet_5.zip › FIG4/Control/1/mono.jpg]

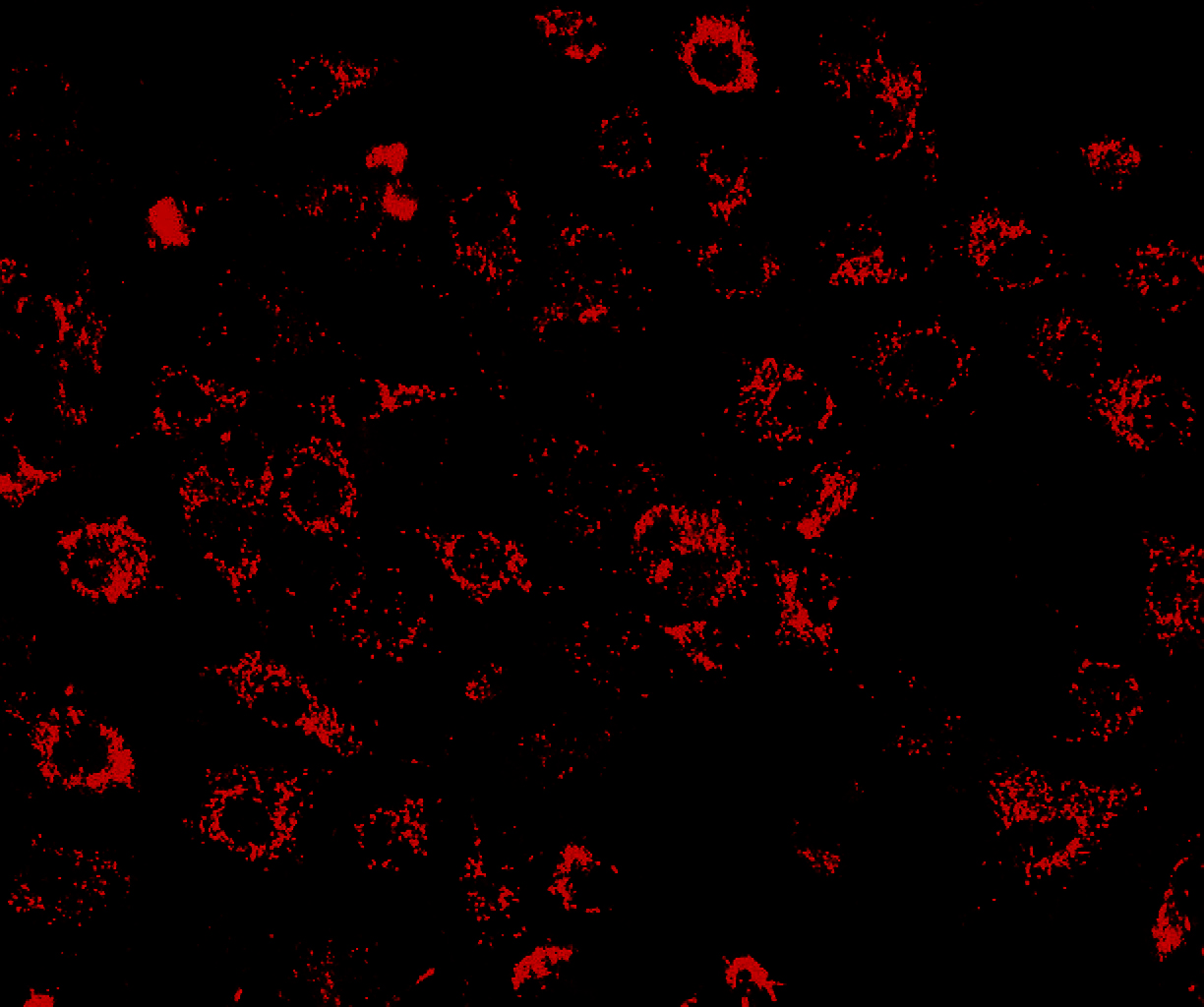

Supplement: Supplementary file 6 [file DataSheet_5.zip › FIG4/Control/1/poly.jpg]

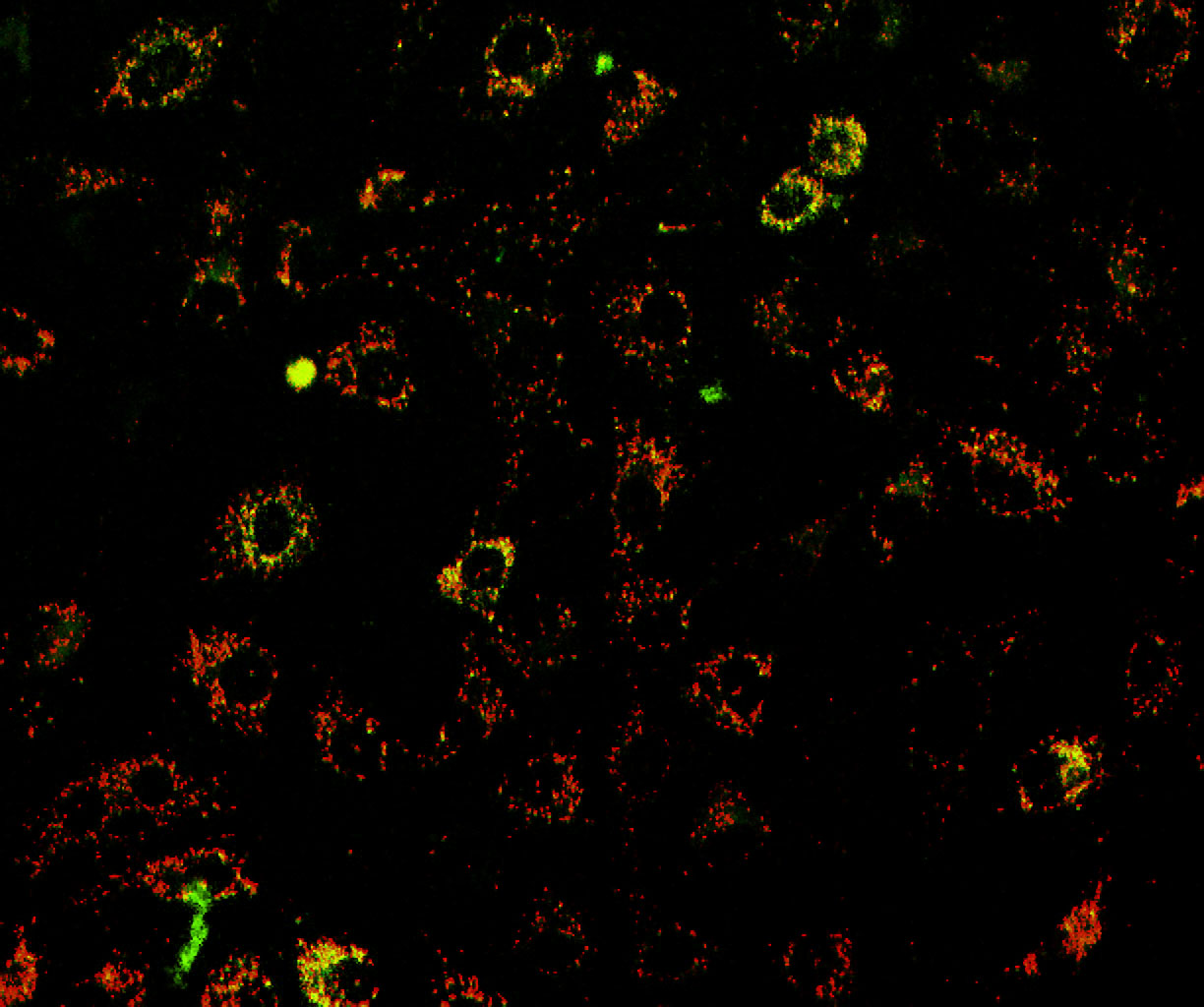

Supplement: Supplementary file 6 [file DataSheet_5.zip › FIG4/Control/2/merge.jpg]

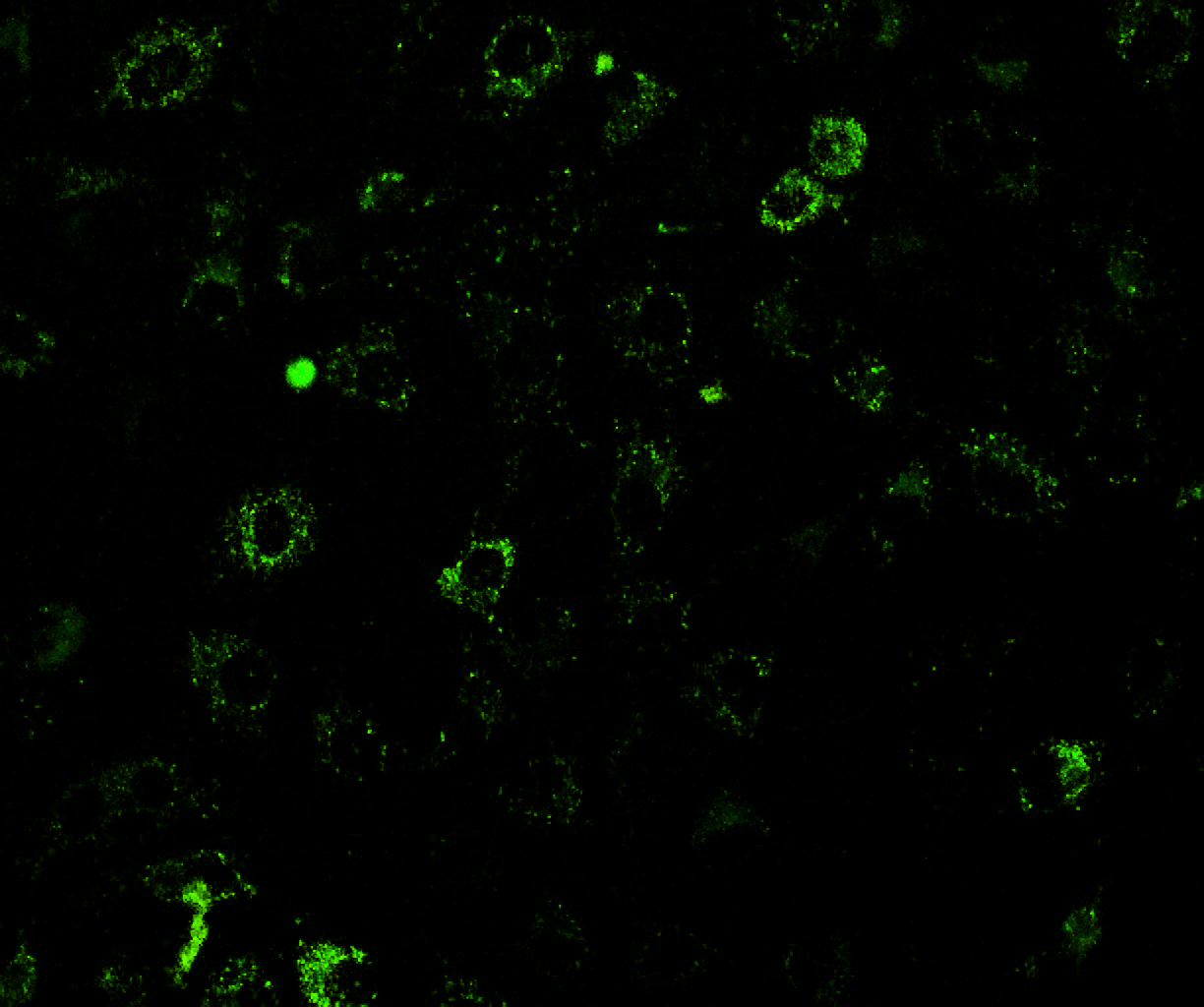

Supplement: Supplementary file 6 [file DataSheet_5.zip › FIG4/Control/2/mono.jpg]

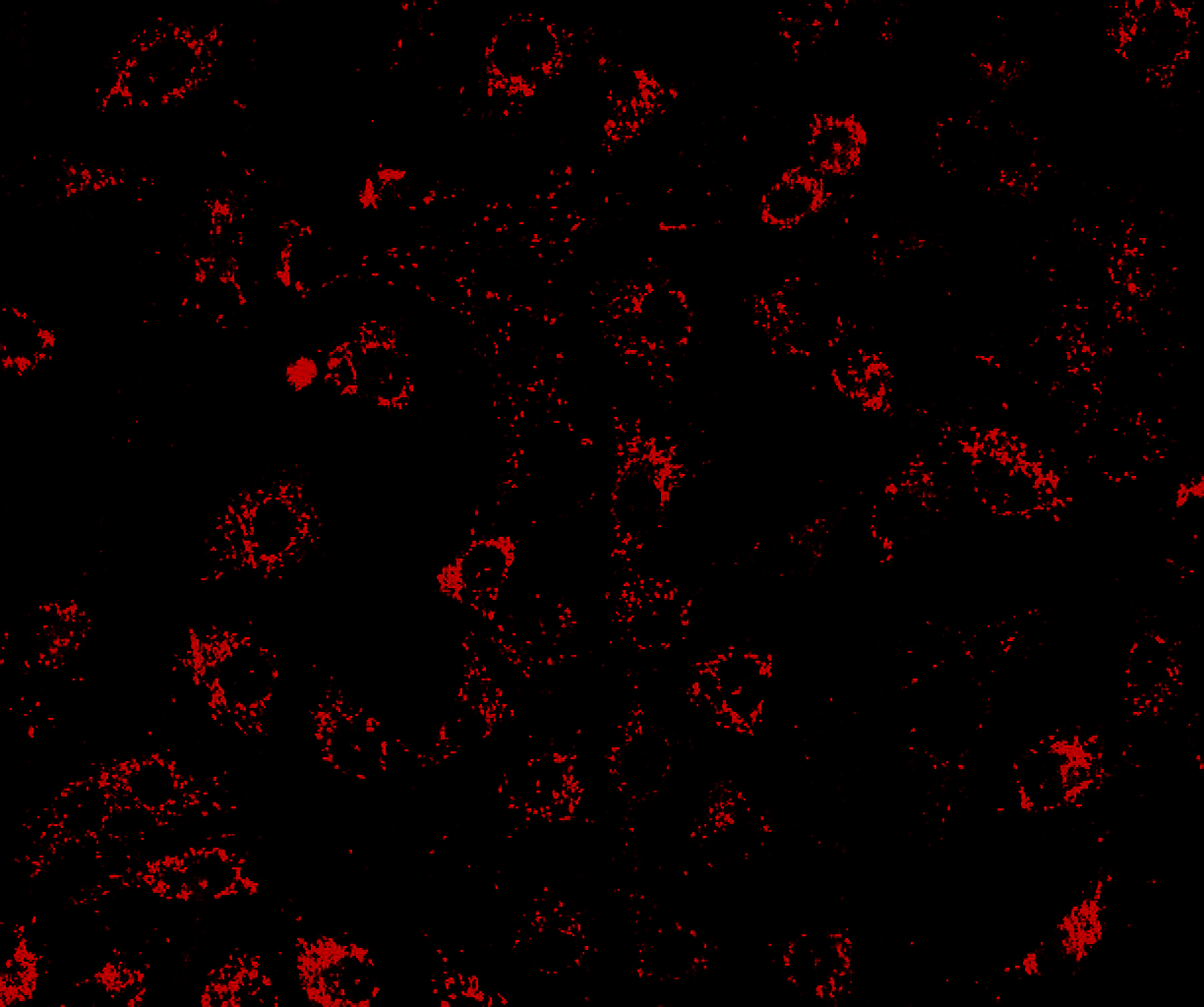

Supplement: Supplementary file 6 [file DataSheet_5.zip › FIG4/Control/2/poly.jpg]

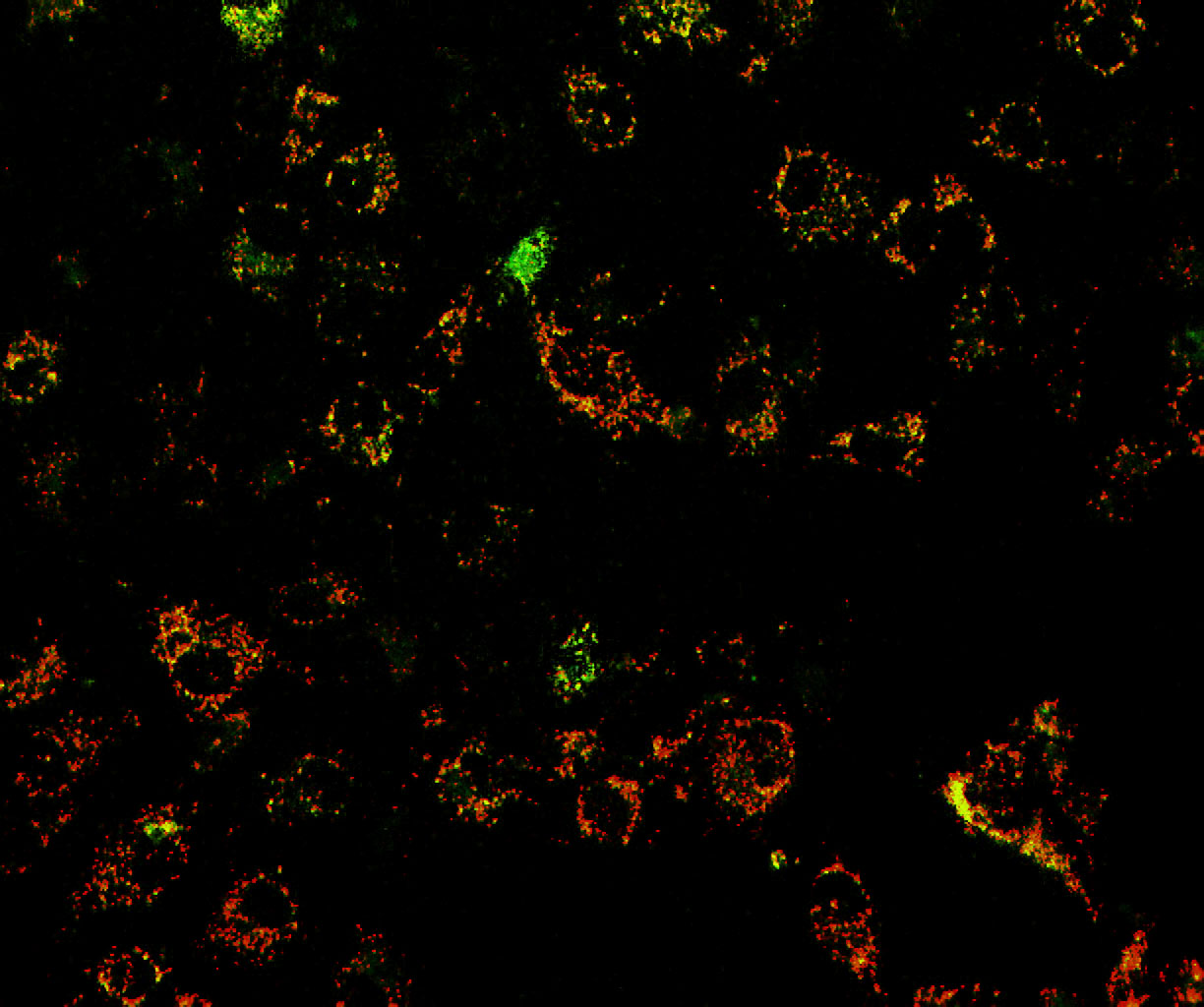

Supplement: Supplementary file 6 [file DataSheet_5.zip › FIG4/Control/3/merge.jpg]

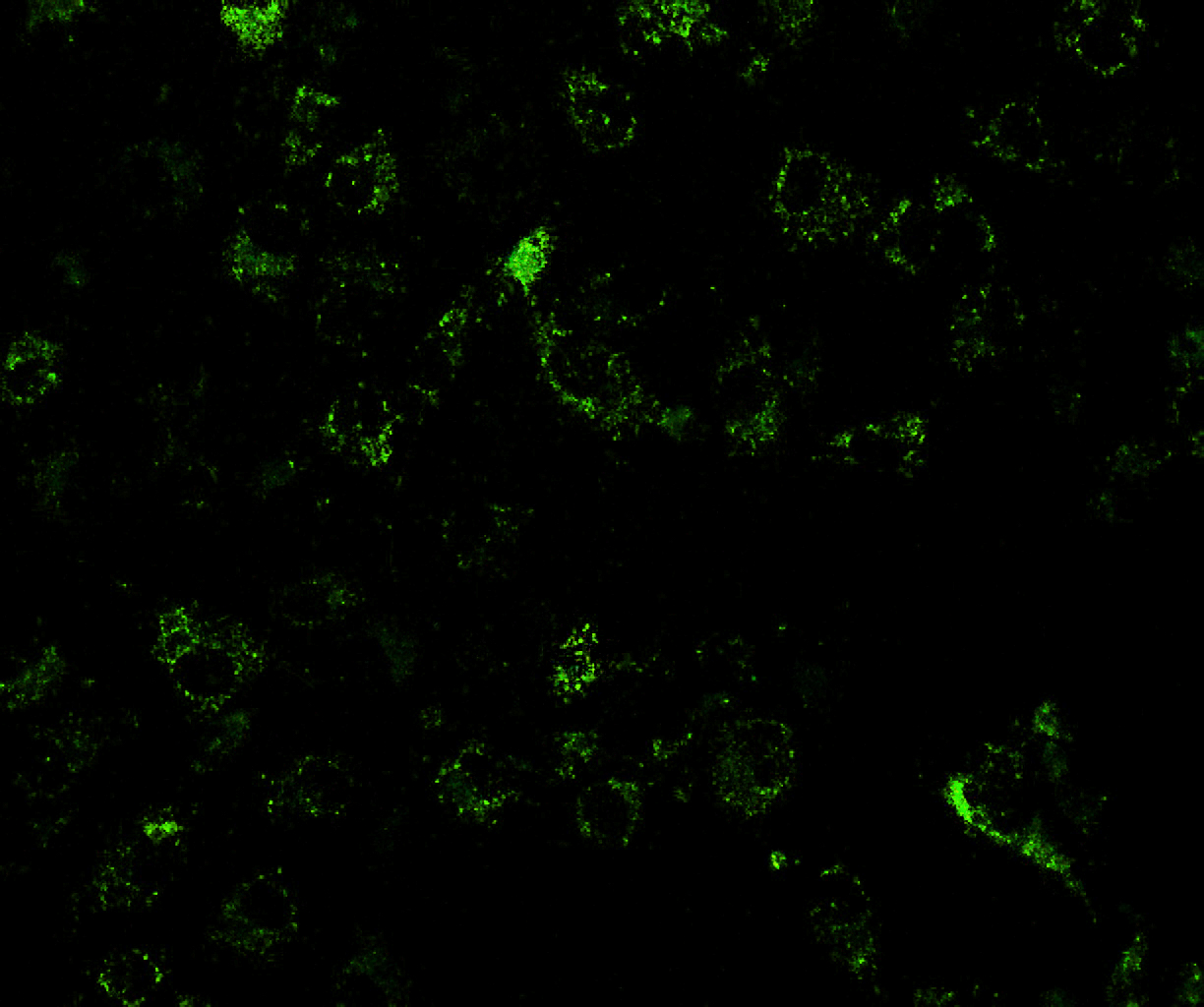

Supplement: Supplementary file 6 [file DataSheet_5.zip › FIG4/Control/3/mono.jpg]
