# Supplementary figures and images for: Exosomes from osteoarthritic fibroblast-like synoviocytes promote cartilage ferroptosis and damage via delivering microRNA-19b-3p to target SLC7A11 in osteoarthritis (part 4 of 6)
Source: Front Immunol. 2023 Aug 24;14:1181156. doi: 10.3389/fimmu.2023.1181156 (PMC10484587; doi:10.3389/fimmu.2023.1181156)

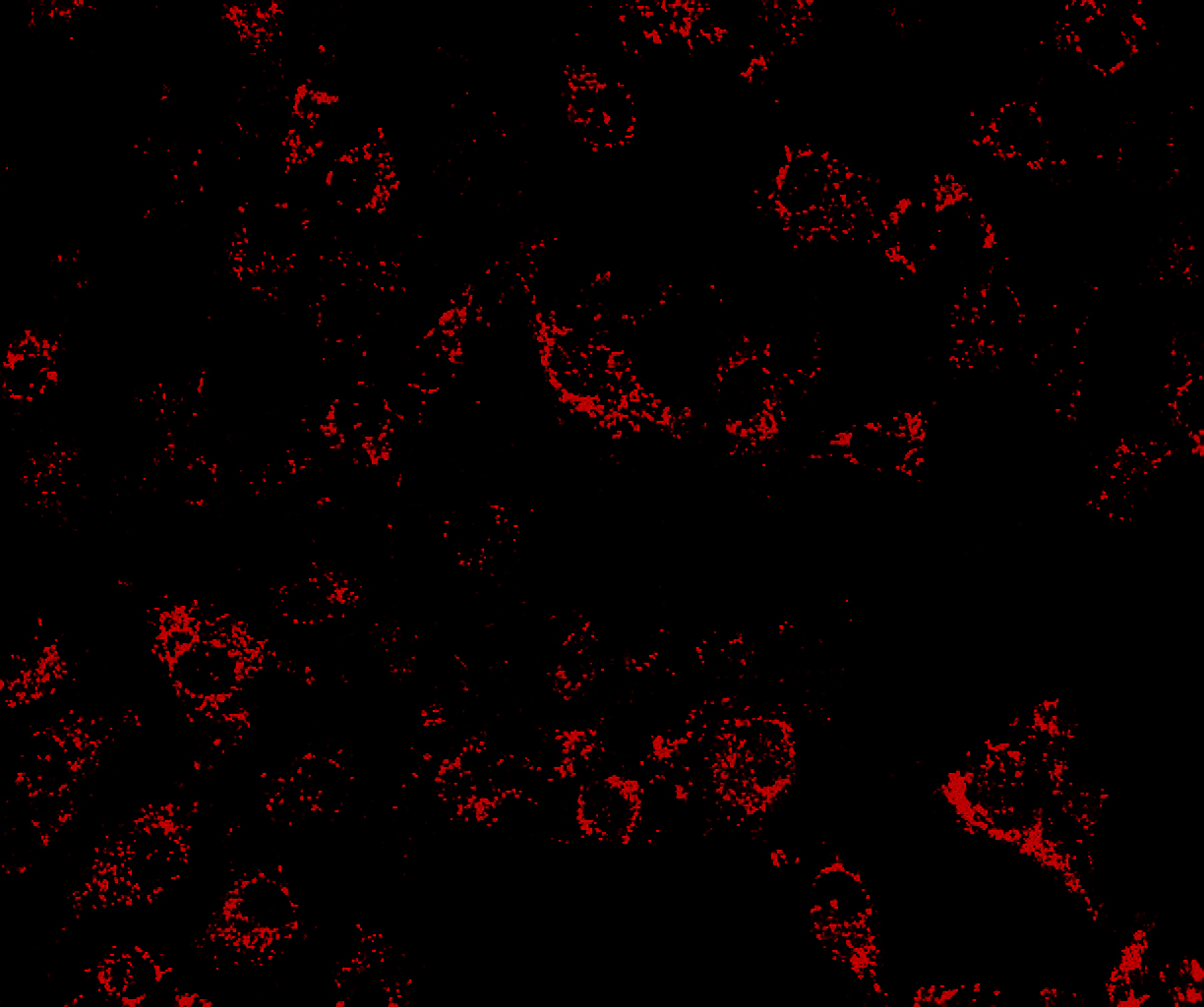

Supplement: Supplementary file 6 [file DataSheet_5.zip › FIG4/Control/3/poly.jpg]

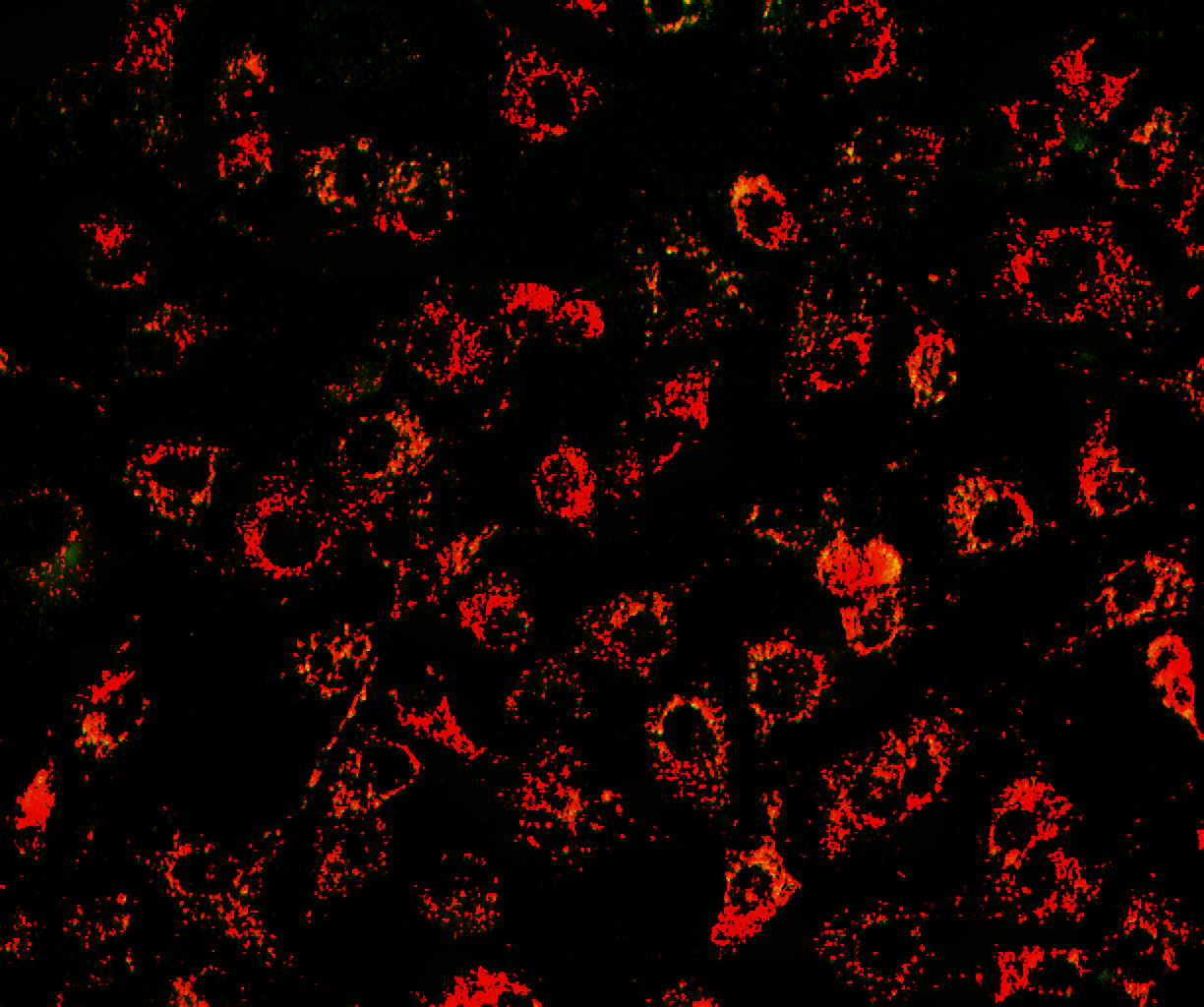

Supplement: Supplementary file 6 [file DataSheet_5.zip › FIG4/miR(-)/1/merge.jpg]

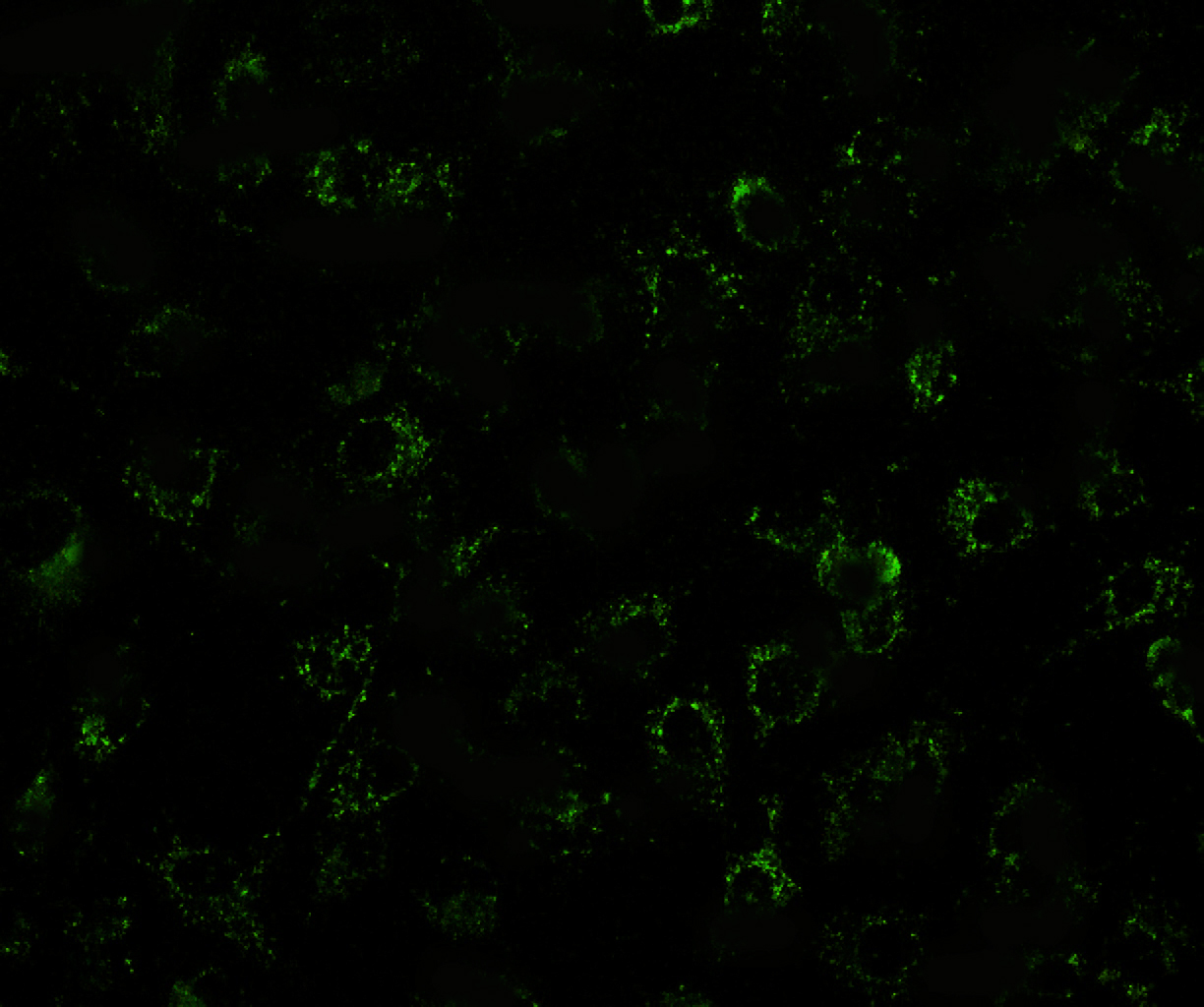

Supplement: Supplementary file 6 [file DataSheet_5.zip › FIG4/miR(-)/1/mono.jpg]

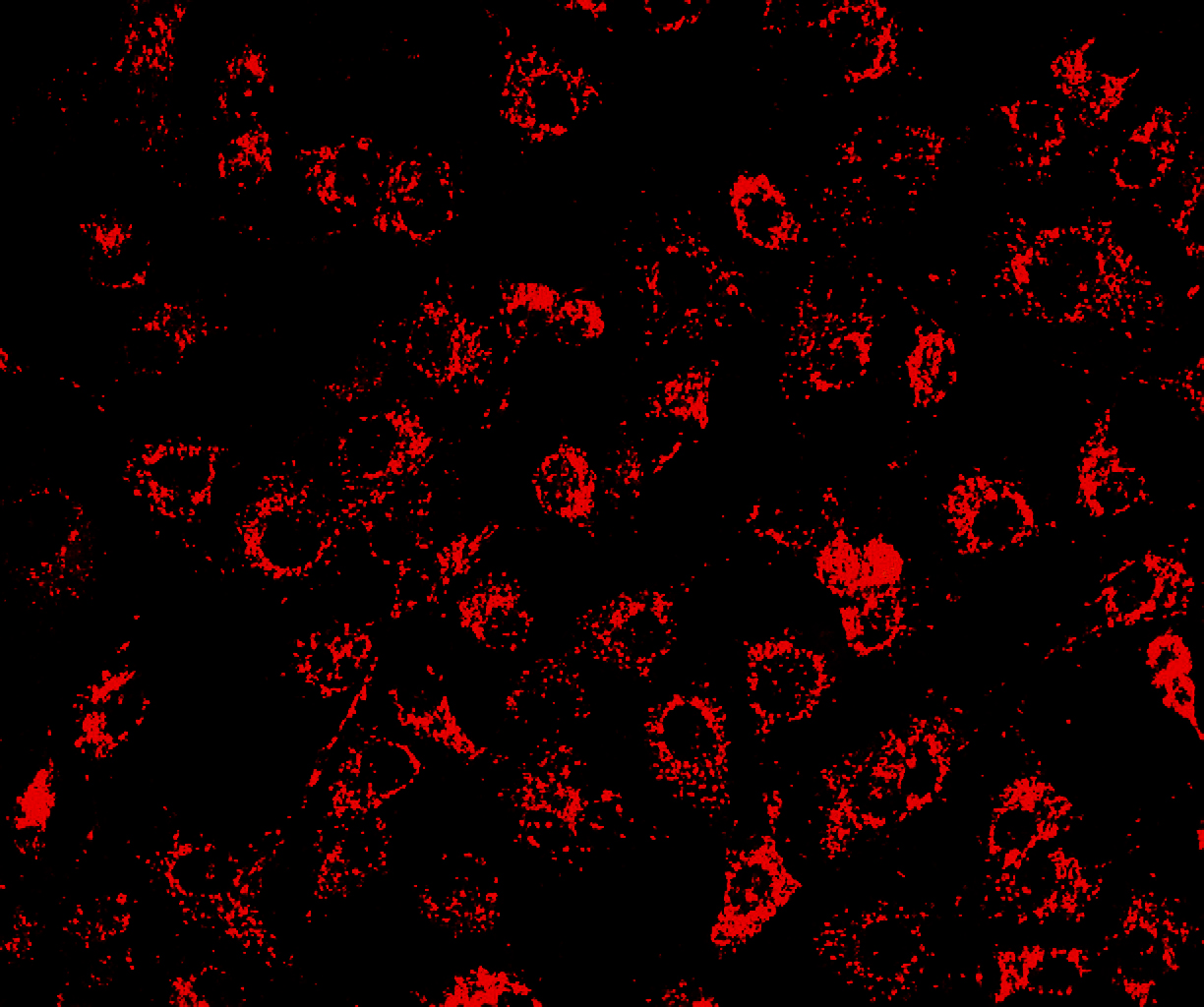

Supplement: Supplementary file 6 [file DataSheet_5.zip › FIG4/miR(-)/1/poly.jpg]

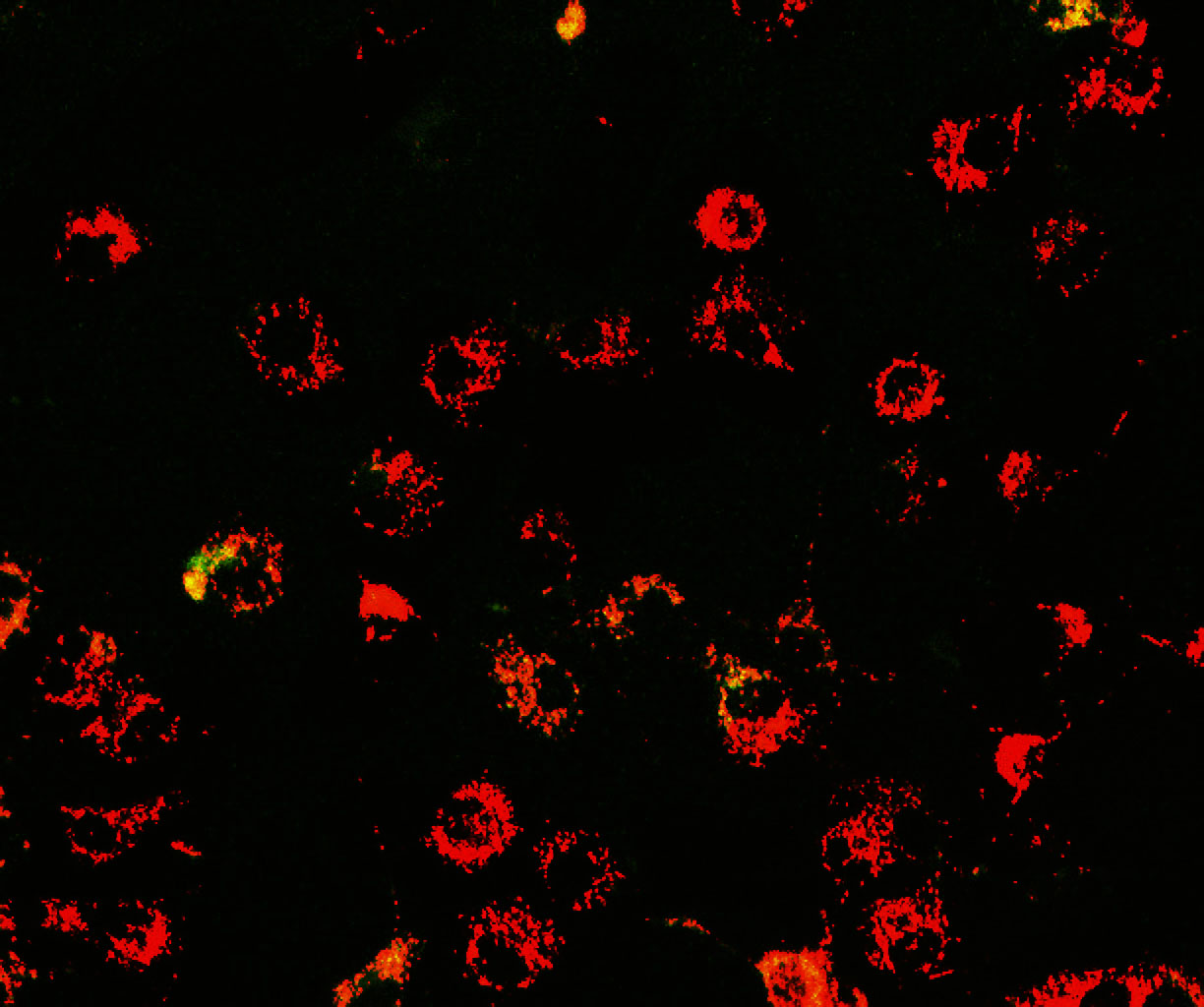

Supplement: Supplementary file 6 [file DataSheet_5.zip › FIG4/miR(-)/2/merge.jpg]

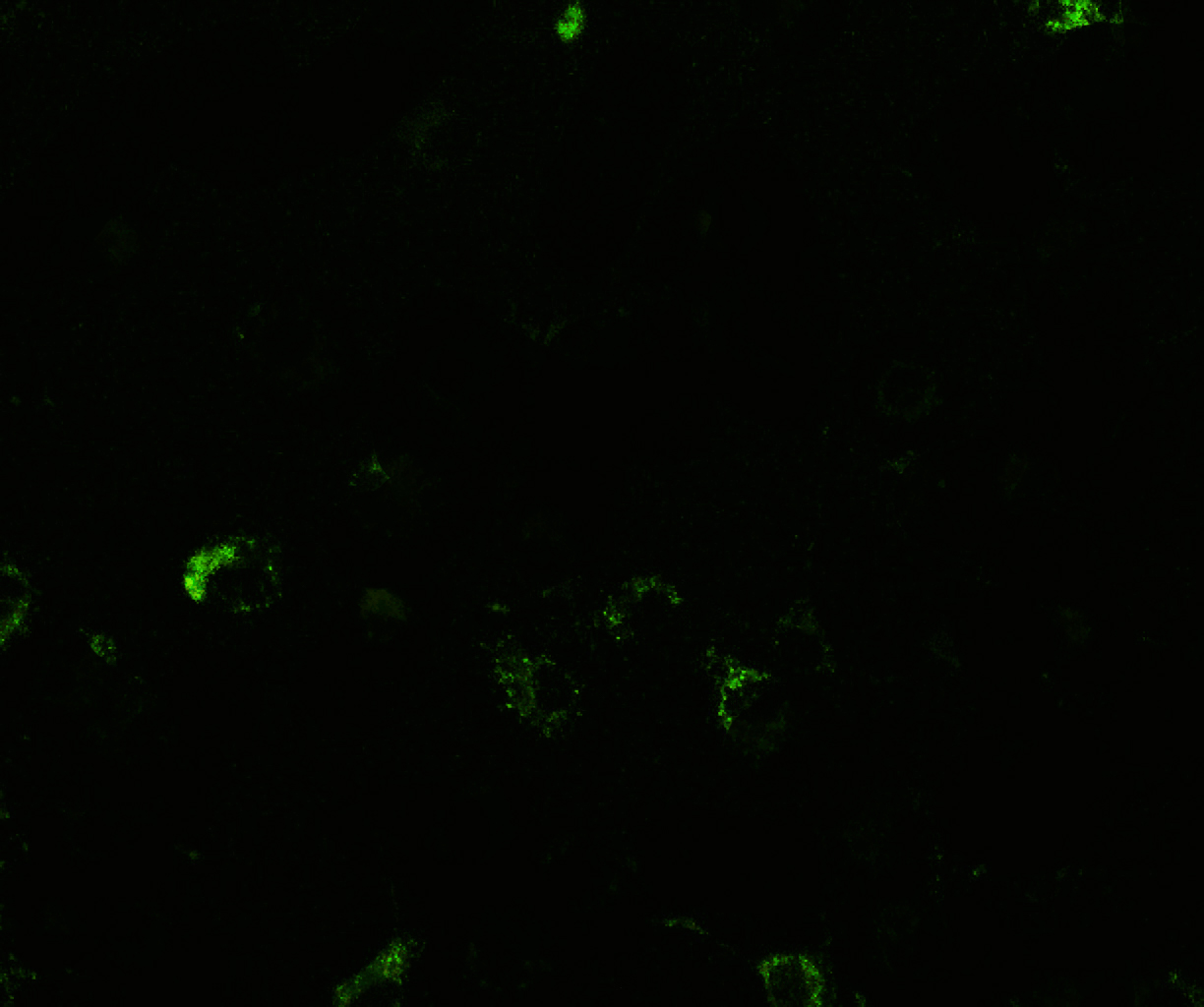

Supplement: Supplementary file 6 [file DataSheet_5.zip › FIG4/miR(-)/2/mono.jpg]

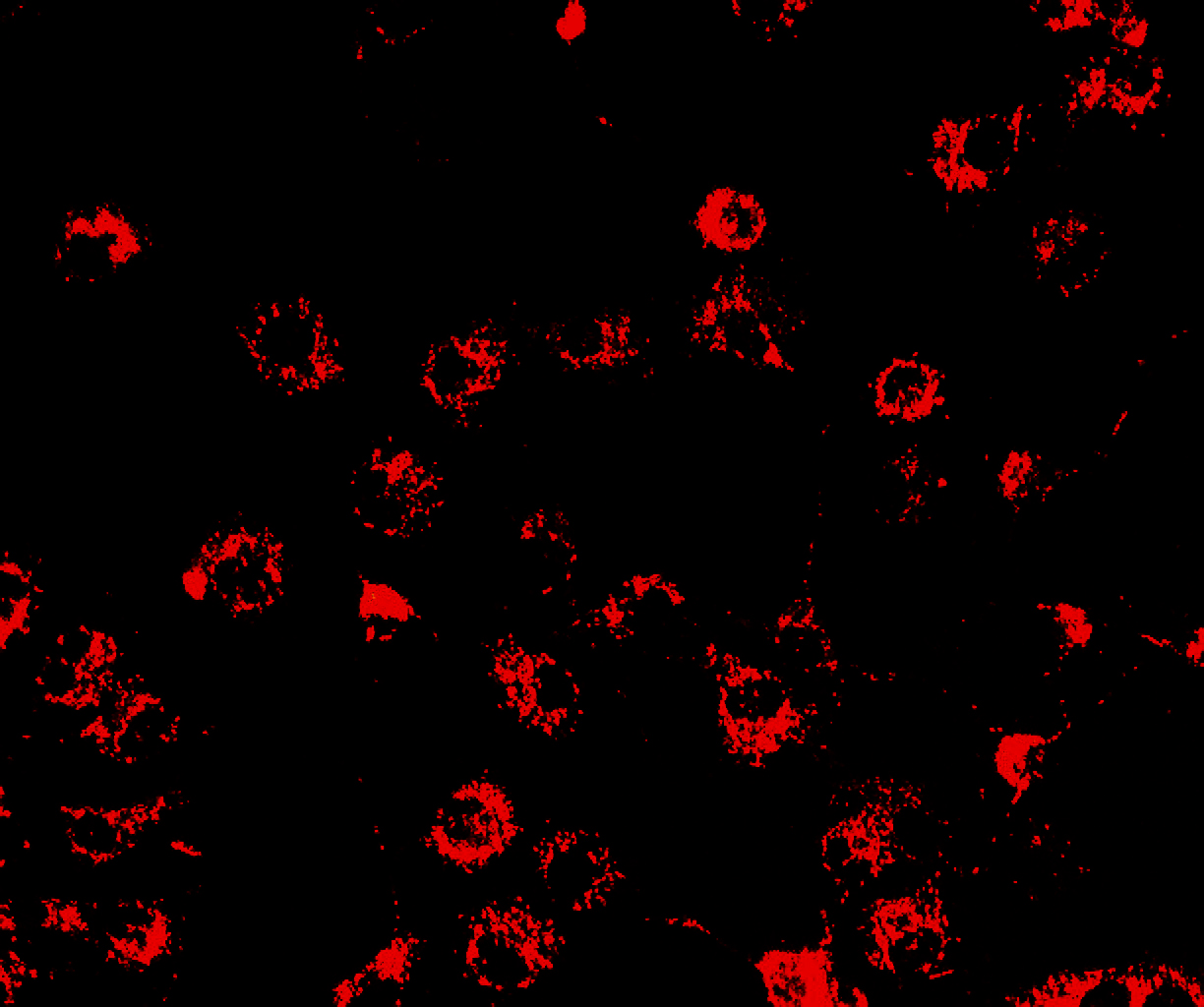

Supplement: Supplementary file 6 [file DataSheet_5.zip › FIG4/miR(-)/2/poly.jpg]

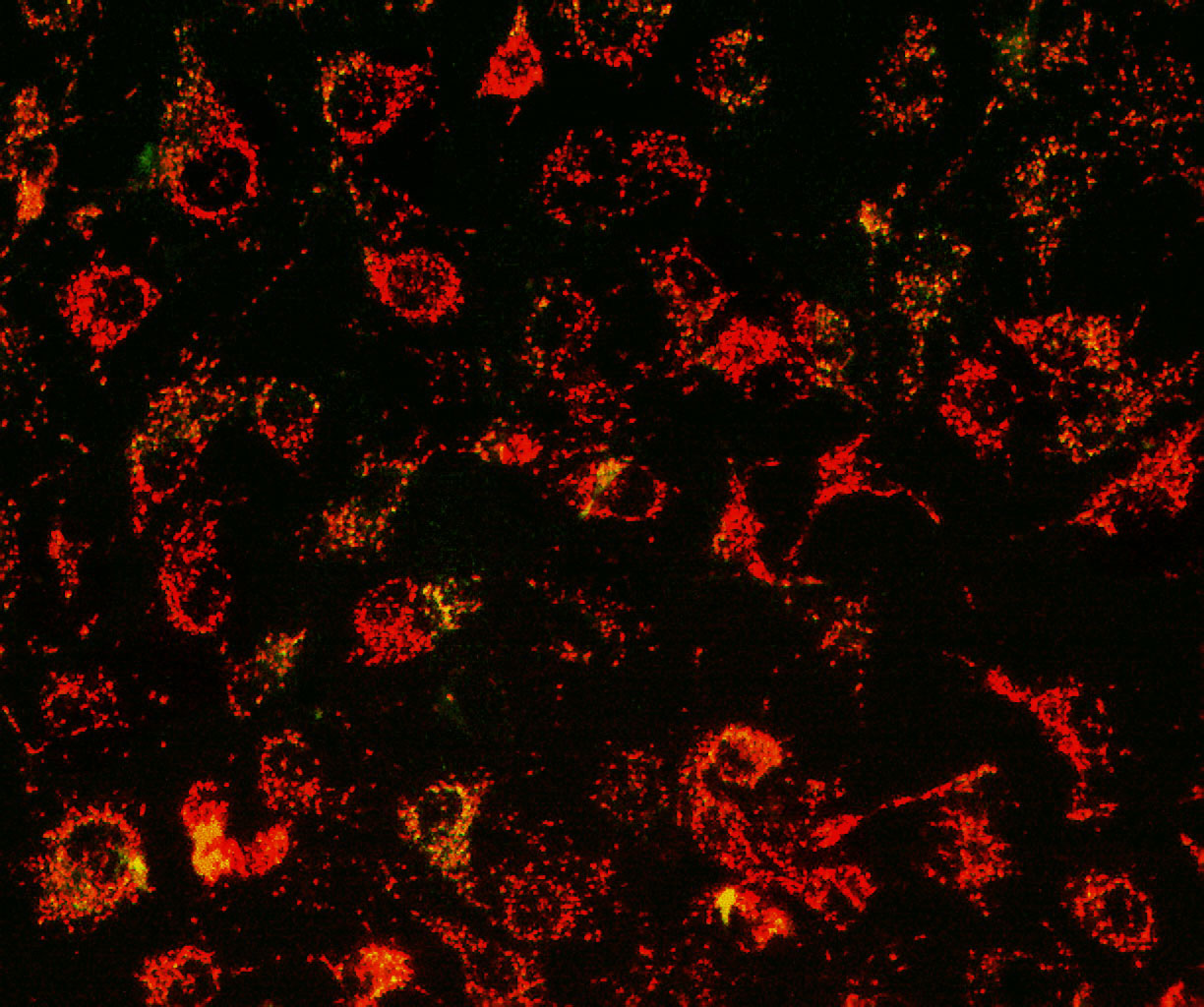

Supplement: Supplementary file 6 [file DataSheet_5.zip › FIG4/miR(-)/3/merge.jpg]

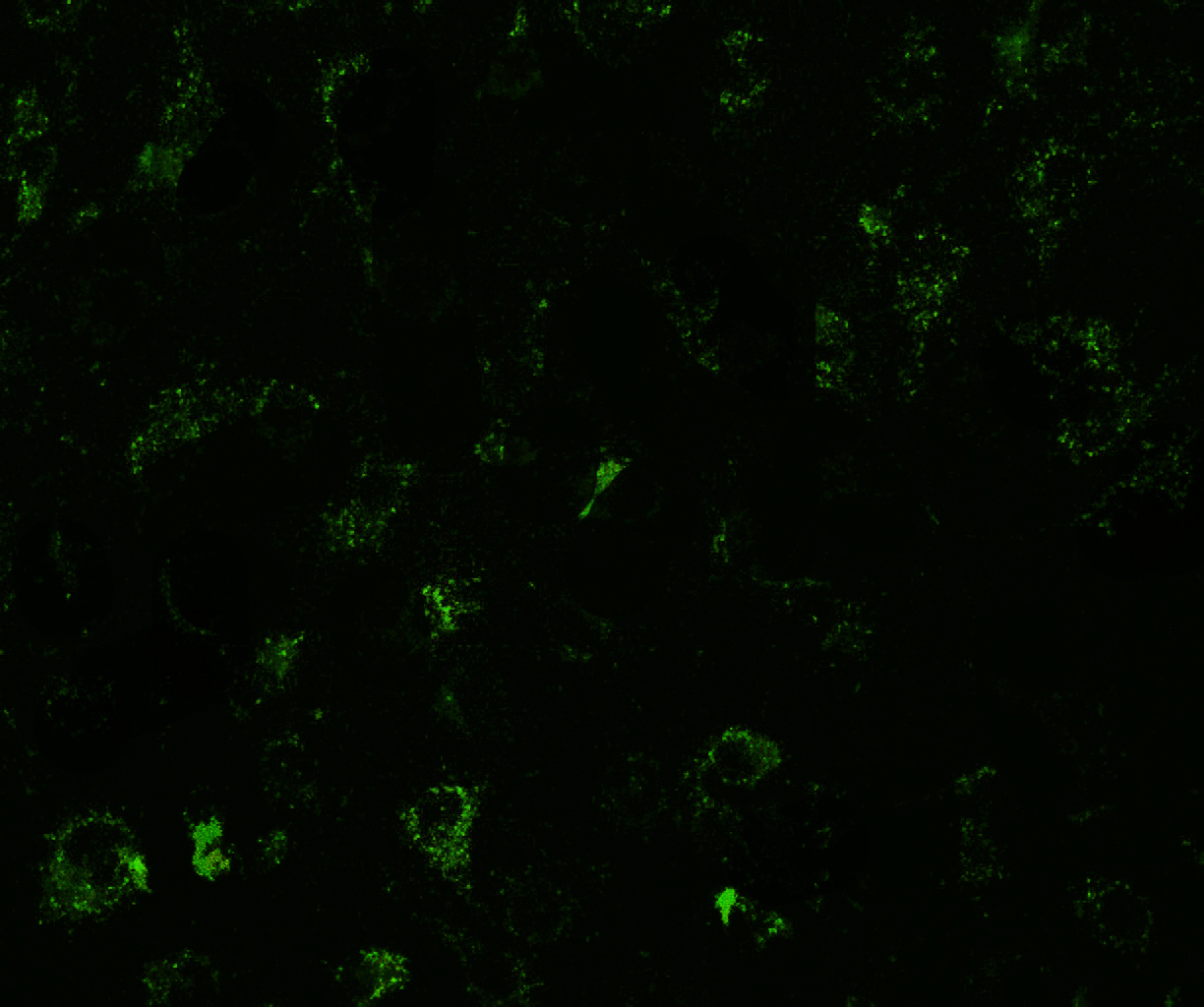

Supplement: Supplementary file 6 [file DataSheet_5.zip › FIG4/miR(-)/3/mono.jpg]

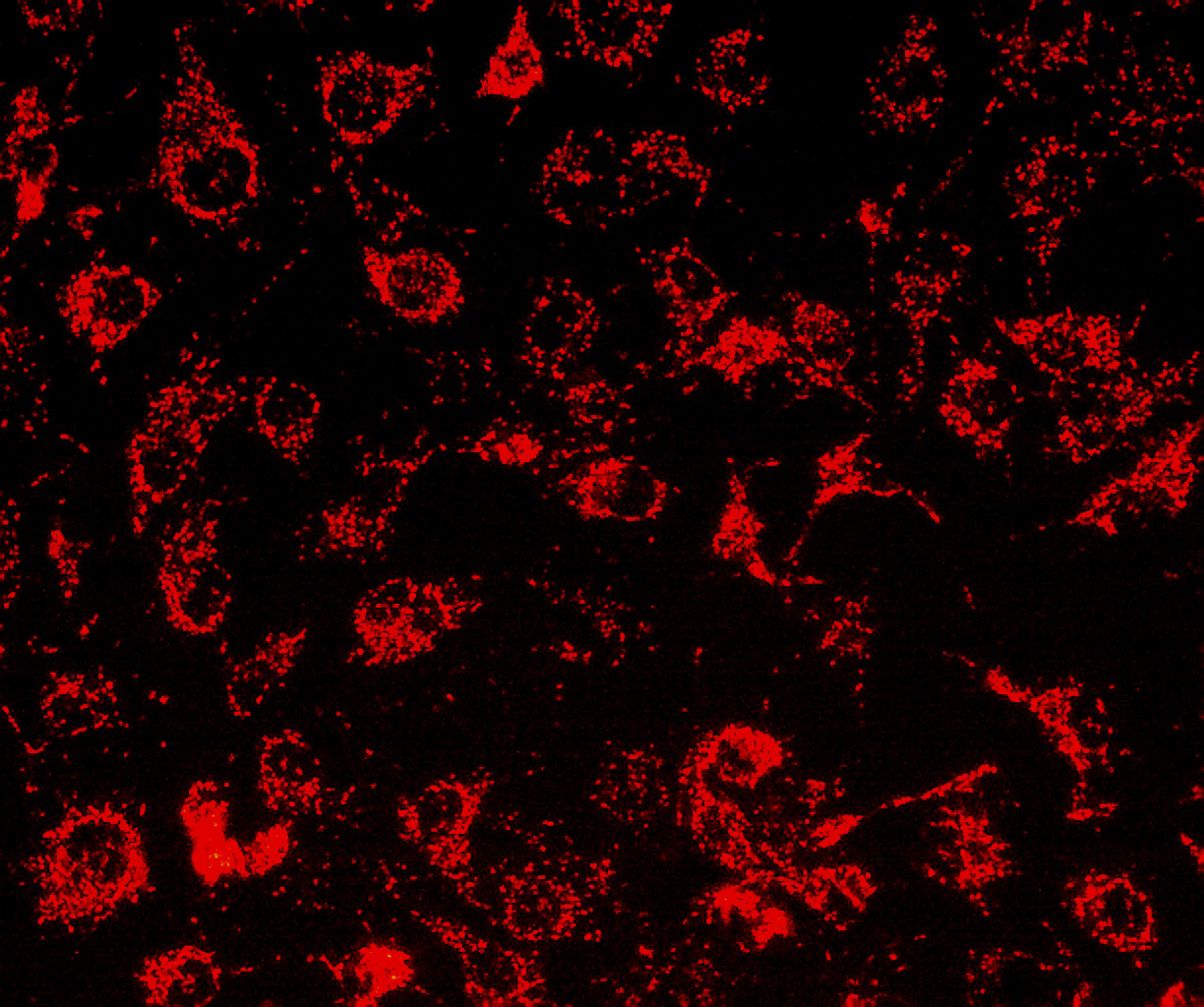

Supplement: Supplementary file 6 [file DataSheet_5.zip › FIG4/miR(-)/3/poly.jpg]

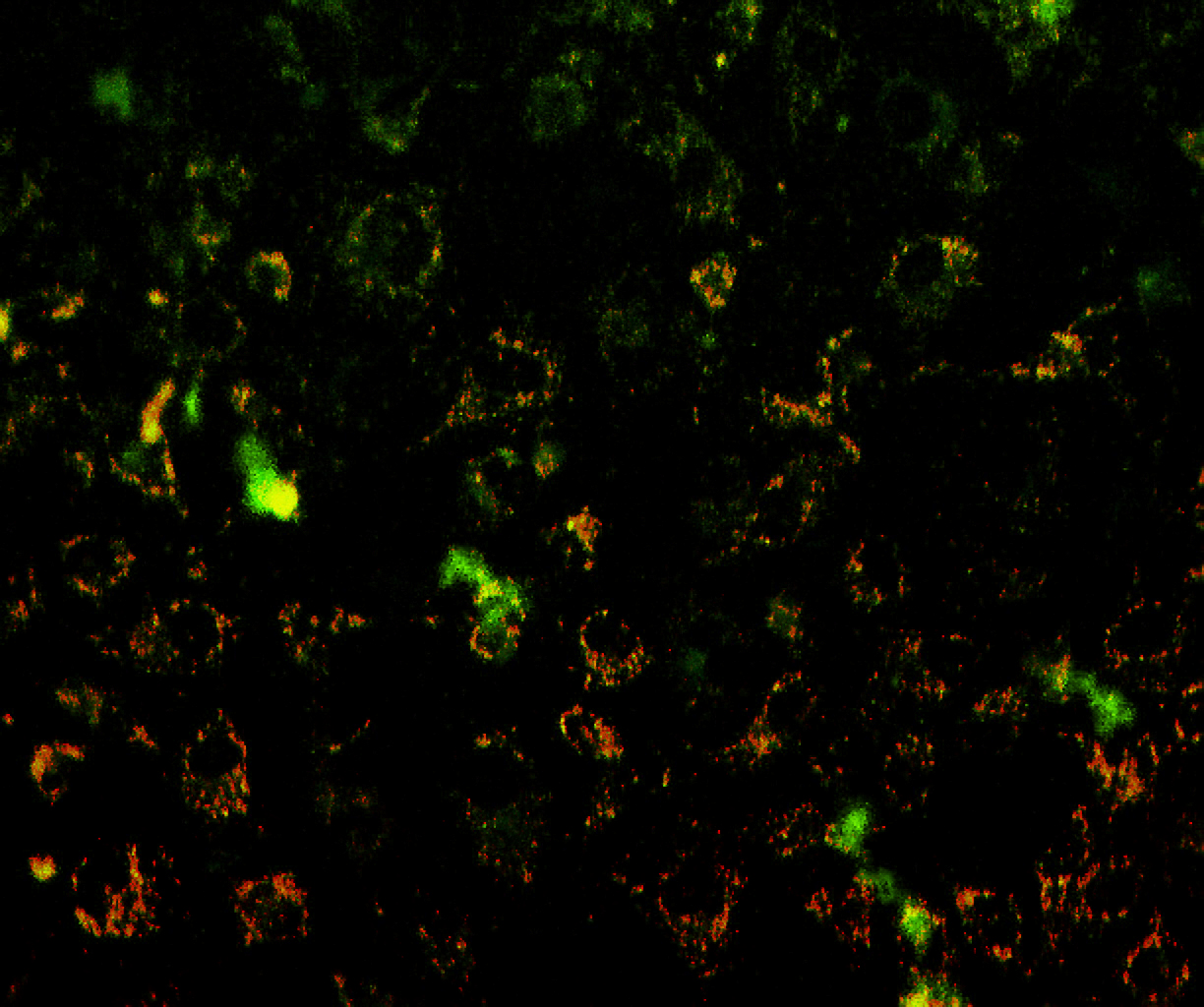

Supplement: Supplementary file 6 [file DataSheet_5.zip › FIG4/miR(+)/1/merge.jpg]

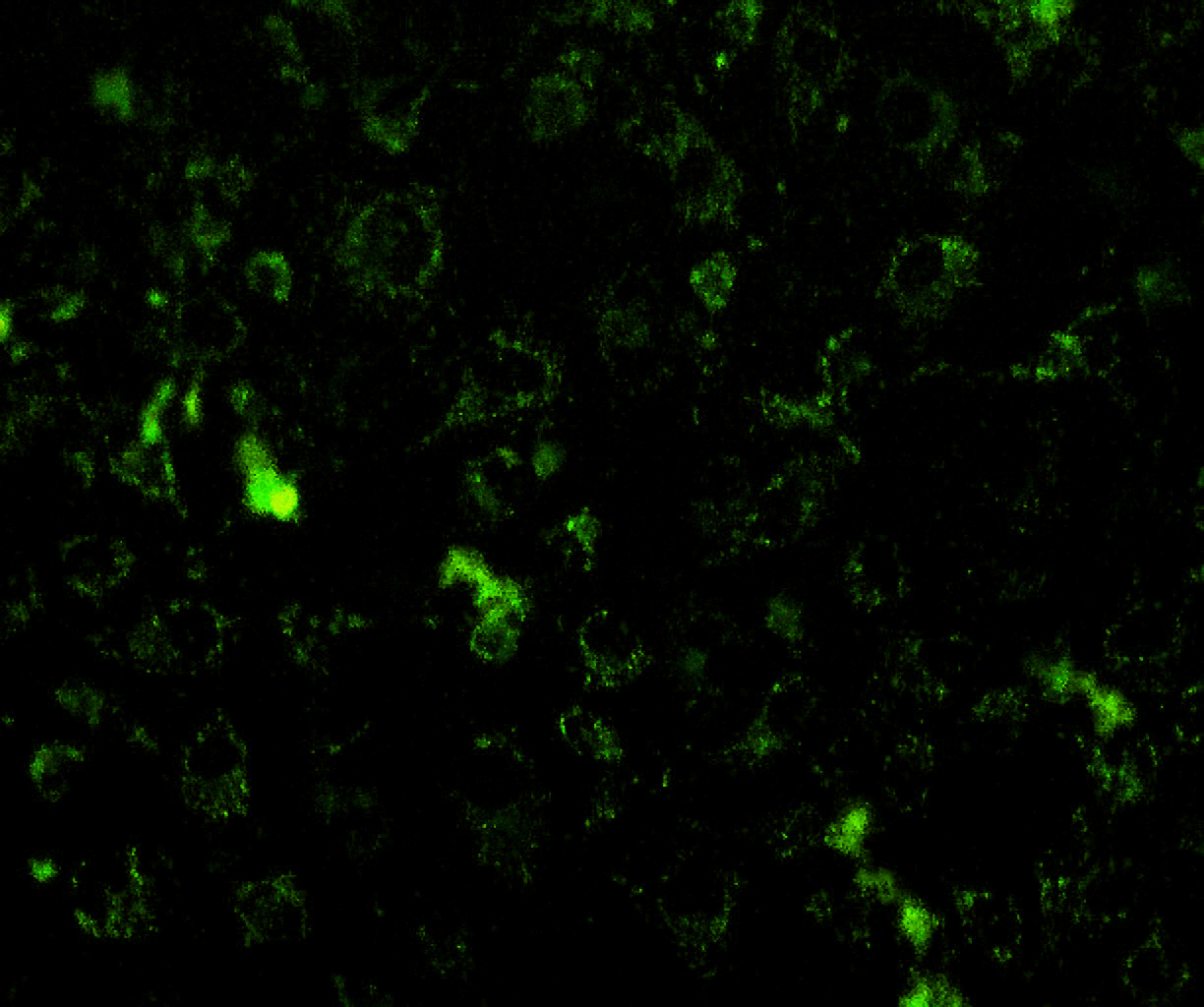

Supplement: Supplementary file 6 [file DataSheet_5.zip › FIG4/miR(+)/1/mono.jpg]

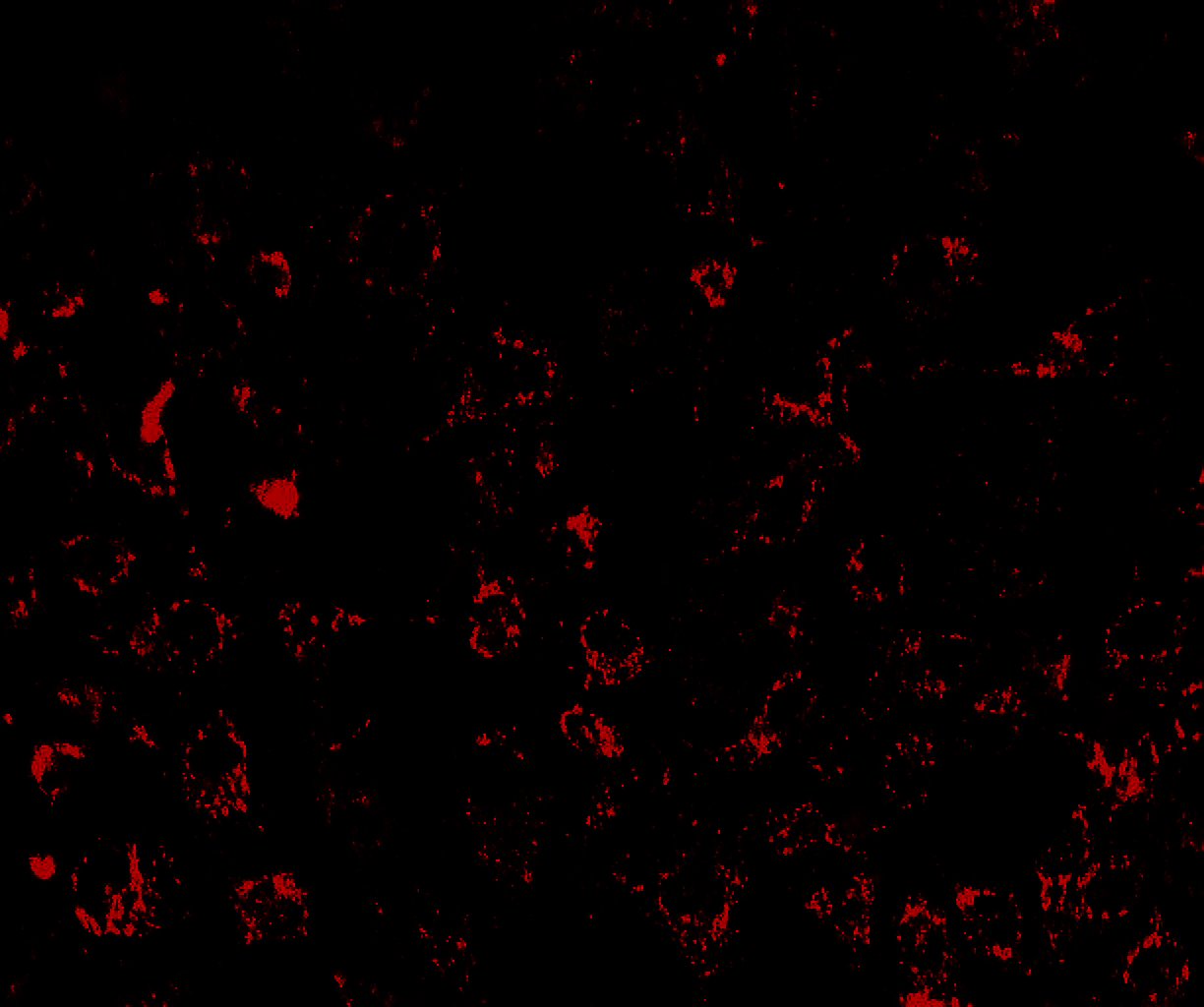

Supplement: Supplementary file 6 [file DataSheet_5.zip › FIG4/miR(+)/1/poly.jpg]

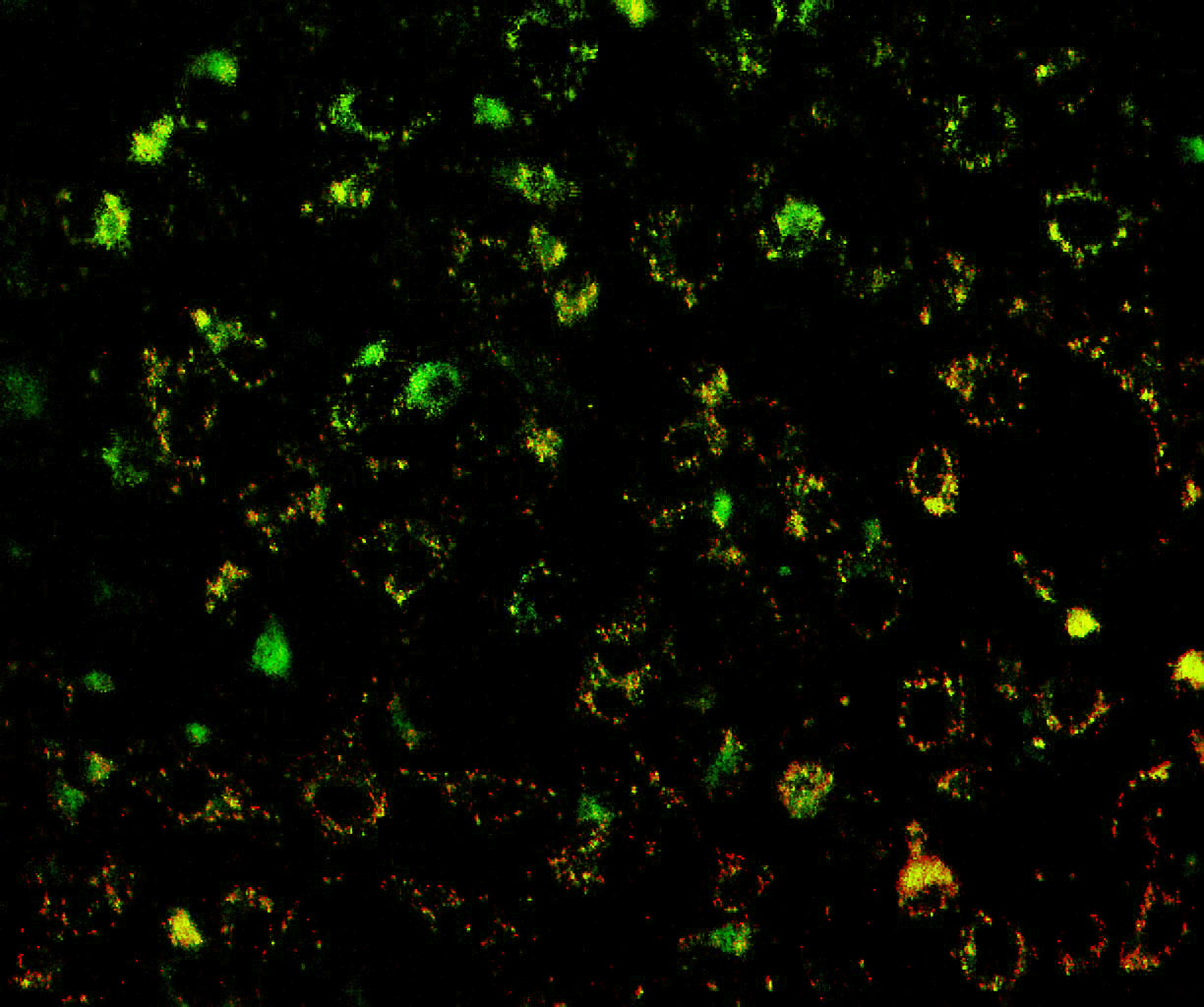

Supplement: Supplementary file 6 [file DataSheet_5.zip › FIG4/miR(+)/2/merge.jpg]

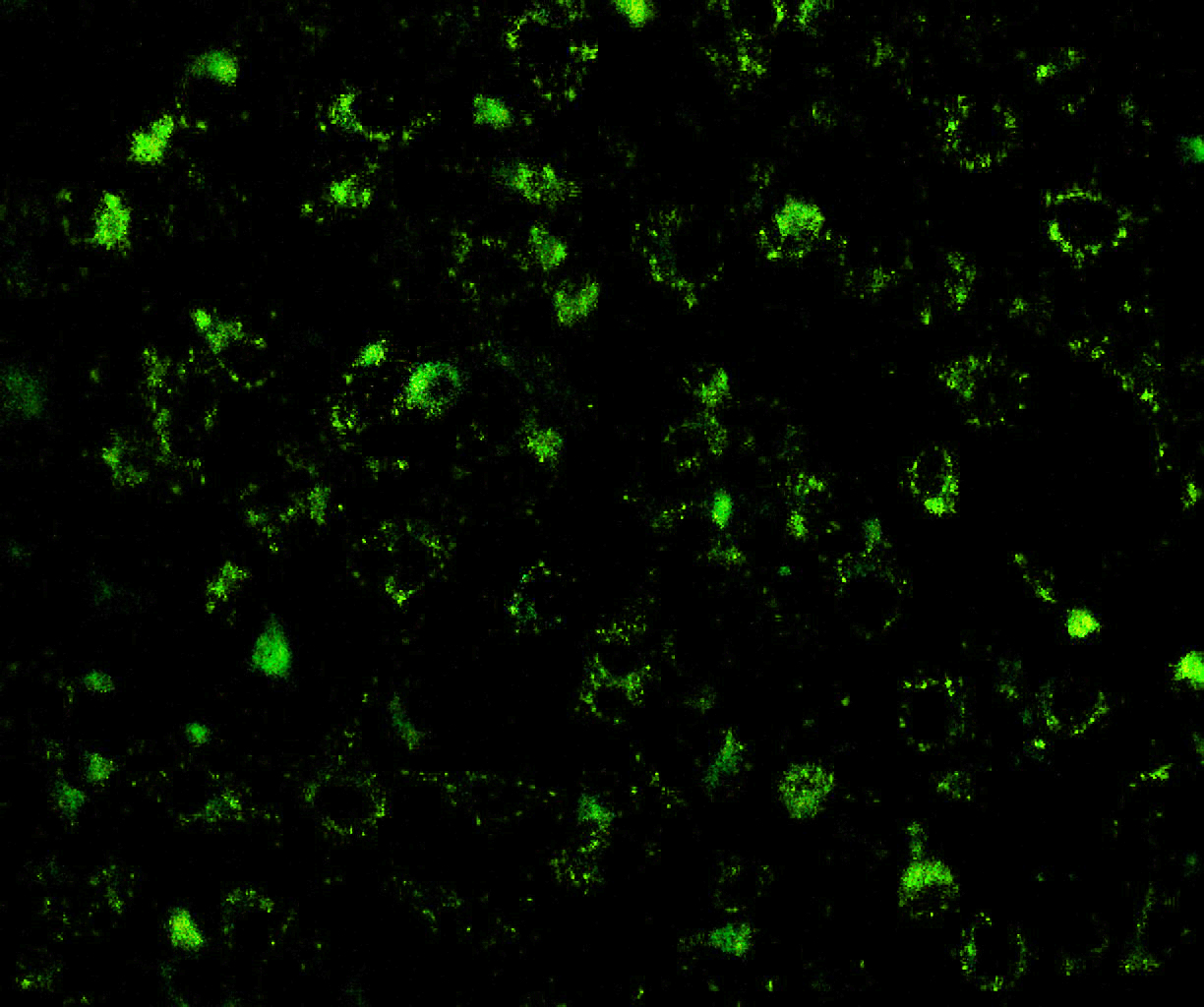

Supplement: Supplementary file 6 [file DataSheet_5.zip › FIG4/miR(+)/2/mono.jpg]

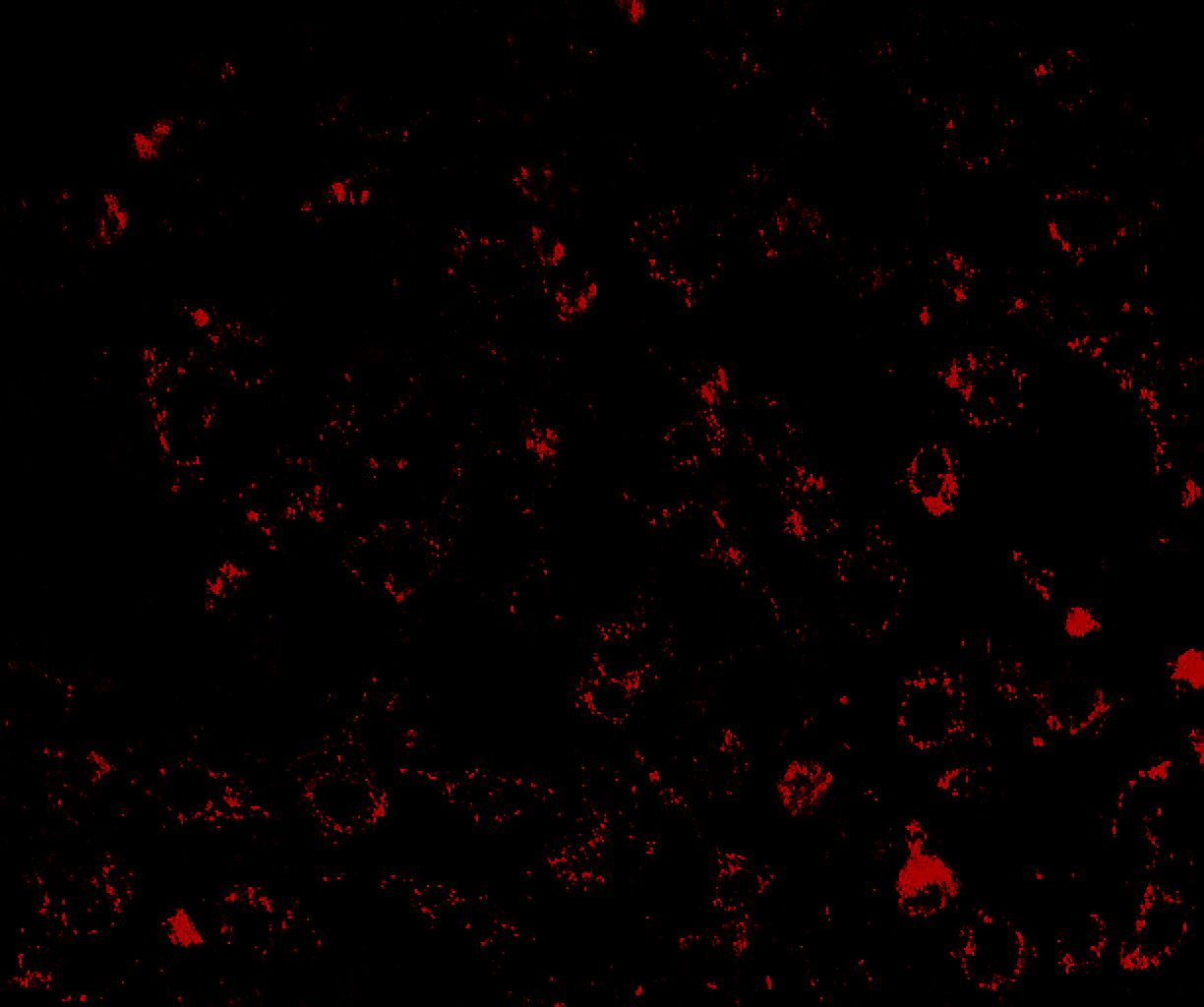

Supplement: Supplementary file 6 [file DataSheet_5.zip › FIG4/miR(+)/2/poly.jpg]

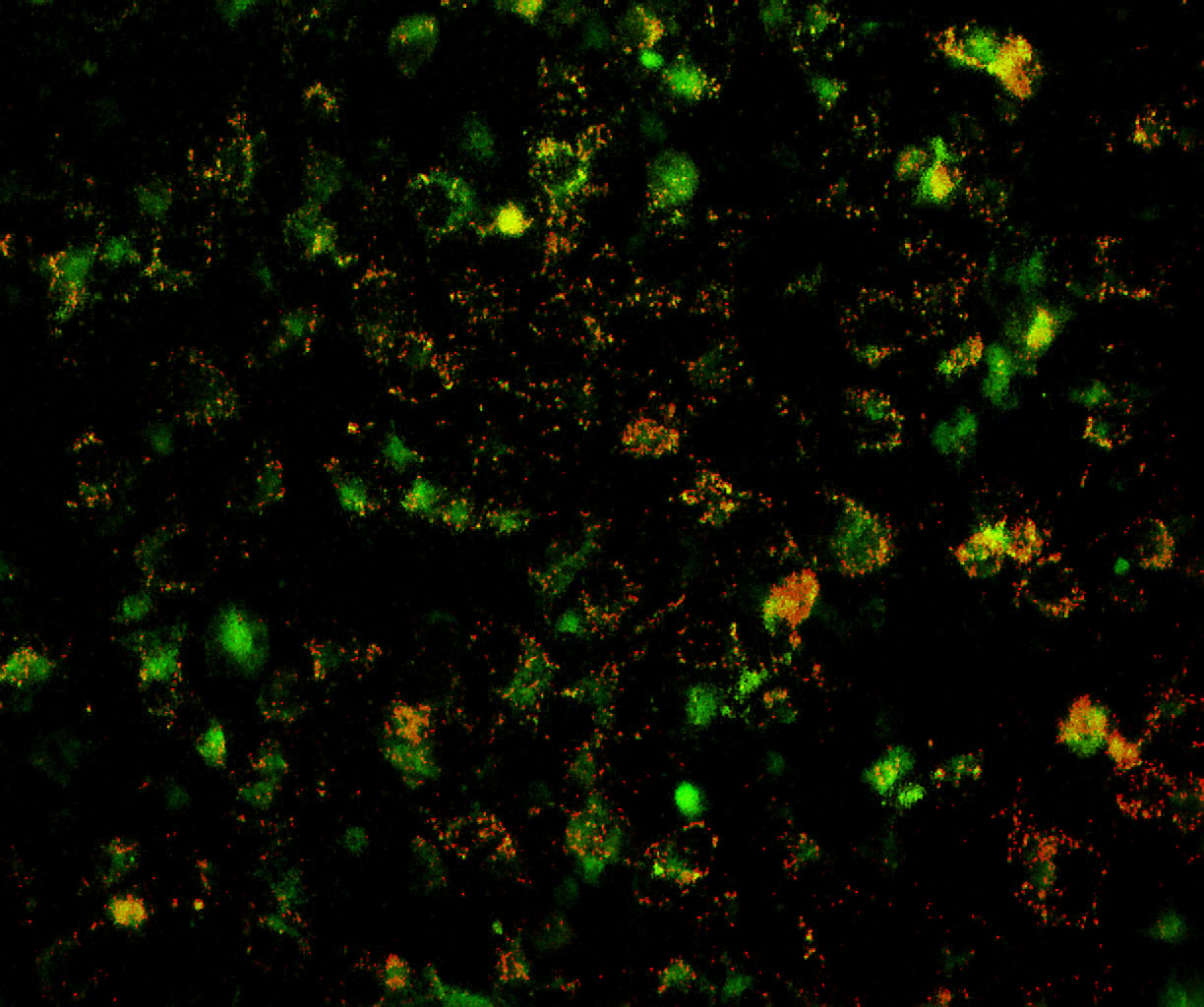

Supplement: Supplementary file 6 [file DataSheet_5.zip › FIG4/miR(+)/3/merge.jpg]

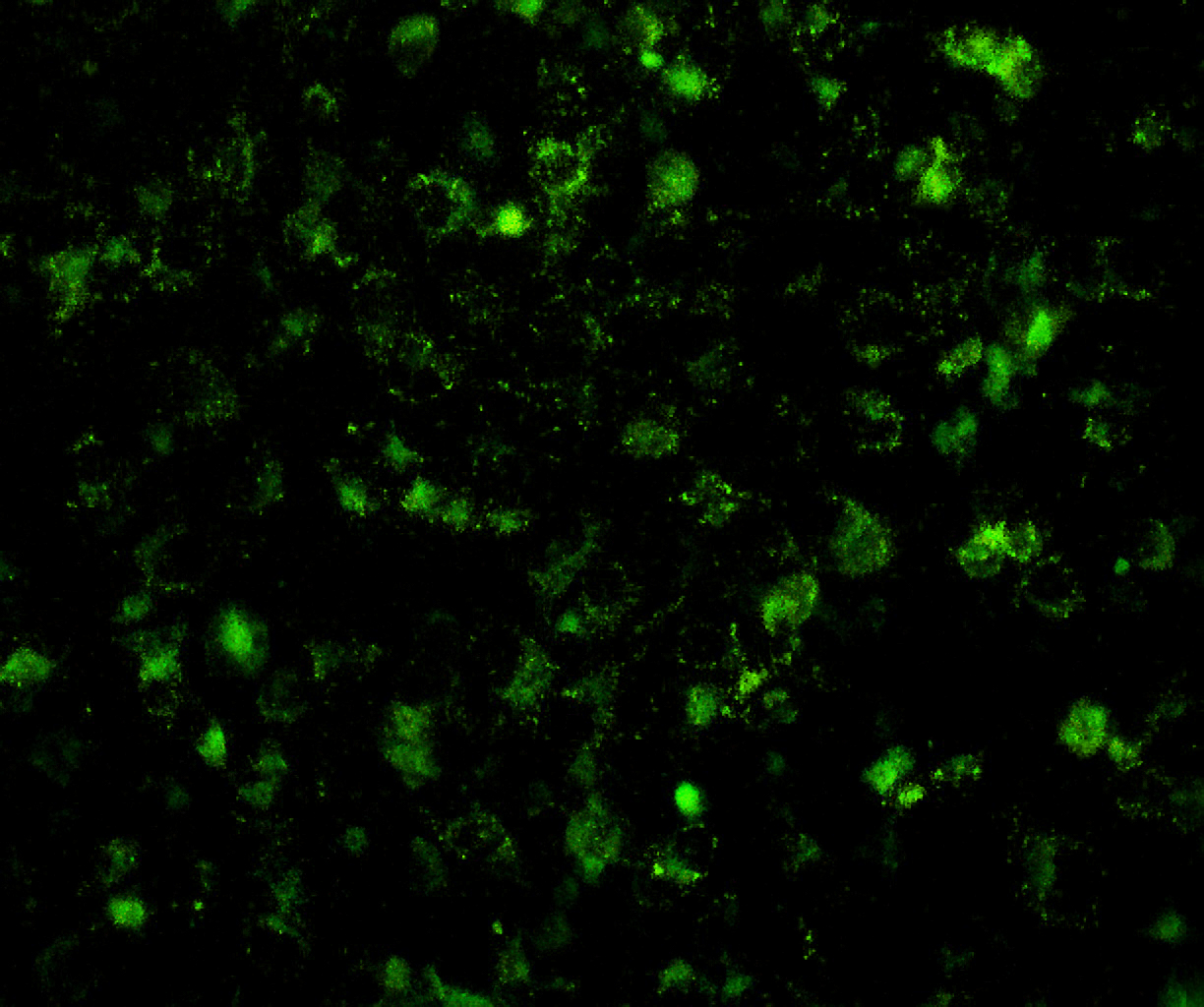

Supplement: Supplementary file 6 [file DataSheet_5.zip › FIG4/miR(+)/3/mono.jpg]

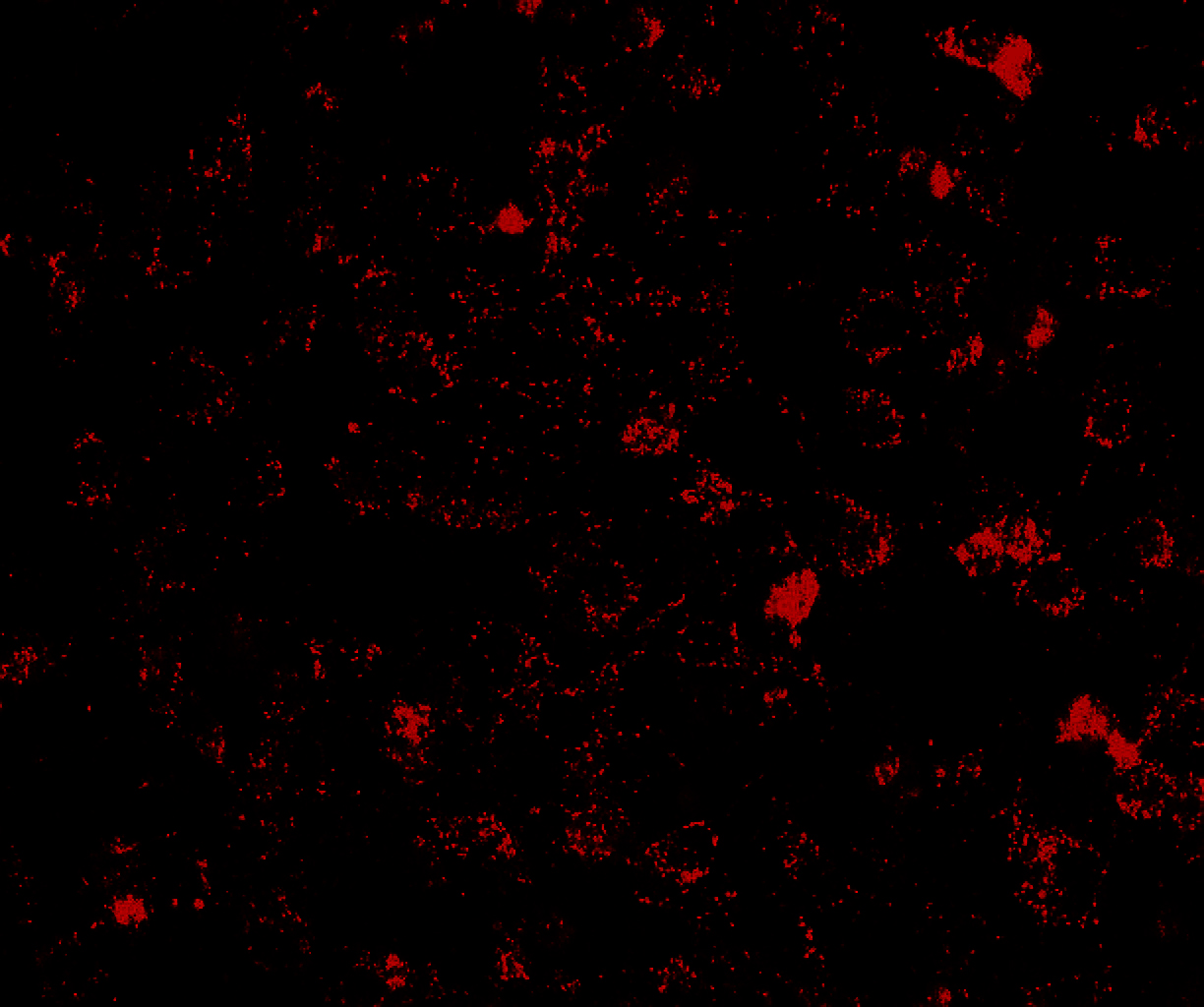

Supplement: Supplementary file 6 [file DataSheet_5.zip › FIG4/miR(+)/3/poly.jpg]

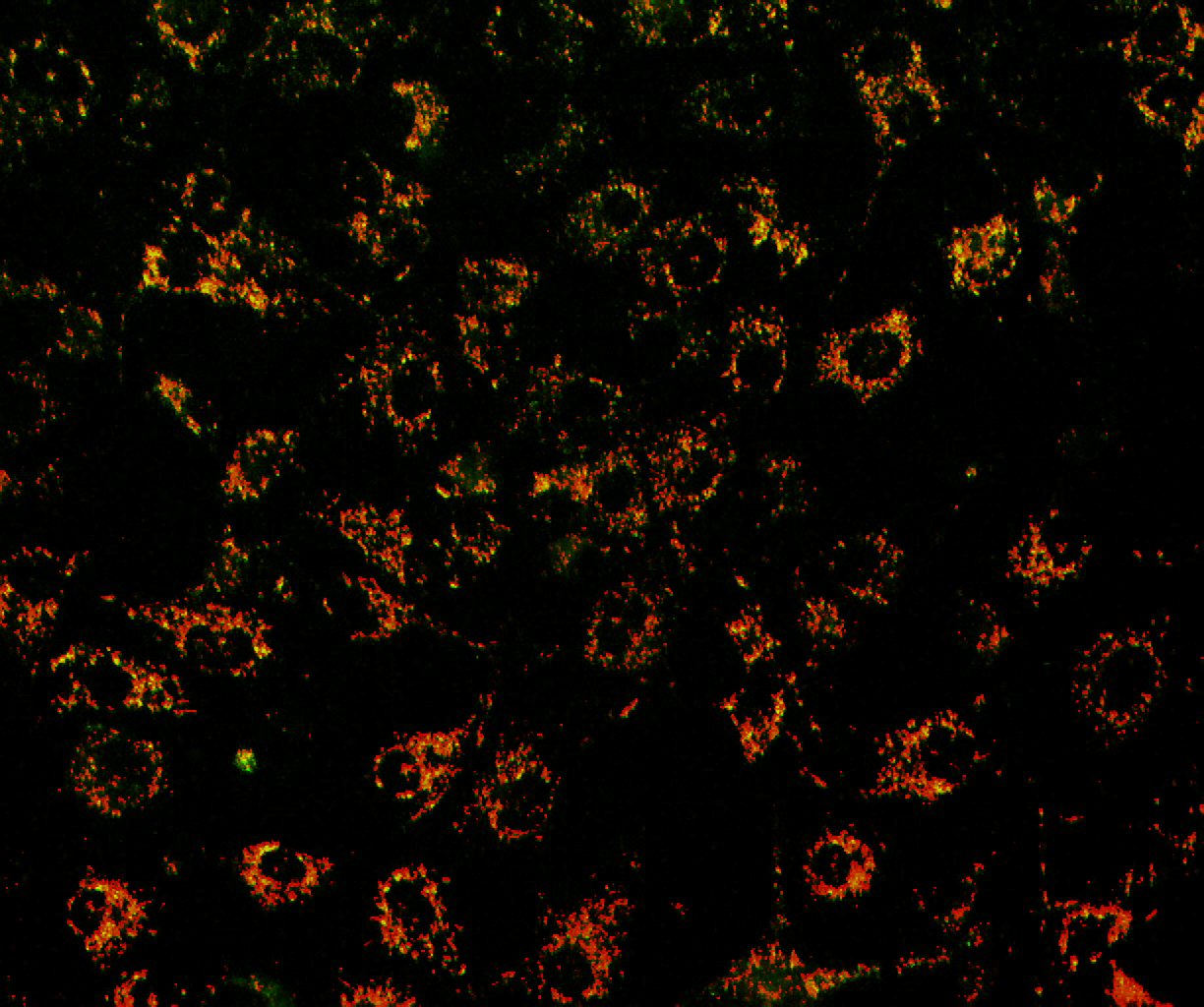

Supplement: Supplementary file 6 [file DataSheet_5.zip › FIG4/NC(-)/1/merge.jpg]

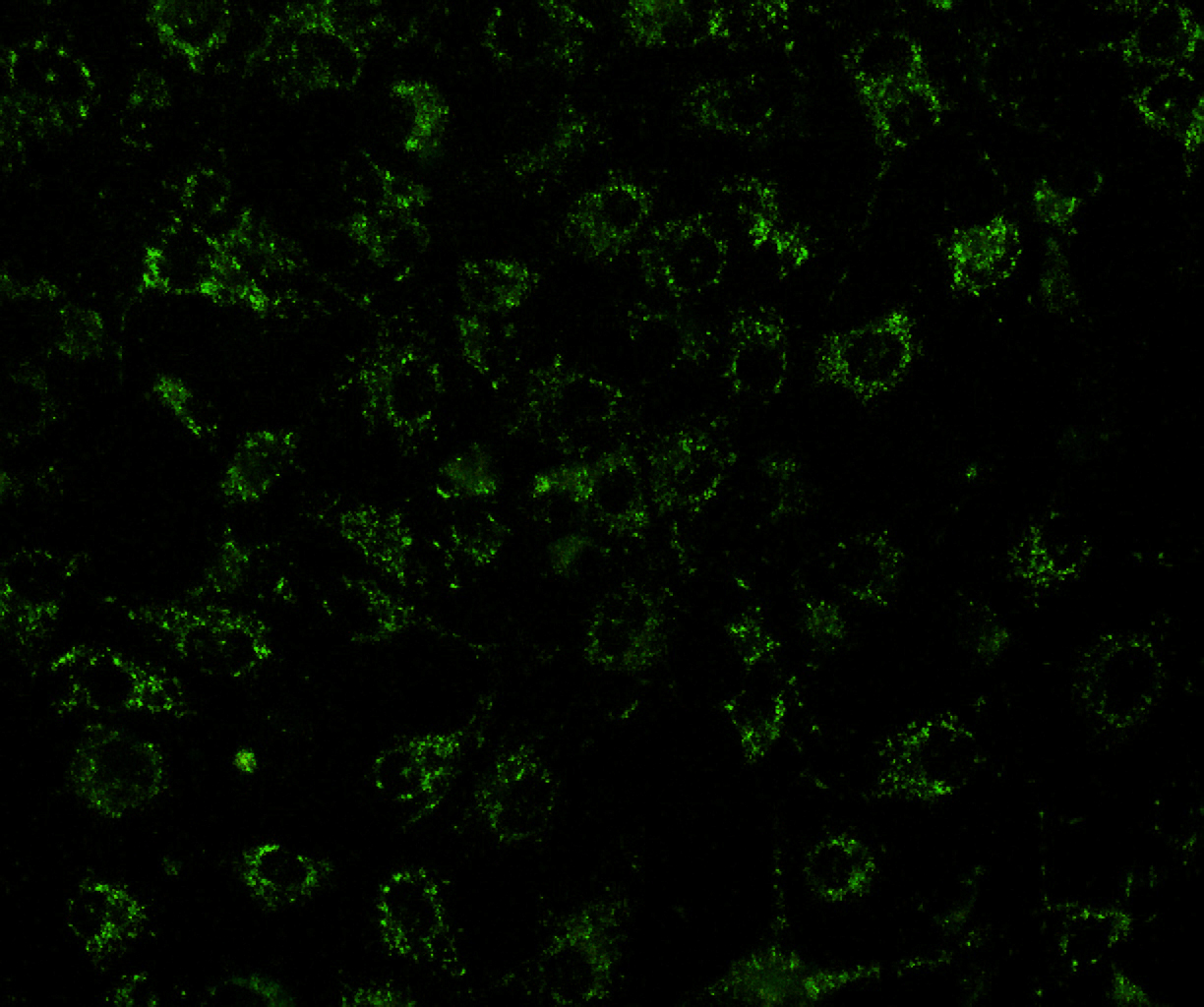

Supplement: Supplementary file 6 [file DataSheet_5.zip › FIG4/NC(-)/1/mono.jpg]

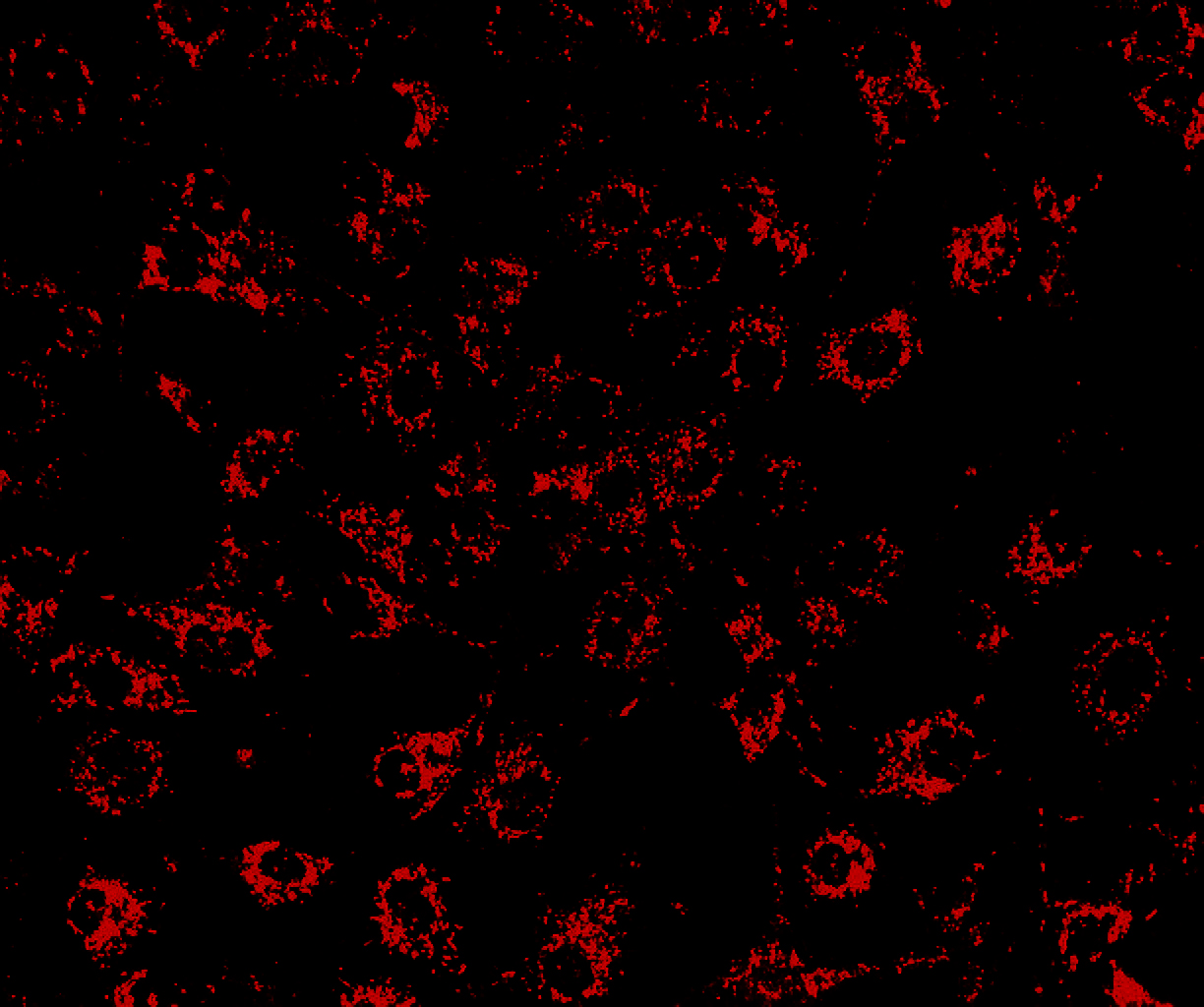

Supplement: Supplementary file 6 [file DataSheet_5.zip › FIG4/NC(-)/1/poly.jpg]

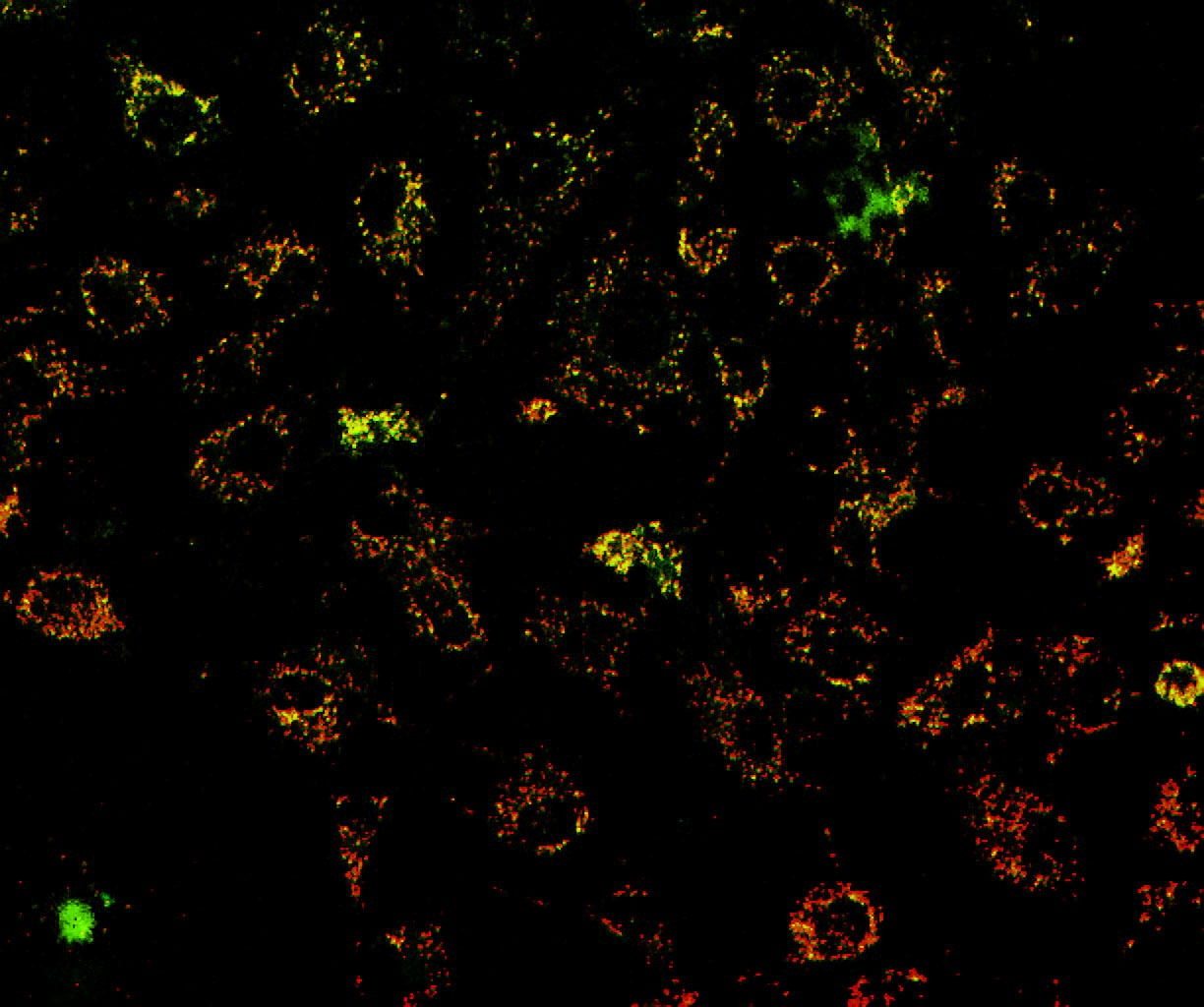

Supplement: Supplementary file 6 [file DataSheet_5.zip › FIG4/NC(-)/2/merge.jpg]

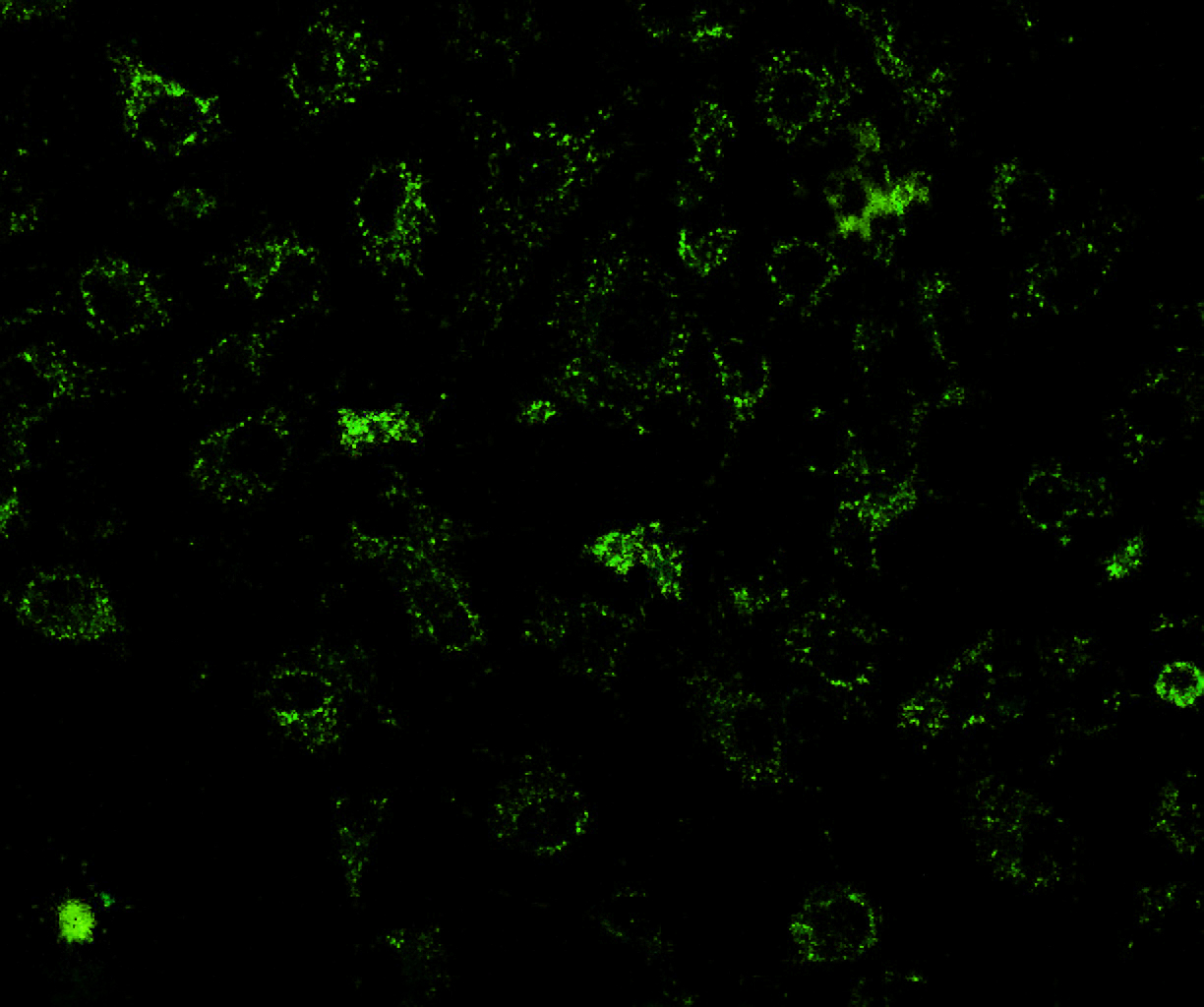

Supplement: Supplementary file 6 [file DataSheet_5.zip › FIG4/NC(-)/2/mono.jpg]

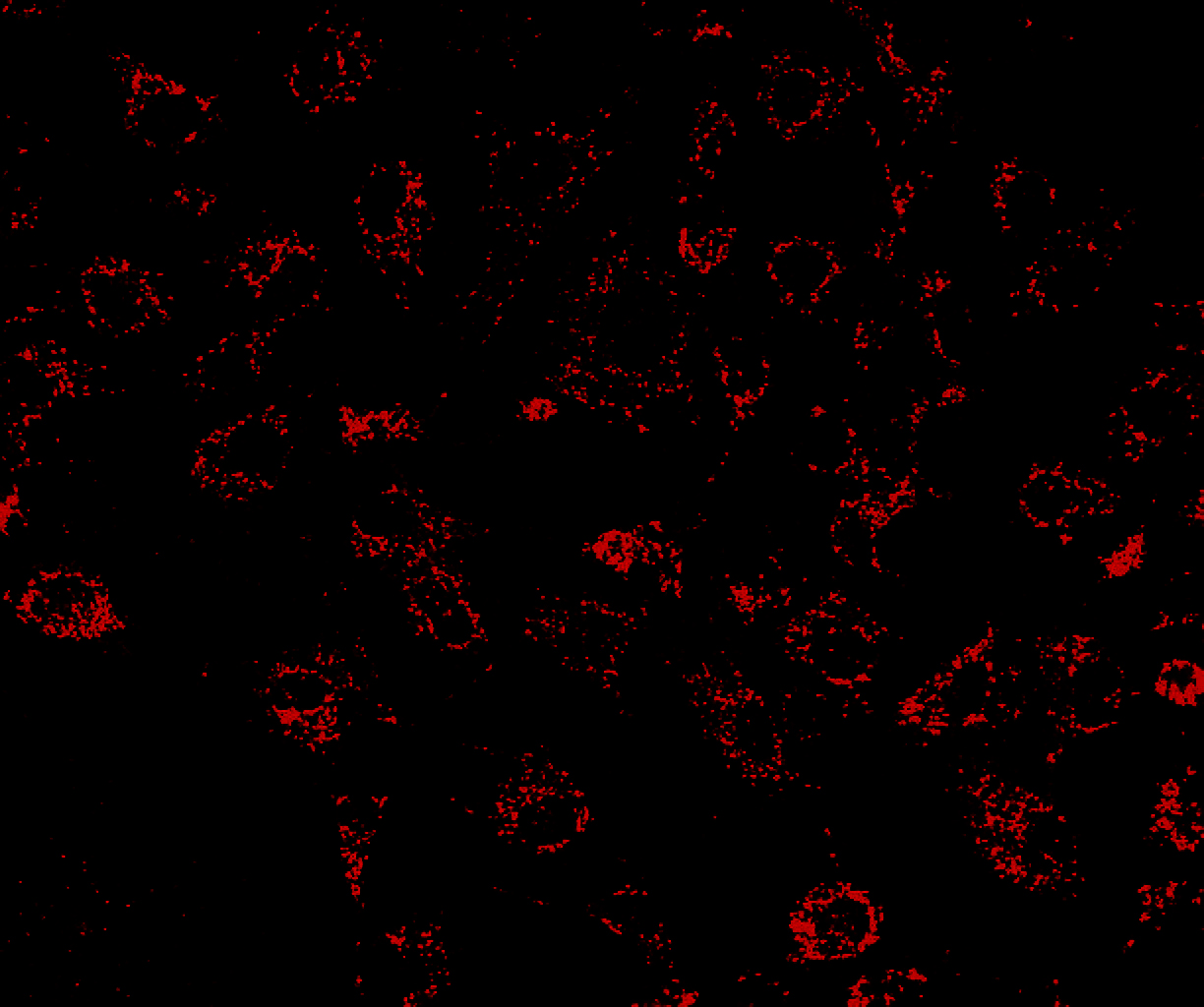

Supplement: Supplementary file 6 [file DataSheet_5.zip › FIG4/NC(-)/2/ploy.jpg]

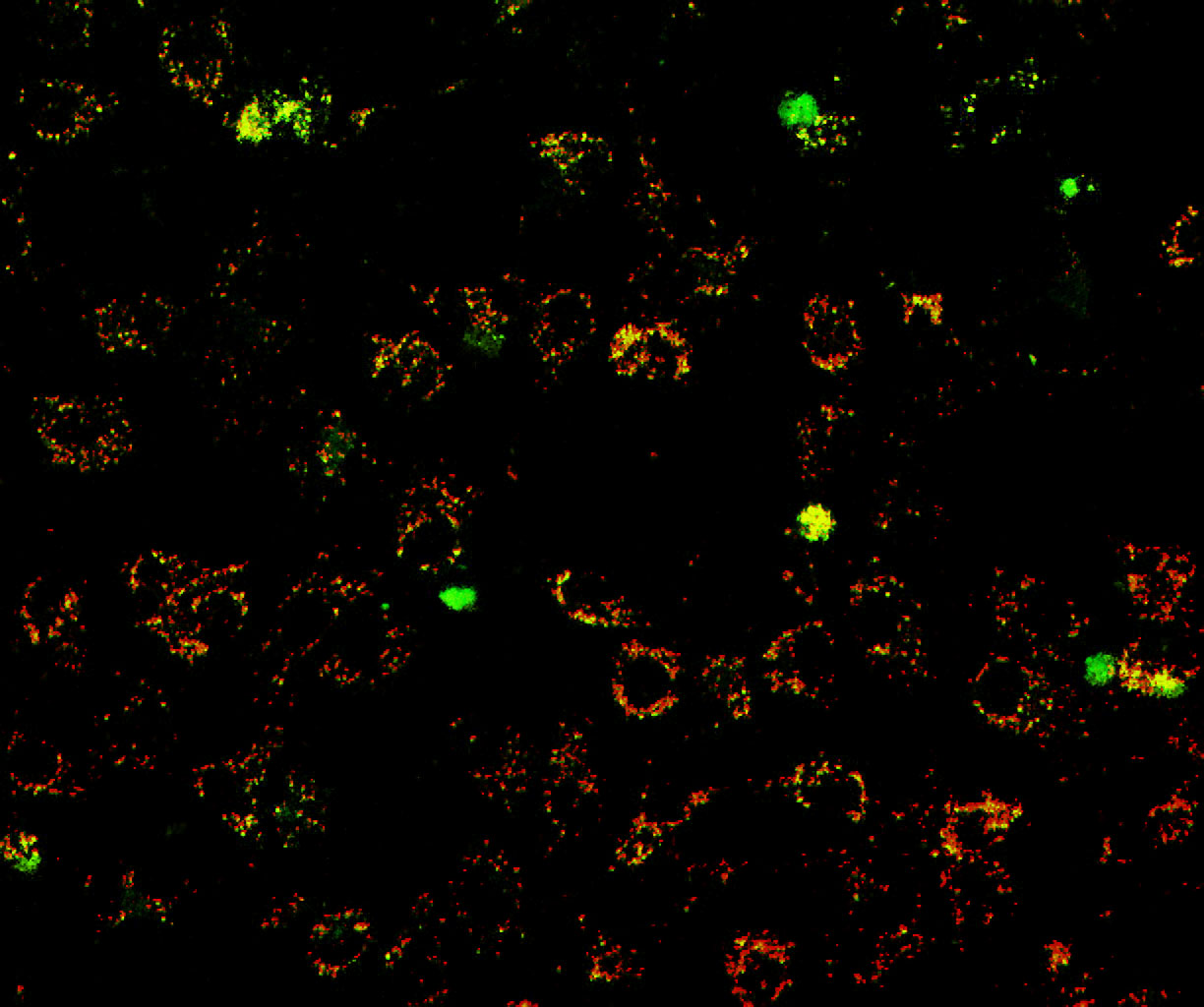

Supplement: Supplementary file 6 [file DataSheet_5.zip › FIG4/NC(-)/3/merge.jpg]

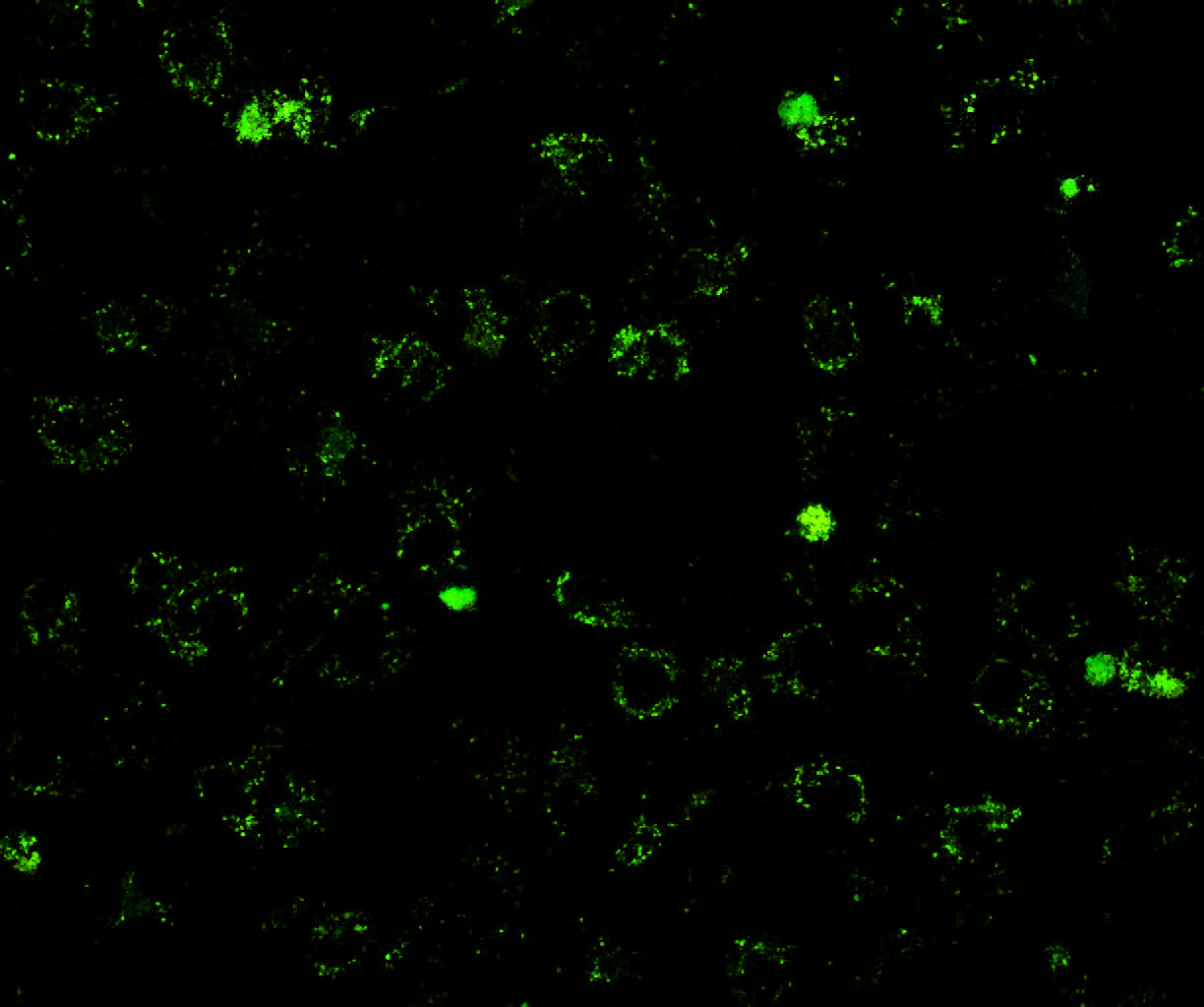

Supplement: Supplementary file 6 [file DataSheet_5.zip › FIG4/NC(-)/3/mono.jpg]

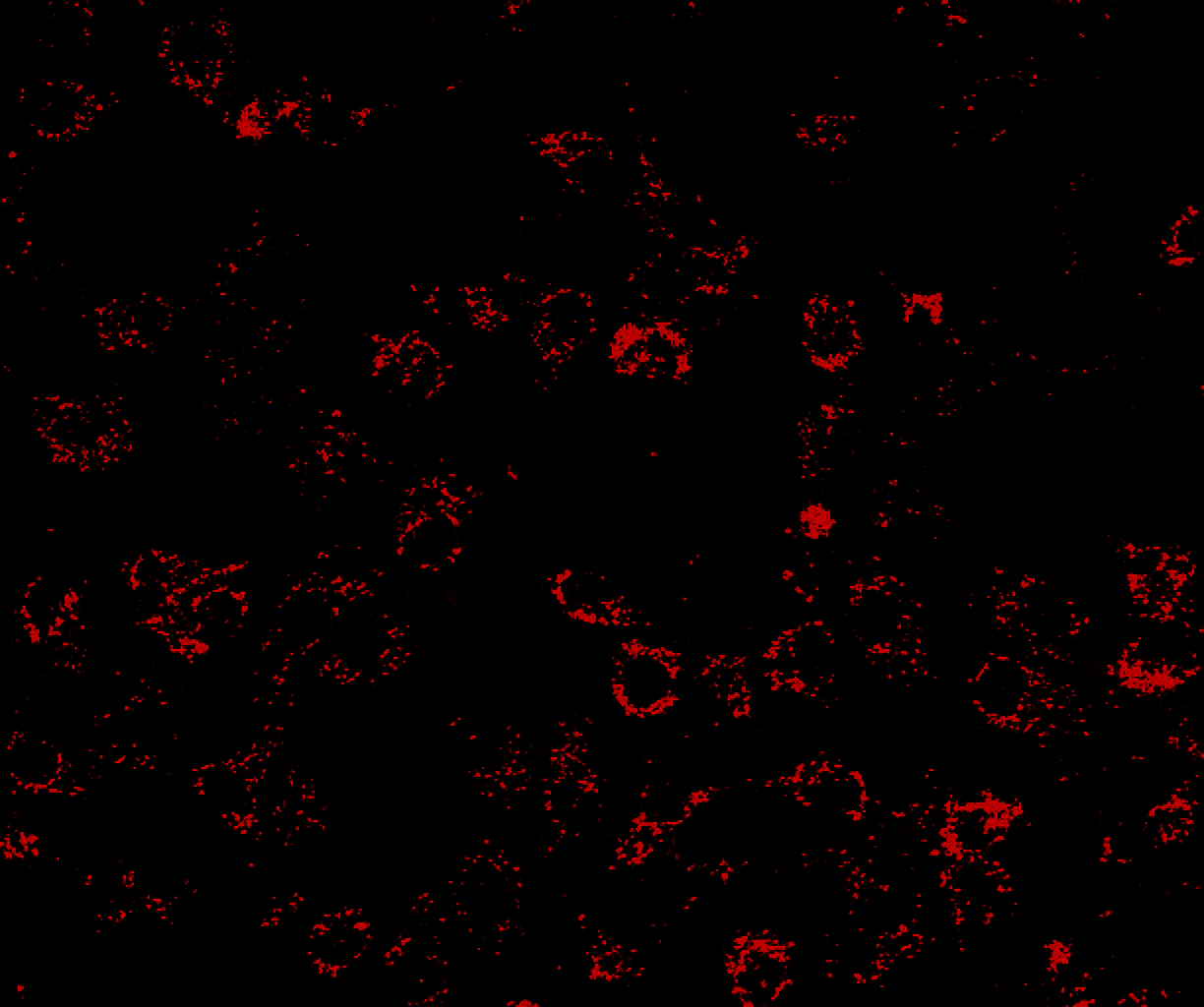

Supplement: Supplementary file 6 [file DataSheet_5.zip › FIG4/NC(-)/3/poly.jpg]

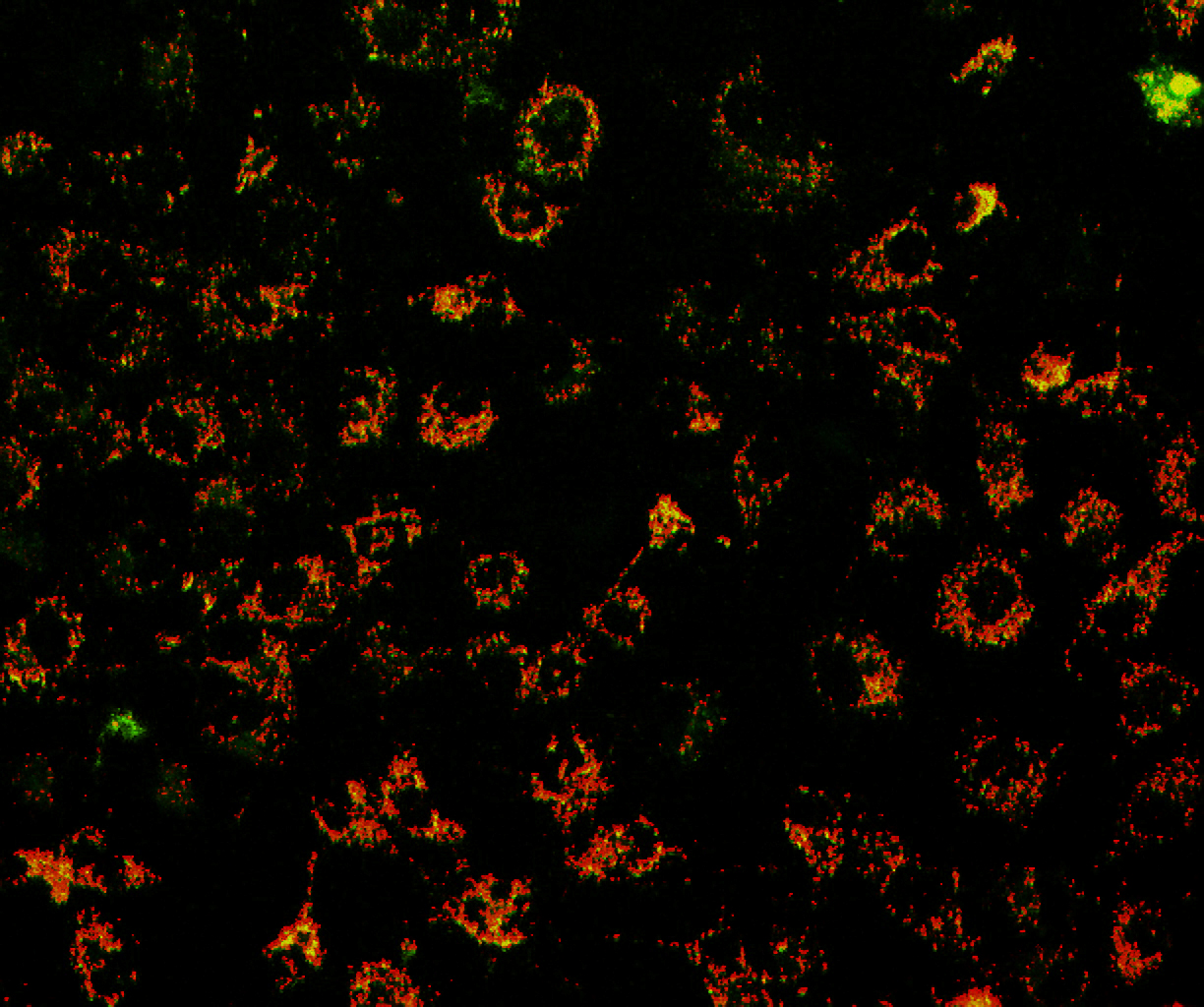

Supplement: Supplementary file 6 [file DataSheet_5.zip › FIG4/NC(+)/1/merge.jpg]

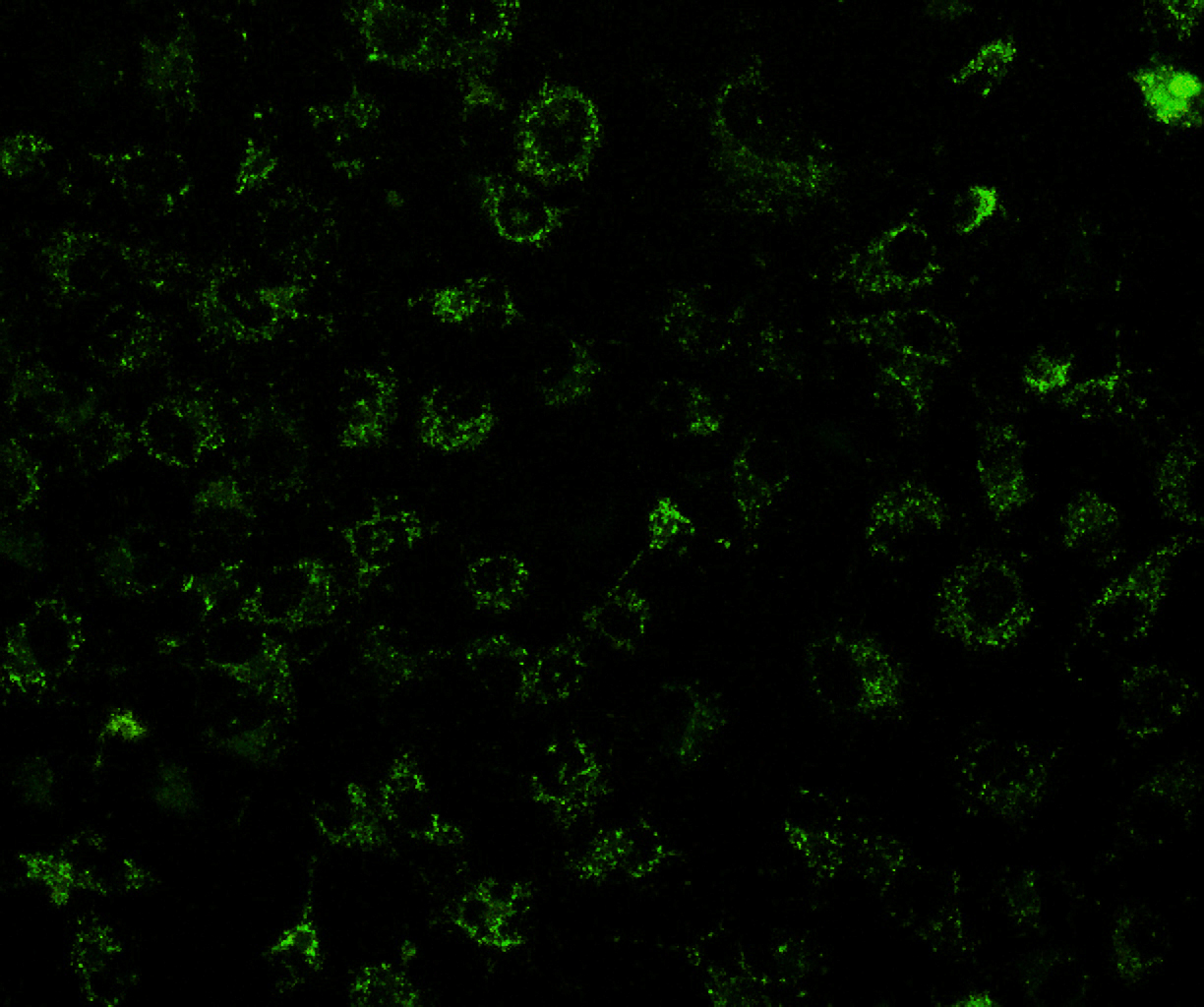

Supplement: Supplementary file 6 [file DataSheet_5.zip › FIG4/NC(+)/1/mono.jpg]

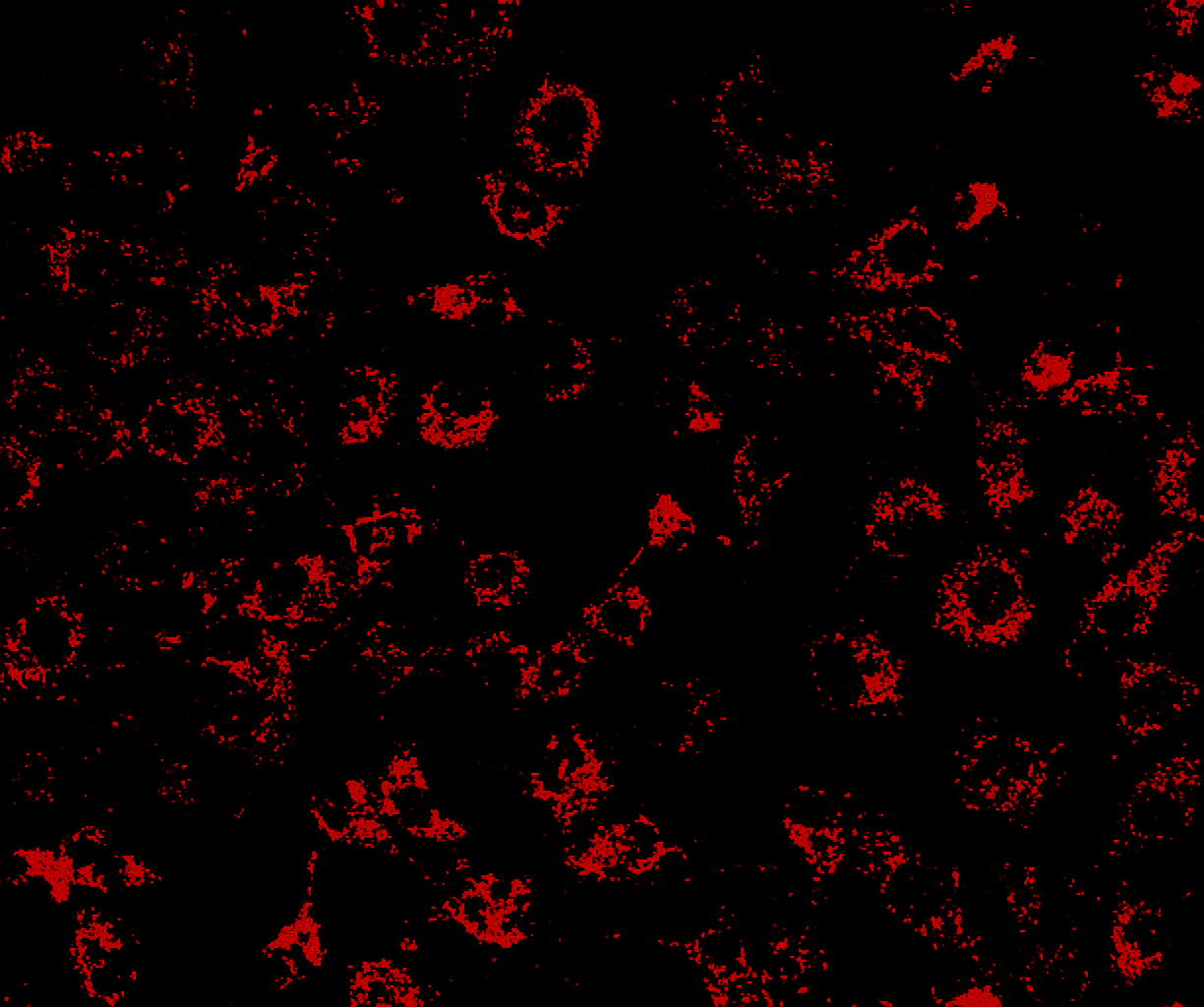

Supplement: Supplementary file 6 [file DataSheet_5.zip › FIG4/NC(+)/1/poly.jpg]

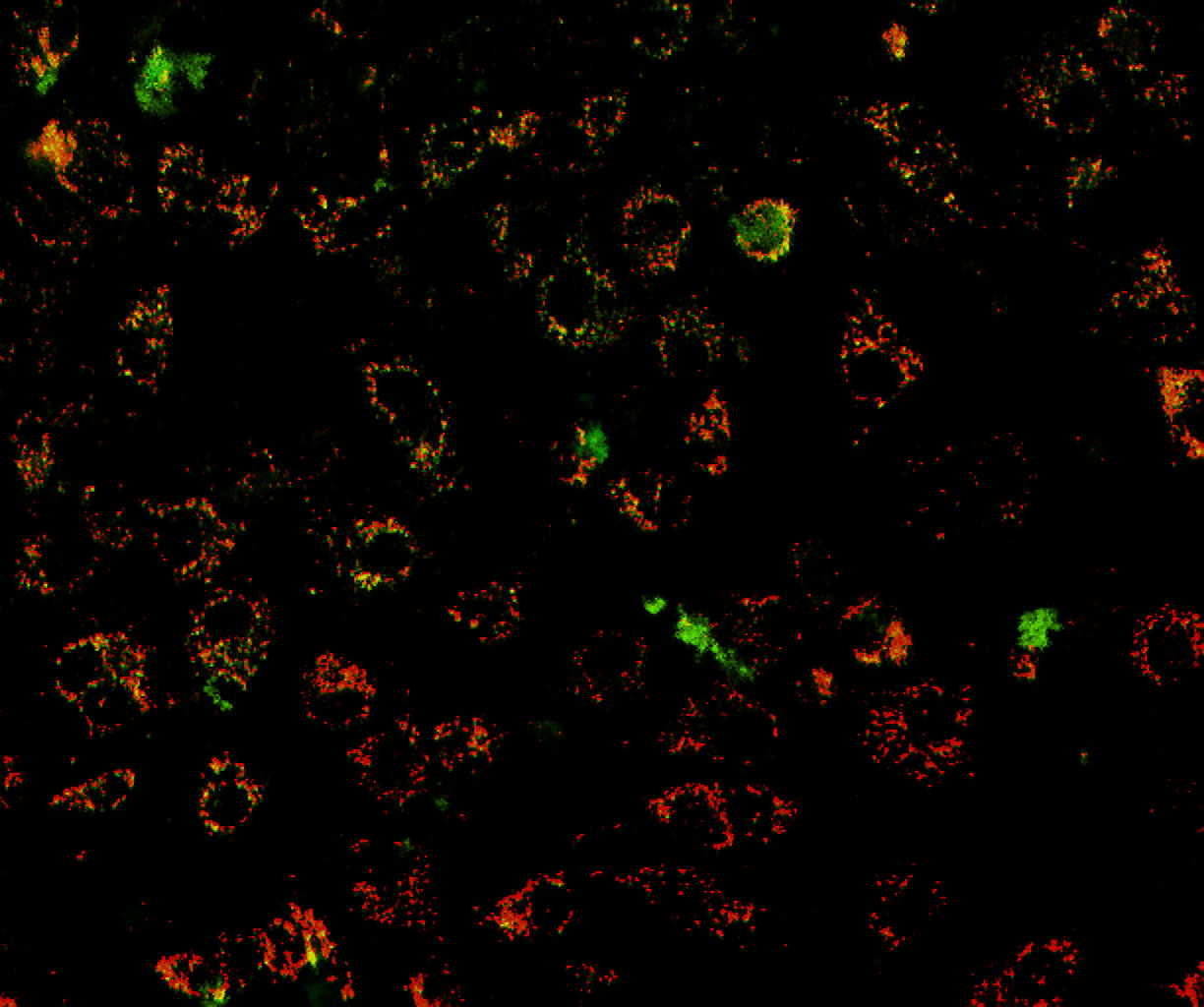

Supplement: Supplementary file 6 [file DataSheet_5.zip › FIG4/NC(+)/2/merge.jpg]

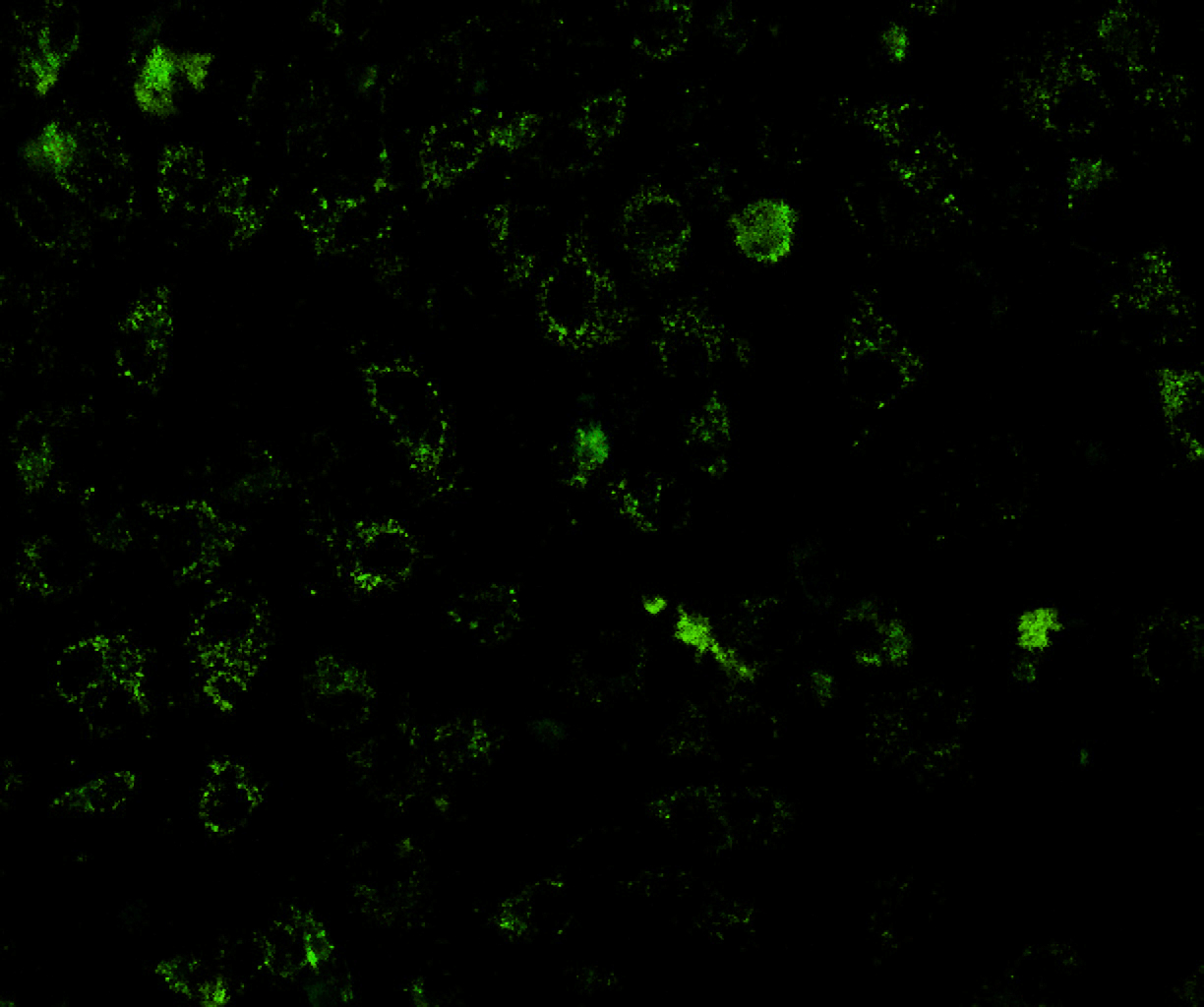

Supplement: Supplementary file 6 [file DataSheet_5.zip › FIG4/NC(+)/2/mono.jpg]

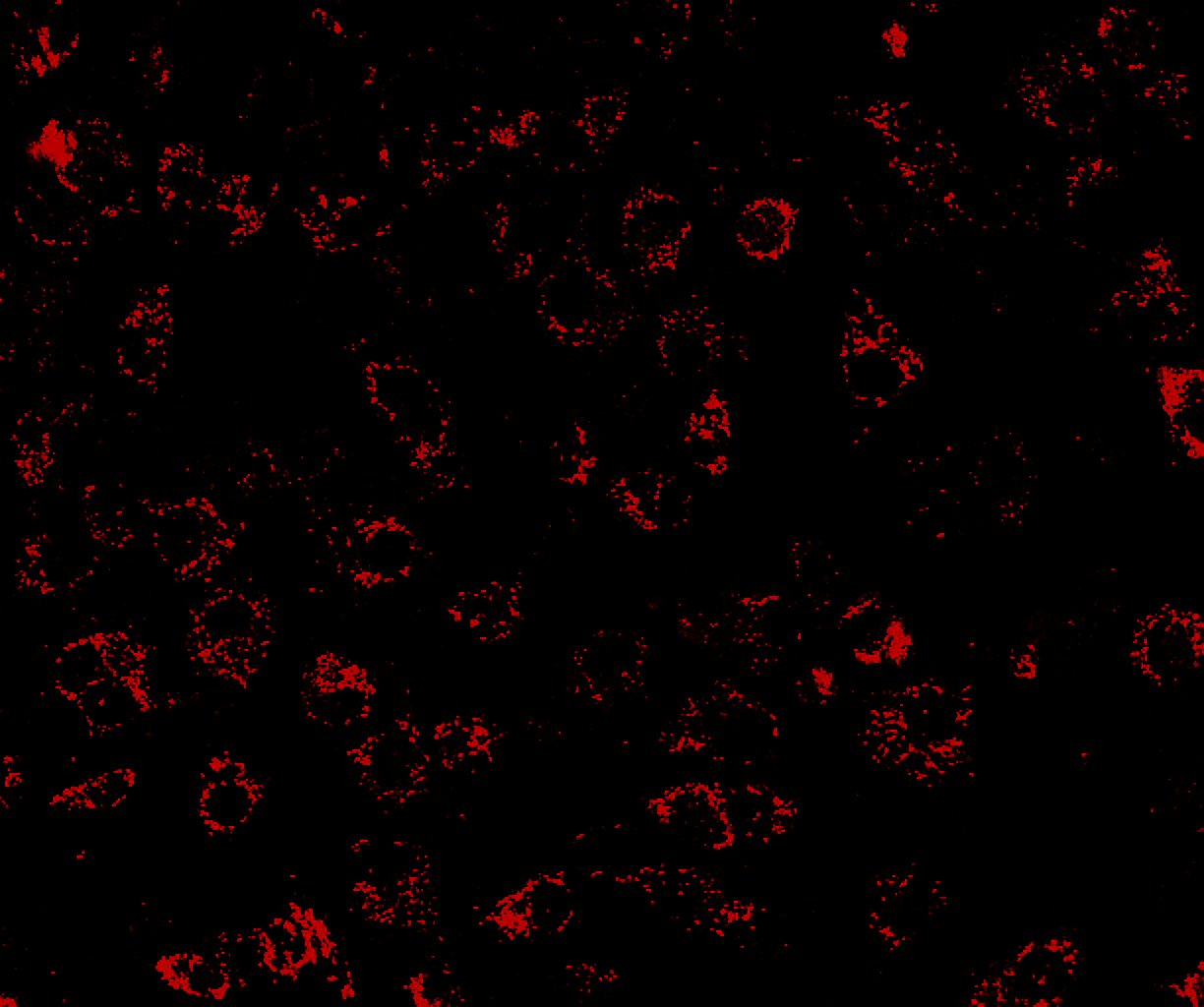

Supplement: Supplementary file 6 [file DataSheet_5.zip › FIG4/NC(+)/2/poly.jpg]

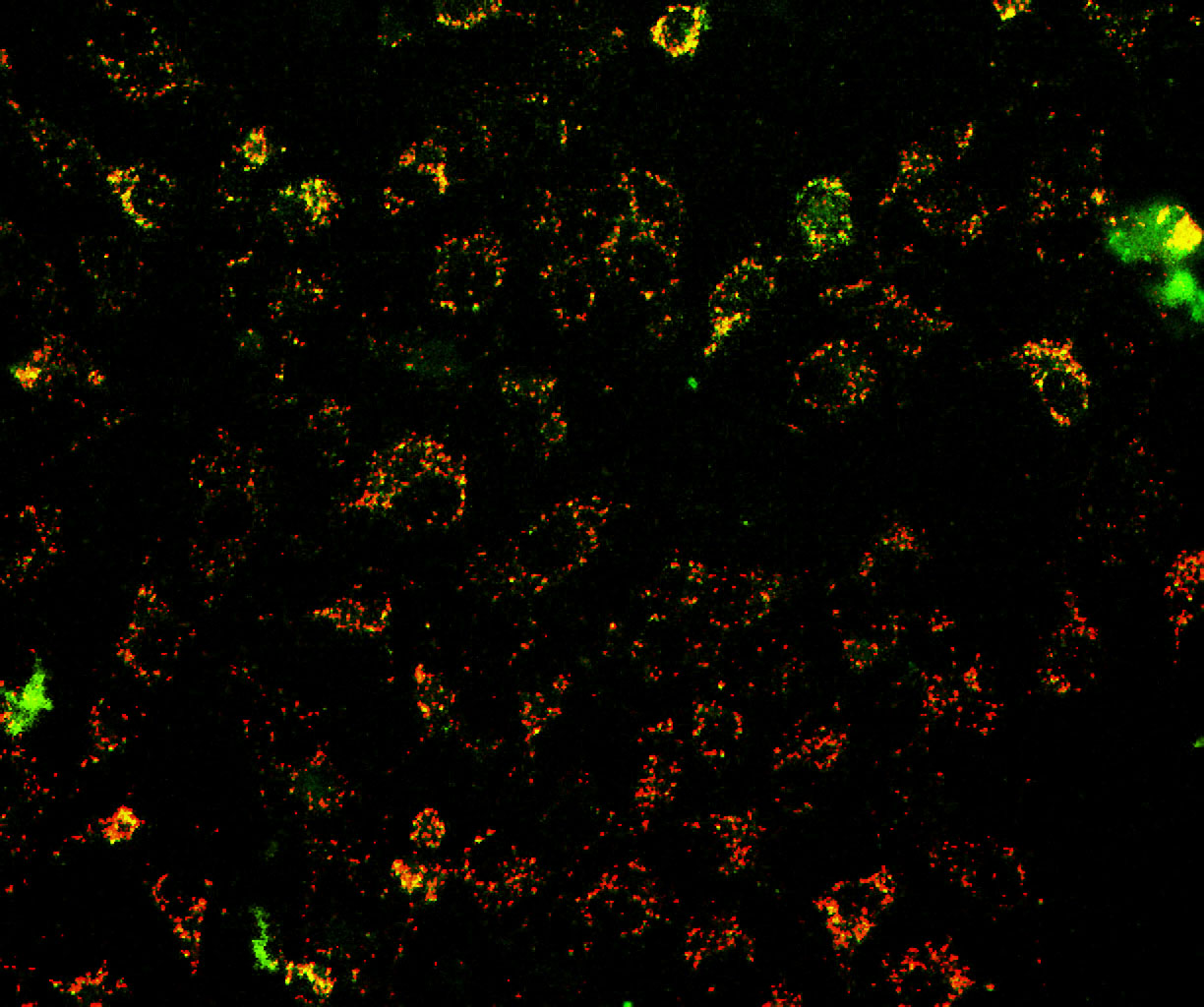

Supplement: Supplementary file 6 [file DataSheet_5.zip › FIG4/NC(+)/3/merge.jpg]

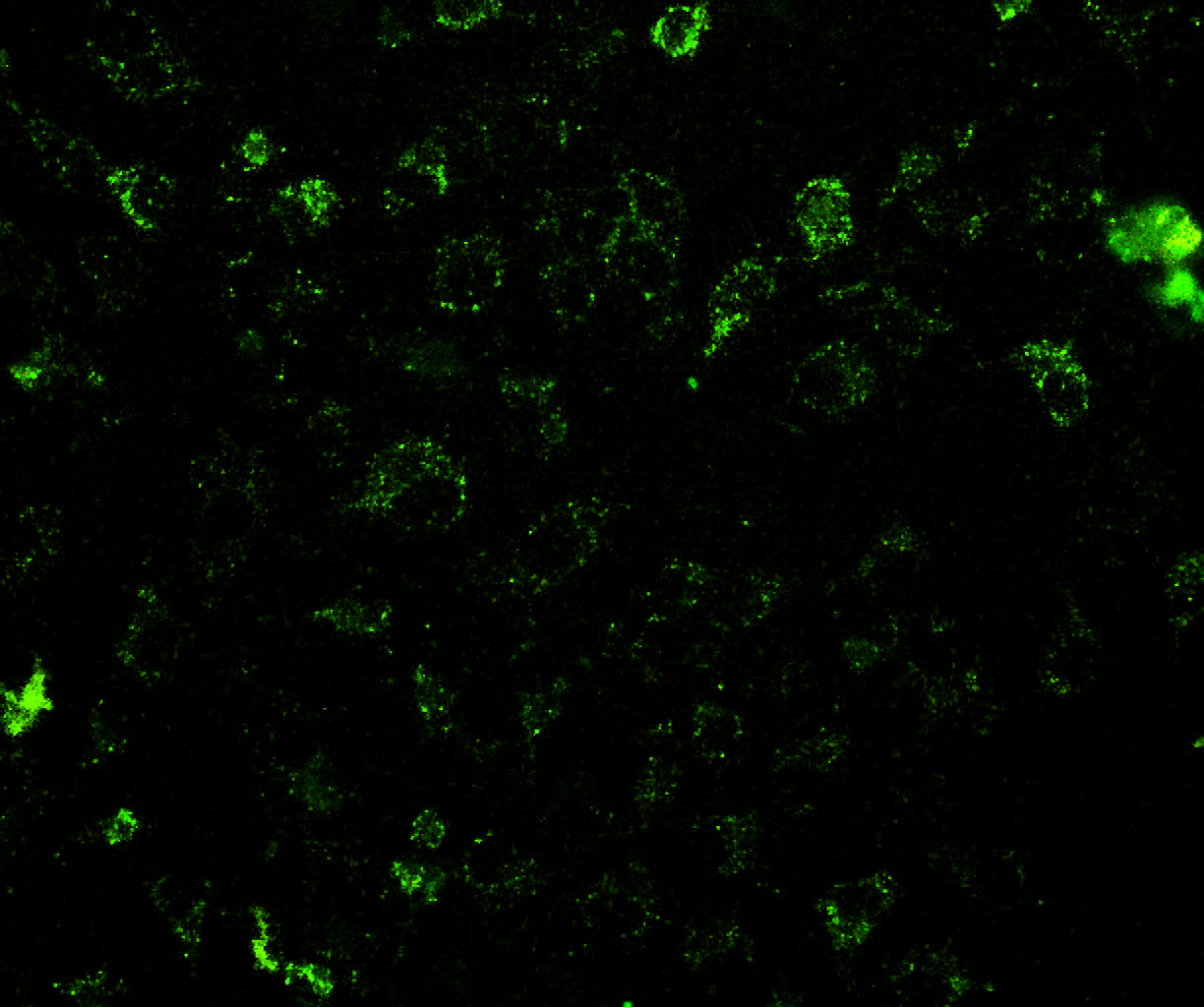

Supplement: Supplementary file 6 [file DataSheet_5.zip › FIG4/NC(+)/3/mono.jpg]

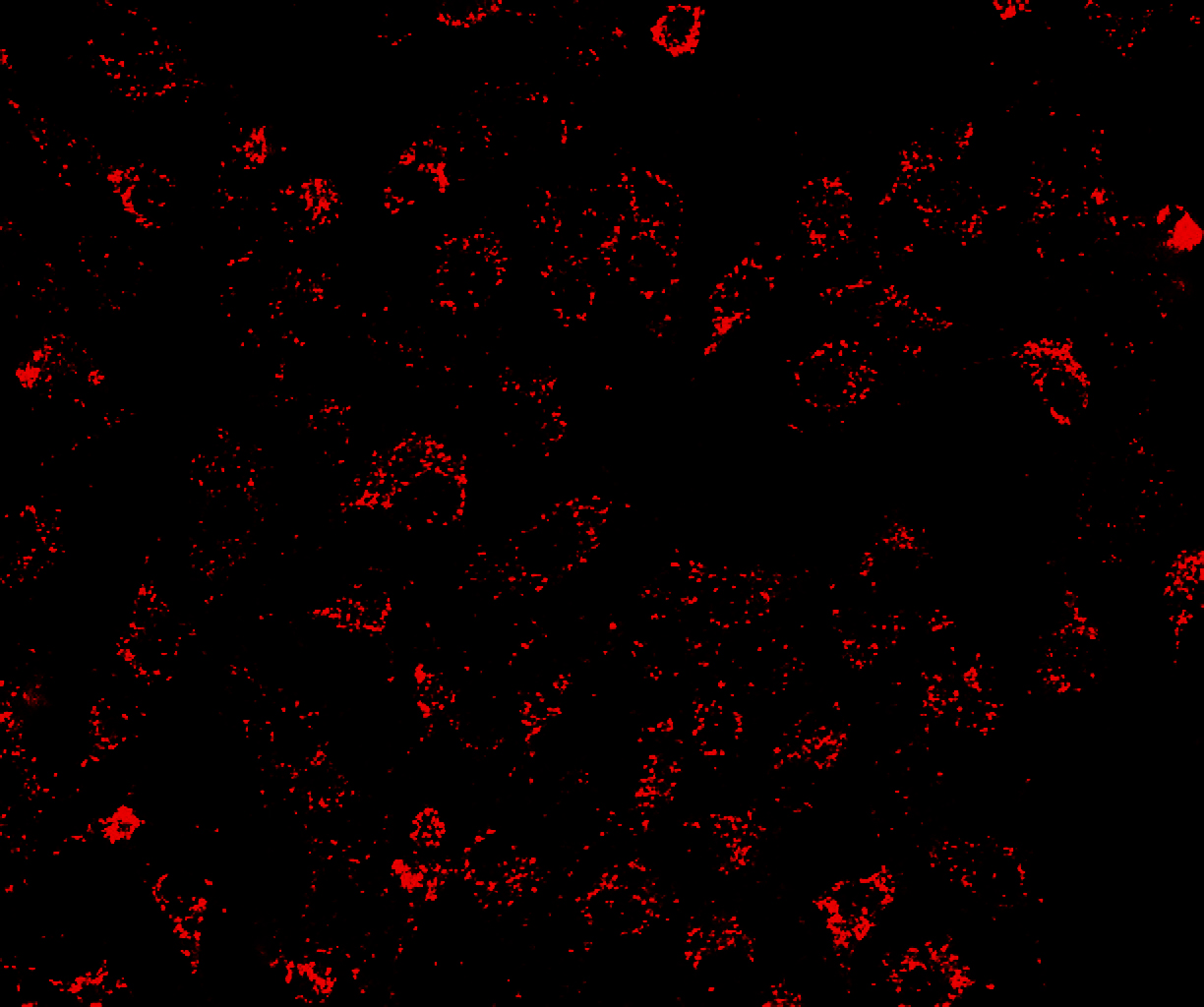

Supplement: Supplementary file 6 [file DataSheet_5.zip › FIG4/NC(+)/3/poly.jpg]

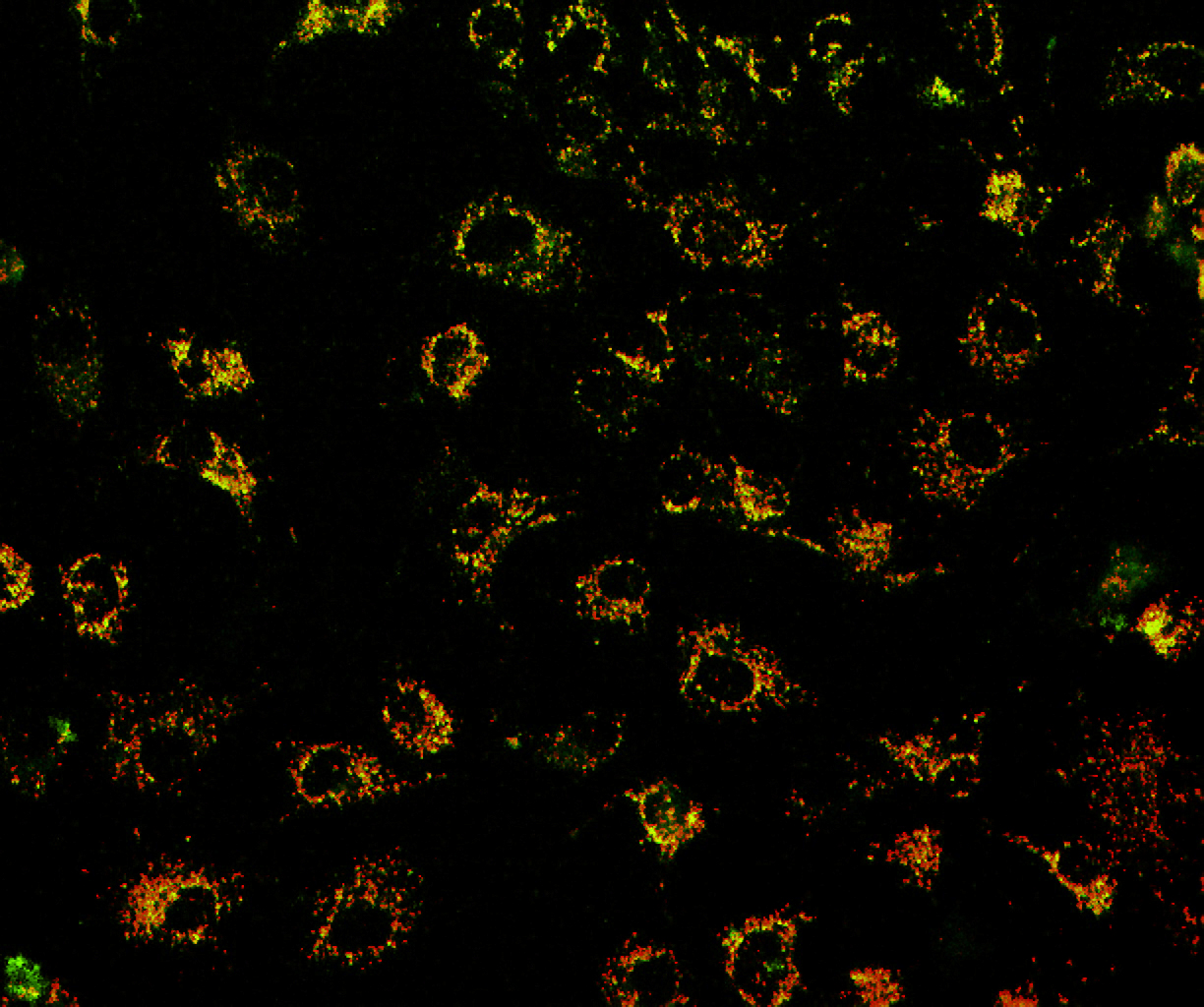

Supplement: Supplementary file 7 [file DataSheet_6.zip › FIG5/Control/1/merge.jpg]

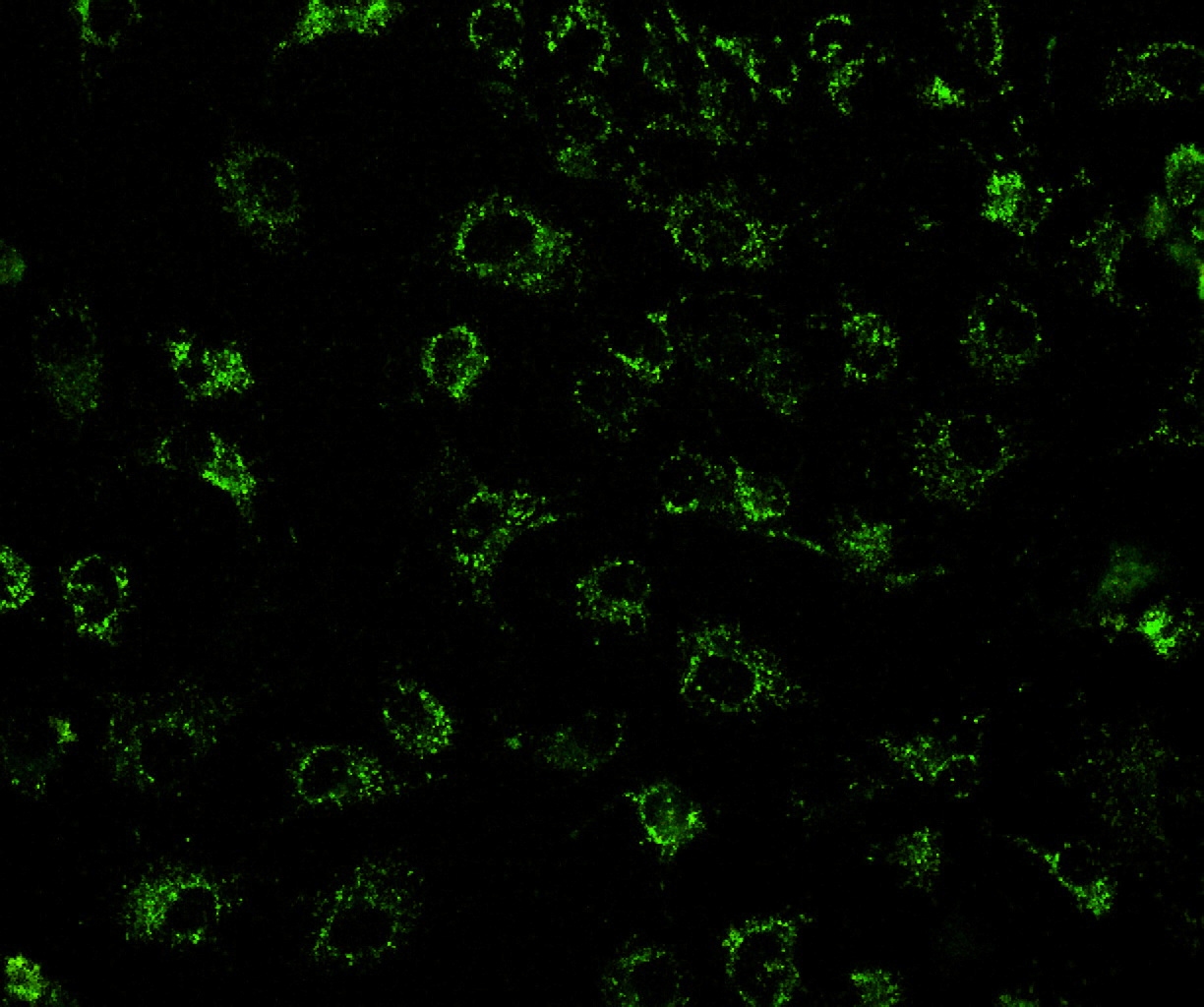

Supplement: Supplementary file 7 [file DataSheet_6.zip › FIG5/Control/1/mono.jpg]

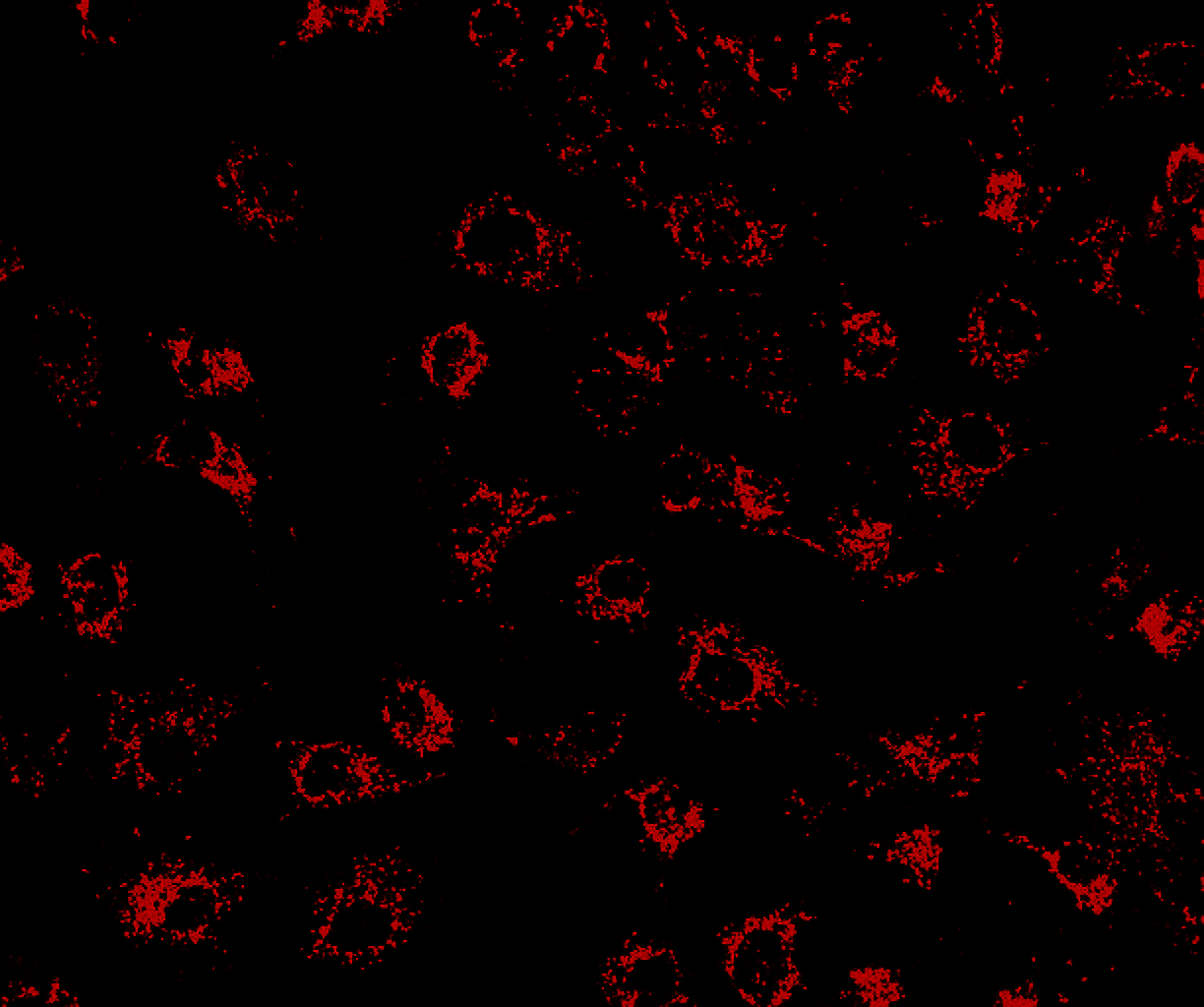

Supplement: Supplementary file 7 [file DataSheet_6.zip › FIG5/Control/1/poly.jpg]

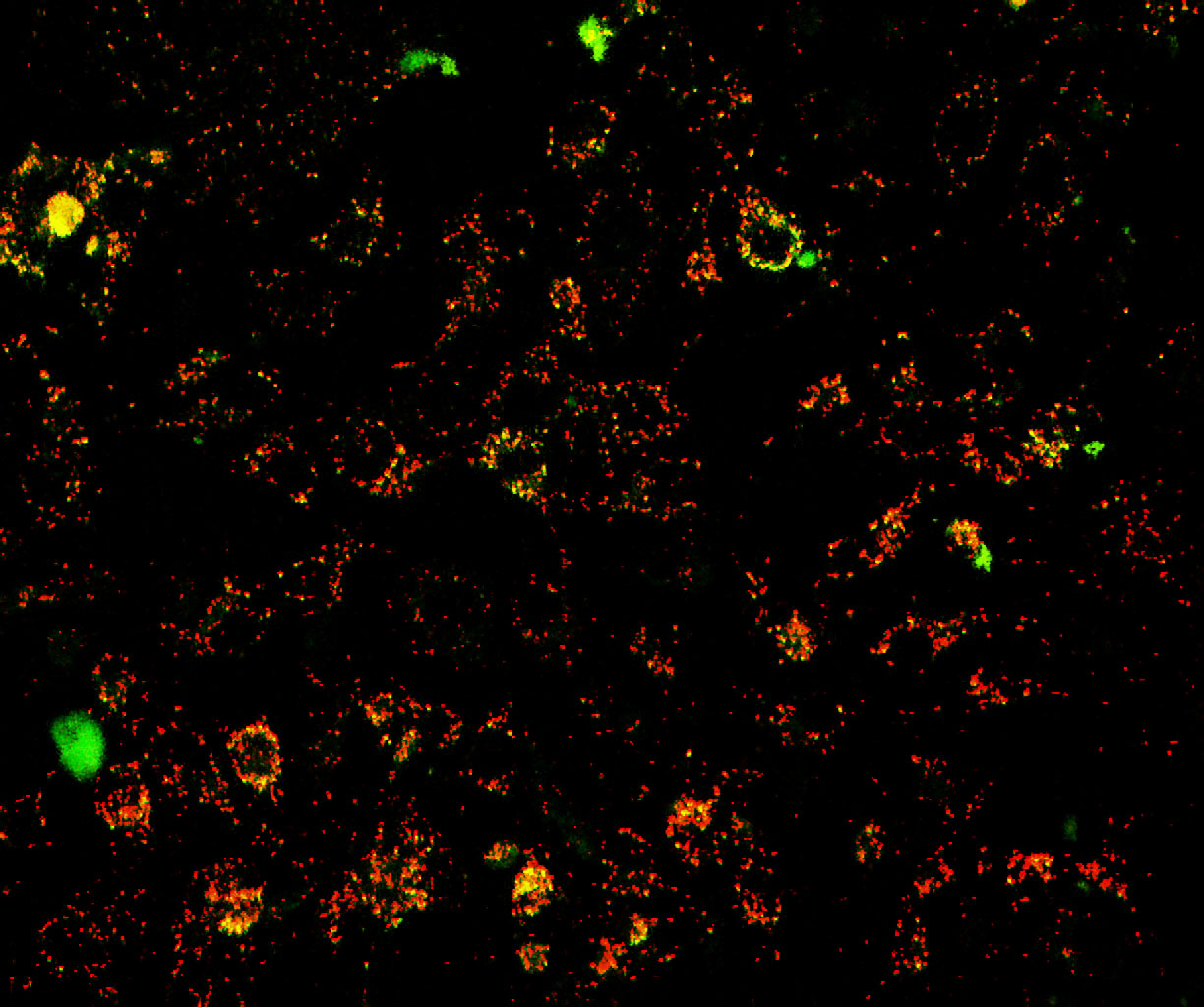

Supplement: Supplementary file 7 [file DataSheet_6.zip › FIG5/Control/2/merge.jpg]

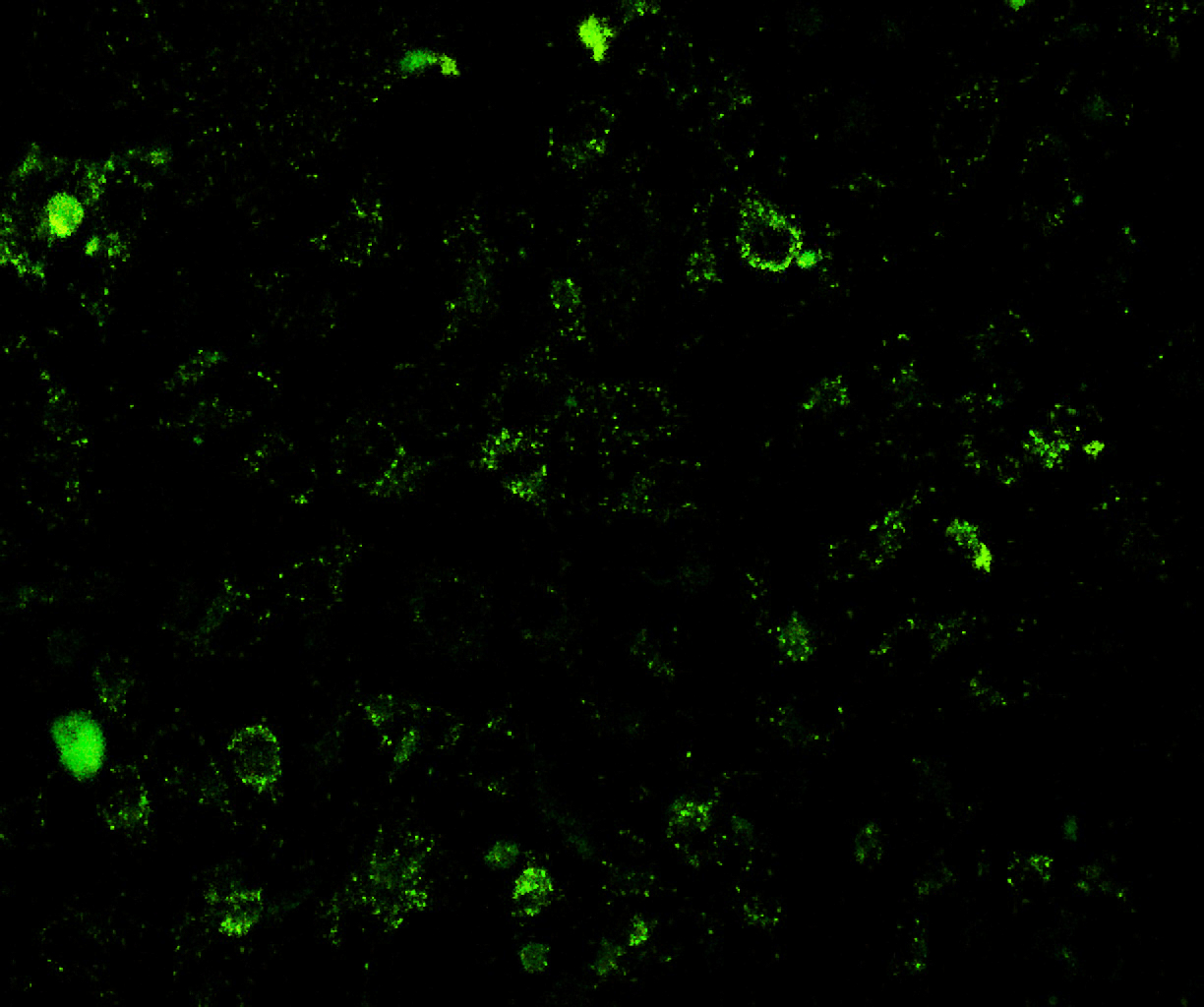

Supplement: Supplementary file 7 [file DataSheet_6.zip › FIG5/Control/2/mono.jpg]

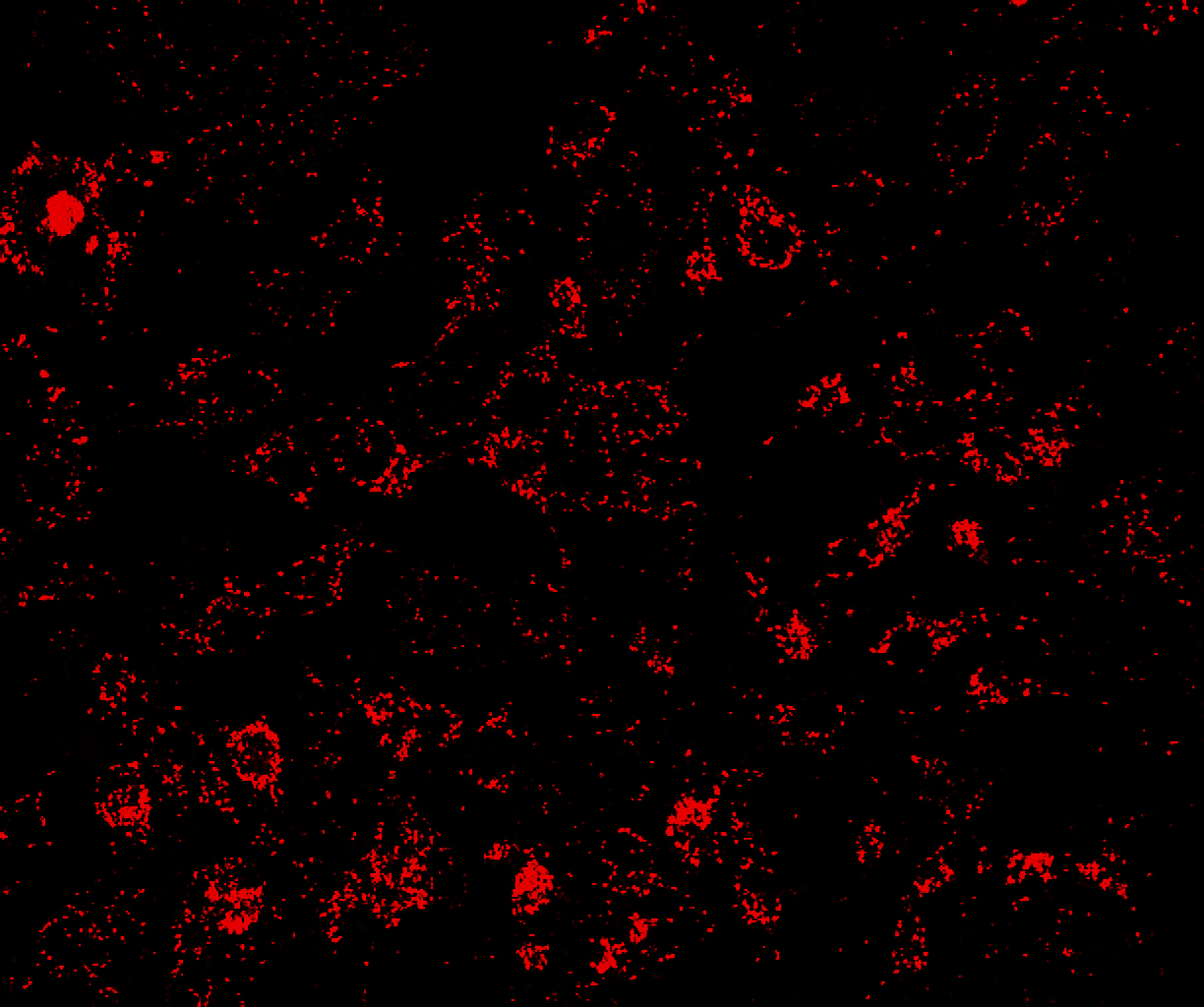

Supplement: Supplementary file 7 [file DataSheet_6.zip › FIG5/Control/2/poly.jpg]

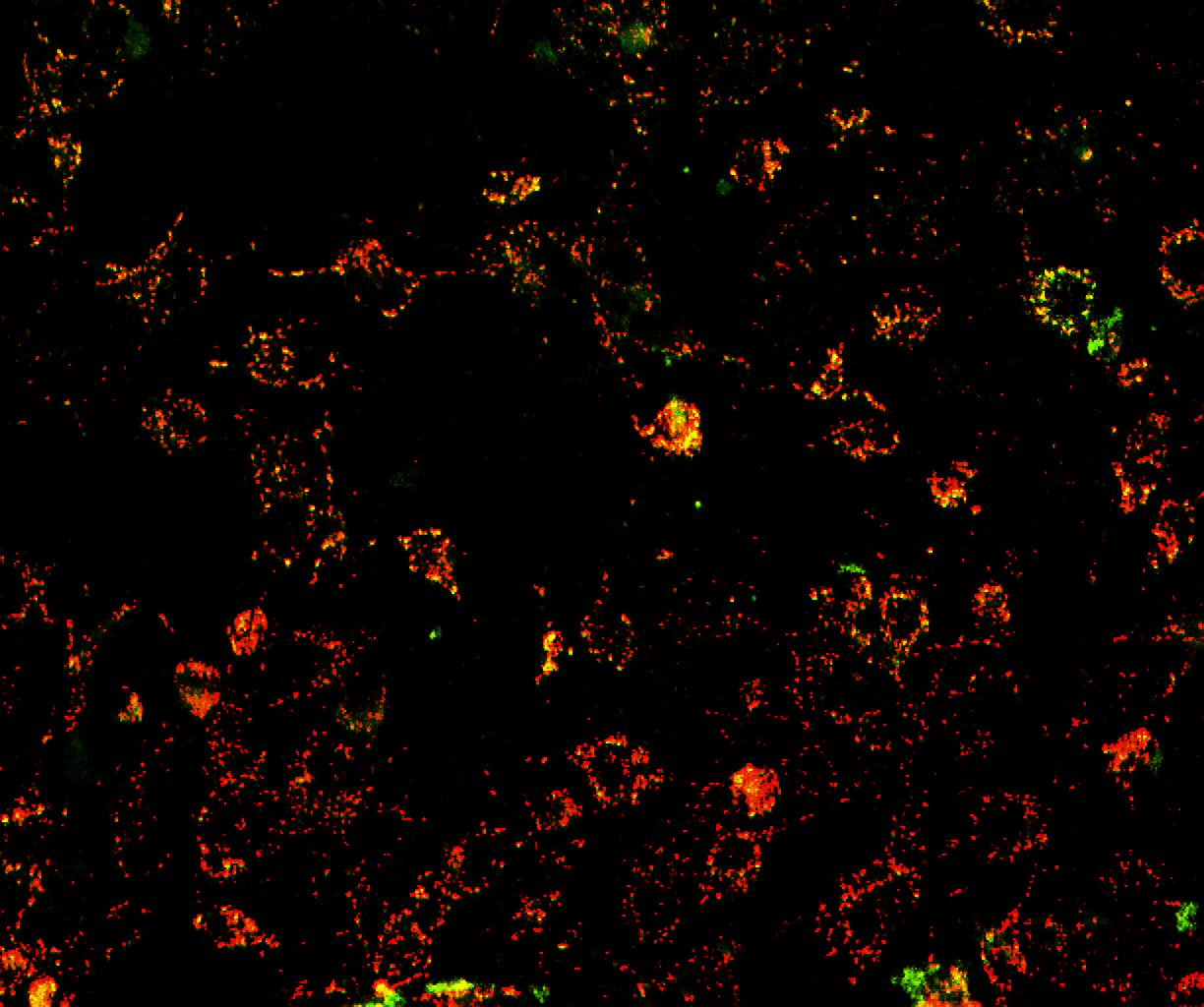

Supplement: Supplementary file 7 [file DataSheet_6.zip › FIG5/Control/3/merge.jpg]

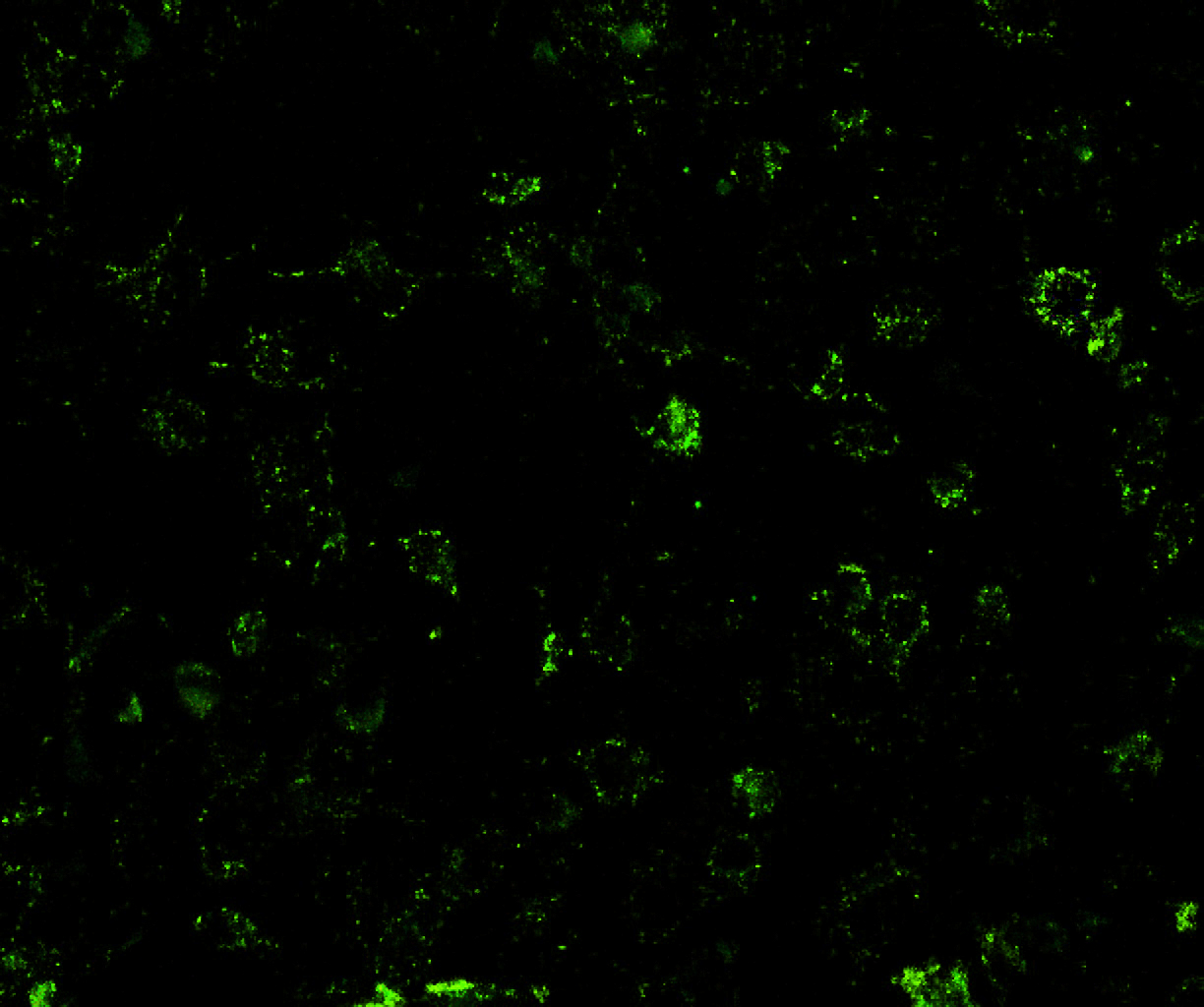

Supplement: Supplementary file 7 [file DataSheet_6.zip › FIG5/Control/3/mono.jpg]

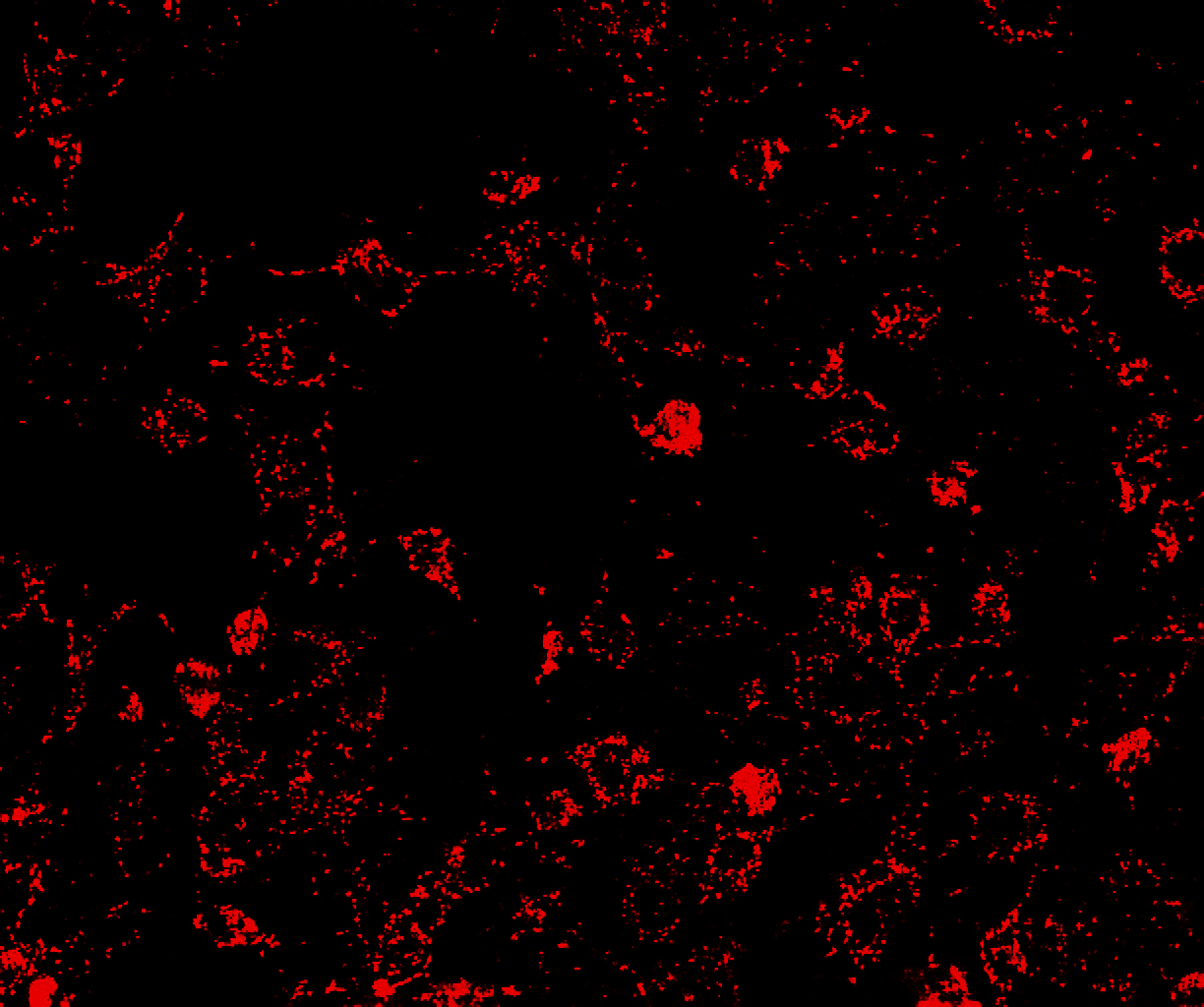

Supplement: Supplementary file 7 [file DataSheet_6.zip › FIG5/Control/3/poly.jpg]

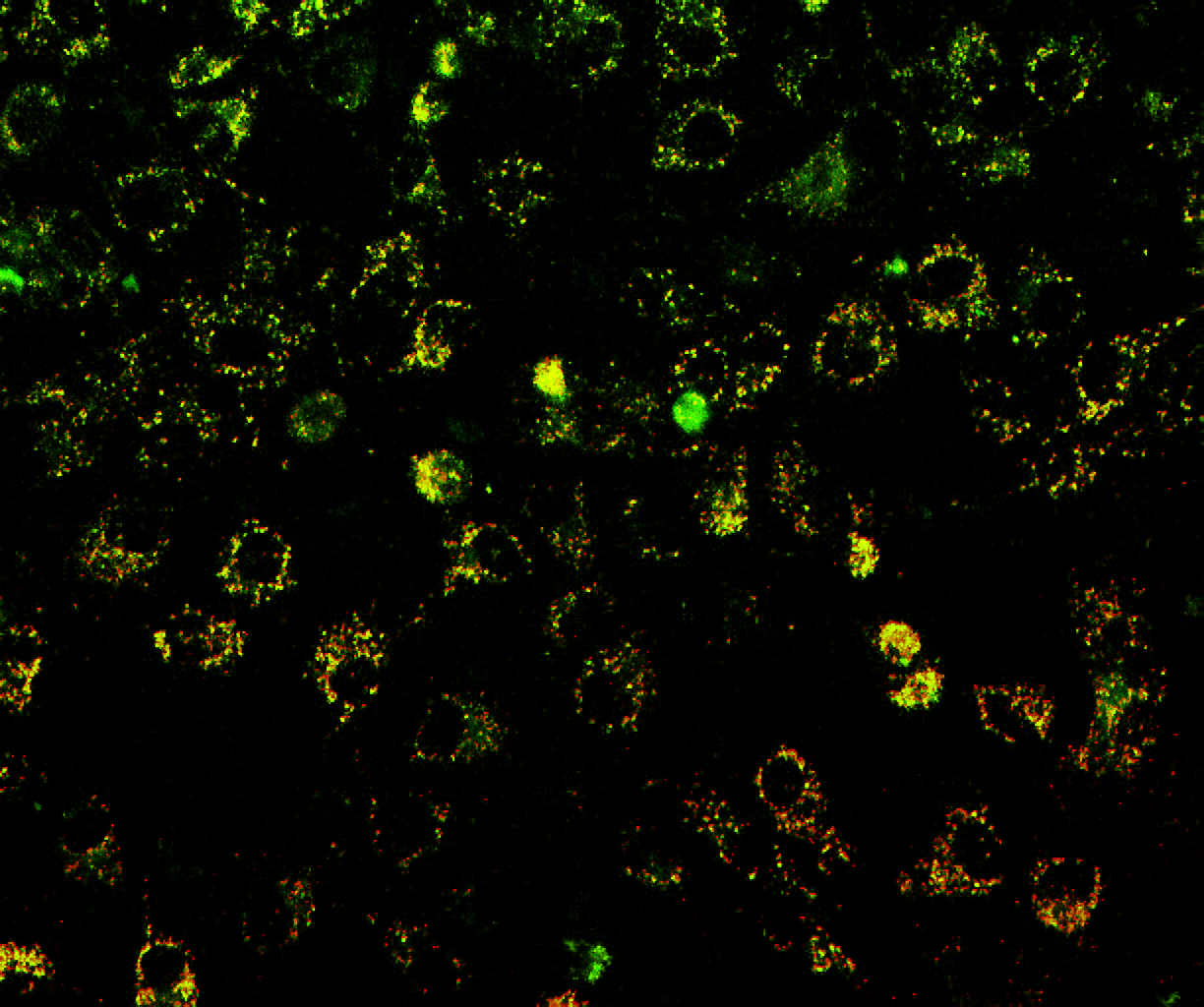

Supplement: Supplementary file 7 [file DataSheet_6.zip › FIG5/miR(+)/1/merge.jpg]

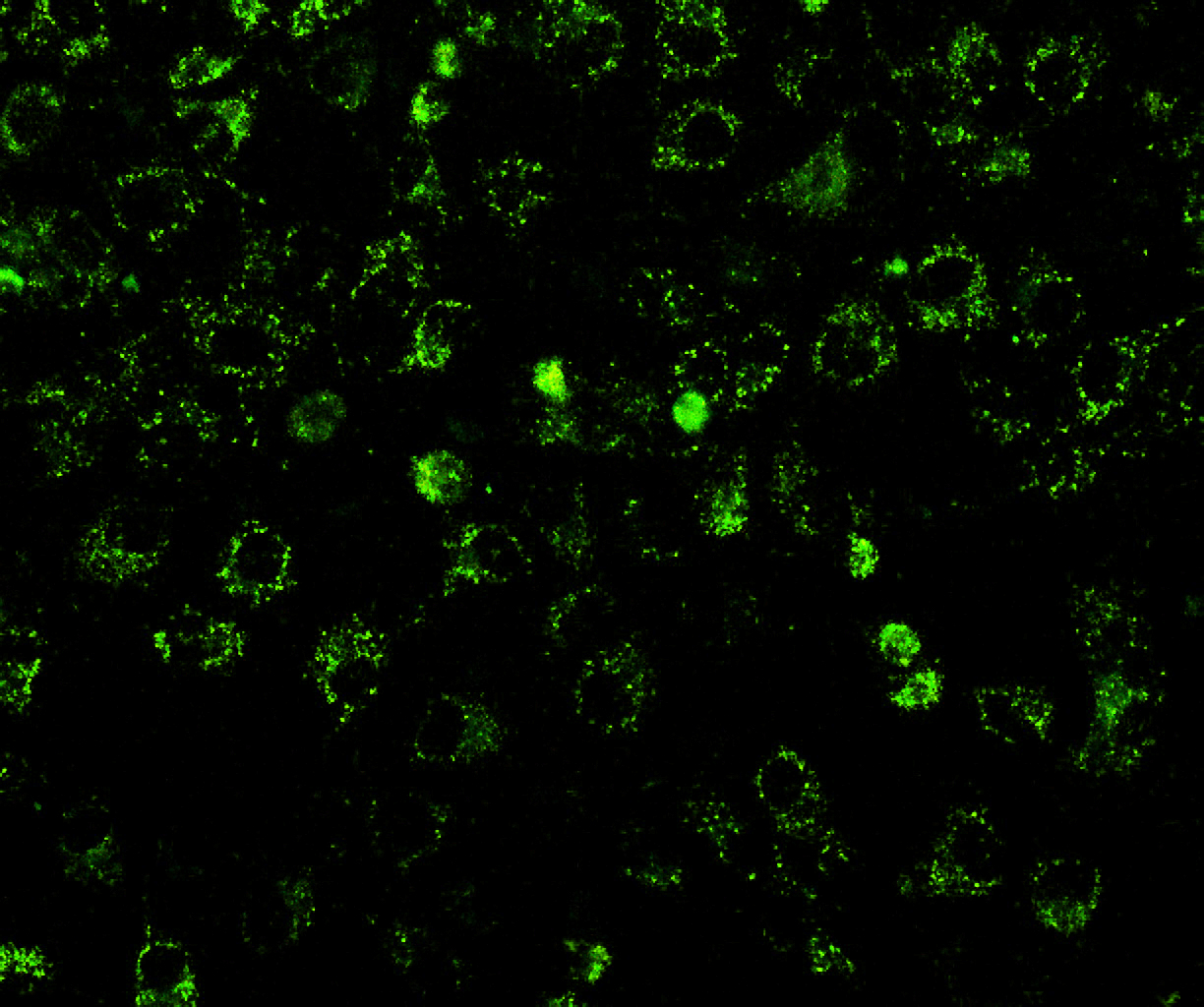

Supplement: Supplementary file 7 [file DataSheet_6.zip › FIG5/miR(+)/1/mono.jpg]

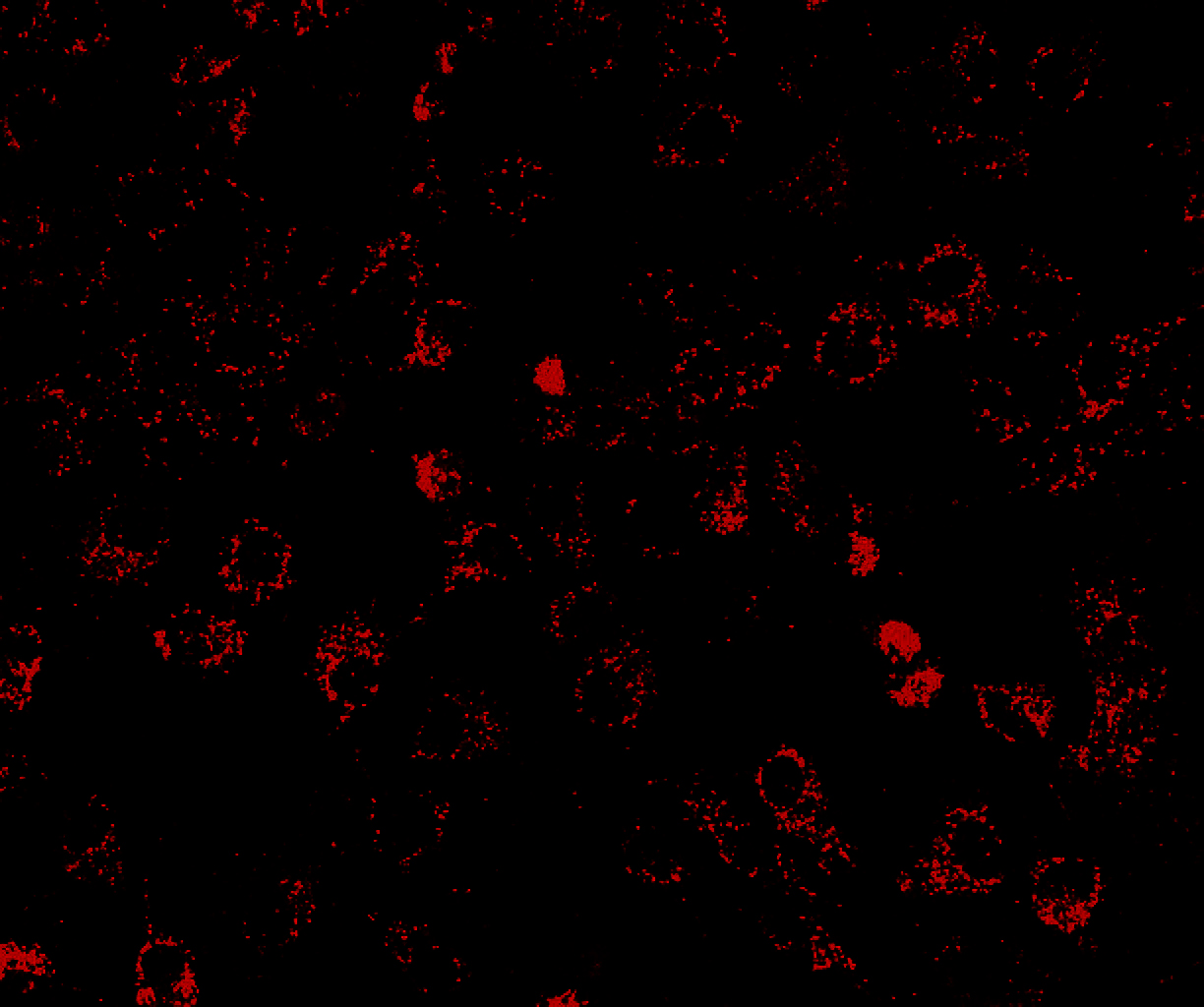

Supplement: Supplementary file 7 [file DataSheet_6.zip › FIG5/miR(+)/1/poly.jpg]

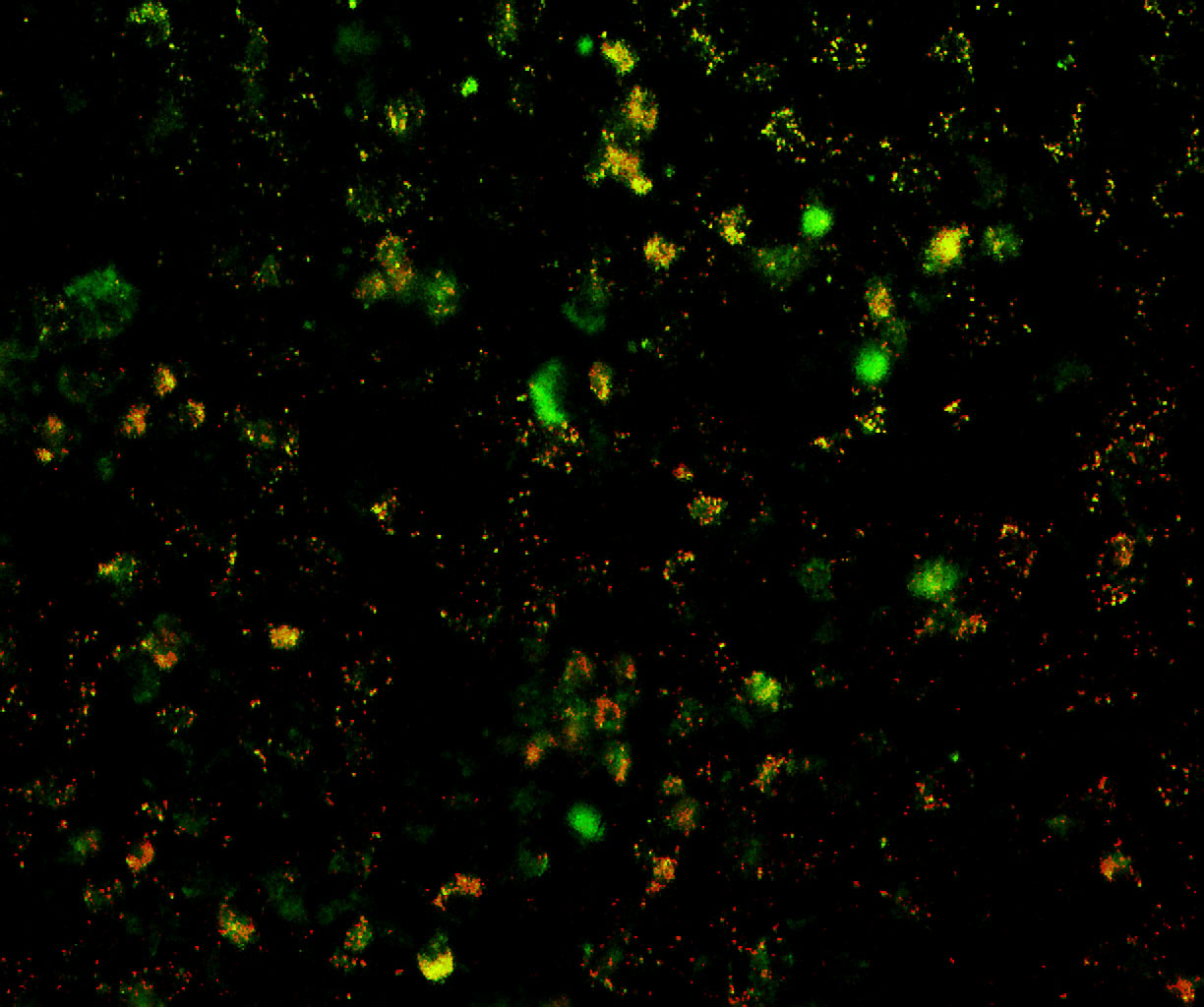

Supplement: Supplementary file 7 [file DataSheet_6.zip › FIG5/miR(+)/2/merge.jpg]

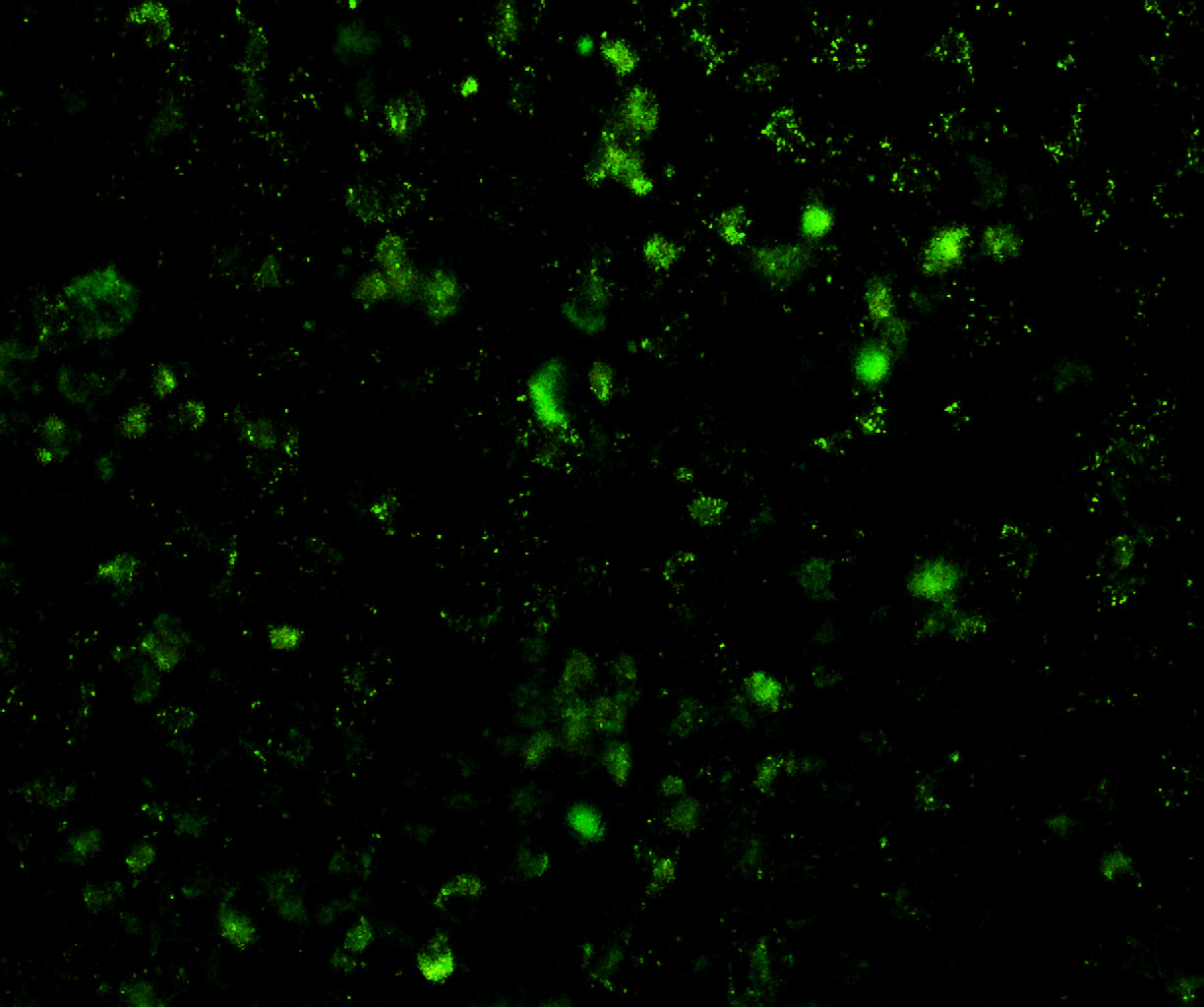

Supplement: Supplementary file 7 [file DataSheet_6.zip › FIG5/miR(+)/2/mono.jpg]

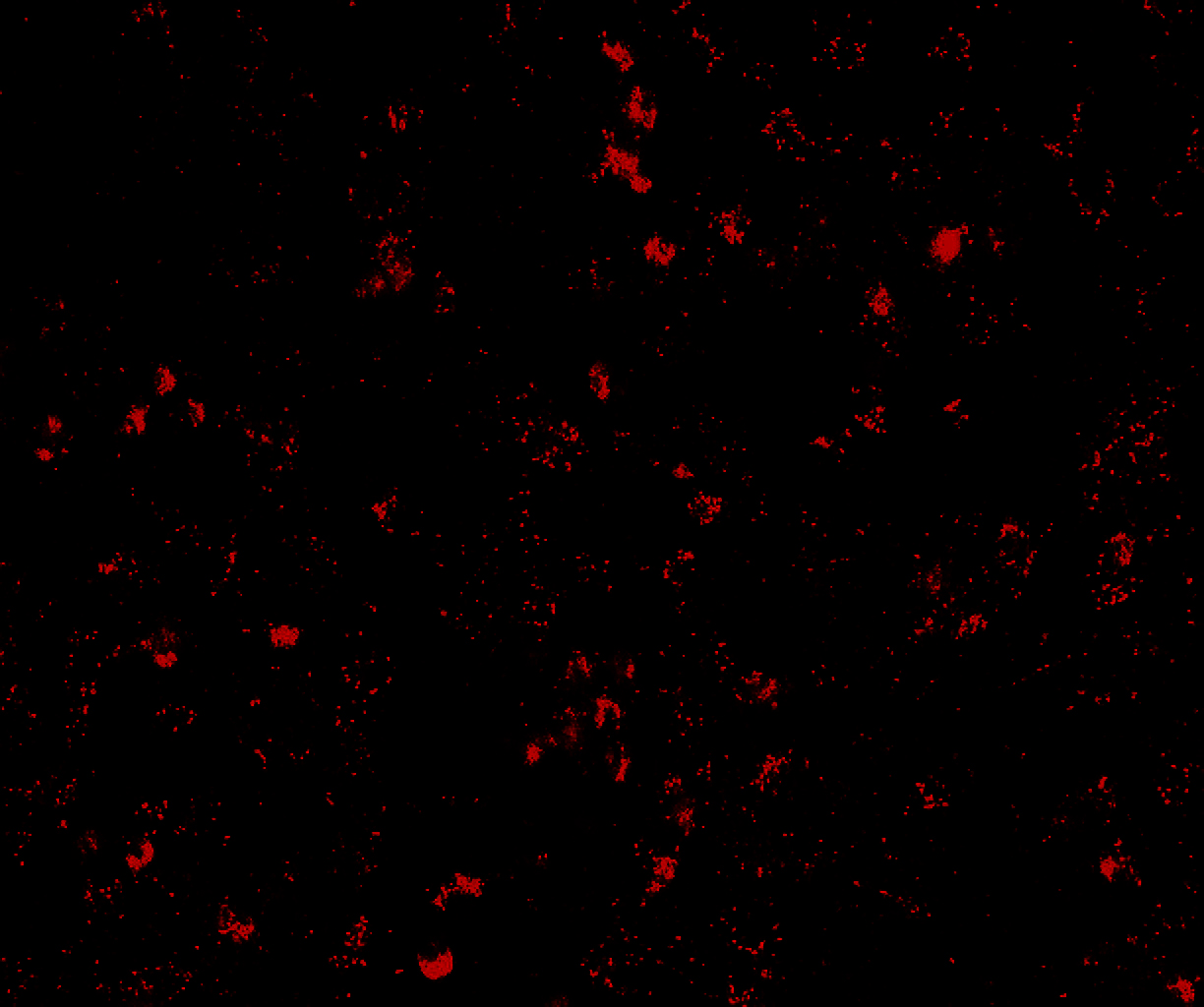

Supplement: Supplementary file 7 [file DataSheet_6.zip › FIG5/miR(+)/2/poly.jpg]

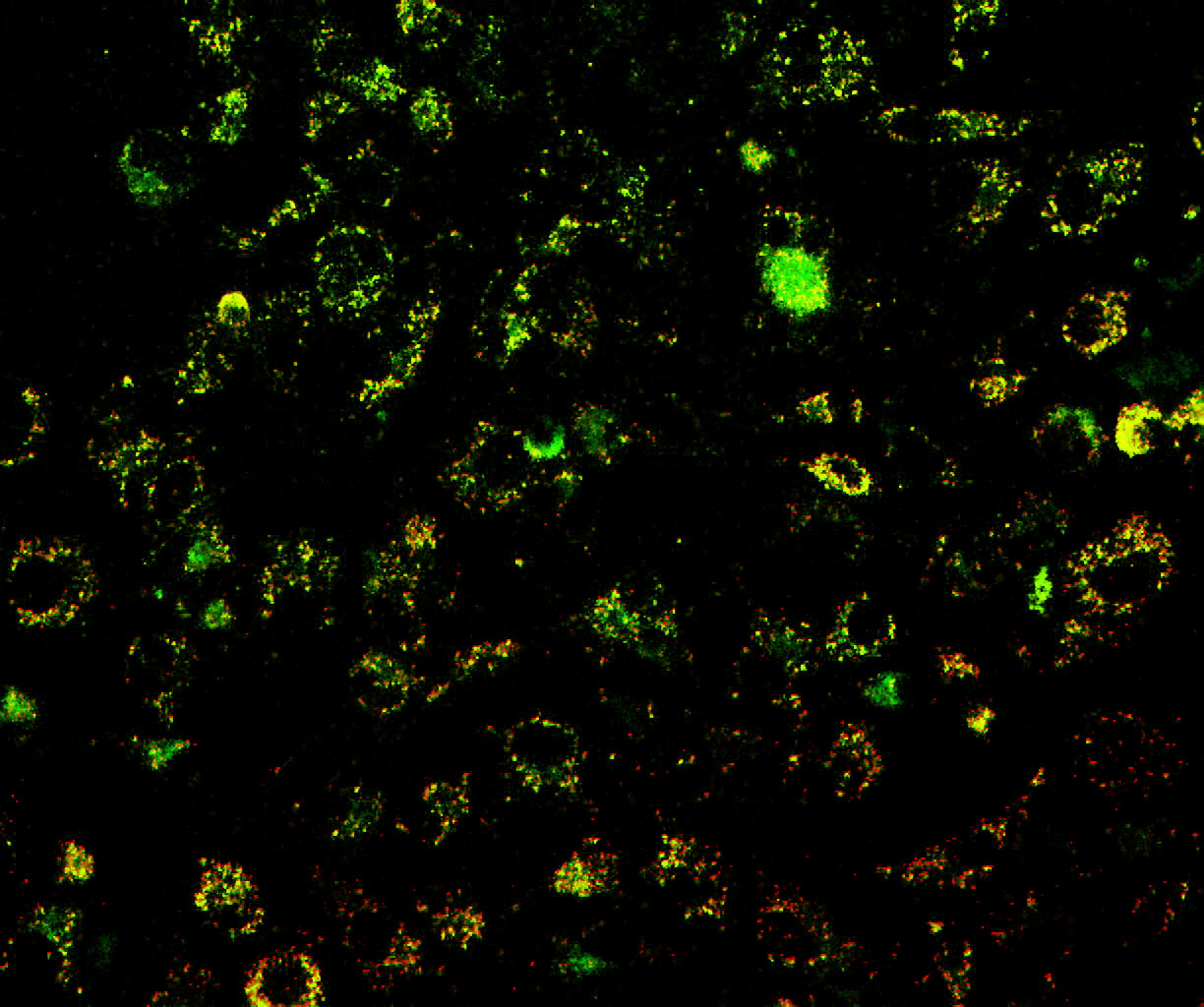

Supplement: Supplementary file 7 [file DataSheet_6.zip › FIG5/miR(+)/3/merge.jpg]

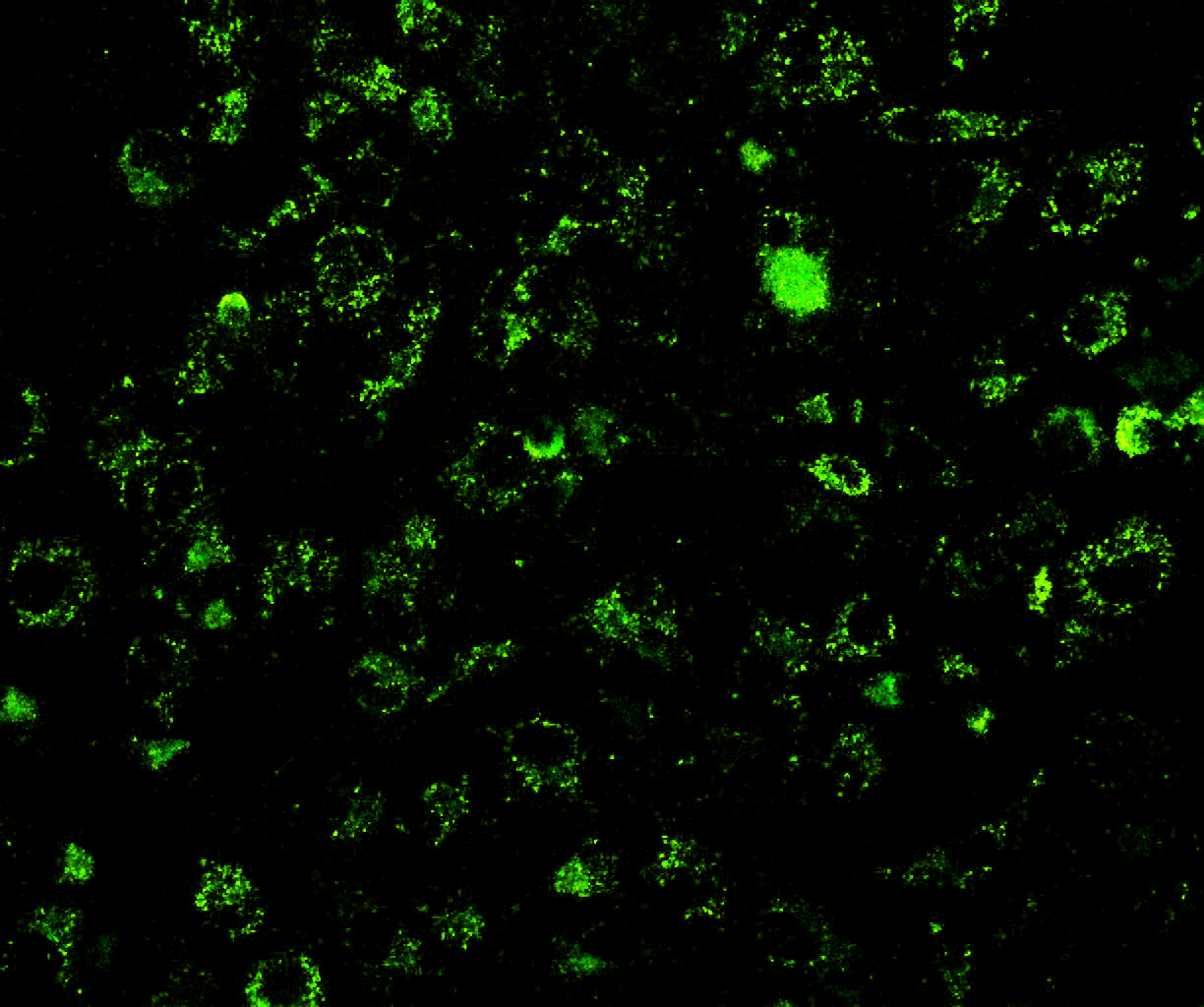

Supplement: Supplementary file 7 [file DataSheet_6.zip › FIG5/miR(+)/3/mono.jpg]

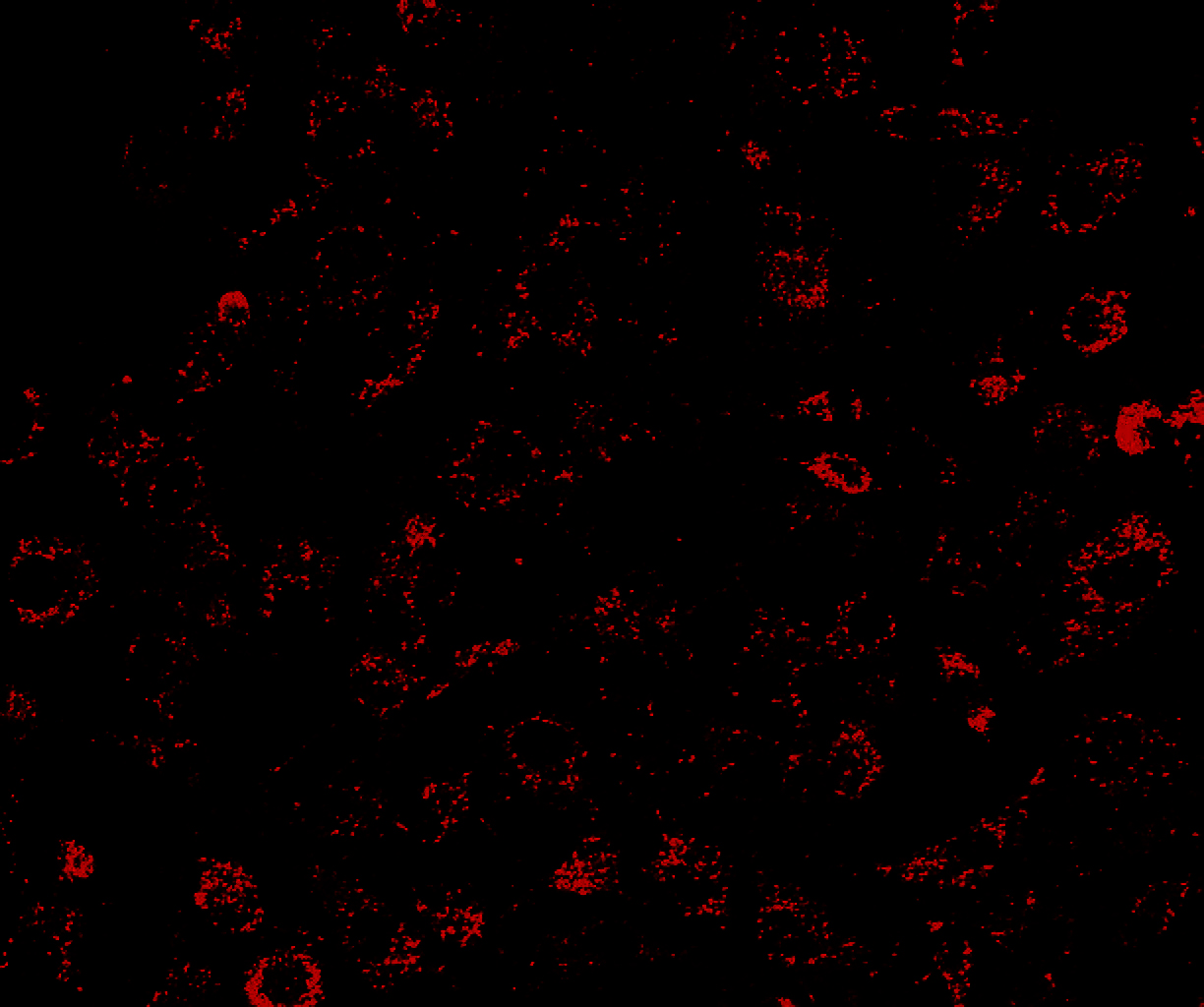

Supplement: Supplementary file 7 [file DataSheet_6.zip › FIG5/miR(+)/3/poly.jpg]

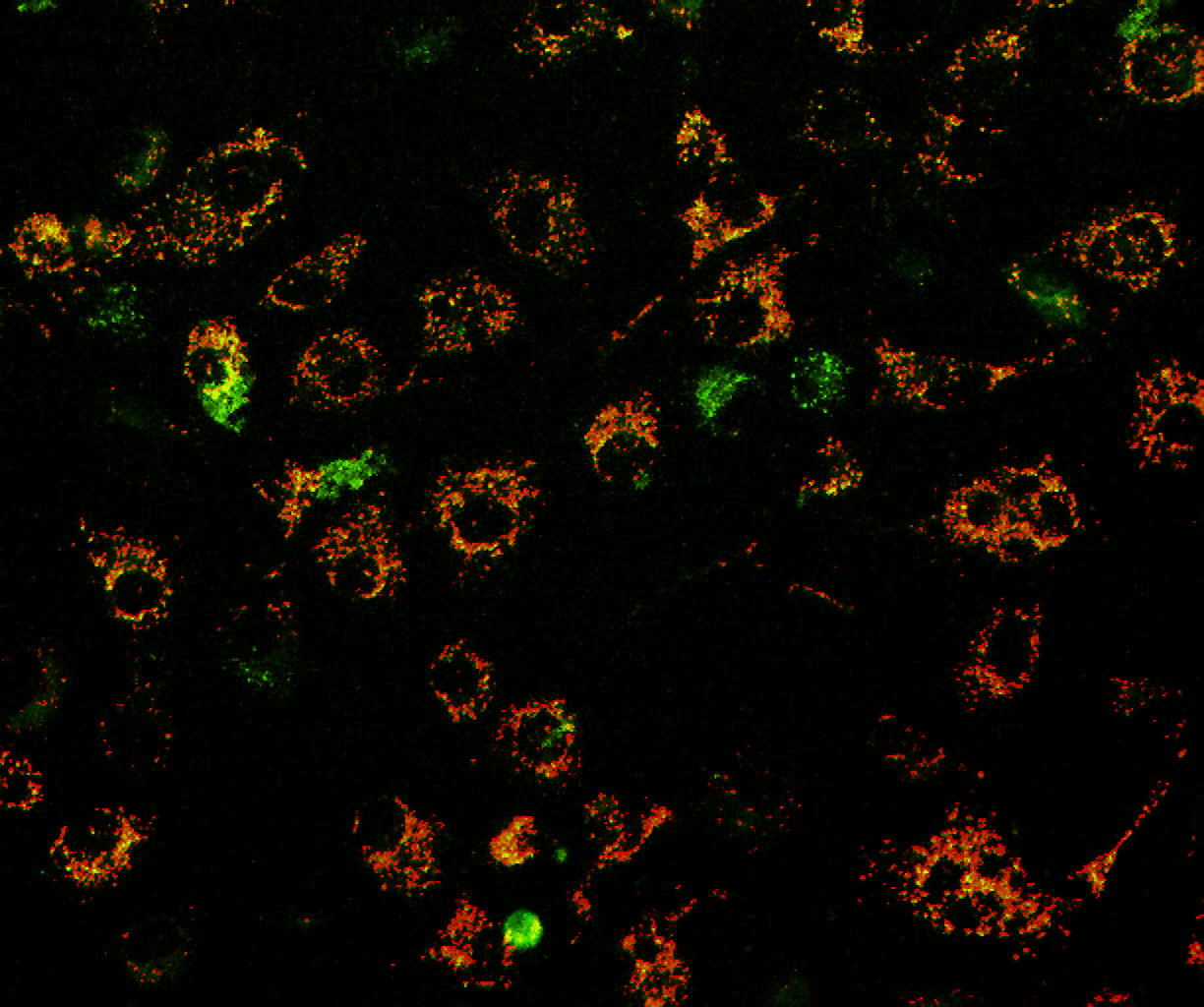

Supplement: Supplementary file 7 [file DataSheet_6.zip › FIG5/miR(+)+SLC7A11(+)/1/merge.jpg]

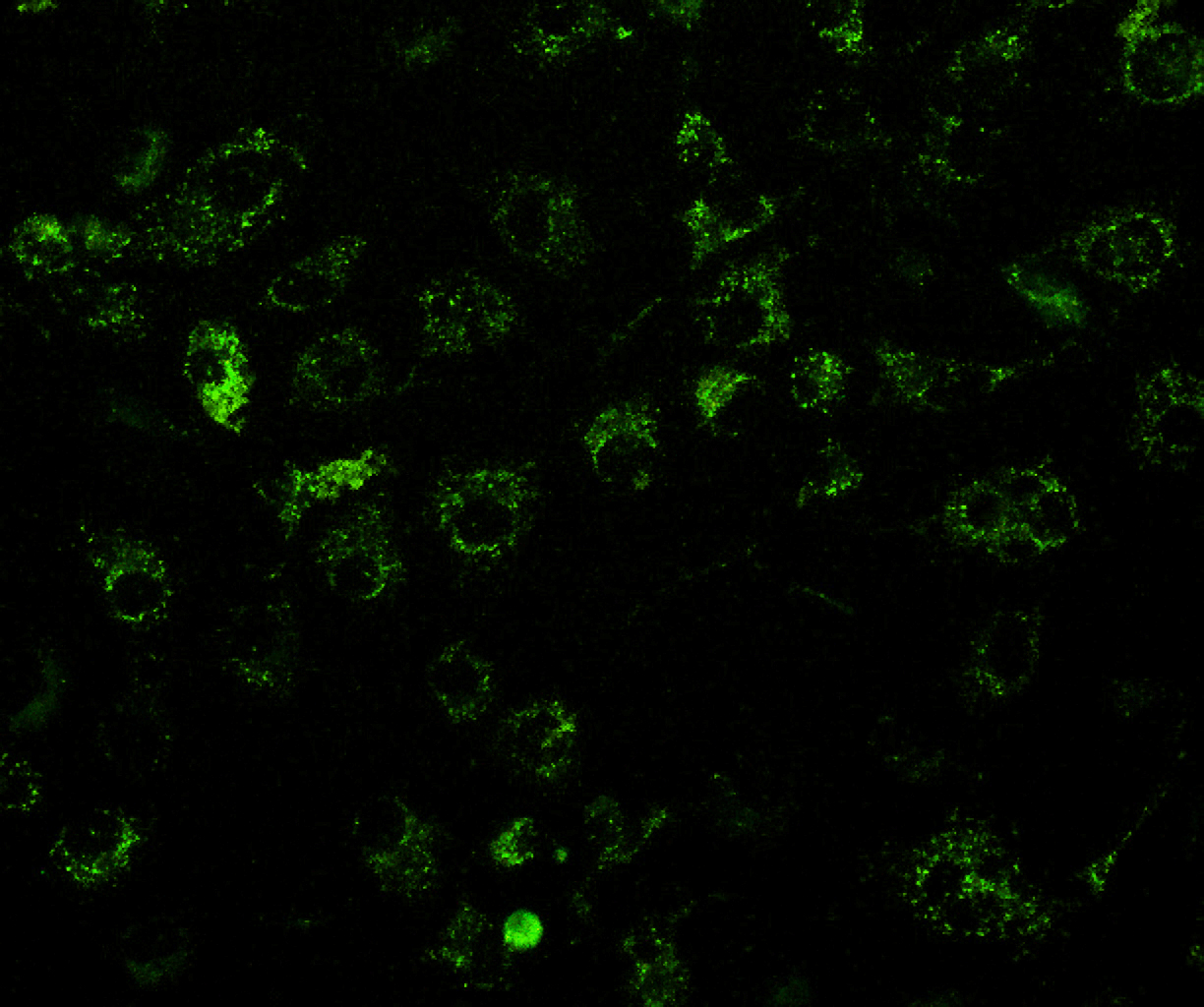

Supplement: Supplementary file 7 [file DataSheet_6.zip › FIG5/miR(+)+SLC7A11(+)/1/mono.jpg]

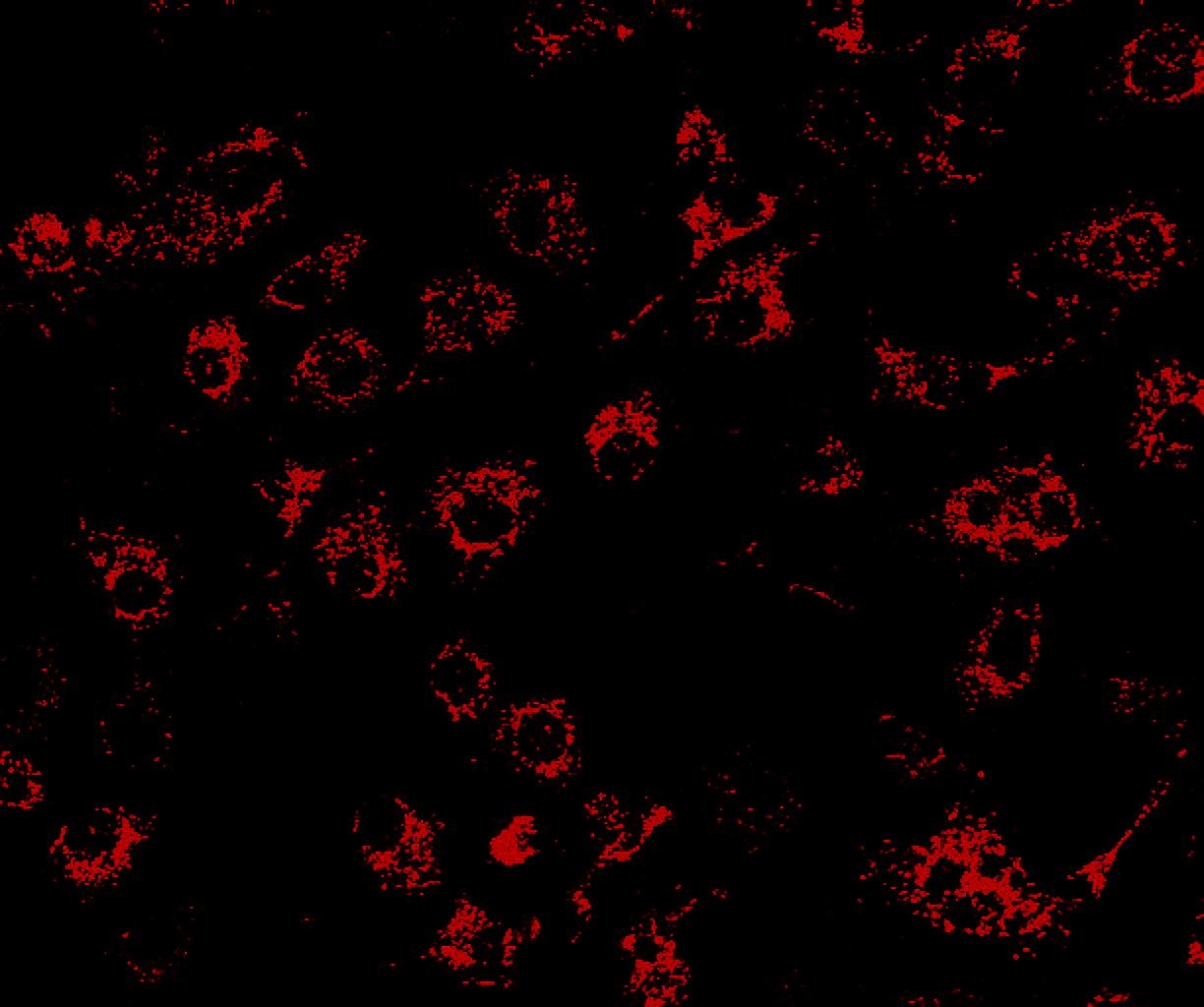

Supplement: Supplementary file 7 [file DataSheet_6.zip › FIG5/miR(+)+SLC7A11(+)/1/poly.jpg]

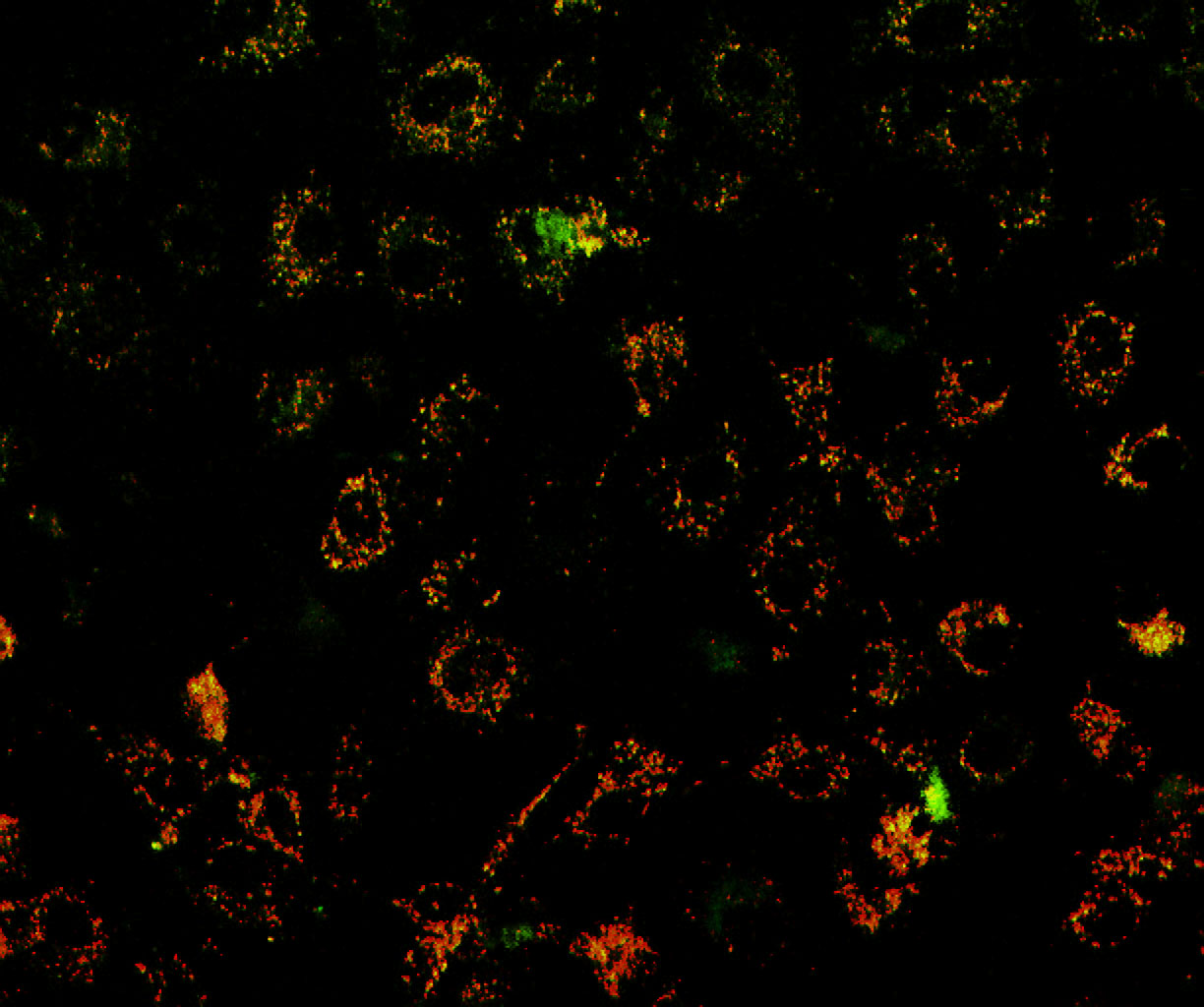

Supplement: Supplementary file 7 [file DataSheet_6.zip › FIG5/miR(+)+SLC7A11(+)/2/merge.jpg]

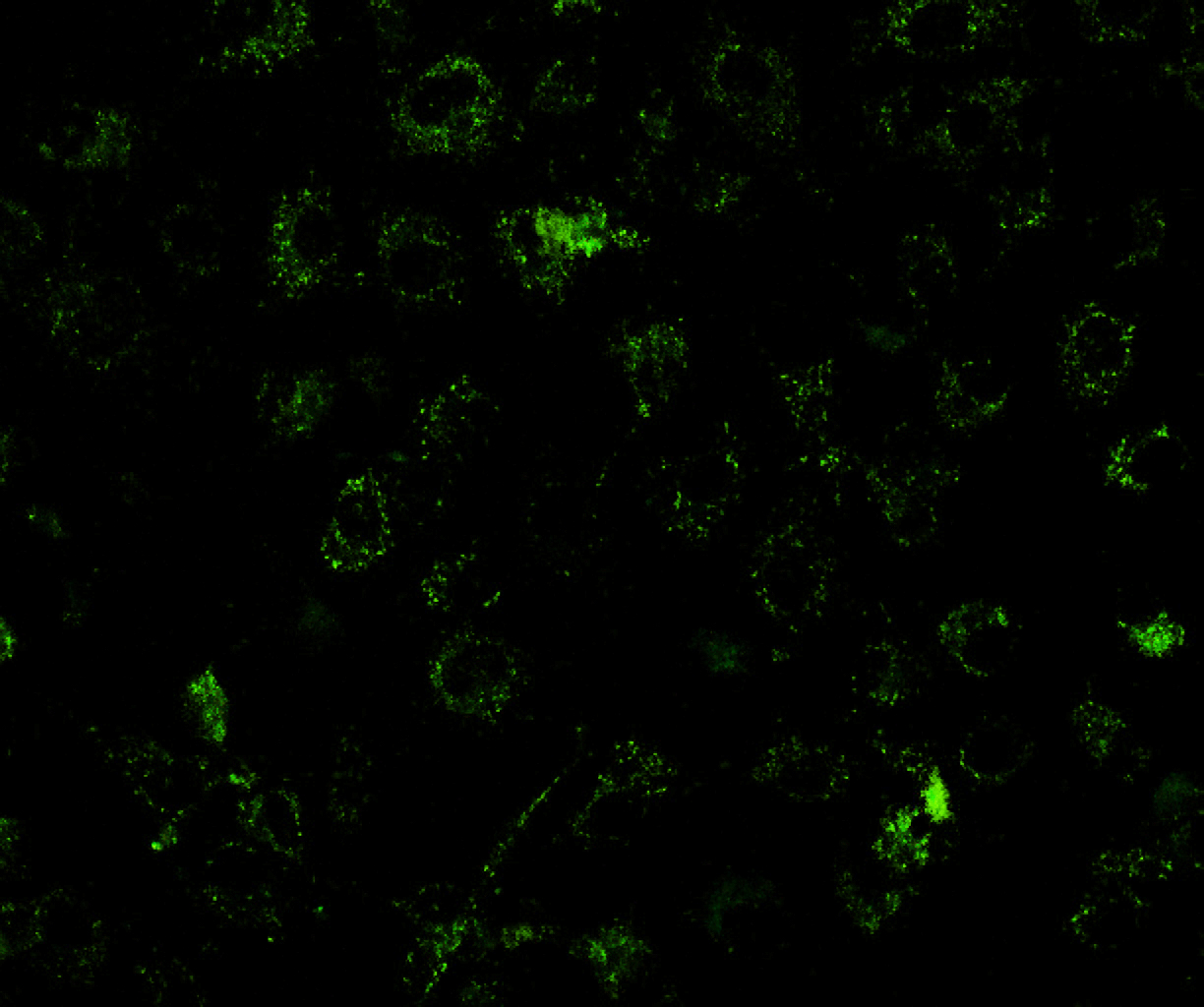

Supplement: Supplementary file 7 [file DataSheet_6.zip › FIG5/miR(+)+SLC7A11(+)/2/mono.jpg]

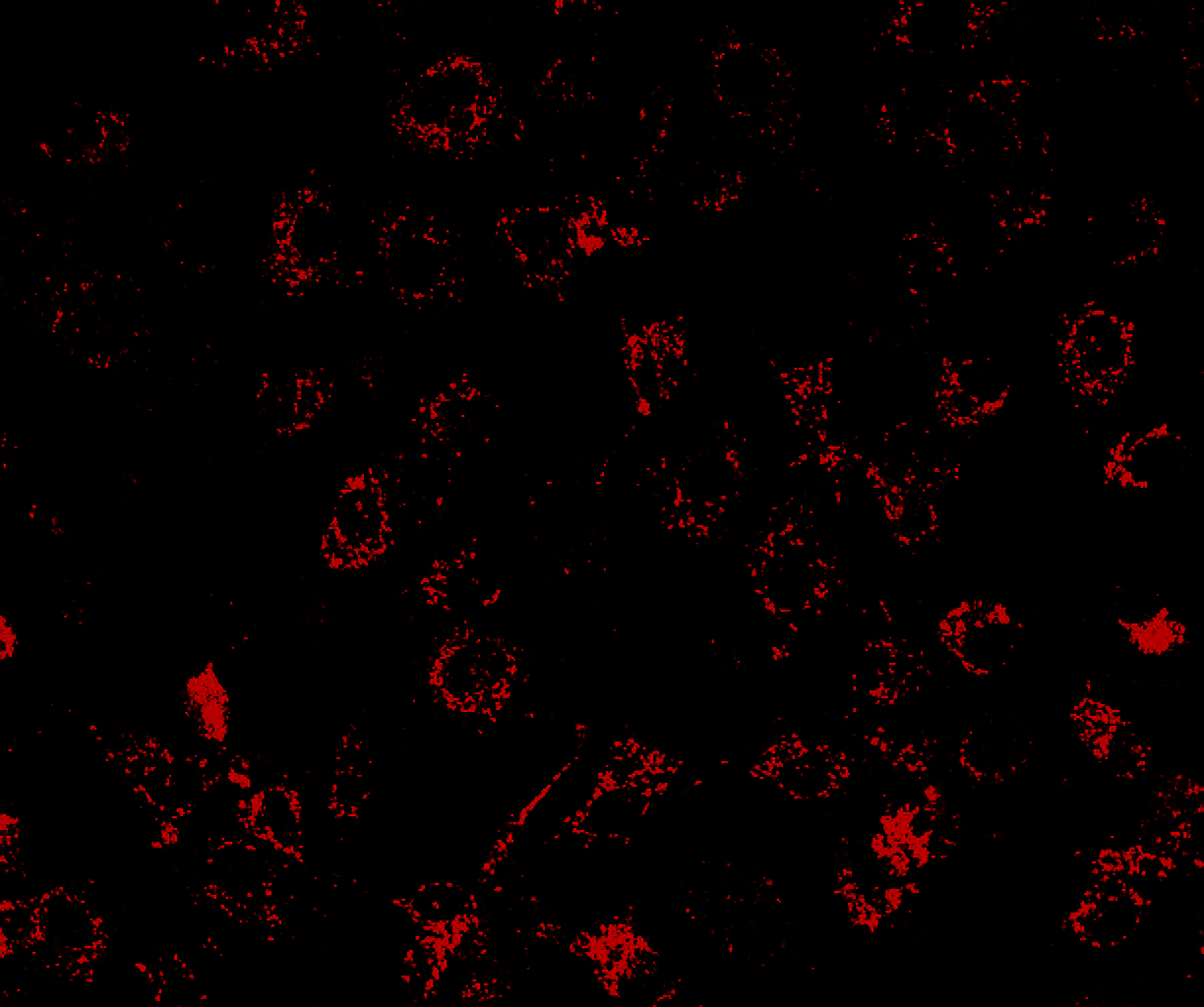

Supplement: Supplementary file 7 [file DataSheet_6.zip › FIG5/miR(+)+SLC7A11(+)/2/poly.jpg]

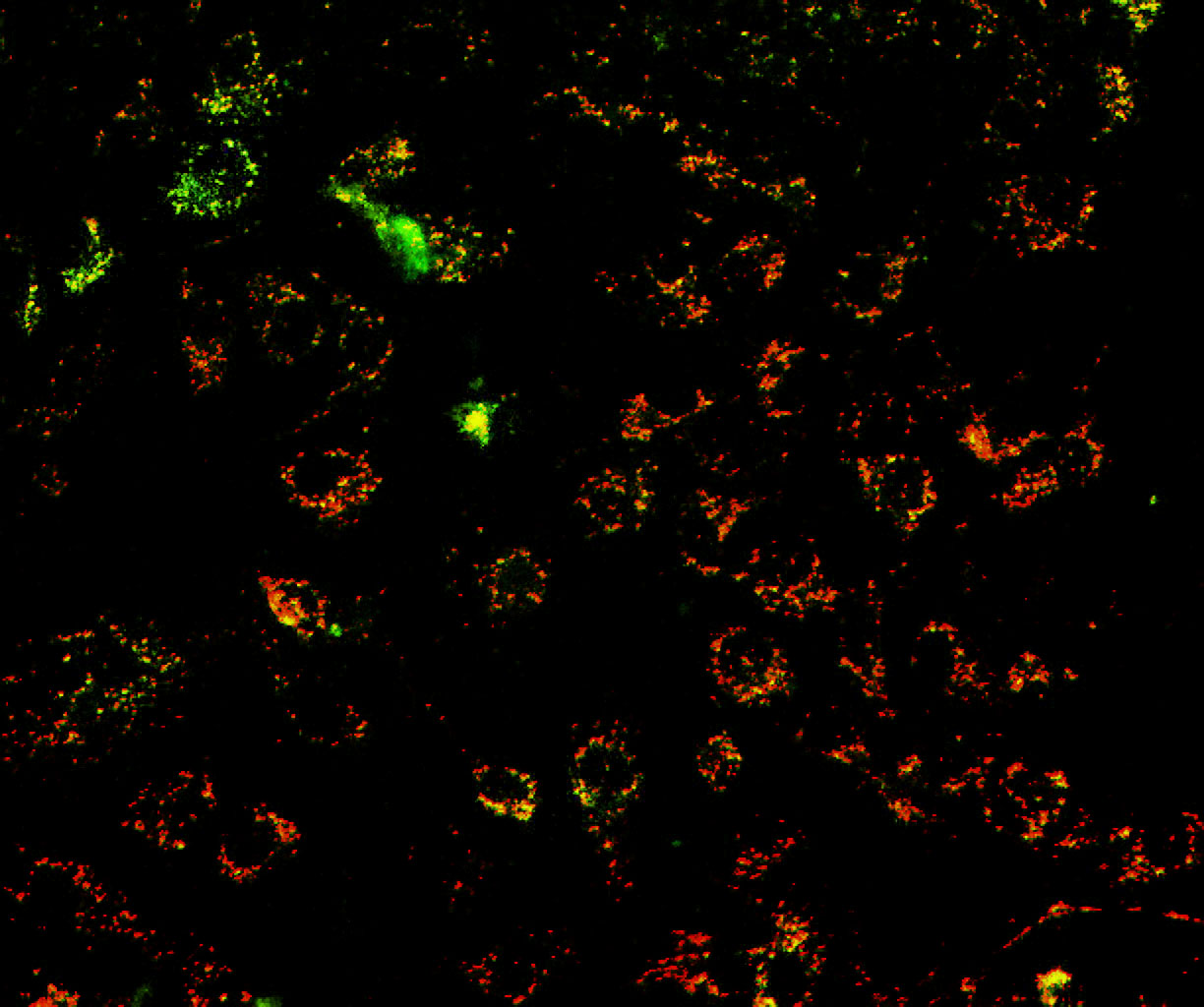

Supplement: Supplementary file 7 [file DataSheet_6.zip › FIG5/miR(+)+SLC7A11(+)/3/merge.jpg]

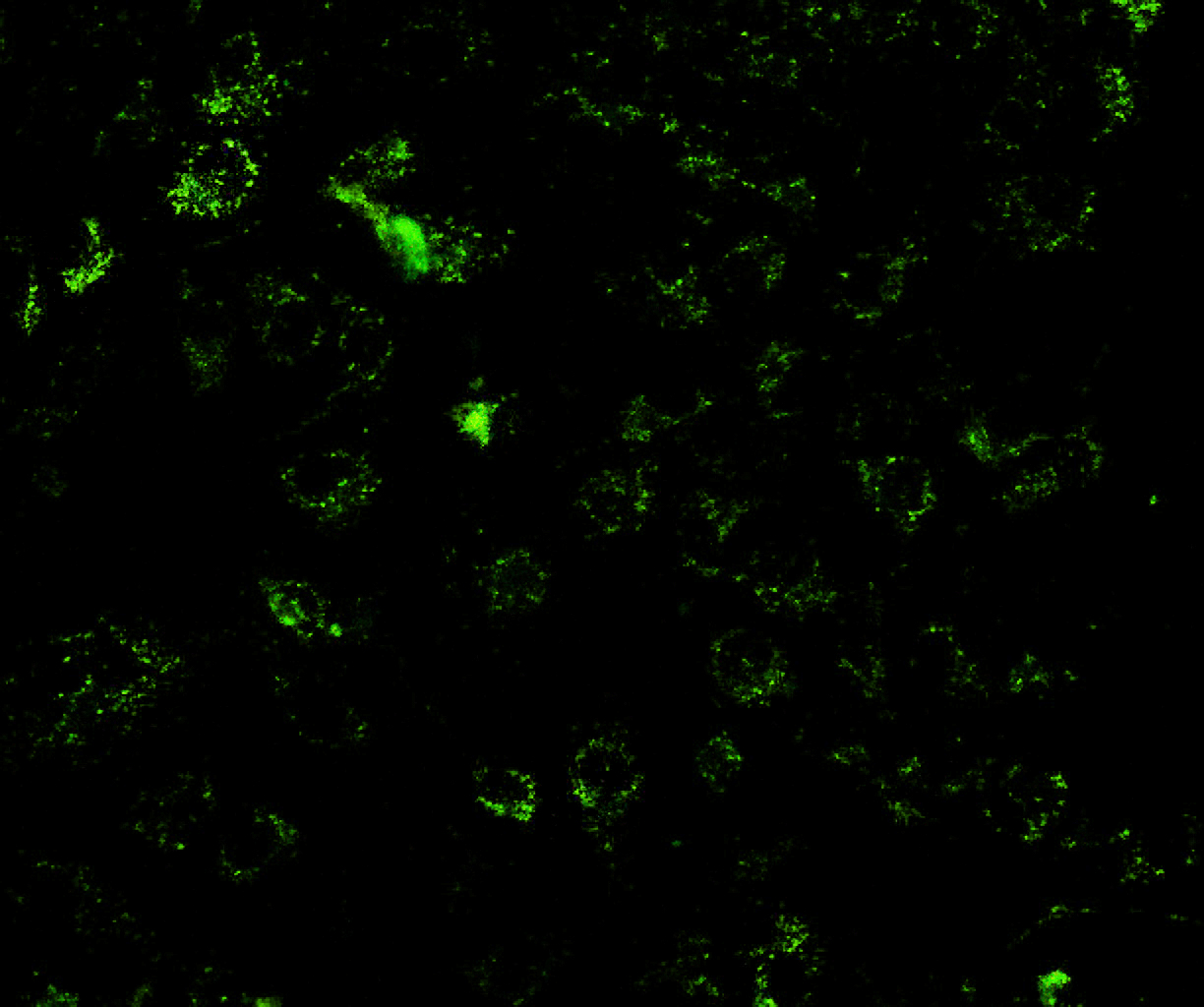

Supplement: Supplementary file 7 [file DataSheet_6.zip › FIG5/miR(+)+SLC7A11(+)/3/mono.jpg]

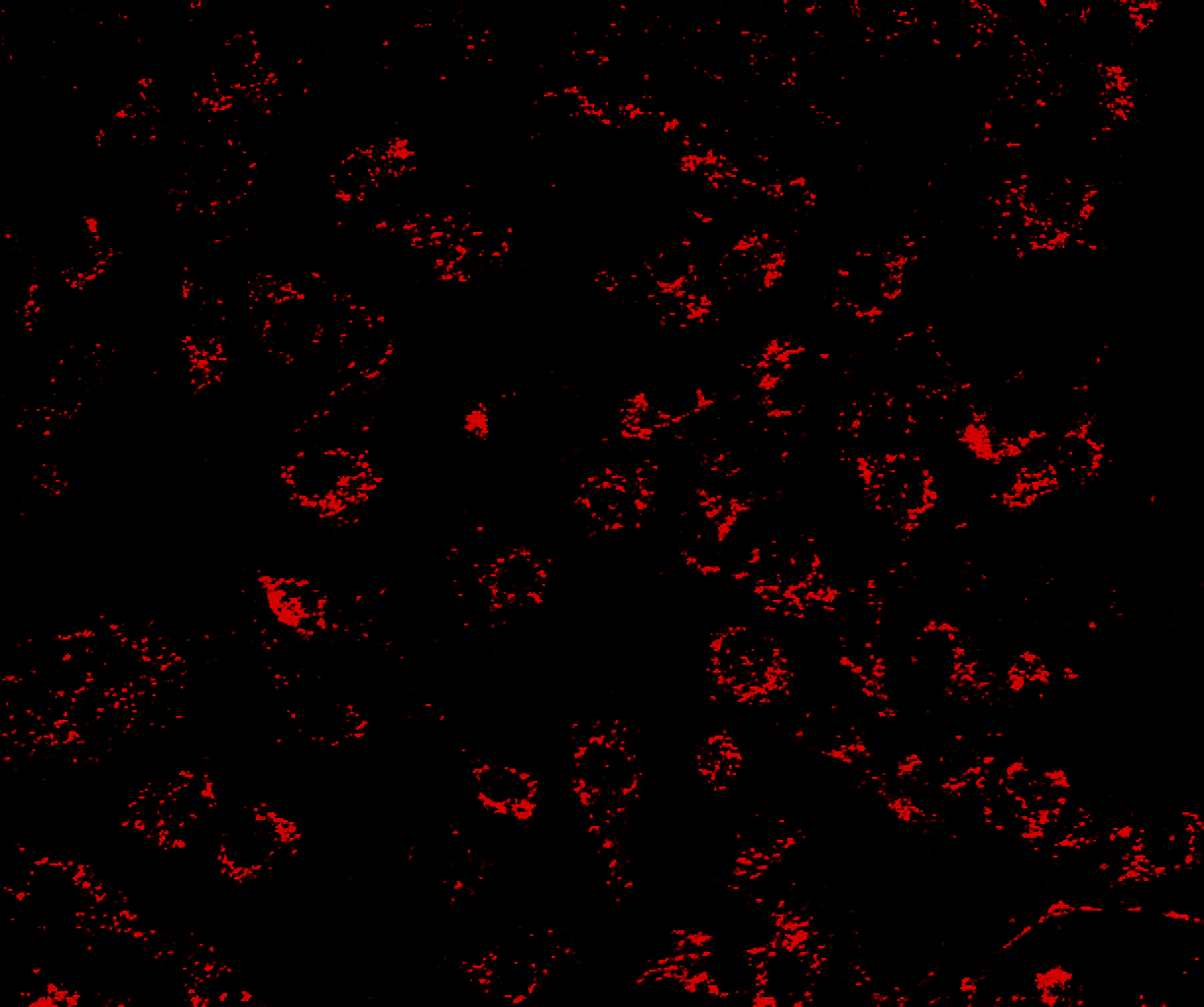

Supplement: Supplementary file 7 [file DataSheet_6.zip › FIG5/miR(+)+SLC7A11(+)/3/poly.jpg]

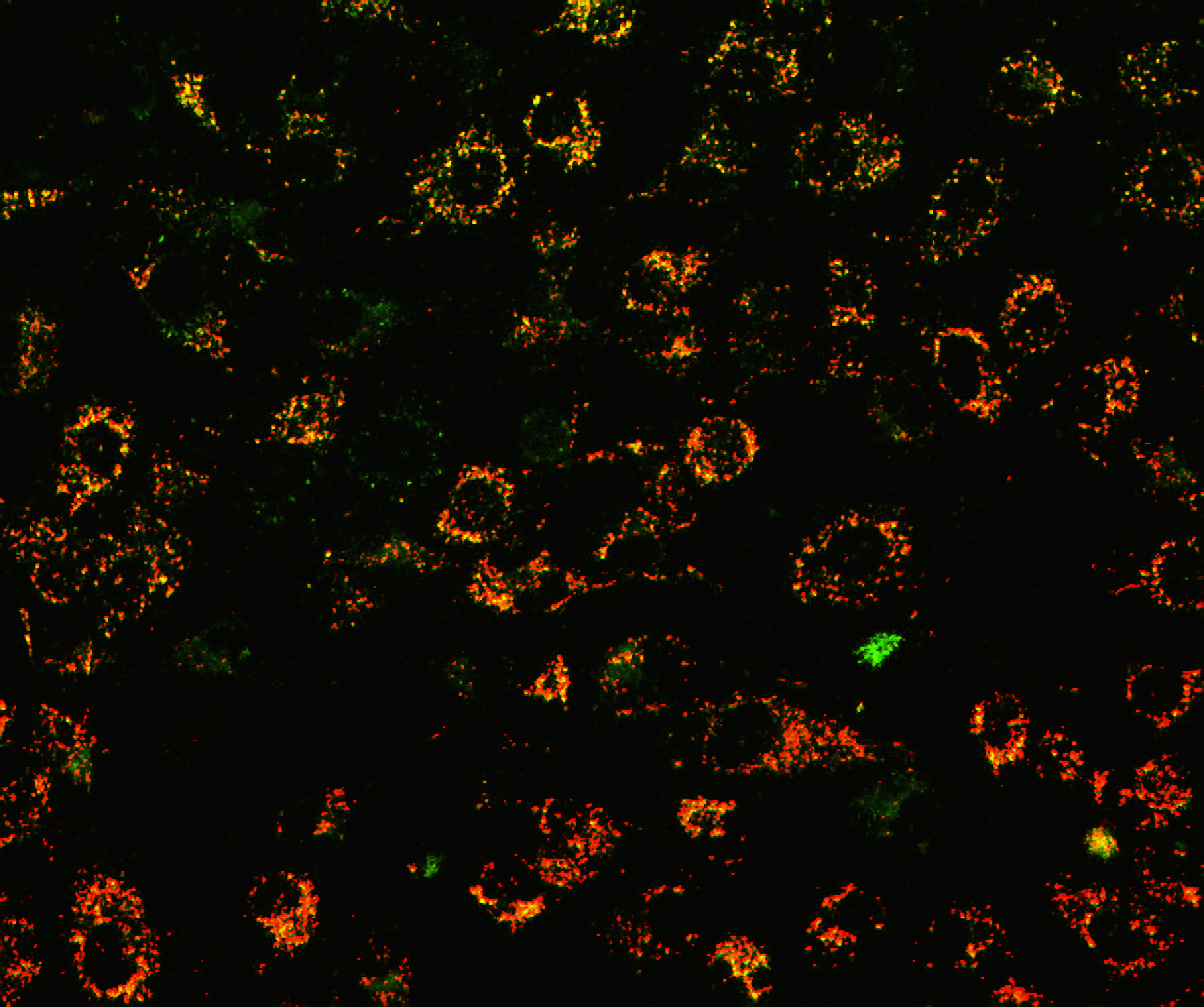

Supplement: Supplementary file 7 [file DataSheet_6.zip › FIG5/NC/1/merge.jpg]

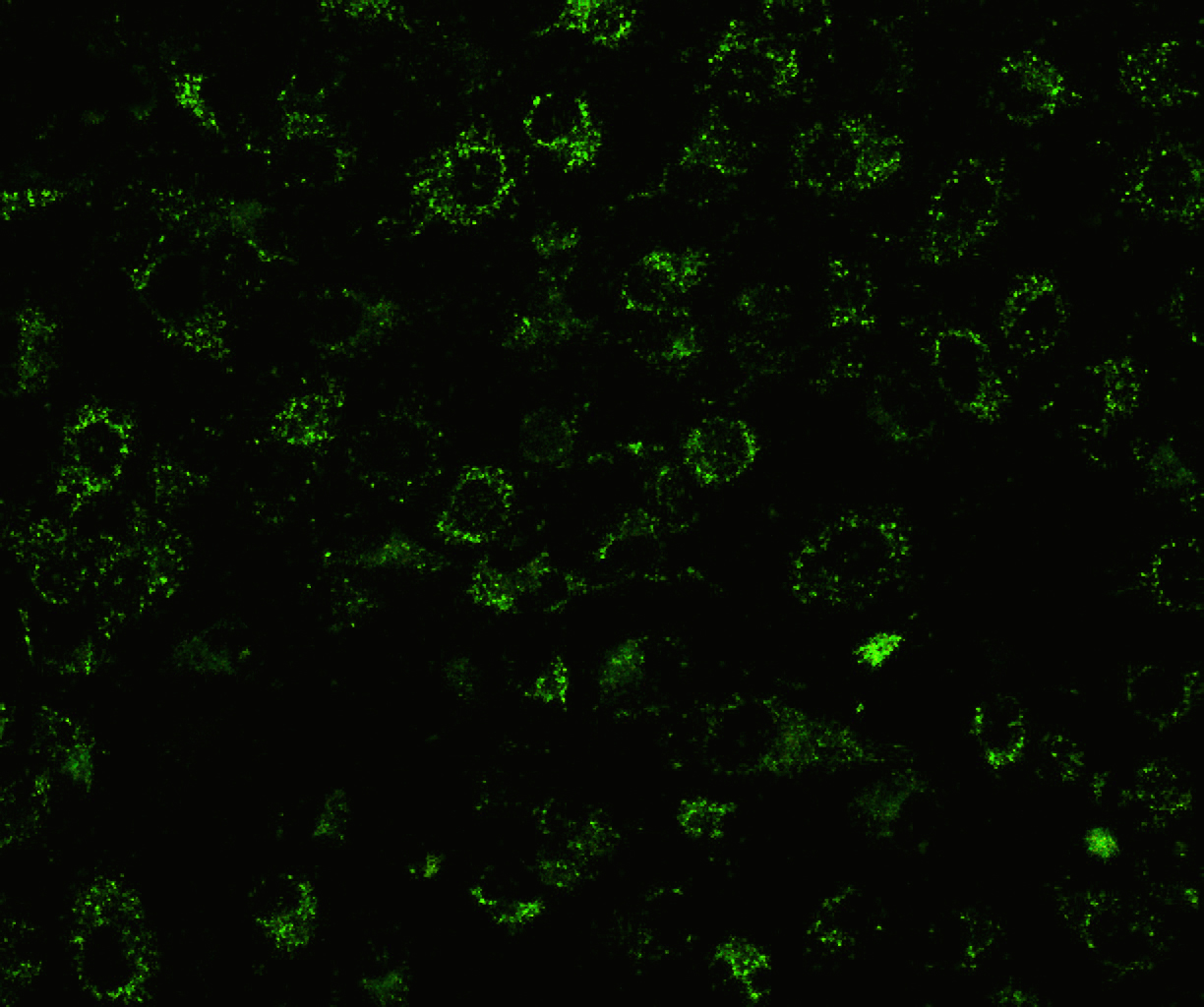

Supplement: Supplementary file 7 [file DataSheet_6.zip › FIG5/NC/1/mono.jpg]

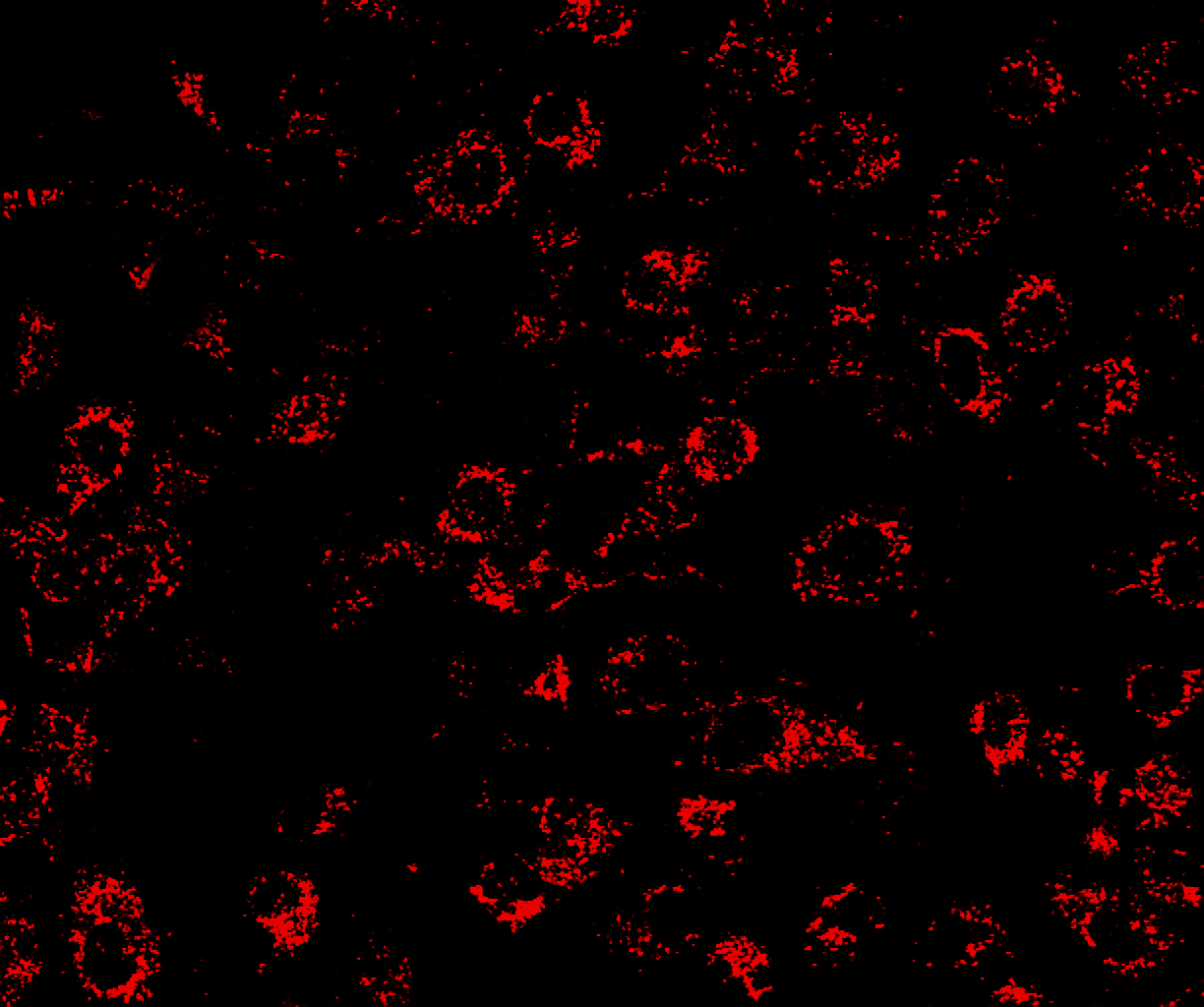

Supplement: Supplementary file 7 [file DataSheet_6.zip › FIG5/NC/1/poly.jpg]

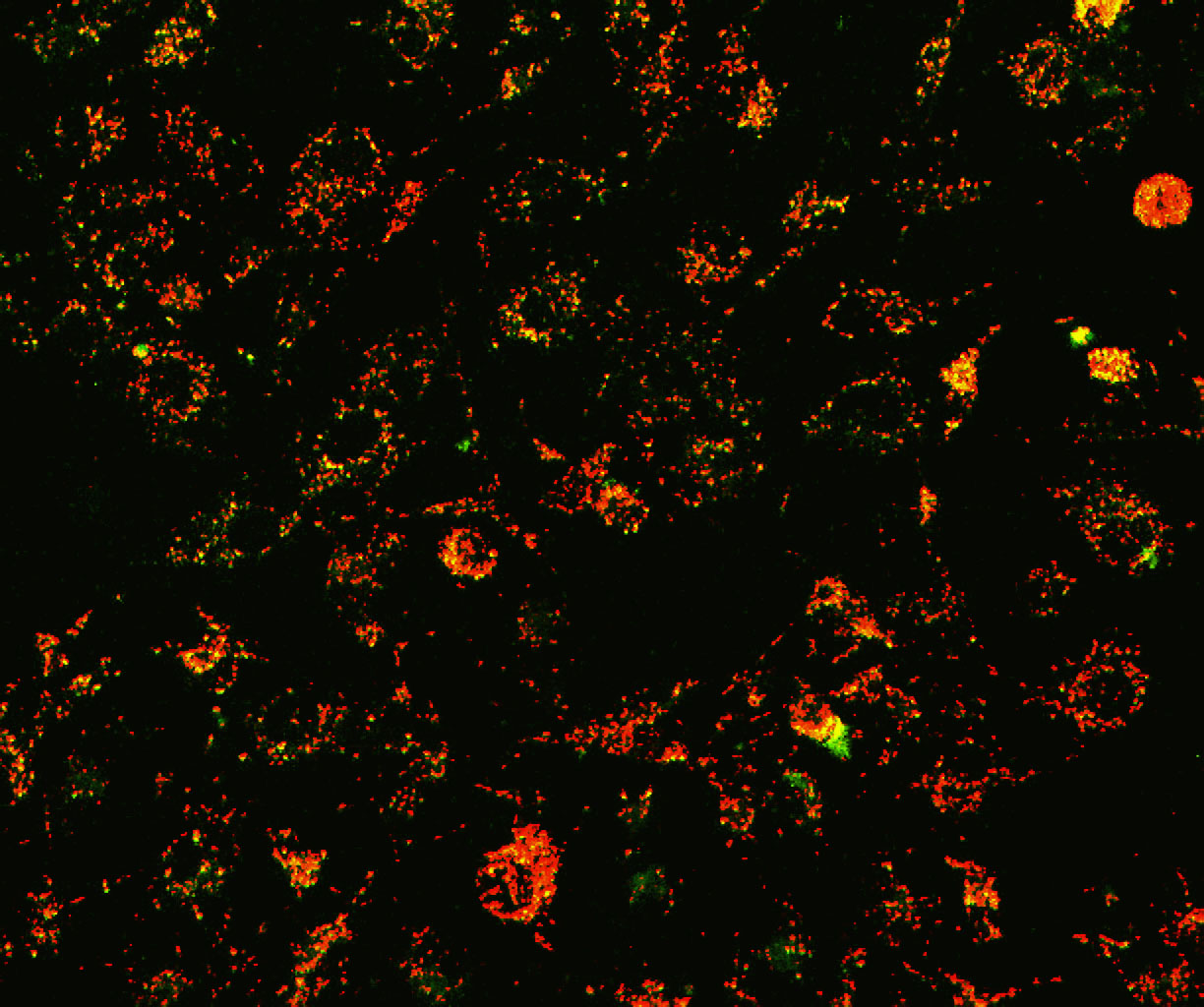

Supplement: Supplementary file 7 [file DataSheet_6.zip › FIG5/NC/2/merge.jpg]

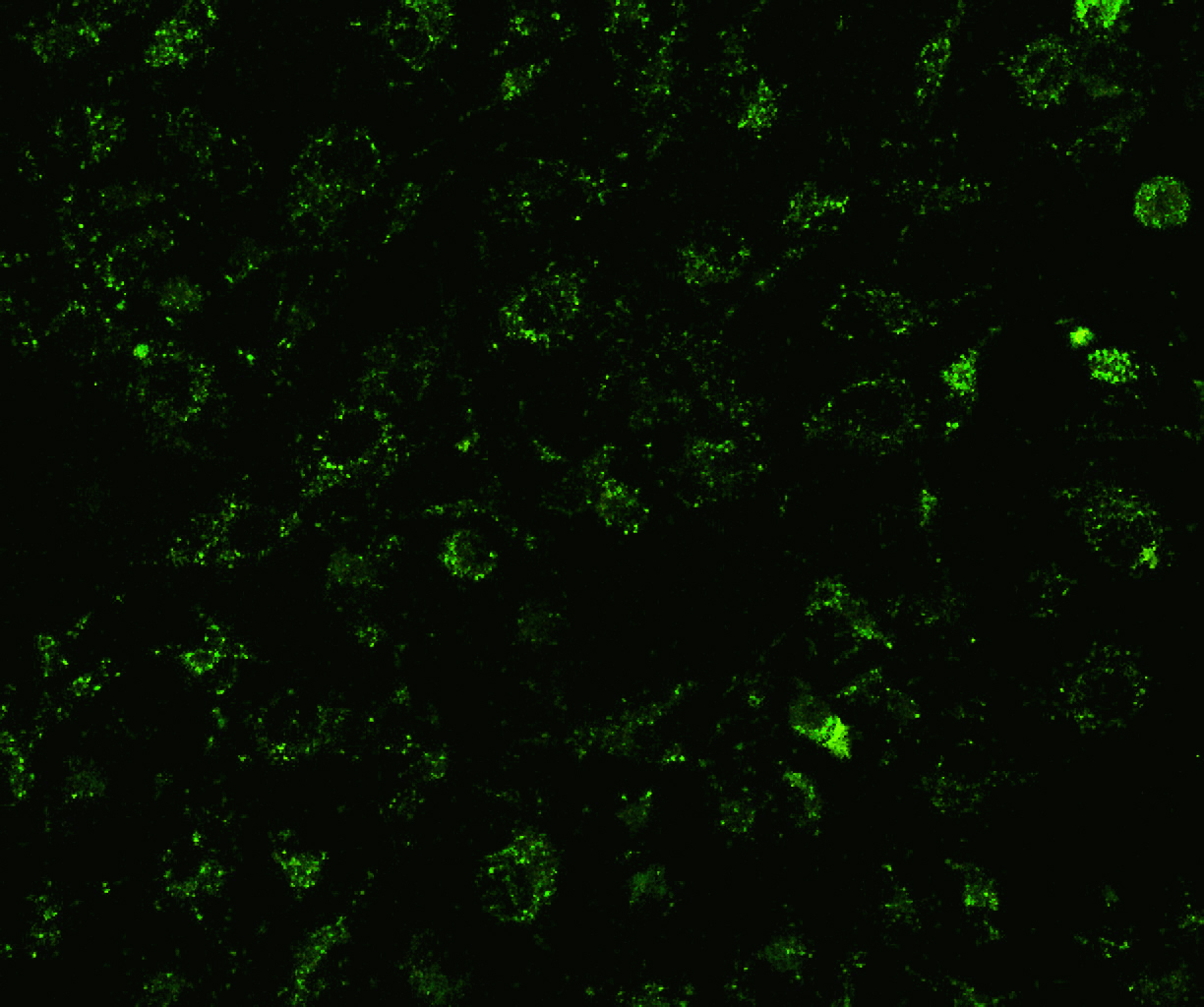

Supplement: Supplementary file 7 [file DataSheet_6.zip › FIG5/NC/2/mono.jpg]

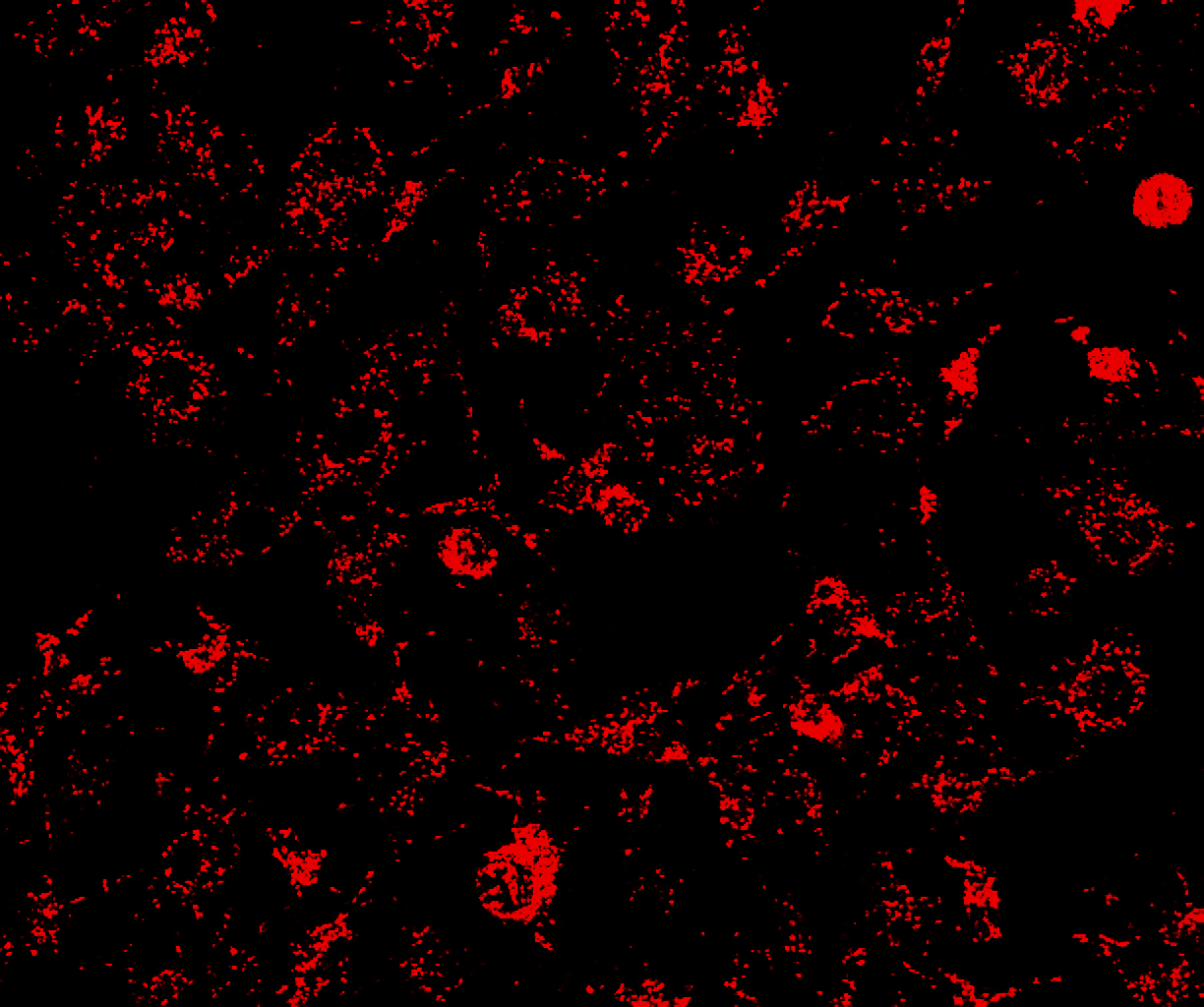

Supplement: Supplementary file 7 [file DataSheet_6.zip › FIG5/NC/2/poly.jpg]

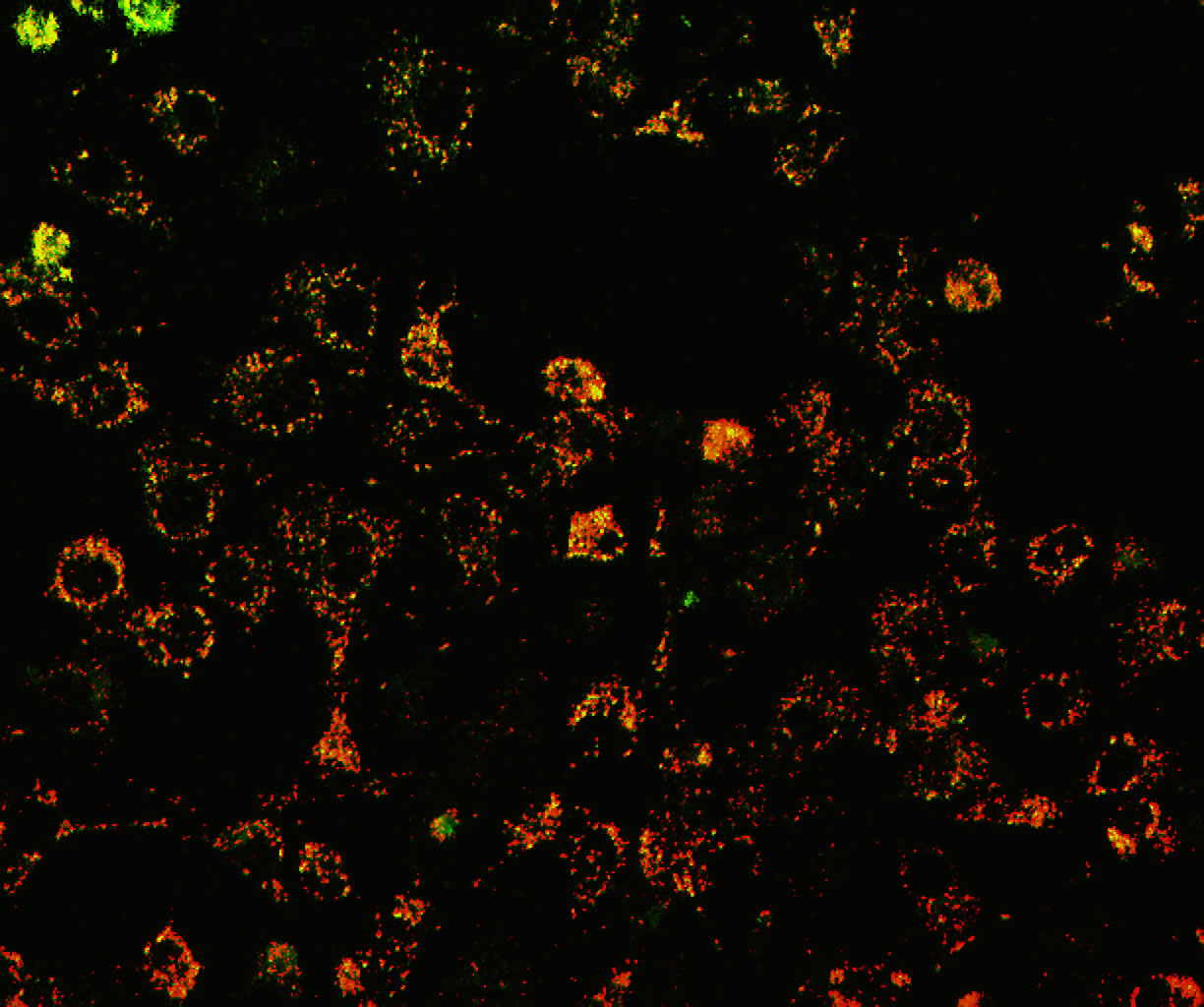

Supplement: Supplementary file 7 [file DataSheet_6.zip › FIG5/NC/3/merge.jpg]

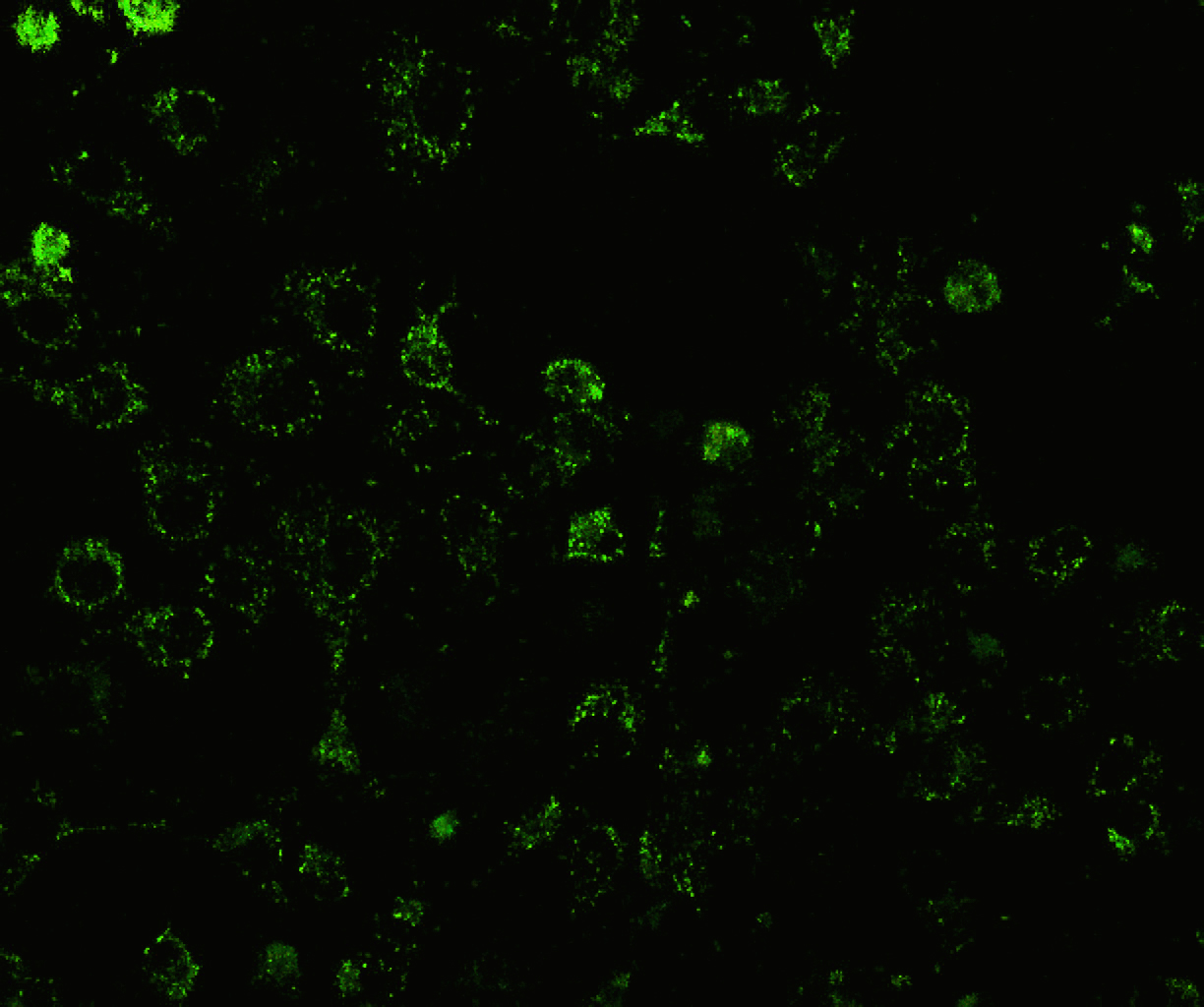

Supplement: Supplementary file 7 [file DataSheet_6.zip › FIG5/NC/3/mono.jpg]

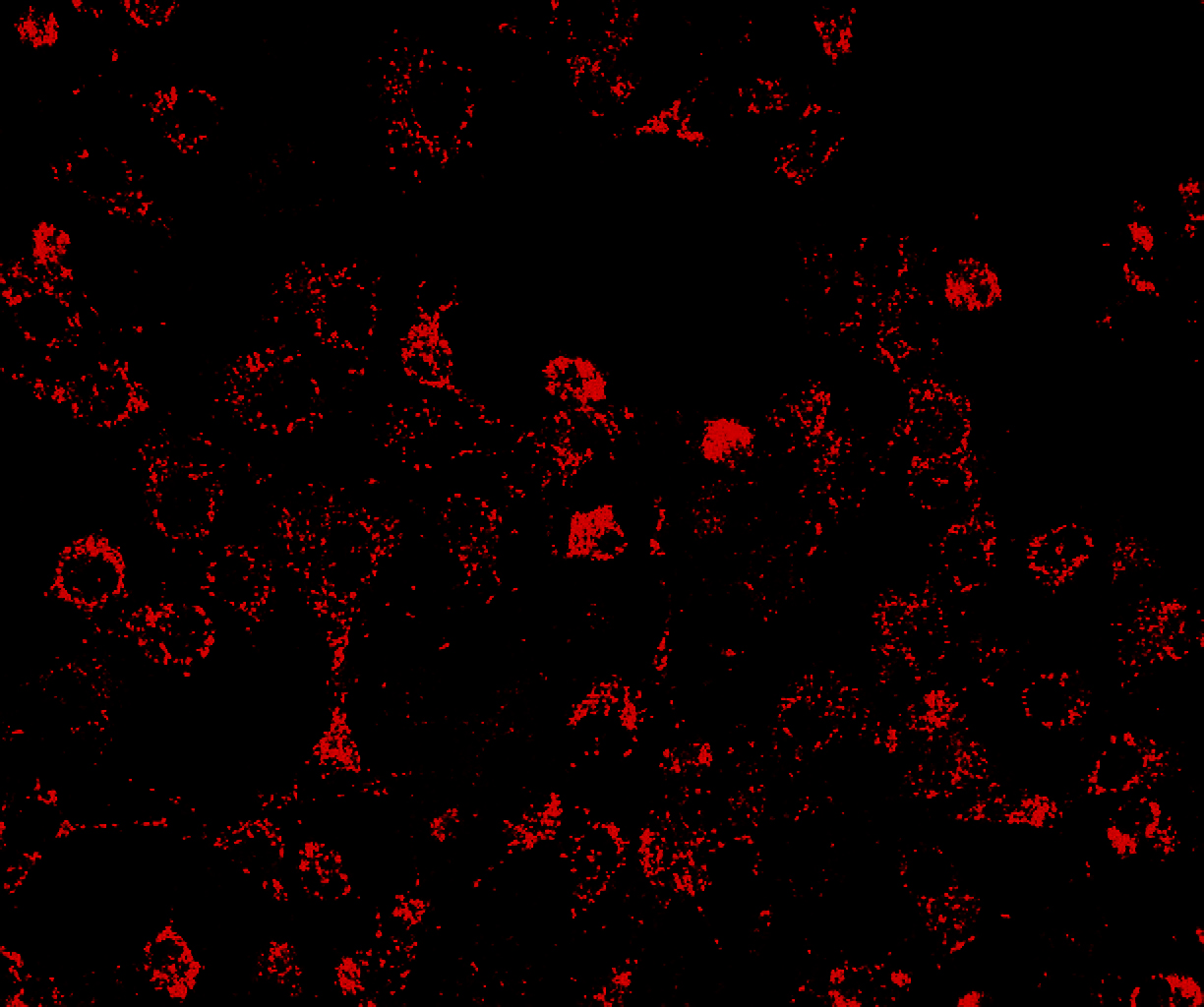

Supplement: Supplementary file 7 [file DataSheet_6.zip › FIG5/NC/3/poly.jpg]

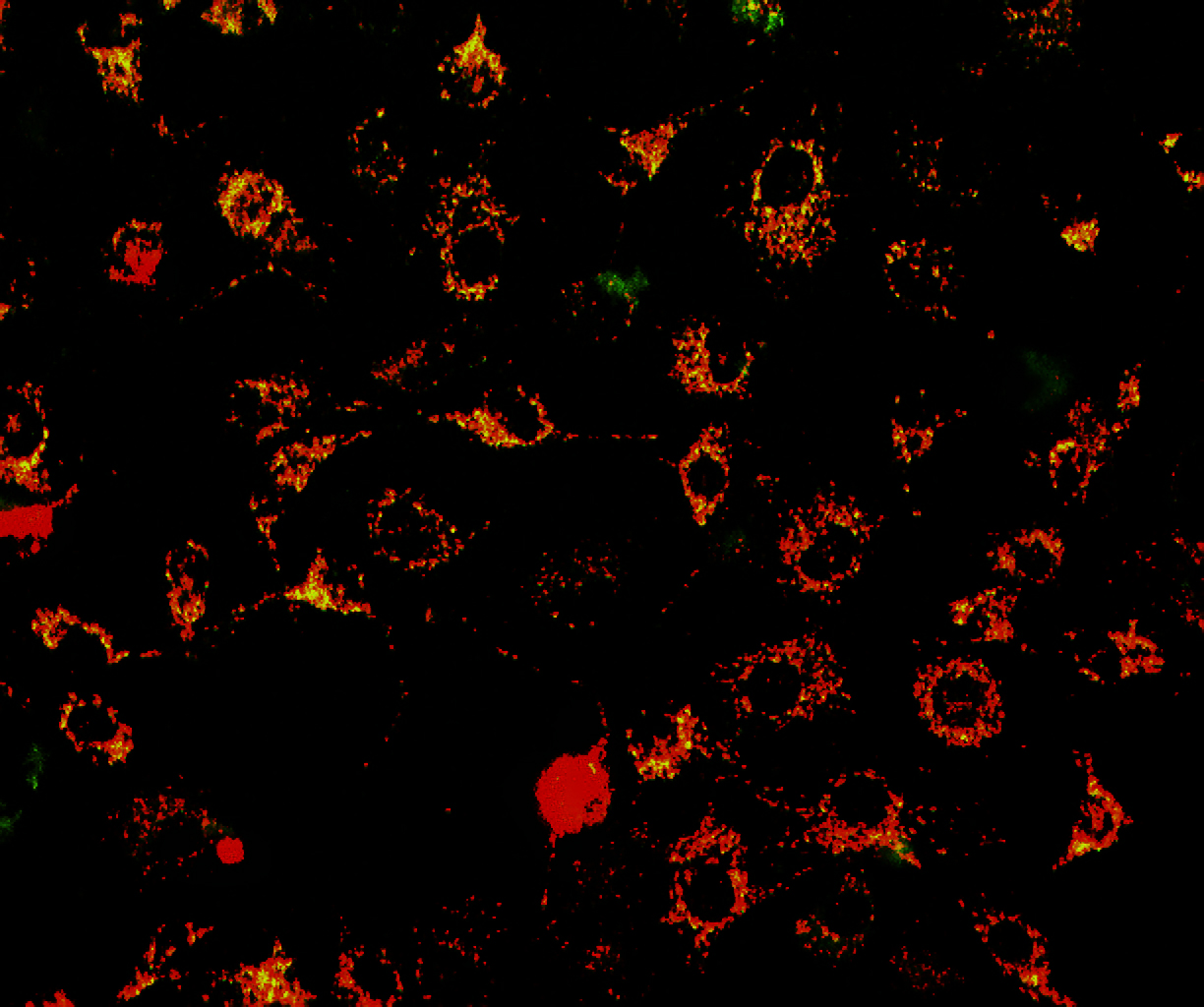

Supplement: Supplementary file 7 [file DataSheet_6.zip › FIG5/SLC7A11(+)/1/merge.jpg]

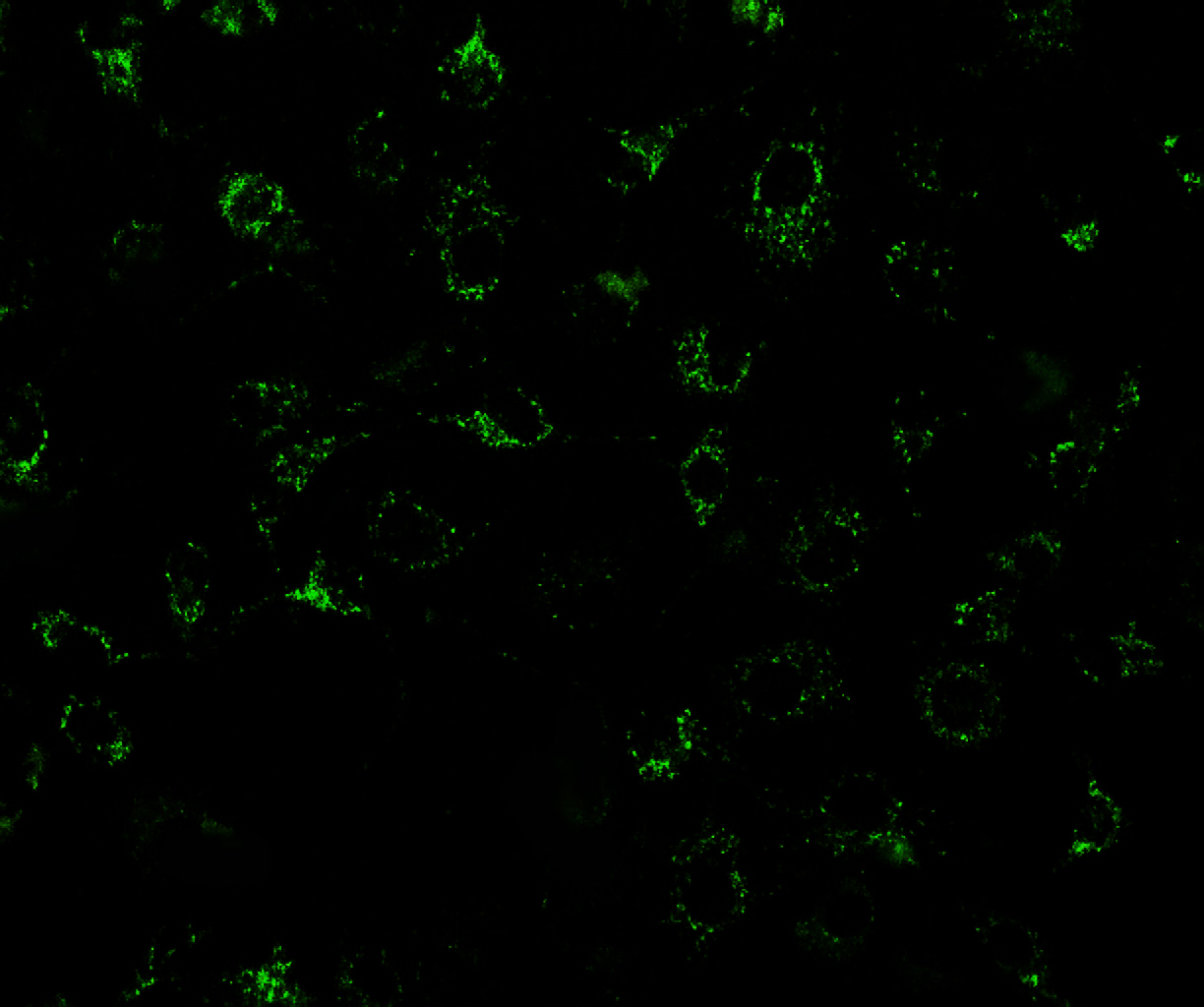

Supplement: Supplementary file 7 [file DataSheet_6.zip › FIG5/SLC7A11(+)/1/mono.jpg]

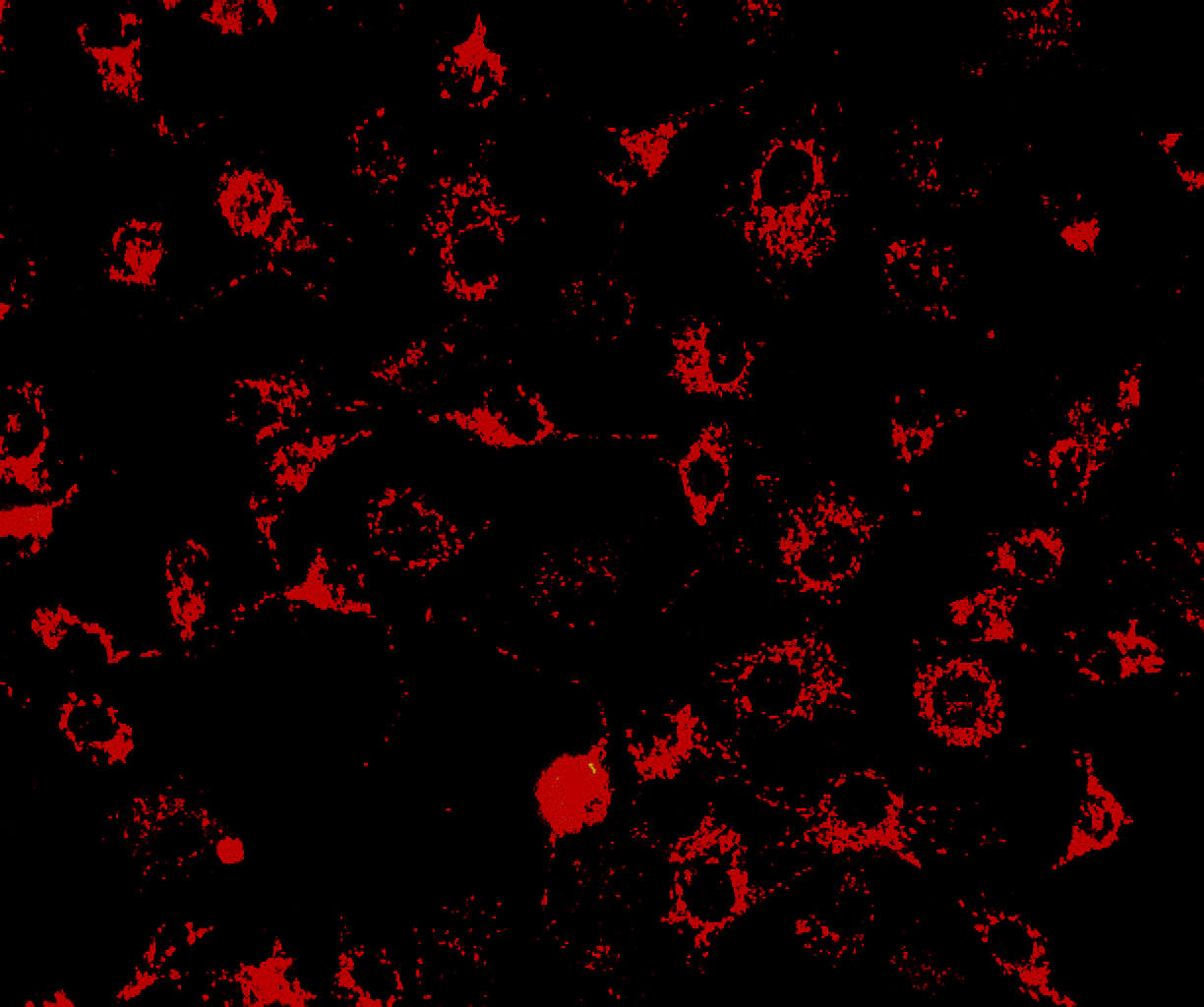

Supplement: Supplementary file 7 [file DataSheet_6.zip › FIG5/SLC7A11(+)/1/poly.jpg]

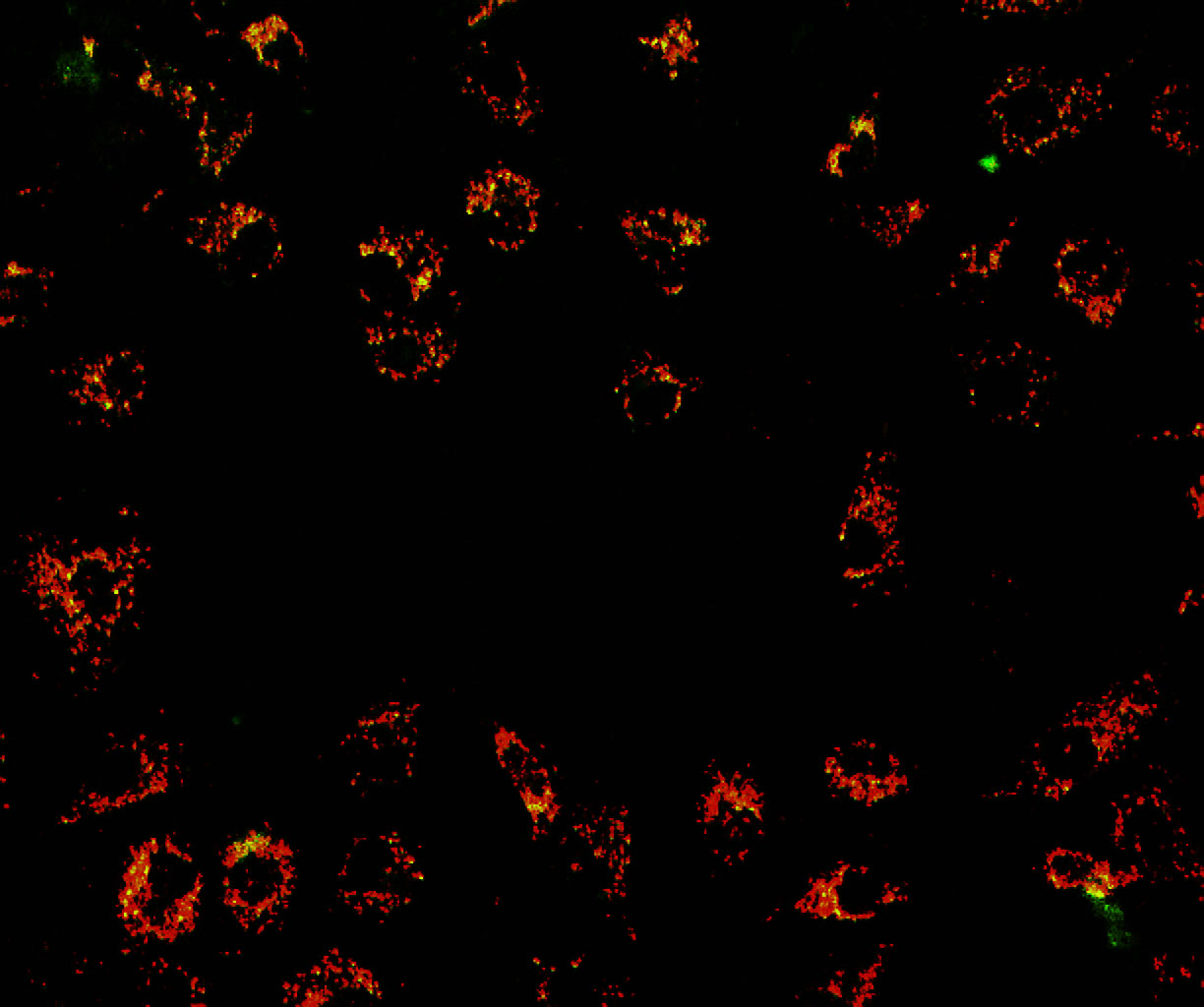

Supplement: Supplementary file 7 [file DataSheet_6.zip › FIG5/SLC7A11(+)/2/merge.jpg]

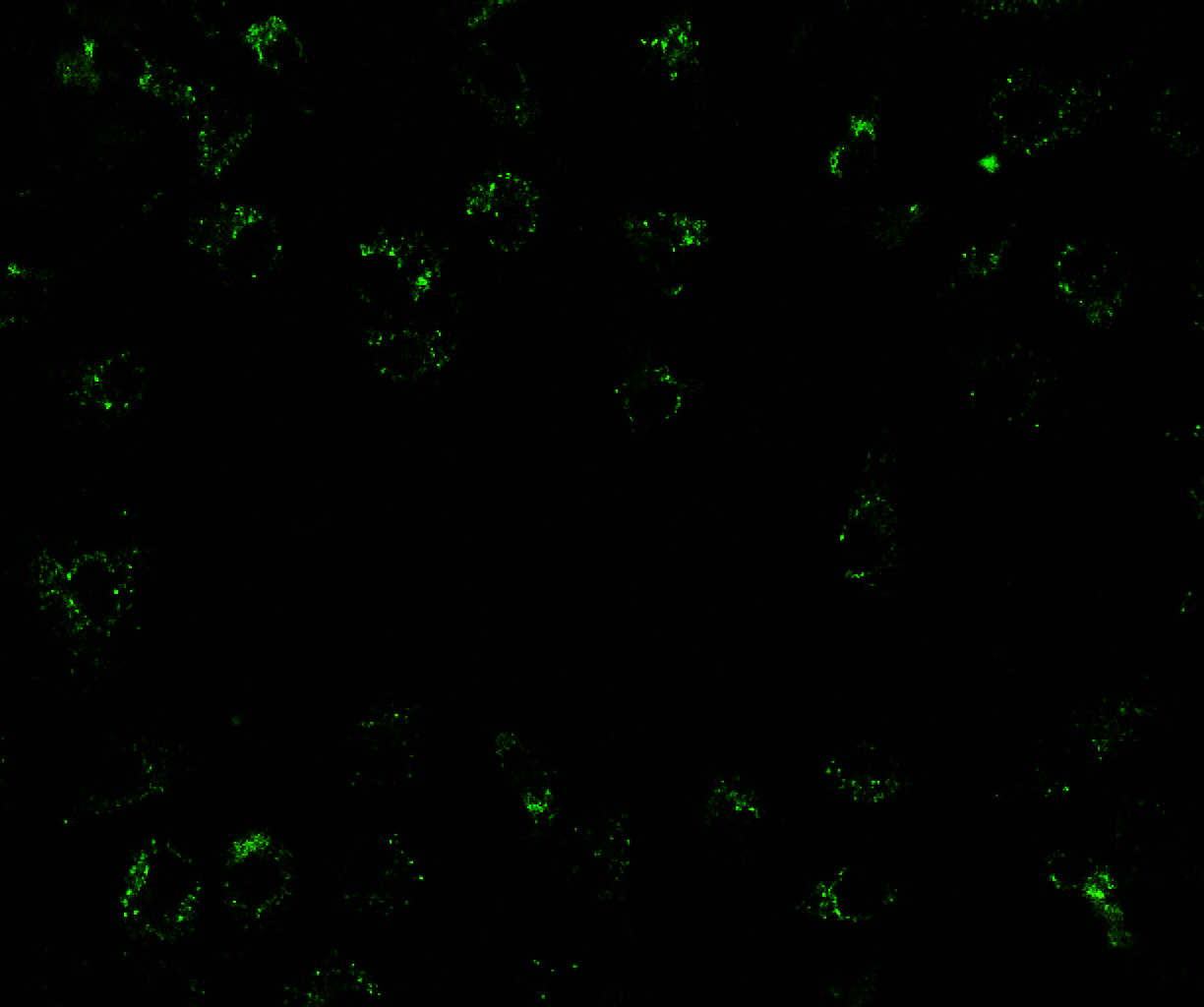

Supplement: Supplementary file 7 [file DataSheet_6.zip › FIG5/SLC7A11(+)/2/mono.jpg]

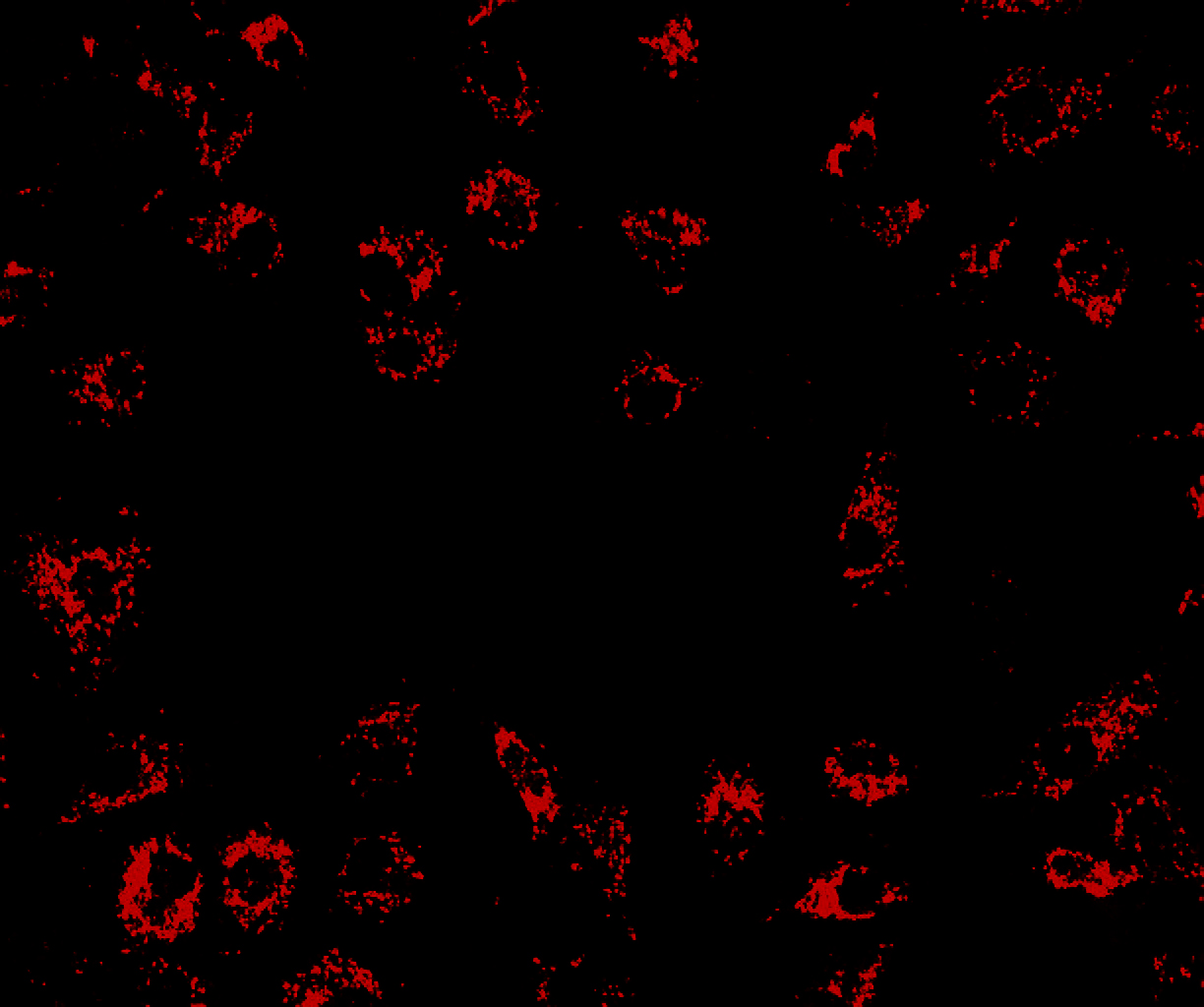

Supplement: Supplementary file 7 [file DataSheet_6.zip › FIG5/SLC7A11(+)/2/poly.jpg]

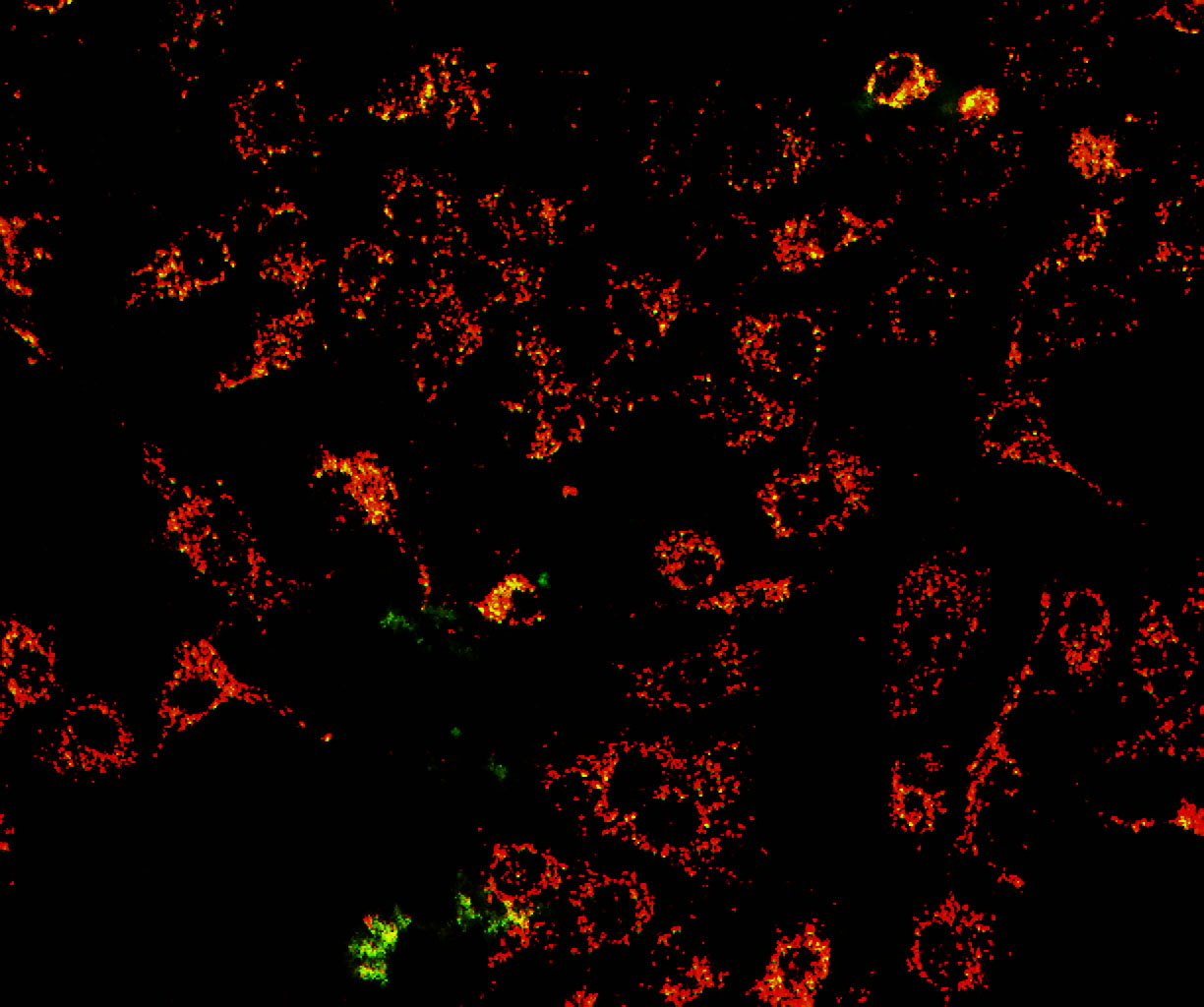

Supplement: Supplementary file 7 [file DataSheet_6.zip › FIG5/SLC7A11(+)/3/merge.jpg]

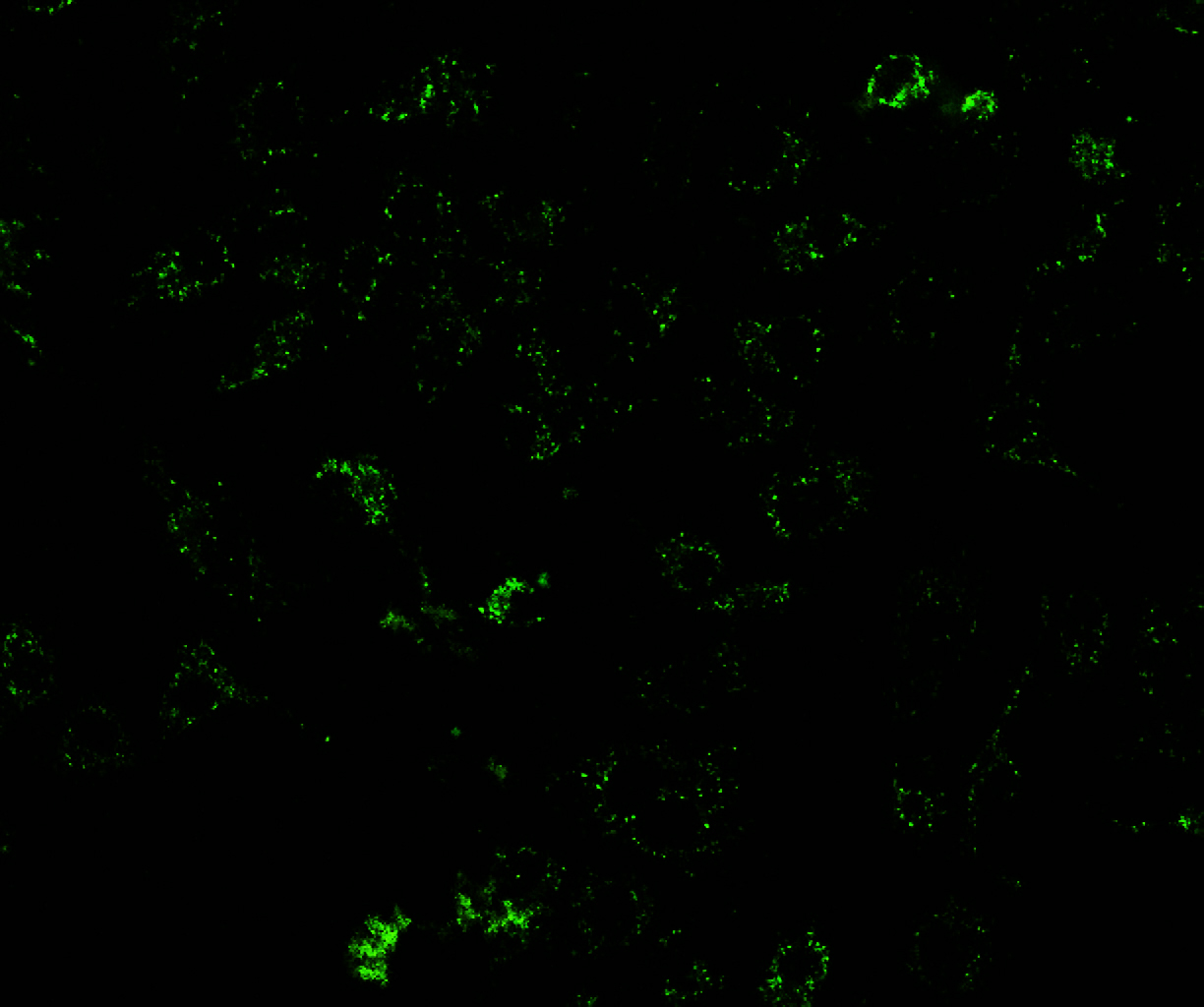

Supplement: Supplementary file 7 [file DataSheet_6.zip › FIG5/SLC7A11(+)/3/mono.jpg]

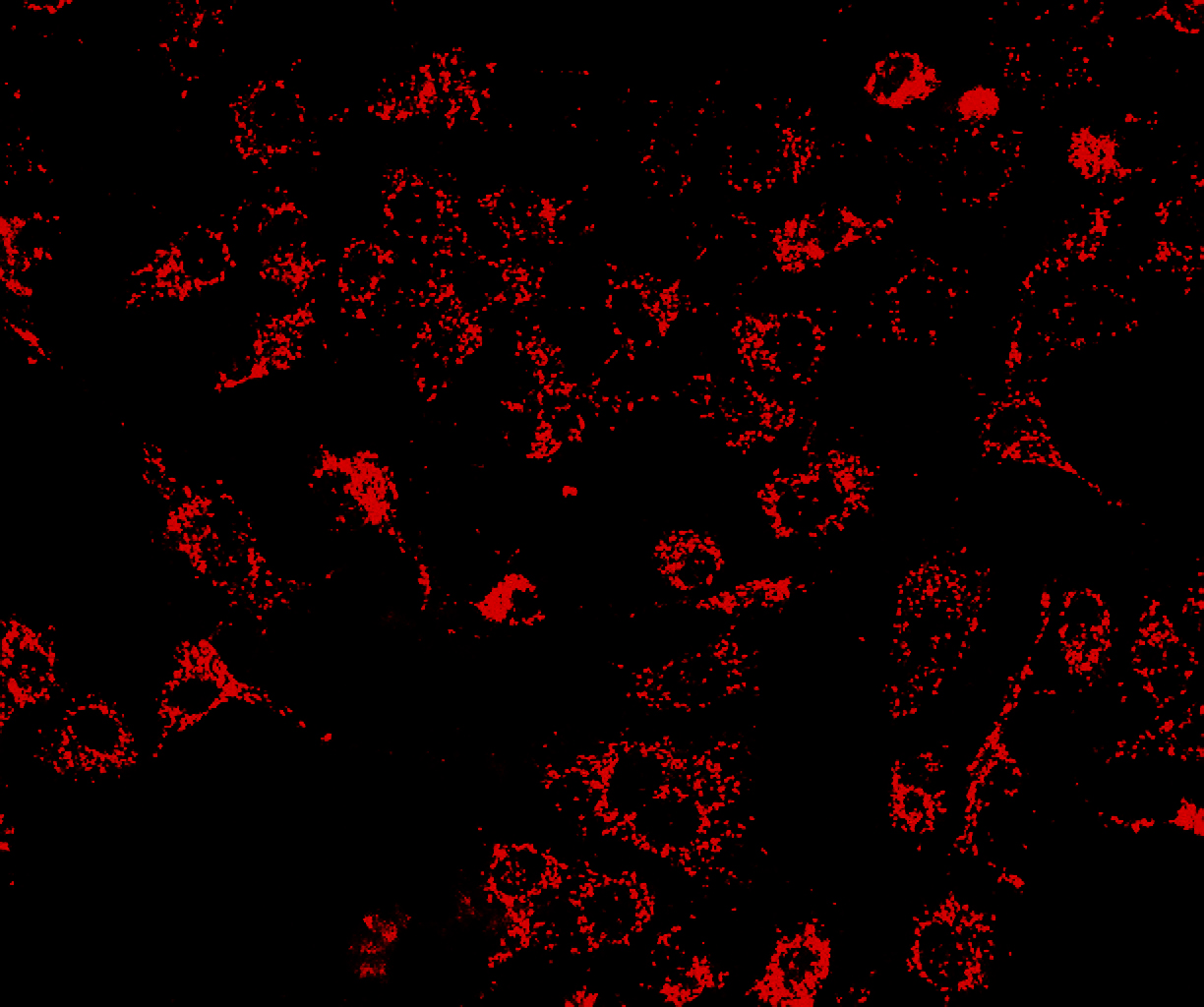

Supplement: Supplementary file 7 [file DataSheet_6.zip › FIG5/SLC7A11(+)/3/poly.jpg]

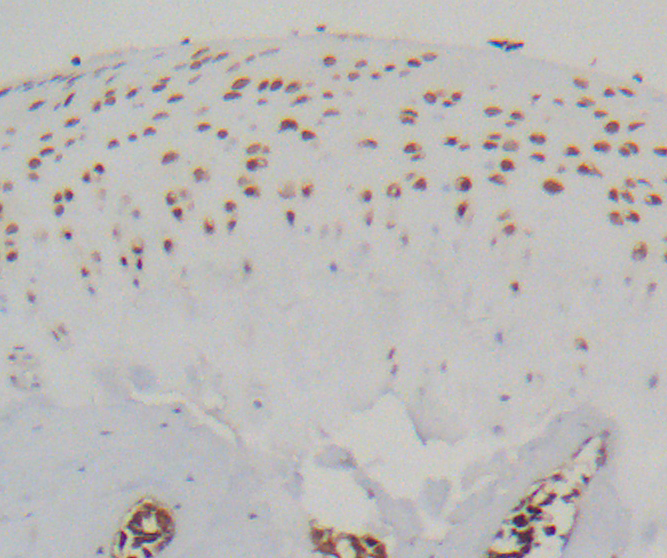

Supplement: Supplementary file 8 [file DataSheet_7.zip › ACSL4/Exo/1.jpg]

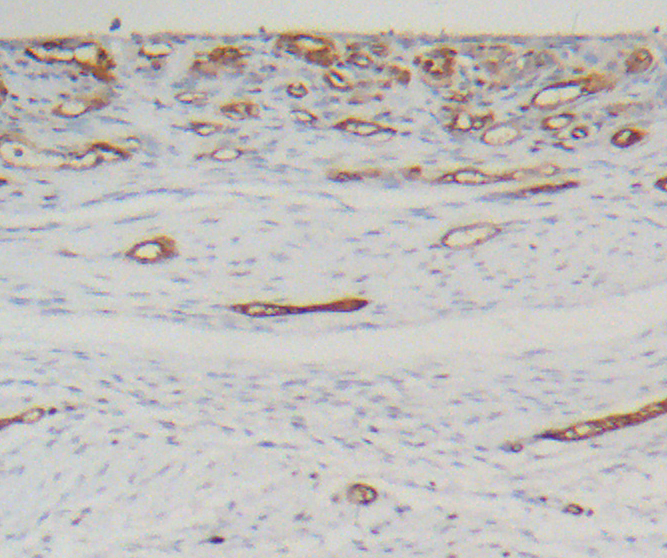

Supplement: Supplementary file 8 [file DataSheet_7.zip › ACSL4/Exo/2.jpg]

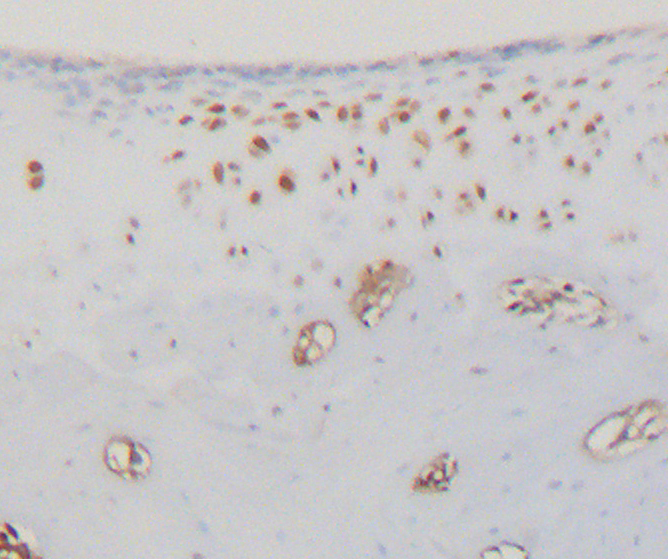

Supplement: Supplementary file 8 [file DataSheet_7.zip › ACSL4/Exo/3.jpg]

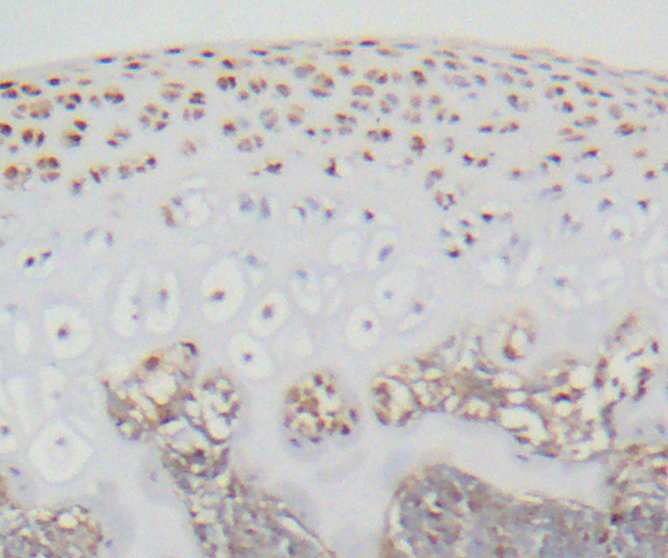

Supplement: Supplementary file 8 [file DataSheet_7.zip › ACSL4/Exo/4.jpg]

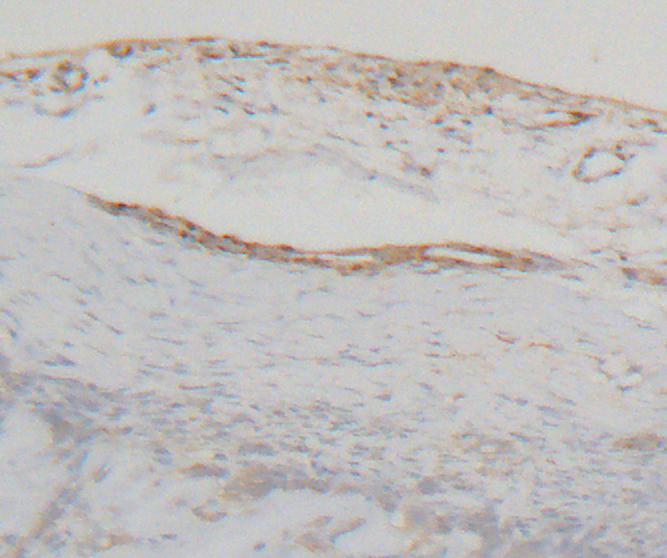

Supplement: Supplementary file 8 [file DataSheet_7.zip › ACSL4/Exo/5.jpg]

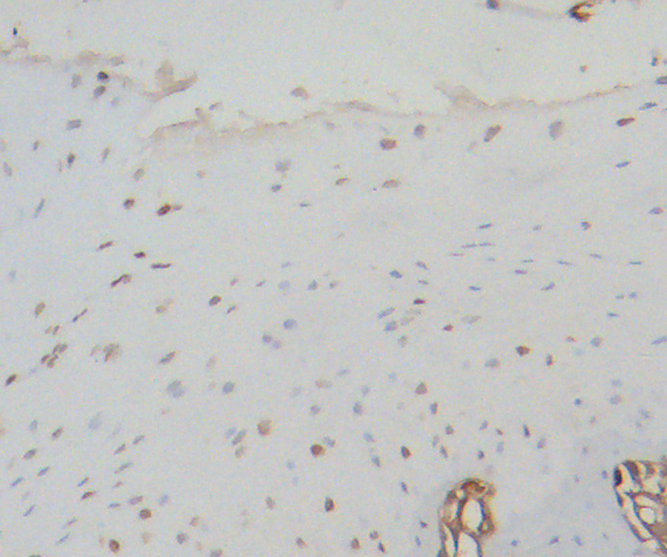

Supplement: Supplementary file 8 [file DataSheet_7.zip › ACSL4/Exo/6.jpg]

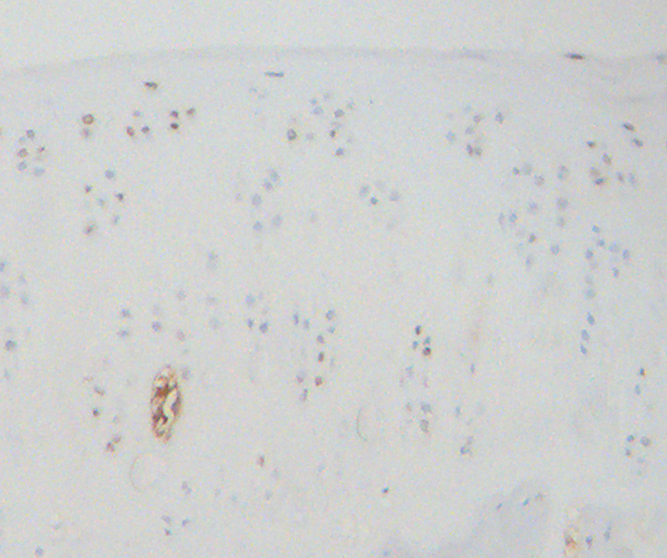

Supplement: Supplementary file 8 [file DataSheet_7.zip › ACSL4/Exo+Fer-1/1.jpg]

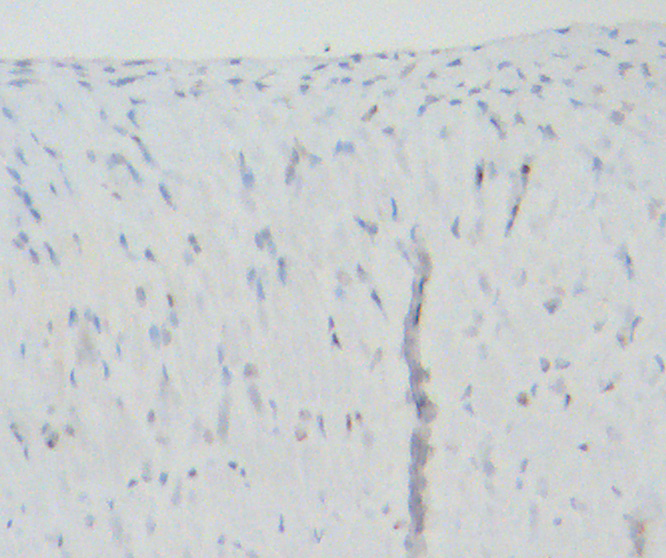

Supplement: Supplementary file 8 [file DataSheet_7.zip › ACSL4/Exo+Fer-1/2.jpg]

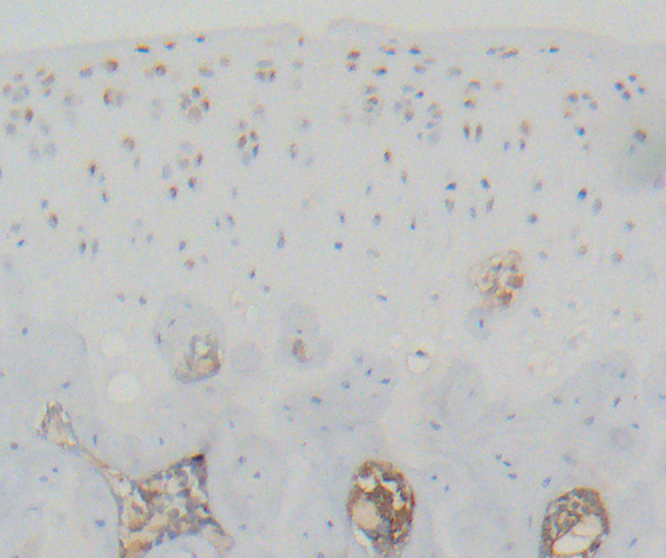

Supplement: Supplementary file 8 [file DataSheet_7.zip › ACSL4/Exo+Fer-1/3.jpg]

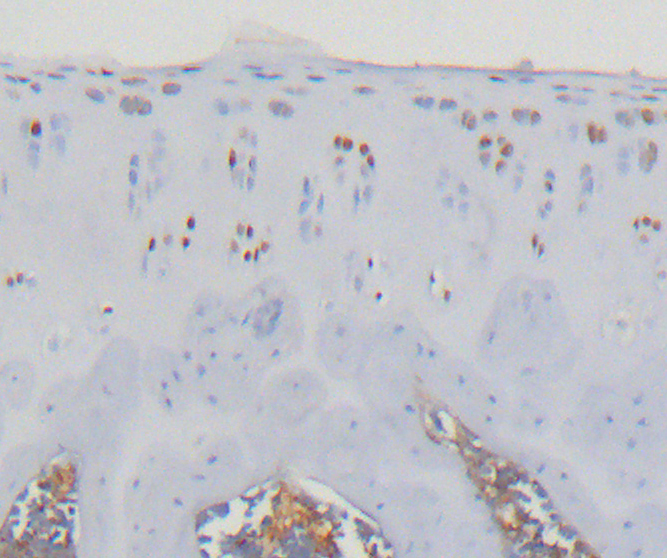

Supplement: Supplementary file 8 [file DataSheet_7.zip › ACSL4/Exo+Fer-1/4.jpg]

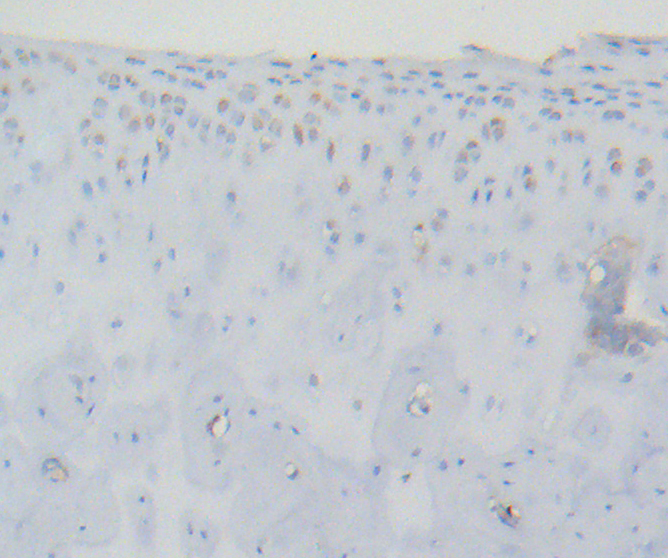

Supplement: Supplementary file 8 [file DataSheet_7.zip › ACSL4/Exo+Fer-1/5.jpg]

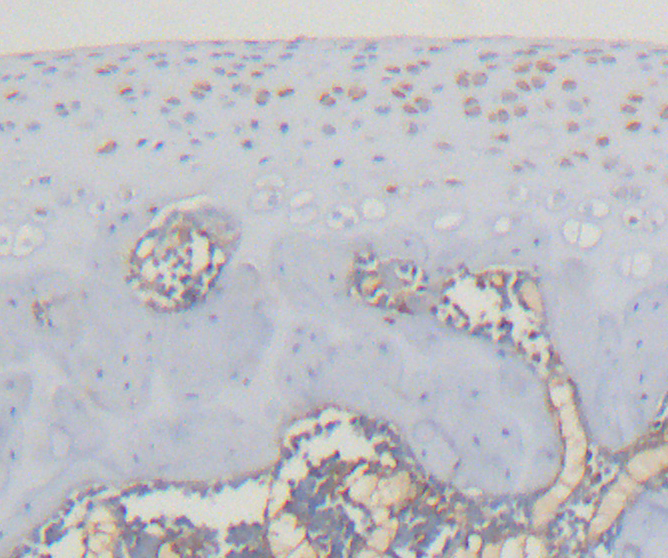

Supplement: Supplementary file 8 [file DataSheet_7.zip › ACSL4/Exo+Fer-1/6.jpg]

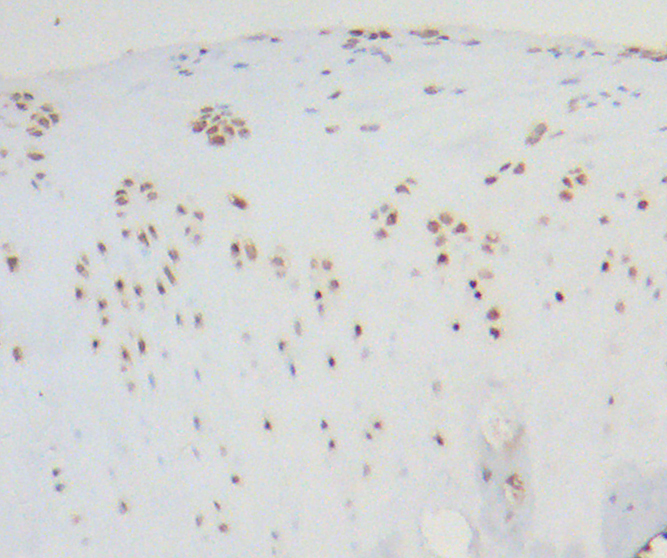

Supplement: Supplementary file 8 [file DataSheet_7.zip › ACSL4/miR(-) Exo/1.jpg]

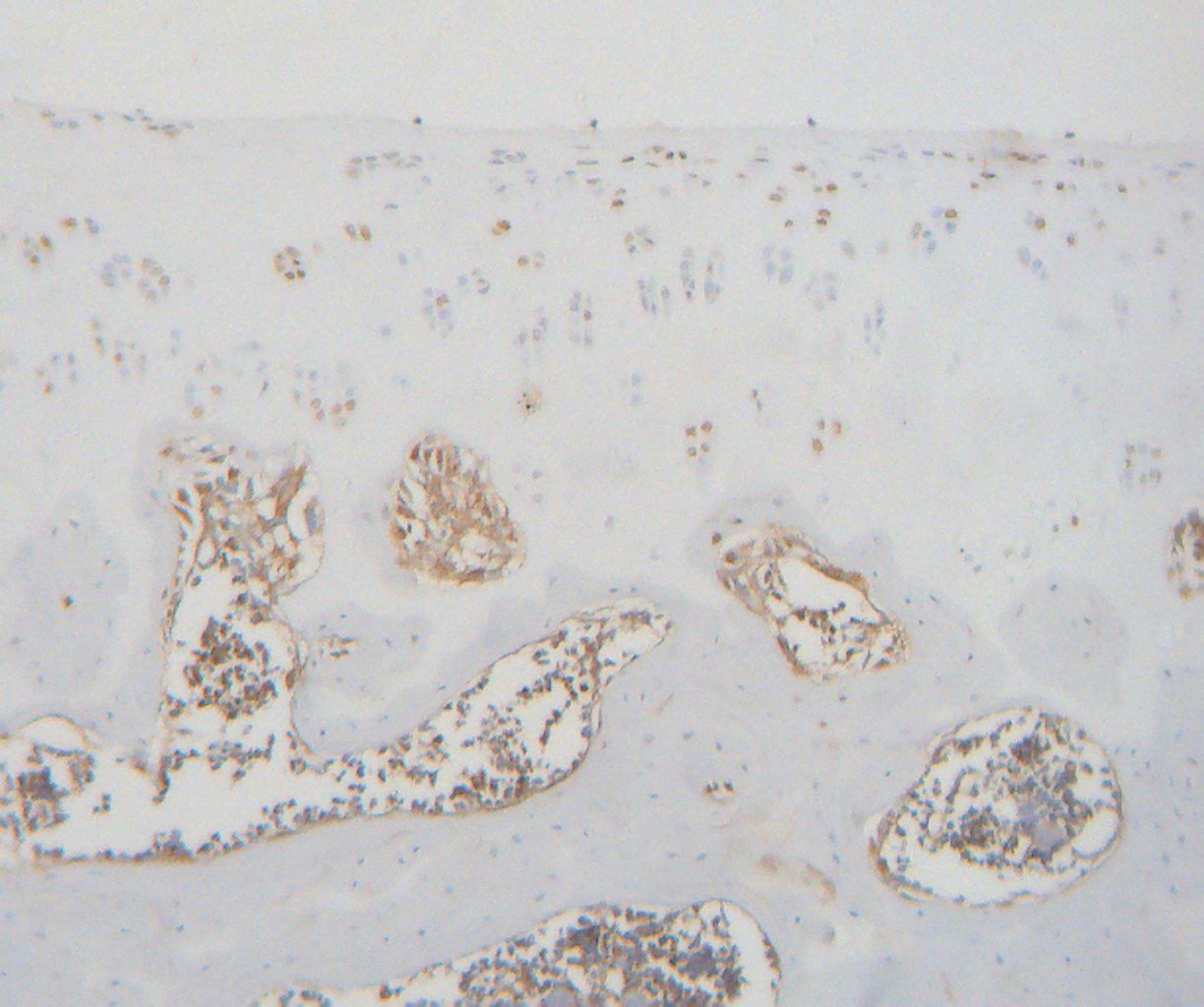

Supplement: Supplementary file 8 [file DataSheet_7.zip › ACSL4/miR(-) Exo/2.jpg]

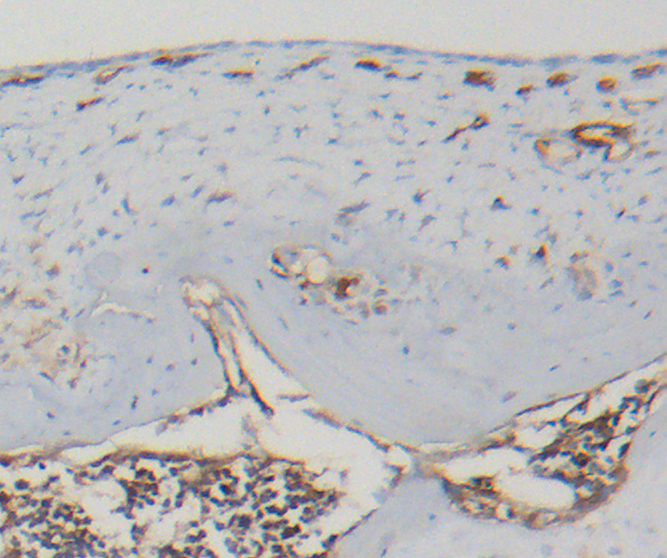

Supplement: Supplementary file 8 [file DataSheet_7.zip › ACSL4/miR(-) Exo/3.jpg]

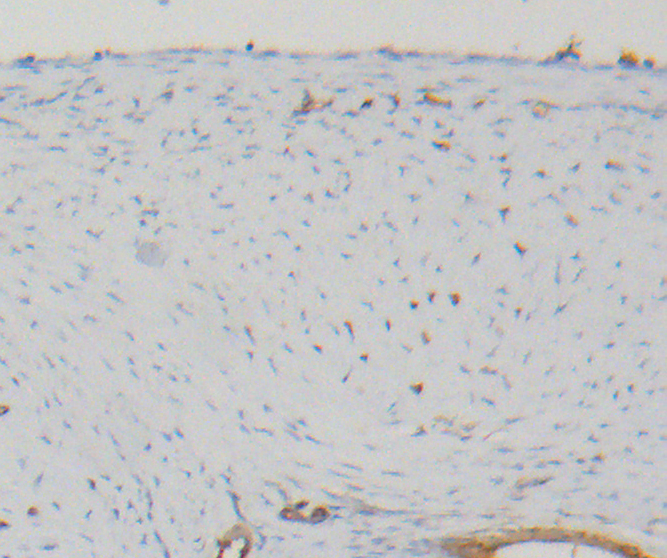

Supplement: Supplementary file 8 [file DataSheet_7.zip › ACSL4/miR(-) Exo/4.jpg]

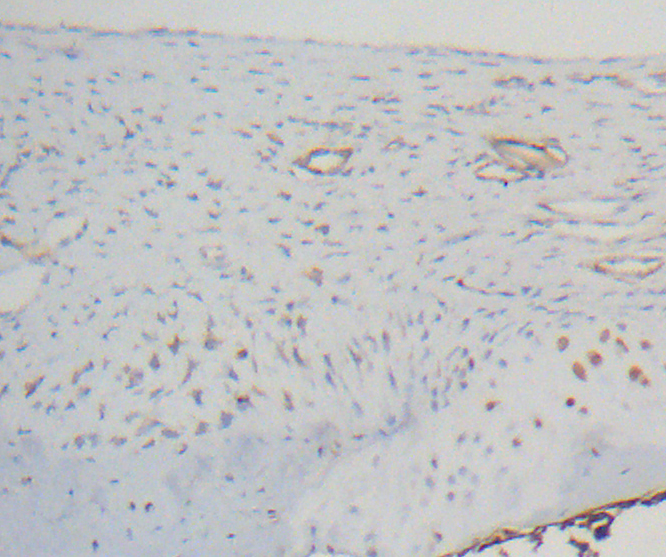

Supplement: Supplementary file 8 [file DataSheet_7.zip › ACSL4/miR(-) Exo/5.jpg]

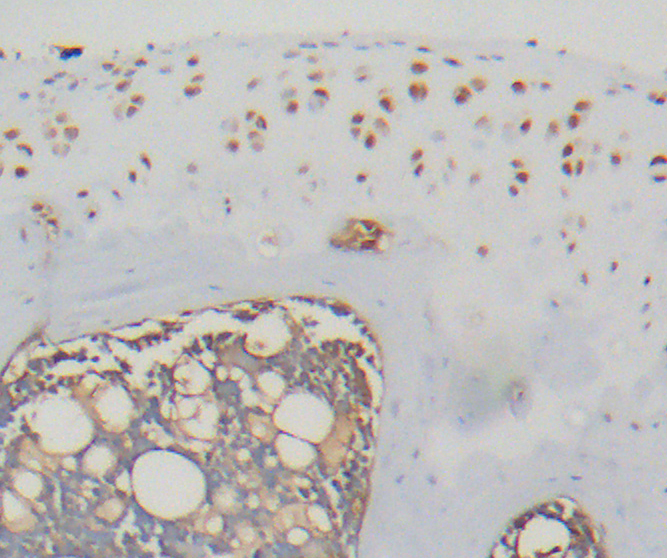

Supplement: Supplementary file 8 [file DataSheet_7.zip › ACSL4/miR(-) Exo/6.jpg]
