# Supplementary figures and images for: Exosomes from osteoarthritic fibroblast-like synoviocytes promote cartilage ferroptosis and damage via delivering microRNA-19b-3p to target SLC7A11 in osteoarthritis (part 5 of 6)
Source: Front Immunol. 2023 Aug 24;14:1181156. doi: 10.3389/fimmu.2023.1181156 (PMC10484587; doi:10.3389/fimmu.2023.1181156)

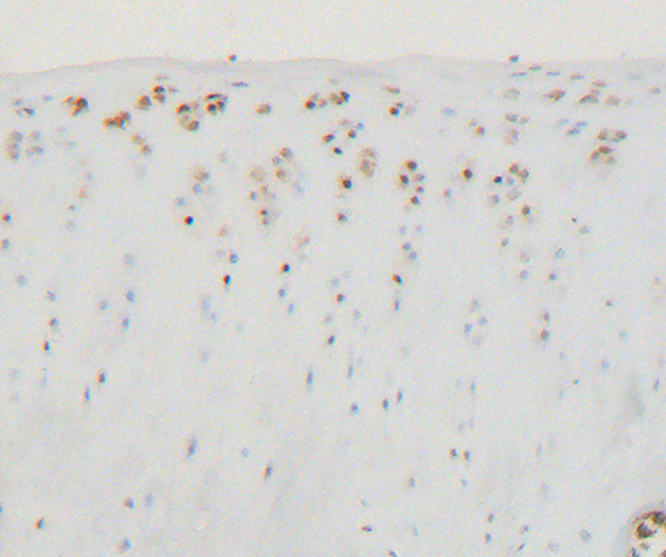

Supplement: Supplementary file 8 [file DataSheet_7.zip › ACSL4/Model/1.jpg]

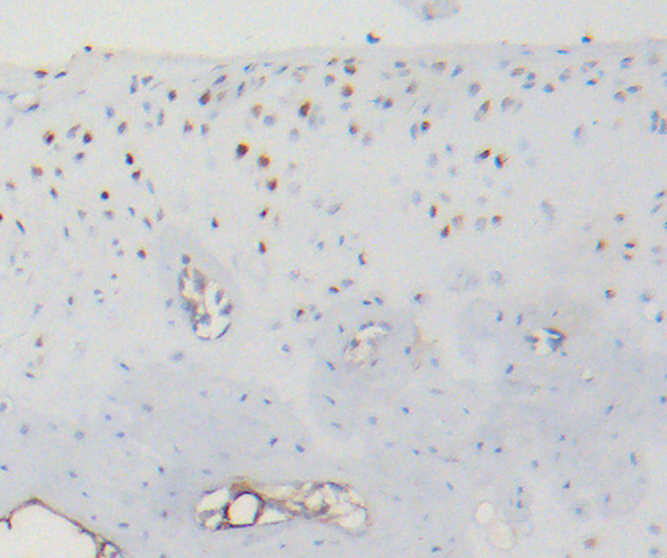

Supplement: Supplementary file 8 [file DataSheet_7.zip › ACSL4/Model/2.jpg]

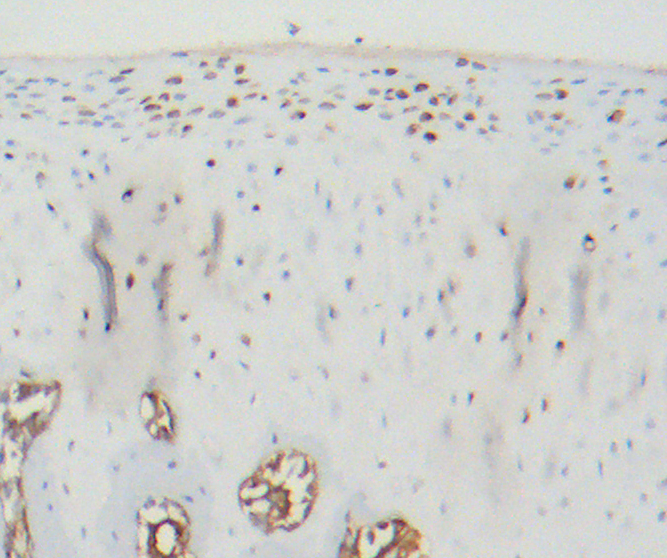

Supplement: Supplementary file 8 [file DataSheet_7.zip › ACSL4/Model/3.jpg]

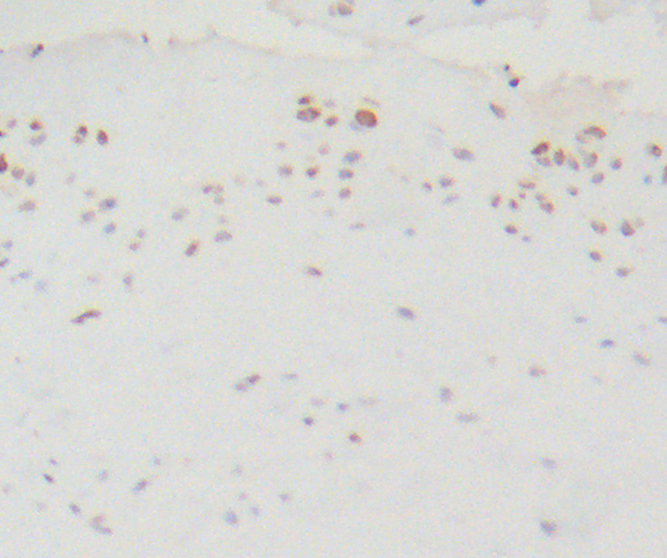

Supplement: Supplementary file 8 [file DataSheet_7.zip › ACSL4/Model/4.jpg]

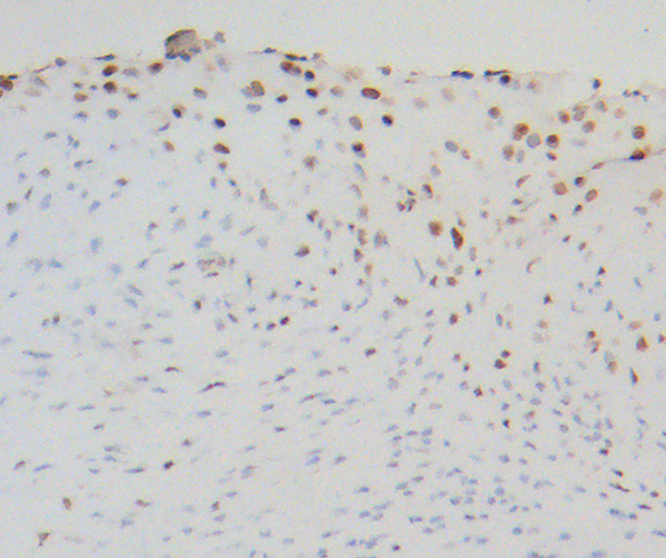

Supplement: Supplementary file 8 [file DataSheet_7.zip › ACSL4/Model/5.jpg]

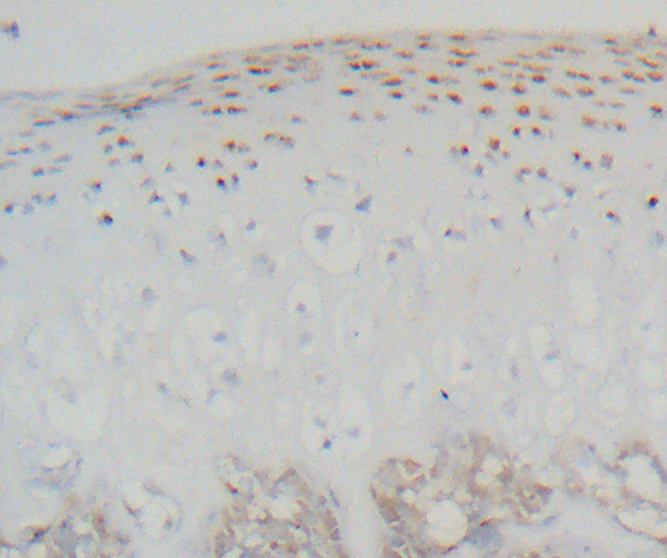

Supplement: Supplementary file 8 [file DataSheet_7.zip › ACSL4/Model/6.jpg]

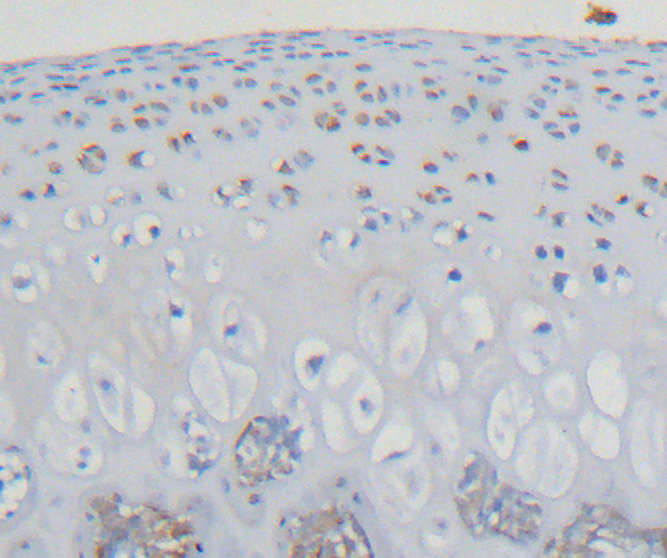

Supplement: Supplementary file 8 [file DataSheet_7.zip › ACSL4/Sham/1.jpg]

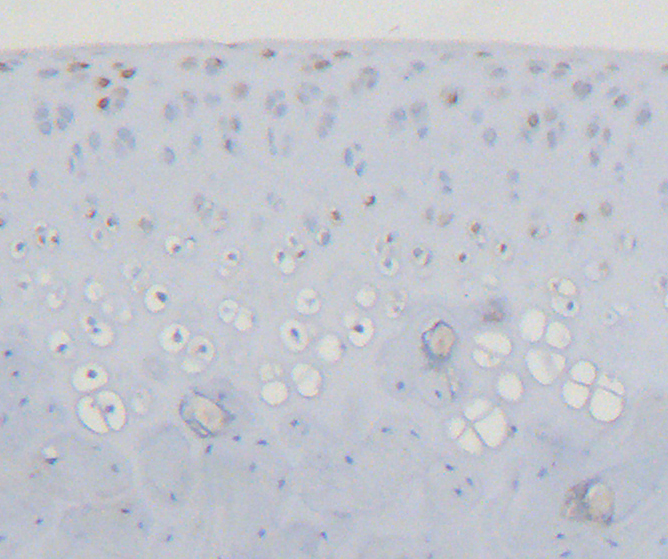

Supplement: Supplementary file 8 [file DataSheet_7.zip › ACSL4/Sham/2.jpg]

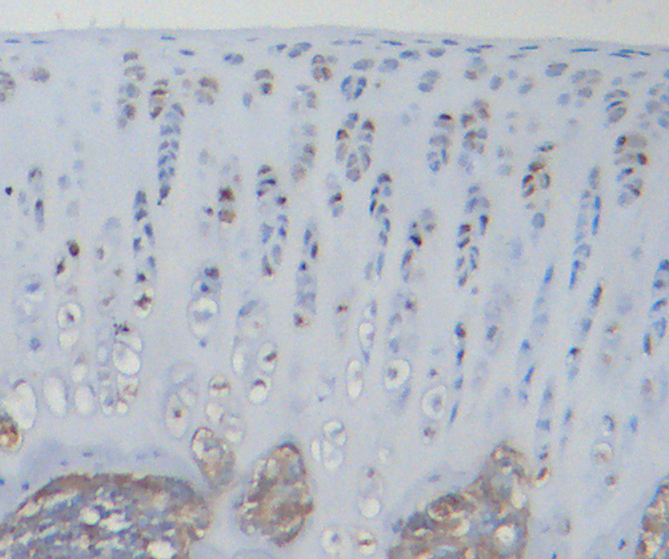

Supplement: Supplementary file 8 [file DataSheet_7.zip › ACSL4/Sham/3.jpg]

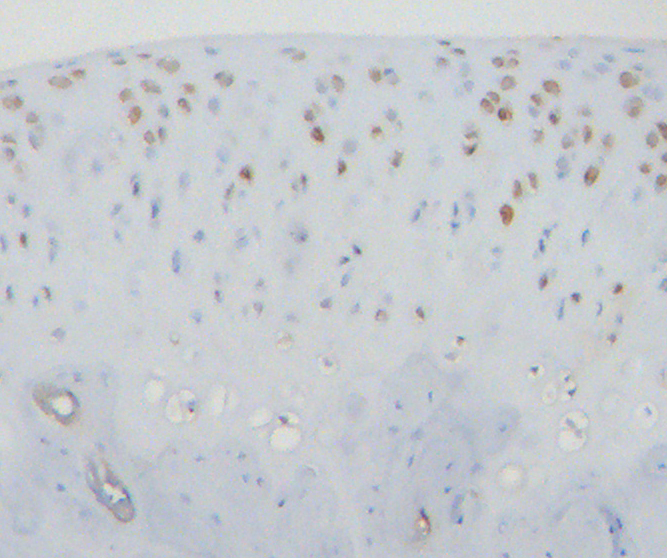

Supplement: Supplementary file 8 [file DataSheet_7.zip › ACSL4/Sham/4.jpg]

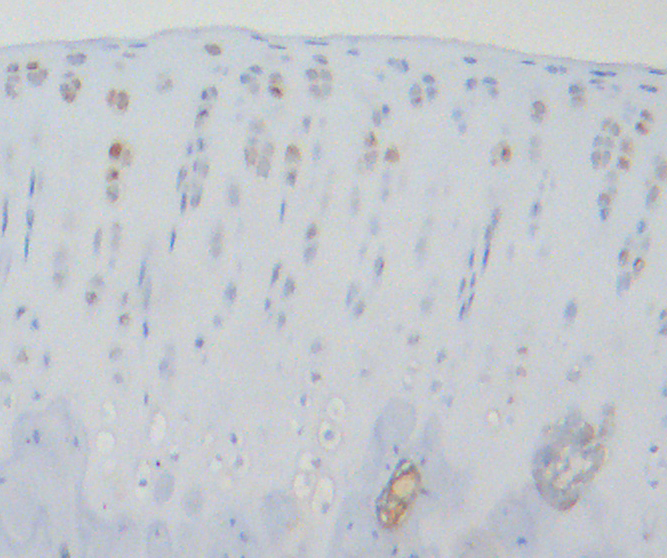

Supplement: Supplementary file 8 [file DataSheet_7.zip › ACSL4/Sham/5.jpg]

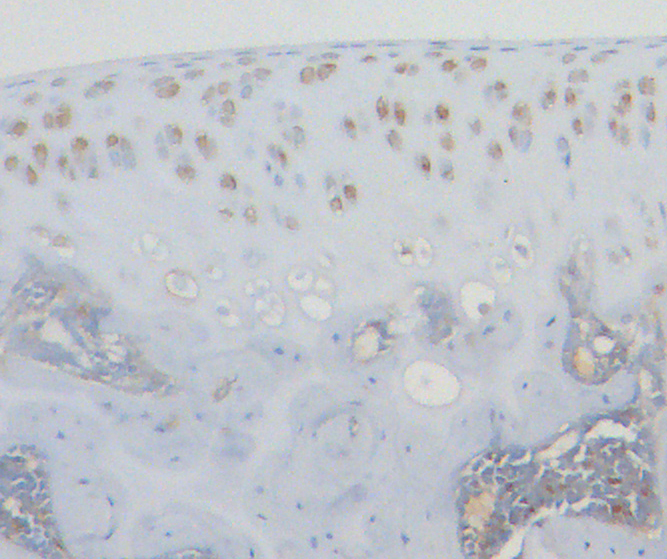

Supplement: Supplementary file 8 [file DataSheet_7.zip › ACSL4/Sham/6.jpg]

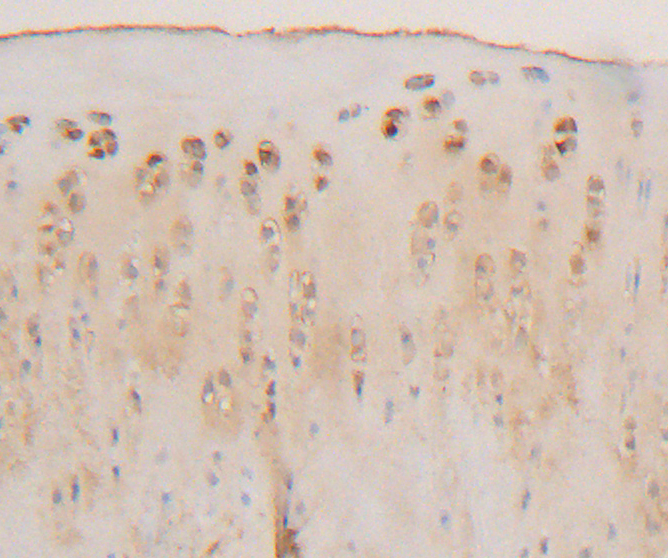

Supplement: Supplementary file 8 [file DataSheet_7.zip › COL2/Exo/1.jpg]

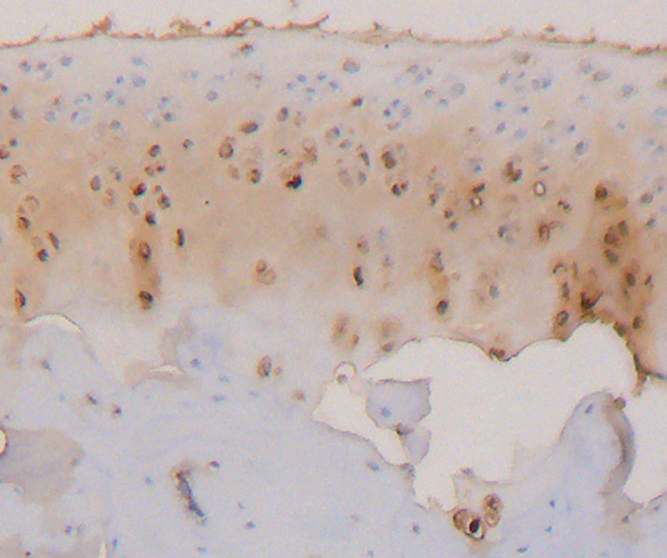

Supplement: Supplementary file 8 [file DataSheet_7.zip › COL2/Exo/2.jpg]

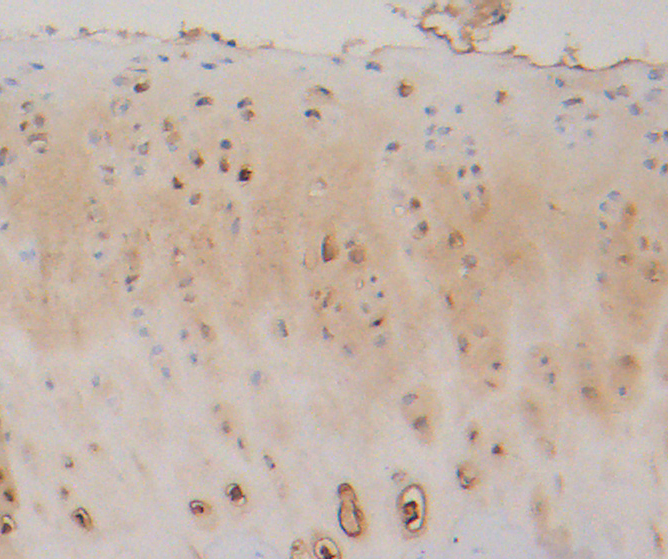

Supplement: Supplementary file 8 [file DataSheet_7.zip › COL2/Exo/3.jpg]

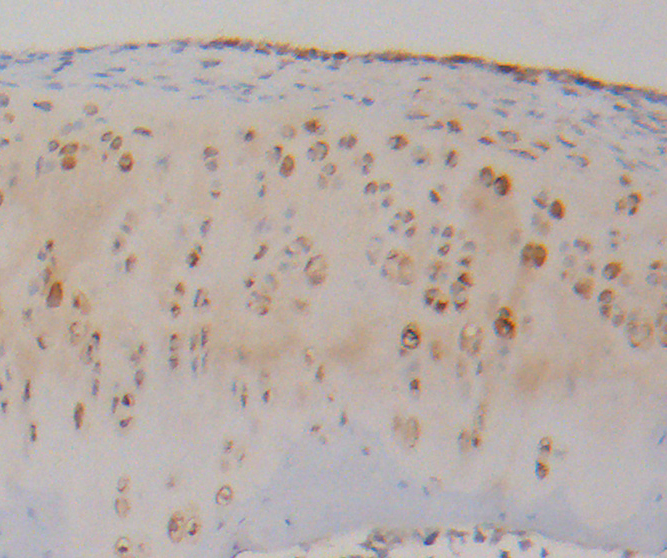

Supplement: Supplementary file 8 [file DataSheet_7.zip › COL2/Exo/4.jpg]

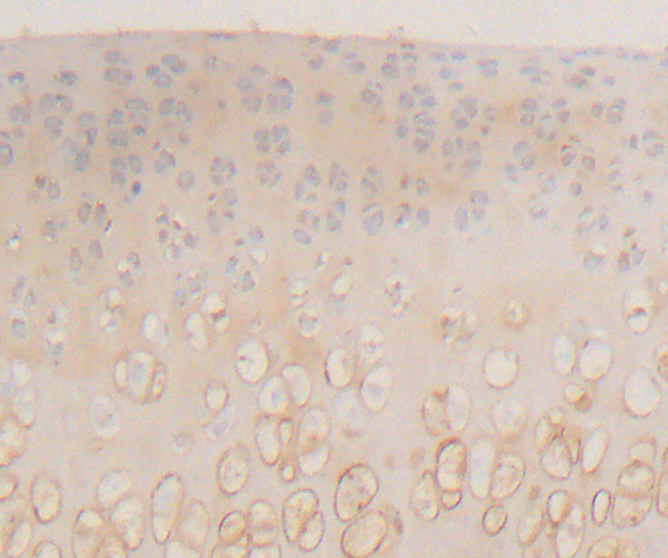

Supplement: Supplementary file 8 [file DataSheet_7.zip › COL2/Exo/5.jpg]

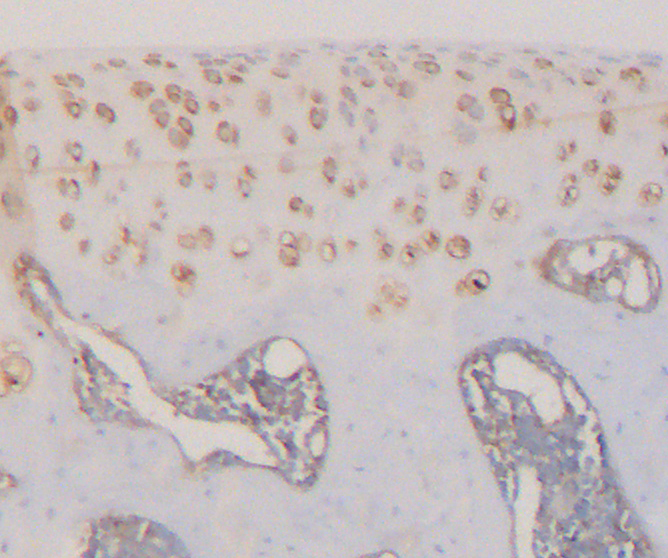

Supplement: Supplementary file 8 [file DataSheet_7.zip › COL2/Exo/6.jpg]

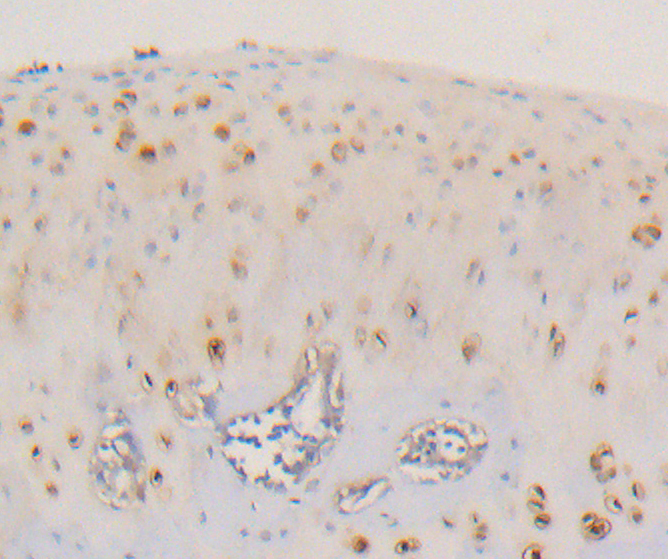

Supplement: Supplementary file 8 [file DataSheet_7.zip › COL2/Exo+Fer-1/1.jpg]

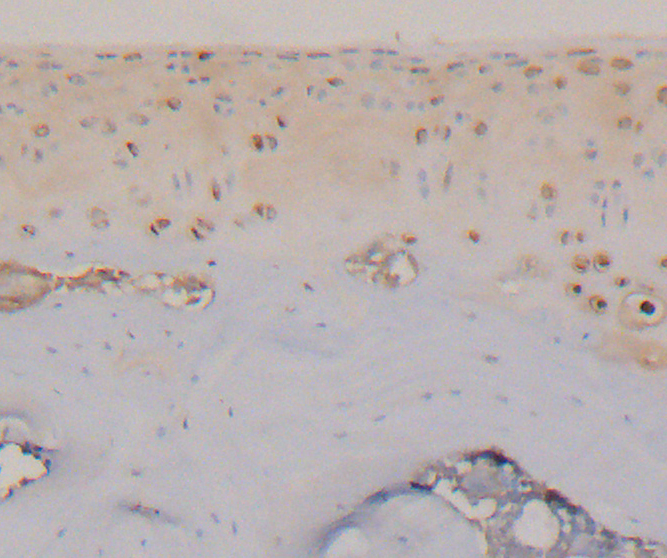

Supplement: Supplementary file 8 [file DataSheet_7.zip › COL2/Exo+Fer-1/2.jpg]

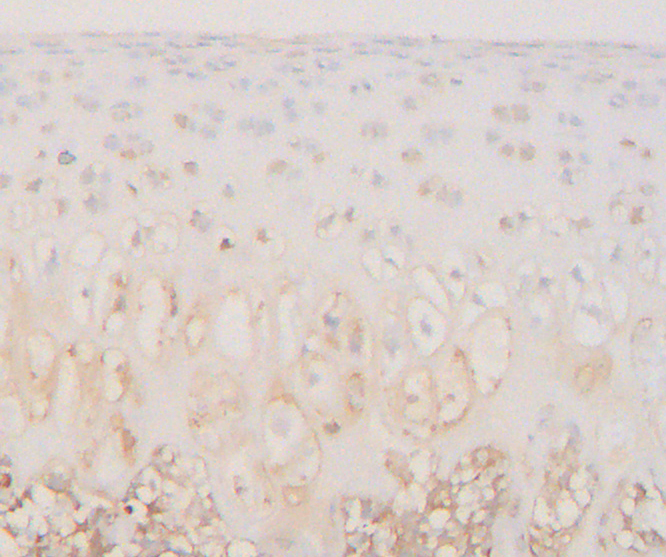

Supplement: Supplementary file 8 [file DataSheet_7.zip › COL2/Exo+Fer-1/3.jpg]

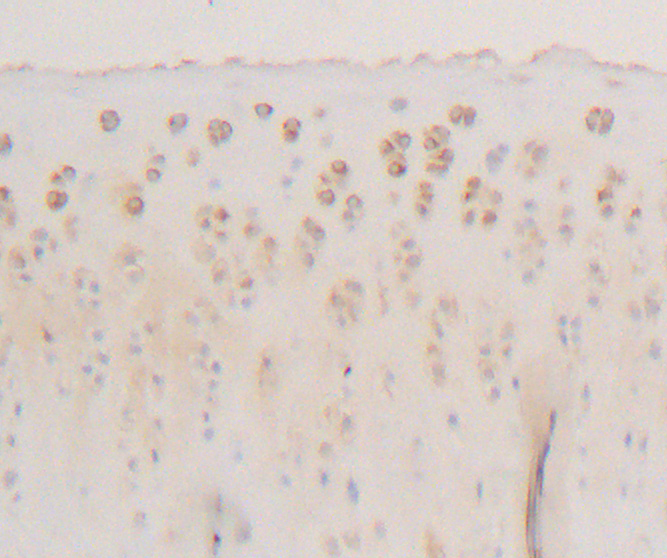

Supplement: Supplementary file 8 [file DataSheet_7.zip › COL2/Exo+Fer-1/4.jpg]

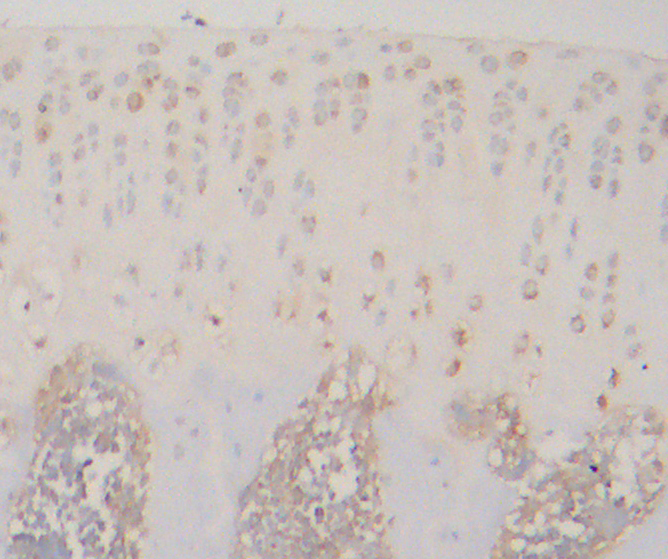

Supplement: Supplementary file 8 [file DataSheet_7.zip › COL2/Exo+Fer-1/5.jpg]

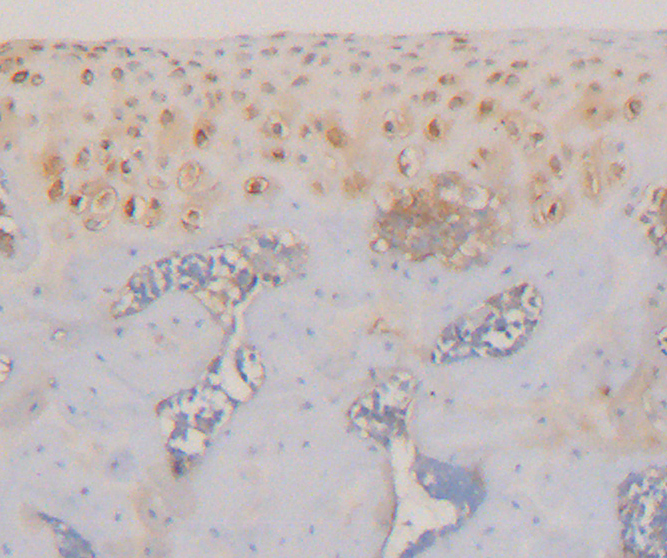

Supplement: Supplementary file 8 [file DataSheet_7.zip › COL2/Exo+Fer-1/6.jpg]

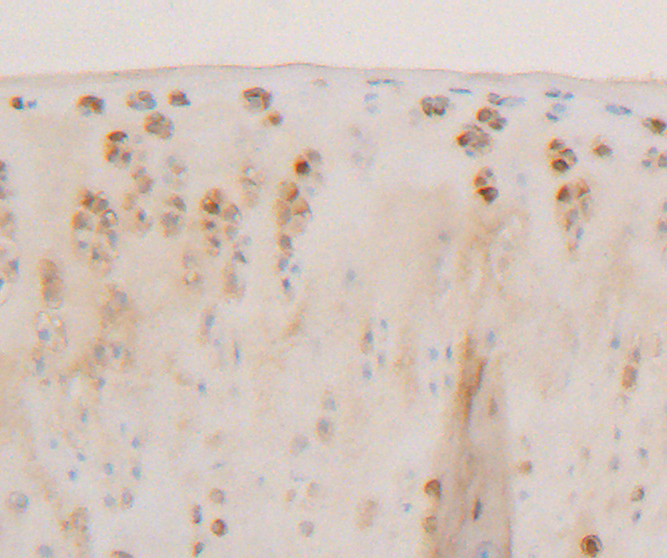

Supplement: Supplementary file 8 [file DataSheet_7.zip › COL2/miR(-) Exo/1.jpg]

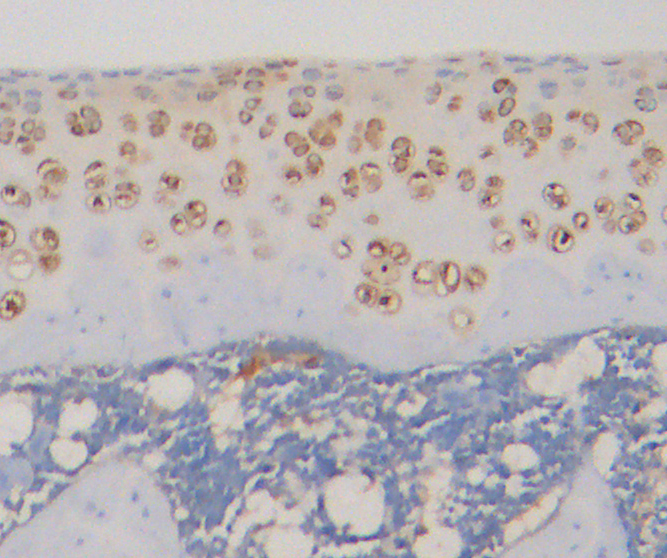

Supplement: Supplementary file 8 [file DataSheet_7.zip › COL2/miR(-) Exo/2.jpg]

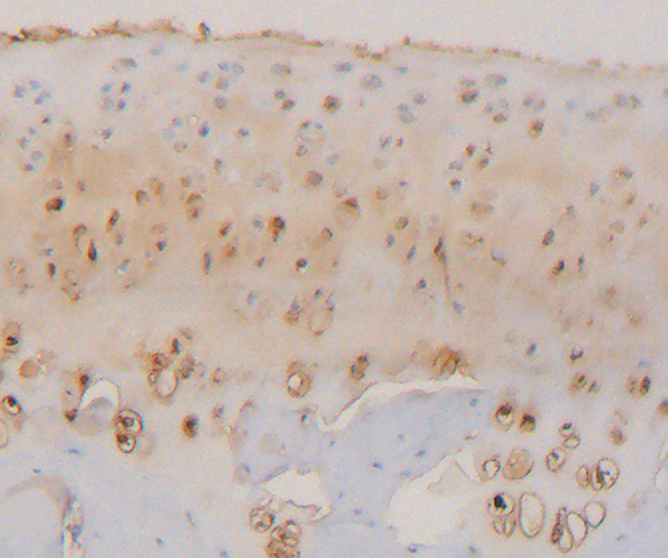

Supplement: Supplementary file 8 [file DataSheet_7.zip › COL2/miR(-) Exo/3.jpg]

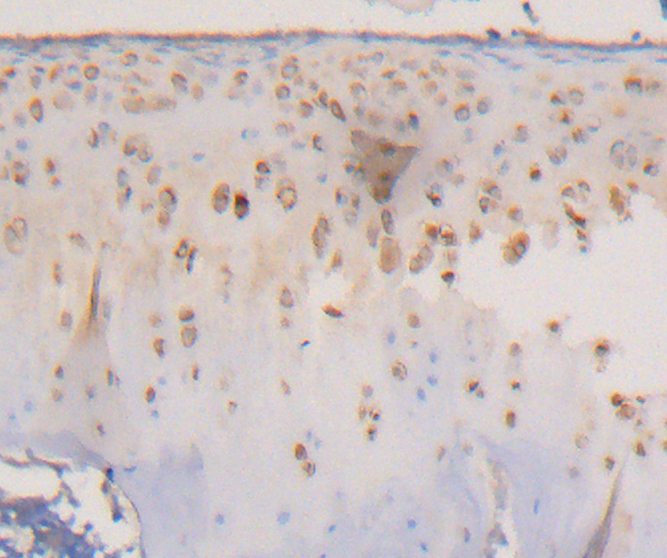

Supplement: Supplementary file 8 [file DataSheet_7.zip › COL2/miR(-) Exo/4.jpg]

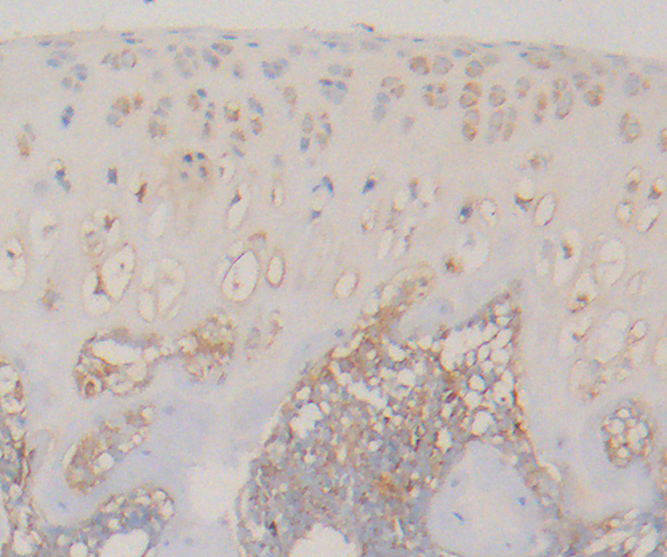

Supplement: Supplementary file 8 [file DataSheet_7.zip › COL2/miR(-) Exo/5.jpg]

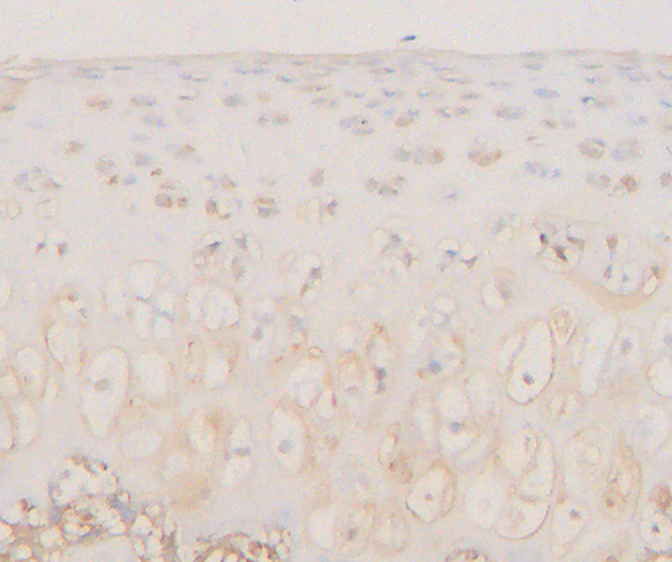

Supplement: Supplementary file 8 [file DataSheet_7.zip › COL2/miR(-) Exo/6.jpg]

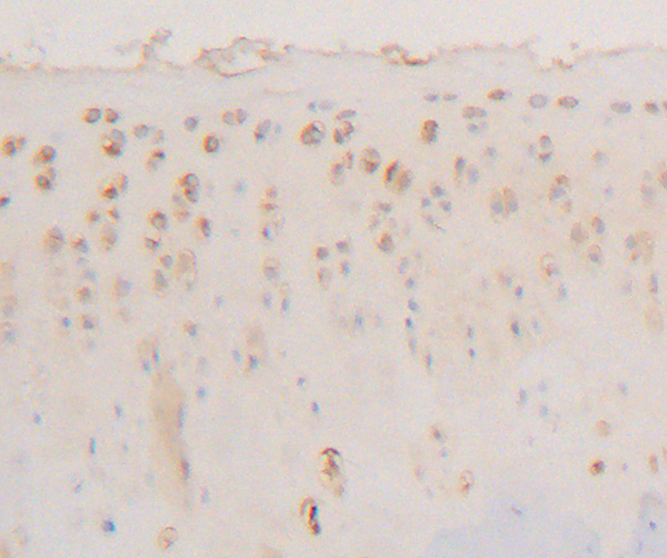

Supplement: Supplementary file 8 [file DataSheet_7.zip › COL2/Model/1.jpg]

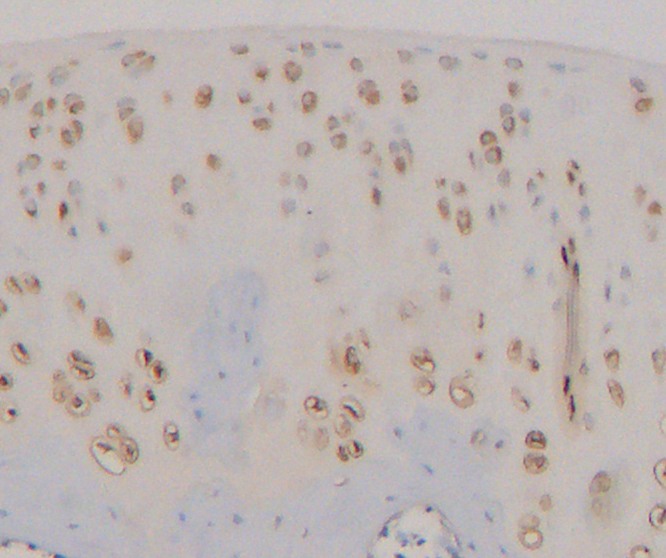

Supplement: Supplementary file 8 [file DataSheet_7.zip › COL2/Model/2.jpg]

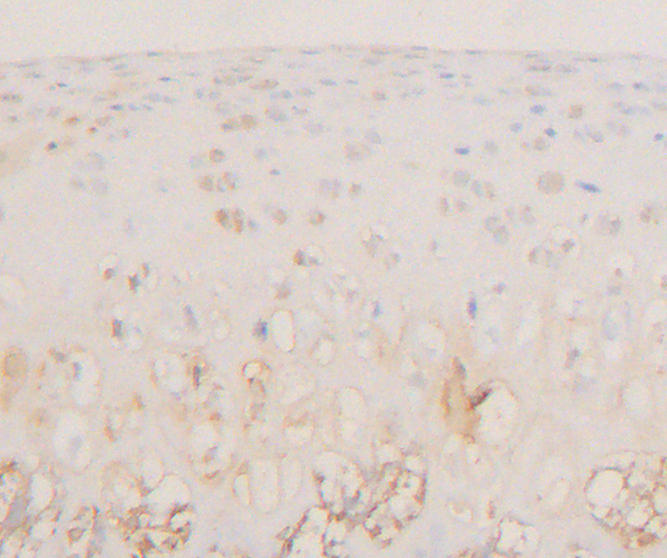

Supplement: Supplementary file 8 [file DataSheet_7.zip › COL2/Model/3.jpg]

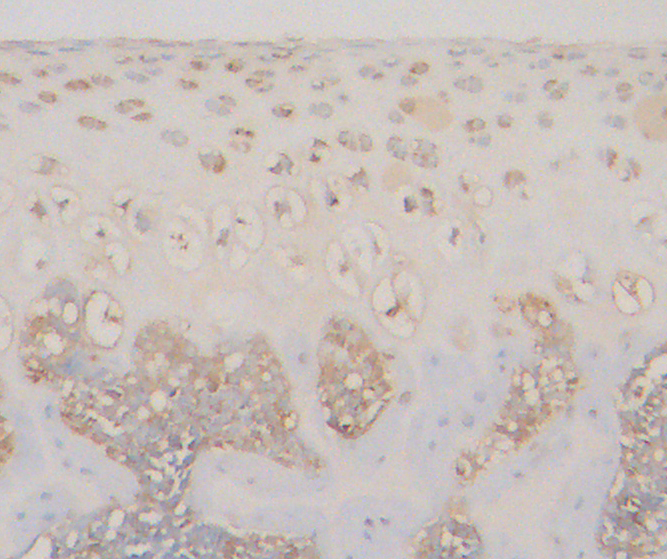

Supplement: Supplementary file 8 [file DataSheet_7.zip › COL2/Model/4.jpg]

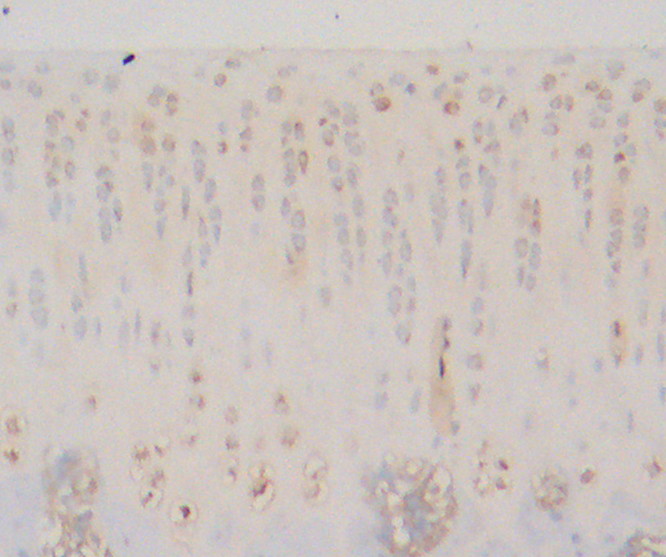

Supplement: Supplementary file 8 [file DataSheet_7.zip › COL2/Model/5.jpg]

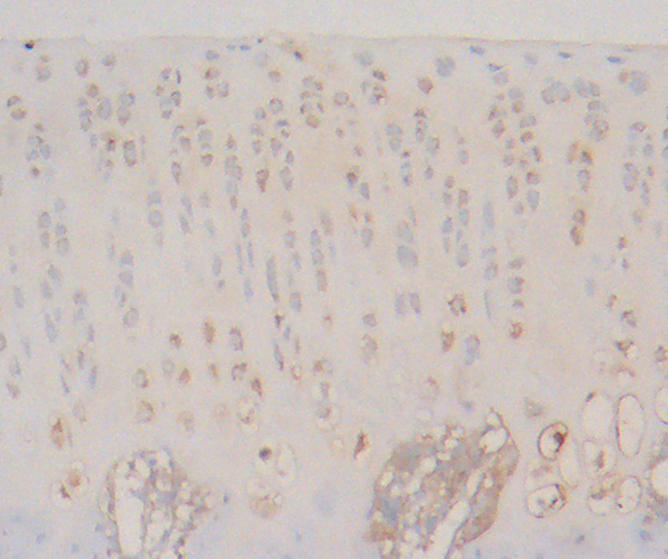

Supplement: Supplementary file 8 [file DataSheet_7.zip › COL2/Model/6.jpg]

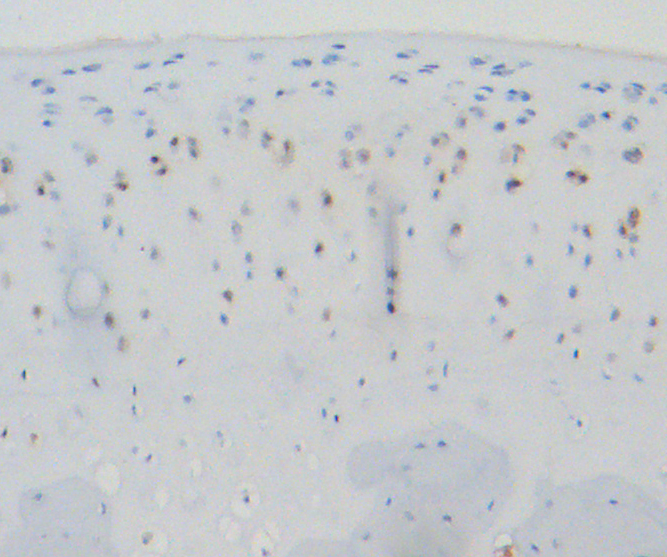

Supplement: Supplementary file 8 [file DataSheet_7.zip › COL2/Sham/1.jpg]

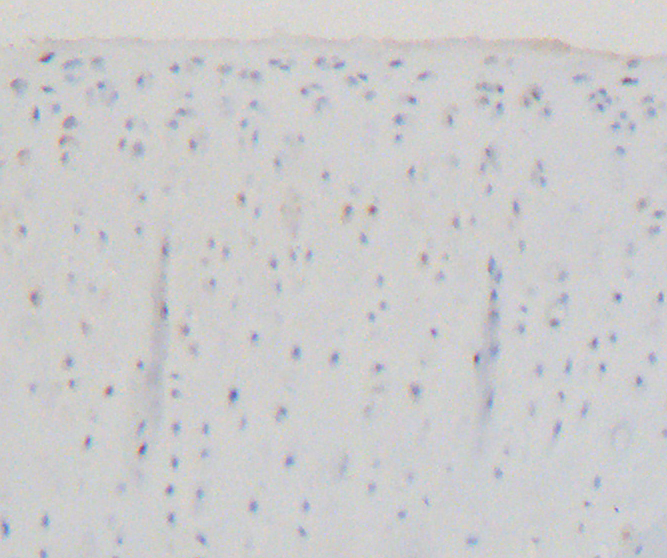

Supplement: Supplementary file 8 [file DataSheet_7.zip › COL2/Sham/2.jpg]

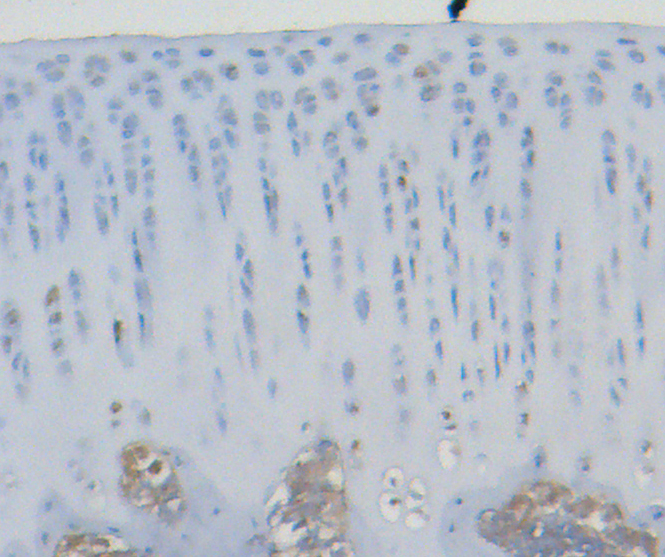

Supplement: Supplementary file 8 [file DataSheet_7.zip › COL2/Sham/3.jpg]

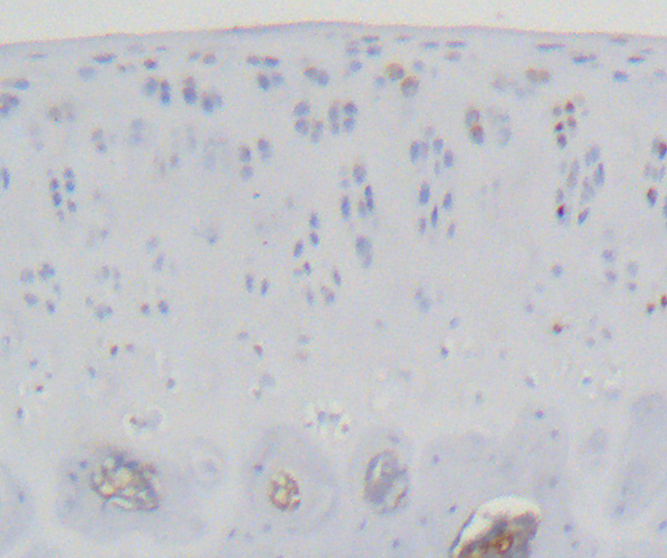

Supplement: Supplementary file 8 [file DataSheet_7.zip › COL2/Sham/4.jpg]

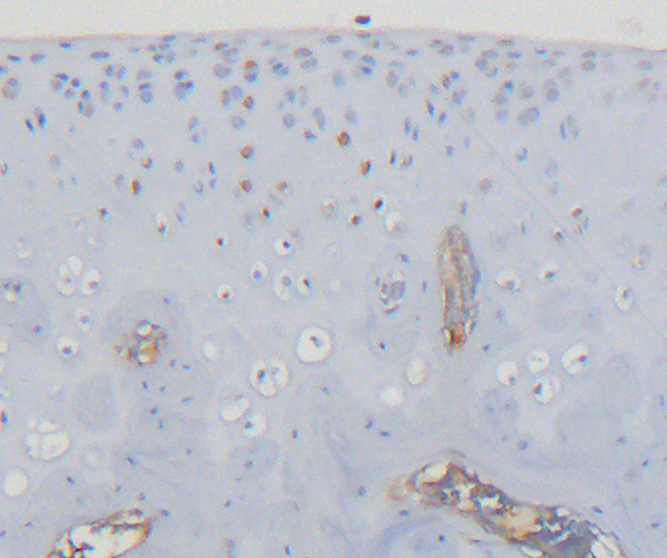

Supplement: Supplementary file 8 [file DataSheet_7.zip › COL2/Sham/5.jpg]

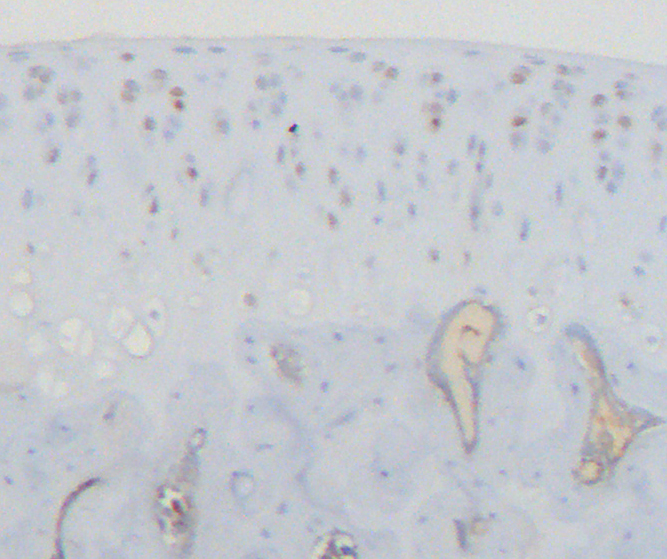

Supplement: Supplementary file 8 [file DataSheet_7.zip › COL2/Sham/6.jpg]

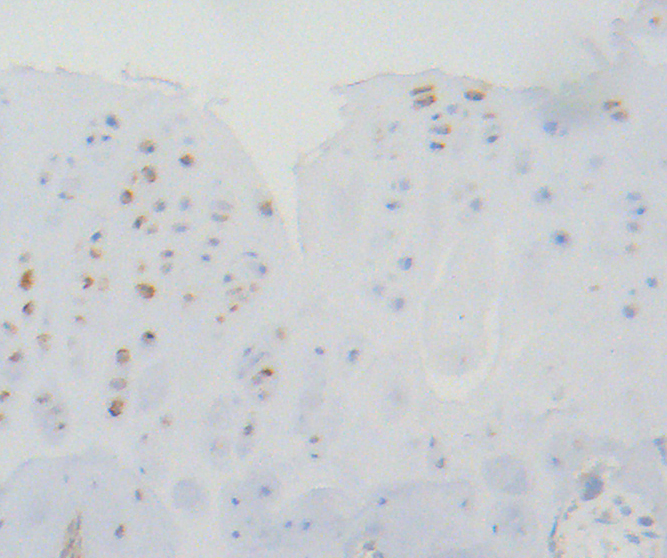

Supplement: Supplementary file 9 [file DataSheet_8.zip › GPX4/Exo/1.jpg]

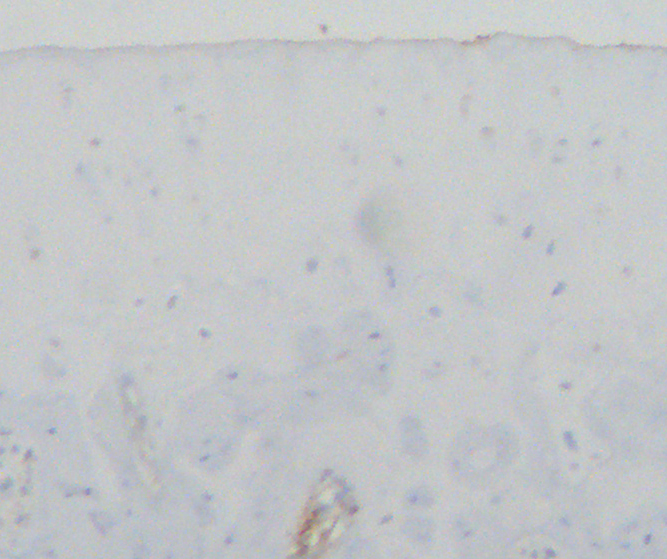

Supplement: Supplementary file 9 [file DataSheet_8.zip › GPX4/Exo/2.jpg]

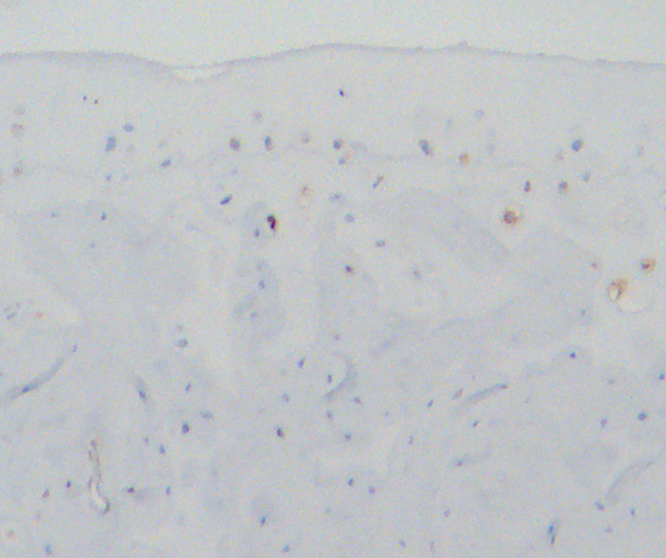

Supplement: Supplementary file 9 [file DataSheet_8.zip › GPX4/Exo/3.jpg]

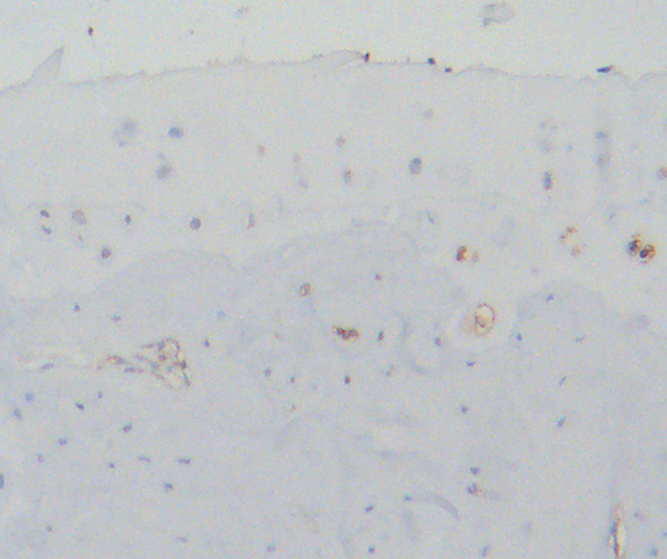

Supplement: Supplementary file 9 [file DataSheet_8.zip › GPX4/Exo/4.jpg]

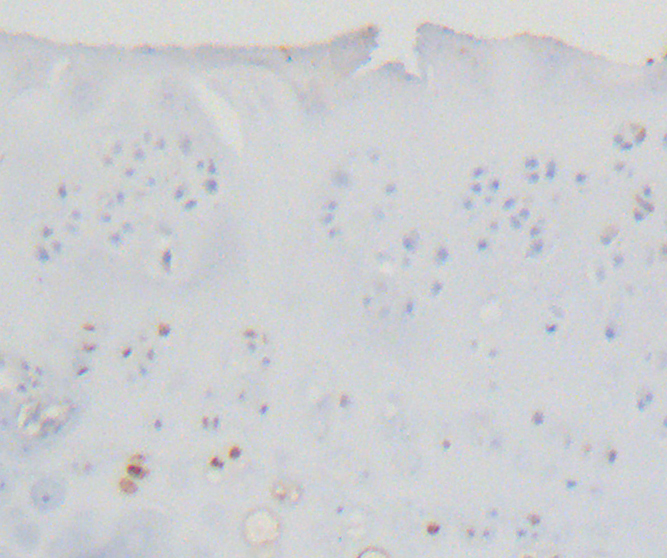

Supplement: Supplementary file 9 [file DataSheet_8.zip › GPX4/Exo/5.jpg]

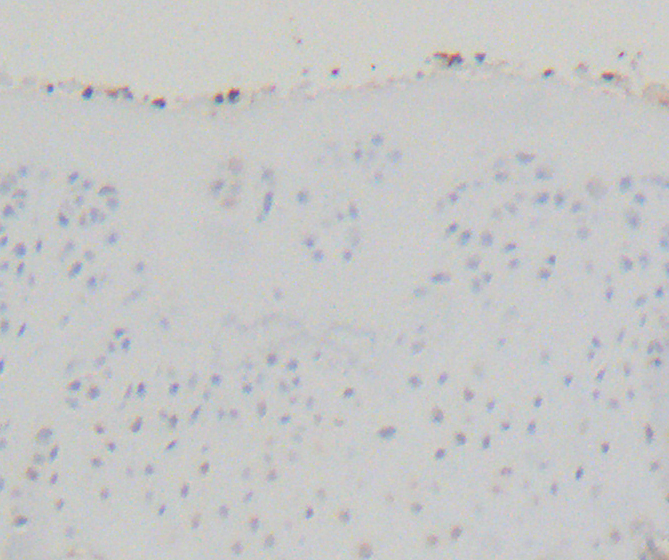

Supplement: Supplementary file 9 [file DataSheet_8.zip › GPX4/Exo/6.jpg]

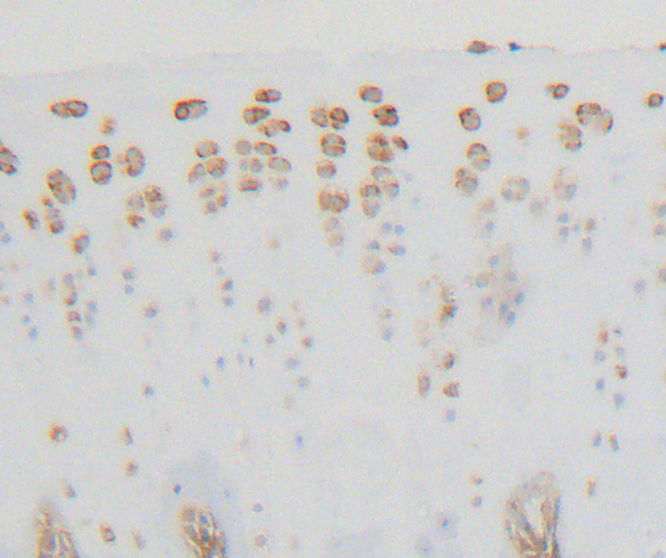

Supplement: Supplementary file 9 [file DataSheet_8.zip › GPX4/Exo+Fer-1/1.jpg]

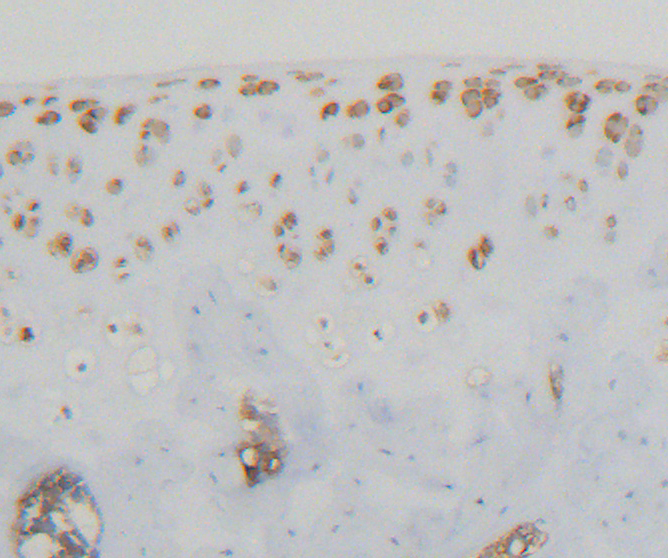

Supplement: Supplementary file 9 [file DataSheet_8.zip › GPX4/Exo+Fer-1/2.jpg]

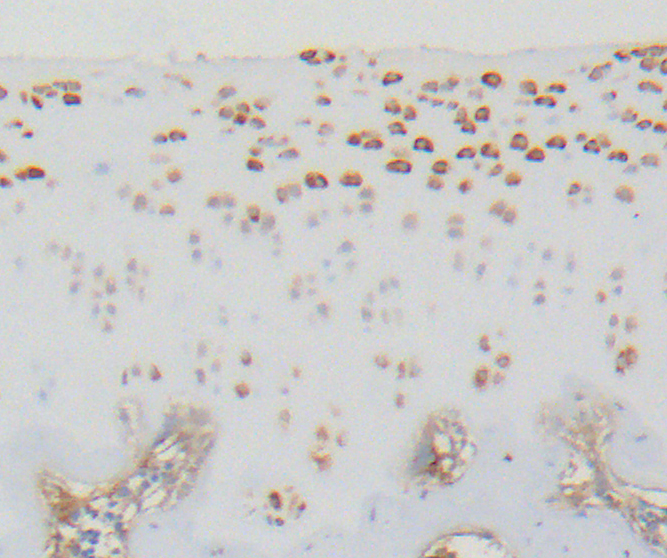

Supplement: Supplementary file 9 [file DataSheet_8.zip › GPX4/Exo+Fer-1/3.jpg]

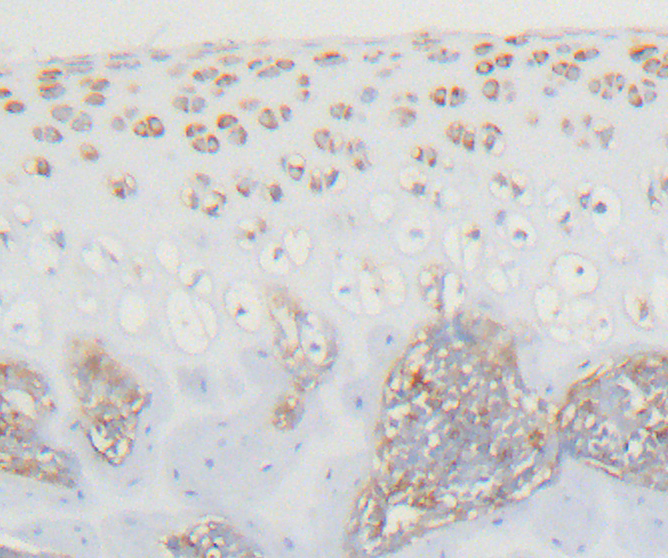

Supplement: Supplementary file 9 [file DataSheet_8.zip › GPX4/Exo+Fer-1/4.jpg]

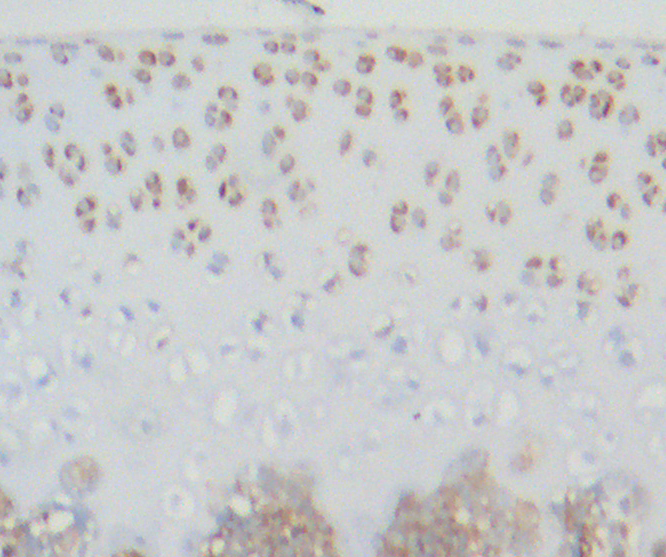

Supplement: Supplementary file 9 [file DataSheet_8.zip › GPX4/Exo+Fer-1/5.jpg]

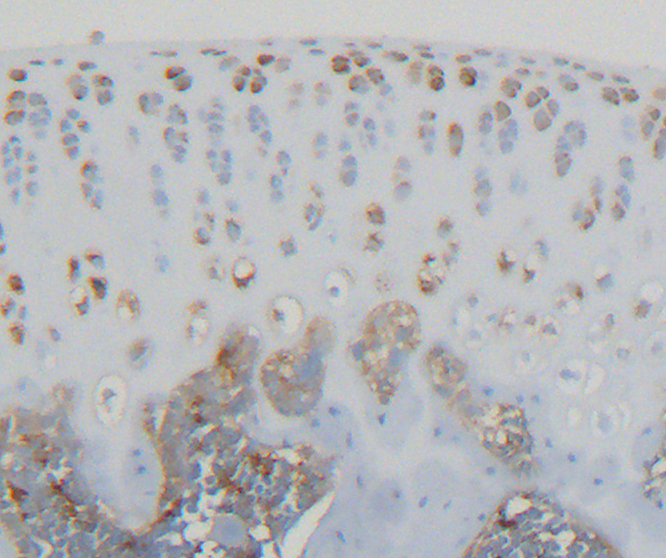

Supplement: Supplementary file 9 [file DataSheet_8.zip › GPX4/Exo+Fer-1/6.jpg]

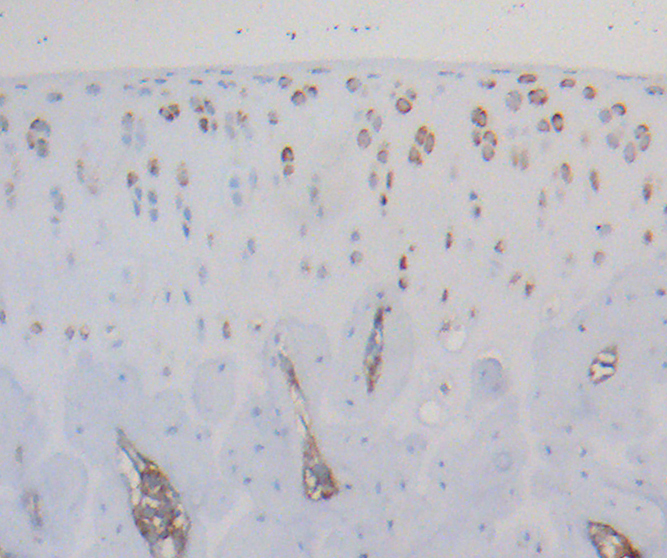

Supplement: Supplementary file 9 [file DataSheet_8.zip › GPX4/miR(-) Exo/1.jpg]

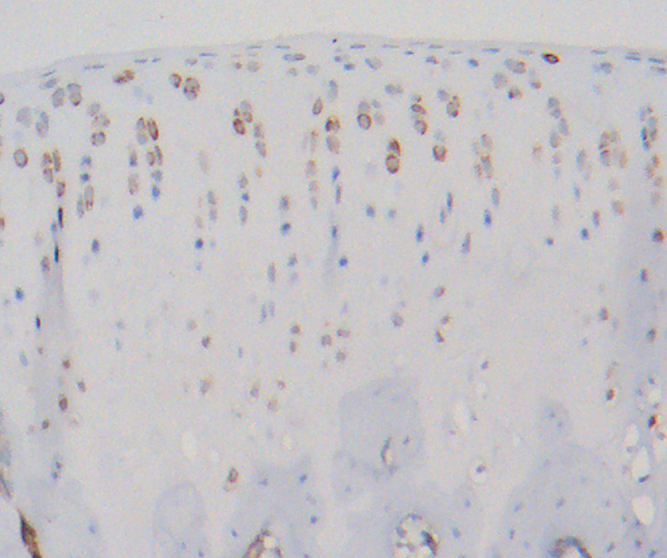

Supplement: Supplementary file 9 [file DataSheet_8.zip › GPX4/miR(-) Exo/2.jpg]

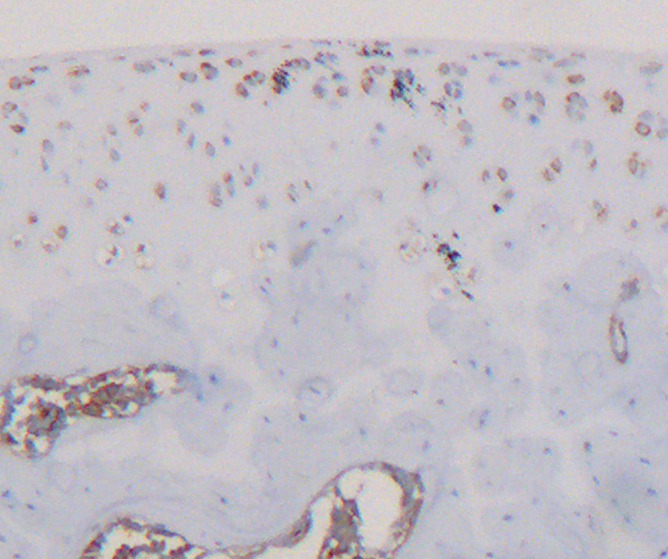

Supplement: Supplementary file 9 [file DataSheet_8.zip › GPX4/miR(-) Exo/3.jpg]

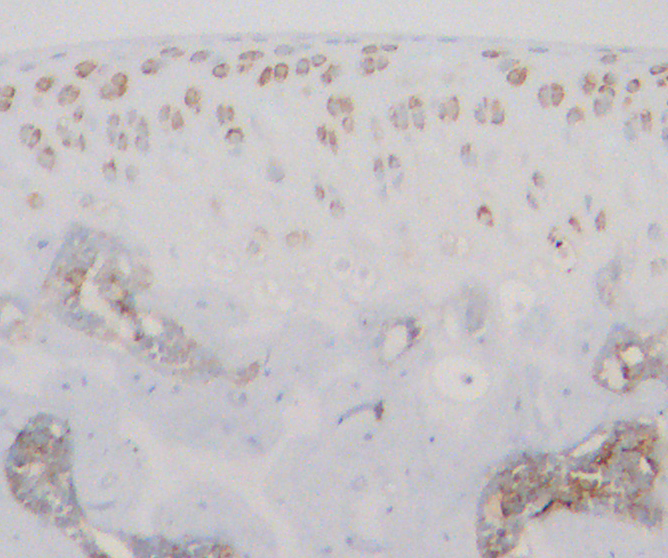

Supplement: Supplementary file 9 [file DataSheet_8.zip › GPX4/miR(-) Exo/4.jpg]

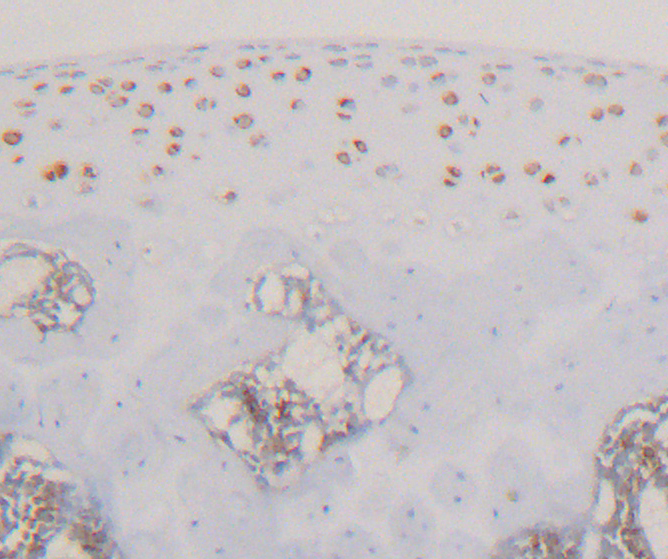

Supplement: Supplementary file 9 [file DataSheet_8.zip › GPX4/miR(-) Exo/5.jpg]

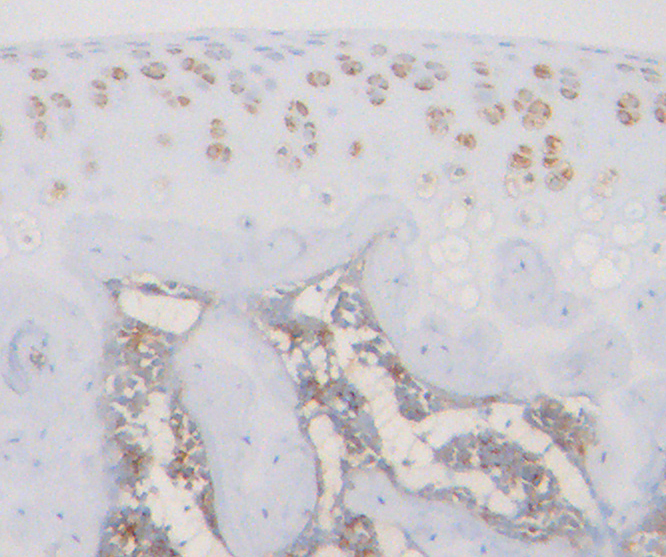

Supplement: Supplementary file 9 [file DataSheet_8.zip › GPX4/miR(-) Exo/6.jpg]

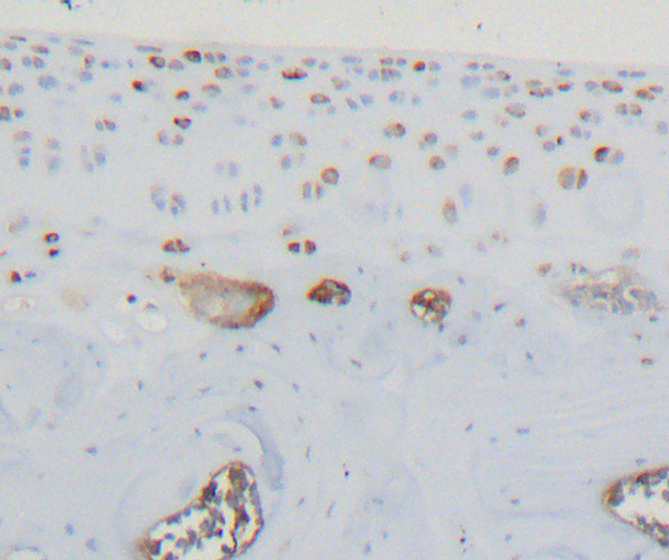

Supplement: Supplementary file 9 [file DataSheet_8.zip › GPX4/Model/1.jpg]

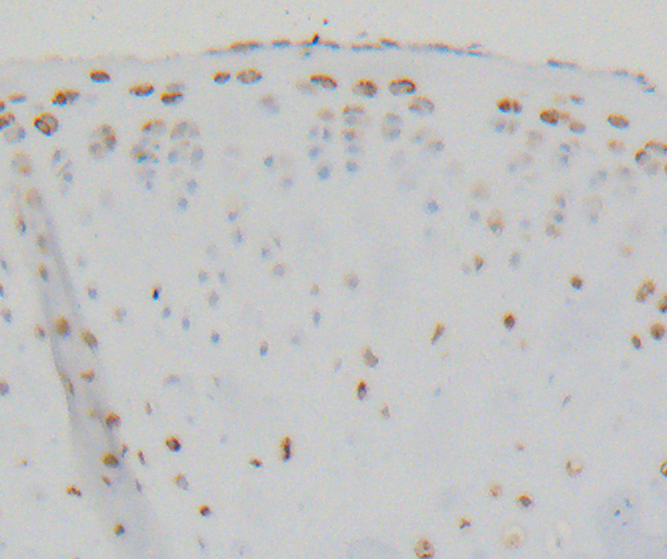

Supplement: Supplementary file 9 [file DataSheet_8.zip › GPX4/Model/2.jpg]

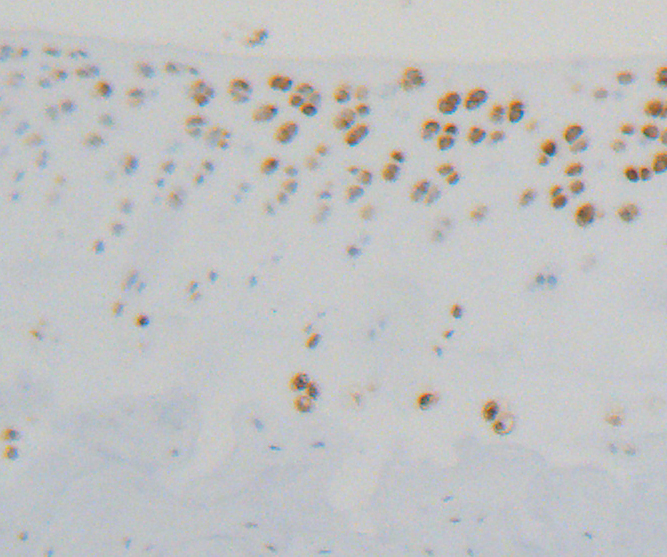

Supplement: Supplementary file 9 [file DataSheet_8.zip › GPX4/Model/3.jpg]

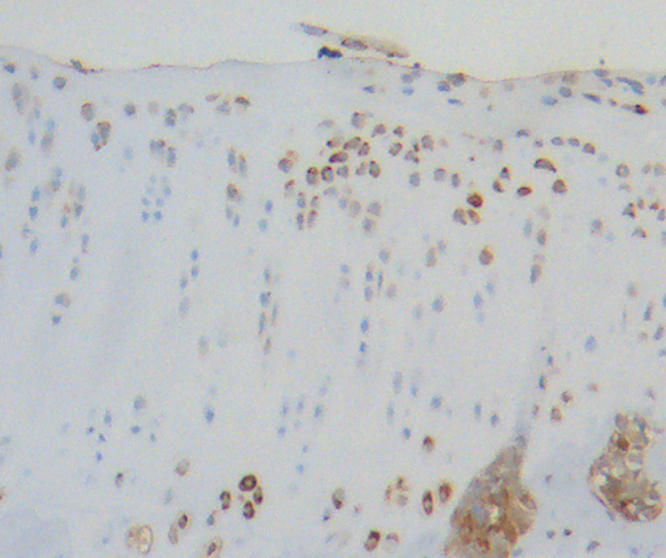

Supplement: Supplementary file 9 [file DataSheet_8.zip › GPX4/Model/4.jpg]

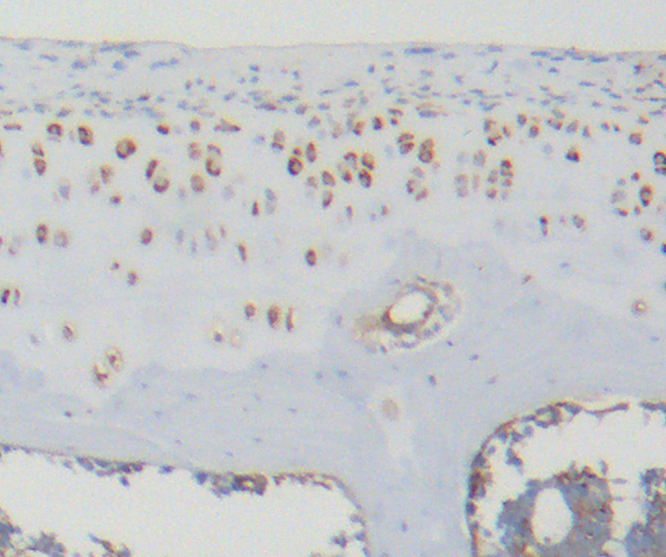

Supplement: Supplementary file 9 [file DataSheet_8.zip › GPX4/Model/5.jpg]

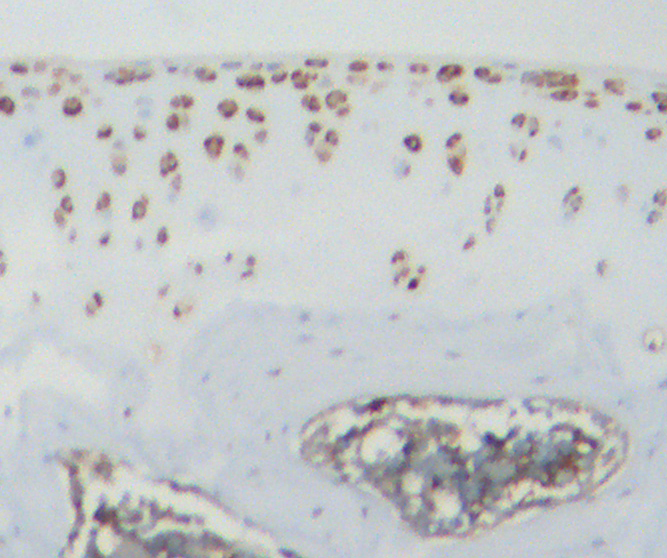

Supplement: Supplementary file 9 [file DataSheet_8.zip › GPX4/Model/6.jpg]

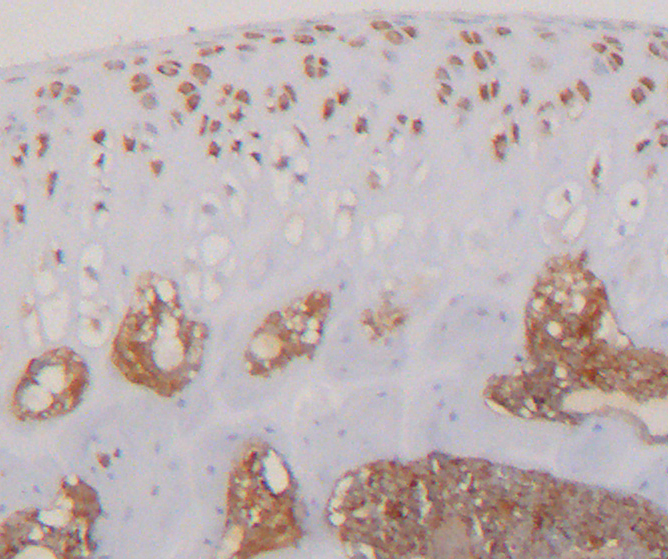

Supplement: Supplementary file 9 [file DataSheet_8.zip › GPX4/Sham/1.jpg]

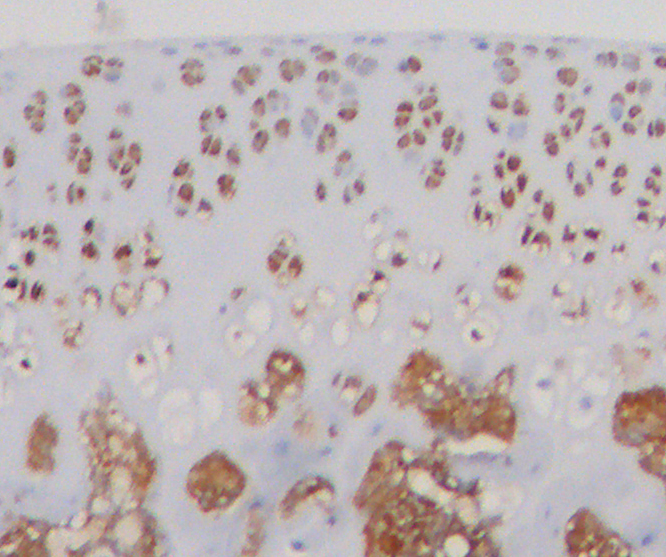

Supplement: Supplementary file 9 [file DataSheet_8.zip › GPX4/Sham/2.jpg]

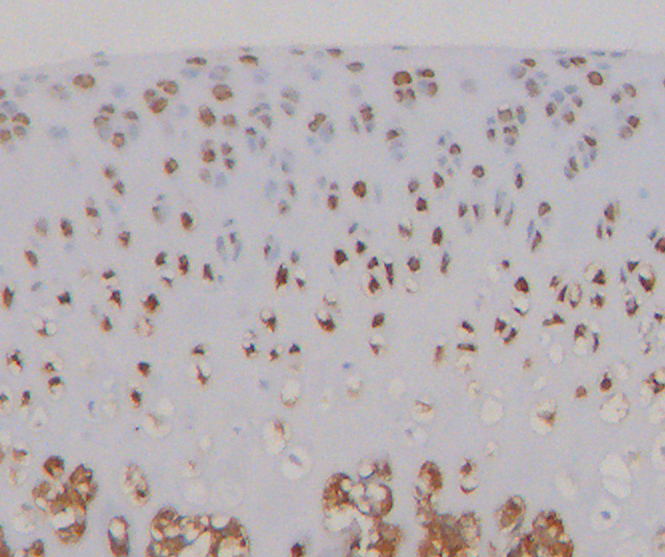

Supplement: Supplementary file 9 [file DataSheet_8.zip › GPX4/Sham/3.jpg]

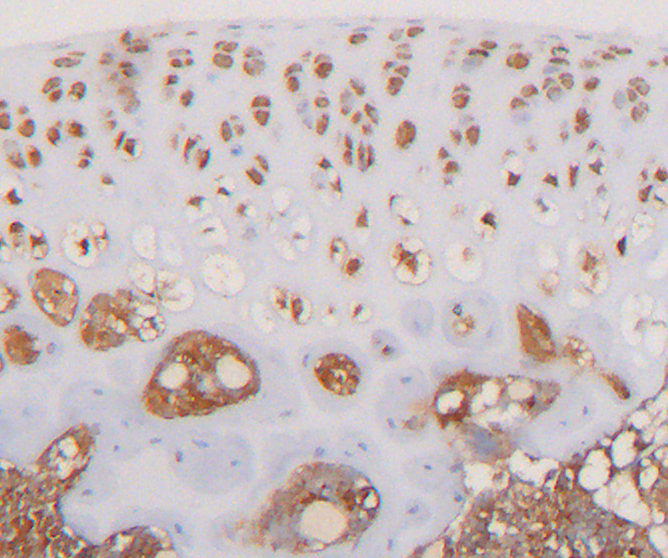

Supplement: Supplementary file 9 [file DataSheet_8.zip › GPX4/Sham/4.jpg]

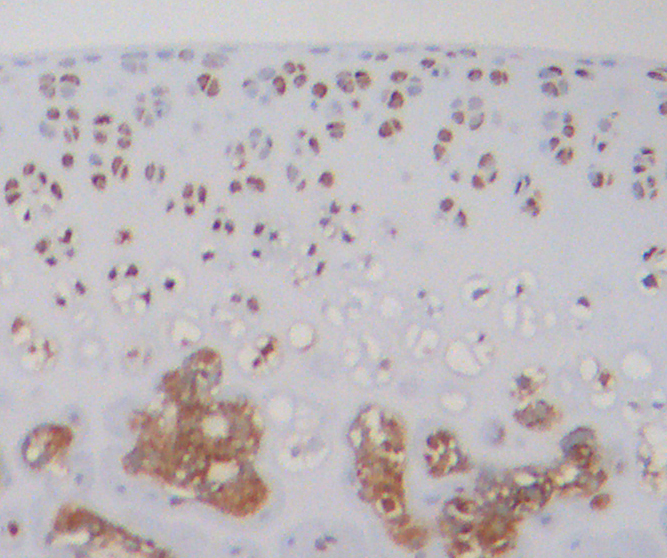

Supplement: Supplementary file 9 [file DataSheet_8.zip › GPX4/Sham/5.jpg]

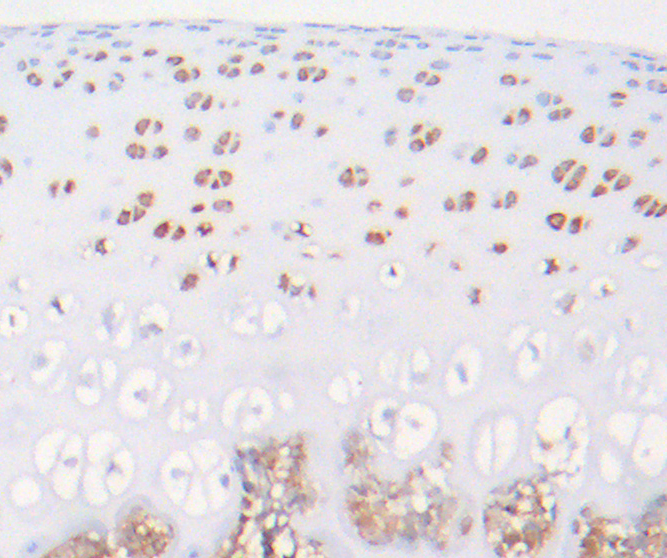

Supplement: Supplementary file 9 [file DataSheet_8.zip › GPX4/Sham/6.jpg]

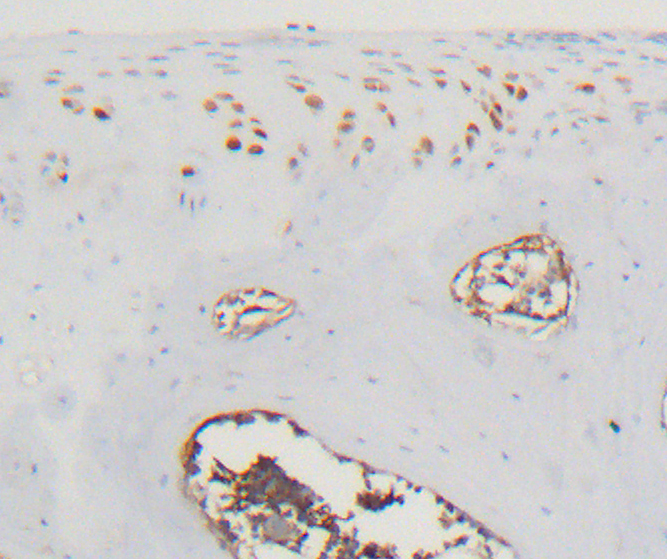

Supplement: Supplementary file 9 [file DataSheet_8.zip › MMP13/Exo/1.jpg]

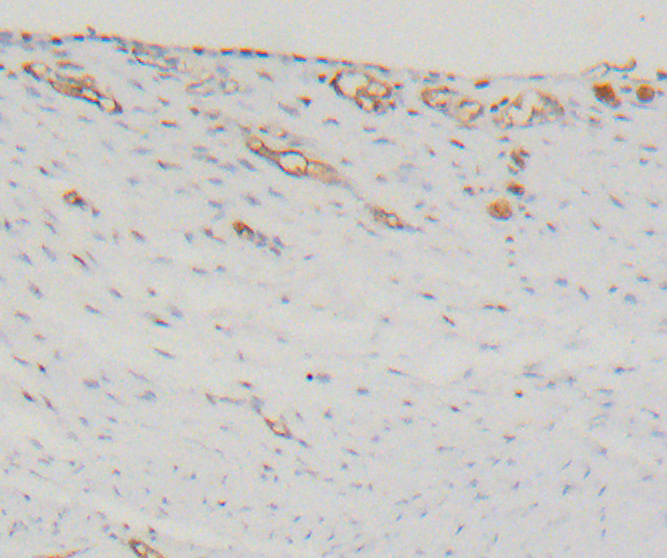

Supplement: Supplementary file 9 [file DataSheet_8.zip › MMP13/Exo/2.jpg]

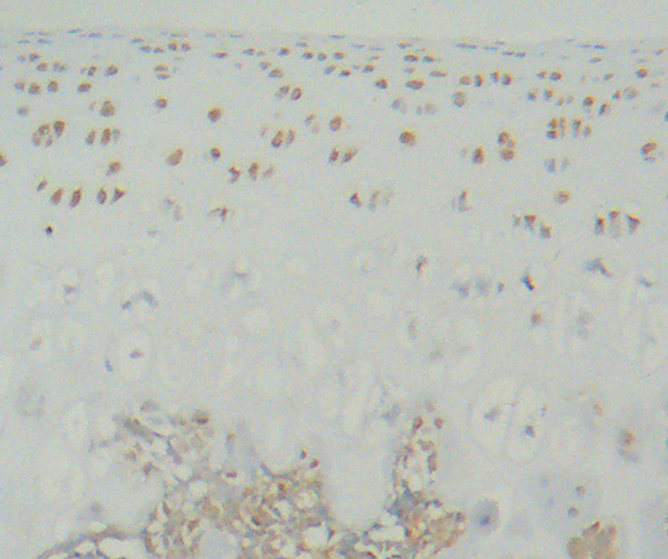

Supplement: Supplementary file 9 [file DataSheet_8.zip › MMP13/Exo/3.jpg]

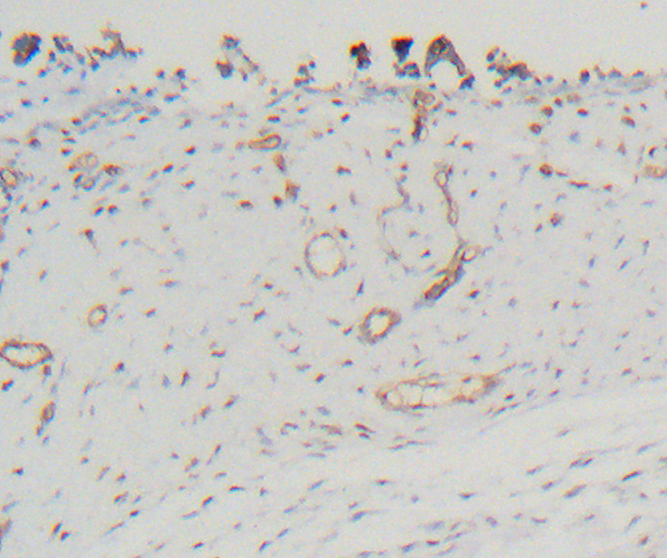

Supplement: Supplementary file 9 [file DataSheet_8.zip › MMP13/Exo/4.jpg]

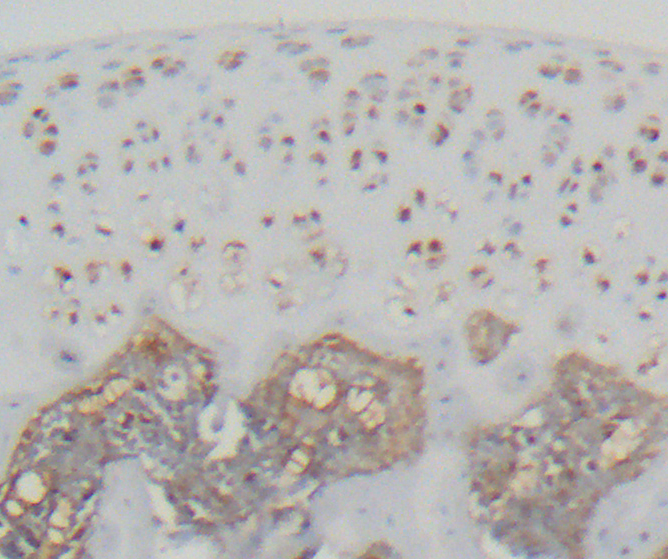

Supplement: Supplementary file 9 [file DataSheet_8.zip › MMP13/Exo/5.jpg]

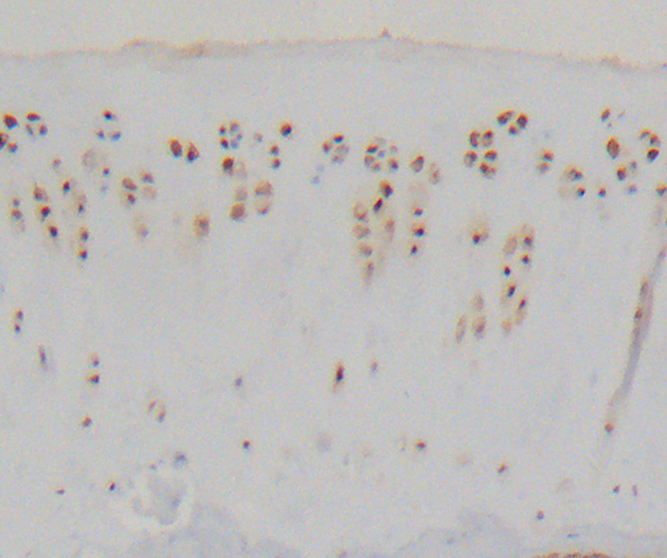

Supplement: Supplementary file 9 [file DataSheet_8.zip › MMP13/Exo/6.jpg]

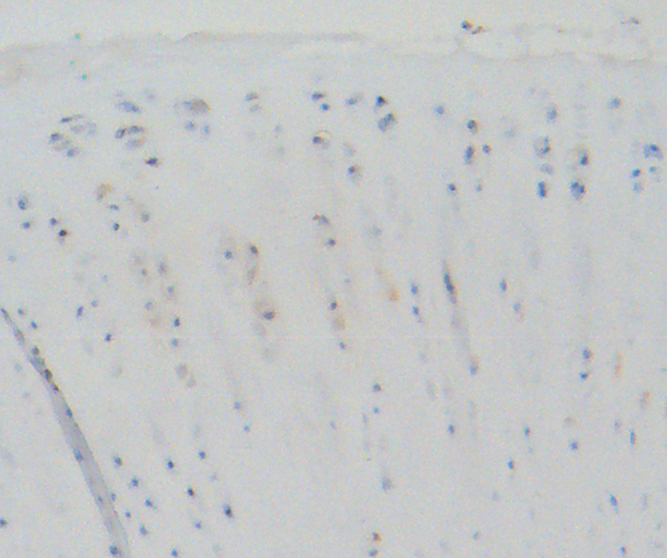

Supplement: Supplementary file 9 [file DataSheet_8.zip › MMP13/Exo+Fer-1/1.jpg]

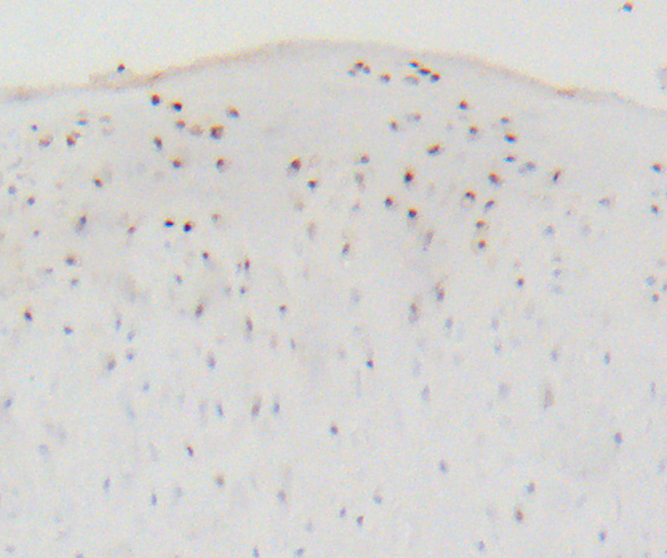

Supplement: Supplementary file 9 [file DataSheet_8.zip › MMP13/Exo+Fer-1/2.jpg]

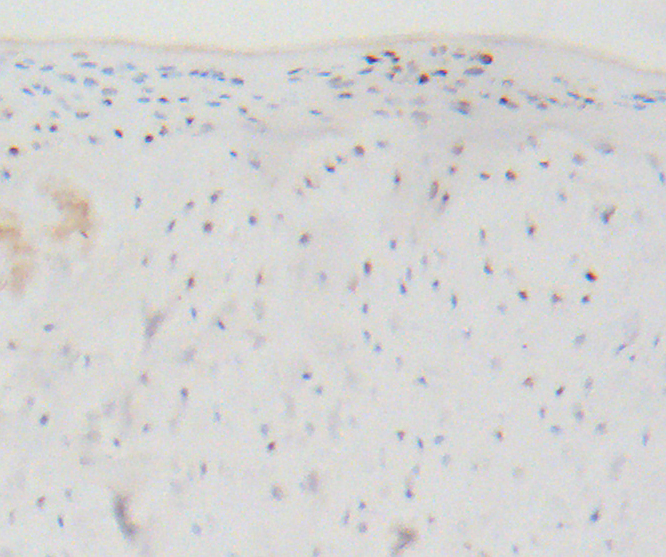

Supplement: Supplementary file 9 [file DataSheet_8.zip › MMP13/Exo+Fer-1/3.jpg]

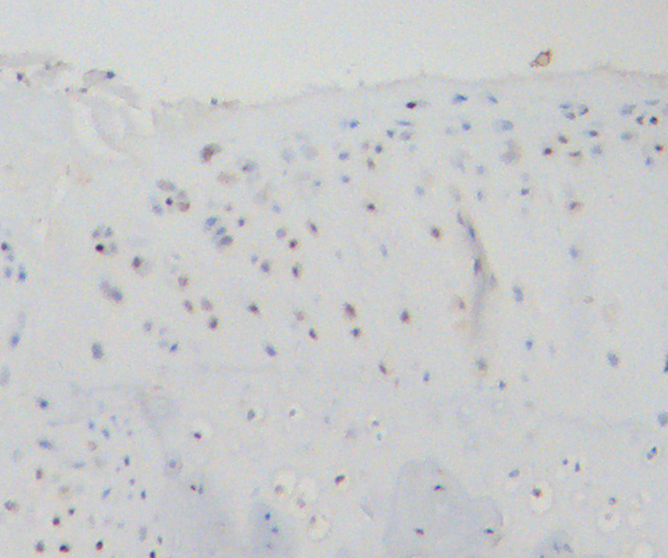

Supplement: Supplementary file 9 [file DataSheet_8.zip › MMP13/Exo+Fer-1/4.jpg]

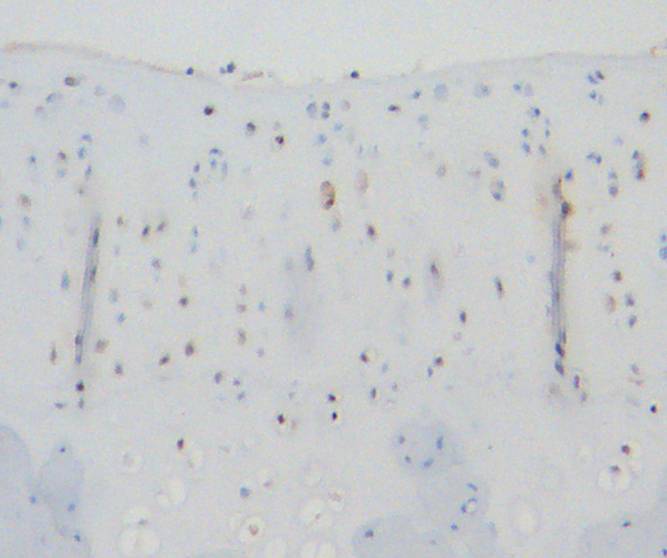

Supplement: Supplementary file 9 [file DataSheet_8.zip › MMP13/Exo+Fer-1/5.jpg]

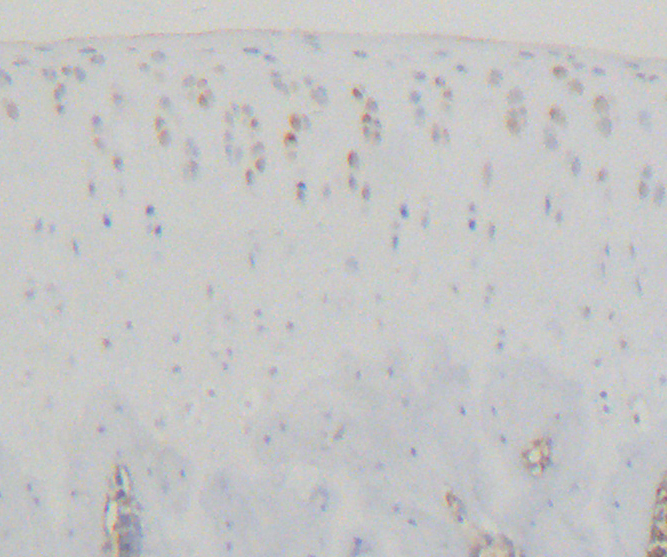

Supplement: Supplementary file 9 [file DataSheet_8.zip › MMP13/Exo+Fer-1/6.jpg]

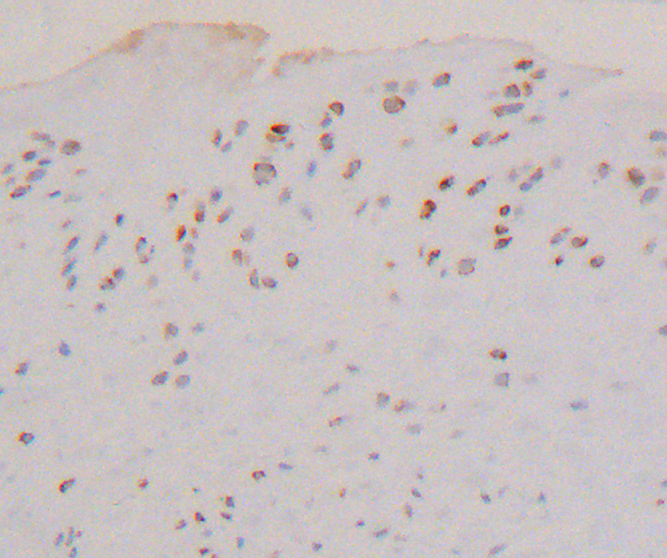

Supplement: Supplementary file 9 [file DataSheet_8.zip › MMP13/miR(-) Exo/1.jpg]

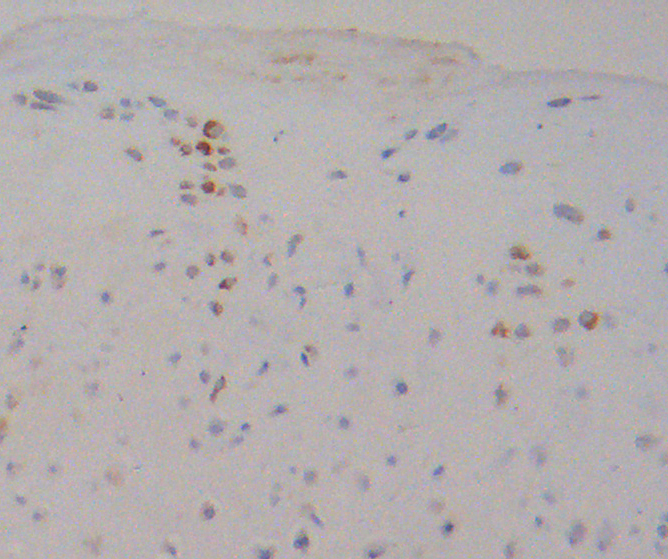

Supplement: Supplementary file 9 [file DataSheet_8.zip › MMP13/miR(-) Exo/2.jpg]

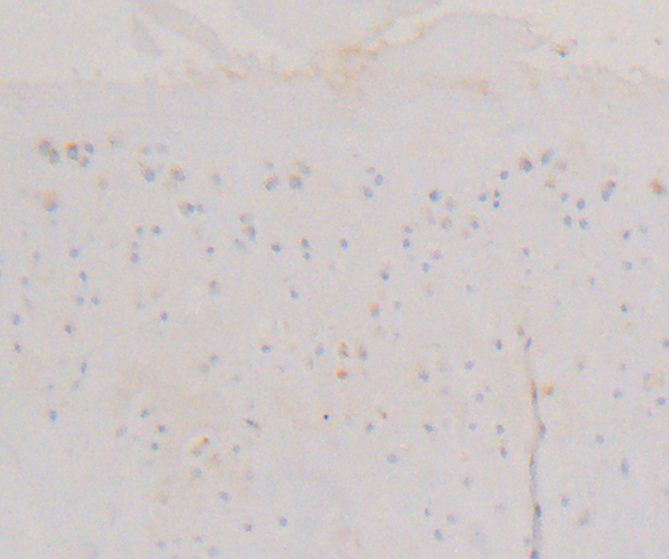

Supplement: Supplementary file 9 [file DataSheet_8.zip › MMP13/miR(-) Exo/4.jpg]

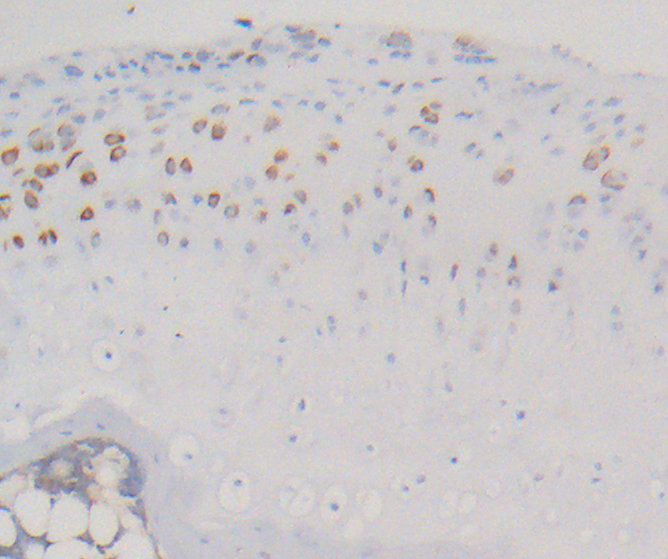

Supplement: Supplementary file 9 [file DataSheet_8.zip › MMP13/miR(-) Exo/5.jpg]

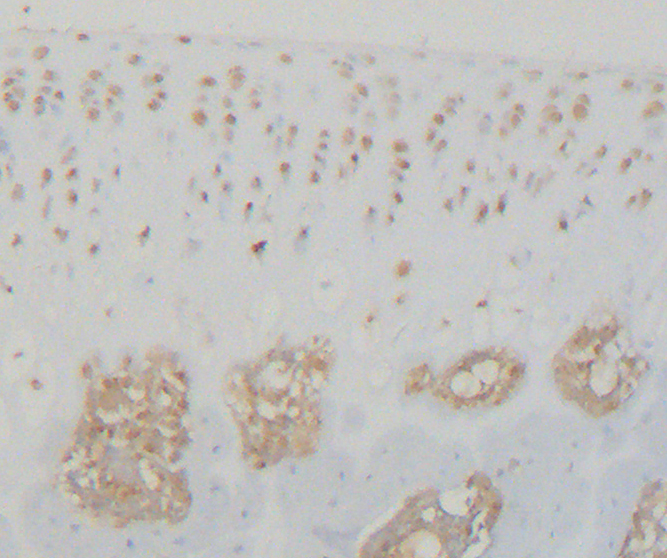

Supplement: Supplementary file 9 [file DataSheet_8.zip › MMP13/miR(-) Exo/6.jpg]

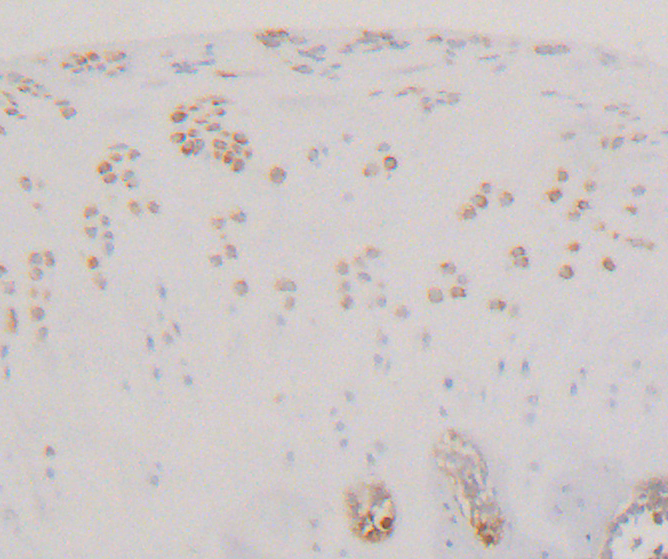

Supplement: Supplementary file 9 [file DataSheet_8.zip › MMP13/miR(-) Exo/776R-4╕▒▒╛.jpg]

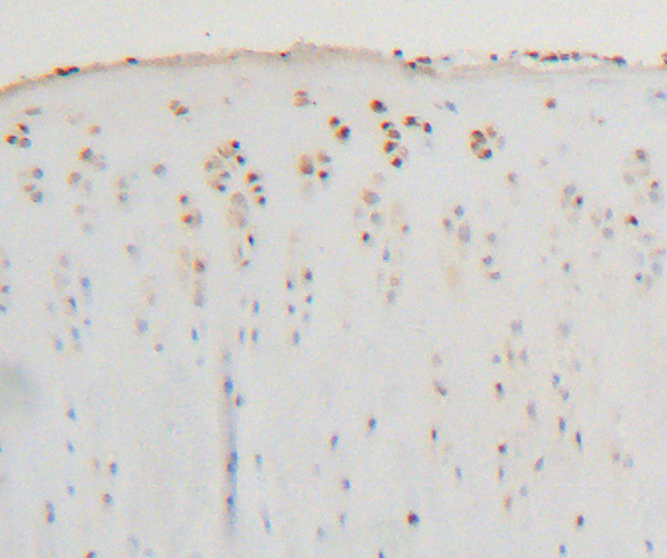

Supplement: Supplementary file 9 [file DataSheet_8.zip › MMP13/Model/1.jpg]

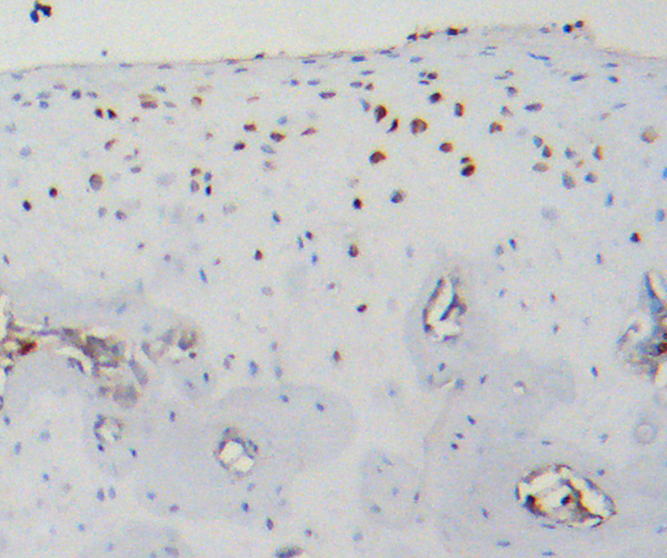

Supplement: Supplementary file 9 [file DataSheet_8.zip › MMP13/Model/2.jpg]

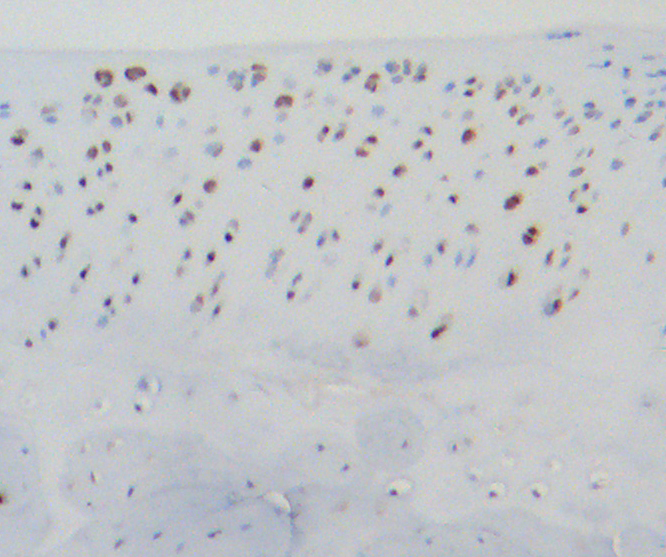

Supplement: Supplementary file 9 [file DataSheet_8.zip › MMP13/Model/3.jpg]

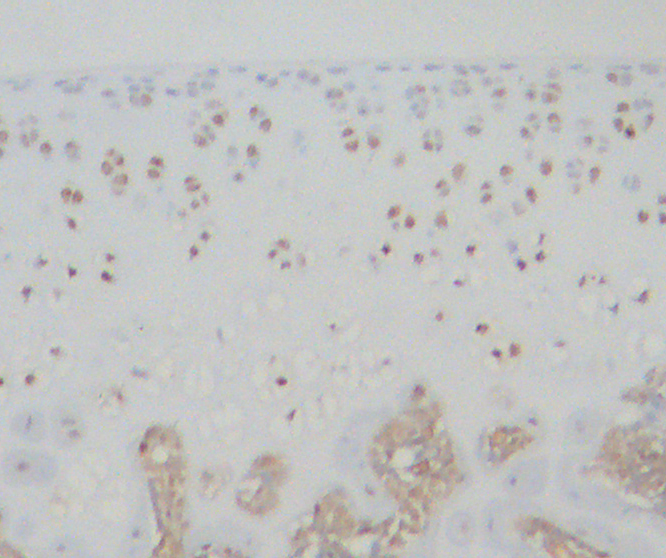

Supplement: Supplementary file 9 [file DataSheet_8.zip › MMP13/Model/4.jpg]

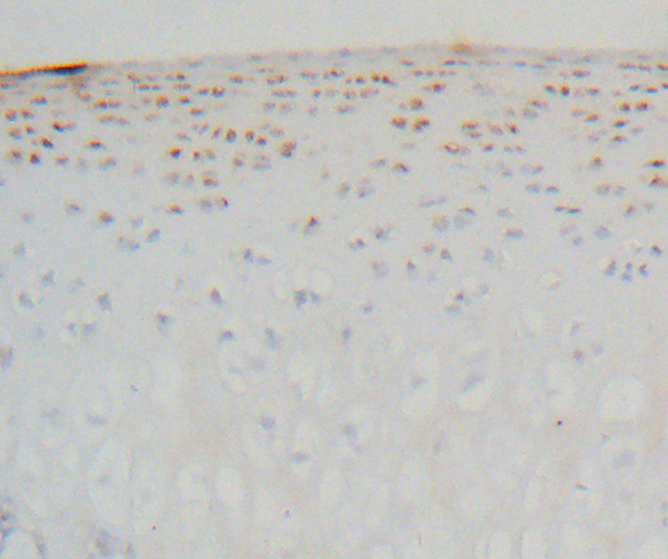

Supplement: Supplementary file 9 [file DataSheet_8.zip › MMP13/Model/5.jpg]

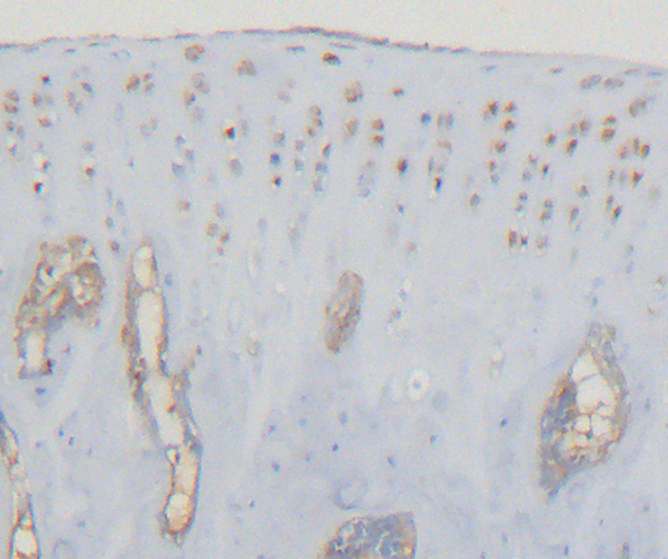

Supplement: Supplementary file 9 [file DataSheet_8.zip › MMP13/Model/6.jpg]

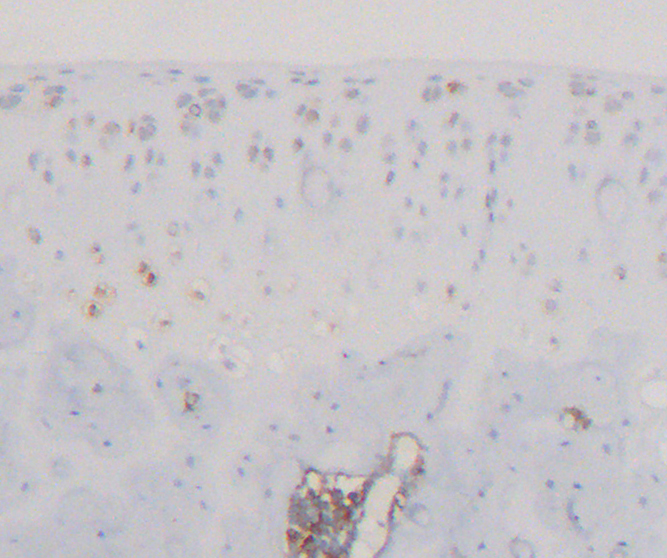

Supplement: Supplementary file 9 [file DataSheet_8.zip › MMP13/Sham/1.jpg]

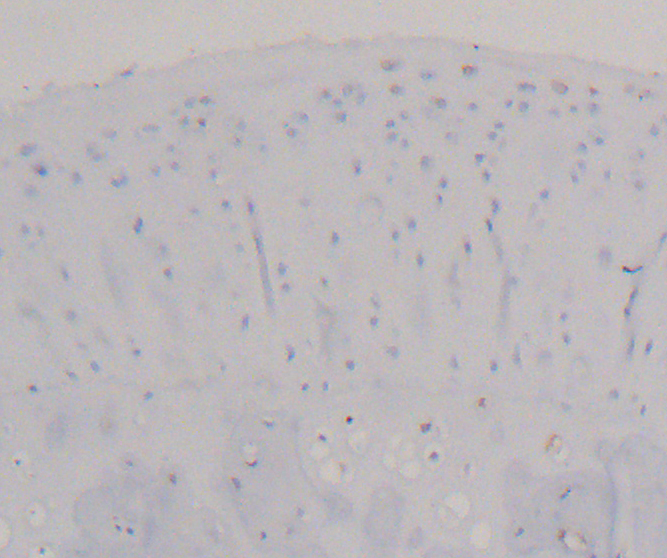

Supplement: Supplementary file 9 [file DataSheet_8.zip › MMP13/Sham/2.jpg]

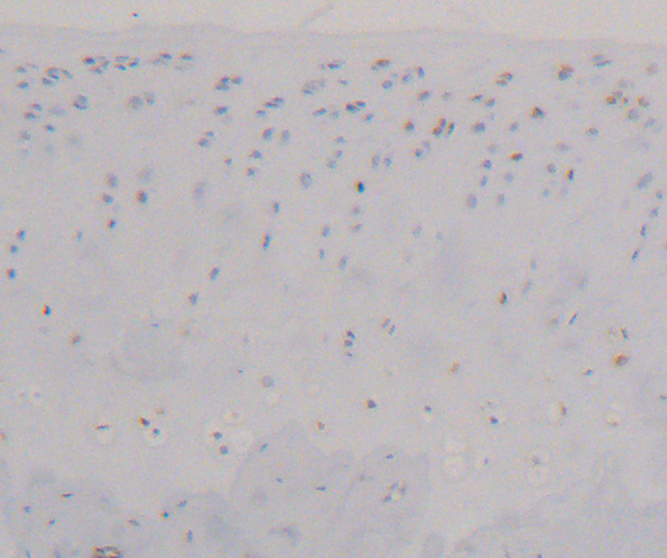

Supplement: Supplementary file 9 [file DataSheet_8.zip › MMP13/Sham/3.jpg]

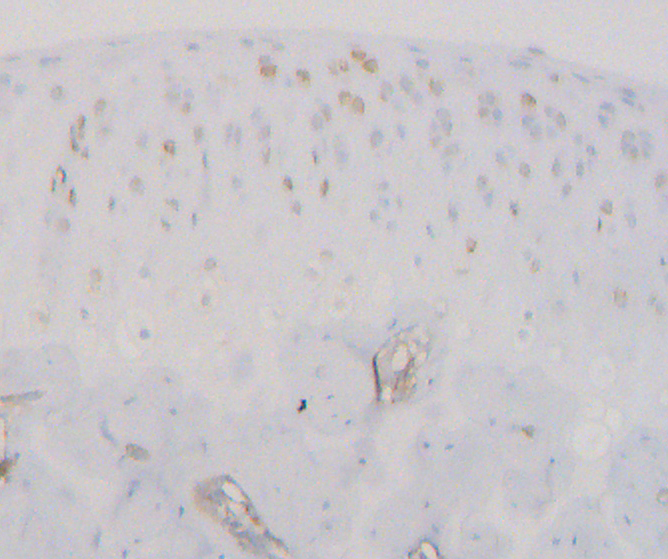

Supplement: Supplementary file 9 [file DataSheet_8.zip › MMP13/Sham/4.jpg]
