# Supplementary figures and images for: Exosomes from osteoarthritic fibroblast-like synoviocytes promote cartilage ferroptosis and damage via delivering microRNA-19b-3p to target SLC7A11 in osteoarthritis (part 6 of 6)
Source: Front Immunol. 2023 Aug 24;14:1181156. doi: 10.3389/fimmu.2023.1181156 (PMC10484587; doi:10.3389/fimmu.2023.1181156)

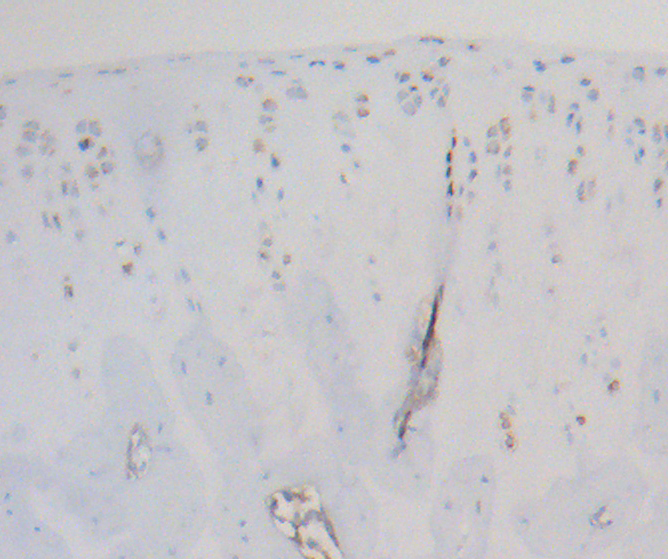

Supplement: Supplementary file 9 [file DataSheet_8.zip › MMP13/Sham/5.jpg]

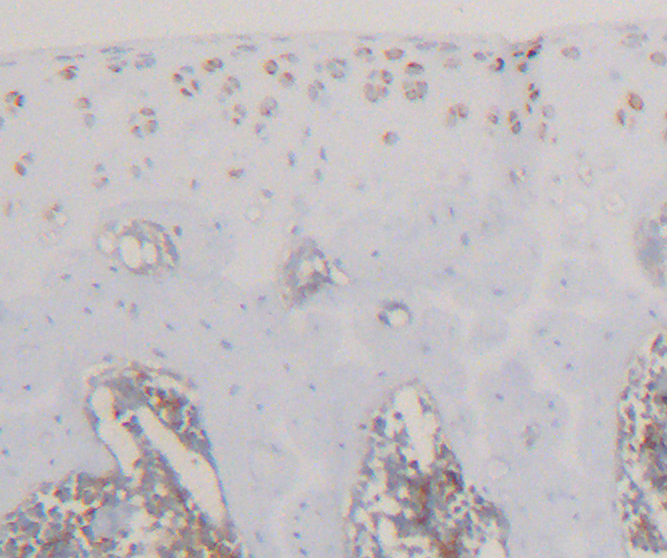

Supplement: Supplementary file 9 [file DataSheet_8.zip › MMP13/Sham/6.jpg]

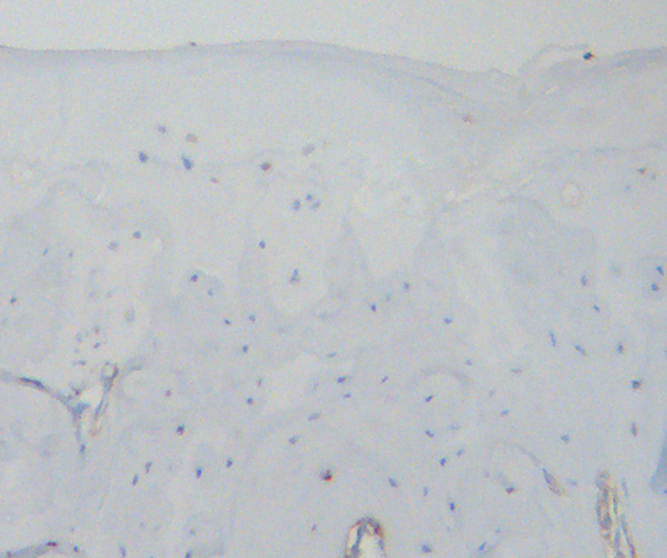

Supplement: Supplementary file 10 [file DataSheet_9.zip › SLC7A11/Exo/1.jpg]

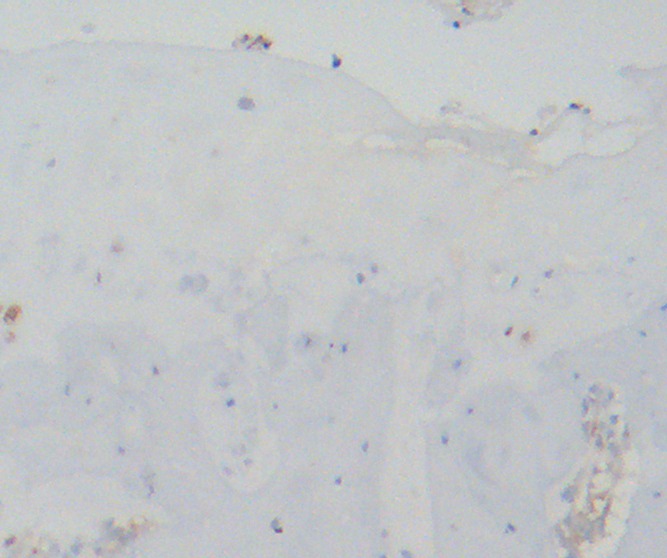

Supplement: Supplementary file 10 [file DataSheet_9.zip › SLC7A11/Exo/2.jpg]

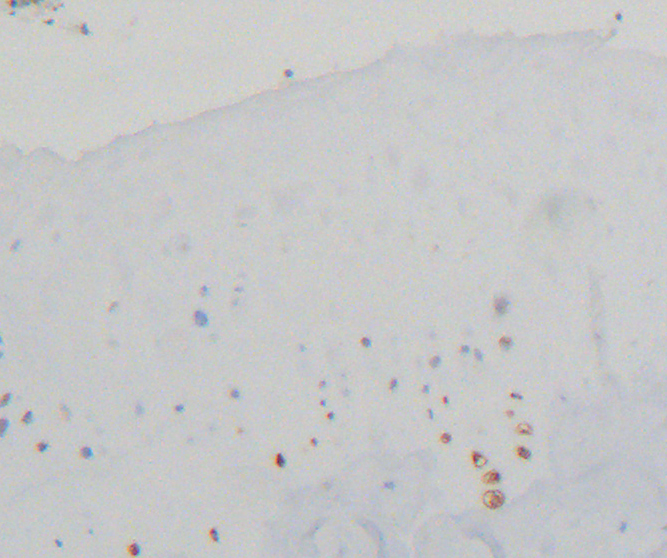

Supplement: Supplementary file 10 [file DataSheet_9.zip › SLC7A11/Exo/3.jpg]

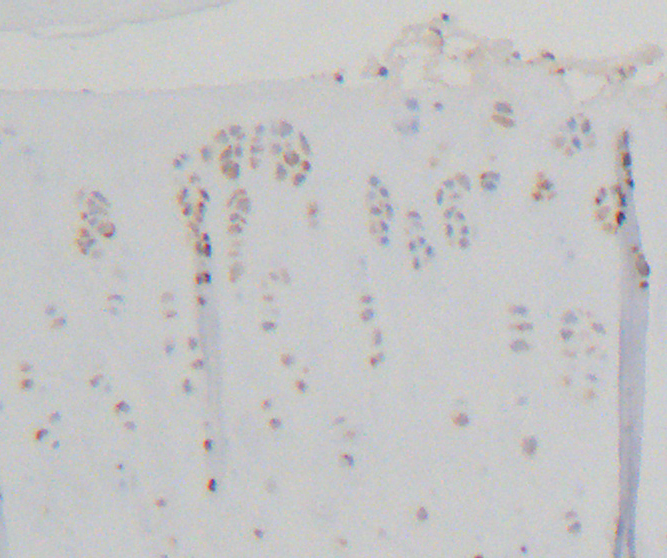

Supplement: Supplementary file 10 [file DataSheet_9.zip › SLC7A11/Exo/4.jpg]

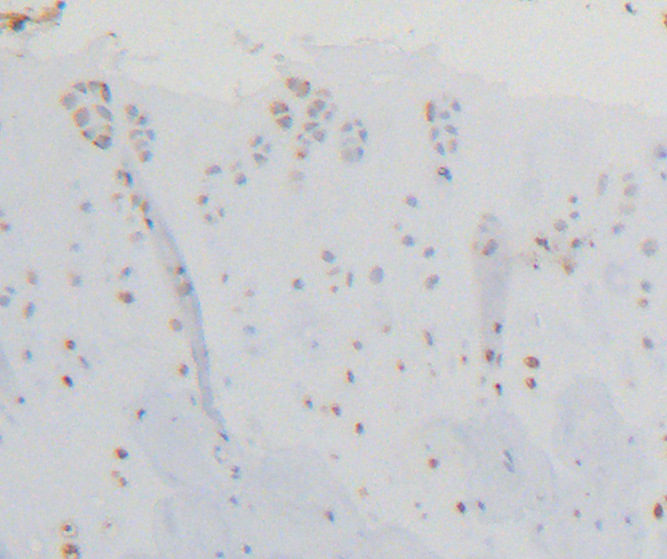

Supplement: Supplementary file 10 [file DataSheet_9.zip › SLC7A11/Exo/5.jpg]

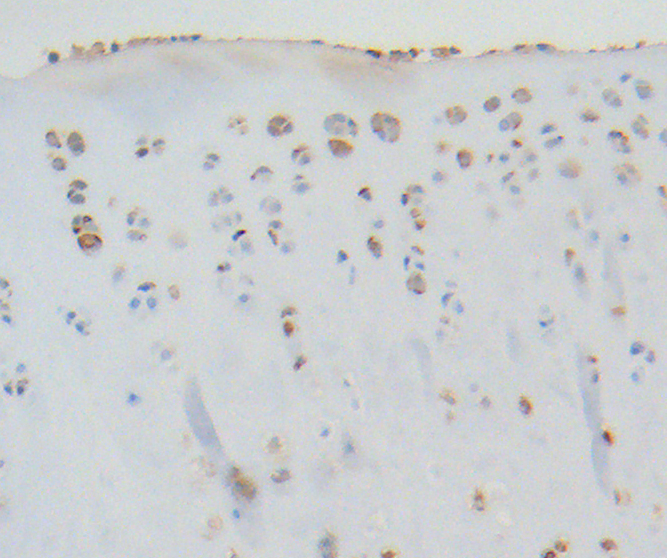

Supplement: Supplementary file 10 [file DataSheet_9.zip › SLC7A11/Exo/6.jpg]

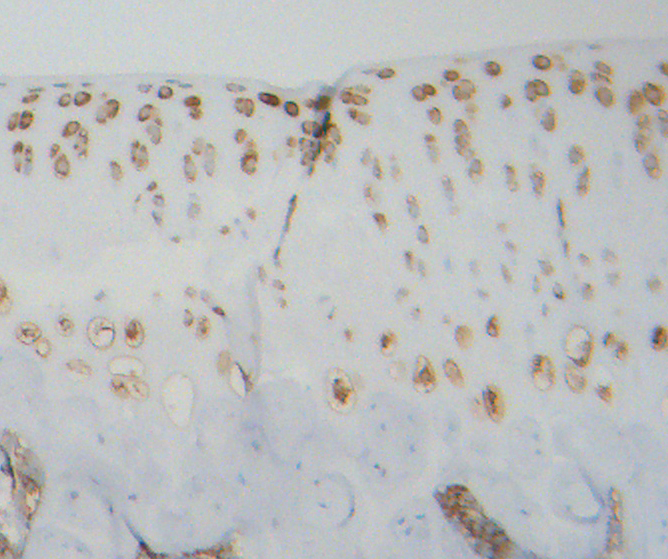

Supplement: Supplementary file 10 [file DataSheet_9.zip › SLC7A11/Exo+Fer-1/1.jpg]

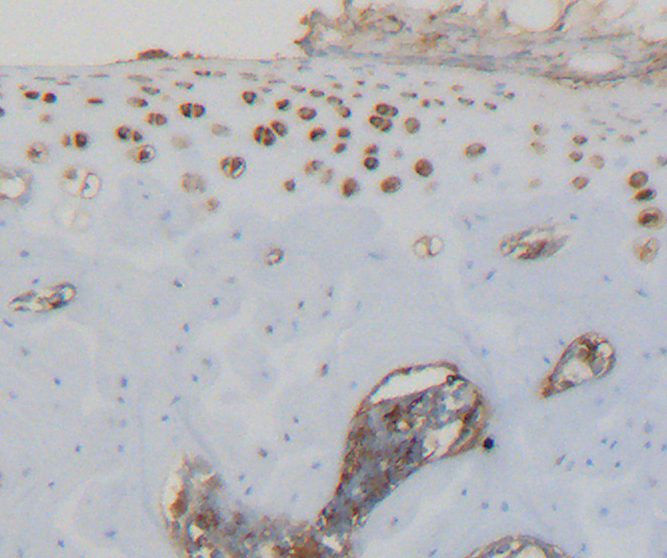

Supplement: Supplementary file 10 [file DataSheet_9.zip › SLC7A11/Exo+Fer-1/2.jpg]

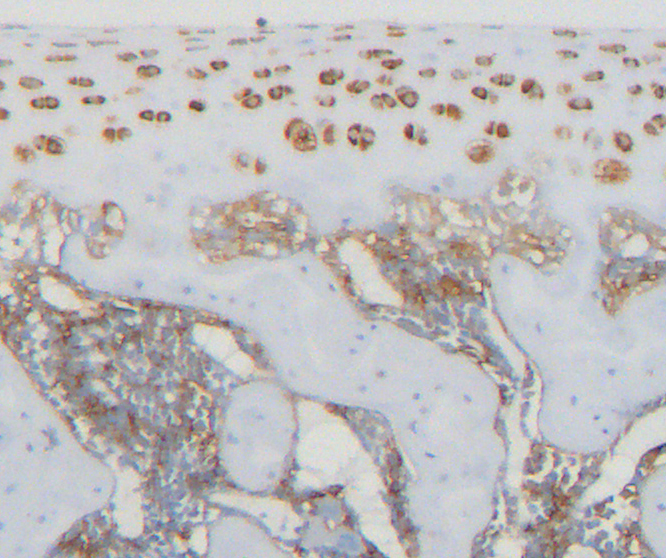

Supplement: Supplementary file 10 [file DataSheet_9.zip › SLC7A11/Exo+Fer-1/3.jpg]

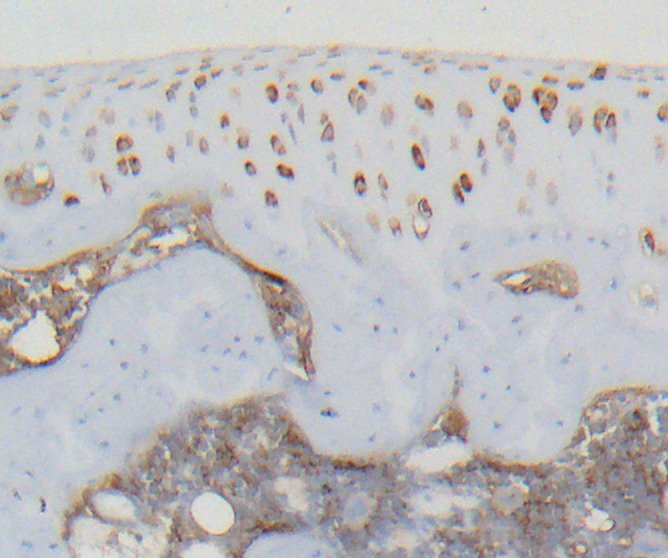

Supplement: Supplementary file 10 [file DataSheet_9.zip › SLC7A11/Exo+Fer-1/4.jpg]

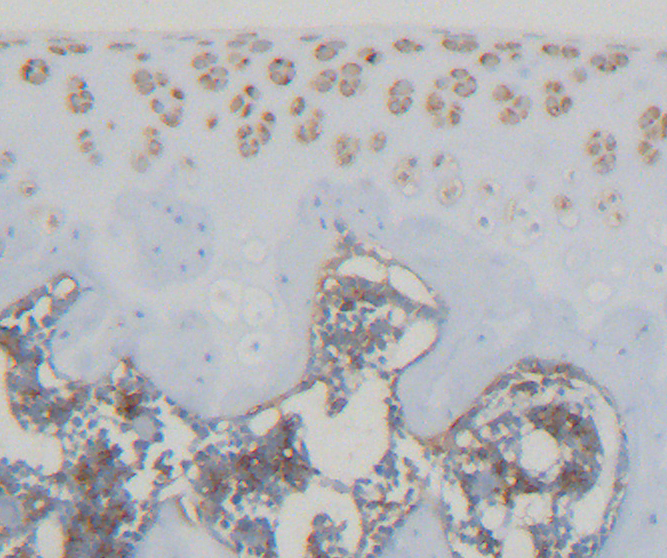

Supplement: Supplementary file 10 [file DataSheet_9.zip › SLC7A11/Exo+Fer-1/5.jpg]

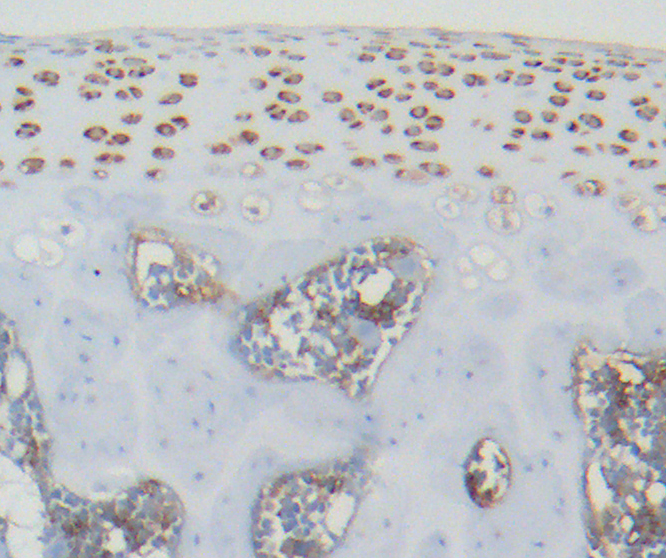

Supplement: Supplementary file 10 [file DataSheet_9.zip › SLC7A11/Exo+Fer-1/6.jpg]

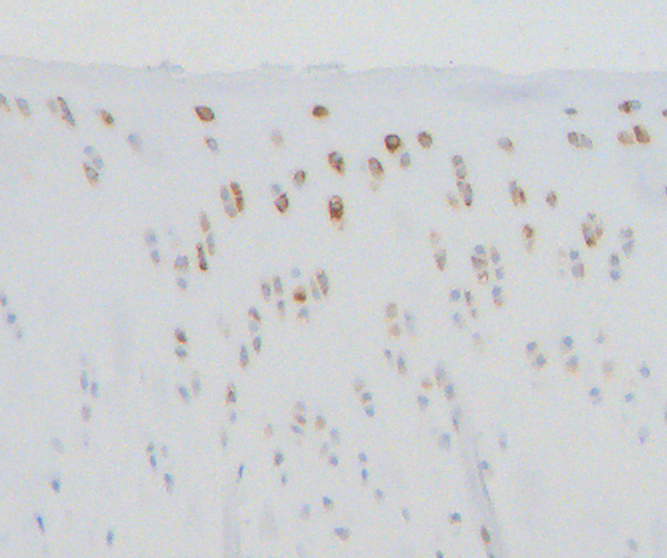

Supplement: Supplementary file 10 [file DataSheet_9.zip › SLC7A11/miR(-) Exo/1.jpg]

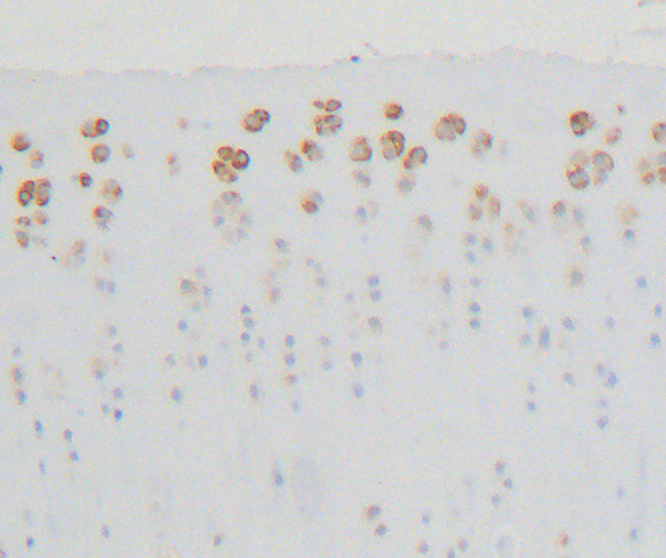

Supplement: Supplementary file 10 [file DataSheet_9.zip › SLC7A11/miR(-) Exo/2.jpg]

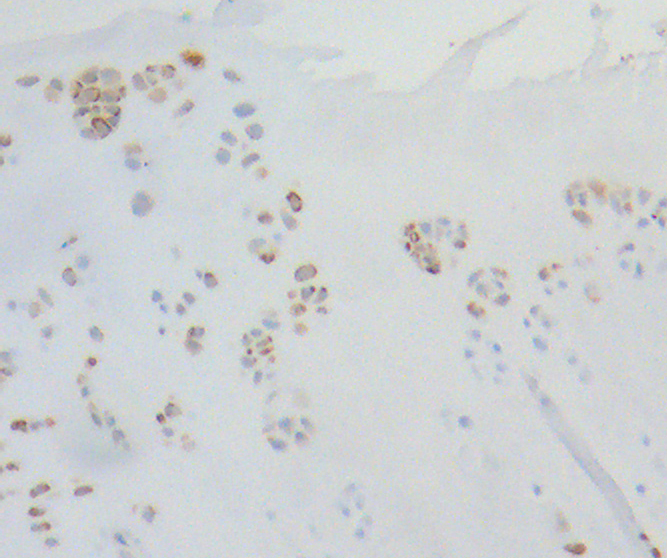

Supplement: Supplementary file 10 [file DataSheet_9.zip › SLC7A11/miR(-) Exo/3.jpg]

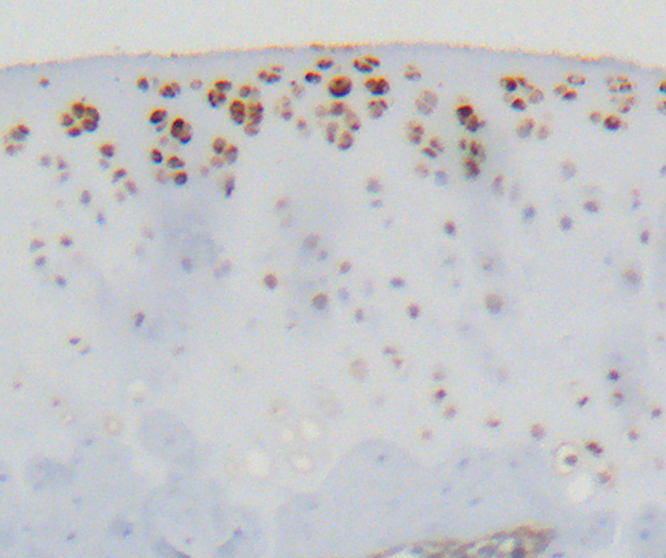

Supplement: Supplementary file 10 [file DataSheet_9.zip › SLC7A11/miR(-) Exo/4.jpg]

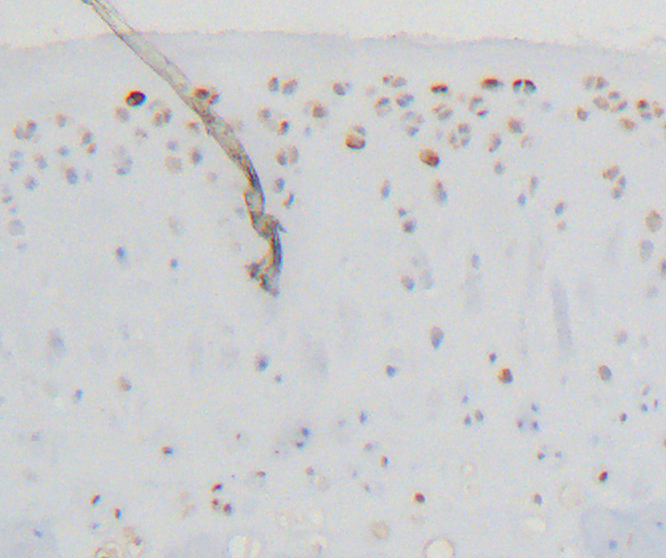

Supplement: Supplementary file 10 [file DataSheet_9.zip › SLC7A11/miR(-) Exo/5.jpg]

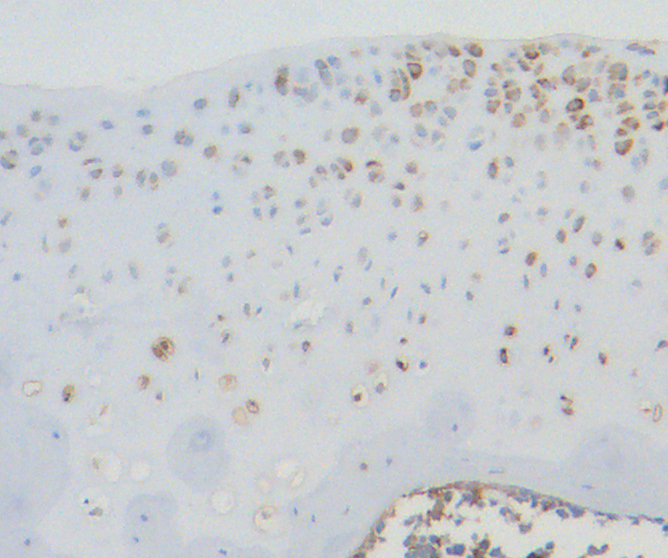

Supplement: Supplementary file 10 [file DataSheet_9.zip › SLC7A11/miR(-) Exo/6.jpg]

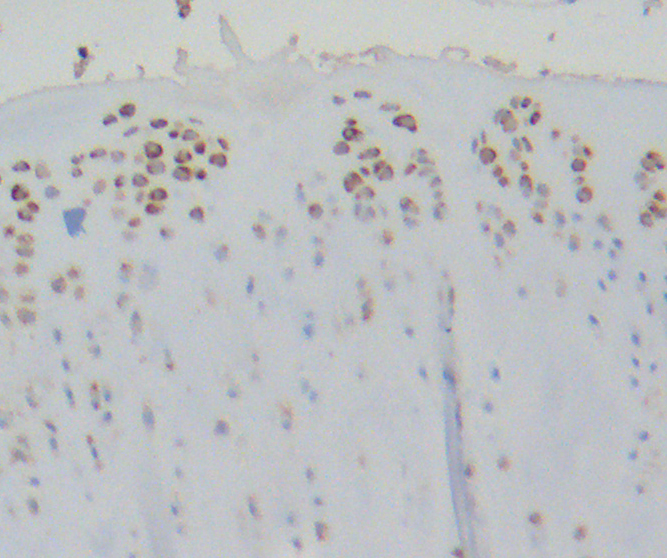

Supplement: Supplementary file 10 [file DataSheet_9.zip › SLC7A11/Model/1.jpg]

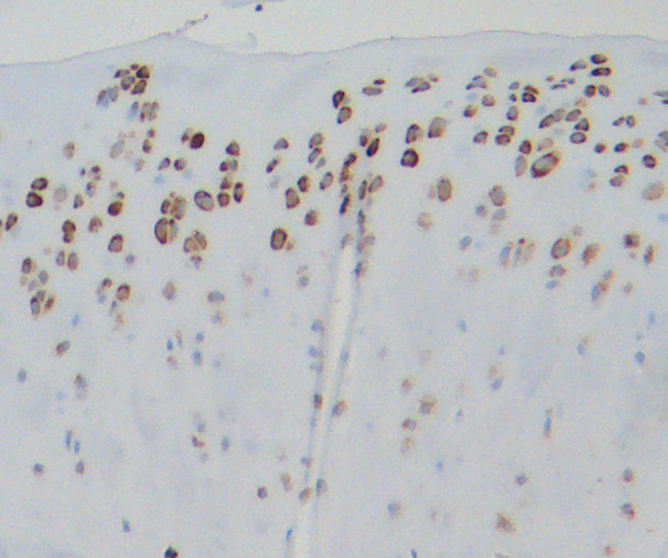

Supplement: Supplementary file 10 [file DataSheet_9.zip › SLC7A11/Model/2.jpg]

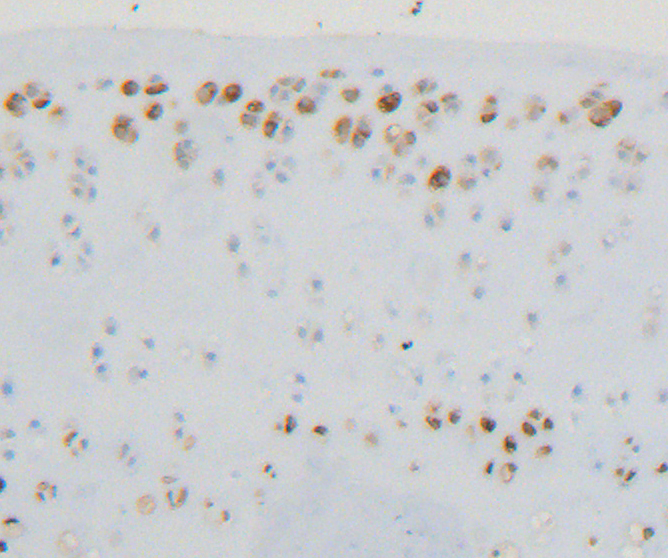

Supplement: Supplementary file 10 [file DataSheet_9.zip › SLC7A11/Model/3.jpg]

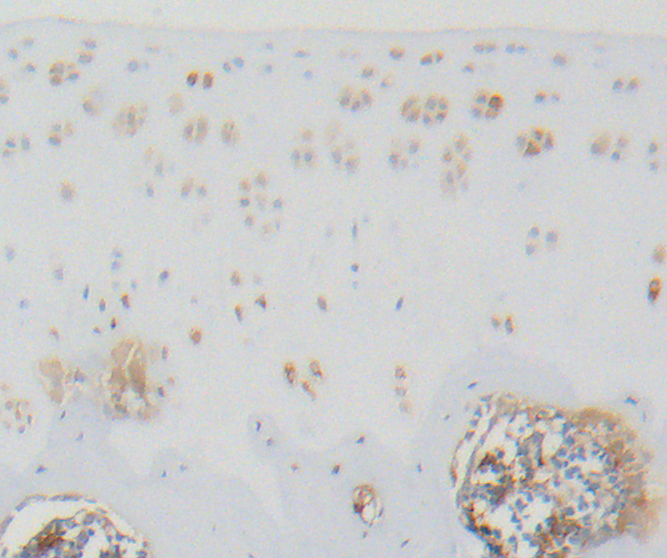

Supplement: Supplementary file 10 [file DataSheet_9.zip › SLC7A11/Model/4.jpg]

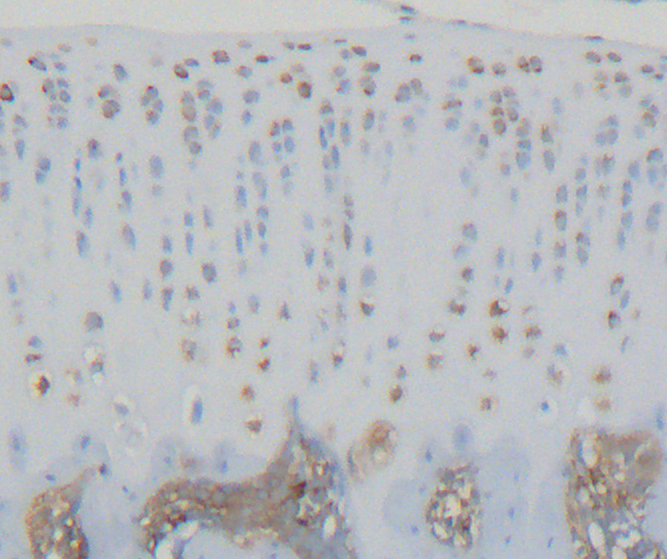

Supplement: Supplementary file 10 [file DataSheet_9.zip › SLC7A11/Model/5.jpg]

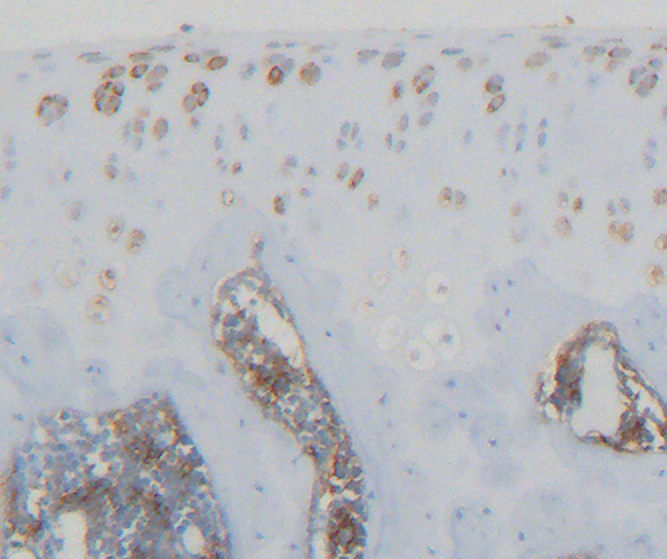

Supplement: Supplementary file 10 [file DataSheet_9.zip › SLC7A11/Model/6.jpg]

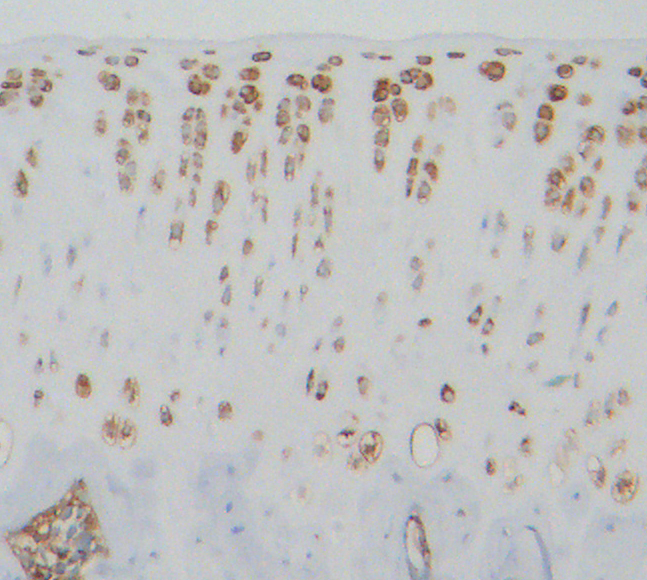

Supplement: Supplementary file 10 [file DataSheet_9.zip › SLC7A11/Sham/1.jpg]

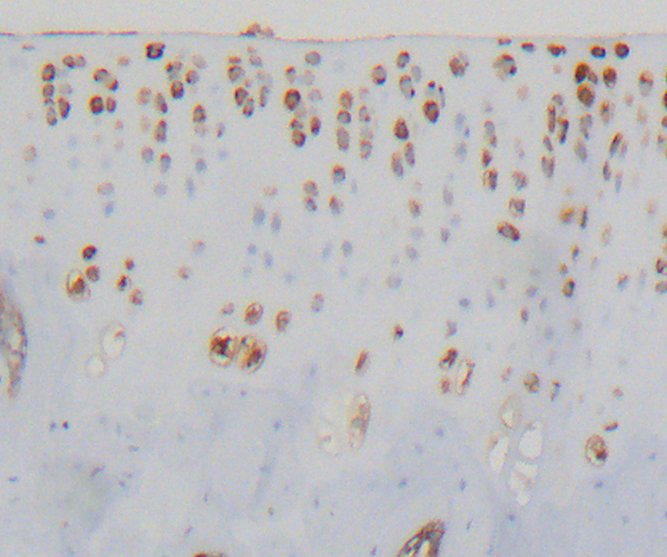

Supplement: Supplementary file 10 [file DataSheet_9.zip › SLC7A11/Sham/2.jpg]

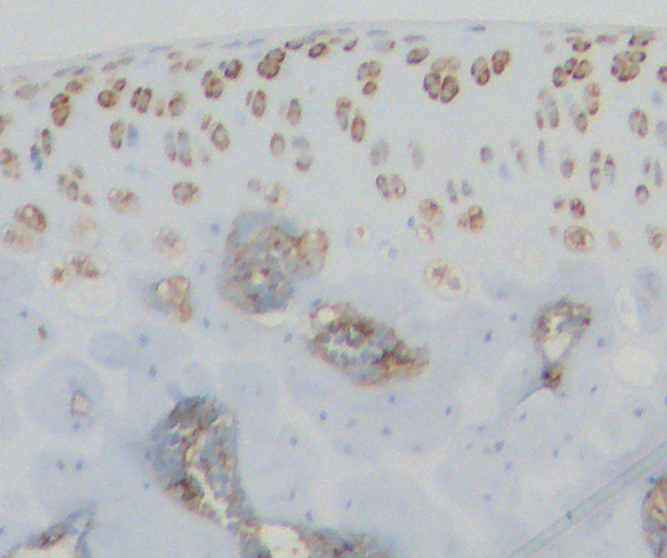

Supplement: Supplementary file 10 [file DataSheet_9.zip › SLC7A11/Sham/3.jpg]

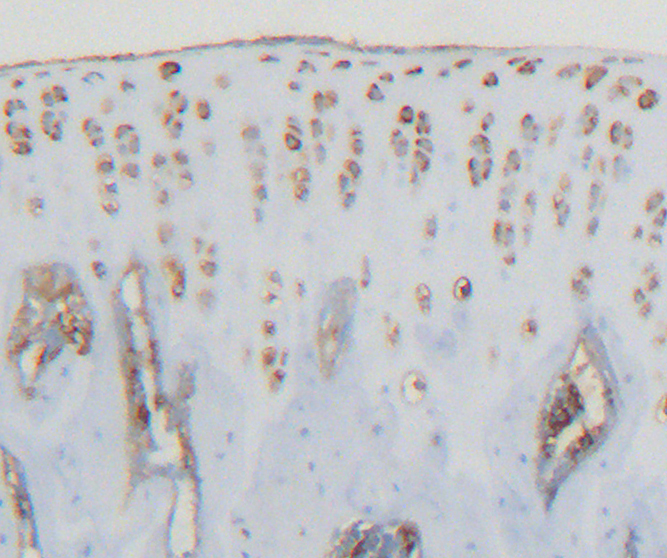

Supplement: Supplementary file 10 [file DataSheet_9.zip › SLC7A11/Sham/4.jpg]

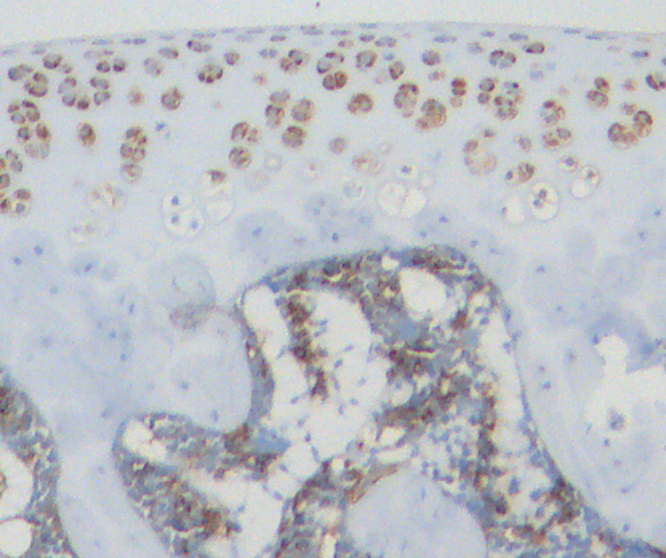

Supplement: Supplementary file 10 [file DataSheet_9.zip › SLC7A11/Sham/5.jpg]

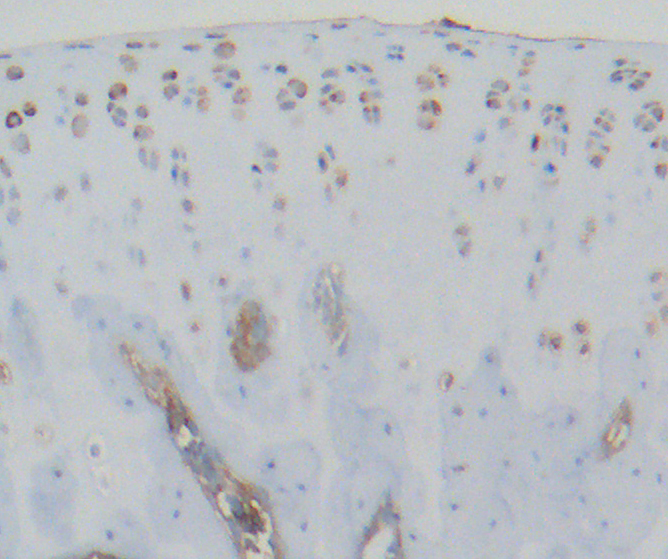

Supplement: Supplementary file 10 [file DataSheet_9.zip › SLC7A11/Sham/6.jpg]
